# Supplementary material for: Arsenite Exposure to Human RPCs (HRTPT) Produces a Reversible Epithelial Mesenchymal Transition (EMT): In-Vitro and In-Silico Study
Source: Int J Mol Sci. 2023 Mar 7;24(6):5092. doi: 10.3390/ijms24065092 (PMC10048886; doi:10.3390/ijms24065092)
Supplement: Supplementary file 1 [file ijms-24-05092-s001.zip › Supplementary Tables.pdf]

Supplementary Table S1: The list of most significant genes (p<0.05) using ANOVA across all possible conditions (i.e., Control, P3, P8, P10, p2, P11).

	probe	PublicGeneIDs	HUGO.gene.symbol	EntrezGene.ID	f.value	p.value	X.log10.p.	FDR	Fisher.s.LSD
TC0700006913.hg.1	TC0700006913.hg.1	NM_001005340	GPNMB	10457	2734.4	6.66E-18	17.176	1.66E-14	ctrl - P10; P11 - ctrl; ctrl - P2; ctrl - P3; ctrl - P8; P11 - P10; P2 - P10; P3 - P10; P8 - P10; P11 - P2; P11 - P3; P11 - P8; P2 - P3; P2 - P8; P3 - P8
TC1100006995.hg.1	TC1100006995.hg.1	NM_000331	SAA1	6288	1095.1	1.60E-15	14.796	2.00E-12	P10 - ctrl; ctrl - P11; ctrl - P2; P3 - ctrl; P8 - ctrl; P10 - P11; P10 - P2; P10 - P3; P8 - P10; P11 - P2; P3 - P11; P8 - P11; P3 - P2; P8 - P2; P8 - P3
TC1600007235.hg.1	TC1600007235.hg.1	NM_005030	PLK1	5347	992.68	2.88E-15	14.541	2.09E-12	P10 - ctrl; ctrl - P11; ctrl - P2; P8 - ctrl; P10 - P11; P10 - P2; P10 - P3; P8 - P10; P11 - P2; P3 - P11; P8 - P11; P3 - P2; P8 - P2; P8 - P3
TC1900009443.hg.1	TC1900009443.hg.1	NM_000064	C3	718	968.24	3.34E-15	14.476	2.09E-12	P10 - ctrl; P3 - ctrl; P8 - ctrl; P10 - P11; P10 - P2; P10 - P3; P3 - P11; P8 - P11; P3 - P2; P8 - P2; P8 - P3
TC1500010739.hg.1	TC1500010739.hg.1	NM_000338	SLC12A1	chloride transporter), member 1	877.5	6.02E-15	14.221	3.01E-12	ctrl - P10; ctrl - P2; ctrl - P3; ctrl - P8; P11 - P10; P2 - P10; P3 - P10; P8 - P10; P11 - P2; P11 - P3; P11 - P8; P2 - P3; P2 - P8; P3 - P8
TC1200009834.hg.1	TC1200009834.hg.1	NM_001297709	MFAP5	8076	710.86	2.12E-14	13.674	7.72E-12	P10 - ctrl; P11 - ctrl; P2 - ctrl; P3 - ctrl; P8 - ctrl; P10 - P11; P10 - P2; P2 - P11; P3 - P11; P8 - P11; P3 - P2; P8 - P2; P8 - P3
TC0900008847.hg.1	TC0900008847.hg.1	NM_005564	LCN2	3934	688.85	2.56E-14	13.592	7.72E-12	P10 - ctrl; P11 - ctrl; P3 - ctrl; P8 - ctrl; P10 - P11; P10 - P2; P10 - P3; P11 - P2; P3 - P11; P8 - P11; P3 - P2; P8 - P2; P8 - P3
TC0200007418.hg.1	TC0200007418.hg.1	NM_000341	SLC3A1	6519	686.87	2.60E-14	13.585	7.72E-12	ctrl - P10; P11 - ctrl; ctrl - P2; ctrl - P3; ctrl - P8; P11 - P10; P2 - P10; P10 - P3; P10 - P8; P11 - P2; P11 - P3; P11 - P8; P2 - P3; P2 - P8; P8 - P3
TC0300012768.hg.1	TC0300012768.hg.1	NM_000096	CP	1356	679.28	2.78E-14	13.556	7.72E-12	P10 - ctrl; P3 - ctrl; P8 - ctrl; P10 - P11; P10 - P2; P10 - P3; P2 - P11; P3 - P11; P8 - P11; P3 - P2; P8 - P2; P8 - P3
TC0400011180.hg.1	TC0400011180.hg.1	NM_001037582	SCD5	79966	640.57	3.95E-14	13.404	9.86E-12	ctrl - P10; ctrl - P11; ctrl - P2; ctrl - P3; ctrl - P8; P11 - P10; P2 - P10; P3 - P10; P8 - P10; P11 - P2; P11 - P3; P11 - P8; P3 - P2; P3 - P8
TC0700012754.hg.1	TC0700012754.hg.1	NM_020632	ATP6V0A4	50617	569.46	7.97E-14	13.099	1.81E-11	ctrl - P10; P11 - ctrl; ctrl - P2; ctrl - P3; ctrl - P8; P11 - P10; P2 - P10; P3 - P10; P11 - P2; P11 - P3; P11 - P8; P2 - P3; P2 - P8; P3 - P8
TC0500012470.hg.1	TC0500012470.hg.1	NM_001025158	CD74	972	551.47	9.65E-14	13.015	2.01E-11	P10 - ctrl; P3 - ctrl; P8 - ctrl; P10 - P11; P10 - P2; P10 - P3; P3 - P11; P8 - P11; P3 - P2; P8 - P2; P8 - P3
TC0600014273.hg.1	TC0600014273.hg.1	NM_001243965	HLA-DRB1	3123	498.82	1.76E-13	12.755	3.38E-11	P10 - ctrl; P3 - ctrl; P8 - ctrl; P10 - P11; P10 - P2; P10 - P3; P8 - P11; P8 - P2; P8 - P3
TC0200009067.hg.1	TC0200009067.hg.1	NM_183240	TMEM37	140738	469.63	2.52E-13	12.599	4.49E-11	ctrl - P10; P11 - ctrl; ctrl - P2; ctrl - P3; ctrl - P8; P11 - P10; P2 - P10; P10 - P3; P10 - P8; P11 - P2; P11 - P3; P11 - P8; P2 - P3; P2 - P8; P8 - P3
TC1100013152.hg.1	TC1100013152.hg.1	NM_001127380	SAA2	6289	456.79	2.97E-13	12.527	4.95E-11	P10 - ctrl; ctrl - P11; ctrl - P2; P3 - ctrl; P8 - ctrl; P10 - P11; P10 - P2; P10 - P3; P11 - P2; P3 - P11; P8 - P11; P3 - P2; P8 - P2; P8 - P3
TC0100017084.hg.1	TC0100017084.hg.1	NM_001910	CTSE	1510	447.6	3.35E-13	12.475	5.23E-11	P10 - ctrl; P11 - ctrl; P2 - ctrl; P3 - ctrl; P8 - ctrl; P10 - P11; P10 - P2; P10 - P3; P8 - P10; P3 - P11; P8 - P11; P3 - P2; P8 - P2; P8 - P3
TC0900010106.hg.1	TC0900010106.hg.1	NA	NA	NA	441.2	3.65E-13	12.437	5.37E-11	ctrl - P10; ctrl - P11; P2 - ctrl; P3 - ctrl; ctrl - P8; P10 - P11; P2 - P10; P3 - P10; P10 - P8; P2 - P11; P3 - P11; P8 - P11; P2 - P3; P2 - P8; P3 - P8
TC1500006925.hg.1	TC1500006925.hg.1	NM_003246	THBS1	7057	435.34	3.96E-13	12.403	5.49E-11	P10 - ctrl; ctrl - P11; ctrl - P2; P3 - ctrl; P8 - ctrl; P10 - P11; P10 - P2; P3 - P10; P8 - P10; P3 - P11; P8 - P11; P3 - P2; P8 - P2; P8 - P3
TC0100017310.hg.1	TC0100017310.hg.1	NM_001134285	ESRRG	2104	423.83	4.64E-13	12.334	6.10E-11	ctrl - P10; P11 - ctrl; ctrl - P2; ctrl - P3; ctrl - P8; P11 - P10; P2 - P10; P10 - P3; P10 - P8; P11 - P2; P11 - P3; P11 - P8; P2 - P3; P2 - P8
TC1200006730.hg.1	TC1200006730.hg.1	NM_001297776	RIMKLB	57494	412.56	5.45E-13	12.264	6.81E-11	P10 - ctrl; ctrl - P11; P2 - ctrl; P3 - ctrl; P8 - ctrl; P10 - P11; P10 - P3; P10 - P8; P2 - P11; P3 - P11; P8 - P11; P2 - P3; P2 - P8; P8 - P3
TC0200010627.hg.1	TC0200010627.hg.1	NM_001309516	PTH2R	5746	405.61	6.03E-13	12.22	7.17E-11	ctrl - P10; ctrl - P11; ctrl - P2; ctrl - P3; ctrl - P8; P11 - P10; P2 - P10; P3 - P10; P11 - P2; P11 - P3; P11 - P8; P2 - P3; P2 - P8; P3 - P8
TC1700006999.hg.1	TC1700006999.hg.1	NM_000676	ADORA2B	136	391.12	7.49E-13	12.126	8.08E-11	P10 - ctrl; ctrl - P2; P3 - ctrl; P8 - ctrl; P10 - P11; P10 - P2; P8 - P10; P11 - P2; P3 - P11; P8 - P11; P3 - P2; P8 - P2; P8 - P3
TC2000009058.hg.1	TC2000009058.hg.1	NM_004613	TGM2	7052	389.66	7.65E-13	12.116	8.08E-11	P10 - ctrl; ctrl - P2; P3 - ctrl; P8 - ctrl; P10 - P11; P10 - P2; P8 - P10; P11 - P2; P3 - P11; P8 - P11; P3 - P2; P8 - P2; P8 - P3
TC0200013861.hg.1	TC0200013861.hg.1	NM_005434	MALL	7851	388.8	7.76E-13	12.11	8.08E-11	P10 - ctrl; ctrl - P2; P3 - ctrl; P8 - ctrl; P10 - P11; P10 - P2; P3 - P10; P8 - P10; P11 - P2; P3 - P11; P8 - P11; P3 - P2; P8 - P2; P8 - P3
TC0100014988.hg.1	TC0100014988.hg.1	NM_001178096	F3	2152	384	8.35E-13	12.078	8.35E-11	P10 - ctrl; P3 - ctrl; P8 - ctrl; P10 - P11; P10 - P2; P10 - P3; P8 - P10; P11 - P2; P3 - P11; P8 - P11; P3 - P2; P8 - P2; P8 - P3
TC0800007888.hg.1	TC0800007888.hg.1	NM_001195639	C8orf34	116328	376.4	9.41E-13	12.027	9.04E-11	P10 - ctrl; ctrl - P11; P2 - ctrl; P3 - ctrl; P8 - ctrl; P10 - P11; P10 - P2; P10 - P3; P2 - P11; P3 - P11; P8 - P11; P3 - P2; P8 - P2; P8 - P3
TC1900011967.hg.1	TC1900011967.hg.1	NM_001301707	PSG9	5678	371.9	1.01E-12	11.995	9.12E-11	P10 - ctrl; ctrl - P11; ctrl - P2; P3 - ctrl; P8 - ctrl; P10 - P11; P10 - P2; P11 - P2; P3 - P11; P8 - P11; P3 - P2; P8 - P2
TC1200008675.hg.1	TC1200008675.hg.1	NM_001093771	TXNRD1	7296	368.3	1.07E-12	11.97	9.12E-11	P10 - ctrl; P11 - ctrl; ctrl - P2; P3 - ctrl; P8 - ctrl; P10 - P11; P10 - P2; P8 - P10; P11 - P2; P3 - P11; P8 - P11; P3 - P2; P8 - P2; P8 - P3
TC2100006834.hg.1	TC2100006834.hg.1	NR_126010	LOC284825	284825	368	1.08E-12	11.968	9.12E-11	P10 - ctrl; P11 - ctrl; P2 - ctrl; P3 - ctrl; P8 - ctrl; P10 - P11; P10 - P2; P10 - P3; P2 - P11; P3 - P11; P8 - P11; P2 - P3; P8 - P2; P8 - P3
TC0700010348.hg.1	TC0700010348.hg.1	NM_015464	SOSTDC1	25928	366.94	1.09E-12	11.961	9.12E-11	ctrl - P10; ctrl - P2; ctrl - P3; ctrl - P8; P11 - P10; P2 - P10; P10 - P3; P11 - P2; P11 - P3; P11 - P8; P2 - P3; P2 - P8; P8 - P3

TC1100007111.hg.1	TC1100007111.hg.1	NM_003986	BBOX1	8424	359.69	1.23E-12	11.909	9.94E-11	ctrl - P10; ctrl - P11; ctrl - P2; ctrl - P3; ctrl - P8; P11 - P10; P2 - P10; P11 - P2; P11 - P3; P11 - P8; P2 - P3; P2 - P8
TC0800007040.hg.1	TC0800007040.hg.1	NM_001304351	ADAM28	10863	355.98	1.31E-12	11.882	1.02E-10	P10 - ctrl; P2 - ctrl; P3 - ctrl; P8 - ctrl; P10 - P11; P10 - P2; P10 - P3; P2 - P11; P3 - P11; P8 - P11; P2 - P3; P8 - P2; P8 - P3
TC1400010618.hg.1	TC1400010618.hg.1	NR_039985	FLJ22447	400221	352.6	1.39E-12	11.858	1.05E-10	P10 - ctrl; ctrl - P11; P3 - ctrl; P8 - ctrl; P10 - P11; P10 - P2; P8 - P10; P3 - P11; P8 - P11; P3 - P2; P8 - P2; P8 - P3
TC1600011514.hg.1	TC1600011514.hg.1	NR_002473	SMG1P2	440354	337.09	1.81E-12	11.741	1.33E-10	ctrl - P10; ctrl - P11; P2 - ctrl; ctrl - P3; ctrl - P8; P11 - P10; P2 - P10; P3 - P10; P10 - P8; P2 - P11; P11 - P8; P2 - P3; P2 - P8; P3 - P8
TC1100007899.hg.1	TC1100007899.hg.1	NM_004585	RARRES3	5920	333.94	1.92E-12	11.717	1.37E-10	P10 - ctrl; P11 - ctrl; ctrl - P2; P3 - ctrl; P8 - ctrl; P10 - P11; P10 - P2; P3 - P10; P8 - P10; P11 - P2; P3 - P11; P8 - P11; P3 - P2; P8 - P2; P3 - P8
TC0400011015.hg.1	TC0400011015.hg.1	NM_002090	CXCL3	2921	331.59	2.00E-12	11.699	1.39E-10	P10 - ctrl; ctrl - P11; ctrl - P2; P3 - ctrl; P8 - ctrl; P10 - P11; P10 - P2; P10 - P3; P8 - P10; P3 - P11; P8 - P11; P3 - P2; P8 - P2; P8 - P3
TC1600007205.hg.1	TC1600007205.hg.1	fusny.aAug10-unspliced	fusny	NA	329.1	2.09E-12	11.679	1.41E-10	ctrl - P10; ctrl - P11; P2 - ctrl; ctrl - P3; ctrl - P8; P11 - P10; P2 - P10; P3 - P10; P10 - P8; P2 - P11; P11 - P8; P2 - P3; P2 - P8; P3 - P8
TC0400007799.hg.1	TC0400007799.hg.1	NM_001098484	SLC4A4	8671	326.17	2.21E-12	11.656	1.45E-10	ctrl - P10; P11 - ctrl; ctrl - P2; ctrl - P3; ctrl - P8; P11 - P10; P2 - P10; P10 - P3; P10 - P8; P11 - P2; P11 - P3; P11 - P8; P2 - P3; P2 - P8; P3 - P8
TC2200008370.hg.1	TC2200008370.hg.1	NM_001079539	XBP1	7494	314.71	2.73E-12	11.564	1.75E-10	P10 - ctrl; P11 - ctrl; P3 - ctrl; P8 - ctrl; P10 - P11; P10 - P2; P10 - P3; P10 - P8; P11 - P2; P11 - P3; P8 - P11; P3 - P2; P8 - P2; P8 - P3
TC1900011742.hg.1	TC1900011742.hg.1	NM_002483	CEACAM6	4680	311.01	2.93E-12	11.533	1.83E-10	P10 - ctrl; P3 - ctrl; P8 - ctrl; P10 - P11; P10 - P2; P3 - P10; P8 - P10; P3 - P11; P8 - P11; P3 - P2; P8 - P2; P3 - P8
TC0600007650.hg.1	TC0600007650.hg.1	NM_019111	HLA-DRA	3122	308.25	3.09E-12	11.51	1.88E-10	P10 - ctrl; P8 - ctrl; P10 - P11; P10 - P2; P10 - P3; P8 - P11; P8 - P2; P8 - P3
TC0100012444.hg.1	TC0100012444.hg.1	NA	NA	NA	306.09	3.22E-12	11.492	1.89E-10	ctrl - P10; ctrl - P11; P2 - ctrl; ctrl - P3; ctrl - P8; P11 - P10; P2 - P10; P3 - P10; P2 - P11; P3 - P11; P11 - P8; P2 - P3; P2 - P8; P3 - P8
TC1500009355.hg.1	TC1500009355.hg.1	NM_203349	SHC4	399694	304.92	3.29E-12	11.482	1.89E-10	P10 - ctrl; ctrl - P11; ctrl - P2; P3 - ctrl; P8 - ctrl; P10 - P11; P10 - P2; P10 - P3; P3 - P11; P8 - P11; P3 - P2; P8 - P2; P8 - P3
TC0600014083.hg.1	TC0600014083.hg.1	NM_021064	HIST1H2AG	8969	304.47	3.32E-12	11.479	1.89E-10	P10 - ctrl; ctrl - P2; P3 - ctrl; P8 - ctrl; P10 - P11; P10 - P2; P10 - P3; P8 - P10; P11 - P2; P3 - P11; P8 - P11; P3 - P2; P8 - P2; P8 - P3
TC1600006658.hg.1	TC1600006658.hg.1	NM_001012631	IL32	9235	301.71	3.51E-12	11.455	1.91E-10	P10 - ctrl; P2 - ctrl; P3 - ctrl; P8 - ctrl; P10 - P11; P10 - P2; P10 - P3; P8 - P10; P2 - P11; P3 - P11; P8 - P11; P3 - P2; P8 - P2; P8 - P3
TC1600011505.hg.1	TC1600011505.hg.1	NM_001310148	NPIPB4	440345	299.11	3.69E-12	11.433	1.91E-10	ctrl - P10; ctrl - P11; P2 - ctrl; ctrl - P3; ctrl - P8; P11 - P10; P2 - P10; P3 - P10; P8 - P10; P2 - P11; P11 - P8; P2 - P3; P2 - P8; P3 - P8
TC0700006890.hg.1	TC0700006890.hg.1	NM_000600	IL6	3569	297.78	3.79E-12	11.421	1.91E-10	P10 - ctrl; ctrl - P11; ctrl - P2; P3 - ctrl; P8 - ctrl; P10 - P11; P10 - P2; P10 - P3; P11 - P2; P3 - P11; P8 - P11; P3 - P2; P8 - P2; P8 - P3
TC2000009216.hg.1	TC2000009216.hg.1	NM_003064	SLPI	6590	297.4	3.82E-12	11.418	1.91E-10	P10 - ctrl; P11 - ctrl; P3 - ctrl; P8 - ctrl; P10 - P11; P10 - P2; P10 - P8; P11 - P2; P3 - P11; P8 - P11; P3 - P2; P8 - P2
TC0700006468.hg.1	TC0700006468.hg.1	NA	NA	NA	296.38	3.90E-12	11.409	1.91E-10	ctrl - P10; ctrl - P11; P2 - ctrl; ctrl - P3; ctrl - P8; P11 - P10; P2 - P10; P3 - P10; P2 - P11; P11 - P8; P2 - P3; P2 - P8; P3 - P8
TC0800011064.hg.1	TC0800011064.hg.1	NM_004929	CALB1	793	295.39	3.98E-12	11.4	1.91E-10	P10 - ctrl; P3 - ctrl; P8 - ctrl; P10 - P11; P10 - P2; P10 - P3; P8 - P10; P3 - P11; P8 - P11; P3 - P2; P8 - P2; P8 - P3
TC2100007792.hg.1	TC2100007792.hg.1	ENST00000419694.1	AP000221.1		294.85	4.02E-12	11.396	1.91E-10	P10 - ctrl; ctrl - P11; P3 - ctrl; P8 - ctrl; P10 - P11; P3 - P10; P8 - P10; P2 - P11; P3 - P11; P8 - P11; P3 - P2; P8 - P2; P3 - P8
TC1100012786.hg.1	TC1100012786.hg.1	NM_000220	KCNJ1	3758	293.93	4.10E-12	11.388	1.91E-10	ctrl - P10; ctrl - P2; ctrl - P3; ctrl - P8; P11 - P10; P2 - P10; P3 - P10; P11 - P2; P11 - P3; P11 - P8; P2 - P3; P2 - P8; P3 - P8
TSUnmapped00000976.hg.1	TSUnmapped00000976.hg.1	NA	NA	NA	293.83	4.10E-12	11.387	1.91E-10	P10 - ctrl; P3 - ctrl; P8 - ctrl; P10 - P11; P10 - P2; P10 - P3; P3 - P11; P8 - P11; P3 - P2; P8 - P2; P8 - P3
TC1400008705.hg.1	TC1400008705.hg.1	NM_001267036	SLC7A8	23428	293.6	4.12E-12	11.385	1.91E-10	ctrl - P10; ctrl - P2; ctrl - P3; ctrl - P8; P11 - P10; P10 - P2; P11 - P2; P11 - P3; P11 - P8
TC1700011813.hg.1	TC1700011813.hg.1	NM_001289107	ST6GALNAC1	55808	292.38	4.23E-12	11.374	1.91E-10	P10 - ctrl; P11 - ctrl; P2 - ctrl; P3 - ctrl; P8 - ctrl; P10 - P11; P10 - P2; P10 - P3; P11 - P2; P11 - P3; P8 - P11; P3 - P2; P8 - P2; P8 - P3
TC0300006677.hg.1	TC0300006677.hg.1	NM_001134367	SLC6A6	6533	290.92	4.35E-12	11.361	1.91E-10	P10 - ctrl; ctrl - P11; ctrl - P2; P3 - ctrl; P8 - ctrl; P10 - P11; P10 - P2; P10 - P3; P8 - P10; P3 - P11; P8 - P11; P3 - P2; P8 - P2; P8 - P3
TC1200010023.hg.1	TC1200010023.hg.1	NM_001300784	ERP27	121506	290.23	4.42E-12	11.355	1.91E-10	P10 - ctrl; P11 - ctrl; ctrl - P2; ctrl - P3; P8 - ctrl; P10 - P11; P10 - P2; P10 - P3; P10 - P8; P11 - P2; P11 - P3; P11 - P8; P3 - P2; P8 - P2; P8 - P3
TC0200016202.hg.1	TC0200016202.hg.1	NM_022449	RAB17	64284	290.03	4.43E-12	11.353	1.91E-10	ctrl - P10; ctrl - P11; ctrl - P2; ctrl - P3; ctrl - P8; P11 - P10; P2 - P10; P10 - P3; P10 - P8; P11 - P2; P11 - P3; P11 - P8; P2 - P3; P2 - P8
TC0100013445.hg.1	TC0100013445.hg.1	NM_002038	IFI6	2537	284.92	4.93E-12	11.307	2.09E-10	P10 - ctrl; ctrl - P2; P3 - ctrl; P8 - ctrl; P10 - P11; P10 - P2; P11 - P2; P3 - P11; P8 - P11; P3 - P2; P8 - P2
TC0800008263.hg.1	TC0800008263.hg.1	NM_001034915	ESRP1	54845	283.37	5.09E-12	11.293	2.12E-10	ctrl - P10; P11 - ctrl; ctrl - P2; ctrl - P3; ctrl - P8; P11 - P10; P2 - P10; P3 - P10; P11 - P2; P11 - P3; P11 - P8; P3 - P2; P2 - P8; P3 - P8
TC1900008286.hg.1	TC1900008286.hg.1	NM_001013257	BCAM	4059	282.59	5.17E-12	11.286	2.12E-10	ctrl - P10; P11 - ctrl; ctrl - P2; ctrl - P3; ctrl - P8; P11 - P10; P10 - P3; P11 - P2; P11 - P3; P11 - P8; P2 - P3; P8 - P2; P8 - P3
TC0100009542.hg.1	TC0100009542.hg.1	flyreebo.aAug10-unspliced	flyreebo	NA	280.09	5.45E-12	11.263	2.20E-10	ctrl - P10; ctrl - P11; ctrl - P2; ctrl - P3; ctrl - P8; P11 - P10; P2 - P10; P3 - P10; P10 - P8; P2 - P11; P11 - P3; P11 - P8; P2 - P3; P2 - P8; P3 - P8
TC1900006814.hg.1	TC1900006814.hg.1	NA	NA	NA	278.18	5.68E-12	11.246	2.25E-10	P10 - ctrl; P11 - ctrl; P3 - ctrl; P8 - ctrl; P10 - P11; P10 - P2; P10 - P3; P10 - P8; P11 - P2; P3 - P11; P8 - P11; P3 - P2; P8 - P2; P8 - P3

TC1000011186.hg.1	TC1000011186.hg.1	NA	NA	NA	277.29	5.79E-12	11.237	2.26E-10	ctrl - P10; ctrl - P11; P2 - ctrl; ctrl - P3; ctrl - P8; P11 - P10; P2 - P10; P3 - P10; P2 - P11; P3 - P11; P11 - P8; P2 - P3; P2 - P8; P3 - P8
TC2200007204.hg.1	TC2200007204.hg.1	NM_002133	HMOX1	3162	267.16	7.22E-12	11.141	2.74E-10	P10 - ctrl; P3 - ctrl; P8 - ctrl; P10 - P11; P10 - P2; P10 - P3; P10 - P8; P3 - P11; P8 - P11; P3 - P2; P8 - P2
TC0200008436.hg.1	TC0200008436.hg.1	NA	NA	NA	266.98	7.25E-12	11.14	2.74E-10	ctrl - P10; ctrl - P11; P2 - ctrl; P3 - ctrl; ctrl - P8; P2 - P10; P3 - P10; P2 - P11; P3 - P11; P8 - P11; P2 - P3; P2 - P8; P3 - P8
TC0400007857.hg.1	TC0400007857.hg.1	NM_001657	AREG	374	259.82	8.52E-12	11.07	3.18E-10	P10 - ctrl; P11 - ctrl; P3 - ctrl; P8 - ctrl; P10 - P11; P10 - P2; P3 - P10; P8 - P10; P11 - P2; P3 - P11; P8 - P11; P3 - P2; P8 - P2; P8 - P3
TC1500007699.hg.1	TC1500007699.hg.1	NM_001281301	KIF23	9493	257.07	9.07E-12	11.042	3.30E-10	P10 - ctrl; ctrl - P11; ctrl - P2; P8 - ctrl; P10 - P11; P10 - P2; P10 - P3; P8 - P10; P2 - P11; P3 - P11; P8 - P11; P3 - P2; P8 - P2; P8 - P3
TC0200009068.hg.1	TC0200009068.hg.1	miniyo.aAug10-unspliced	miniyo	NA	256.29	9.24E-12	11.034	3.30E-10	ctrl - P10; ctrl - P11; ctrl - P2; ctrl - P3; ctrl - P8; P11 - P10; P2 - P10; P10 - P3; P10 - P8; P11 - P3; P11 - P8; P2 - P3; P2 - P8
TC0100018286.hg.1	TC0100018286.hg.1	ENST00000369227	NBPF19	1.01E+08	256.24	9.25E-12	11.034	3.30E-10	ctrl - P10; ctrl - P11; ctrl - P3; ctrl - P8; P11 - P10; P2 - P10; P3 - P10; P2 - P11; P11 - P3; P11 - P8; P2 - P3; P2 - P8; P3 - P8
TC0200016099.hg.1	TC0200016099.hg.1	NM_001282962	HJURP	55355	253.39	9.88E-12	11.005	3.47E-10	P10 - ctrl; ctrl - P11; ctrl - P2; P8 - ctrl; P10 - P11; P10 - P2; P10 - P3; P8 - P10; P2 - P11; P3 - P11; P8 - P11; P3 - P2; P8 - P2; P8 - P3
TC0200015616.hg.1	TC0200015616.hg.1	NM_001042599	ERBB4	2066	252.93	9.99E-12	11	3.47E-10	ctrl - P10; P11 - ctrl; ctrl - P2; ctrl - P3; ctrl - P8; P11 - P10; P2 - P10; P10 - P3; P10 - P8; P11 - P2; P11 - P3; P11 - P8; P2 - P3; P2 - P8; P8 - P3
TC1600009958.hg.1	TC1600009958.hg.1	ENST00000520915	NPIPB4	440345	251.18	1.04E-11	10.982	3.56E-10	ctrl - P10; ctrl - P11; P2 - ctrl; ctrl - P3; ctrl - P8; P11 - P10; P2 - P10; P3 - P10; P2 - P11; P11 - P8; P2 - P3; P2 - P8; P3 - P8
TC1500010618.hg.1	TC1500010618.hg.1	dazee.aAug10	dazee		250.64	1.05E-11	10.977	3.56E-10	ctrl - P10; ctrl - P11; ctrl - P3; ctrl - P8; P11 - P10; P2 - P10; P3 - P10; P10 - P8; P2 - P11; P11 - P3; P11 - P8; P2 - P3; P2 - P8; P3 - P8
TC0400007840.hg.1	TC0400007840.hg.1	NM_001511	CXCL1	2919	249.09	1.09E-11	10.961	3.65E-10	P10 - ctrl; ctrl - P11; ctrl - P2; P3 - ctrl; P8 - ctrl; P10 - P11; P10 - P2; P10 - P3; P8 - P10; P2 - P11; P3 - P11; P8 - P11; P3 - P2; P8 - P2; P8 - P3
TC0500011705.hg.1	TC0500011705.hg.1	NM_001142474	NREP	9315	248.07	1.12E-11	10.95	3.67E-10	ctrl - P10; ctrl - P2; ctrl - P3; ctrl - P8; P11 - P10; P2 - P10; P3 - P10; P8 - P10; P11 - P2; P11 - P3; P11 - P8; P2 - P8; P3 - P8
TC0300013520.hg.1	TC0300013520.hg.1	NM_021101	CLDN1	9076	247.72	1.13E-11	10.947	3.67E-10	P10 - ctrl; ctrl - P11; ctrl - P2; P3 - ctrl; P8 - ctrl; P10 - P11; P10 - P2; P10 - P3; P8 - P10; P2 - P11; P3 - P11; P8 - P11; P3 - P2; P8 - P2; P8 - P3
TC1600010375.hg.1	TC1600010375.hg.1	NM_001308963	CRNDE	643911	245.84	1.18E-11	10.927	3.79E-10	ctrl - P10; ctrl - P11; ctrl - P3; ctrl - P8; P11 - P10; P2 - P10; P10 - P3; P2 - P11; P11 - P3; P11 - P8; P2 - P3; P2 - P8; P8 - P3
TC1500007067.hg.1	TC1500007067.hg.1	NM_001015001	CKMT1A	548596	245.28	1.20E-11	10.921	3.79E-10	P10 - ctrl; ctrl - P2; P3 - ctrl; P8 - ctrl; P10 - P11; P10 - P2; P10 - P3; P11 - P2; P3 - P11; P8 - P11; P3 - P2; P8 - P2; P8 - P3
TC0100015155.hg.1	TC0100015155.hg.1	NM_001079874	VAV3	10451	243.73	1.24E-11	10.905	3.89E-10	ctrl - P10; ctrl - P2; ctrl - P3; ctrl - P8; P11 - P10; P2 - P10; P10 - P3; P10 - P8; P11 - P2; P11 - P3; P11 - P8; P2 - P3; P2 - P8; P3 - P8
TC0400012829.hg.1	TC0400012829.hg.1	NM_001242729	ARHGEF38	54848	243.02	1.27E-11	10.897	3.91E-10	ctrl - P10; ctrl - P11; ctrl - P2; ctrl - P3; ctrl - P8; P11 - P10; P2 - P10; P10 - P3; P10 - P8; P2 - P11; P11 - P3; P11 - P8; P2 - P3; P2 - P8; P3 - P8
TC1700008719.hg.1	TC1700008719.hg.1	NM_002266	KPNA2	3838	241.96	1.30E-11	10.886	3.93E-10	P10 - ctrl; ctrl - P11; ctrl - P2; P3 - ctrl; P8 - ctrl; P10 - P11; P10 - P2; P10 - P3; P8 - P10; P11 - P2; P3 - P11; P8 - P11; P3 - P2; P8 - P2; P8 - P3
TC0600014277.hg.1	TC0600014277.hg.1	NM_006120	HLA-DMA	3108	241.62	1.31E-11	10.883	3.93E-10	P10 - ctrl; ctrl - P2; P3 - ctrl; P8 - ctrl; P10 - P11; P10 - P2; P10 - P3; P11 - P2; P3 - P11; P8 - P11; P3 - P2; P8 - P2; P8 - P3
TC1600006946.hg.1	TC1600006946.hg.1	wupa.aAug10-unspliced	wupa	NA	241.27	1.32E-11	10.879	3.93E-10	ctrl - P10; ctrl - P11; P2 - ctrl; ctrl - P3; ctrl - P8; P11 - P10; P2 - P10; P3 - P10; P10 - P8; P2 - P11; P11 - P3; P11 - P8; P2 - P3; P2 - P8; P3 - P8
TC0500010553.hg.1	TC0500010553.hg.1	NM_001243093	FYB	2533	239.69	1.37E-11	10.862	4.04E-10	ctrl - P10; P11 - ctrl; ctrl - P2; ctrl - P3; ctrl - P8; P11 - P10; P2 - P10; P3 - P10; P11 - P2; P11 - P3; P11 - P8; P2 - P3; P2 - P8
TC0700008182.hg.1	TC0700008182.hg.1	kleysmeebu.aAug10-unspliced	kleysmeebu	NA	238.88	1.40E-11	10.853	4.07E-10	ctrl - P10; ctrl - P11; P2 - ctrl; ctrl - P3; ctrl - P8; P11 - P10; P2 - P10; P3 - P10; P10 - P8; P2 - P11; P11 - P8; P2 - P3; P2 - P8; P3 - P8
TC1100008018.hg.1	TC1100008018.hg.1	NR_028272	NEAT1	283131	237.88	1.44E-11	10.842	4.13E-10	ctrl - P10; ctrl - P11; ctrl - P3; ctrl - P8; P11 - P10; P2 - P10; P3 - P10; P10 - P8; P2 - P11; P3 - P11; P11 - P8; P2 - P3; P2 - P8; P3 - P8
TC0400012621.hg.1	TC0400012621.hg.1	NM_001286708	ACSL1	2180	236.23	1.50E-11	10.824	4.25E-10	ctrl - P10; P11 - ctrl; ctrl - P2; ctrl - P3; ctrl - P8; P11 - P10; P10 - P3; P10 - P8; P11 - P2; P11 - P3; P11 - P8; P2 - P3; P2 - P8; P3 - P8
TC0300008123.hg.1	TC0300008123.hg.1	ENST00000473756.1	LINC00973		235.24	1.54E-11	10.814	4.31E-10	P10 - ctrl; ctrl - P11; ctrl - P2; P3 - ctrl; P8 - ctrl; P10 - P11; P10 - P2; P3 - P10; P8 - P10; P3 - P11; P8 - P11; P3 - P2; P8 - P2; P3 - P8
TC0100015868.hg.1	TC0100015868.hg.1	NM_005978	S100A2	6273	234.39	1.57E-11	10.804	4.35E-10	ctrl - P10; ctrl - P2; P3 - ctrl; ctrl - P8; P11 - P10; P2 - P10; P3 - P10; P8 - P10; P11 - P2; P3 - P11; P11 - P8; P3 - P2; P2 - P8; P3 - P8
TC0100010798.hg.1	TC0100010798.hg.1	NM_001004128	QSOX1	5768	234.02	1.58E-11	10.8	4.35E-10	P10 - ctrl; ctrl - P2; P3 - ctrl; P8 - ctrl; P10 - P11; P10 - P2; P10 - P3; P11 - P2; P3 - P11; P8 - P11; P3 - P2; P8 - P2; P8 - P3
TC0200015624.hg.1	TC0200015624.hg.1	zoyklu.aAug10-unspliced	zoyklu	NA	233.38	1.61E-11	10.793	4.37E-10	ctrl - P10; ctrl - P11; ctrl - P3; ctrl - P8; P11 - P10; P2 - P10; P10 - P3; P10 - P8; P2 - P11; P11 - P3; P11 - P8; P2 - P3; P2 - P8; P3 - P8
TC1600008890.hg.1	TC1600008890.hg.1	NA	NA	NA	230.13	1.75E-11	10.757	4.68E-10	ctrl - P10; ctrl - P11; P2 - ctrl; ctrl - P8; P10 - P11; P2 - P10; P3 - P10; P2 - P11; P3 - P11; P8 - P11; P2 - P3; P2 - P8; P3 - P8
TC0200007261.hg.1	TC0200007261.hg.1	NM_012413	QPCT	25797	229.86	1.76E-11	10.754	4.68E-10	ctrl - P10; ctrl - P2; ctrl - P8; P11 - P10; P2 - P10; P3 - P10; P11 - P2; P11 - P8; P3 - P2; P2 - P8; P3 - P8
TC2100007208.hg.1	TC2100007208.hg.1	NM_001144925	MX1	4599	227.94	1.85E-11	10.733	4.87E-10	P10 - ctrl; ctrl - P11; ctrl - P2; P3 - ctrl; P8 - ctrl; P10 - P11; P10 - P2; P10 - P3; P11 - P2; P3 - P11; P8 - P11; P3 - P2; P8 - P2; P8 - P3

TC0400011014.hg.1	TC0400011014.hg.1	NM_002994	CXCL5	6374	226.39	1.93E-11	10.715	5.02E-10	ctrl - P10; ctrl - P2; P3 - ctrl; ctrl - P8; P11 - P10; P3 - P10; P8 - P10; P11 - P2; P3 - P11; P11 - P8; P3 - P2; P8 - P2; P3 - P8
TC1000012235.hg.1	TC1000012235.hg.1	NM_001145966	MKI67	4288	224.63	2.02E-11	10.695	5.20E-10	P10 - ctrl; ctrl - P11; ctrl - P2; P8 - ctrl; P10 - P11; P10 - P2; P10 - P3; P8 - P10; P3 - P11; P8 - P11; P3 - P2; P8 - P2; P8 - P3
TC1900009559.hg.1	TC1900009559.hg.1	NM_024690	MUC16	94025	224.18	2.04E-11	10.69	5.21E-10	P10 - ctrl; P3 - ctrl; P8 - ctrl; P10 - P11; P10 - P2; P10 - P3; P8 - P10; P3 - P11; P8 - P11; P3 - P2; P8 - P2; P8 - P3
TC2000009604.hg.1	TC2000009604.hg.1	NM_001255976	PMEPA1	56937	223.68	2.07E-11	10.684	5.22E-10	P10 - ctrl; ctrl - P11; P3 - ctrl; P8 - ctrl; P10 - P11; P10 - P2; P10 - P3; P8 - P10; P2 - P11; P3 - P11; P8 - P11; P3 - P2; P8 - P2; P8 - P3
TC0500007974.hg.1	TC0500007974.hg.1	spoyployby.aAug10-unspliced	spoyployby	NA	222.28	2.15E-11	10.668	5.37E-10	ctrl - P10; ctrl - P11; P2 - ctrl; P3 - ctrl; ctrl - P8; P10 - P11; P2 - P10; P3 - P10; P10 - P8; P2 - P11; P3 - P11; P2 - P3; P2 - P8; P3 - P8
TC0X00006799.hg.1	TC0X00006799.hg.1	NM_002970	SAT1	Xp22.1	221.93	2.17E-11	10.664	5.37E-10	P10 - ctrl; ctrl - P11; ctrl - P2; P3 - ctrl; P8 - ctrl; P10 - P11; P10 - P2; P3 - P11; P8 - P11; P3 - P2; P8 - P2
TC0700013427.hg.1	TC0700013427.hg.1	NR_036569	STAG3L5P-PVRIG2P-PILRB	1.02E+08	220.33	2.26E-11	10.645	5.55E-10	ctrl - P10; ctrl - P11; ctrl - P2; ctrl - P3; ctrl - P8; P11 - P10; P2 - P10; P3 - P10; P2 - P11; P11 - P8; P2 - P3; P2 - P8; P3 - P8
TC0200010980.hg.1	TC0200010980.hg.1	NM_001130046	CCL20	6364	218.98	2.35E-11	10.629	5.70E-10	P10 - ctrl; P11 - ctrl; P2 - ctrl; P3 - ctrl; P8 - ctrl; P10 - P11; P10 - P2; P10 - P3; P8 - P10; P3 - P11; P8 - P11; P3 - P2; P8 - P2; P8 - P3
TC0100009364.hg.1	TC0100009364.hg.1	NM_000757	CSF1	1435	217.38	2.45E-11	10.611	5.87E-10	P10 - ctrl; P11 - ctrl; ctrl - P2; P3 - ctrl; P8 - ctrl; P10 - P11; P10 - P2; P3 - P10; P8 - P10; P11 - P2; P3 - P11; P8 - P11; P3 - P2; P8 - P2; P8 - P3
TC0300009853.hg.1	TC0300009853.hg.1	NM_006580	CLDN16	10686	217.17	2.47E-11	10.608	5.87E-10	ctrl - P10; ctrl - P11; ctrl - P2; ctrl - P3; ctrl - P8; P11 - P10; P2 - P10; P3 - P10; P11 - P2; P11 - P3; P11 - P8; P3 - P2; P2 - P8; P3 - P8
TC0800007715.hg.1	TC0800007715.hg.1	NR_038235_2	LINC01606		215.26	2.60E-11	10.585	6.09E-10	ctrl - P10; ctrl - P11; ctrl - P2; ctrl - P3; ctrl - P8; P11 - P10; P2 - P10; P3 - P10; P11 - P2; P11 - P3; P11 - P8; P2 - P3; P2 - P8; P3 - P8
TC0100014193.hg.1	TC0100014193.hg.1	NM_015913	TXNDC12	51060	215.16	2.61E-11	10.584	6.09E-10	P10 - ctrl; P11 - ctrl; ctrl - P2; ctrl - P3; P8 - ctrl; P10 - P11; P10 - P2; P10 - P3; P10 - P8; P11 - P2; P11 - P3; P8 - P11; P3 - P2; P8 - P2; P8 - P3
TC1500010407.hg.1	TC1500010407.hg.1	NM_001150	ANPEP	290	213.71	2.71E-11	10.567	6.27E-10	ctrl - P10; ctrl - P11; ctrl - P2; P3 - ctrl; ctrl - P8; P11 - P10; P2 - P10; P3 - P10; P8 - P10; P3 - P11; P8 - P11; P3 - P2; P3 - P8
TC0600011938.hg.1	TC0600011938.hg.1	NM_001114086	CLIC5	53405	212.2	2.83E-11	10.549	6.48E-10	P10 - ctrl; ctrl - P2; P3 - ctrl; P8 - ctrl; P10 - P11; P10 - P2; P8 - P10; P3 - P11; P8 - P11; P3 - P2; P8 - P2; P8 - P3
TC0300009139.hg.1	TC0300009139.hg.1	NM_004617	TM4SF4	7104	211.75	2.86E-11	10.543	6.51E-10	P10 - ctrl; ctrl - P11; P2 - ctrl; P3 - ctrl; P8 - ctrl; P10 - P11; P10 - P2; P10 - P8; P2 - P11; P3 - P11; P8 - P11; P3 - P2; P3 - P8
TC1600010168.hg.1	TC1600010168.hg.1	NM_024745	SHCBP1	79801	209.05	3.09E-11	10.51	6.95E-10	P10 - ctrl; ctrl - P11; ctrl - P2; P8 - ctrl; P10 - P11; P10 - P2; P10 - P3; P8 - P10; P3 - P11; P8 - P11; P3 - P2; P8 - P2; P8 - P3
TC0900011072.hg.1	TC0900011072.hg.1	NM_005502	ABCA1	19	208.55	3.13E-11	10.504	6.99E-10	P10 - ctrl; P11 - ctrl; P2 - ctrl; P3 - ctrl; P8 - ctrl; P10 - P11; P10 - P2; P10 - P3; P3 - P11; P8 - P11; P3 - P2; P8 - P2; P8 - P3
TC0300012236.hg.1	TC0300012236.hg.1	NM_033049	MUC13	56667	208.27	3.16E-11	10.501	6.99E-10	P10 - ctrl; P11 - ctrl; P2 - ctrl; P3 - ctrl; P8 - ctrl; P10 - P11; P10 - P2; P10 - P3; P10 - P8; P11 - P2; P3 - P11; P8 - P11; P3 - P2; P8 - P2; P8 - P3
TC1400008677.hg.1	TC1400008677.hg.1	NM_001126105	SLC7A7	9056	207.5	3.23E-11	10.491	7.08E-10	P10 - ctrl; P11 - ctrl; P2 - ctrl; P3 - ctrl; P8 - ctrl; P10 - P11; P10 - P2; P10 - P3; P3 - P11; P8 - P11; P3 - P2; P8 - P2; P8 - P3
TC1700010618.hg.1	TC1700010618.hg.1	NM_001067	TOP2A	7153	205.8	3.39E-11	10.47	7.37E-10	P10 - ctrl; ctrl - P11; ctrl - P2; P3 - ctrl; P8 - ctrl; P10 - P11; P10 - P2; P10 - P3; P8 - P10; P3 - P11; P8 - P11; P3 - P2; P8 - P2; P8 - P3
TC0400008137.hg.1	TC0400008137.hg.1	NM_001145065	CCSER1	401145	204.81	3.49E-11	10.458	7.51E-10	ctrl - P10; P11 - ctrl; ctrl - P2; ctrl - P3; ctrl - P8; P11 - P10; P2 - P10; P3 - P10; P11 - P2; P11 - P3; P11 - P8; P2 - P3; P2 - P8; P3 - P8
TC2000008316.hg.1	TC2000008316.hg.1	NM_152611	LRRN4	164312	202.71	3.71E-11	10.431	7.87E-10	ctrl - P11; ctrl - P2; P3 - ctrl; P8 - ctrl; P10 - P11; P10 - P2; P3 - P10; P8 - P10; P11 - P2; P3 - P11; P8 - P11; P3 - P2; P8 - P2
TC0100015641.hg.1	TC0100015641.hg.1	NR_111936	PDZK1P1	1E+08	202.62	3.72E-11	10.43	7.87E-10	ctrl - P10; ctrl - P11; ctrl - P2; ctrl - P3; ctrl - P8; P11 - P10; P2 - P10; P10 - P3; P10 - P8; P11 - P3; P11 - P8; P2 - P3; P2 - P8
TC0400010242.hg.1	TC0400010242.hg.1	NM_013261	PPARGC1A	10891	200.36	3.97E-11	10.401	8.34E-10	ctrl - P10; ctrl - P2; ctrl - P3; ctrl - P8; P11 - P10; P2 - P10; P10 - P3; P10 - P8; P11 - P2; P11 - P3; P11 - P8; P2 - P3; P2 - P8; P8 - P3
TC1200010145.hg.1	TC1200010145.hg.1	NM_001178091	BCAT1	586	198.14	4.24E-11	10.372	8.81E-10	P10 - ctrl; P3 - ctrl; P8 - ctrl; P10 - P11; P10 - P2; P10 - P3; P8 - P10; P3 - P11; P8 - P11; P3 - P2; P8 - P2; P8 - P3
TC1500008370.hg.1	TC1500008370.hg.1	NM_006011	ST8SIA2	8128	197.94	4.27E-11	10.37	8.81E-10	P10 - ctrl; P11 - ctrl; P3 - ctrl; P8 - ctrl; P10 - P11; P10 - P2; P10 - P3; P8 - P10; P11 - P2; P3 - P11; P8 - P11; P3 - P2; P8 - P2; P8 - P3
TC0200016769.hg.1	TC0200016769.hg.1	NA	NA	NA	197.14	4.37E-11	10.359	8.92E-10	ctrl - P10; ctrl - P11; ctrl - P2; ctrl - P3; ctrl - P8; P11 - P10; P2 - P10; P3 - P10; P11 - P2; P11 - P3; P11 - P8; P2 - P3; P2 - P8; P3 - P8
TC1500007409.hg.1	TC1500007409.hg.1	NM_004751	GCNT3	9245	196.99	4.39E-11	10.358	8.92E-10	P10 - ctrl; P11 - ctrl; P2 - ctrl; P3 - ctrl; P8 - ctrl; P10 - P11; P10 - P2; P11 - P2; P3 - P11; P8 - P11; P3 - P2; P8 - P2; P8 - P3
TC1500006999.hg.1	TC1500006999.hg.1	NM_001243142	NUSAP1	51203	196.36	4.47E-11	10.349	9.02E-10	P10 - ctrl; ctrl - P11; ctrl - P2; ctrl - P3; P8 - ctrl; P10 - P11; P10 - P2; P10 - P3; P8 - P10; P8 - P11; P3 - P2; P8 - P2; P8 - P3
TC1900007808.hg.1	TC1900007808.hg.1	NA	NA	NA	195.55	4.59E-11	10.339	9.17E-10	ctrl - P10; ctrl - P11; ctrl - P2; ctrl - P3; ctrl - P8; P11 - P10; P2 - P10; P3 - P10; P10 - P8; P2 - P11; P11 - P8; P2 - P3; P2 - P8; P3 - P8
TC1200012748.hg.1	TC1200012748.hg.1	NM_001297650	CD163L1	283316	193.98	4.81E-11	10.318	9.54E-10	P10 - ctrl; P11 - ctrl; P2 - ctrl; P3 - ctrl; P8 - ctrl; P10 - P11; P10 - P2; P8 - P10; P11 - P2; P3 - P11; P8 - P11; P3 - P2; P8 - P2; P8 - P3
TC1000010567.hg.1	TC1000010567.hg.1	charsho.aAug10-unspliced	charsho	NA	193.38	4.90E-11	10.31	9.64E-10	ctrl - P10; ctrl - P11; P2 - ctrl; ctrl - P3; ctrl - P8; P11 - P10; P2 - P10; P3 - P10; P2 - P11; P3 - P11; P11 - P8; P2 - P3; P2 - P8; P3 - P8
TC1100011375.hg.1	TC1100011375.hg.1	NM_001300913	C11orf24	53838	192.61	5.01E-11	10.3	9.79E-10	P10 - ctrl; ctrl - P2; P3 - ctrl; P8 - ctrl; P10 - P11; P10 - P2; P10 - P3; P8 - P10; P11 - P2; P3 - P11; P8 - P11; P3 - P2; P8 - P2; P8 - P3

TC0800007491.hg.1	TC0800007491.hg.1	NA	NA	NA	191.81	5.14E-11	10.289	9.95E-10	ctrl - P10; ctrl - P11; P2 - ctrl; ctrl - P8; P2 - P10; P3 - P10; P8 - P10; P2 - P11; P3 - P11; P8 - P11; P2 - P3; P2 - P8; P3 - P8
TC1200012768.hg.1	TC1200012768.hg.1	NM_001190726	RERG	85004	191.57	5.18E-11	10.286	9.95E-10	ctrl - P10; ctrl - P2; ctrl - P3; ctrl - P8; P11 - P10; P2 - P10; P3 - P10; P11 - P2; P11 - P3; P11 - P8; P2 - P3; P2 - P8; P3 - P8
TC0700010965.hg.1	TC0700010965.hg.1	NM_000598	IGFBP3	3486	191.34	5.21E-11	10.283	9.95E-10	P10 - ctrl; P11 - ctrl; ctrl - P2; P3 - ctrl; P8 - ctrl; P10 - P11; P10 - P2; P10 - P3; P8 - P10; P11 - P2; P11 - P3; P8 - P11; P3 - P2; P8 - P2; P8 - P3
TC1600010006.hg.1	TC1600010006.hg.1	NM_002773	PRSS8	5652	190.77	5.31E-11	10.275	1.00E-09	P10 - ctrl; P11 - ctrl; ctrl - P2; ctrl - P3; P8 - ctrl; P10 - P11; P10 - P2; P10 - P3; P10 - P8; P11 - P2; P11 - P3; P8 - P11; P2 - P3; P8 - P2; P8 - P3
TC0800009970.hg.1	TC0800009970.hg.1	NM_001278945	PBK	55872	190.62	5.33E-11	10.273	1.00E-09	P10 - ctrl; ctrl - P11; ctrl - P2; P3 - ctrl; P8 - ctrl; P10 - P11; P10 - P2; P10 - P3; P8 - P10; P3 - P11; P8 - P11; P3 - P2; P8 - P2; P8 - P3
TC0Y00006486.hg.1	TC0Y00006486.hg.1	rorbor.aAug10-unspliced	rorbor	NA	190	5.44E-11	10.265	1.01E-09	ctrl - P10; ctrl - P11; P2 - ctrl; ctrl - P3; ctrl - P8; P11 - P10; P2 - P10; P3 - P10; P10 - P8; P2 - P11; P11 - P8; P2 - P3; P2 - P8; P3 - P8
TC1500009504.hg.1	TC1500009504.hg.1	NM_015617	PYGO1	26108	189.9	5.45E-11	10.263	1.01E-09	P10 - ctrl; ctrl - P11; P3 - ctrl; P8 - ctrl; P10 - P11; P10 - P2; P10 - P3; P2 - P11; P3 - P11; P8 - P11; P3 - P2; P8 - P2; P8 - P3
TC0400007836.hg.1	TC0400007836.hg.1	NM_000584	CXCL8	3576	189.54	5.51E-11	10.259	1.01E-09	P10 - ctrl; ctrl - P11; P3 - ctrl; P8 - ctrl; P10 - P11; P10 - P2; P8 - P10; P3 - P11; P8 - P11; P3 - P2; P8 - P2; P8 - P3
TC1200009829.hg.1	TC1200009829.hg.1	NM_014358	CLEC4E	26253	186.77	6.02E-11	10.221	1.09E-09	P10 - ctrl; P2 - ctrl; P3 - ctrl; P8 - ctrl; P10 - P11; P10 - P2; P10 - P3; P2 - P11; P3 - P11; P8 - P11; P3 - P2; P8 - P2; P8 - P3
TC0700010604.hg.1	TC0700010604.hg.1	NM_019029	CPVL	54504	186.45	6.08E-11	10.216	1.09E-09	ctrl - P10; ctrl - P2; ctrl - P3; ctrl - P8; P11 - P10; P2 - P10; P3 - P10; P10 - P8; P11 - P2; P11 - P3; P11 - P8; P2 - P8; P3 - P8
TC0300013076.hg.1	TC0300013076.hg.1	skorsweyby.aAug10-unspliced	skorsweyby	NA	186.41	6.08E-11	10.216	1.09E-09	ctrl - P10; ctrl - P11; P2 - ctrl; ctrl - P3; ctrl - P8; P11 - P10; P2 - P10; P10 - P8; P2 - P11; P11 - P3; P11 - P8; P2 - P3; P2 - P8; P3 - P8
TC0400006889.hg.1	TC0400006889.hg.1	NA	NA	NA	185.59	6.25E-11	10.204	1.11E-09	P10 - ctrl; P3 - ctrl; P8 - ctrl; P10 - P11; P10 - P2; P3 - P10; P10 - P8; P3 - P11; P8 - P11; P3 - P2; P8 - P2; P3 - P8
TC1600009929.hg.1	TC1600009929.hg.1	uc021tgb.2	RP11-231C14.4		185.13	6.34E-11	10.198	1.12E-09	ctrl - P10; ctrl - P11; P2 - ctrl; ctrl - P3; ctrl - P8; P11 - P10; P2 - P10; P3 - P10; P8 - P10; P2 - P11; P11 - P8; P2 - P3; P2 - P8; P3 - P8
TC0500011596.hg.1	TC0500011596.hg.1	NM_180991	SLCO4C1	353189	185.02	6.36E-11	10.197	1.12E-09	ctrl - P10; ctrl - P11; ctrl - P2; ctrl - P3; ctrl - P8; P11 - P10; P10 - P3; P10 - P8; P11 - P2; P11 - P3; P11 - P8; P2 - P3; P2 - P8; P3 - P8
TC0700013394.hg.1	TC0700013394.hg.1	NM_020879	CCDC146	57639	184.39	6.49E-11	10.188	1.13E-09	P10 - ctrl; ctrl - P11; P2 - ctrl; P3 - ctrl; P8 - ctrl; P10 - P11; P10 - P2; P10 - P3; P8 - P10; P2 - P11; P3 - P11; P8 - P11; P3 - P2; P8 - P2; P8 - P3
TC1300009580.hg.1	TC1300009580.hg.1	NM_005073	SLC15A1	6564	183.79	6.62E-11	10.179	1.15E-09	P10 - ctrl; P2 - ctrl; P3 - ctrl; P8 - ctrl; P10 - P11; P10 - P2; P10 - P3; P10 - P8; P2 - P11; P3 - P11; P8 - P11; P2 - P3; P8 - P2; P8 - P3
TC1300008013.hg.1	TC1300008013.hg.1	NA	NA	NA	182.93	6.80E-11	10.167	1.17E-09	ctrl - P10; ctrl - P11; P2 - ctrl; ctrl - P3; ctrl - P8; P10 - P11; P2 - P10; P10 - P8; P2 - P11; P3 - P11; P11 - P8; P2 - P3; P2 - P8; P3 - P8
TC1000007471.hg.1	TC1000007471.hg.1	NM_000698	ALOX5	240	182.76	6.84E-11	10.165	1.17E-09	P10 - ctrl; P11 - ctrl; P3 - ctrl; P8 - ctrl; P10 - P11; P10 - P2; P10 - P3; P11 - P2; P3 - P11; P8 - P11; P3 - P2; P8 - P2; P8 - P3
TC0500008777.hg.1	TC0500008777.hg.1	NM_005733	KIF20A	10112	182.43	6.91E-11	10.16	1.18E-09	P10 - ctrl; ctrl - P11; ctrl - P2; ctrl - P3; P8 - ctrl; P10 - P11; P10 - P2; P10 - P3; P8 - P10; P11 - P2; P3 - P11; P8 - P11; P3 - P2; P8 - P2; P8 - P3
TC1800006484.hg.1	TC1800006484.hg.1	NM_006101	NDC80	10403	181.84	7.04E-11	10.152	1.19E-09	P10 - ctrl; ctrl - P11; ctrl - P2; P8 - ctrl; P10 - P11; P10 - P2; P10 - P3; P8 - P10; P11 - P2; P3 - P11; P8 - P11; P3 - P2; P8 - P2; P8 - P3
TC0100018480.hg.1	TC0100018480.hg.1	NM_015383	NBPF14	25832	181.29	7.17E-11	10.144	1.20E-09	ctrl - P10; ctrl - P11; ctrl - P3; ctrl - P8; P11 - P10; P2 - P10; P3 - P10; P2 - P11; P11 - P3; P11 - P8; P2 - P3; P2 - P8; P3 - P8
TC1600011512.hg.1	TC1600011512.hg.1	NA	NA	NA	181.11	7.21E-11	10.142	1.20E-09	ctrl - P10; ctrl - P11; P2 - ctrl; ctrl - P3; ctrl - P8; P11 - P10; P2 - P10; P3 - P10; P2 - P11; P11 - P8; P2 - P3; P2 - P8; P3 - P8
TC1500007143.hg.1	TC1500007143.hg.1	NM_001198999	SEMA6D	80031	178.53	7.85E-11	10.105	1.30E-09	ctrl - P10; P11 - ctrl; ctrl - P2; ctrl - P3; ctrl - P8; P11 - P10; P2 - P10; P3 - P10; P11 - P2; P11 - P3; P11 - P8; P2 - P3; P2 - P8; P3 - P8
TC1000011400.hg.1	TC1000011400.hg.1	NM_014391	ANKRD1	27063	178.41	7.88E-11	10.103	1.30E-09	P10 - ctrl; P3 - ctrl; P8 - ctrl; P10 - P11; P10 - P2; P8 - P10; P3 - P11; P8 - P11; P3 - P2; P8 - P2; P8 - P3
TC1900011233.hg.1	TC1900011233.hg.1	NM_001077500	KLK10	5655	177.5	8.12E-11	10.09	1.32E-09	P10 - ctrl; ctrl - P2; P3 - ctrl; P8 - ctrl; P10 - P11; P10 - P2; P8 - P10; P11 - P2; P3 - P11; P8 - P11; P3 - P2; P8 - P2; P8 - P3
TC1900006671.hg.1	TC1900006671.hg.1	NA	NA	NA	177.42	8.15E-11	10.089	1.32E-09	ctrl - P10; ctrl - P11; P2 - ctrl; ctrl - P3; ctrl - P8; P11 - P10; P2 - P10; P3 - P10; P2 - P11; P11 - P8; P2 - P3; P2 - P8; P3 - P8
TC0500012294.hg.1	TC0500012294.hg.1	NM_001278613	PCDH1	5097	176.66	8.35E-11	10.078	1.35E-09	P10 - ctrl; P11 - ctrl; P3 - ctrl; P8 - ctrl; P10 - P11; P10 - P2; P11 - P2; P3 - P11; P8 - P11; P3 - P2; P8 - P2
TC0800007382.hg.1	TC0800007382.hg.1	NM_002164	IDO1	3620	175.82	8.59E-11	10.066	1.37E-09	P10 - ctrl; P8 - ctrl; P10 - P11; P10 - P2; P10 - P3; P8 - P10; P3 - P11; P8 - P11; P8 - P2; P8 - P3
TC1900011741.hg.1	TC1900011741.hg.1	NM_001291484	CEACAM5	1048	175.66	8.64E-11	10.063	1.37E-09	P10 - ctrl; ctrl - P2; P3 - ctrl; P8 - ctrl; P10 - P11; P10 - P2; P3 - P10; P8 - P10; P11 - P2; P3 - P11; P8 - P11; P3 - P2; P8 - P2; P3 - P8
TC0X00010155.hg.1	TC0X00010155.hg.1	NM_001171581	ITM2A	9452	175.64	8.65E-11	10.063	1.37E-09	ctrl - P10; ctrl - P2; ctrl - P3; ctrl - P8; P11 - P10; P2 - P10; P3 - P10; P8 - P10; P11 - P2; P11 - P3; P11 - P8; P3 - P2; P2 - P8; P3 - P8
TC1300008095.hg.1	TC1300008095.hg.1	NA	NA	NA	175.14	8.79E-11	10.056	1.38E-09	ctrl - P10; ctrl - P11; ctrl - P3; ctrl - P8; P11 - P10; P2 - P10; P10 - P8; P2 - P11; P11 - P3; P11 - P8; P2 - P3; P2 - P8; P3 - P8
TC1200007012.hg.1	TC1200007012.hg.1	cheenar.aAug10-unspliced	cheenar	NA	174.7	8.92E-11	10.049	1.39E-09	ctrl - P10; ctrl - P11; P2 - ctrl; ctrl - P8; P11 - P10; P2 - P10; P3 - P10; P10 - P8; P2 - P11; P3 - P11; P11 - P8; P2 - P3; P2 - P8; P3 - P8
TC0400011013.hg.1	TC0400011013.hg.1	NM_002704	PPBP	5473	174.32	9.04E-11	10.044	1.40E-09	P10 - ctrl; P3 - ctrl; P8 - ctrl; P10 - P11; P10 - P2; P10 - P3; P8 - P10; P3 - P11; P8 - P11; P3 - P2; P8 - P2; P8 - P3

TC0500010639.hg.1	TC0500010639.hg.1	NM_001301873	CCL28	56477	171.46	9.97E-11	10.002	1.54E-09	P10 - ctrl; ctrl - P2; P3 - ctrl; P8 - ctrl; P10 - P11; P10 - P2; P10 - P3; P8 - P10; P11 - P2; P3 - P11; P8 - P11; P3 - P2; P8 - P2; P8 - P3
TC1900010807.hg.1	TC1900010807.hg.1	NM_001024912	CEACAM1	634	168.74	1.10E-10	9.9605	1.66E-09	P10 - ctrl; ctrl - P2; P3 - ctrl; P8 - ctrl; P10 - P11; P10 - P2; P3 - P10; P11 - P2; P3 - P11; P8 - P11; P3 - P2; P8 - P2; P3 - P8
TC1700012274.hg.1	TC1700012274.hg.1	NM_000212	ITGB3	3690	168.7	1.10E-10	9.9599	1.66E-09	P10 - ctrl; ctrl - P11; P3 - ctrl; P8 - ctrl; P10 - P11; P10 - P2; P8 - P10; P2 - P11; P3 - P11; P8 - P11; P3 - P2; P8 - P2
TC0500007044.hg.1	TC0500007044.hg.1	NM_004932	CDH6	1004	168.7	1.10E-10	9.9599	1.66E-09	P10 - ctrl; ctrl - P11; P3 - ctrl; P8 - ctrl; P10 - P11; P10 - P2; P3 - P10; P8 - P10; P2 - P11; P3 - P11; P8 - P11; P3 - P2; P8 - P2
TC1800008210.hg.1	TC1800008210.hg.1	OTTHUMT00000443414	BNIP3P3		167.81	1.13E-10	9.9463	1.70E-09	ctrl - P10; ctrl - P11; P2 - ctrl; P3 - ctrl; ctrl - P8; P10 - P11; P2 - P10; P3 - P10; P10 - P8; P2 - P11; P3 - P11; P8 - P11; P2 - P3; P2 - P8; P3 - P8
TC0600014106.hg.1	TC0600014106.hg.1	NM_001710	CFB	629	167.76	1.13E-10	9.9456	1.70E-09	P10 - ctrl; P3 - ctrl; P8 - ctrl; P10 - P11; P10 - P2; P10 - P3; P3 - P11; P8 - P11; P3 - P2; P8 - P2; P8 - P3
TC1700007135.hg.1	TC1700007135.hg.1	NM_001037330	TRIM16L	147166	167.35	1.15E-10	9.9394	1.71E-09	P10 - ctrl; ctrl - P2; P3 - ctrl; P8 - ctrl; P10 - P11; P10 - P2; P8 - P10; P11 - P2; P3 - P11; P8 - P11; P3 - P2; P8 - P2; P8 - P3
TC0Y00007337.hg.1	TC0Y00007337.hg.1	moybu.aAug10	moybu	NA	167	1.16E-10	9.9339	1.72E-09	P10 - ctrl; ctrl - P11; P2 - ctrl; P3 - ctrl; P8 - ctrl; P10 - P11; P10 - P2; P10 - P3; P2 - P11; P3 - P11; P8 - P11; P8 - P2; P8 - P3
TC1900007465.hg.1	TC1900007465.hg.1	NM_052852	ZNF486	90649	166.63	1.18E-10	9.9283	1.73E-09	P10 - ctrl; ctrl - P11; P2 - ctrl; P3 - ctrl; P8 - ctrl; P10 - P11; P10 - P2; P10 - P3; P10 - P8; P2 - P11; P3 - P11; P8 - P11; P3 - P2; P8 - P2; P8 - P3
TC0200006677.hg.1	TC0200006677.hg.1	NM_001034	RRM2	6241	166.44	1.19E-10	9.9254	1.74E-09	P10 - ctrl; ctrl - P11; ctrl - P2; P8 - ctrl; P10 - P11; P10 - P2; P10 - P3; P8 - P10; P3 - P11; P8 - P11; P3 - P2; P8 - P2; P8 - P3
TC1000008891.hg.1	TC1000008891.hg.1	NM_004419	DUSP5	1847	165.99	1.21E-10	9.9185	1.75E-09	P10 - ctrl; P11 - ctrl; ctrl - P2; P3 - ctrl; P8 - ctrl; P10 - P11; P10 - P2; P11 - P2; P3 - P11; P8 - P11; P3 - P2; P8 - P2
TC1600009916.hg.1	TC1600009916.hg.1	NM_001310137	NPIPB11	728888	165.44	1.23E-10	9.91	1.77E-09	ctrl - P10; ctrl - P11; P2 - ctrl; ctrl - P3; ctrl - P8; P11 - P10; P2 - P10; P3 - P10; P8 - P10; P2 - P11; P11 - P8; P2 - P3; P2 - P8; P3 - P8
TC0300007915.hg.1	TC0300007915.hg.1	ENST00000461528.1	RP11-314M24.1		165.43	1.23E-10	9.9097	1.77E-09	P10 - ctrl; ctrl - P11; P2 - ctrl; ctrl - P3; P10 - P11; P2 - P10; P10 - P3; P10 - P8; P2 - P11; P11 - P3; P8 - P11; P2 - P3; P2 - P8; P8 - P3
TC0700011809.hg.1	TC0700011809.hg.1	NM_001271003	TFPI2	7980	164.77	1.26E-10	9.8996	1.80E-09	P10 - ctrl; P11 - ctrl; P3 - ctrl; P8 - ctrl; P10 - P11; P10 - P2; P8 - P10; P11 - P2; P3 - P11; P8 - P11; P3 - P2; P8 - P2; P8 - P3
TC0500011064.hg.1	TC0500011064.hg.1	NA	NA	NA	163.27	1.33E-10	9.8761	1.89E-09	ctrl - P10; ctrl - P11; P2 - ctrl; ctrl - P3; ctrl - P8; P11 - P10; P2 - P10; P3 - P10; P10 - P8; P2 - P11; P11 - P8; P2 - P3; P2 - P8; P3 - P8
TC0500013301.hg.1	TC0500013301.hg.1	NM_001244871	DAB2	1601	162.35	1.38E-10	9.8617	1.93E-09	ctrl - P10; ctrl - P2; ctrl - P3; ctrl - P8; P11 - P10; P2 - P10; P3 - P10; P11 - P2; P11 - P3; P11 - P8; P2 - P8; P3 - P8
TC0100015586.hg.1	TC0100015586.hg.1	NM_001201325	PDZK1	5174	162.34	1.38E-10	9.8615	1.93E-09	ctrl - P10; ctrl - P11; ctrl - P3; ctrl - P8; P11 - P10; P2 - P10; P10 - P8; P2 - P11; P11 - P3; P11 - P8; P2 - P3; P2 - P8
TC1600011501.hg.1	TC1600011501.hg.1	NM_130464	NPIPB3	23117	161.54	1.42E-10	9.8488	1.98E-09	ctrl - P10; ctrl - P11; P2 - ctrl; ctrl - P3; ctrl - P8; P11 - P10; P2 - P10; P3 - P10; P2 - P11; P11 - P8; P2 - P3; P2 - P8; P3 - P8
TC0800010002.hg.1	TC0800010002.hg.1	NM_001394	DUSP4	1846	161.23	1.43E-10	9.844	1.98E-09	P10 - ctrl; P3 - ctrl; P8 - ctrl; P10 - P11; P10 - P2; P11 - P2; P3 - P11; P8 - P11; P3 - P2; P8 - P2; P8 - P3
TC0800007673.hg.1	TC0800007673.hg.1	NA	NA	NA	161.2	1.43E-10	9.8434	1.98E-09	ctrl - P10; ctrl - P11; P2 - ctrl; P3 - ctrl; ctrl - P8; P10 - P11; P2 - P10; P3 - P10; P10 - P8; P2 - P11; P3 - P11; P11 - P8; P2 - P3; P2 - P8; P3 - P8
TC1800006897.hg.1	TC1800006897.hg.1	NM_000227	LAMA3	3909	161.03	1.44E-10	9.8408	1.98E-09	P10 - ctrl; P11 - ctrl; P3 - ctrl; P8 - ctrl; P10 - P11; P10 - P2; P3 - P10; P8 - P10; P11 - P2; P3 - P11; P8 - P11; P3 - P2; P8 - P2
TC0500007665.hg.1	TC0500007665.hg.1	NM_031966	CCNB1	891	160.92	1.45E-10	9.839	1.98E-09	P10 - ctrl; ctrl - P11; ctrl - P2; P3 - ctrl; P8 - ctrl; P10 - P11; P10 - P2; P10 - P3; P8 - P10; P3 - P11; P8 - P11; P3 - P2; P8 - P2; P8 - P3
TC0600011945.hg.1	TC0600011945.hg.1	NM_001251973	RCAN2	10231	160.72	1.46E-10	9.8359	1.98E-09	P10 - ctrl; ctrl - P11; ctrl - P2; P8 - ctrl; P10 - P11; P10 - P2; P10 - P3; P8 - P10; P3 - P11; P8 - P11; P3 - P2; P8 - P2; P8 - P3
TC1500008889.hg.1	TC1500008889.hg.1	NA	NA	NA	160.06	1.50E-10	9.8253	2.01E-09	ctrl - P10; ctrl - P11; P2 - ctrl; ctrl - P3; ctrl - P8; P11 - P10; P2 - P10; P3 - P10; P2 - P11; P3 - P11; P11 - P8; P2 - P3; P2 - P8; P3 - P8
TC0100008812.hg.1	TC0100008812.hg.1	NM_000959	PTGFR	5737	159.86	1.51E-10	9.8221	2.01E-09	ctrl - P10; ctrl - P11; ctrl - P2; ctrl - P3; ctrl - P8; P11 - P10; P2 - P10; P3 - P10; P11 - P2; P11 - P3; P11 - P8; P2 - P8; P3 - P8
TC0400006538.hg.1	TC0400006538.hg.1	NM_006342	TACC3	10460	159.85	1.51E-10	9.8219	2.01E-09	P10 - ctrl; ctrl - P11; ctrl - P2; P8 - ctrl; P10 - P11; P10 - P2; P10 - P3; P8 - P10; P11 - P2; P3 - P11; P8 - P11; P3 - P2; P8 - P2; P8 - P3
TC0900007667.hg.1	TC0900007667.hg.1	NM_021154	PSAT1	29968	158.76	1.57E-10	9.8045	2.07E-09	P10 - ctrl; ctrl - P2; P3 - ctrl; P8 - ctrl; P10 - P11; P10 - P2; P10 - P3; P11 - P2; P3 - P11; P8 - P11; P3 - P2; P8 - P2; P8 - P3
TC0500007738.hg.1	TC0500007738.hg.1	NM_005909	MAP1B	4131	158.76	1.57E-10	9.8044	2.07E-09	P10 - ctrl; ctrl - P11; P2 - ctrl; P3 - ctrl; P8 - ctrl; P10 - P11; P10 - P2; P10 - P3; P2 - P11; P3 - P11; P8 - P11; P3 - P2; P8 - P2; P8 - P3
TC0300011978.hg.1	TC0300011978.hg.1	ENST00000462510	RP11-615J4.4	NA	158.59	1.58E-10	9.8016	2.07E-09	P10 - ctrl; ctrl - P2; P3 - ctrl; P8 - ctrl; P10 - P11; P10 - P2; P10 - P3; P11 - P2; P3 - P11; P8 - P11; P3 - P2; P8 - P2; P8 - P3
TC0800007119.hg.1	TC0800007119.hg.1	NM_001017420	ESCO2	157570	158.5	1.58E-10	9.8003	2.07E-09	P10 - ctrl; ctrl - P11; ctrl - P2; P8 - ctrl; P10 - P11; P10 - P2; P10 - P3; P8 - P10; P2 - P11; P3 - P11; P8 - P11; P3 - P2; P8 - P2; P8 - P3
TC0600013205.hg.1	TC0600013205.hg.1	NM_001242350	VNN2	8875	158.42	1.59E-10	9.799	2.07E-09	P10 - ctrl; P2 - ctrl; P3 - ctrl; P8 - ctrl; P10 - P11; P10 - P2; P10 - P3; P10 - P8; P3 - P11; P8 - P11; P3 - P2; P8 - P2; P8 - P3
TC1000008628.hg.1	TC1000008628.hg.1	NM_000392	ABCC2	1244	157.96	1.62E-10	9.7914	2.09E-09	P10 - ctrl; P2 - ctrl; P3 - ctrl; P8 - ctrl; P10 - P11; P10 - P2; P3 - P10; P8 - P10; P3 - P11; P8 - P11; P3 - P2; P8 - P2; P3 - P8
TC0300014082.hg.1	TC0300014082.hg.1	NM_004454	ETV5	2119	157.81	1.63E-10	9.7891	2.09E-09	P10 - ctrl; P3 - ctrl; P8 - ctrl; P10 - P11; P10 - P2; P3 - P10; P8 - P10; P3 - P11; P8 - P11; P3 - P2; P8 - P2; P3 - P8

TC1900007242.hg.1	TC1900007242.hg.1	NR_015379	UCA1	652995	157.19	1.66E-10	9.7789	2.13E-09	P10 - ctrl; ctrl - P11; P3 - ctrl; P8 - ctrl; P10 - P11; P10 - P2; P3 - P11; P8 - P11; P3 - P2; P8 - P2
TC0500012017.hg.1	TC0500012017.hg.1	NM_002198	IRF1	3659	156.67	1.70E-10	9.7705	2.16E-09	P10 - ctrl; P11 - ctrl; ctrl - P2; P3 - ctrl; P8 - ctrl; P10 - P11; P10 - P2; P10 - P3; P8 - P10; P11 - P2; P3 - P11; P8 - P11; P3 - P2; P8 - P2; P8 - P3
TC0900009520.hg.1	TC0900009520.hg.1	NM_001040712	PTPRD	5789	156.34	1.72E-10	9.765	2.18E-09	ctrl - P10; ctrl - P11; ctrl - P2; ctrl - P3; ctrl - P8; P11 - P10; P2 - P10; P3 - P10; P11 - P2; P11 - P3; P11 - P8; P3 - P2; P2 - P8; P3 - P8
TC0800008759.hg.1	TC0800008759.hg.1	NM_001039112	FER1L6	654463	155.82	1.75E-10	9.7566	2.21E-09	P10 - ctrl; P11 - ctrl; P2 - ctrl; P3 - ctrl; P8 - ctrl; P10 - P11; P10 - P2; P8 - P10; P3 - P11; P8 - P11; P3 - P2; P8 - P2; P8 - P3
TC1700007319.hg.1	TC1700007319.hg.1	NM_015626	WSB1	26118	154.38	1.85E-10	9.7329	2.31E-09	ctrl - P10; ctrl - P11; ctrl - P2; ctrl - P3; ctrl - P8; P10 - P11; P2 - P10; P10 - P8; P2 - P11; P3 - P11; P11 - P8; P2 - P3; P2 - P8; P3 - P8
TC0400008483.hg.1	TC0400008483.hg.1	NM_001128174	UGT8	7368	154.34	1.85E-10	9.7322	2.31E-09	ctrl - P10; ctrl - P2; ctrl - P3; ctrl - P8; P11 - P10; P2 - P10; P10 - P8; P11 - P2; P11 - P3; P11 - P8; P2 - P3; P2 - P8; P3 - P8
TC0900007552.hg.1	TC0900007552.hg.1	NM_001242505	GDA	9615	154.29	1.86E-10	9.7314	2.31E-09	P10 - ctrl; P2 - ctrl; P3 - ctrl; P8 - ctrl; P10 - P11; P10 - P2; P10 - P3; P10 - P8; P2 - P11; P3 - P11; P8 - P11; P3 - P2; P8 - P2; P8 - P3
TC1_KI270706v1_random00006439.hg.1	TC1_KI270706v1_random00006439.hg.1	ENST00000413897.2	RP11-782C8.2		154.03	1.87E-10	9.7271	2.31E-09	P10 - ctrl; P11 - ctrl; P2 - ctrl; P3 - ctrl; P8 - ctrl; P10 - P11; P2 - P10; P3 - P10; P8 - P10; P2 - P11; P3 - P11; P8 - P11; P3 - P2; P3 - P8
TC0600008539.hg.1	TC0600008539.hg.1	NM_001159587	CD109	135228	153.96	1.88E-10	9.7258	2.31E-09	P10 - ctrl; ctrl - P11; P3 - ctrl; P8 - ctrl; P10 - P11; P10 - P2; P10 - P3; P8 - P10; P2 - P11; P3 - P11; P8 - P11; P3 - P2; P8 - P2; P8 - P3
TC0100007295.hg.1	TC0100007295.hg.1	NM_001309192	EPHB2	2048	153.07	1.95E-10	9.7111	2.36E-09	P10 - ctrl; ctrl - P11; ctrl - P2; P3 - ctrl; P8 - ctrl; P10 - P11; P10 - P2; P8 - P10; P11 - P2; P3 - P11; P8 - P11; P3 - P2; P8 - P2; P8 - P3
TC0100013223.hg.1	TC0100013223.hg.1	NM_001145657	RAP1GAP	5909	152.93	1.96E-10	9.7088	2.36E-09	ctrl - P10; P11 - ctrl; ctrl - P2; ctrl - P3; ctrl - P8; P11 - P10; P10 - P2; P10 - P3; P10 - P8; P11 - P2; P11 - P3; P11 - P8
TC0100016649.hg.1	TC0100016649.hg.1	NM_015039	NMNAT2	23057	152.81	1.97E-10	9.7066	2.36E-09	ctrl - P10; P11 - ctrl; ctrl - P2; P3 - ctrl; ctrl - P8; P11 - P10; P3 - P10; P8 - P10; P11 - P2; P11 - P8; P3 - P2; P3 - P8
TC0300008544.hg.1	TC0300008544.hg.1	NM_000388	CASR	846	152.73	1.97E-10	9.7054	2.36E-09	ctrl - P10; ctrl - P2; ctrl - P3; ctrl - P8; P11 - P10; P2 - P10; P8 - P10; P11 - P2; P11 - P3; P11 - P8; P2 - P3; P2 - P8
TC0800011611.hg.1	TC0800011611.hg.1	NM_002546	TNFRSF11B	4982	152.64	1.98E-10	9.7039	2.36E-09	P10 - ctrl; P11 - ctrl; P2 - ctrl; P3 - ctrl; P8 - ctrl; P10 - P11; P10 - P2; P10 - P3; P2 - P11; P3 - P11; P8 - P11; P3 - P2; P8 - P2; P8 - P3
TC1100009453.hg.1	TC1100009453.hg.1	NM_001254757	ST3GAL4	6484	152.63	1.98E-10	9.7037	2.36E-09	ctrl - P10; P11 - ctrl; ctrl - P2; ctrl - P3; ctrl - P8; P11 - P10; P2 - P10; P10 - P3; P11 - P2; P11 - P3; P11 - P8; P2 - P3; P2 - P8
TC0200014819.hg.1	TC0200014819.hg.1	NM_002977	SCN9A	6335	152.59	1.98E-10	9.7031	2.36E-09	ctrl - P10; ctrl - P11; ctrl - P2; ctrl - P3; ctrl - P8; P11 - P10; P2 - P10; P3 - P10; P11 - P3; P11 - P8; P2 - P3; P2 - P8; P3 - P8
TC2000008217.hg.1	TC2000008217.hg.1	NM_001174089	SLC4A11	83959	152.31	2.00E-10	9.6984	2.37E-09	P10 - ctrl; ctrl - P11; ctrl - P2; P3 - ctrl; P8 - ctrl; P10 - P11; P10 - P2; P10 - P3; P8 - P10; P3 - P11; P8 - P11; P3 - P2; P8 - P2; P8 - P3
TC0800010887.hg.1	TC0800010887.hg.1	NA	NA	NA	152.13	2.02E-10	9.6952	2.38E-09	P10 - ctrl; P8 - ctrl; P10 - P11; P10 - P2; P10 - P3; P8 - P11; P8 - P2; P8 - P3
TC0100011402.hg.1	TC0100011402.hg.1	NM_000715	C4BPA	722	151.96	2.03E-10	9.6925	2.38E-09	P10 - ctrl; P3 - ctrl; P8 - ctrl; P10 - P11; P10 - P2; P10 - P3; P10 - P8; P3 - P11; P8 - P11; P3 - P2; P8 - P2; P8 - P3
TC0X00007130.hg.1	TC0X00007130.hg.1	sperfor.aAug10-unspliced	sperfor	NA	151.4	2.07E-10	9.6831	2.42E-09	ctrl - P10; ctrl - P11; ctrl - P2; ctrl - P3; ctrl - P8; P11 - P10; P2 - P10; P3 - P10; P2 - P11; P11 - P8; P2 - P3; P2 - P8; P3 - P8
TC1200011991.hg.1	TC1200011991.hg.1	NM_138451	IQCD	115811	151.17	2.09E-10	9.6792	2.43E-09	P10 - ctrl; ctrl - P2; P3 - ctrl; P8 - ctrl; P10 - P11; P10 - P2; P8 - P10; P11 - P2; P3 - P11; P8 - P11; P3 - P2; P8 - P2; P8 - P3
HTA2-pos-PSR02023981.hg.1	23151074	NA	NA	NA	151.1	2.10E-10	9.6779	2.43E-09	ctrl - P10; ctrl - P11; P2 - ctrl; ctrl - P3; ctrl - P8; P11 - P10; P2 - P10; P3 - P10; P2 - P11; P11 - P8; P2 - P3; P2 - P8; P3 - P8
TC1600011507.hg.1	TC1600011507.hg.1	NPIPL3.iAug10	NPIPL3	NA	150.66	2.14E-10	9.6704	2.46E-09	ctrl - P10; ctrl - P11; P2 - ctrl; ctrl - P3; ctrl - P8; P2 - P10; P10 - P8; P2 - P11; P11 - P3; P11 - P8; P2 - P3; P2 - P8; P3 - P8
TC0100009770.hg.1	TC0100009770.hg.1	NM_001278141	NBPF12	149013	150.48	2.15E-10	9.6674	2.46E-09	ctrl - P10; ctrl - P11; ctrl - P3; ctrl - P8; P11 - P10; P2 - P10; P3 - P10; P2 - P11; P11 - P3; P11 - P8; P2 - P3; P2 - P8; P3 - P8
TC0300012924.hg.1	TC0300012924.hg.1	NM_001167911	VEPH1	79674	150.46	2.15E-10	9.6672	2.46E-09	ctrl - P10; ctrl - P2; ctrl - P3; ctrl - P8; P11 - P10; P2 - P10; P3 - P10; P11 - P2; P11 - P3; P11 - P8; P2 - P8; P3 - P8
TC1400007740.hg.1	TC1400007740.hg.1	ENST00000553613.1	RP11-7F17.7		149.53	2.23E-10	9.6512	2.54E-09	P10 - ctrl; P3 - ctrl; P8 - ctrl; P10 - P11; P10 - P2; P3 - P10; P8 - P10; P11 - P2; P3 - P11; P8 - P11; P3 - P2; P8 - P2
TC0700012230.hg.1	TC0700012230.hg.1	NM_001037132	NRCAM	4897	149.32	2.25E-10	9.6476	2.55E-09	P10 - ctrl; ctrl - P11; ctrl - P2; P3 - ctrl; P8 - ctrl; P10 - P11; P10 - P2; P8 - P10; P3 - P11; P8 - P11; P3 - P2; P8 - P2; P8 - P3
TC2100008391.hg.1	TC2100008391.hg.1	NA	NA	NA	149.16	2.26E-10	9.645	2.55E-09	ctrl - P10; ctrl - P11; ctrl - P2; ctrl - P3; ctrl - P8; P2 - P10; P3 - P10; P10 - P8; P2 - P11; P3 - P11; P11 - P8; P2 - P3; P2 - P8; P3 - P8
TC0600010626.hg.1	TC0600010626.hg.1	ENST00000566733.1	RP1-140K8.5		148.36	2.34E-10	9.6312	2.62E-09	ctrl - P10; P11 - ctrl; ctrl - P2; ctrl - P3; ctrl - P8; P11 - P10; P2 - P10; P10 - P3; P10 - P8; P11 - P2; P11 - P3; P11 - P8; P2 - P3; P2 - P8
TC0400006994.hg.1	TC0400006994.hg.1	NM_022346	NCAPG	64151	148.23	2.35E-10	9.6289	2.62E-09	P10 - ctrl; ctrl - P11; ctrl - P2; P8 - ctrl; P10 - P11; P10 - P2; P10 - P3; P8 - P10; P2 - P11; P3 - P11; P8 - P11; P3 - P2; P8 - P2; P8 - P3
TC1900009744.hg.1	TC1900009744.hg.1	NR_002751	SNORD41	19p13.2	147.78	2.39E-10	9.6212	2.65E-09	ctrl - P10; ctrl - P11; ctrl - P3; ctrl - P8; P11 - P10; P2 - P10; P2 - P11; P11 - P3; P11 - P8; P2 - P3; P2 - P8
TC1000012050.hg.1	TC1000012050.hg.1	NM_000141	FGFR2	2263	147.33	2.44E-10	9.6133	2.65E-09	ctrl - P10; ctrl - P2; ctrl - P3; ctrl - P8; P11 - P10; P2 - P10; P10 - P3; P11 - P2; P11 - P3; P11 - P8; P2 - P3; P2 - P8; P8 - P3
TC0300011815.hg.1	TC0300011815.hg.1	NM_080927	DCBLD2	131566	146.68	2.50E-10	9.6021	2.65E-09	P10 - ctrl; ctrl - P11; ctrl - P2; P3 - ctrl; P8 - ctrl; P10 - P11; P10 - P2; P3 - P10; P8 - P10; P3 - P11; P8 - P11; P3 - P2; P8 - P2

TC0300011005.hg.1	TC0300011005.hg.1	syfeybo.aAug10-unspliced	syfeybo	NA	146.39	2.53E-10	9.597	2.65E-09	ctrl - P10; ctrl - P11; P2 - ctrl; ctrl - P3; ctrl - P8; P2 - P10; P3 - P10; P10 - P8; P2 - P11; P3 - P11; P11 - P8; P2 - P3; P2 - P8; P3 - P8
TC1300007845.hg.1	TC1300007845.hg.1	NM_206808	CLYBL	171425	146.17	2.55E-10	9.5932	2.65E-09	ctrl - P10; P11 - ctrl; ctrl - P2; ctrl - P3; ctrl - P8; P11 - P10; P2 - P10; P10 - P3; P10 - P8; P11 - P2; P11 - P3; P11 - P8; P2 - P3; P2 - P8
TC0600013228.hg.1	TC0600013228.hg.1	NM_145176	SLC2A12	154091	146.14	2.55E-10	9.5927	2.65E-09	P10 - ctrl; ctrl - P11; ctrl - P2; ctrl - P3; P8 - ctrl; P10 - P11; P10 - P2; P10 - P3; P8 - P10; P11 - P2; P3 - P11; P8 - P11; P3 - P2; P8 - P2; P8 - P3
TC0200009537.hg.1	TC0200009537.hg.1	NM_001032998	KYNU	8942	146.12	2.56E-10	9.5923	2.65E-09	P10 - ctrl; P3 - ctrl; P8 - ctrl; P10 - P11; P10 - P2; P10 - P3; P3 - P11; P8 - P11; P3 - P2; P8 - P2; P8 - P3
TC0400010125.hg.1	TC0400010125.hg.1	NM_005130	FGFBP1	9982	145.99	2.57E-10	9.59	2.65E-09	P10 - ctrl; ctrl - P11; ctrl - P2; P3 - ctrl; P8 - ctrl; P10 - P11; P10 - P2; P3 - P10; P8 - P10; P11 - P2; P3 - P11; P8 - P11; P3 - P2; P8 - P2; P8 - P3
TC1700010767.hg.1	TC1700010767.hg.1	NR_002716	RNU2-1	6066	145.87	2.58E-10	9.5879	2.65E-09	P10 - ctrl; P11 - ctrl; P2 - ctrl; P3 - ctrl; P8 - ctrl; P11 - P10; P2 - P10; P3 - P10; P2 - P11; P3 - P11; P11 - P8; P2 - P8; P3 - P8
TC1700010768.hg.1	TC1700010768.hg.1	NR_002716_2	RNU2-1		145.87	2.58E-10	9.5879	2.65E-09	P10 - ctrl; P11 - ctrl; P2 - ctrl; P3 - ctrl; P8 - ctrl; P11 - P10; P2 - P10; P3 - P10; P2 - P11; P3 - P11; P11 - P8; P2 - P8; P3 - P8
TC1700010769.hg.1	TC1700010769.hg.1	NR_002716_3	RNU2-1		145.87	2.58E-10	9.5879	2.65E-09	P10 - ctrl; P11 - ctrl; P2 - ctrl; P3 - ctrl; P8 - ctrl; P11 - P10; P2 - P10; P3 - P10; P2 - P11; P3 - P11; P11 - P8; P2 - P8; P3 - P8
TC1700010770.hg.1	TC1700010770.hg.1	NR_002716_4	RNU2-1		145.87	2.58E-10	9.5879	2.65E-09	P10 - ctrl; P11 - ctrl; P2 - ctrl; P3 - ctrl; P8 - ctrl; P11 - P10; P2 - P10; P3 - P10; P2 - P11; P3 - P11; P11 - P8; P2 - P8; P3 - P8
TC1700010771.hg.1	TC1700010771.hg.1	NR_002716_5	RNU2-1		145.87	2.58E-10	9.5879	2.65E-09	P10 - ctrl; P11 - ctrl; P2 - ctrl; P3 - ctrl; P8 - ctrl; P11 - P10; P2 - P10; P3 - P10; P2 - P11; P3 - P11; P11 - P8; P2 - P8; P3 - P8
TC1700010772.hg.1	TC1700010772.hg.1	NR_002716_6	RNU2-1		145.87	2.58E-10	9.5879	2.65E-09	P10 - ctrl; P11 - ctrl; P2 - ctrl; P3 - ctrl; P8 - ctrl; P11 - P10; P2 - P10; P3 - P10; P2 - P11; P3 - P11; P11 - P8; P2 - P8; P3 - P8
TC1700010773.hg.1	TC1700010773.hg.1	uc060ftg.1	U2	NA	145.87	2.58E-10	9.5879	2.65E-09	P10 - ctrl; P11 - ctrl; P2 - ctrl; P3 - ctrl; P8 - ctrl; P11 - P10; P2 - P10; P3 - P10; P2 - P11; P3 - P11; P11 - P8; P2 - P8; P3 - P8
TC1700010774.hg.1	TC1700010774.hg.1	uc032fjc.2	U2	NA	145.87	2.58E-10	9.5879	2.65E-09	P10 - ctrl; P11 - ctrl; P2 - ctrl; P3 - ctrl; P8 - ctrl; P11 - P10; P2 - P10; P3 - P10; P2 - P11; P3 - P11; P11 - P8; P2 - P8; P3 - P8
TC1700010775.hg.1	TC1700010775.hg.1	NR_002716_7	RNU2-1		145.87	2.58E-10	9.5879	2.65E-09	P10 - ctrl; P11 - ctrl; P2 - ctrl; P3 - ctrl; P8 - ctrl; P11 - P10; P2 - P10; P3 - P10; P2 - P11; P3 - P11; P11 - P8; P2 - P8; P3 - P8
TC1700010776.hg.1	TC1700010776.hg.1	NR_002716_8	RNU2-1		145.87	2.58E-10	9.5879	2.65E-09	P10 - ctrl; P11 - ctrl; P2 - ctrl; P3 - ctrl; P8 - ctrl; P11 - P10; P2 - P10; P3 - P10; P2 - P11; P3 - P11; P11 - P8; P2 - P8; P3 - P8
TC1700010778.hg.1	TC1700010778.hg.1	NR_002716_9	RNU2-1		145.87	2.58E-10	9.5879	2.65E-09	P10 - ctrl; P11 - ctrl; P2 - ctrl; P3 - ctrl; P8 - ctrl; P11 - P10; P2 - P10; P3 - P10; P2 - P11; P3 - P11; P11 - P8; P2 - P8; P3 - P8
TC1700010779.hg.1	TC1700010779.hg.1	NR_002716_10	RNU2-1		145.87	2.58E-10	9.5879	2.65E-09	P10 - ctrl; P11 - ctrl; P2 - ctrl; P3 - ctrl; P8 - ctrl; P11 - P10; P2 - P10; P3 - P10; P2 - P11; P3 - P11; P11 - P8; P2 - P8; P3 - P8
TC1500007062.hg.1	TC1500007062.hg.1	NM_020990	CKMT1B	1159	144.81	2.70E-10	9.5693	2.75E-09	P10 - ctrl; P11 - ctrl; ctrl - P2; P3 - ctrl; P8 - ctrl; P10 - P11; P10 - P2; P10 - P3; P11 - P2; P8 - P11; P3 - P2; P8 - P2; P8 - P3
TC1400008106.hg.1	TC1400008106.hg.1	NR_038861	TUNAR	1.01E+08	144.38	2.74E-10	9.5617	2.79E-09	P10 - ctrl; P11 - ctrl; P2 - ctrl; P3 - ctrl; P8 - ctrl; P10 - P11; P10 - P2; P10 - P3; P8 - P10; P2 - P11; P3 - P11; P8 - P11; P3 - P2; P8 - P2; P8 - P3
TC0200011138.hg.1	TC0200011138.hg.1	NM_000463	UGT1A1	54658	144.11	2.77E-10	9.5569	2.81E-09	P10 - ctrl; P11 - ctrl; P2 - ctrl; P3 - ctrl; P8 - ctrl; P10 - P11; P10 - P2; P10 - P3; P11 - P2; P8 - P11; P3 - P2; P8 - P2; P8 - P3
TC1400008382.hg.1	TC1400008382.hg.1	NM_006291	TNFAIP2	7127	143.91	2.80E-10	9.5533	2.82E-09	P10 - ctrl; ctrl - P11; P3 - ctrl; P8 - ctrl; P10 - P11; P10 - P2; P10 - P3; P8 - P10; P2 - P11; P3 - P11; P8 - P11; P3 - P2; P8 - P2; P8 - P3
TC0500009211.hg.1	TC0500009211.hg.1	NM_001037333	CYFIP2	26999	143.41	2.85E-10	9.5445	2.86E-09	ctrl - P10; P11 - ctrl; ctrl - P2; ctrl - P3; ctrl - P8; P11 - P10; P11 - P2; P11 - P3; P11 - P8
TC0600011232.hg.1	TC0600011232.hg.1	NM_005322	HIST1H1B	3009	143.33	2.86E-10	9.543	2.86E-09	P10 - ctrl; P3 - ctrl; P8 - ctrl; P10 - P11; P10 - P2; P10 - P3; P3 - P11; P8 - P11; P3 - P2; P8 - P2; P8 - P3
TC1600009959.hg.1	TC1600009959.hg.1	NR_002453	SMG1P5	595101	141.99	3.03E-10	9.519	3.01E-09	ctrl - P10; ctrl - P11; P2 - ctrl; ctrl - P3; ctrl - P8; P11 - P10; P2 - P10; P10 - P8; P2 - P11; P11 - P3; P11 - P8; P2 - P3; P2 - P8
TC0800012462.hg.1	TC0800012462.hg.1	NR_120364	CASC19	1.03E+08	141.78	3.05E-10	9.5153	3.03E-09	P10 - ctrl; P2 - ctrl; P3 - ctrl; P8 - ctrl; P10 - P11; P10 - P2; P3 - P10; P8 - P10; P2 - P11; P3 - P11; P8 - P11; P3 - P2; P8 - P2; P3 - P8
TC1600011364.hg.1	TC1600011364.hg.1	NM_001135865	NPIPB5	1E+08	139.91	3.30E-10	9.4815	3.26E-09	ctrl - P10; ctrl - P11; P2 - ctrl; ctrl - P3; ctrl - P8; P11 - P10; P2 - P10; P3 - P10; P2 - P11; P11 - P8; P2 - P3; P2 - P8; P3 - P8
TC0100015234.hg.1	TC0100015234.hg.1	NM_001201546	SLC16A4	9122	139.77	3.32E-10	9.4789	3.27E-09	ctrl - P10; ctrl - P2; ctrl - P8; P11 - P10; P2 - P10; P3 - P10; P11 - P2; P11 - P8; P3 - P2; P2 - P8; P3 - P8
TC0700013623.hg.1	TC0700013623.hg.1	NM_001135914	KCP	7q32.1	139.51	3.36E-10	9.4741	3.29E-09	ctrl - P10; ctrl - P11; ctrl - P2; ctrl - P3; ctrl - P8; P11 - P10; P2 - P10; P10 - P3; P10 - P8; P11 - P3; P11 - P8; P2 - P3; P2 - P8
TC1700008231.hg.1	TC1700008231.hg.1	OTTHUMT00000473570	RP5-875H18.9		139.34	3.38E-10	9.4709	3.30E-09	P10 - ctrl; ctrl - P11; P3 - ctrl; P8 - ctrl; P10 - P11; P10 - P2; P3 - P10; P8 - P10; P2 - P11; P3 - P11; P8 - P11; P3 - P2; P8 - P2
TC1700008228.hg.1	TC1700008228.hg.1	NM_002204	ITGA3	3675	138.98	3.43E-10	9.4643	3.34E-09	P10 - ctrl; ctrl - P2; P3 - ctrl; P8 - ctrl; P10 - P11; P10 - P2; P3 - P10; P8 - P10; P11 - P2; P3 - P11; P8 - P11; P3 - P2; P8 - P2; P3 - P8
TC1700012285.hg.1	TC1700012285.hg.1	NM_018304	PRR11	55771	138.68	3.48E-10	9.4589	3.36E-09	P10 - ctrl; ctrl - P11; ctrl - P2; P3 - ctrl; P8 - ctrl; P10 - P11; P10 - P2; P8 - P10; P11 - P2; P3 - P11; P8 - P11; P3 - P2; P8 - P2; P8 - P3
TC0700007434.hg.1	TC0700007434.hg.1	NM_000596	IGFBP1	3484	138.66	3.48E-10	9.4585	3.36E-09	P10 - ctrl; P11 - ctrl; P2 - ctrl; P3 - ctrl; P8 - ctrl; P10 - P11; P10 - P2; P10 - P3; P8 - P10; P2 - P11; P3 - P11; P8 - P11; P3 - P2; P8 - P2; P8 - P3

TC0100013349.hg.1	TC0100013349.hg.1	NM_020317	RSRP1	1p36.13-p35.1	138.54	3.50E-10	9.4563	3.36E-09	ctrl - P10; ctrl - P11; ctrl - P2; ctrl - P3; ctrl - P8; P11 - P10; P2 - P10; P10 - P3; P10 - P8; P2 - P11; P11 - P3; P11 - P8; P2 - P3; P2 - P8
TC0100018059.hg.1	TC0100018059.hg.1	NA	NA	NA	138.37	3.52E-10	9.4531	3.36E-09	ctrl - P10; ctrl - P11; P2 - ctrl; ctrl - P3; ctrl - P8; P11 - P10; P2 - P10; P3 - P10; P10 - P8; P2 - P11; P3 - P11; P11 - P8; P2 - P3; P2 - P8; P3 - P8
TC0900010261.hg.1	TC0900010261.hg.1	vuvybu.aAug10-unspliced	vuvybu	NA	138.37	3.52E-10	9.4531	3.36E-09	ctrl - P10; ctrl - P11; P2 - ctrl; P3 - ctrl; ctrl - P8; P10 - P11; P2 - P10; P3 - P10; P10 - P8; P2 - P11; P3 - P11; P8 - P11; P2 - P3; P2 - P8; P3 - P8
TC0300009774.hg.1	TC0300009774.hg.1	NM_000412	HRG	3273	137.78	3.61E-10	9.4423	3.43E-09	ctrl - P10; ctrl - P11; ctrl - P2; ctrl - P3; ctrl - P8; P11 - P10; P11 - P2; P11 - P3; P11 - P8
TC1500008988.hg.1	TC1500008988.hg.1	NM_181077	GOLGA8A	23015	137.72	3.62E-10	9.4411	3.43E-09	ctrl - P10; ctrl - P11; ctrl - P2; ctrl - P3; ctrl - P8; P11 - P10; P2 - P10; P3 - P10; P2 - P11; P11 - P8; P2 - P3; P2 - P8; P3 - P8
TC1500008888.hg.1	TC1500008888.hg.1	NA	NA	NA	137.64	3.63E-10	9.4397	3.43E-09	ctrl - P10; ctrl - P11; P2 - ctrl; ctrl - P3; ctrl - P8; P2 - P10; P3 - P10; P10 - P8; P2 - P11; P3 - P11; P11 - P8; P2 - P3; P2 - P8; P3 - P8
TC0200007976.hg.1	TC0200007976.hg.1	NM_001692	ATP6V1B1	2p13.1	137.54	3.65E-10	9.4377	3.43E-09	ctrl - P10; P11 - ctrl; ctrl - P2; ctrl - P3; ctrl - P8; P11 - P10; P11 - P2; P11 - P3; P11 - P8; P3 - P2
TC0900008150.hg.1	TC0900008150.hg.1	NM_001166116	TMOD1	7111	137.05	3.73E-10	9.4286	3.49E-09	P10 - ctrl; P3 - ctrl; P8 - ctrl; P10 - P11; P10 - P2; P8 - P10; P3 - P11; P8 - P11; P3 - P2; P8 - P2; P8 - P3
TC0700013388.hg.1	TC0700013388.hg.1	NR_040583	STAG3L1	54441	136.85	3.76E-10	9.4251	3.50E-09	ctrl - P10; ctrl - P11; ctrl - P2; ctrl - P3; ctrl - P8; P11 - P10; P2 - P10; P3 - P10; P2 - P11; P11 - P8; P2 - P3; P2 - P8; P3 - P8
TC1800007471.hg.1	TC1800007471.hg.1	NM_021127	PMAIP1	5366	136.74	3.78E-10	9.4229	3.51E-09	P10 - ctrl; P11 - ctrl; ctrl - P2; P3 - ctrl; P8 - ctrl; P10 - P11; P10 - P2; P8 - P10; P11 - P2; P3 - P11; P8 - P11; P3 - P2; P8 - P2; P8 - P3
TC0600014255.hg.1	TC0600014255.hg.1	ENST00000377050	UBD	10537	136.61	3.80E-10	9.4205	3.51E-09	P10 - ctrl; P8 - ctrl; P10 - P11; P10 - P2; P10 - P3; P8 - P10; P8 - P11; P8 - P2; P8 - P3
TC0600011173.hg.1	TC0600011173.hg.1	NR_003504	GUSBP2	387036	136.52	3.81E-10	9.4188	3.52E-09	ctrl - P10; ctrl - P11; P2 - ctrl; ctrl - P3; ctrl - P8; P11 - P10; P2 - P10; P3 - P10; P10 - P8; P2 - P11; P11 - P3; P11 - P8; P2 - P3; P2 - P8; P3 - P8
TC0X00010989.hg.1	TC0X00010989.hg.1	NM_012317	LDOC1	23641	136.29	3.85E-10	9.4145	3.54E-09	ctrl - P10; ctrl - P2; ctrl - P3; ctrl - P8; P11 - P10; P3 - P10; P11 - P2; P11 - P3; P11 - P8; P3 - P2; P3 - P8
TC0900008891.hg.1	TC0900008891.hg.1	NM_001127244	LRRC8A	56262	136.17	3.87E-10	9.4123	3.54E-09	P10 - ctrl; ctrl - P2; P3 - ctrl; P8 - ctrl; P10 - P11; P10 - P2; P3 - P10; P8 - P10; P11 - P2; P3 - P11; P8 - P11; P3 - P2; P8 - P2
TC1200006896.hg.1	TC1200006896.hg.1	NM_003979	GPRC5A	9052	136.13	3.88E-10	9.4116	3.54E-09	P10 - ctrl; ctrl - P2; P3 - ctrl; P8 - ctrl; P10 - P11; P10 - P2; P11 - P2; P3 - P11; P8 - P11; P3 - P2; P8 - P2; P8 - P3
TC0100008057.hg.1	TC0100008057.hg.1	NM_001255	CDC20	991	135.88	3.92E-10	9.4068	3.55E-09	P10 - ctrl; ctrl - P11; ctrl - P2; P8 - ctrl; P10 - P11; P10 - P2; P10 - P3; P8 - P10; P3 - P11; P8 - P11; P3 - P2; P8 - P2; P8 - P3
TC2200007356.hg.1	TC2200007356.hg.1	NM_006855	KDELR3	11015	135.87	3.92E-10	9.4067	3.55E-09	P10 - ctrl; ctrl - P11; ctrl - P2; P3 - ctrl; P8 - ctrl; P10 - P11; P10 - P2; P3 - P10; P8 - P10; P11 - P2; P3 - P11; P8 - P11; P3 - P2; P8 - P2
TC0500012138.hg.1	TC0500012138.hg.1	NM_004598	SPOCK1	5q31.2	135.5	3.98E-10	9.3997	3.59E-09	ctrl - P10; ctrl - P2; P3 - ctrl; ctrl - P8; P11 - P10; P2 - P10; P3 - P10; P11 - P2; P3 - P11; P11 - P8; P3 - P2; P2 - P8; P3 - P8
TC0400011738.hg.1	TC0400011738.hg.1	NM_198179	QRFPR	84109	135.44	3.99E-10	9.3985	3.59E-09	ctrl - P10; P11 - ctrl; ctrl - P2; ctrl - P3; ctrl - P8; P11 - P10; P2 - P10; P3 - P10; P8 - P10; P11 - P2; P11 - P3; P11 - P8; P3 - P2; P2 - P8; P3 - P8
TC1800006592.hg.1	TC1800006592.hg.1	leeterbu.aAug10-unspliced	leeterbu	NA	135.37	4.01E-10	9.3972	3.59E-09	ctrl - P10; ctrl - P11; P2 - ctrl; ctrl - P3; ctrl - P8; P2 - P10; P3 - P10; P10 - P8; P2 - P11; P3 - P11; P11 - P8; P2 - P3; P2 - P8; P3 - P8
TC2100007543.hg.1	TC2100007543.hg.1	ENST00000624041	bP-21264C1.1	NA	135.03	4.07E-10	9.3908	3.63E-09	P10 - ctrl; P2 - ctrl; P3 - ctrl; P8 - ctrl; P10 - P11; P2 - P10; P3 - P10; P8 - P10; P2 - P11; P3 - P11; P8 - P11; P3 - P2; P2 - P8; P3 - P8
TC1700010994.hg.1	TC1700010994.hg.1	skozy.aAug10-unspliced	skozy	NA	134.78	4.11E-10	9.3861	3.66E-09	ctrl - P10; ctrl - P11; ctrl - P3; ctrl - P8; P11 - P10; P2 - P10; P3 - P10; P2 - P11; P11 - P3; P11 - P8; P2 - P3; P2 - P8; P3 - P8
TC0800010716.hg.1	TC0800010716.hg.1	NR_038877	C8orf34-AS1	286189	134.24	4.21E-10	9.3759	3.73E-09	P10 - ctrl; ctrl - P11; P2 - ctrl; P3 - ctrl; P8 - ctrl; P10 - P11; P10 - P2; P10 - P3; P2 - P11; P3 - P11; P8 - P11; P3 - P2; P8 - P2; P8 - P3
TC1100011602.hg.1	TC1100011602.hg.1	NM_003355	UCP2	7351	134.09	4.24E-10	9.373	3.74E-09	P10 - ctrl; ctrl - P11; ctrl - P2; P3 - ctrl; P8 - ctrl; P10 - P11; P10 - P2; P10 - P3; P2 - P11; P3 - P11; P8 - P11; P3 - P2; P8 - P2; P8 - P3
TC1600009917.hg.1	TC1600009917.hg.1	NA	NA	NA	133.71	4.31E-10	9.3658	3.79E-09	ctrl - P10; ctrl - P11; P2 - ctrl; ctrl - P3; ctrl - P8; P11 - P10; P2 - P10; P3 - P10; P8 - P10; P2 - P11; P11 - P3; P11 - P8; P2 - P3; P2 - P8; P3 - P8
TC0900007815.hg.1	TC0900007815.hg.1	warteyby.aAug10-unspliced	warteyby	NA	133.59	4.33E-10	9.3635	3.80E-09	ctrl - P10; ctrl - P11; ctrl - P3; ctrl - P8; P11 - P10; P2 - P10; P3 - P10; P2 - P11; P11 - P8; P2 - P3; P2 - P8; P3 - P8
TC0500009603.hg.1	TC0500009603.hg.1	NM_133369	UNC5A	90249	132.65	4.51E-10	9.3455	3.94E-09	P10 - ctrl; ctrl - P2; P3 - ctrl; P8 - ctrl; P10 - P11; P10 - P2; P10 - P3; P8 - P10; P11 - P2; P3 - P11; P8 - P11; P3 - P2; P8 - P2; P8 - P3
TC1200008921.hg.1	TC1200008921.hg.1	NM_001032731	OAS2	4939	132.59	4.53E-10	9.3444	3.94E-09	P10 - ctrl; P11 - ctrl; P3 - ctrl; P8 - ctrl; P10 - P11; P10 - P2; P10 - P3; P11 - P2; P3 - P11; P8 - P11; P3 - P2; P8 - P2; P8 - P3
TC0300008839.hg.1	TC0300008839.hg.1	NM_001099	ACPP	55	132.37	4.57E-10	9.3401	3.96E-09	ctrl - P10; ctrl - P2; ctrl - P3; ctrl - P8; P11 - P10; P2 - P10; P3 - P10; P11 - P2; P11 - P3; P11 - P8; P2 - P3; P2 - P8; P3 - P8
TC0200013912.hg.1	TC0200013912.hg.1	NM_001304361	CKAP2L	150468	132.32	4.58E-10	9.3391	3.96E-09	P10 - ctrl; ctrl - P11; ctrl - P2; P8 - ctrl; P10 - P11; P10 - P2; P10 - P3; P8 - P10; P3 - P11; P8 - P11; P3 - P2; P8 - P2; P8 - P3
TSUnmapped00000784.hg.1	TSUnmapped00000784.hg.1	ENST00000631074	PADI1	29943	131.58	4.73E-10	9.3249	4.07E-09	P10 - ctrl; P3 - ctrl; P8 - ctrl; P10 - P11; P10 - P2; P8 - P10; P3 - P11; P8 - P11; P3 - P2; P8 - P2
TC1800008349.hg.1	TC1800008349.hg.1	NM_001256316	CHST9	83539	131.54	4.74E-10	9.324	4.07E-09	ctrl - P10; ctrl - P8; P11 - P10; P2 - P10; P3 - P10; P8 - P10; P11 - P8; P2 - P8; P3 - P8
TC0800010397.hg.1	TC0800010397.hg.1	ENST00000518968	PSAT1P1	NA	131.18	4.82E-10	9.3172	4.12E-09	P10 - ctrl; P3 - ctrl; P8 - ctrl; P10 - P11; P10 - P2; P10 - P3; P3 - P11; P8 - P11; P3 - P2; P8 - P2; P8 - P3

TC0300007191.hg.1	TC0300007191.hg.1	ENST00000462240	RN7SL517P		130.37	5.00E-10	9.3013	4.26E-09	ctrl - P10; ctrl - P11; ctrl - P3; ctrl - P8; P11 - P10; P2 - P10; P3 - P10; P10 - P8; P2 - P11; P11 - P3; P11 - P8; P2 - P3; P2 - P8; P3 - P8
TC0600007847.hg.1	TC0600007847.hg.1	NM_000389	CDKN1A	1026	130.26	5.02E-10	9.299	4.27E-09	P10 - ctrl; P11 - ctrl; P3 - ctrl; P8 - ctrl; P10 - P2; P11 - P2; P3 - P2; P8 - P2
TC0100014081.hg.1	TC0100014081.hg.1	NM_005764	PDZK1IP1	10158	130.16	5.04E-10	9.2973	4.27E-09	P10 - ctrl; ctrl - P2; P3 - ctrl; P8 - ctrl; P10 - P11; P10 - P2; P10 - P3; P11 - P2; P3 - P11; P8 - P11; P3 - P2; P8 - P2; P8 - P3
TC0800010382.hg.1	TC0800010382.hg.1	NM_003068	SNAI2	6591	130.1	5.06E-10	9.296	4.27E-09	P10 - ctrl; P3 - ctrl; P8 - ctrl; P10 - P11; P10 - P2; P10 - P3; P3 - P11; P8 - P11; P3 - P2; P8 - P2; P8 - P3
TC0200012163.hg.1	TC0200012163.hg.1	NM_000379	XDH	7498	129.79	5.13E-10	9.2899	4.31E-09	P10 - ctrl; P3 - ctrl; P8 - ctrl; P10 - P11; P10 - P2; P3 - P11; P8 - P11; P3 - P2; P8 - P2
TC1600011375.hg.1	TC1600011375.hg.1	NM_014298	QPRT	23475	129.72	5.15E-10	9.2886	4.31E-09	ctrl - P10; ctrl - P11; ctrl - P2; ctrl - P8; P11 - P10; P2 - P10; P3 - P10; P8 - P10; P11 - P2; P11 - P8; P3 - P2; P3 - P8
TC0500011528.hg.1	TC0500011528.hg.1	NM_153234	LIX1	167410	129.62	5.17E-10	9.2866	4.31E-09	ctrl - P10; ctrl - P11; ctrl - P2; ctrl - P3; ctrl - P8; P11 - P10; P2 - P10; P3 - P10; P8 - P10; P11 - P2; P11 - P3; P11 - P8; P3 - P2; P2 - P8; P3 - P8
TC0400011748.hg.1	TC0400011748.hg.1	NM_001237	CCNA2	890	129.6	5.17E-10	9.2862	4.31E-09	P10 - ctrl; ctrl - P11; ctrl - P2; P8 - ctrl; P10 - P11; P10 - P2; P10 - P3; P8 - P10; P3 - P11; P8 - P11; P3 - P2; P8 - P2; P8 - P3
TC1800008094.hg.1	TC1800008094.hg.1	ENST00000609238.1	RP11-78A19.4		129.37	5.23E-10	9.2817	4.34E-09	P10 - ctrl; ctrl - P11; P3 - ctrl; P8 - ctrl; P10 - P11; P10 - P2; P10 - P3; P2 - P11; P3 - P11; P8 - P11; P3 - P2; P8 - P2; P8 - P3
TC0700011953.hg.1	TC0700011953.hg.1	NM_000777	CYP3A5	1577	128.39	5.47E-10	9.2622	4.52E-09	P10 - ctrl; P3 - ctrl; P8 - ctrl; P10 - P11; P10 - P2; P3 - P10; P8 - P10; P2 - P11; P3 - P11; P8 - P11; P3 - P2; P8 - P2; P3 - P8
TC1500010369.hg.1	TC1500010369.hg.1	NM_001114614	MFGE8	4240	127.9	5.59E-10	9.2526	4.61E-09	P10 - ctrl; ctrl - P11; ctrl - P2; P3 - ctrl; P8 - ctrl; P10 - P11; P10 - P2; P10 - P3; P3 - P11; P8 - P11; P3 - P2; P8 - P2; P8 - P3
TC1400009248.hg.1	TC1400009248.hg.1	NM_001146015	DLGAP5	9787	127.28	5.75E-10	9.2402	4.70E-09	P10 - ctrl; ctrl - P11; ctrl - P2; P8 - ctrl; P10 - P11; P10 - P2; P10 - P3; P8 - P10; P11 - P2; P3 - P11; P8 - P11; P3 - P2; P8 - P2; P8 - P3
TC0900011305.hg.1	TC0900011305.hg.1	NM_002160	TNC	3371	127.19	5.78E-10	9.2384	4.70E-09	ctrl - P10; ctrl - P11; ctrl - P2; P3 - ctrl; ctrl - P8; P11 - P10; P2 - P10; P3 - P10; P8 - P10; P3 - P11; P8 - P11; P3 - P2; P8 - P2; P3 - P8
TC0500013189.hg.1	TC0500013189.hg.1	ENST00000427584.2	RP11-60A8.1		127.19	5.78E-10	9.2384	4.70E-09	ctrl - P10; ctrl - P11; ctrl - P2; ctrl - P3; ctrl - P8; P11 - P10; P2 - P10; P11 - P2; P11 - P3; P11 - P8; P2 - P3; P2 - P8
TC0600006967.hg.1	TC0600006967.hg.1	NM_001168319	EDN1	1906	127.18	5.78E-10	9.2381	4.70E-09	P10 - ctrl; ctrl - P11; P2 - ctrl; P3 - ctrl; P8 - ctrl; P10 - P11; P10 - P2; P10 - P3; P8 - P10; P2 - P11; P3 - P11; P8 - P11; P3 - P2; P8 - P2; P8 - P3
TC0100017541.hg.1	TC0100017541.hg.1	NA	NA	NA	127.1	5.80E-10	9.2365	4.71E-09	ctrl - P10; ctrl - P11; ctrl - P2; ctrl - P3; ctrl - P8; P11 - P10; P2 - P10; P3 - P10; P11 - P3; P11 - P8; P2 - P3; P2 - P8; P3 - P8
TC1100012545.hg.1	TC1100012545.hg.1	NM_012101	TRIM29	23650	126.4	5.99E-10	9.2225	4.85E-09	P10 - ctrl; P11 - ctrl; ctrl - P2; P3 - ctrl; P8 - ctrl; P10 - P2; P3 - P10; P8 - P10; P11 - P2; P3 - P11; P8 - P11; P3 - P2; P8 - P2
TC1100007273.hg.1	TC1100007273.hg.1	NM_000610	CD44	960	126.13	6.07E-10	9.217	4.89E-09	P10 - ctrl; P3 - ctrl; P8 - ctrl; P10 - P11; P10 - P2; P3 - P10; P8 - P10; P3 - P11; P8 - P11; P3 - P2; P8 - P2; P3 - P8
TC0500010835.hg.1	TC0500010835.hg.1	NM_001252226	PLK2	10769	125.88	6.14E-10	9.2119	4.93E-09	P10 - ctrl; ctrl - P11; P2 - ctrl; P3 - ctrl; P8 - ctrl; P10 - P11; P10 - P2; P3 - P10; P2 - P11; P3 - P11; P8 - P11; P3 - P2; P8 - P2
TC0900008955.hg.1	TC0900008955.hg.1	NA	NA	NA	125.39	6.28E-10	9.202	5.03E-09	ctrl - P10; ctrl - P11; ctrl - P3; ctrl - P8; P11 - P10; P2 - P10; P10 - P3; P10 - P8; P2 - P11; P11 - P3; P11 - P8; P2 - P3; P2 - P8
TC1700008598.hg.1	TC1700008598.hg.1	NM_001085423	MILR1	284021	125.1	6.37E-10	9.1962	5.08E-09	P10 - ctrl; P2 - ctrl; P3 - ctrl; P8 - ctrl; P10 - P11; P10 - P2; P10 - P3; P2 - P11; P3 - P11; P8 - P11; P3 - P2; P8 - P2; P8 - P3
TC0200015650.hg.1	TC0200015650.hg.1	NM_001306129	FN1	2335	124.06	6.69E-10	9.1748	5.32E-09	ctrl - P11; ctrl - P2; P3 - ctrl; P8 - ctrl; P10 - P11; P10 - P2; P3 - P10; P8 - P10; P3 - P11; P8 - P11; P3 - P2; P8 - P2; P3 - P8
TC1300009646.hg.1	TC1300009646.hg.1	NM_052867	NALCN	259232	123.83	6.76E-10	9.1702	5.36E-09	P10 - ctrl; ctrl - P11; ctrl - P2; P3 - ctrl; P8 - ctrl; P10 - P11; P10 - P2; P3 - P10; P8 - P10; P3 - P11; P8 - P11; P3 - P2; P8 - P2; P8 - P3
TC0800007662.hg.1	TC0800007662.hg.1	NM_022454	SOX17	64321	123.4	6.90E-10	9.1614	5.45E-09	P10 - ctrl; P11 - ctrl; P3 - ctrl; P8 - ctrl; P10 - P11; P10 - P2; P10 - P3; P8 - P10; P11 - P2; P3 - P11; P8 - P11; P3 - P2; P8 - P2; P8 - P3
TC1100008236.hg.1	TC1100008236.hg.1	NM_053056	CCND1	595	123.29	6.93E-10	9.159	5.45E-09	P10 - ctrl; ctrl - P2; P3 - ctrl; P8 - ctrl; P10 - P11; P10 - P2; P3 - P10; P8 - P10; P11 - P2; P3 - P11; P8 - P11; P3 - P2; P8 - P2
TC0400012949.hg.1	TC0400012949.hg.1	NM_001135146	SLC39A8	64116	123.24	6.95E-10	9.158	5.45E-09	P10 - ctrl; ctrl - P2; P3 - ctrl; P8 - ctrl; P10 - P11; P10 - P2; P10 - P3; P11 - P2; P3 - P11; P8 - P11; P3 - P2; P8 - P2; P8 - P3
TC1600006483.hg.1	TC1600006483.hg.1	NM_001177355	MSLN	10232	123.18	6.97E-10	9.1568	5.45E-09	P10 - ctrl; P11 - ctrl; P3 - ctrl; P8 - ctrl; P10 - P11; P10 - P2; P10 - P3; P11 - P2; P3 - P11; P8 - P11; P3 - P2; P8 - P2; P8 - P3
TC0200008663.hg.1	TC0200008663.hg.1	NM_001261419	IL1R2	7850	123.16	6.98E-10	9.1564	5.45E-09	P10 - ctrl; P11 - ctrl; ctrl - P2; P3 - ctrl; P8 - ctrl; P10 - P2; P3 - P10; P8 - P10; P11 - P2; P3 - P11; P8 - P11; P3 - P2; P8 - P2; P8 - P3
TC1200010229.hg.1	TC1200010229.hg.1	NM_002820	PTHLH	5744	122.91	7.06E-10	9.1511	5.49E-09	P10 - ctrl; P3 - ctrl; P8 - ctrl; P10 - P11; P10 - P2; P3 - P10; P8 - P10; P11 - P2; P3 - P11; P8 - P11; P3 - P2; P8 - P2
TC0100013028.hg.1	TC0100013028.hg.1	NM_004431	EPHA2	1969	122.85	7.08E-10	9.15	5.49E-09	P10 - ctrl; ctrl - P11; ctrl - P2; P3 - ctrl; P8 - ctrl; P10 - P11; P10 - P2; P3 - P10; P8 - P10; P11 - P2; P3 - P11; P8 - P11; P3 - P2; P8 - P2
TC0700013297.hg.1	TC0700013297.hg.1	farbley.aAug10-unspliced	farbley	NA	121.95	7.39E-10	9.1312	5.72E-09	ctrl - P10; ctrl - P11; P2 - ctrl; ctrl - P3; ctrl - P8; P11 - P10; P2 - P10; P3 - P10; P10 - P8; P2 - P11; P3 - P11; P11 - P8; P2 - P3; P2 - P8; P3 - P8
TC0800008202.hg.1	TC0800008202.hg.1	ENST00000467010	CTD-2114J12.1	NA	121.5	7.55E-10	9.1219	5.83E-09	P10 - ctrl; ctrl - P11; P2 - ctrl; P3 - ctrl; P8 - ctrl; P10 - P11; P10 - P2; P10 - P3; P8 - P10; P2 - P11; P3 - P11; P8 - P11; P3 - P2; P8 - P2; P8 - P3
TC0500012842.hg.1	TC0500012842.hg.1	NM_004417	DUSP1	1843	121.4	7.59E-10	9.1197	5.84E-09	P10 - ctrl; ctrl - P2; P3 - ctrl; P8 - ctrl; P10 - P11; P10 - P2; P10 - P3; P11 - P2; P3 - P11; P8 - P11; P3 - P2; P8 - P2; P8 - P3
TC0200010545.hg.1	TC0200010545.hg.1	NM_003872	NRP2	8828	121.33	7.62E-10	9.1182	5.84E-09	P10 - ctrl; P11 - ctrl; ctrl - P2; P3 - ctrl; P8 - ctrl; P10 - P2; P3 - P10; P8 - P10; P11 - P2; P3 - P11; P8 - P11; P3 - P2; P8 - P2; P3 - P8

TC1000006754.hg.1	TC1000006754.hg.1	NM_001025076	CELF2	10659	121.16	7.68E-10	9.1146	5.86E-09	P10 - ctrl; ctrl - P11; P3 - ctrl; P8 - ctrl; P10 - P11; P10 - P2; P10 - P3; P8 - P10; P2 - P11; P3 - P11; P8 - P11; P3 - P2; P8 - P2; P8 - P3
TC0800007114.hg.1	TC0800007114.hg.1	NM_016240	SCARA3	51435	121.13	7.69E-10	9.1141	5.86E-09	P10 - ctrl; ctrl - P2; ctrl - P3; P8 - ctrl; P10 - P11; P10 - P2; P10 - P3; P8 - P11; P8 - P2; P8 - P3
TC1000007748.hg.1	TC1000007748.hg.1	NM_001170406	CDK1	983	120.65	7.87E-10	9.1041	5.98E-09	P10 - ctrl; ctrl - P11; ctrl - P2; P8 - ctrl; P10 - P11; P10 - P2; P10 - P3; P8 - P10; P11 - P2; P3 - P11; P8 - P11; P3 - P2; P8 - P2; P8 - P3
TC0800012069.hg.1	TC0800012069.hg.1	jozuby.aAug10-unspliced	jozuby	NA	120.19	8.05E-10	9.0943	6.09E-09	ctrl - P10; ctrl - P11; ctrl - P2; ctrl - P3; ctrl - P8; P11 - P10; P2 - P10; P10 - P3; P10 - P8; P2 - P11; P11 - P3; P11 - P8; P2 - P3; P2 - P8
TC0400007169.hg.1	TC0400007169.hg.1	NM_001173523	PCDH7	5099	119.77	8.22E-10	9.0853	6.20E-09	P10 - ctrl; P11 - ctrl; ctrl - P2; P3 - ctrl; P8 - ctrl; P10 - P11; P10 - P2; P10 - P3; P8 - P10; P11 - P2; P3 - P11; P8 - P11; P3 - P2; P8 - P2; P8 - P3
TC0100017532.hg.1	TC0100017532.hg.1	NA	NA	NA	119.59	8.29E-10	9.0815	6.24E-09	ctrl - P10; P11 - ctrl; ctrl - P2; ctrl - P3; ctrl - P8; P11 - P10; P10 - P3; P11 - P2; P11 - P3; P11 - P8; P2 - P3; P8 - P3
TC0X00007190.hg.1	TC0X00007190.hg.1	NM_001282167	PORCN	64840	118.99	8.54E-10	9.0687	6.39E-09	P10 - ctrl; P11 - ctrl; ctrl - P2; P3 - ctrl; P8 - ctrl; P10 - P11; P10 - P2; P10 - P3; P8 - P10; P11 - P2; P8 - P11; P3 - P2; P8 - P2; P8 - P3
TC1500007695.hg.1	TC1500007695.hg.1	NM_001104554	PAQR5	54852	118.91	8.57E-10	9.0671	6.39E-09	ctrl - P10; P11 - ctrl; ctrl - P2; ctrl - P8; P11 - P10; P2 - P10; P3 - P10; P8 - P10; P11 - P2; P11 - P3; P11 - P8; P3 - P2; P2 - P8; P3 - P8
TSUnmapped00000003.hg.1	TSUnmapped00000003.hg.1	ENST00000625425	SLC2A6	NA	118.9	8.57E-10	9.0668	6.39E-09	P10 - ctrl; ctrl - P11; ctrl - P2; P3 - ctrl; P8 - ctrl; P10 - P11; P10 - P2; P8 - P10; P3 - P11; P8 - P11; P3 - P2; P8 - P2; P8 - P3
TC0500013318.hg.1	TC0500013318.hg.1	NR_027386_5	GUSBP3		118.87	8.59E-10	9.0663	6.39E-09	ctrl - P10; ctrl - P11; P2 - ctrl; ctrl - P3; ctrl - P8; P11 - P10; P2 - P10; P3 - P10; P10 - P8; P2 - P11; P11 - P3; P11 - P8; P2 - P3; P2 - P8; P3 - P8
TC0200015623.hg.1	TC0200015623.hg.1	gleygobu.aAug10-unspliced	gleygobu	NA	118.61	8.70E-10	9.0606	6.45E-09	ctrl - P10; P2 - ctrl; ctrl - P3; ctrl - P8; P11 - P10; P2 - P10; P10 - P3; P10 - P8; P2 - P11; P11 - P3; P11 - P8; P2 - P3; P2 - P8; P3 - P8
TC0500009424.hg.1	TC0500009424.hg.1	NM_003862	FGF18	8817	118.52	8.74E-10	9.0586	6.46E-09	P10 - ctrl; P11 - ctrl; ctrl - P2; P3 - ctrl; P8 - ctrl; P10 - P11; P10 - P2; P3 - P10; P8 - P10; P11 - P2; P3 - P11; P8 - P11; P3 - P2; P8 - P2
TC0300013075.hg.1	TC0300013075.hg.1	ENST00000469794.1	RP11-3K16.2		118.46	8.76E-10	9.0574	6.46E-09	ctrl - P10; ctrl - P11; P2 - ctrl; ctrl - P3; ctrl - P8; P11 - P10; P2 - P10; P10 - P8; P2 - P11; P11 - P3; P11 - P8; P2 - P3; P2 - P8; P3 - P8
TC1600007931.hg.1	TC1600007931.hg.1	NM_017839	LPCAT2	54947	118.34	8.81E-10	9.0549	6.47E-09	ctrl - P10; ctrl - P11; ctrl - P2; ctrl - P3; ctrl - P8; P11 - P10; P2 - P10; P3 - P10; P8 - P10; P2 - P11; P3 - P11; P11 - P8; P2 - P8; P3 - P8
TC0700007067.hg.1	TC0700007067.hg.1	NM_152793	MTURN	222166	118.3	8.83E-10	9.054	6.47E-09	ctrl - P10; ctrl - P2; ctrl - P3; ctrl - P8; P11 - P10; P2 - P10; P11 - P2; P11 - P3; P11 - P8; P2 - P3; P2 - P8
TC2200007312.hg.1	TC2200007312.hg.1	NM_002305	LGALS1	3956	118.08	8.93E-10	9.0491	6.53E-09	P10 - ctrl; ctrl - P11; P3 - ctrl; P8 - ctrl; P10 - P11; P10 - P2; P3 - P10; P8 - P10; P2 - P11; P3 - P11; P8 - P11; P3 - P2; P8 - P2; P3 - P8
TC1300008945.hg.1	TC1300008945.hg.1	gachy.aAug10-unspliced	gachy	NA	117.8	9.05E-10	9.0432	6.60E-09	ctrl - P10; ctrl - P11; P2 - ctrl; ctrl - P3; ctrl - P8; P11 - P10; P2 - P10; P3 - P10; P10 - P8; P2 - P11; P11 - P8; P2 - P3; P2 - P8; P3 - P8
TC0900007811.hg.1	TC0900007811.hg.1	steylaw.aAug10-unspliced	steylaw	NA	117.44	9.22E-10	9.0353	6.70E-09	ctrl - P10; ctrl - P11; P2 - ctrl; ctrl - P3; ctrl - P8; P2 - P10; P3 - P10; P10 - P8; P2 - P11; P3 - P11; P11 - P8; P2 - P3; P2 - P8; P3 - P8
TC1300009736.hg.1	TC1300009736.hg.1	NR_046848	FAM155A-IT1	1.01E+08	117.28	9.29E-10	9.0318	6.73E-09	ctrl - P10; ctrl - P11; P2 - ctrl; ctrl - P8; P11 - P10; P2 - P10; P3 - P10; P10 - P8; P2 - P11; P3 - P11; P11 - P8; P2 - P3; P2 - P8; P3 - P8
TC0900008930.hg.1	TC0900008930.hg.1	NM_016307	PRRX2	51450	117.06	9.40E-10	9.0271	6.79E-09	P10 - ctrl; P11 - ctrl; P2 - ctrl; P3 - ctrl; P8 - ctrl; P10 - P11; P10 - P2; P10 - P3; P11 - P2; P3 - P11; P8 - P11; P3 - P2; P8 - P2; P8 - P3
TC1500007664.hg.1	TC1500007664.hg.1	slerzo.aAug10-unspliced	slerzo	NA	116.75	9.54E-10	9.0205	6.87E-09	ctrl - P10; ctrl - P11; P2 - ctrl; ctrl - P3; ctrl - P8; P10 - P11; P2 - P10; P3 - P10; P10 - P8; P2 - P11; P3 - P11; P2 - P3; P2 - P8; P3 - P8
TC0400011920.hg.1	TC0400011920.hg.1	NM_014331	SLC7A11	23657	116.15	9.83E-10	9.0072	7.06E-09	P10 - ctrl; P11 - ctrl; P2 - ctrl; P3 - ctrl; P8 - ctrl; P10 - P11; P10 - P2; P11 - P2; P3 - P11; P8 - P11; P3 - P2; P8 - P2
TC0200014617.hg.1	TC0200014617.hg.1	NM_001164507	NEB	4703	115.98	9.92E-10	9.0036	7.10E-09	ctrl - P10; ctrl - P3; ctrl - P8; P11 - P10; P2 - P10; P3 - P10; P2 - P11; P11 - P3; P11 - P8; P2 - P3; P2 - P8; P3 - P8
TC1200011067.hg.1	TC1200011067.hg.1	NM_173812	DPY19L2	283417	115.48	1.02E-09	8.9926	7.26E-09	ctrl - P10; ctrl - P11; ctrl - P3; ctrl - P8; P11 - P10; P2 - P10; P3 - P10; P8 - P10; P2 - P11; P11 - P8; P2 - P3; P2 - P8; P3 - P8
TC0600012745.hg.1	TC0600012745.hg.1	tugleyby.aAug10-unspliced	tugleyby	NA	115.31	1.03E-09	8.9888	7.31E-09	ctrl - P10; ctrl - P11; P2 - ctrl; ctrl - P3; ctrl - P8; P2 - P10; P3 - P10; P10 - P8; P2 - P11; P11 - P8; P2 - P3; P2 - P8; P3 - P8
TC1000012117.hg.1	TC1000012117.hg.1	NM_001270764	CHST15	51363	115.06	1.04E-09	8.9833	7.38E-09	P10 - ctrl; ctrl - P11; P3 - ctrl; P8 - ctrl; P10 - P11; P10 - P2; P10 - P3; P2 - P11; P3 - P11; P8 - P11; P3 - P2; P8 - P2; P8 - P3
TC0800012147.hg.1	TC0800012147.hg.1	NM_003313	TSTA3	7264	114.88	1.05E-09	8.9794	7.42E-09	P10 - ctrl; P2 - ctrl; P3 - ctrl; P8 - ctrl; P10 - P11; P10 - P2; P3 - P10; P8 - P10; P2 - P11; P3 - P11; P8 - P11; P3 - P2; P8 - P2
TSUnmapped00000051.hg.1	TSUnmapped00000051.hg.1	ENST00000625489	SLC2A6	NA	114.4	1.07E-09	8.9687	7.59E-09	P10 - ctrl; ctrl - P11; ctrl - P2; P3 - ctrl; P8 - ctrl; P10 - P11; P10 - P2; P10 - P3; P8 - P10; P3 - P11; P8 - P11; P3 - P2; P8 - P2; P8 - P3
TC1100006732.hg.1	TC1100006732.hg.1	sugyby.aAug10	sugyby	NA	114.26	1.08E-09	8.9657	7.60E-09	P10 - ctrl; P11 - ctrl; ctrl - P2; P3 - ctrl; P8 - ctrl; P10 - P2; P10 - P3; P10 - P8; P11 - P2; P11 - P3; P11 - P8; P3 - P2; P8 - P2; P8 - P3
TC0500013317.hg.1	TC0500013317.hg.1	NR_024054	SMA4	11039	114.24	1.08E-09	8.9651	7.60E-09	ctrl - P10; ctrl - P11; P2 - ctrl; ctrl - P3; ctrl - P8; P11 - P10; P2 - P10; P3 - P10; P2 - P11; P11 - P8; P2 - P3; P2 - P8; P3 - P8
TC1200008224.hg.1	TC1200008224.hg.1	NM_006851	GLIPR1	11010	114.21	1.09E-09	8.9645	7.60E-09	P10 - ctrl; ctrl - P2; P3 - ctrl; P8 - ctrl; P10 - P11; P10 - P2; P3 - P10; P8 - P10; P3 - P11; P8 - P11; P3 - P2; P8 - P2; P8 - P3
TC1300006543.hg.1	TC1300006543.hg.1	NM_002010	FGF9	2254	114.14	1.09E-09	8.9629	7.60E-09	ctrl - P10; ctrl - P3; ctrl - P8; P11 - P10; P2 - P10; P2 - P11; P11 - P3; P11 - P8; P2 - P3; P2 - P8; P3 - P8
TC0200016019.hg.1	TC0200016019.hg.1	NR_002921	SNORA75	2q37.1	113.89	1.10E-09	8.9575	7.68E-09	ctrl - P10; P11 - ctrl; P2 - ctrl; ctrl - P8; P11 - P10; P2 - P10; P3 - P10; P2 - P11; P11 - P3; P11 - P8; P2 - P3; P2 - P8; P3 - P8

TC0800011595.hg.1	TC0800011595.hg.1	hsa_circ_0002817	EXT1		113.33	1.14E-09	8.9449	7.88E-09	ctrl - P10; ctrl - P11; P2 - ctrl; ctrl - P3; ctrl - P8; P10 - P11; P2 - P10; P3 - P10; P10 - P8; P2 - P11; P3 - P11; P11 - P8; P2 - P3; P2 - P8; P3 - P8
TC1100011844.hg.1	TC1100011844.hg.1	fomorby.aAug10-unspliced	fomorby	NA	113.19	1.14E-09	8.9417	7.91E-09	ctrl - P10; ctrl - P11; P2 - ctrl; ctrl - P3; ctrl - P8; P11 - P10; P2 - P10; P3 - P10; P10 - P8; P2 - P11; P11 - P8; P2 - P3; P2 - P8; P3 - P8
TC0500013319.hg.1	TC0500013319.hg.1	NR_033968	GUSBP9	1E+08	113.15	1.15E-09	8.9409	7.91E-09	ctrl - P10; ctrl - P11; P2 - ctrl; ctrl - P3; ctrl - P8; P11 - P10; P2 - P10; P3 - P10; P10 - P8; P2 - P11; P11 - P8; P2 - P3; P2 - P8; P3 - P8
TC1600008712.hg.1	TC1600008712.hg.1	NM_002163	IRF8	3394	112.81	1.17E-09	8.9331	8.00E-09	P10 - ctrl; P3 - ctrl; P8 - ctrl; P10 - P11; P10 - P2; P10 - P3; P3 - P11; P8 - P11; P8 - P2; P8 - P3
TC1300009737.hg.1	TC1300009737.hg.1	roynoy.aAug10-unspliced	roynoy	NA	112.79	1.17E-09	8.9327	8.00E-09	ctrl - P10; ctrl - P11; P2 - ctrl; ctrl - P3; ctrl - P8; P2 - P10; P3 - P10; P10 - P8; P2 - P11; P3 - P11; P11 - P8; P2 - P3; P2 - P8; P3 - P8
TC1300008096.hg.1	TC1300008096.hg.1	NA	NA	NA	112.77	1.17E-09	8.9323	8.00E-09	ctrl - P10; ctrl - P11; ctrl - P3; ctrl - P8; P2 - P10; P10 - P3; P10 - P8; P2 - P11; P11 - P3; P11 - P8; P2 - P3; P2 - P8; P3 - P8
TC2000006688.hg.1	TC2000006688.hg.1	NM_003081	SNAP25	6616	112.49	1.19E-09	8.926	8.10E-09	P10 - ctrl; P11 - ctrl; P3 - ctrl; P8 - ctrl; P10 - P11; P10 - P2; P10 - P3; P11 - P2; P3 - P11; P8 - P11; P3 - P2; P8 - P2; P8 - P3
TC0200009762.hg.1	TC0200009762.hg.1	PKP4.vkAug10-unspliced	PKP4	NA	111.98	1.22E-09	8.9144	8.27E-09	ctrl - P10; P2 - ctrl; ctrl - P3; ctrl - P8; P11 - P10; P2 - P10; P3 - P10; P10 - P8; P2 - P11; P11 - P3; P11 - P8; P2 - P3; P2 - P8; P3 - P8
TC0600012743.hg.1	TC0600012743.hg.1	NR_033557	LOC100422737	1E+08	111.97	1.22E-09	8.9142	8.27E-09	ctrl - P10; ctrl - P11; ctrl - P2; ctrl - P3; ctrl - P8; P11 - P10; P2 - P10; P10 - P8; P2 - P11; P11 - P3; P11 - P8; P2 - P3; P2 - P8; P3 - P8
TC1000010845.hg.1	TC1000010845.hg.1	jalor.aAug10-unspliced	jalor	NA	111.81	1.23E-09	8.9105	8.27E-09	ctrl - P10; ctrl - P11; P2 - ctrl; ctrl - P3; ctrl - P8; P2 - P10; P10 - P8; P2 - P11; P11 - P8; P2 - P3; P2 - P8; P3 - P8
TC1100013087.hg.1	TC1100013087.hg.1	NM_001165	BIRC3	330	111.73	1.23E-09	8.9089	8.27E-09	P10 - ctrl; P11 - ctrl; P3 - ctrl; P8 - ctrl; P10 - P11; P10 - P2; P8 - P10; P3 - P11; P8 - P11; P3 - P2; P8 - P2; P8 - P3
TC2100006465.hg.1	TC2100006465.hg.1	uc032psp.2	FP671120.2	NA	111.65	1.24E-09	8.907	8.27E-09	ctrl - P10; ctrl - P11; P2 - ctrl; ctrl - P3; ctrl - P8; P2 - P10; P10 - P8; P2 - P11; P2 - P3; P2 - P8; P3 - P8
TC2100006481.hg.1	TC2100006481.hg.1	uc032ptl.2	FP236383.8	NA	111.65	1.24E-09	8.907	8.27E-09	ctrl - P10; ctrl - P11; P2 - ctrl; ctrl - P3; ctrl - P8; P2 - P10; P10 - P8; P2 - P11; P2 - P3; P2 - P8; P3 - P8
TC22_KI270733v1_random00006438.hg.1	TC22_KI270733v1_random00006438.hg.1	uc032qtv.2	AL353644.9	NA	111.65	1.24E-09	8.907	8.27E-09	ctrl - P10; ctrl - P11; P2 - ctrl; ctrl - P3; ctrl - P8; P2 - P10; P10 - P8; P2 - P11; P2 - P3; P2 - P8; P3 - P8
TCUn_GL000220v1 00006438.hg.1	TCUn_GL000220v1 00006438.hg.1	uc022brg.2	AL592188.2	NA	111.65	1.24E-09	8.907	8.27E-09	ctrl - P10; ctrl - P11; P2 - ctrl; ctrl - P3; ctrl - P8; P2 - P10; P10 - P8; P2 - P11; P2 - P3; P2 - P8; P3 - P8
TC0200009707.hg.1	TC0200009707.hg.1	NM_001260508	KCNJ3	3760	111.63	1.24E-09	8.9065	8.27E-09	ctrl - P10; P11 - ctrl; P2 - ctrl; ctrl - P3; ctrl - P8; P11 - P10; P2 - P10; P11 - P2; P11 - P3; P11 - P8; P2 - P3; P2 - P8
TC0300009777.hg.1	TC0300009777.hg.1	NM_000893	KNG1	3827	110.97	1.28E-09	8.8914	8.53E-09	ctrl - P10; ctrl - P11; ctrl - P2; ctrl - P3; ctrl - P8; P11 - P10; P10 - P2; P10 - P3; P10 - P8; P11 - P2; P11 - P3; P11 - P8; P8 - P2; P8 - P3
TC0300008196.hg.1	TC0300008196.hg.1	NM_175056	ZPLD1	131368	110.64	1.31E-09	8.8838	8.65E-09	P10 - ctrl; P2 - ctrl; P3 - ctrl; P8 - ctrl; P10 - P11; P10 - P2; P10 - P3; P2 - P11; P3 - P11; P8 - P11; P3 - P2; P8 - P2
HTA2-pos-2985914_st	23150250	NA	NA	NA	110.62	1.31E-09	8.8834	8.65E-09	ctrl - P10; ctrl - P11; ctrl - P3; ctrl - P8; P11 - P10; P2 - P10; P3 - P10; P2 - P11; P11 - P8; P2 - P3; P2 - P8; P3 - P8
TC1200009547.hg.1	TC1200009547.hg.1	NM_001190997	SLC6A13	6540	110.49	1.32E-09	8.8803	8.67E-09	ctrl - P10; P11 - ctrl; ctrl - P2; ctrl - P3; ctrl - P8; P11 - P10; P3 - P10; P10 - P8; P11 - P2; P11 - P3; P11 - P8; P2 - P8; P3 - P8
TC1400009175.hg.1	TC1400009175.hg.1	NM_007361	NID2	22795	110.45	1.32E-09	8.8795	8.67E-09	P10 - ctrl; P2 - ctrl; P3 - ctrl; P8 - ctrl; P10 - P11; P10 - P2; P10 - P3; P2 - P11; P3 - P11; P8 - P11; P3 - P2; P8 - P2; P8 - P3
TC0400011284.hg.1	TC0400011284.hg.1	NA	NA	NA	110.43	1.32E-09	8.879	8.67E-09	ctrl - P10; ctrl - P11; ctrl - P3; ctrl - P8; P11 - P10; P2 - P10; P3 - P10; P10 - P8; P2 - P11; P11 - P3; P11 - P8; P2 - P3; P2 - P8; P3 - P8
TC0100018482.hg.1	TC0100018482.hg.1	NM_001040874	HIST2H2AA4	723790	110.1	1.34E-09	8.8713	8.80E-09	P10 - ctrl; P11 - ctrl; ctrl - P2; P3 - ctrl; P8 - ctrl; P10 - P11; P10 - P2; P10 - P3; P11 - P2; P8 - P11; P3 - P2; P8 - P2; P8 - P3
TC1500009709.hg.1	TC1500009709.hg.1	NM_001218	CA12	771	109.97	1.35E-09	8.8685	8.83E-09	P10 - ctrl; P11 - ctrl; P3 - ctrl; P8 - ctrl; P10 - P11; P10 - P2; P3 - P11; P8 - P11; P3 - P2; P8 - P2; P8 - P3
TC1000008767.hg.1	TC1000008767.hg.1	NM_032727	INA	9118	109.88	1.36E-09	8.8664	8.85E-09	P10 - ctrl; ctrl - P11; P2 - ctrl; P3 - ctrl; P8 - ctrl; P10 - P11; P10 - P2; P8 - P10; P2 - P11; P3 - P11; P8 - P11; P3 - P2; P8 - P2; P8 - P3
TC0200015093.hg.1	TC0200015093.hg.1	NM_001113397	ZNF385B	151126	109.82	1.36E-09	8.865	8.86E-09	ctrl - P10; P11 - ctrl; ctrl - P2; ctrl - P8; P11 - P10; P3 - P10; P8 - P10; P11 - P2; P11 - P3; P11 - P8; P3 - P2; P3 - P8
TC1000007954.hg.1	TC1000007954.hg.1	NM_001174098	SLC29A3	55315	109.78	1.37E-09	8.864	8.86E-09	ctrl - P10; P11 - ctrl; ctrl - P2; ctrl - P3; ctrl - P8; P11 - P10; P10 - P2; P10 - P3; P11 - P2; P11 - P3; P11 - P8; P3 - P2; P8 - P2; P8 - P3
TC1800008892.hg.1	TC1800008892.hg.1	snysky.aAug10-unspliced	snysky	NA	109.71	1.37E-09	8.8623	8.87E-09	ctrl - P10; ctrl - P11; P2 - ctrl; ctrl - P3; ctrl - P8; P11 - P10; P2 - P10; P3 - P10; P2 - P11; P11 - P8; P2 - P3; P2 - P8; P3 - P8
TC0200008666.hg.1	TC0200008666.hg.1	NM_000877	IL1R1	3554	109.44	1.39E-09	8.8562	8.97E-09	P10 - ctrl; P11 - ctrl; P3 - ctrl; P8 - ctrl; P10 - P11; P10 - P2; P10 - P3; P11 - P2; P8 - P11; P3 - P2; P8 - P2; P8 - P3
TC0100018210.hg.1	TC0100018210.hg.1	NM_001162530	SH3D21	79729	109.22	1.41E-09	8.8511	9.05E-09	ctrl - P10; ctrl - P11; ctrl - P2; ctrl - P3; ctrl - P8; P11 - P10; P2 - P10; P3 - P10; P8 - P10; P11 - P2; P11 - P3; P11 - P8; P3 - P2; P3 - P8
TC1900010856.hg.1	TC1900010856.hg.1	NM_001005376	PLAUR	5329	108.87	1.44E-09	8.8429	9.20E-09	P10 - ctrl; ctrl - P2; P3 - ctrl; P8 - ctrl; P10 - P11; P10 - P2; P8 - P10; P11 - P2; P3 - P11; P8 - P11; P3 - P2; P8 - P2; P8 - P3
TC0500011066.hg.1	TC0500011066.hg.1	NA	NA	NA	108.65	1.45E-09	8.8378	9.29E-09	ctrl - P10; ctrl - P11; P2 - ctrl; ctrl - P3; ctrl - P8; P11 - P10; P2 - P10; P3 - P10; P10 - P8; P2 - P11; P11 - P8; P2 - P3; P2 - P8; P3 - P8
TC1800008083.hg.1	TC1800008083.hg.1	NA	NA	NA	108.34	1.48E-09	8.8306	9.42E-09	ctrl - P10; ctrl - P11; P2 - ctrl; ctrl - P3; ctrl - P8; P11 - P10; P2 - P10; P3 - P10; P10 - P8; P2 - P11; P11 - P8; P2 - P3; P2 - P8; P3 - P8

TC1000010843.hg.1	TC1000010843.hg.1	NM_001033083	PBLD	64081	108.23	1.49E-09	8.828	9.42E-09	ctrl - P10; P11 - ctrl; ctrl - P2; ctrl - P3; ctrl - P8; P11 - P10; P10 - P3; P11 - P2; P11 - P3; P11 - P8; P2 - P3; P8 - P3
TC0300010930.hg.1	TC0300010930.hg.1	NM_020208	SLC6A20	54716	108.2	1.49E-09	8.8273	9.42E-09	P10 - ctrl; P11 - ctrl; P2 - ctrl; P3 - ctrl; P8 - ctrl; P10 - P11; P10 - P2; P10 - P3; P8 - P11; P8 - P2; P8 - P3
TC0300010350.hg.1	TC0300010350.hg.1	NM_004625	WNT7A	7476	108.19	1.49E-09	8.8269	9.42E-09	P10 - ctrl; ctrl - P11; ctrl - P2; P3 - ctrl; P8 - ctrl; P10 - P11; P10 - P2; P3 - P11; P8 - P11; P3 - P2; P8 - P2; P8 - P3
TC0X00010954.hg.1	TC0X00010954.hg.1	NM_001139498	FGF13	2258	107.94	1.51E-09	8.8212	9.53E-09	ctrl - P10; ctrl - P2; ctrl - P3; ctrl - P8; P11 - P10; P2 - P10; P3 - P10; P11 - P2; P11 - P3; P11 - P8; P2 - P3; P2 - P8; P3 - P8
TC1000008236.hg.1	TC1000008236.hg.1	NM_001128309	TSPAN14	81619	107.77	1.52E-09	8.8172	9.59E-09	P10 - ctrl; ctrl - P11; ctrl - P2; P8 - ctrl; P10 - P11; P10 - P2; P10 - P3; P11 - P2; P3 - P11; P8 - P11; P3 - P2; P8 - P2; P8 - P3
TC0300013072.hg.1	TC0300013072.hg.1	shorjeebu.aAug10-unspliced	shorjeebu	NA	107.7	1.53E-09	8.8156	9.60E-09	ctrl - P10; ctrl - P11; P2 - ctrl; ctrl - P3; ctrl - P8; P11 - P10; P2 - P10; P10 - P8; P2 - P11; P11 - P3; P11 - P8; P2 - P3; P2 - P8; P3 - P8
TC0100017113.hg.1	TC0100017113.hg.1	NM_002644	PIGR	5284	107.44	1.55E-09	8.8095	9.71E-09	P10 - ctrl; P8 - ctrl; P10 - P11; P10 - P2; P10 - P3; P8 - P10; P8 - P11; P8 - P2; P8 - P3
TC0700011626.hg.1	TC0700011626.hg.1	NM_006379	SEMA3C	10512	105.82	1.69E-09	8.7709	1.06E-08	ctrl - P2; P3 - ctrl; P8 - ctrl; P10 - P11; P10 - P2; P3 - P10; P8 - P10; P11 - P2; P3 - P11; P8 - P11; P3 - P2; P8 - P2; P3 - P8
TC0600011499.hg.1	TC0600011499.hg.1	NM_001243961	HLA-DQB1	3119	105.73	1.70E-09	8.7686	1.06E-08	P10 - ctrl; ctrl - P2; P8 - ctrl; P10 - P11; P10 - P2; P10 - P3; P11 - P2; P8 - P11; P3 - P2; P8 - P2; P8 - P3
TC0400009129.hg.1	TC0400009129.hg.1	NM_001253727	RXFP1	4q32.1	105.68	1.71E-09	8.7676	1.06E-08	P10 - ctrl; P2 - ctrl; P3 - ctrl; P8 - ctrl; P10 - P11; P10 - P2; P10 - P3; P2 - P11; P3 - P11; P8 - P11; P3 - P2; P8 - P2; P8 - P3
TC0300007061.hg.1	TC0300007061.hg.1	ployswerby.aAug10-unspliced	ployswerby	NA	105.67	1.71E-09	8.7672	1.06E-08	ctrl - P10; ctrl - P11; ctrl - P3; ctrl - P8; P11 - P10; P2 - P10; P3 - P10; P10 - P8; P2 - P11; P11 - P3; P11 - P8; P2 - P3; P2 - P8; P3 - P8
TC1000009851.hg.1	TC1000009851.hg.1	NM_018027	FRMD4A	55691	105.48	1.73E-09	8.7628	1.07E-08	ctrl - P10; P11 - ctrl; ctrl - P2; ctrl - P3; ctrl - P8; P11 - P10; P10 - P3; P10 - P8; P11 - P2; P11 - P3; P11 - P8; P2 - P3; P2 - P8
TC0X00007537.hg.1	TC0X00007537.hg.1	NM_012310	KIF4A	24137	105.35	1.74E-09	8.7595	1.07E-08	P10 - ctrl; ctrl - P11; ctrl - P2; ctrl - P3; P8 - ctrl; P10 - P11; P10 - P2; P10 - P3; P8 - P10; P8 - P11; P3 - P2; P8 - P2; P8 - P3
TC0300007189.hg.1	TC0300007189.hg.1	NM_001100594	SNRK	54861	105.28	1.75E-09	8.7579	1.07E-08	ctrl - P10; P11 - ctrl; ctrl - P2; ctrl - P3; ctrl - P8; P11 - P10; P11 - P2; P11 - P3; P11 - P8; P2 - P3; P2 - P8
TC0300009852.hg.1	TC0300009852.hg.1	NA	NA	NA	104.68	1.81E-09	8.7434	1.11E-08	P10 - ctrl; P3 - ctrl; P8 - ctrl; P10 - P11; P10 - P2; P10 - P3; P3 - P11; P8 - P11; P3 - P2; P8 - P2; P8 - P3
TC0500011352.hg.1	TC0500011352.hg.1	NM_001278642	EDIL3	10085	103.63	1.91E-09	8.7179	1.17E-08	ctrl - P10; P2 - ctrl; ctrl - P8; P11 - P10; P2 - P10; P3 - P10; P10 - P8; P2 - P11; P11 - P8; P2 - P3; P2 - P8; P3 - P8
TC0800011416.hg.1	TC0800011416.hg.1	rawwybu.aAug10-unspliced	rawwybu	NA	103.29	1.95E-09	8.7094	1.19E-08	ctrl - P10; ctrl - P11; P2 - ctrl; ctrl - P3; ctrl - P8; P11 - P10; P2 - P10; P10 - P8; P2 - P11; P11 - P8; P2 - P3; P2 - P8; P3 - P8
TC2000007297.hg.1	TC2000007297.hg.1	NM_080607	VSTM2L	128434	103.26	1.96E-09	8.7088	1.19E-08	P10 - ctrl; P11 - ctrl; P3 - ctrl; P8 - ctrl; P10 - P11; P10 - P2; P3 - P10; P8 - P10; P11 - P2; P3 - P11; P8 - P11; P3 - P2; P8 - P2; P8 - P3
TC1000012516.hg.1	TC1000012516.hg.1	NM_001135241	AKR1C2	1646	102.94	1.99E-09	8.7009	1.21E-08	P10 - ctrl; P11 - ctrl; ctrl - P2; P3 - ctrl; P8 - ctrl; P10 - P11; P10 - P2; P10 - P3; P11 - P2; P3 - P11; P8 - P11; P3 - P2; P8 - P2; P8 - P3
TC1200009621.hg.1	TC1200009621.hg.1	NM_001243088	FOXM1	2305	102.72	2.02E-09	8.6955	1.22E-08	P10 - ctrl; ctrl - P11; ctrl - P2; P3 - ctrl; P8 - ctrl; P10 - P11; P10 - P2; P10 - P3; P8 - P10; P3 - P11; P8 - P11; P3 - P2; P8 - P2; P8 - P3
TC0200014361.hg.1	TC0200014361.hg.1	NM_001077427	LYPD1	2q21.2	102.62	2.03E-09	8.693	1.22E-08	P10 - ctrl; P11 - ctrl; P3 - ctrl; P8 - ctrl; P10 - P11; P10 - P2; P10 - P3; P10 - P8; P11 - P2; P3 - P11; P8 - P11; P3 - P2; P8 - P2
TC0700012226.hg.1	TC0700012226.hg.1	NA	NA	NA	102.61	2.03E-09	8.6928	1.22E-08	ctrl - P10; ctrl - P11; P2 - ctrl; ctrl - P3; ctrl - P8; P11 - P10; P2 - P10; P3 - P10; P2 - P11; P11 - P8; P2 - P3; P2 - P8; P3 - P8
TC0800008378.hg.1	TC0800008378.hg.1	ENST00000519566.1	KB-1991G8.1		102.55	2.04E-09	8.6914	1.23E-08	P10 - ctrl; P2 - ctrl; P3 - ctrl; P8 - ctrl; P10 - P11; P10 - P2; P10 - P3; P2 - P11; P3 - P11; P8 - P11; P8 - P2; P8 - P3
TC1700007790.hg.1	TC1700007790.hg.1	NM_001254	CDC6	990	102.35	2.06E-09	8.6864	1.24E-08	P10 - ctrl; ctrl - P11; P3 - ctrl; P8 - ctrl; P10 - P11; P10 - P2; P3 - P10; P8 - P10; P2 - P11; P3 - P11; P8 - P11; P3 - P2; P8 - P2
TC1500009738.hg.1	TC1500009738.hg.1	NM_001029989	KIAA0101	9768	102.21	2.07E-09	8.683	1.24E-08	P10 - ctrl; ctrl - P11; ctrl - P2; P3 - ctrl; P8 - ctrl; P10 - P11; P10 - P2; P10 - P3; P8 - P10; P3 - P11; P8 - P11; P3 - P2; P8 - P2; P8 - P3
TC0100013561.hg.1	TC0100013561.hg.1	NM_004102	FABP3	2170	102.08	2.09E-09	8.6798	1.25E-08	ctrl - P10; P11 - ctrl; ctrl - P2; ctrl - P3; ctrl - P8; P11 - P10; P10 - P3; P10 - P8; P11 - P2; P11 - P3; P11 - P8; P2 - P8
TC1000008731.hg.1	TC1000008731.hg.1	NR_038937	RPARP-AS1	1.01E+08	102.01	2.10E-09	8.6779	1.25E-08	ctrl - P10; P11 - ctrl; ctrl - P2; ctrl - P3; ctrl - P8; P11 - P10; P2 - P10; P3 - P10; P11 - P2; P11 - P3; P11 - P8; P2 - P3; P2 - P8; P3 - P8
TC1700008247.hg.1	TC1700008247.hg.1	NM_001168215	TMEM92	162461	101.36	2.18E-09	8.6617	1.30E-08	P10 - ctrl; P3 - ctrl; P8 - ctrl; P10 - P11; P10 - P2; P8 - P10; P2 - P11; P3 - P11; P8 - P11; P3 - P2; P8 - P2; P8 - P3
TC1700012468.hg.1	TC1700012468.hg.1	NM_001002032	HN1	51155	101.24	2.19E-09	8.6586	1.30E-08	P10 - ctrl; ctrl - P11; ctrl - P2; P3 - ctrl; P8 - ctrl; P10 - P11; P10 - P2; P10 - P3; P3 - P11; P8 - P11; P3 - P2; P8 - P2; P8 - P3
TC1000011524.hg.1	TC1000011524.hg.1	NM_152309	PIK3AP1	118788	100.84	2.25E-09	8.6486	1.33E-08	P10 - ctrl; ctrl - P11; P2 - ctrl; P3 - ctrl; P8 - ctrl; P10 - P11; P10 - P2; P10 - P3; P2 - P11; P3 - P11; P8 - P11; P3 - P2; P8 - P2; P8 - P3
TC1400007950.hg.1	TC1400007950.hg.1	NM_006888	CALM1	801	100.82	2.25E-09	8.6483	1.33E-08	P10 - ctrl; ctrl - P11; P3 - ctrl; P8 - ctrl; P10 - P11; P10 - P2; P10 - P3; P2 - P11; P3 - P11; P8 - P11; P3 - P2; P8 - P2; P8 - P3
TC1100012788.hg.1	TC1100012788.hg.1	NA	NA	NA	100.77	2.26E-09	8.6468	1.33E-08	ctrl - P10; ctrl - P11; ctrl - P2; ctrl - P3; ctrl - P8; P11 - P10; P2 - P10; P11 - P2; P11 - P3; P11 - P8; P2 - P3; P2 - P8
TC0600007006.hg.1	TC0600007006.hg.1	NM_001165032	RNF182	221687	100.29	2.32E-09	8.6349	1.36E-08	ctrl - P10; ctrl - P11; ctrl - P2; ctrl - P8; P11 - P10; P2 - P10; P3 - P10; P8 - P10; P11 - P2; P3 - P11; P11 - P8; P3 - P2; P2 - P8; P3 - P8

TC1500008995.hg.1	TC1500008995.hg.1	NM_001023567	GOLGA8B	440270	100.16	2.34E-09	8.6316	1.37E-08	ctrl - P10; ctrl - P11; ctrl - P2; ctrl - P3; ctrl - P8; P11 - P10; P2 - P10; P3 - P10; P2 - P11; P11 - P8; P2 - P3; P2 - P8; P3 - P8
TC0500011062.hg.1	TC0500011062.hg.1	kleyplarby.aAug10-unspliced	kleyplarby	NA	99.78	2.39E-09	8.6219	1.40E-08	ctrl - P10; ctrl - P11; P2 - ctrl; ctrl - P3; ctrl - P8; P11 - P10; P2 - P10; P3 - P10; P10 - P8; P2 - P11; P11 - P8; P2 - P3; P2 - P8; P3 - P8
TC1100011834.hg.1	TC1100011834.hg.1	riyare.aAug10-unspliced	riyare	NA	99.555	2.42E-09	8.6162	1.41E-08	ctrl - P10; ctrl - P11; ctrl - P2; ctrl - P3; ctrl - P8; P11 - P10; P2 - P10; P3 - P10; P10 - P8; P2 - P11; P3 - P11; P11 - P8; P2 - P3; P2 - P8; P3 - P8
TC0700008053.hg.1	TC0700008053.hg.1	snarsharby.aAug10-unspliced	snarsharby	NA	99.491	2.43E-09	8.6146	1.41E-08	ctrl - P10; ctrl - P11; ctrl - P3; ctrl - P8; P11 - P10; P2 - P10; P3 - P10; P2 - P11; P3 - P11; P11 - P8; P2 - P3; P2 - P8; P3 - P8
TC0100017313.hg.1	TC0100017313.hg.1	peenubu.aAug10-unspliced	peenubu	NA	99.358	2.45E-09	8.6112	1.42E-08	ctrl - P10; ctrl - P2; ctrl - P3; ctrl - P8; P11 - P10; P2 - P10; P3 - P10; P10 - P8; P11 - P3; P11 - P8; P2 - P3; P2 - P8; P3 - P8
TC0800009600.hg.1	TC0800009600.hg.1	NM_053279	FAM167A	83648	98.977	2.50E-09	8.6015	1.45E-08	P10 - ctrl; ctrl - P2; ctrl - P3; P8 - ctrl; P10 - P11; P10 - P2; P10 - P3; P11 - P2; P11 - P3; P8 - P11; P3 - P2; P8 - P2; P8 - P3
TC0900010262.hg.1	TC0900010262.hg.1	gleytoyby.aAug10-unspliced	gleytoyby	NA	98.823	2.53E-09	8.5976	1.46E-08	ctrl - P11; P2 - ctrl; P3 - ctrl; ctrl - P8; P10 - P11; P2 - P10; P3 - P10; P10 - P8; P2 - P11; P3 - P11; P8 - P11; P2 - P3; P2 - P8; P3 - P8
TC0800010252.hg.1	TC0800010252.hg.1	NM_000037	ANK1	286	98.514	2.57E-09	8.5896	1.48E-08	P10 - ctrl; P11 - ctrl; P2 - ctrl; P3 - ctrl; P8 - ctrl; P10 - P2; P3 - P10; P8 - P10; P11 - P2; P3 - P11; P3 - P2; P8 - P2
TC1700012191.hg.1	TC1700012191.hg.1	NM_001040059	CD68	968	98.432	2.59E-09	8.5875	1.49E-08	P10 - ctrl; P11 - ctrl; ctrl - P2; P3 - ctrl; P8 - ctrl; P10 - P2; P3 - P10; P10 - P8; P11 - P2; P11 - P8; P3 - P2; P8 - P2; P3 - P8
TC1500007410.hg.1	TC1500007410.hg.1	morser.aAug10-unspliced	morser	NA	98.388	2.59E-09	8.5864	1.49E-08	P10 - ctrl; P11 - ctrl; P3 - ctrl; P8 - ctrl; P10 - P11; P10 - P2; P8 - P10; P11 - P2; P3 - P11; P8 - P11; P3 - P2; P8 - P2; P8 - P3
TC0800007674.hg.1	TC0800007674.hg.1	ENST00000520251	LOC105375844	1.05E+08	98.383	2.59E-09	8.5863	1.49E-08	ctrl - P11; P2 - ctrl; P3 - ctrl; ctrl - P8; P10 - P11; P2 - P10; P3 - P10; P10 - P8; P2 - P11; P3 - P11; P11 - P8; P2 - P3; P2 - P8; P3 - P8
TC0Y00007309.hg.1	TC0Y00007309.hg.1	blabo.aAug10-unspliced	blabo	NA	98.294	2.61E-09	8.584	1.49E-08	ctrl - P10; ctrl - P11; ctrl - P2; ctrl - P3; ctrl - P8; P11 - P10; P3 - P10; P8 - P10; P11 - P2; P3 - P11; P11 - P8; P3 - P2; P8 - P2; P3 - P8
TC0600008563.hg.1	TC0600008563.hg.1	uc063pqw.1	RNU6-1016P	NA	98.067	2.64E-09	8.5781	1.51E-08	ctrl - P10; ctrl - P11; P2 - ctrl; ctrl - P3; ctrl - P8; P11 - P10; P2 - P10; P3 - P10; P2 - P11; P3 - P11; P11 - P8; P2 - P3; P2 - P8; P3 - P8
TC1500006588.hg.1	TC1500006588.hg.1	NR_003303	SNORD115-11	15q11.2	97.858	2.67E-09	8.5727	1.51E-08	P10 - ctrl; P11 - ctrl; P2 - ctrl; P3 - ctrl; P8 - ctrl; P10 - P11; P10 - P2; P3 - P10; P3 - P11; P8 - P11; P3 - P2; P8 - P2; P3 - P8
TC1500006603.hg.1	TC1500006603.hg.1	ENST00000362834	SNORD115-29	15q11.2	97.858	2.67E-09	8.5727	1.51E-08	P10 - ctrl; P11 - ctrl; P2 - ctrl; P3 - ctrl; P8 - ctrl; P10 - P11; P10 - P2; P3 - P10; P3 - P11; P8 - P11; P3 - P2; P8 - P2; P3 - P8
TC1500006604.hg.1	TC1500006604.hg.1	ENST00000365629	SNORD115-36	15q11.2	97.858	2.67E-09	8.5727	1.51E-08	P10 - ctrl; P11 - ctrl; P2 - ctrl; P3 - ctrl; P8 - ctrl; P10 - P11; P10 - P2; P3 - P10; P3 - P11; P8 - P11; P3 - P2; P8 - P2; P3 - P8
TC1500006607.hg.1	TC1500006607.hg.1	ENST00000365503	SNORD115-43	15q11.2	97.858	2.67E-09	8.5727	1.51E-08	P10 - ctrl; P11 - ctrl; P2 - ctrl; P3 - ctrl; P8 - ctrl; P10 - P11; P10 - P2; P3 - P10; P3 - P11; P8 - P11; P3 - P2; P8 - P2; P3 - P8
TC1500006598.hg.1	TC1500006598.hg.1	NR_003314	SNORD115-22	15q11.2	97.254	2.77E-09	8.5571	1.56E-08	P10 - ctrl; P11 - ctrl; P2 - ctrl; P3 - ctrl; P8 - ctrl; P10 - P11; P10 - P2; P3 - P10; P3 - P11; P8 - P11; P3 - P2; P8 - P2; P3 - P8
TC0200016684.hg.1	TC0200016684.hg.1	NM_022037	TIA1	7072	97.047	2.81E-09	8.5517	1.58E-08	ctrl - P10; ctrl - P11; ctrl - P3; ctrl - P8; P11 - P10; P2 - P10; P3 - P10; P8 - P10; P2 - P11; P3 - P11; P11 - P8; P2 - P3; P2 - P8
TC0600006976.hg.1	TC0600006976.hg.1	NM_001242648	PHACTR1	221692	96.87	2.84E-09	8.5471	1.59E-08	ctrl - P10; P11 - ctrl; ctrl - P2; ctrl - P3; ctrl - P8; P11 - P10; P2 - P10; P3 - P10; P10 - P8; P11 - P2; P11 - P3; P11 - P8; P2 - P3; P2 - P8; P3 - P8
TC1500007392.hg.1	TC1500007392.hg.1	NM_004701	CCNB2	9133	96.298	2.94E-09	8.5321	1.65E-08	P10 - ctrl; ctrl - P2; P3 - ctrl; P8 - ctrl; P10 - P11; P10 - P2; P10 - P3; P8 - P10; P11 - P2; P3 - P11; P8 - P11; P3 - P2; P8 - P2; P8 - P3
TC2100006539.hg.1	TC2100006539.hg.1	NA	NA	NA	96.204	2.95E-09	8.5296	1.65E-08	ctrl - P11; P2 - ctrl; P3 - ctrl; ctrl - P8; P10 - P11; P2 - P10; P3 - P10; P10 - P8; P2 - P11; P3 - P11; P8 - P11; P2 - P3; P2 - P8; P3 - P8
TC0600008544.hg.1	TC0600008544.hg.1	NR_110856	LOC101928516	1.02E+08	96.169	2.96E-09	8.5287	1.65E-08	P10 - ctrl; P2 - ctrl; P3 - ctrl; P8 - ctrl; P10 - P11; P10 - P3; P2 - P11; P3 - P11; P8 - P11; P2 - P3; P8 - P3
TC1700012395.hg.1	TC1700012395.hg.1	OTTHUMT00000477403	TBC1D3L	1.01E+08	95.924	3.00E-09	8.5222	1.67E-08	ctrl - P10; ctrl - P11; ctrl - P3; ctrl - P8; P11 - P10; P2 - P10; P3 - P10; P2 - P11; P11 - P8; P2 - P3; P2 - P8; P3 - P8
TC0700008100.hg.1	TC0700008100.hg.1	barmorby.aAug10-unspliced	barmorby	NA	95.897	3.01E-09	8.5215	1.67E-08	ctrl - P10; ctrl - P11; ctrl - P3; ctrl - P8; P11 - P10; P2 - P10; P3 - P10; P2 - P11; P11 - P8; P2 - P3; P2 - P8; P3 - P8
TC2000007503.hg.1	TC2000007503.hg.1	NM_001281741	UBE2C	11065	95.762	3.03E-09	8.518	1.68E-08	P10 - ctrl; ctrl - P2; P8 - ctrl; P10 - P11; P10 - P2; P10 - P3; P8 - P10; P3 - P11; P8 - P11; P3 - P2; P8 - P2; P8 - P3
TC0400012971.hg.1	TC0400012971.hg.1	NA	NA	NA	95.476	3.09E-09	8.5104	1.71E-08	ctrl - P10; ctrl - P11; ctrl - P2; ctrl - P3; ctrl - P8; P11 - P10; P2 - P10; P3 - P10; P11 - P2; P11 - P3; P11 - P8; P2 - P8; P3 - P8
TC0600013757.hg.1	TC0600013757.hg.1	NM_000636	SOD2	6648	95.391	3.10E-09	8.5081	1.71E-08	P10 - ctrl; P11 - ctrl; P3 - ctrl; P8 - ctrl; P10 - P11; P10 - P2; P10 - P3; P10 - P8; P11 - P2; P8 - P11; P3 - P2; P8 - P2; P8 - P3
TC0700012096.hg.1	TC0700012096.hg.1	boneyby.aAug10-unspliced	boneyby	NA	95.265	3.13E-09	8.5048	1.72E-08	ctrl - P10; ctrl - P11; P2 - ctrl; ctrl - P3; ctrl - P8; P2 - P10; P3 - P10; P10 - P8; P2 - P11; P3 - P11; P11 - P8; P2 - P3; P2 - P8; P3 - P8
TC2000007089.hg.1	TC2000007089.hg.1	NM_012112	TPX2	22974	95.192	3.14E-09	8.5029	1.73E-08	P10 - ctrl; ctrl - P11; ctrl - P2; P3 - ctrl; P8 - ctrl; P10 - P11; P10 - P2; P10 - P3; P8 - P10; P3 - P11; P8 - P11; P3 - P2; P8 - P2; P8 - P3
TC1000010727.hg.1	TC1000010727.hg.1	NM_001149	ANK3	288	95.103	3.16E-09	8.5005	1.73E-08	ctrl - P10; ctrl - P3; ctrl - P8; P11 - P10; P2 - P10; P11 - P3; P11 - P8; P2 - P3; P2 - P8
TC1100011185.hg.1	TC1100011185.hg.1	NA	NA	NA	94.796	3.22E-09	8.4923	1.76E-08	ctrl - P10; ctrl - P11; ctrl - P3; ctrl - P8; P11 - P10; P2 - P10; P3 - P10; P2 - P11; P11 - P8; P2 - P3; P2 - P8; P3 - P8

TC0500011004.hg.1	TC0500011004.hg.1	OTTHUMT00000369094	GUSBP3	653188	94.724	3.23E-09	8.4904	1.76E-08	ctrl - P10; ctrl - P11; ctrl - P3; ctrl - P8; P11 - P10; P2 - P10; P3 - P10; P10 - P8; P2 - P11; P11 - P3; P11 - P8; P2 - P3; P2 - P8; P3 - P8
TC0100018431.hg.1	TC0100018431.hg.1	ENST00000466213	SFPQ	1p34.3	94.259	3.33E-09	8.478	1.81E-08	ctrl - P10; ctrl - P11; ctrl - P3; ctrl - P8; P11 - P10; P2 - P10; P3 - P10; P8 - P10; P2 - P11; P11 - P8; P2 - P3; P2 - P8; P3 - P8
TC0600011809.hg.1	TC0600011809.hg.1	NM_001136017	CCND3	896	94.201	3.34E-09	8.4764	1.81E-08	P10 - ctrl; ctrl - P11; ctrl - P2; P3 - ctrl; P8 - ctrl; P10 - P11; P10 - P2; P3 - P10; P3 - P11; P8 - P11; P3 - P2; P8 - P2
TC2000008315.hg.1	TC2000008315.hg.1	NA	NA	NA	94.127	3.35E-09	8.4744	1.82E-08	ctrl - P11; ctrl - P2; P3 - ctrl; P8 - ctrl; P10 - P11; P10 - P2; P3 - P10; P8 - P10; P2 - P11; P3 - P11; P8 - P11; P3 - P2; P8 - P2
TC1700008103.hg.1	TC1700008103.hg.1	NA	NA	NA	94.09	3.36E-09	8.4734	1.82E-08	ctrl - P10; ctrl - P11; P2 - ctrl; ctrl - P3; ctrl - P8; P11 - P10; P2 - P10; P10 - P8; P2 - P11; P11 - P8; P2 - P3; P2 - P8; P3 - P8
TC1300008558.hg.1	TC1300008558.hg.1	NR_026928	N4BP2L2-IT2	116828	94.053	3.37E-09	8.4724	1.82E-08	ctrl - P10; ctrl - P11; P2 - ctrl; ctrl - P3; ctrl - P8; P11 - P10; P2 - P10; P3 - P10; P10 - P8; P2 - P11; P11 - P8; P2 - P3; P2 - P8; P3 - P8
TC0100014543.hg.1	TC0100014543.hg.1	NM_001114120	DEPDC1	55635	94.005	3.38E-09	8.4711	1.82E-08	P10 - ctrl; ctrl - P11; ctrl - P2; P8 - ctrl; P10 - P11; P10 - P2; P10 - P3; P8 - P10; P3 - P11; P8 - P11; P3 - P2; P8 - P2; P8 - P3
TC1900008287.hg.1	TC1900008287.hg.1	NM_001042724	PVRL2	5819	93.625	3.46E-09	8.4609	1.86E-08	P10 - ctrl; ctrl - P11; ctrl - P2; P8 - ctrl; P10 - P11; P10 - P2; P10 - P3; P8 - P10; P11 - P2; P3 - P11; P8 - P11; P3 - P2; P8 - P2; P8 - P3
TC0700011519.hg.1	TC0700011519.hg.1	NR_040584	STAG3L2	442582	93.62	3.46E-09	8.4608	1.86E-08	ctrl - P10; ctrl - P11; ctrl - P3; ctrl - P8; P11 - P10; P2 - P10; P3 - P10; P2 - P11; P11 - P8; P2 - P3; P2 - P8; P3 - P8
TC1100007727.hg.1	TC1100007727.hg.1	NM_001142703	FAM111B	374393	93.581	3.47E-09	8.4597	1.86E-08	P10 - ctrl; ctrl - P11; ctrl - P2; P3 - ctrl; P8 - ctrl; P10 - P11; P10 - P2; P3 - P10; P8 - P10; P2 - P11; P3 - P11; P8 - P11; P3 - P2; P8 - P2
TC0200007717.hg.1	TC0200007717.hg.1	NM_152392	AHSA2	130872	93.483	3.49E-09	8.4571	1.86E-08	ctrl - P10; ctrl - P11; ctrl - P2; ctrl - P3; ctrl - P8; P11 - P10; P2 - P10; P3 - P10; P2 - P11; P11 - P8; P2 - P3; P2 - P8; P3 - P8
TC0100010544.hg.1	TC0100010544.hg.1	momybu.aAug10-unspliced	momybu	NA	93.464	3.49E-09	8.4566	1.86E-08	ctrl - P10; ctrl - P11; ctrl - P3; ctrl - P8; P11 - P10; P2 - P10; P10 - P3; P10 - P8; P2 - P11; P11 - P3; P11 - P8; P2 - P3; P2 - P8
TC0400008007.hg.1	TC0400008007.hg.1	NM_001256421	GPAT3	84803	93.421	3.50E-09	8.4554	1.86E-08	P10 - ctrl; ctrl - P2; P3 - ctrl; P8 - ctrl; P10 - P11; P10 - P2; P11 - P2; P3 - P11; P8 - P11; P3 - P2; P8 - P2
TC0300013863.hg.1	TC0300013863.hg.1	NM_006952	UPK1B	7348	92.976	3.60E-09	8.4434	1.91E-08	ctrl - P10; ctrl - P11; ctrl - P2; P3 - ctrl; ctrl - P8; P11 - P10; P2 - P10; P3 - P10; P8 - P10; P3 - P11; P8 - P11; P3 - P2; P8 - P2; P3 - P8
TC1300007561.hg.1	TC1300007561.hg.1	NM_001160706	SCEL	8796	92.496	3.71E-09	8.4303	1.97E-08	P10 - ctrl; ctrl - P2; P3 - ctrl; P8 - ctrl; P10 - P11; P10 - P2; P3 - P10; P8 - P10; P3 - P11; P8 - P11; P3 - P2; P8 - P2
TC2000008722.hg.1	TC2000008722.hg.1	NM_001252675	ACSS1	84532	92.218	3.78E-09	8.4227	2.00E-08	ctrl - P10; ctrl - P2; ctrl - P3; ctrl - P8; P11 - P10; P10 - P3; P10 - P8; P11 - P2; P11 - P3; P11 - P8; P2 - P8
TC1100008453.hg.1	TC1100008453.hg.1	NM_001145211	SLCO2B1	11309	92.052	3.82E-09	8.4181	2.01E-08	P10 - ctrl; P3 - ctrl; P8 - ctrl; P10 - P11; P10 - P2; P3 - P10; P8 - P10; P3 - P11; P8 - P11; P3 - P2; P8 - P2; P8 - P3
TC1500009813.hg.1	TC1500009813.hg.1	ENST00000288745	MEGF11	84465	91.812	3.88E-09	8.4115	2.04E-08	P10 - ctrl; P2 - ctrl; P3 - ctrl; P8 - ctrl; P10 - P11; P10 - P2; P10 - P3; P10 - P8; P2 - P11; P3 - P11; P8 - P11; P8 - P2; P8 - P3
TC0700010443.hg.1	TC0700010443.hg.1	NM_001164460	STEAP1B	256227	91.803	3.88E-09	8.4113	2.04E-08	P10 - ctrl; P11 - ctrl; P3 - ctrl; P8 - ctrl; P10 - P11; P10 - P2; P3 - P10; P8 - P10; P3 - P11; P8 - P11; P3 - P2; P8 - P2
TC1500009806.hg.1	TC1500009806.hg.1	uc059kld.1	snoU13	NA	91.757	3.89E-09	8.41	2.04E-08	ctrl - P10; ctrl - P11; P2 - ctrl; ctrl - P3; ctrl - P8; P11 - P10; P2 - P10; P3 - P10; P2 - P11; P11 - P8; P2 - P3; P2 - P8; P3 - P8
TC0500007761.hg.1	TC0500007761.hg.1	NM_001161342	TMEM171	134285	91.515	3.95E-09	8.4033	2.07E-08	ctrl - P10; ctrl - P11; ctrl - P2; ctrl - P3; ctrl - P8; P11 - P10; P10 - P2; P10 - P3; P11 - P2; P11 - P3; P11 - P8; P8 - P2; P8 - P3
TC0700011396.hg.1	TC0700011396.hg.1	NA	NA	NA	91.231	4.02E-09	8.3955	2.10E-08	ctrl - P10; ctrl - P11; P2 - ctrl; ctrl - P3; ctrl - P8; P2 - P10; P3 - P10; P10 - P8; P2 - P11; P3 - P11; P11 - P8; P2 - P3; P2 - P8; P3 - P8
TC0100013872.hg.1	TC0100013872.hg.1	NM_001302269	EDN2	1907	91.224	4.02E-09	8.3953	2.10E-08	ctrl - P11; ctrl - P2; ctrl - P3; P8 - ctrl; P10 - P11; P10 - P2; P10 - P3; P8 - P10; P3 - P11; P8 - P11; P3 - P2; P8 - P2; P8 - P3
TC0800007187.hg.1	TC0800007187.hg.1	mayaya.aAug10-unspliced	mayaya	NA	91.153	4.04E-09	8.3933	2.10E-08	ctrl - P10; ctrl - P11; P2 - ctrl; ctrl - P3; ctrl - P8; P10 - P11; P2 - P10; P10 - P8; P2 - P11; P3 - P11; P2 - P3; P2 - P8; P3 - P8
TC0300012814.hg.1	TC0300012814.hg.1	NM_023915	GPR87	53836	90.651	4.17E-09	8.3794	2.16E-08	P10 - ctrl; ctrl - P11; P3 - ctrl; P8 - ctrl; P10 - P11; P10 - P2; P3 - P10; P8 - P10; P2 - P11; P3 - P11; P8 - P11; P3 - P2; P8 - P2
TC0700012913.hg.1	TC0700012913.hg.1	kosmarbu.aAug10-unspliced	kosmarbu	NA	90.62	4.18E-09	8.3785	2.16E-08	ctrl - P10; ctrl - P11; P2 - ctrl; ctrl - P3; ctrl - P8; P11 - P10; P2 - P10; P3 - P10; P10 - P8; P2 - P11; P11 - P8; P2 - P3; P2 - P8; P3 - P8
TC0100011533.hg.1	TC0100011533.hg.1	NM_001030287	ATF3	467	90.574	4.20E-09	8.3773	2.17E-08	P10 - ctrl; P11 - ctrl; P2 - ctrl; P3 - ctrl; P8 - ctrl; P10 - P11; P10 - P2; P10 - P3; P8 - P10; P3 - P11; P8 - P11; P3 - P2; P8 - P2; P8 - P3
TC0200009739.hg.1	TC0200009739.hg.1	NM_014568	GALNT5	11227	90.501	4.21E-09	8.3752	2.17E-08	P10 - ctrl; P2 - ctrl; P3 - ctrl; P8 - ctrl; P10 - P11; P10 - P2; P8 - P10; P2 - P11; P3 - P11; P8 - P11; P3 - P2; P8 - P2; P8 - P3
TC1200010038.hg.1	TC1200010038.hg.1	NM_004447	EPS8	2059	90.423	4.24E-09	8.373	2.18E-08	P10 - ctrl; P2 - ctrl; P3 - ctrl; P8 - ctrl; P10 - P11; P10 - P2; P10 - P3; P10 - P8; P2 - P11; P3 - P11; P8 - P11; P8 - P2; P8 - P3
TC0500010510.hg.1	TC0500010510.hg.1	NA	NA	NA	90.35	4.26E-09	8.371	2.18E-08	ctrl - P10; ctrl - P11; P2 - ctrl; ctrl - P3; ctrl - P8; P2 - P10; P3 - P10; P10 - P8; P2 - P11; P3 - P11; P11 - P8; P2 - P3; P2 - P8; P3 - P8
TC0600008146.hg.1	TC0600008146.hg.1	NM_001015051	RUNX2	860	89.921	4.38E-09	8.359	2.24E-08	P10 - ctrl; ctrl - P2; P3 - ctrl; P8 - ctrl; P10 - P11; P10 - P2; P3 - P10; P8 - P10; P11 - P2; P3 - P11; P8 - P11; P3 - P2; P8 - P2
TC0800007640.hg.1	TC0800007640.hg.1	ENST00000517688	RP11-463C14.1	NA	89.581	4.47E-09	8.3494	2.29E-08	ctrl - P10; ctrl - P2; ctrl - P3; ctrl - P8; P11 - P10; P2 - P10; P3 - P10; P11 - P2; P11 - P3; P11 - P8; P2 - P8; P3 - P8
TC0800007732.hg.1	TC0800007732.hg.1	toyskoybu.aAug10-unspliced	toyskoybu	NA	89.427	4.52E-09	8.3451	2.30E-08	ctrl - P10; ctrl - P11; P2 - ctrl; ctrl - P3; ctrl - P8; P11 - P10; P2 - P10; P3 - P10; P10 - P8; P2 - P11; P11 - P8; P2 - P3; P2 - P8; P3 - P8

TC0800008243.hg.1	TC0800008243.hg.1	NM_001161779	PDP1	54704	89.386	4.53E-09	8.3439	2.31E-08	P10 - ctrl; P2 - ctrl; P3 - ctrl; P8 - ctrl; P10 - P11; P10 - P2; P10 - P3; P2 - P11; P3 - P11; P8 - P11; P3 - P2; P8 - P2; P8 - P3
TC1300009812.hg.1	TC1300009812.hg.1	ENST00000426991.1	ANKRD10-IT1		89.149	4.60E-09	8.3372	2.34E-08	ctrl - P10; ctrl - P11; ctrl - P3; ctrl - P8; P11 - P10; P2 - P10; P3 - P10; P10 - P8; P2 - P11; P11 - P8; P2 - P3; P2 - P8; P3 - P8
TC0100009873.hg.1	TC0100009873.hg.1	uc057klq.1	AC239868.3	NA	88.936	4.66E-09	8.3312	2.36E-08	P10 - ctrl; P11 - ctrl; ctrl - P2; P8 - ctrl; P10 - P11; P10 - P2; P10 - P3; P10 - P8; P11 - P2; P11 - P3; P8 - P11; P3 - P2; P8 - P2; P8 - P3
TC1400007495.hg.1	TC1400007495.hg.1	NM_020715	PLEKHH1	57475	88.646	4.75E-09	8.3229	2.40E-08	ctrl - P10; ctrl - P11; ctrl - P3; ctrl - P8; P11 - P10; P2 - P10; P3 - P10; P2 - P11; P11 - P3; P11 - P8; P2 - P3; P2 - P8; P3 - P8
TC1600008917.hg.1	TC1600008917.hg.1	neereyby.aAug10-unspliced	neereyby	NA	88.246	4.88E-09	8.3116	2.46E-08	ctrl - P10; ctrl - P11; P2 - ctrl; ctrl - P3; ctrl - P8; P11 - P10; P2 - P10; P3 - P10; P10 - P8; P2 - P11; P11 - P8; P2 - P3; P2 - P8; P3 - P8
TC0800006975.hg.1	TC0800006975.hg.1	NM_001199	BMP1	649	88.207	4.89E-09	8.3105	2.47E-08	P10 - ctrl; ctrl - P2; P3 - ctrl; P8 - ctrl; P10 - P11; P10 - P2; P10 - P3; P8 - P10; P11 - P2; P3 - P11; P8 - P11; P3 - P2; P8 - P2; P8 - P3
TC1500007346.hg.1	TC1500007346.hg.1	NM_001252335	CGNL1	84952	88.047	4.94E-09	8.3059	2.49E-08	ctrl - P10; ctrl - P2; ctrl - P3; ctrl - P8; P11 - P10; P2 - P10; P3 - P10; P11 - P2; P11 - P3; P11 - P8; P3 - P2; P3 - P8
TC0500009857.hg.1	TC0500009857.hg.1	blorplaby.aAug10-unspliced	blorplaby	NA	87.999	4.96E-09	8.3045	2.49E-08	ctrl - P10; ctrl - P11; P2 - ctrl; ctrl - P3; ctrl - P8; P2 - P10; P10 - P8; P2 - P11; P11 - P8; P2 - P3; P2 - P8; P3 - P8
TC1900009896.hg.1	TC1900009896.hg.1	NM_001128932	CYP4F11	57834	87.7	5.06E-09	8.2959	2.53E-08	P10 - ctrl; P11 - ctrl; ctrl - P2; P3 - ctrl; P8 - ctrl; P10 - P11; P10 - P2; P10 - P3; P11 - P2; P3 - P11; P8 - P11; P3 - P2; P8 - P2; P8 - P3
TC0600013010.hg.1	TC0600013010.hg.1	NM_005907	MAN1A1	4121	87.454	5.14E-09	8.2888	2.57E-08	ctrl - P10; ctrl - P2; ctrl - P3; ctrl - P8; P11 - P10; P2 - P10; P3 - P10; P11 - P2; P11 - P3; P11 - P8; P2 - P3; P2 - P8; P3 - P8
TC0X00007240.hg.1	TC0X00007240.hg.1	NM_000084	CLCN5	1184	87.292	5.20E-09	8.2841	2.59E-08	ctrl - P10; ctrl - P2; ctrl - P3; ctrl - P8; P11 - P10; P2 - P10; P11 - P2; P11 - P3; P11 - P8; P2 - P3; P2 - P8
TC1400009422.hg.1	TC1400009422.hg.1	NM_001308154	RAB15	376267	87.287	5.20E-09	8.284	2.59E-08	P10 - ctrl; ctrl - P2; P3 - ctrl; P8 - ctrl; P10 - P11; P10 - P2; P8 - P10; P3 - P11; P8 - P11; P3 - P2; P8 - P2; P8 - P3
TC2100006467.hg.1	TC2100006467.hg.1	NR_037458	MIR3687-1	1.01E+08	87.017	5.29E-09	8.2762	2.63E-08	ctrl - P10; ctrl - P11; ctrl - P3; ctrl - P8; P10 - P11; P2 - P10; P10 - P3; P10 - P8; P2 - P11; P2 - P3; P2 - P8; P8 - P3
TC22_KI270733v1_random00006440.hg.1	TC22_KI270733v1_random00006440.hg.1	NR_037458_4	MIR3687-1		87.017	5.29E-09	8.2762	2.63E-08	ctrl - P10; ctrl - P11; ctrl - P3; ctrl - P8; P10 - P11; P2 - P10; P10 - P3; P10 - P8; P2 - P11; P2 - P3; P2 - P8; P8 - P3
TC0900009425.hg.1	TC0900009425.hg.1	ENST00000457383.1	RP11-70J12.1		86.973	5.31E-09	8.2749	2.63E-08	ctrl - P10; ctrl - P11; ctrl - P3; ctrl - P8; P11 - P10; P2 - P10; P2 - P11; P11 - P3; P11 - P8; P2 - P3; P2 - P8; P3 - P8
TC0600007111.hg.1	TC0600007111.hg.1	NA	NA	NA	86.954	5.32E-09	8.2744	2.63E-08	P10 - ctrl; ctrl - P11; P2 - ctrl; P3 - ctrl; P8 - ctrl; P10 - P11; P10 - P2; P3 - P10; P2 - P11; P3 - P11; P8 - P11; P3 - P2; P3 - P8
TC0100008631.hg.1	TC0100008631.hg.1	NM_001308203	SGIP1	84251	86.803	5.37E-09	8.27	2.65E-08	ctrl - P10; ctrl - P11; ctrl - P2; P3 - ctrl; ctrl - P8; P11 - P10; P2 - P10; P3 - P10; P8 - P10; P3 - P11; P3 - P2; P3 - P8
TC0100014585.hg.1	TC0100014585.hg.1	NM_173808	NEGR1	257194	86.771	5.38E-09	8.2691	2.65E-08	P10 - ctrl; ctrl - P2; P3 - ctrl; P8 - ctrl; P10 - P11; P10 - P2; P3 - P10; P8 - P10; P11 - P2; P3 - P11; P8 - P11; P3 - P2; P8 - P2
TC1100012389.hg.1	TC1100012389.hg.1	NM_001098517	CADM1	23705	86.663	5.42E-09	8.2659	2.66E-08	P10 - ctrl; P11 - ctrl; P8 - ctrl; P10 - P11; P10 - P2; P10 - P3; P10 - P8; P11 - P2; P11 - P3; P8 - P11; P8 - P2; P8 - P3
TC1100009330.hg.1	TC1100009330.hg.1	NM_032873	UBASH3B	84959	86.416	5.51E-09	8.2587	2.70E-08	P10 - ctrl; ctrl - P11; ctrl - P2; P3 - ctrl; P8 - ctrl; P10 - P11; P10 - P2; P3 - P10; P8 - P10; P3 - P11; P8 - P11; P3 - P2; P8 - P2; P3 - P8
TC1600011546.hg.1	TC1600011546.hg.1	NM_015993	PLLP	51090	86.211	5.59E-09	8.2527	2.73E-08	P10 - ctrl; P2 - ctrl; P3 - ctrl; P8 - ctrl; P10 - P11; P10 - P2; P10 - P3; P8 - P10; P2 - P11; P3 - P11; P8 - P11; P3 - P2; P8 - P2; P8 - P3
TC1700009075.hg.1	TC1700009075.hg.1	meeter.aAug10-unspliced	meeter		86.072	5.64E-09	8.2487	2.75E-08	P10 - ctrl; P11 - ctrl; ctrl - P2; P3 - ctrl; P8 - ctrl; P10 - P11; P10 - P2; P10 - P3; P10 - P8; P11 - P2; P11 - P3; P3 - P2; P8 - P2; P8 - P3
TC0500010043.hg.1	TC0500010043.hg.1	NM_003966	SEMA5A	9037	85.98	5.68E-09	8.246	2.76E-08	ctrl - P10; ctrl - P3; ctrl - P8; P11 - P10; P2 - P10; P3 - P10; P11 - P3; P11 - P8; P2 - P3; P2 - P8; P3 - P8
TC0200011107.hg.1	TC0200011107.hg.1	NM_001243252	EFHD1	80303	85.863	5.72E-09	8.2425	2.78E-08	ctrl - P10; ctrl - P2; ctrl - P3; ctrl - P8; P11 - P10; P10 - P2; P10 - P3; P11 - P2; P11 - P3; P11 - P8; P8 - P2; P8 - P3
TC1600011453.hg.1	TC1600011453.hg.1	NM_001197181	TUBB3	10381	85.494	5.87E-09	8.2317	2.85E-08	P10 - ctrl; ctrl - P2; P3 - ctrl; P8 - ctrl; P10 - P11; P10 - P2; P10 - P3; P8 - P10; P3 - P11; P8 - P11; P3 - P2; P8 - P2; P8 - P3
TC1900011383.hg.1	TC1900011383.hg.1	ENST00000413496.2	AC008440.5		84.825	6.14E-09	8.2119	2.97E-08	P10 - ctrl; P3 - ctrl; P8 - ctrl; P10 - P11; P10 - P2; P10 - P3; P8 - P10; P3 - P11; P8 - P11; P3 - P2; P8 - P2; P8 - P3
TC1600011502.hg.1	TC1600011502.hg.1	NR_024456	LOC100190986	1E+08	84.787	6.15E-09	8.2108	2.98E-08	ctrl - P10; ctrl - P11; P2 - ctrl; ctrl - P3; ctrl - P8; P11 - P10; P2 - P10; P10 - P8; P2 - P11; P11 - P8; P2 - P3; P2 - P8; P3 - P8
TC0300011995.hg.1	TC0300011995.hg.1	NA	NA	NA	84.464	6.29E-09	8.2012	3.04E-08	ctrl - P10; ctrl - P11; P2 - ctrl; ctrl - P3; ctrl - P8; P2 - P10; P10 - P8; P2 - P11; P2 - P3; P2 - P8; P3 - P8
TC0100017172.hg.1	TC0100017172.hg.1	NM_001206696	IRF6	3664	84.311	6.36E-09	8.1966	3.06E-08	P10 - ctrl; ctrl - P2; P8 - ctrl; P10 - P11; P10 - P2; P10 - P3; P8 - P11; P8 - P2; P8 - P3
TC0200015621.hg.1	TC0200015621.hg.1	zerklu.aAug10-unspliced	zerklu	NA	84.297	6.37E-09	8.1962	3.06E-08	ctrl - P10; ctrl - P3; ctrl - P8; P11 - P10; P2 - P10; P10 - P3; P10 - P8; P11 - P3; P11 - P8; P2 - P3; P2 - P8
TC0100018528.hg.1	TC0100018528.hg.1	NM_003851	CREG1	8804	84.223	6.40E-09	8.194	3.07E-08	P11 - ctrl; ctrl - P2; ctrl - P3; P11 - P10; P10 - P2; P10 - P3; P10 - P8; P11 - P2; P11 - P3; P11 - P8; P8 - P2; P8 - P3
TC0600007262.hg.1	TC0600007262.hg.1	NM_003529	HIST1H3A	8350	84.183	6.42E-09	8.1928	3.07E-08	P10 - ctrl; ctrl - P2; P3 - ctrl; P8 - ctrl; P10 - P11; P10 - P2; P10 - P3; P11 - P2; P3 - P11; P8 - P11; P3 - P2; P8 - P2; P8 - P3
TC1100011841.hg.1	TC1100011841.hg.1	sportobo.aAug10-unspliced	sportobo	NA	84.079	6.46E-09	8.1897	3.09E-08	ctrl - P10; ctrl - P11; P2 - ctrl; ctrl - P3; ctrl - P8; P11 - P10; P2 - P10; P10 - P8; P2 - P11; P11 - P8; P2 - P3; P2 - P8; P3 - P8

TC0200014343.hg.1	TC0200014343.hg.1	NA	NA	NA	84.01	6.49E-09	8.1876	3.10E-08	ctrl - P11; P2 - ctrl; P3 - ctrl; ctrl - P8; P10 - P11; P2 - P10; P3 - P10; P10 - P8; P2 - P11; P3 - P11; P8 - P11; P2 - P3; P2 - P8; P3 - P8
TC2100006475.hg.1	TC2100006475.hg.1	uc061yyt.1	FP671120.1	NA	83.879	6.55E-09	8.1837	3.11E-08	ctrl - P10; ctrl - P11; ctrl - P3; ctrl - P8; P10 - P11; P2 - P10; P10 - P3; P10 - P8; P2 - P11; P2 - P3; P2 - P8; P8 - P3
TC0500013299.hg.1	TC0500013299.hg.1	ENST00000623525	RP11-1250I15.1		83.87	6.56E-09	8.1834	3.11E-08	ctrl - P10; ctrl - P11; ctrl - P3; ctrl - P8; P11 - P10; P2 - P10; P3 - P10; P2 - P11; P11 - P8; P2 - P3; P2 - P8; P3 - P8
TC1300009734.hg.1	TC1300009734.hg.1	plozoy.aAug10-unspliced	plozoy	NA	83.738	6.62E-09	8.1794	3.14E-08	ctrl - P10; ctrl - P11; P2 - ctrl; ctrl - P8; P2 - P10; P3 - P10; P10 - P8; P2 - P11; P3 - P11; P11 - P8; P2 - P3; P2 - P8; P3 - P8
TC0X00010734.hg.1	TC0X00010734.hg.1	NM_001163278	TENM1	10178	83.71	6.63E-09	8.1786	3.14E-08	ctrl - P10; ctrl - P2; ctrl - P3; ctrl - P8; P11 - P10; P2 - P10; P3 - P10; P11 - P2; P11 - P3; P11 - P8; P2 - P3; P2 - P8; P3 - P8
TC1600007353.hg.1	TC1600007353.hg.1	NM_001310136	NPIPB8	728734	83.477	6.74E-09	8.1716	3.18E-08	ctrl - P10; ctrl - P11; ctrl - P3; ctrl - P8; P11 - P10; P2 - P10; P3 - P10; P8 - P10; P2 - P11; P11 - P8; P2 - P3; P2 - P8; P3 - P8
TC0100016206.hg.1	TC0100016206.hg.1	NM_053282	SH2D1B	117157	83.459	6.75E-09	8.171	3.18E-08	P10 - ctrl; P11 - ctrl; P2 - ctrl; P3 - ctrl; P8 - ctrl; P10 - P11; P10 - P2; P10 - P3; P3 - P11; P8 - P11; P3 - P2; P8 - P2; P8 - P3
TC1800008902.hg.1	TC1800008902.hg.1	gloysar.aAug10-unspliced	gloysar	NA	83.413	6.77E-09	8.1696	3.18E-08	ctrl - P10; ctrl - P11; P2 - ctrl; ctrl - P3; ctrl - P8; P2 - P10; P2 - P11; P11 - P8; P2 - P3; P2 - P8
TC0400009086.hg.1	TC0400009086.hg.1	NM_000856	GUCY1A3	2982	83.255	6.84E-09	8.1649	3.21E-08	ctrl - P10; ctrl - P3; ctrl - P8; P11 - P10; P2 - P10; P3 - P10; P10 - P8; P11 - P3; P11 - P8; P2 - P3; P2 - P8; P3 - P8
TC0800007186.hg.1	TC0800007186.hg.1	sashor.aAug10	sashor	NA	83.239	6.85E-09	8.1644	3.21E-08	ctrl - P11; P2 - ctrl; ctrl - P8; P10 - P11; P2 - P10; P10 - P8; P2 - P11; P3 - P11; P2 - P3; P2 - P8; P3 - P8
TC0900009335.hg.1	TC0900009335.hg.1	fleewarby.aAug10-unspliced	fleewarby		82.746	7.09E-09	8.1494	3.32E-08	ctrl - P10; ctrl - P11; ctrl - P3; ctrl - P8; P2 - P10; P3 - P10; P10 - P8; P2 - P11; P11 - P8; P2 - P3; P2 - P8; P3 - P8
TC0700012684.hg.1	TC0700012684.hg.1	NM_001628	AKR1B1	231	82.719	7.10E-09	8.1486	3.32E-08	P10 - ctrl; ctrl - P11; ctrl - P2; P3 - ctrl; P8 - ctrl; P10 - P11; P10 - P2; P3 - P10; P3 - P11; P8 - P11; P3 - P2; P8 - P2
TC1100012513.hg.1	TC1100012513.hg.1	NM_002105	H2AFX	3014	82.454	7.24E-09	8.1405	3.37E-08	P10 - ctrl; ctrl - P2; P3 - ctrl; P8 - ctrl; P10 - P11; P10 - P2; P10 - P3; P8 - P10; P11 - P2; P3 - P11; P8 - P11; P3 - P2; P8 - P2; P8 - P3
TC0500013184.hg.1	TC0500013184.hg.1	NR_029426	SMA4	11039	82.283	7.32E-09	8.1353	3.41E-08	ctrl - P10; ctrl - P11; ctrl - P3; ctrl - P8; P11 - P10; P2 - P10; P3 - P10; P10 - P8; P2 - P11; P11 - P3; P11 - P8; P2 - P3; P2 - P8; P3 - P8
TC0200016503.hg.1	TC0200016503.hg.1	NM_006236	POU3F3	5455	82.155	7.39E-09	8.1314	3.43E-08	ctrl - P10; P11 - ctrl; ctrl - P2; ctrl - P3; ctrl - P8; P11 - P10; P2 - P10; P3 - P10; P11 - P2; P11 - P3; P11 - P8; P2 - P3; P2 - P8; P3 - P8
TC0600007376.hg.1	TC0600007376.hg.1	uc063mkd.1	AL049822.1	NA	82.147	7.39E-09	8.1311	3.43E-08	P10 - ctrl; P11 - ctrl; ctrl - P2; P8 - ctrl; P10 - P11; P10 - P2; P10 - P3; P11 - P2; P11 - P3; P8 - P11; P3 - P2; P8 - P2; P8 - P3
TC0600008310.hg.1	TC0600008310.hg.1	NM_014464	TINAG	27283	82.089	7.42E-09	8.1294	3.44E-08	ctrl - P10; P11 - ctrl; ctrl - P2; P3 - ctrl; ctrl - P8; P11 - P10; P2 - P10; P3 - P10; P11 - P2; P3 - P11; P11 - P8; P3 - P2; P2 - P8; P3 - P8
TC0400006956.hg.1	TC0400006956.hg.1	NA	NA	NA	82.031	7.45E-09	8.1276	3.44E-08	P10 - ctrl; P3 - ctrl; P8 - ctrl; P10 - P11; P10 - P2; P10 - P3; P3 - P11; P8 - P11; P3 - P2; P8 - P2; P8 - P3
TC0600007374.hg.1	TC0600007374.hg.1	NM_003509	HIST1H2AI	8329	81.459	7.76E-09	8.11	3.57E-08	P10 - ctrl; ctrl - P2; P3 - ctrl; P8 - ctrl; P10 - P11; P10 - P2; P10 - P3; P11 - P2; P3 - P11; P8 - P11; P3 - P2; P8 - P2; P8 - P3
TC0300007056.hg.1	TC0300007056.hg.1	jeefarbo.aAug10-unspliced	jeefarbo	NA	81.454	7.77E-09	8.1098	3.57E-08	ctrl - P10; ctrl - P11; P2 - ctrl; ctrl - P3; ctrl - P8; P11 - P10; P2 - P10; P3 - P10; P10 - P8; P2 - P11; P11 - P3; P11 - P8; P2 - P3; P2 - P8; P3 - P8
TC0100010713.hg.1	TC0100010713.hg.1	NM_020318	PAPPA2	60676	81.175	7.92E-09	8.1012	3.64E-08	ctrl - P10; P11 - ctrl; ctrl - P2; ctrl - P8; P11 - P10; P3 - P10; P11 - P2; P11 - P3; P11 - P8; P3 - P2; P3 - P8
TC1700009064.hg.1	TC1700009064.hg.1	ENST00000587434.1	RP11-323N12.5		81.029	8.00E-09	8.0967	3.67E-08	P10 - ctrl; P11 - ctrl; P3 - ctrl; P8 - ctrl; P10 - P2; P10 - P3; P10 - P8; P11 - P2; P11 - P3; P11 - P8; P3 - P2; P8 - P2
TC0X00010851.hg.1	TC0X00010851.hg.1	NM_001164617	GPC3	2719	80.871	8.10E-09	8.0918	3.71E-08	ctrl - P10; ctrl - P11; ctrl - P2; ctrl - P3; ctrl - P8; P11 - P10; P2 - P10; P11 - P3; P11 - P8; P2 - P3; P2 - P8
TC1400009186.hg.1	TC1400009186.hg.1	NM_014584	ERO1A	30001	80.543	8.29E-09	8.0815	3.79E-08	P10 - ctrl; P11 - ctrl; ctrl - P2; P3 - ctrl; P8 - ctrl; P10 - P11; P10 - P2; P10 - P3; P11 - P2; P8 - P11; P3 - P2; P8 - P2; P8 - P3
TC0300008284.hg.1	TC0300008284.hg.1	NM_001282556	HHLA2	11148	80.486	8.32E-09	8.0798	3.80E-08	P10 - ctrl; P11 - ctrl; P8 - ctrl; P10 - P11; P10 - P2; P10 - P3; P10 - P8; P11 - P2; P11 - P3; P8 - P11; P8 - P2; P8 - P3
TC0700008183.hg.1	TC0700008183.hg.1	zysheeby.aAug10-unspliced	zysheeby	NA	80.253	8.46E-09	8.0725	3.85E-08	ctrl - P10; ctrl - P11; P2 - ctrl; ctrl - P3; ctrl - P8; P11 - P10; P2 - P10; P3 - P10; P2 - P11; P11 - P8; P2 - P3; P2 - P8; P3 - P8
TC0500007697.hg.1	TC0500007697.hg.1	ENST00000505516	RP11-589F5.4	NA	79.996	8.62E-09	8.0644	3.92E-08	ctrl - P10; ctrl - P11; ctrl - P3; ctrl - P8; P11 - P10; P2 - P10; P3 - P10; P2 - P11; P11 - P3; P11 - P8; P2 - P3; P2 - P8; P3 - P8
TC1900010596.hg.1	TC1900010596.hg.1	NA	NA	NA	79.786	8.75E-09	8.0578	3.97E-08	P10 - ctrl; P11 - ctrl; P3 - ctrl; P8 - ctrl; P10 - P11; P10 - P2; P10 - P3; P11 - P2; P8 - P11; P3 - P2; P8 - P2; P8 - P3
TC0700007000.hg.1	TC0700007000.hg.1	NR_038366	HOTAIRM1	1.01E+08	79.646	8.84E-09	8.0534	4.00E-08	ctrl - P10; ctrl - P11; ctrl - P2; ctrl - P3; ctrl - P8; P11 - P10; P2 - P10; P11 - P3; P11 - P8; P2 - P3; P2 - P8; P8 - P3
TC0600014192.hg.1	TC0600014192.hg.1	ENST00000427015.1	RP11-307P5.1		79.569	8.89E-09	8.0509	4.02E-08	P10 - ctrl; P2 - ctrl; P3 - ctrl; P8 - ctrl; P10 - P11; P2 - P10; P10 - P8; P2 - P11; P3 - P11; P8 - P11; P2 - P3; P2 - P8
TC0100010155.hg.1	TC0100010155.hg.1	NM_001193300	SEMA4A	64218	79.425	8.99E-09	8.0464	4.05E-08	ctrl - P10; ctrl - P2; ctrl - P3; ctrl - P8; P11 - P10; P2 - P10; P3 - P10; P11 - P2; P11 - P3; P11 - P8; P2 - P8; P3 - P8
TC0600013326.hg.1	TC0600013326.hg.1	NM_022121	PERP	64065	79.014	9.26E-09	8.0334	4.17E-08	P10 - ctrl; P3 - ctrl; P8 - ctrl; P10 - P11; P10 - P2; P3 - P11; P8 - P11; P3 - P2; P8 - P2
TC1200010538.hg.1	TC1200010538.hg.1	NM_001143668	AMIGO2	347902	78.765	9.43E-09	8.0255	4.23E-08	P10 - ctrl; P3 - ctrl; P8 - ctrl; P10 - P11; P10 - P2; P10 - P3; P3 - P11; P8 - P11; P3 - P2; P8 - P2; P8 - P3

TSUnmapped00000246.hg.1	TSUnmapped00000246.hg.1	ENST00000627452	CCDC84	338657	78.764	9.43E-09	8.0254	4.23E-08	ctrl - P10; ctrl - P11; ctrl - P3; ctrl - P8; P2 - P10; P3 - P10; P2 - P11; P3 - P11; P11 - P8; P2 - P3; P2 - P8; P3 - P8
TC1100009187.hg.1	TC1100009187.hg.1	ENST00000525260.1	RP11-728F11.4		78.705	9.47E-09	8.0235	4.24E-08	ctrl - P10; ctrl - P2; ctrl - P3; ctrl - P8; P11 - P10; P10 - P2; P10 - P3; P10 - P8; P11 - P2; P11 - P3; P11 - P8; P2 - P3; P8 - P2; P8 - P3
TC1600009921.hg.1	TC1600009921.hg.1	barseybo.aAug10-unspliced	barseybo	NA	78.641	9.52E-09	8.0215	4.25E-08	ctrl - P10; ctrl - P11; P2 - ctrl; ctrl - P3; ctrl - P8; P11 - P10; P2 - P10; P3 - P10; P10 - P8; P2 - P11; P11 - P8; P2 - P3; P2 - P8; P3 - P8
TC0200013750.hg.1	TC0200013750.hg.1	NM_001039492	FHL2	2274	78.601	9.55E-09	8.0202	4.26E-08	P10 - ctrl; P11 - ctrl; ctrl - P2; P3 - ctrl; P8 - ctrl; P10 - P11; P10 - P2; P11 - P2; P3 - P11; P8 - P11; P3 - P2; P8 - P2
TC0400007750.hg.1	TC0400007750.hg.1	wadabo.aAug10-unspliced	wadabo	NA	78.41	9.68E-09	8.0141	4.31E-08	ctrl - P10; ctrl - P11; P2 - ctrl; ctrl - P8; P11 - P10; P2 - P10; P3 - P10; P2 - P11; P3 - P11; P11 - P8; P2 - P3; P2 - P8; P3 - P8
TC0700010671.hg.1	TC0700010671.hg.1	NM_001191056	PDE1C	5137	78.241	9.80E-09	8.0087	4.36E-08	P10 - ctrl; ctrl - P11; ctrl - P2; P3 - ctrl; P8 - ctrl; P10 - P11; P10 - P2; P2 - P11; P3 - P11; P8 - P11; P3 - P2; P8 - P2
TC0X00007053.hg.1	TC0X00007053.hg.1	NM_000240	MAOA	4128	78.17	9.85E-09	8.0064	4.37E-08	ctrl - P10; ctrl - P2; ctrl - P3; ctrl - P8; P11 - P10; P2 - P10; P3 - P10; P8 - P10; P11 - P2; P11 - P3; P11 - P8; P2 - P8; P3 - P8
TC0X00009044.hg.1	TC0X00009044.hg.1	NM_000273	GPR143	4935	78.112	9.90E-09	8.0045	4.38E-08	P10 - ctrl; ctrl - P11; ctrl - P2; ctrl - P3; P8 - ctrl; P10 - P11; P10 - P2; P10 - P3; P2 - P11; P8 - P11; P8 - P2; P8 - P3
TC1600007204.hg.1	TC1600007204.hg.1	wospar.aAug10-unspliced	wospar	NA	77.714	1.02E-08	7.9917	4.51E-08	ctrl - P10; ctrl - P11; ctrl - P3; ctrl - P8; P11 - P10; P2 - P10; P3 - P10; P10 - P8; P2 - P11; P11 - P8; P2 - P3; P2 - P8; P3 - P8
TC0300009855.hg.1	TC0300009855.hg.1	NM_001167928	IL1RAP	3556	76.943	1.08E-08	7.9667	4.77E-08	P10 - ctrl; ctrl - P2; P3 - ctrl; P8 - ctrl; P10 - P11; P10 - P2; P10 - P3; P11 - P2; P3 - P11; P8 - P11; P3 - P2; P8 - P2; P8 - P3
TC0100015830.hg.1	TC0100015830.hg.1	NM_002016	FLG	2312	76.91	1.08E-08	7.9656	4.77E-08	P2 - ctrl; P3 - ctrl; P8 - ctrl; P2 - P10; P3 - P10; P8 - P10; P2 - P11; P3 - P11; P8 - P11; P3 - P2; P8 - P2; P3 - P8
TC0600009362.hg.1	TC0600009362.hg.1	NM_012259	HEY2	23493	76.845	1.09E-08	7.9635	4.79E-08	ctrl - P10; P11 - ctrl; ctrl - P2; ctrl - P3; ctrl - P8; P11 - P10; P11 - P2; P11 - P3; P11 - P8
TC0900007457.hg.1	TC0900007457.hg.1	NR_111893	CNTNAP3P2	643827	76.774	1.09E-08	7.9611	4.80E-08	ctrl - P10; P2 - ctrl; ctrl - P8; P11 - P10; P2 - P10; P3 - P10; P2 - P11; P3 - P11; P11 - P8; P2 - P3; P2 - P8; P3 - P8
TC0800006669.hg.1	TC0800006669.hg.1	NR_125431	LOC101929128	1.02E+08	76.654	1.10E-08	7.9572	4.83E-08	P10 - ctrl; ctrl - P11; P3 - ctrl; P8 - ctrl; P10 - P11; P10 - P2; P10 - P3; P8 - P10; P2 - P11; P3 - P11; P8 - P11; P3 - P2; P8 - P2; P8 - P3
TC1800007986.hg.1	TC1800007986.hg.1	NM_005559	LAMA1	284217	76.653	1.10E-08	7.9572	4.83E-08	ctrl - P10; ctrl - P2; ctrl - P3; ctrl - P8; P11 - P10; P2 - P10; P3 - P10; P11 - P2; P11 - P3; P11 - P8; P2 - P3; P2 - P8; P3 - P8
TC0200014705.hg.1	TC0200014705.hg.1	jagarbo.aAug10-unspliced	jagarbo	NA	76.59	1.11E-08	7.9551	4.83E-08	ctrl - P10; ctrl - P11; ctrl - P3; ctrl - P8; P11 - P10; P2 - P10; P3 - P10; P2 - P11; P11 - P8; P2 - P3; P2 - P8; P3 - P8
TC1400010071.hg.1	TC1400010071.hg.1	NM_024734	CLMN	79789	76.571	1.11E-08	7.9545	4.83E-08	ctrl - P10; ctrl - P2; ctrl - P3; ctrl - P8; P11 - P10; P2 - P10; P3 - P10; P8 - P10; P11 - P2; P11 - P3; P11 - P8; P2 - P8; P3 - P8
TC0900009880.hg.1	TC0900009880.hg.1	vysoby.aAug10-unspliced	vysoby	NA	76.57	1.11E-08	7.9545	4.83E-08	ctrl - P10; ctrl - P11; ctrl - P3; ctrl - P8; P2 - P10; P3 - P10; P10 - P8; P2 - P11; P3 - P11; P11 - P8; P2 - P3; P2 - P8; P3 - P8
TC1100010111.hg.1	TC1100010111.hg.1	teygoby.aAug10-unspliced	teygoby	NA	76.453	1.12E-08	7.9506	4.87E-08	ctrl - P10; ctrl - P11; ctrl - P3; ctrl - P8; P11 - P10; P2 - P10; P3 - P10; P11 - P3; P11 - P8; P2 - P3; P2 - P8; P3 - P8
TC0800010285.hg.1	TC0800010285.hg.1	NM_001257180	SLC20A2	6575	76.388	1.13E-08	7.9485	4.88E-08	P10 - ctrl; P8 - ctrl; P10 - P11; P10 - P2; P10 - P3; P11 - P3; P8 - P11; P8 - P2; P8 - P3
TC1000008727.hg.1	TC1000008727.hg.1	NM_001077494	NFKB2	10q24	76.121	1.15E-08	7.9397	4.98E-08	P10 - ctrl; P3 - ctrl; P8 - ctrl; P10 - P11; P10 - P2; P8 - P10; P11 - P2; P3 - P11; P8 - P11; P3 - P2; P8 - P2; P8 - P3
TC2100006620.hg.1	TC2100006620.hg.1	geefly.aAug10-unspliced	geefly	NA	76.056	1.15E-08	7.9376	4.99E-08	ctrl - P10; ctrl - P11; P2 - ctrl; ctrl - P3; ctrl - P8; P11 - P10; P2 - P10; P3 - P10; P2 - P11; P11 - P8; P2 - P3; P2 - P8; P3 - P8
TC1300008092.hg.1	TC1300008092.hg.1	OTTHUMT00000474565	RP11-73M11.3		75.992	1.16E-08	7.9355	5.00E-08	ctrl - P10; ctrl - P11; ctrl - P3; ctrl - P8; P2 - P10; P10 - P8; P2 - P11; P11 - P3; P11 - P8; P2 - P3; P2 - P8; P3 - P8
TC1600006888.hg.1	TC1600006888.hg.1	NM_000246	CIITA	4261	75.983	1.16E-08	7.9352	5.00E-08	P10 - ctrl; P8 - ctrl; P10 - P11; P10 - P2; P10 - P3; P8 - P11; P8 - P2; P8 - P3
TC0100007584.hg.1	TC0100007584.hg.1	NM_001195001	PTPRU	10076	75.847	1.17E-08	7.9307	5.05E-08	P10 - ctrl; P11 - ctrl; ctrl - P2; P3 - ctrl; P8 - ctrl; P10 - P11; P10 - P2; P3 - P10; P8 - P10; P11 - P2; P3 - P11; P8 - P11; P3 - P2; P8 - P2
TC1200012327.hg.1	TC1200012327.hg.1	NM_001082959	SCARB1	949	75.821	1.18E-08	7.9298	5.05E-08	P10 - ctrl; P3 - ctrl; P8 - ctrl; P10 - P11; P10 - P2; P10 - P3; P3 - P11; P8 - P11; P3 - P2; P8 - P2; P8 - P3
TC1000009025.hg.1	TC1000009025.hg.1	NM_001165924	EMX2	2018	75.64	1.19E-08	7.9238	5.11E-08	ctrl - P10; ctrl - P3; ctrl - P8; P11 - P10; P2 - P10; P3 - P10; P10 - P8; P11 - P3; P11 - P8; P2 - P3; P2 - P8; P3 - P8
TC1100007692.hg.1	TC1100007692.hg.1	glyny.aAug10-unspliced	glyny	NA	75.454	1.21E-08	7.9176	5.17E-08	ctrl - P10; ctrl - P11; P2 - ctrl; ctrl - P3; ctrl - P8; P2 - P10; P3 - P10; P2 - P11; P3 - P11; P11 - P8; P2 - P3; P2 - P8; P3 - P8
TC1200010182.hg.1	TC1200010182.hg.1	NM_030762	BHLHE41	79365	75.345	1.22E-08	7.914	5.21E-08	P10 - ctrl; P11 - ctrl; P2 - ctrl; P3 - ctrl; P8 - ctrl; P11 - P10; P10 - P2; P3 - P10; P11 - P2; P3 - P11; P11 - P8; P3 - P2; P8 - P2; P3 - P8
TC0200008322.hg.1	TC0200008322.hg.1	NA	NA	NA	75.155	1.24E-08	7.9077	5.27E-08	ctrl - P10; ctrl - P11; ctrl - P3; ctrl - P8; P11 - P10; P2 - P10; P3 - P10; P2 - P11; P11 - P3; P11 - P8; P2 - P3; P2 - P8; P3 - P8
TC0300006580.hg.1	TC0300006580.hg.1	NM_001570	IRAK2	3656	75.094	1.24E-08	7.9056	5.29E-08	P10 - ctrl; P11 - ctrl; P3 - ctrl; P8 - ctrl; P10 - P11; P10 - P2; P3 - P10; P8 - P10; P11 - P2; P3 - P11; P8 - P11; P3 - P2; P8 - P2
TC0100015710.hg.1	TC0100015710.hg.1	uc057klw.1	AC239868.1	NA	75.074	1.24E-08	7.905	5.29E-08	P10 - ctrl; P11 - ctrl; P3 - ctrl; P8 - ctrl; P10 - P11; P10 - P2; P10 - P3; P10 - P8; P11 - P2; P11 - P3; P8 - P11; P3 - P2; P8 - P2; P8 - P3
TC0800006869.hg.1	TC0800006869.hg.1	NM_001008539	SLC7A2	6542	75.056	1.25E-08	7.9044	5.29E-08	P10 - ctrl; P11 - ctrl; P2 - ctrl; P8 - ctrl; P10 - P11; P10 - P2; P10 - P3; P10 - P8; P11 - P3; P8 - P11; P8 - P2; P8 - P3
TC0600013203.hg.1	TC0600013203.hg.1	NM_004666	VNN1	8876	74.936	1.26E-08	7.9003	5.33E-08	P10 - ctrl; P3 - ctrl; P8 - ctrl; P10 - P11; P10 - P2; P8 - P10; P3 - P11; P8 - P11; P3 - P2; P8 - P2; P8 - P3

TC0700006629.hg.1	TC0700006629.hg.1	NM_003088	FSCN1	6624	74.756	1.28E-08	7.8943	5.39E-08	P10 - ctrl; ctrl - P11; ctrl - P2; P3 - ctrl; P8 - ctrl; P10 - P11; P10 - P2; P3 - P10; P8 - P10; P3 - P11; P8 - P11; P3 - P2; P8 - P2
TC1700011097.hg.1	TC1700011097.hg.1	NM_018509	LRRC59	55379	74.723	1.28E-08	7.8932	5.40E-08	P10 - ctrl; ctrl - P2; P3 - ctrl; P8 - ctrl; P10 - P11; P10 - P2; P10 - P3; P11 - P2; P3 - P11; P8 - P11; P3 - P2; P8 - P2; P8 - P3
TC1100010207.hg.1	TC1100010207.hg.1	NM_001145811	SOX6	55553	74.59	1.29E-08	7.8887	5.44E-08	ctrl - P10; ctrl - P3; ctrl - P8; P11 - P10; P2 - P10; P3 - P10; P10 - P8; P11 - P3; P11 - P8; P2 - P3; P2 - P8; P3 - P8
TC0100009723.hg.1	TC0100009723.hg.1	NM_207418	FAM72D	728833	74.483	1.30E-08	7.8851	5.48E-08	P10 - ctrl; ctrl - P11; ctrl - P2; P8 - ctrl; P10 - P11; P10 - P2; P10 - P3; P8 - P10; P3 - P11; P8 - P11; P3 - P2; P8 - P2; P8 - P3
TC1800006448.hg.1	TC1800006448.hg.1	NM_001071	TYMS	7298	74.387	1.31E-08	7.8819	5.51E-08	P10 - ctrl; ctrl - P11; ctrl - P2; P3 - ctrl; P8 - ctrl; P10 - P11; P10 - P2; P8 - P10; P3 - P11; P8 - P11; P3 - P2; P8 - P2; P8 - P3
TC0800011589.hg.1	TC0800011589.hg.1	snerla.aAug10-unspliced	snerla	NA	74.22	1.33E-08	7.8763	5.57E-08	ctrl - P10; ctrl - P11; P2 - ctrl; ctrl - P8; P10 - P11; P2 - P10; P3 - P10; P10 - P8; P2 - P11; P3 - P11; P2 - P3; P2 - P8; P3 - P8
TC0700008050.hg.1	TC0700008050.hg.1	STAG3L1andTRIM73andNSUN5P1.voAug10	STAG3L1andTRIM73andNSUN5P1		74.204	1.33E-08	7.8757	5.57E-08	ctrl - P10; ctrl - P11; ctrl - P3; ctrl - P8; P11 - P10; P2 - P10; P3 - P10; P2 - P11; P11 - P8; P2 - P3; P2 - P8; P3 - P8
TC0600011669.hg.1	TC0600011669.hg.1	skargluby.aAug10-unspliced	skargluby		74.161	1.34E-08	7.8743	5.58E-08	P10 - ctrl; P11 - ctrl; P3 - ctrl; P8 - ctrl; P10 - P2; P10 - P3; P11 - P2; P11 - P3; P3 - P2; P8 - P2; P8 - P3
TC0500013322.hg.1	TC0500013322.hg.1	NM_022892	NAIP	4671	74.089	1.34E-08	7.8719	5.60E-08	ctrl - P10; ctrl - P11; P2 - ctrl; ctrl - P3; ctrl - P8; P11 - P10; P2 - P10; P3 - P10; P2 - P11; P11 - P8; P2 - P3; P2 - P8; P3 - P8
TC0100017420.hg.1	TC0100017420.hg.1	NM_007207	DUSP10	11221	73.926	1.36E-08	7.8663	5.67E-08	P10 - ctrl; P3 - ctrl; P8 - ctrl; P10 - P11; P10 - P2; P8 - P10; P3 - P11; P8 - P11; P3 - P2; P8 - P2; P8 - P3
TC1600007170.hg.1	TC1600007170.hg.1	ENST00000564271.1	CTB-31N19.3		73.801	1.37E-08	7.8621	5.71E-08	ctrl - P10; ctrl - P11; ctrl - P3; ctrl - P8; P11 - P10; P2 - P10; P10 - P8; P2 - P11; P11 - P3; P11 - P8; P2 - P3; P2 - P8; P3 - P8
TC0400010786.hg.1	TC0400010786.hg.1	peegaw.aAug10-unspliced	peegaw	NA	73.722	1.38E-08	7.8594	5.73E-08	ctrl - P10; ctrl - P11; P2 - ctrl; ctrl - P8; P2 - P10; P3 - P10; P10 - P8; P2 - P11; P3 - P11; P11 - P8; P2 - P3; P2 - P8; P3 - P8
TC0500012531.hg.1	TC0500012531.hg.1	NM_020167	NMUR2	56923	73.72	1.38E-08	7.8594	5.73E-08	P10 - ctrl; P3 - ctrl; P8 - ctrl; P10 - P11; P10 - P2; P8 - P10; P3 - P11; P8 - P11; P3 - P2; P8 - P2; P8 - P3
TC0400006930.hg.1	TC0400006930.hg.1	NM_001177381	CPEB2	132864	73.599	1.40E-08	7.8552	5.77E-08	ctrl - P10; ctrl - P3; ctrl - P8; P11 - P10; P2 - P10; P3 - P10; P11 - P3; P11 - P8; P2 - P3; P2 - P8; P3 - P8
TC0600009866.hg.1	TC0600009866.hg.1	NA	NA	NA	73.413	1.42E-08	7.8489	5.85E-08	ctrl - P10; ctrl - P11; ctrl - P3; ctrl - P8; P11 - P10; P2 - P10; P3 - P10; P2 - P11; P11 - P8; P2 - P3; P2 - P8; P3 - P8
TC1700011566.hg.1	TC1700011566.hg.1	NM_001243746	FAM20A	54757	73.057	1.46E-08	7.8367	6.01E-08	P10 - ctrl; P11 - ctrl; P3 - ctrl; P8 - ctrl; P10 - P11; P10 - P2; P10 - P3; P10 - P8; P8 - P11; P8 - P2; P8 - P3
TC0500013324.hg.1	TC0500013324.hg.1	NR_033968_7	GUSBP9		72.972	1.47E-08	7.8338	6.04E-08	ctrl - P10; ctrl - P11; ctrl - P3; ctrl - P8; P11 - P10; P2 - P10; P3 - P10; P10 - P8; P2 - P11; P11 - P8; P2 - P3; P2 - P8; P3 - P8
TC1700011892.hg.1	TC1700011892.hg.1	NM_003258	TK1	7083	72.907	1.47E-08	7.8316	6.06E-08	P10 - ctrl; ctrl - P11; ctrl - P2; P3 - ctrl; P8 - ctrl; P10 - P11; P10 - P2; P10 - P3; P8 - P10; P3 - P11; P8 - P11; P3 - P2; P8 - P2; P8 - P3
TC2000006444.hg.1	TC2000006444.hg.1	NM_001301188	TRIB3	57761	72.676	1.50E-08	7.8236	6.16E-08	P10 - ctrl; P11 - ctrl; ctrl - P2; P3 - ctrl; P8 - ctrl; P11 - P10; P10 - P2; P10 - P3; P11 - P2; P11 - P3; P3 - P2; P8 - P2; P8 - P3
TC0600010781.hg.1	TC0600010781.hg.1	ENST00000431401.1	RP1-290I10.5		72.524	1.52E-08	7.8184	6.22E-08	P10 - ctrl; P3 - ctrl; P8 - ctrl; P10 - P11; P10 - P2; P8 - P10; P3 - P11; P8 - P11; P3 - P2; P8 - P2; P8 - P3
TC0800010704.hg.1	TC0800010704.hg.1	wargey.aAug10-unspliced	wargey	NA	72.513	1.52E-08	7.818	6.22E-08	ctrl - P10; ctrl - P11; P2 - ctrl; ctrl - P8; P2 - P10; P3 - P10; P10 - P8; P2 - P11; P3 - P11; P11 - P8; P2 - P3; P2 - P8; P3 - P8
TC1300008668.hg.1	TC1300008668.hg.1	NM_005780	LHFP	10186	72.228	1.56E-08	7.8081	6.35E-08	ctrl - P10; ctrl - P11; ctrl - P2; P3 - ctrl; P11 - P10; P3 - P10; P8 - P10; P11 - P2; P3 - P11; P8 - P11; P3 - P2; P8 - P2; P3 - P8
TC0200009219.hg.1	TC0200009219.hg.1	NM_001080527	MYO7B	4648	72.143	1.57E-08	7.8052	6.38E-08	ctrl - P10; P11 - ctrl; ctrl - P2; ctrl - P3; ctrl - P8; P11 - P10; P10 - P3; P10 - P8; P11 - P2; P11 - P3; P11 - P8; P2 - P3; P2 - P8; P8 - P3
TC0500011146.hg.1	TC0500011146.hg.1	NM_001256574	ENC1	8507	71.896	1.60E-08	7.7966	6.50E-08	P10 - ctrl; ctrl - P2; P3 - ctrl; P8 - ctrl; P10 - P11; P10 - P2; P3 - P10; P8 - P10; P11 - P2; P3 - P11; P8 - P11; P3 - P2; P8 - P2
TC0X00011355.hg.1	TC0X00011355.hg.1	NM_001018109	PIR	8544	71.758	1.62E-08	7.7918	6.56E-08	P10 - ctrl; P11 - ctrl; P3 - ctrl; P8 - ctrl; P10 - P11; P10 - P2; P11 - P2; P3 - P11; P8 - P11; P3 - P2; P8 - P2
TC1500006582.hg.1	TC1500006582.hg.1	NR_003296	SNORD115-4	15q11.2	71.658	1.63E-08	7.7883	6.61E-08	P10 - ctrl; P11 - ctrl; P2 - ctrl; P3 - ctrl; P8 - ctrl; P10 - P11; P10 - P2; P3 - P10; P3 - P11; P8 - P11; P3 - P2; P8 - P2; P3 - P8
TC0800008511.hg.1	TC0800008511.hg.1	floydarby.aAug10-unspliced	floydarby	NA	71.632	1.63E-08	7.7874	6.61E-08	ctrl - P10; ctrl - P11; P2 - ctrl; ctrl - P3; ctrl - P8; P11 - P10; P2 - P10; P3 - P10; P2 - P11; P3 - P11; P11 - P8; P2 - P3; P2 - P8; P3 - P8
TC1700008069.hg.1	TC1700008069.hg.1	ENST00000586348.1	RP11-798G7.6		71.576	1.64E-08	7.7854	6.63E-08	ctrl - P10; ctrl - P11; P2 - ctrl; ctrl - P3; ctrl - P8; P11 - P10; P2 - P10; P2 - P11; P11 - P3; P11 - P8; P2 - P3; P2 - P8
TC1400008615.hg.1	TC1400008615.hg.1	NM_001282211	NDRG2	57447	71.352	1.67E-08	7.7776	6.73E-08	ctrl - P10; P11 - ctrl; ctrl - P2; ctrl - P3; ctrl - P8; P11 - P10; P2 - P10; P11 - P2; P11 - P3; P11 - P8; P2 - P8
TC1100009817.hg.1	TC1100009817.hg.1	NM_003311	PHLDA2	7262	71.345	1.67E-08	7.7773	6.73E-08	P10 - ctrl; P11 - ctrl; ctrl - P2; P3 - ctrl; P8 - ctrl; P10 - P11; P10 - P2; P3 - P10; P8 - P10; P11 - P2; P3 - P11; P8 - P11; P3 - P2; P8 - P2
TC1400008054.hg.1	TC1400008054.hg.1	NM_023112	OTUB2	78990	71.27	1.68E-08	7.7747	6.76E-08	P10 - ctrl; ctrl - P11; ctrl - P2; P3 - ctrl; P8 - ctrl; P10 - P11; P10 - P2; P3 - P10; P8 - P10; P3 - P11; P8 - P11; P3 - P2; P8 - P2; P3 - P8
TC1700009679.hg.1	TC1700009679.hg.1	NM_001256834	AURKB	9212	71.188	1.69E-08	7.7718	6.79E-08	P10 - ctrl; ctrl - P11; ctrl - P2; P8 - ctrl; P10 - P11; P10 - P2; P10 - P3; P8 - P10; P3 - P11; P8 - P11; P3 - P2; P8 - P2; P8 - P3
TC0900011708.hg.1	TC0900011708.hg.1	NM_004878	PTGES	9536	71.159	1.70E-08	7.7708	6.80E-08	P10 - ctrl; ctrl - P11; ctrl - P2; P3 - ctrl; P8 - ctrl; P10 - P11; P10 - P2; P10 - P3; P3 - P11; P8 - P11; P3 - P2; P8 - P2; P8 - P3

TC0100011512.hg.1	TC0100011512.hg.1	NM_001286229	DTL	51514	71.054	1.71E-08	7.7671	6.85E-08	P10 - ctrl; ctrl - P11; ctrl - P2; P3 - ctrl; P8 - ctrl; P10 - P11; P10 - P2; P3 - P10; P8 - P10; P2 - P11; P3 - P11; P8 - P11; P3 - P2; P8 - P2
TC0200014797.hg.1	TC0200014797.hg.1	chudey.aAug10-unspliced	chudey	NA	70.898	1.73E-08	7.7616	6.92E-08	ctrl - P10; ctrl - P11; ctrl - P3; ctrl - P8; P11 - P10; P2 - P10; P3 - P10; P10 - P8; P2 - P11; P11 - P8; P2 - P3; P2 - P8; P3 - P8
TC1000009899.hg.1	TC1000009899.hg.1	NA	NA	NA	70.767	1.75E-08	7.757	6.99E-08	P10 - ctrl; P3 - ctrl; P8 - ctrl; P10 - P11; P10 - P2; P3 - P10; P3 - P11; P8 - P11; P3 - P2; P8 - P2; P3 - P8
TC1300008948.hg.1	TC1300008948.hg.1	geypy.aAug10-unspliced	geypy	NA	70.688	1.76E-08	7.7542	7.02E-08	ctrl - P10; ctrl - P11; P2 - ctrl; ctrl - P3; ctrl - P8; P11 - P10; P2 - P10; P3 - P10; P2 - P11; P11 - P8; P2 - P3; P2 - P8; P3 - P8
TC1800008900.hg.1	TC1800008900.hg.1	mokori.aAug10-unspliced	mokori	NA	70.617	1.77E-08	7.7517	7.05E-08	ctrl - P10; ctrl - P11; P2 - ctrl; ctrl - P3; ctrl - P8; P2 - P10; P10 - P8; P2 - P11; P11 - P8; P2 - P3; P2 - P8; P3 - P8
TC0100018291.hg.1	TC0100018291.hg.1	NR_103778	FLG-AS1	339400	70.388	1.80E-08	7.7436	7.17E-08	ctrl - P10; ctrl - P11; P3 - ctrl; ctrl - P8; P2 - P10; P3 - P10; P8 - P10; P2 - P11; P3 - P11; P8 - P11; P3 - P2; P2 - P8; P3 - P8
TC0300007273.hg.1	TC0300007273.hg.1	NM_000316	PTH1R	5745	70.366	1.81E-08	7.7428	7.17E-08	ctrl - P10; ctrl - P11; ctrl - P2; ctrl - P3; ctrl - P8; P11 - P10; P11 - P2; P11 - P3; P11 - P8
TC0300009867.hg.1	TC0300009867.hg.1	NA	NA	NA	70.254	1.82E-08	7.7388	7.23E-08	ctrl - P10; ctrl - P11; ctrl - P2; ctrl - P3; ctrl - P8; P11 - P10; P2 - P10; P3 - P10; P10 - P8; P2 - P11; P11 - P3; P11 - P8; P2 - P3; P2 - P8; P3 - P8
TC0100015509.hg.1	TC0100015509.hg.1	NM_001100910	FAM72B	653820	70.202	1.83E-08	7.7369	7.25E-08	P10 - ctrl; ctrl - P11; ctrl - P2; P8 - ctrl; P10 - P11; P10 - P2; P10 - P3; P8 - P10; P3 - P11; P8 - P11; P3 - P2; P8 - P2; P8 - P3
TC0600012467.hg.1	TC0600012467.hg.1	ENST00000455071	LOC101928820	1.02E+08	70.159	1.84E-08	7.7354	7.26E-08	P10 - ctrl; P3 - ctrl; P8 - ctrl; P10 - P11; P10 - P2; P3 - P10; P2 - P11; P3 - P11; P8 - P11; P3 - P2; P8 - P2; P3 - P8
TC0700010718.hg.1	TC0700010718.hg.1	NR_002833	DPY19L2P1	554236	70.106	1.85E-08	7.7335	7.28E-08	ctrl - P10; ctrl - P11; ctrl - P8; P11 - P10; P2 - P10; P3 - P10; P8 - P10; P2 - P11; P3 - P11; P11 - P8; P2 - P8; P3 - P8
TC1400007120.hg.1	TC1400007120.hg.1	NM_001127713	ATL1	51062	70.027	1.86E-08	7.7307	7.31E-08	P10 - ctrl; ctrl - P11; P3 - ctrl; P8 - ctrl; P10 - P11; P10 - P2; P10 - P3; P2 - P11; P3 - P11; P8 - P11; P3 - P2; P8 - P2; P8 - P3
TC2200006827.hg.1	TC2200006827.hg.1	NM_002073	GNAZ	2781	70.025	1.86E-08	7.7306	7.31E-08	ctrl - P10; ctrl - P11; ctrl - P2; ctrl - P3; ctrl - P8; P11 - P10; P2 - P10; P11 - P3; P11 - P8; P2 - P3; P2 - P8
TC1200007741.hg.1	TC1200007741.hg.1	ENST00000562848.1	AC012531.25		70.003	1.86E-08	7.7298	7.31E-08	ctrl - P10; ctrl - P11; ctrl - P3; ctrl - P8; P11 - P10; P2 - P10; P3 - P10; P2 - P11; P11 - P8; P2 - P3; P2 - P8; P3 - P8
TC0700006923.hg.1	TC0700006923.hg.1	ENST00000428259	AC079780.3	NA	69.934	1.87E-08	7.7274	7.34E-08	ctrl - P10; ctrl - P11; ctrl - P2; ctrl - P3; ctrl - P8; P11 - P10; P2 - P10; P3 - P10; P11 - P2; P11 - P3; P11 - P8; P2 - P8; P3 - P8
TC0200012159.hg.1	TC0200012159.hg.1	NM_144575	CAPN13	92291	69.888	1.88E-08	7.7257	7.35E-08	P10 - ctrl; P8 - ctrl; P10 - P11; P10 - P2; P10 - P3; P10 - P8; P8 - P11; P8 - P2; P8 - P3
TC0200010634.hg.1	TC0200010634.hg.1	ENST00000416092	MEAF6P1		69.871	1.88E-08	7.7251	7.35E-08	ctrl - P10; ctrl - P3; ctrl - P8; P11 - P10; P2 - P10; P3 - P10; P11 - P3; P11 - P8; P2 - P3; P2 - P8; P3 - P8
TC0400010750.hg.1	TC0400010750.hg.1	NM_001292045	NMU	10874	69.824	1.89E-08	7.7234	7.36E-08	P10 - ctrl; P3 - ctrl; P8 - ctrl; P10 - P11; P10 - P2; P8 - P10; P3 - P11; P8 - P11; P3 - P2; P8 - P2; P8 - P3
TC2000007705.hg.1	TC2000007705.hg.1	blovey.aAug10-unspliced	blovey	NA	69.818	1.89E-08	7.7232	7.36E-08	ctrl - P10; ctrl - P11; ctrl - P3; ctrl - P8; P11 - P10; P2 - P10; P10 - P8; P2 - P11; P11 - P3; P11 - P8; P2 - P3; P2 - P8; P3 - P8
TC0500007000.hg.1	TC0500007000.hg.1	NR_038848	LINC01021	643401	69.806	1.89E-08	7.7228	7.36E-08	P10 - ctrl; P2 - ctrl; P3 - ctrl; P8 - ctrl; P10 - P11; P3 - P10; P2 - P11; P3 - P11; P8 - P11; P3 - P2; P8 - P2; P3 - P8
TC0200010411.hg.1	TC0200010411.hg.1	rorswaby.aAug10-unspliced	rorswaby	NA	69.781	1.90E-08	7.7219	7.36E-08	ctrl - P10; ctrl - P11; ctrl - P3; ctrl - P8; P11 - P10; P2 - P10; P3 - P10; P2 - P11; P3 - P11; P11 - P8; P2 - P3; P2 - P8; P3 - P8
TC1900011203.hg.1	TC1900011203.hg.1	ENST00000598194.1	CTD-2545M3.2		69.638	1.92E-08	7.7168	7.44E-08	ctrl - P10; P11 - ctrl; ctrl - P2; P3 - ctrl; ctrl - P8; P11 - P10; P3 - P10; P8 - P10; P11 - P2; P11 - P3; P11 - P8; P3 - P2; P3 - P8
TC2000007438.hg.1	TC2000007438.hg.1	NM_001278610	MYBL2	4605	69.413	1.96E-08	7.7087	7.55E-08	P10 - ctrl; ctrl - P11; ctrl - P2; P8 - ctrl; P10 - P11; P10 - P2; P10 - P3; P8 - P10; P3 - P11; P8 - P11; P3 - P2; P8 - P2; P8 - P3
TC1100012318.hg.1	TC1100012318.hg.1	NM_001243211	IL18	3606	69.405	1.96E-08	7.7084	7.55E-08	P10 - ctrl; P2 - ctrl; P3 - ctrl; P8 - ctrl; P10 - P11; P10 - P2; P10 - P3; P2 - P11; P3 - P11; P8 - P11; P3 - P2; P8 - P2; P8 - P3
TC0600008622.hg.1	TC0600008622.hg.1	NM_031469	SH3BGRL2	83699	69.399	1.96E-08	7.7082	7.55E-08	P10 - ctrl; P3 - ctrl; P8 - ctrl; P10 - P11; P10 - P2; P10 - P3; P3 - P11; P8 - P11; P3 - P2; P8 - P2; P8 - P3
TC0100018423.hg.1	TC0100018423.hg.1	NR_110616	LINC01355	1.01E+08	69.357	1.96E-08	7.7066	7.57E-08	ctrl - P10; ctrl - P11; P2 - ctrl; ctrl - P3; ctrl - P8; P11 - P10; P2 - P10; P3 - P10; P2 - P11; P11 - P3; P11 - P8; P2 - P3; P2 - P8
TC0100007206.hg.1	TC0100007206.hg.1	NM_001785	CDA	978	69.334	1.97E-08	7.7058	7.57E-08	P10 - ctrl; P3 - ctrl; P8 - ctrl; P10 - P11; P10 - P2; P8 - P10; P3 - P11; P8 - P11; P3 - P2; P8 - P2; P8 - P3
TC0700011842.hg.1	TC0700011842.hg.1	NM_002612	PDK4	5166	69.268	1.98E-08	7.7034	7.60E-08	P10 - ctrl; P2 - ctrl; P3 - ctrl; P8 - ctrl; P10 - P11; P10 - P2; P3 - P10; P10 - P8; P2 - P11; P3 - P11; P8 - P11; P3 - P2; P8 - P2; P3 - P8
TC0700008539.hg.1	TC0700008539.hg.1	NA	NA	NA	69.102	2.01E-08	7.6974	7.69E-08	ctrl - P10; ctrl - P11; ctrl - P2; ctrl - P3; ctrl - P8; P11 - P10; P2 - P10; P3 - P10; P2 - P11; P11 - P8; P2 - P3; P2 - P8; P3 - P8
TC1200012295.hg.1	TC1200012295.hg.1	ENST00000602474.1	RP11-214K3.22		69.064	2.01E-08	7.696	7.71E-08	ctrl - P10; ctrl - P11; P2 - ctrl; ctrl - P3; ctrl - P8; P11 - P10; P2 - P10; P3 - P10; P2 - P11; P11 - P8; P2 - P3; P2 - P8; P3 - P8
TC0300013074.hg.1	TC0300013074.hg.1	pergubo.aAug10-unspliced	pergubo	NA	68.86	2.05E-08	7.6887	7.83E-08	ctrl - P10; ctrl - P11; P2 - ctrl; ctrl - P3; ctrl - P8; P2 - P10; P10 - P8; P2 - P11; P11 - P8; P2 - P3; P2 - P8; P3 - P8
TC2000007341.hg.1	TC2000007341.hg.1	NM_030919	FAM83D	81610	68.627	2.09E-08	7.6802	7.97E-08	P10 - ctrl; ctrl - P11; ctrl - P2; P3 - ctrl; P8 - ctrl; P10 - P11; P10 - P2; P10 - P3; P8 - P10; P3 - P11; P8 - P11; P3 - P2; P8 - P2; P8 - P3
TC1000010509.hg.1	TC1000010509.hg.1	NM_001276343	AGAP4	119016	68.261	2.15E-08	7.6668	8.20E-08	ctrl - P10; ctrl - P11; P2 - ctrl; ctrl - P3; ctrl - P8; P11 - P10; P2 - P10; P3 - P10; P2 - P11; P11 - P8; P2 - P3; P2 - P8; P3 - P8

TC0100015706.hg.1	TC0100015706.hg.1	uc057klt.1	AC239868.2	NA	68.011	2.20E-08	7.6577	8.37E-08	P10 - ctrl; P11 - ctrl; P8 - ctrl; P10 - P11; P10 - P2; P10 - P3; P10 - P8; P11 - P2; P11 - P3; P8 - P11; P3 - P2; P8 - P2; P8 - P3
TC1100012688.hg.1	TC1100012688.hg.1	NA	NA	NA	67.994	2.20E-08	7.657	8.37E-08	ctrl - P10; ctrl - P11; ctrl - P3; ctrl - P8; P11 - P10; P2 - P10; P3 - P10; P10 - P8; P2 - P11; P11 - P8; P2 - P3; P2 - P8; P3 - P8
TC0X00010737.hg.1	TC0X00010737.hg.1	ENST00000625970	SMAD5-AS1_2		67.952	2.21E-08	7.6555	8.38E-08	ctrl - P10; ctrl - P2; ctrl - P3; ctrl - P8; P11 - P10; P2 - P10; P3 - P10; P11 - P2; P11 - P3; P11 - P8; P2 - P8; P3 - P8
TC0700009134.hg.1	TC0700009134.hg.1	NM_001163446	CPA4	51200	67.835	2.23E-08	7.6512	8.45E-08	P3 - ctrl; P8 - ctrl; P10 - P11; P10 - P2; P3 - P10; P8 - P10; P3 - P11; P8 - P11; P3 - P2; P8 - P2; P3 - P8
TC0100010921.hg.1	TC0100010921.hg.1	NM_031935	HMCN1	83872	67.734	2.25E-08	7.6475	8.51E-08	ctrl - P10; ctrl - P11; ctrl - P2; ctrl - P3; ctrl - P8; P11 - P10; P2 - P10; P10 - P8; P2 - P11; P11 - P3; P11 - P8; P2 - P3; P2 - P8; P3 - P8
TC1500006570.hg.1	TC1500006570.hg.1	ENST00000604451.1	RP11-701H24.7		67.666	2.26E-08	7.6449	8.55E-08	ctrl - P10; ctrl - P11; ctrl - P3; ctrl - P8; P11 - P10; P2 - P10; P3 - P10; P2 - P11; P11 - P3; P11 - P8; P2 - P3; P2 - P8; P3 - P8
TC0200016557.hg.1	TC0200016557.hg.1	OTTHUMT00000480993	AC013463.2		67.586	2.28E-08	7.642	8.59E-08	ctrl - P10; P11 - ctrl; ctrl - P3; ctrl - P8; P11 - P10; P2 - P10; P11 - P2; P11 - P3; P11 - P8; P2 - P3; P2 - P8
TC0600011517.hg.1	TC0600011517.hg.1	NM_001242525	HLA-DPA1	3113	67.514	2.29E-08	7.6393	8.64E-08	P10 - ctrl; P8 - ctrl; P10 - P11; P10 - P2; P10 - P3; P8 - P10; P8 - P11; P8 - P2; P8 - P3
TC1200007594.hg.1	TC1200007594.hg.1	NM_001095	ASIC1	41	67.46	2.30E-08	7.6374	8.66E-08	P10 - ctrl; P3 - ctrl; P8 - ctrl; P10 - P11; P10 - P2; P8 - P10; P3 - P11; P8 - P11; P3 - P2; P8 - P2
TC0100016359.hg.1	TC0100016359.hg.1	NM_000655	SELL	6402	67.39	2.32E-08	7.6348	8.70E-08	P10 - ctrl; P3 - ctrl; P8 - ctrl; P10 - P11; P10 - P2; P8 - P10; P3 - P11; P8 - P11; P3 - P2; P8 - P2; P8 - P3
TC1700010625.hg.1	TC1700010625.hg.1	NM_032865	TNS4	84951	67.155	2.37E-08	7.626	8.77E-08	P10 - ctrl; P3 - ctrl; P8 - ctrl; P10 - P11; P10 - P2; P3 - P10; P8 - P10; P3 - P11; P8 - P11; P3 - P2; P8 - P2; P3 - P8
TC0100017626.hg.1	TC0100017626.hg.1	NR_023364	RNA5S2	1E+08	67.099	2.38E-08	7.624	8.77E-08	P10 - ctrl; P11 - ctrl; P2 - ctrl; P3 - ctrl; P8 - ctrl; P11 - P10; P2 - P10; P3 - P10; P8 - P10; P2 - P11; P3 - P11; P3 - P2; P2 - P8; P3 - P8
TC0100017627.hg.1	TC0100017627.hg.1	NR_023365	RNA5S3	1E+08	67.099	2.38E-08	7.624	8.77E-08	P10 - ctrl; P11 - ctrl; P2 - ctrl; P3 - ctrl; P8 - ctrl; P11 - P10; P2 - P10; P3 - P10; P8 - P10; P2 - P11; P3 - P11; P3 - P2; P2 - P8; P3 - P8
TC0100017628.hg.1	TC0100017628.hg.1	NR_023366	RNA5S4	1E+08	67.099	2.38E-08	7.624	8.77E-08	P10 - ctrl; P11 - ctrl; P2 - ctrl; P3 - ctrl; P8 - ctrl; P11 - P10; P2 - P10; P3 - P10; P8 - P10; P2 - P11; P3 - P11; P3 - P2; P2 - P8; P3 - P8
TC0100017629.hg.1	TC0100017629.hg.1	NR_023363	RNA5S1	1E+08	67.099	2.38E-08	7.624	8.77E-08	P10 - ctrl; P11 - ctrl; P2 - ctrl; P3 - ctrl; P8 - ctrl; P11 - P10; P2 - P10; P3 - P10; P8 - P10; P2 - P11; P3 - P11; P3 - P2; P2 - P8; P3 - P8
TC0100017630.hg.1	TC0100017630.hg.1	NR_023368	RNA5S6	1E+08	67.099	2.38E-08	7.624	8.77E-08	P10 - ctrl; P11 - ctrl; P2 - ctrl; P3 - ctrl; P8 - ctrl; P11 - P10; P2 - P10; P3 - P10; P8 - P10; P2 - P11; P3 - P11; P3 - P2; P2 - P8; P3 - P8
TC0100017631.hg.1	TC0100017631.hg.1	NR_023373	RNA5S11	1E+08	67.099	2.38E-08	7.624	8.77E-08	P10 - ctrl; P11 - ctrl; P2 - ctrl; P3 - ctrl; P8 - ctrl; P11 - P10; P2 - P10; P3 - P10; P8 - P10; P2 - P11; P3 - P11; P3 - P2; P2 - P8; P3 - P8
TC0100017632.hg.1	TC0100017632.hg.1	ENST00000365651	RNA5S7	1E+08	67.099	2.38E-08	7.624	8.77E-08	P10 - ctrl; P11 - ctrl; P2 - ctrl; P3 - ctrl; P8 - ctrl; P11 - P10; P2 - P10; P3 - P10; P8 - P10; P2 - P11; P3 - P11; P3 - P2; P2 - P8; P3 - P8
TC0100017633.hg.1	TC0100017633.hg.1	NR_023372	RNA5S10	1E+08	67.099	2.38E-08	7.624	8.77E-08	P10 - ctrl; P11 - ctrl; P2 - ctrl; P3 - ctrl; P8 - ctrl; P11 - P10; P2 - P10; P3 - P10; P8 - P10; P2 - P11; P3 - P11; P3 - P2; P2 - P8; P3 - P8
TC0100017635.hg.1	TC0100017635.hg.1	ENST00000363040	RNA5S10	1E+08	67.099	2.38E-08	7.624	8.77E-08	P10 - ctrl; P11 - ctrl; P2 - ctrl; P3 - ctrl; P8 - ctrl; P11 - P10; P2 - P10; P3 - P10; P8 - P10; P2 - P11; P3 - P11; P3 - P2; P2 - P8; P3 - P8
TC0100017642.hg.1	TC0100017642.hg.1	ENST00000363500	RNA5S17	1E+08	67.099	2.38E-08	7.624	8.77E-08	P10 - ctrl; P11 - ctrl; P2 - ctrl; P3 - ctrl; P8 - ctrl; P11 - P10; P2 - P10; P3 - P10; P8 - P10; P2 - P11; P3 - P11; P3 - P2; P2 - P8; P3 - P8
TC0X00008084.hg.1	TC0X00008084.hg.1	NM_000495	COL4A5	1287	66.901	2.42E-08	7.6166	8.90E-08	ctrl - P10; ctrl - P11; ctrl - P2; ctrl - P3; ctrl - P8; P11 - P10; P2 - P10; P3 - P10; P11 - P2; P11 - P3; P11 - P8; P2 - P3; P2 - P8; P3 - P8
TC0500010822.hg.1	TC0500010822.hg.1	NM_001017992	ACTBL2	345651	66.892	2.42E-08	7.6162	8.90E-08	P10 - ctrl; P3 - ctrl; P8 - ctrl; P10 - P11; P10 - P2; P3 - P11; P8 - P11; P3 - P2; P8 - P2; P3 - P8
TC1900007609.hg.1	TC1900007609.hg.1	NA	NA	NA	66.879	2.42E-08	7.6158	8.90E-08	ctrl - P10; ctrl - P11; ctrl - P2; ctrl - P8; P3 - P10; P8 - P10; P2 - P11; P3 - P11; P8 - P11; P3 - P2; P8 - P2; P3 - P8
TC0X00008760.hg.1	TC0X00008760.hg.1	NM_001178106	ZNF185	7739	66.845	2.43E-08	7.6145	8.92E-08	P10 - ctrl; P3 - ctrl; P8 - ctrl; P10 - P11; P10 - P2; P8 - P10; P3 - P11; P8 - P11; P3 - P2; P8 - P2
TC0200015529.hg.1	TC0200015529.hg.1	NM_001039845	MDH1B	130752	66.703	2.46E-08	7.6092	9.01E-08	ctrl - P10; ctrl - P11; ctrl - P2; ctrl - P3; ctrl - P8; P11 - P10; P2 - P10; P11 - P3; P11 - P8; P2 - P3; P2 - P8
TC0100007885.hg.1	TC0100007885.hg.1	stokloy.aAug10-unspliced	stokloy	NA	66.661	2.47E-08	7.6076	9.03E-08	ctrl - P10; ctrl - P11; ctrl - P3; ctrl - P8; P11 - P10; P2 - P10; P10 - P8; P2 - P11; P11 - P3; P11 - P8; P2 - P3; P2 - P8; P3 - P8
TC0200009681.hg.1	TC0200009681.hg.1	plufubu.aAug10-unspliced	plufubu	NA	66.636	2.47E-08	7.6067	9.04E-08	ctrl - P10; ctrl - P11; P2 - ctrl; ctrl - P3; ctrl - P8; P10 - P11; P2 - P10; P10 - P8; P2 - P11; P3 - P11; P11 - P8; P2 - P3; P2 - P8; P3 - P8
TC0600010078.hg.1	TC0600010078.hg.1	NM_021977	SLC22A3	6581	66.325	2.54E-08	7.595	9.27E-08	ctrl - P10; P11 - ctrl; ctrl - P2; ctrl - P8; P11 - P10; P2 - P10; P3 - P10; P11 - P2; P11 - P3; P11 - P8; P3 - P2; P2 - P8; P3 - P8
TC0900007845.hg.1	TC0900007845.hg.1	plawtawbo.aAug10-unspliced	plawtawbo	NA	66.199	2.57E-08	7.5902	9.36E-08	ctrl - P10; ctrl - P11; P2 - ctrl; ctrl - P3; ctrl - P8; P2 - P10; P3 - P10; P2 - P11; P3 - P11; P11 - P8; P2 - P3; P2 - P8; P3 - P8
TC0400008030.hg.1	TC0400008030.hg.1	NM_001025616	ARHGAP24	83478	66.164	2.58E-08	7.5889	9.36E-08	ctrl - P10; ctrl - P2; ctrl - P3; ctrl - P8; P11 - P10; P11 - P2; P11 - P3; P11 - P8
TC0500006472.hg.1	TC0500006472.hg.1	NM_001166260	TRIP13	9319	66.151	2.58E-08	7.5884	9.36E-08	P10 - ctrl; ctrl - P11; ctrl - P2; P3 - ctrl; P8 - ctrl; P10 - P11; P10 - P2; P10 - P3; P8 - P10; P3 - P11; P8 - P11; P3 - P2; P8 - P2; P8 - P3
TC0Y00007306.hg.1	TC0Y00007306.hg.1	NA	NA	NA	66.142	2.58E-08	7.5881	9.36E-08	ctrl - P10; ctrl - P11; ctrl - P2; ctrl - P3; ctrl - P8; P11 - P10; P3 - P10; P8 - P10; P11 - P2; P3 - P11; P3 - P2; P8 - P2; P3 - P8

TC0200008534.hg.1	TC0200008534.hg.1	NM_001164315	ANKRD36	375248	66.115	2.59E-08	7.5871	9.37E-08	ctrl - P10; ctrl - P11; ctrl - P3; ctrl - P8; P2 - P10; P3 - P10; P10 - P8; P2 - P11; P3 - P11; P11 - P8; P2 - P3; P2 - P8; P3 - P8
TC1600011363.hg.1	TC1600011363.hg.1	NR_027154	SMG1P1	641298	65.89	2.64E-08	7.5786	9.54E-08	ctrl - P10; ctrl - P11; ctrl - P3; ctrl - P8; P2 - P10; P10 - P3; P10 - P8; P2 - P11; P11 - P3; P11 - P8; P2 - P3; P2 - P8
TC0500011031.hg.1	TC0500011031.hg.1	NA	NA	NA	65.813	2.66E-08	7.5757	9.59E-08	ctrl - P10; ctrl - P11; ctrl - P3; ctrl - P8; P11 - P10; P2 - P10; P3 - P10; P2 - P11; P11 - P3; P11 - P8; P2 - P3; P2 - P8; P3 - P8
TC1100011184.hg.1	TC1100011184.hg.1	hsa_circ_0000321	SF1	NA	65.74	2.67E-08	7.5729	9.64E-08	ctrl - P10; ctrl - P11; ctrl - P3; ctrl - P8; P11 - P10; P2 - P10; P3 - P10; P10 - P8; P2 - P11; P11 - P8; P2 - P3; P2 - P8; P3 - P8
TC0600007655.hg.1	TC0600007655.hg.1	NA	NA	NA	65.673	2.69E-08	7.5704	9.68E-08	P10 - ctrl; P8 - ctrl; P10 - P11; P10 - P2; P10 - P3; P8 - P11; P8 - P2; P8 - P3
TC1800008901.hg.1	TC1800008901.hg.1	ENST00000588307.1	RP11-28F1.2		65.633	2.70E-08	7.5688	9.70E-08	ctrl - P10; ctrl - P11; P2 - ctrl; ctrl - P3; ctrl - P8; P2 - P10; P10 - P3; P10 - P8; P2 - P11; P11 - P3; P11 - P8; P2 - P3; P2 - P8
TC1100009291.hg.1	TC1100009291.hg.1	kesimi.bAug10-unspliced	kesimi	NA	65.61	2.70E-08	7.5679	9.71E-08	ctrl - P10; ctrl - P11; P2 - ctrl; ctrl - P8; P11 - P10; P2 - P10; P3 - P10; P2 - P11; P3 - P11; P11 - P8; P2 - P3; P2 - P8; P3 - P8
TC2000009550.hg.1	TC2000009550.hg.1	NM_003600	AURKA	6790	65.571	2.71E-08	7.5665	9.73E-08	P10 - ctrl; ctrl - P11; ctrl - P2; P8 - ctrl; P10 - P11; P10 - P2; P10 - P3; P8 - P10; P3 - P11; P8 - P11; P3 - P2; P8 - P2; P8 - P3
TC0700009079.hg.1	TC0700009079.hg.1	NM_178562	TSPAN33	340348	65.518	2.73E-08	7.5645	9.76E-08	ctrl - P10; P11 - ctrl; ctrl - P2; ctrl - P3; ctrl - P8; P11 - P10; P11 - P2; P11 - P3; P11 - P8; P3 - P2
TC0800007065.hg.1	TC0800007065.hg.1	NM_152562	CDCA2	157313	65.327	2.77E-08	7.5572	9.91E-08	P10 - ctrl; P3 - ctrl; P8 - ctrl; P10 - P11; P10 - P2; P10 - P3; P8 - P10; P3 - P11; P8 - P11; P3 - P2; P8 - P2; P8 - P3
TC0400011018.hg.1	TC0400011018.hg.1	NM_002089	CXCL2	2920	65.261	2.79E-08	7.5546	9.95E-08	P10 - ctrl; ctrl - P2; P3 - ctrl; P8 - ctrl; P10 - P11; P10 - P2; P10 - P3; P3 - P11; P8 - P11; P3 - P2; P8 - P2; P8 - P3
TC1100009760.hg.1	TC1100009760.hg.1	spaforby.aAug10-unspliced	spaforby	NA	65.252	2.79E-08	7.5543	9.95E-08	ctrl - P10; ctrl - P11; ctrl - P2; ctrl - P3; ctrl - P8; P3 - P10; P3 - P11; P11 - P8; P3 - P2; P3 - P8
TC0600010237.hg.1	TC0600010237.hg.1	toysmerby.aAug10-unspliced	toysmerby	NA	65.169	2.81E-08	7.5511	1.00E-07	ctrl - P10; ctrl - P11; ctrl - P3; ctrl - P8; P11 - P10; P2 - P10; P10 - P8; P2 - P11; P11 - P8; P2 - P3; P2 - P8; P3 - P8
TC0200008099.hg.1	TC0200008099.hg.1	NM_000189	HK2	3099	65.164	2.81E-08	7.5509	1.00E-07	P10 - ctrl; P11 - ctrl; P3 - ctrl; P8 - ctrl; P10 - P2; P10 - P3; P11 - P2; P11 - P3; P11 - P8; P3 - P2; P8 - P2
TC0200015264.hg.1	TC0200015264.hg.1	NM_004657	SDPR	8436	65.113	2.83E-08	7.549	1.00E-07	ctrl - P10; P11 - ctrl; ctrl - P2; ctrl - P3; ctrl - P8; P11 - P10; P3 - P10; P8 - P10; P11 - P2; P11 - P3; P11 - P8; P3 - P2; P8 - P2; P3 - P8
TC1600007999.hg.1	TC1600007999.hg.1	NM_001304392	CX3CL1	6376	65.042	2.84E-08	7.5463	1.01E-07	P10 - ctrl; P11 - ctrl; ctrl - P2; P3 - ctrl; P8 - ctrl; P10 - P11; P10 - P2; P10 - P3; P11 - P2; P8 - P11; P3 - P2; P8 - P2; P8 - P3
TC1900009076.hg.1	TC1900009076.hg.1	NM_003712	PLPP2	8612	65.035	2.84E-08	7.546	1.01E-07	P10 - ctrl; P11 - ctrl; P3 - ctrl; P8 - ctrl; P10 - P11; P10 - P2; P10 - P3; P11 - P2; P8 - P11; P3 - P2; P8 - P2; P8 - P3
TC0400012147.hg.1	TC0400012147.hg.1	feefloy.aAug10-unspliced	feefloy	NA	64.93	2.87E-08	7.542	1.01E-07	ctrl - P10; ctrl - P11; P2 - ctrl; ctrl - P3; ctrl - P8; P2 - P10; P3 - P10; P2 - P11; P3 - P11; P11 - P8; P2 - P3; P2 - P8; P3 - P8
TC0700010536.hg.1	TC0700010536.hg.1	ENST00000608362.1	CTB-119C2.1		64.907	2.88E-08	7.5411	1.02E-07	ctrl - P10; ctrl - P11; ctrl - P2; ctrl - P3; ctrl - P8; P11 - P10; P2 - P10; P3 - P10; P2 - P11; P11 - P8; P2 - P3; P2 - P8; P3 - P8
TC0500008833.hg.1	TC0500008833.hg.1	cherskorby.aAug10-unspliced	cherskorby	NA	64.685	2.93E-08	7.5325	1.03E-07	ctrl - P10; ctrl - P11; P2 - ctrl; ctrl - P3; ctrl - P8; P10 - P11; P2 - P10; P10 - P8; P2 - P11; P3 - P11; P2 - P3; P2 - P8; P3 - P8
TC0300013698.hg.1	TC0300013698.hg.1	rorverbo.aAug10-unspliced	rorverbo	NA	64.573	2.96E-08	7.5282	1.04E-07	ctrl - P10; ctrl - P11; P2 - ctrl; ctrl - P3; ctrl - P8; P2 - P10; P10 - P8; P2 - P11; P11 - P8; P2 - P3; P2 - P8; P3 - P8
TC0700008184.hg.1	TC0700008184.hg.1	sneeko.aAug10-unspliced	sneeko	NA	64.569	2.96E-08	7.5281	1.04E-07	ctrl - P10; ctrl - P11; ctrl - P3; ctrl - P8; P11 - P10; P2 - P10; P3 - P10; P10 - P8; P2 - P11; P11 - P8; P2 - P3; P2 - P8; P3 - P8
TC1400007584.hg.1	TC1400007584.hg.1	NM_015351	TTC9	23508	64.504	2.98E-08	7.5256	1.05E-07	P10 - ctrl; ctrl - P2; P3 - ctrl; P8 - ctrl; P10 - P11; P10 - P2; P11 - P2; P3 - P11; P8 - P11; P3 - P2; P8 - P2
TC1100012966.hg.1	TC1100012966.hg.1	NM_001282663	MICAL2	9645	64.297	3.04E-08	7.5176	1.06E-07	P10 - ctrl; P3 - ctrl; P8 - ctrl; P10 - P11; P10 - P2; P10 - P3; P8 - P10; P3 - P11; P8 - P11; P3 - P2; P8 - P2; P8 - P3
TC0600008630.hg.1	TC0600008630.hg.1	NM_001166691	TTK	7272	64.224	3.06E-08	7.5147	1.07E-07	P10 - ctrl; ctrl - P11; ctrl - P2; P8 - ctrl; P10 - P11; P10 - P2; P10 - P3; P8 - P10; P3 - P11; P8 - P11; P3 - P2; P8 - P2; P8 - P3
TC1100007510.hg.1	TC1100007510.hg.1	keeny.aAug10-unspliced	keeny	NA	64.222	3.06E-08	7.5146	1.07E-07	ctrl - P10; ctrl - P11; ctrl - P8; P2 - P10; P3 - P10; P2 - P11; P3 - P11; P11 - P8; P2 - P8; P3 - P8
TC0700006813.hg.1	TC0700006813.hg.1	NM_001204144	HDAC9	9734	64.186	3.07E-08	7.5132	1.07E-07	P10 - ctrl; P3 - ctrl; P8 - ctrl; P10 - P11; P10 - P2; P10 - P3; P2 - P11; P3 - P11; P8 - P11; P3 - P2; P8 - P2; P8 - P3
TC0100009908.hg.1	TC0100009908.hg.1	NM_001202858	ECM1	1893	64.109	3.09E-08	7.5103	1.08E-07	P10 - ctrl; P3 - ctrl; P8 - ctrl; P10 - P11; P10 - P2; P8 - P10; P3 - P11; P8 - P11; P3 - P2; P8 - P2; P8 - P3
TC1500010699.hg.1	TC1500010699.hg.1	NA	NA	NA	63.927	3.14E-08	7.5032	1.09E-07	ctrl - P10; ctrl - P11; P2 - ctrl; ctrl - P3; ctrl - P8; P11 - P10; P2 - P10; P3 - P10; P2 - P11; P11 - P8; P2 - P3; P2 - P8; P3 - P8
TC0200009095.hg.1	TC0200009095.hg.1	NM_002193	INHBB	3625	63.765	3.19E-08	7.4968	1.11E-07	P10 - ctrl; ctrl - P11; ctrl - P2; P3 - ctrl; P8 - ctrl; P10 - P11; P10 - P2; P3 - P11; P8 - P11; P3 - P2; P8 - P2; P8 - P3
TC1500010368.hg.1	TC1500010368.hg.1	NM_001307952	HAPLN3	145864	63.66	3.22E-08	7.4927	1.12E-07	P10 - ctrl; P3 - ctrl; P8 - ctrl; P10 - P11; P10 - P2; P10 - P3; P8 - P10; P3 - P11; P8 - P11; P3 - P2; P8 - P2; P8 - P3
TC2200007894.hg.1	TC2200007894.hg.1	NM_001282225	CECR1	51816	63.55	3.25E-08	7.4884	1.13E-07	ctrl - P10; ctrl - P2; ctrl - P3; ctrl - P8; P11 - P10; P10 - P3; P10 - P8; P11 - P2; P11 - P3; P11 - P8; P2 - P3; P2 - P8
TC0500011225.hg.1	TC0500011225.hg.1	NM_005779	LHFPL2	10184	63.437	3.28E-08	7.484	1.14E-07	P10 - ctrl; P11 - ctrl; P3 - ctrl; P8 - ctrl; P10 - P11; P10 - P2; P8 - P10; P3 - P11; P8 - P11; P3 - P2; P8 - P2; P8 - P3
TC1500006592.hg.1	TC1500006592.hg.1	NR_003307	SNORD115-15	15q11.2	63.347	3.31E-08	7.4805	1.14E-07	P10 - ctrl; P11 - ctrl; P2 - ctrl; P3 - ctrl; P8 - ctrl; P10 - P11; P10 - P2; P3 - P10; P3 - P11; P8 - P11; P3 - P2; P8 - P2; P3 - P8

TC0300010135.hg.1	TC0300010135.hg.1	NR_004428	EGOT	1E+08	63.319	3.32E-08	7.4793	1.14E-07	ctrl - P10; ctrl - P11; ctrl - P2; ctrl - P3; ctrl - P8; P11 - P10; P2 - P10; P3 - P10; P11 - P2; P11 - P3; P11 - P8; P2 - P8
TC0600007596.hg.1	TC0600007596.hg.1	ENST00000449264	TNF	7124	63.191	3.35E-08	7.4743	1.16E-07	P10 - ctrl; ctrl - P2; P3 - ctrl; P8 - ctrl; P10 - P11; P10 - P2; P8 - P10; P11 - P2; P3 - P11; P8 - P11; P3 - P2; P8 - P2; P8 - P3
TC0900010219.hg.1	TC0900010219.hg.1	NR_002817	AQP7P1	375719	63.066	3.39E-08	7.4694	1.17E-07	P10 - ctrl; P2 - ctrl; P3 - ctrl; P8 - ctrl; P10 - P11; P3 - P10; P8 - P10; P2 - P11; P3 - P11; P8 - P11; P3 - P2; P8 - P2; P3 - P8
TC0500013385.hg.1	TC0500013385.hg.1	NM_024577	SH3TC2	79628	63.062	3.39E-08	7.4692	1.17E-07	P10 - ctrl; P3 - ctrl; P8 - ctrl; P10 - P11; P10 - P2; P3 - P10; P8 - P10; P3 - P11; P8 - P11; P3 - P2; P8 - P2; P3 - P8
TC0700010543.hg.1	TC0700010543.hg.1	ENST00000451264.1	AC004540.4		62.691	3.51E-08	7.4546	1.21E-07	ctrl - P10; ctrl - P2; ctrl - P3; ctrl - P8; P11 - P10; P2 - P10; P11 - P2; P11 - P3; P11 - P8; P2 - P3
TC0100017167.hg.1	TC0100017167.hg.1	NM_000228	LAMB3	3914	62.455	3.59E-08	7.4452	1.23E-07	P10 - ctrl; ctrl - P2; P3 - ctrl; P8 - ctrl; P10 - P11; P10 - P2; P10 - P3; P11 - P2; P3 - P11; P8 - P11; P3 - P2; P8 - P2; P8 - P3
TC1300009674.hg.1	TC1300009674.hg.1	NM_024089	KDELC1	79070	62.24	3.66E-08	7.4366	1.25E-07	ctrl - P10; ctrl - P11; ctrl - P2; ctrl - P3; ctrl - P8; P11 - P10; P2 - P10; P3 - P10; P8 - P10; P11 - P2; P11 - P3; P11 - P8; P2 - P8; P3 - P8
TC0100007102.hg.1	TC0100007102.hg.1	NM_013358	PADI1	29943	62.146	3.69E-08	7.4328	1.26E-07	P10 - ctrl; P3 - ctrl; P8 - ctrl; P10 - P11; P10 - P2; P8 - P10; P3 - P11; P8 - P11; P3 - P2; P8 - P2
TC0800006887.hg.1	TC0800006887.hg.1	NM_000662	NAT1	9	62.046	3.73E-08	7.4288	1.27E-07	P10 - ctrl; P3 - ctrl; P8 - ctrl; P10 - P11; P10 - P2; P10 - P3; P3 - P11; P8 - P11; P3 - P2; P8 - P2; P8 - P3
TC0500012312.hg.1	TC0500012312.hg.1	NM_001127496	SPRY4	81848	62.032	3.73E-08	7.4283	1.27E-07	P10 - ctrl; P11 - ctrl; P3 - ctrl; P8 - ctrl; P10 - P11; P10 - P2; P11 - P2; P3 - P11; P8 - P11; P3 - P2; P8 - P2
TC0600007375.hg.1	TC0600007375.hg.1	NM_003536	HIST1H3H	8357	61.986	3.75E-08	7.4264	1.28E-07	P10 - ctrl; ctrl - P2; P3 - ctrl; P8 - ctrl; P10 - P11; P10 - P2; P10 - P3; P11 - P2; P3 - P11; P8 - P11; P3 - P2; P8 - P2; P8 - P3
TC0500007379.hg.1	TC0500007379.hg.1	uc063dny.1	AC024569.1	NA	61.88	3.78E-08	7.4222	1.29E-07	ctrl - P10; ctrl - P11; P2 - ctrl; ctrl - P3; ctrl - P8; P2 - P10; P3 - P10; P10 - P8; P2 - P11; P11 - P8; P2 - P3; P2 - P8; P3 - P8
TC0800011909.hg.1	TC0800011909.hg.1	ENST00000623679	AC083843.4		61.87	3.79E-08	7.4217	1.29E-07	ctrl - P10; ctrl - P11; P2 - ctrl; ctrl - P3; ctrl - P8; P11 - P10; P2 - P10; P10 - P8; P2 - P11; P11 - P3; P11 - P8; P2 - P3; P2 - P8; P3 - P8
TC0900008054.hg.1	TC0900008054.hg.1	NA	NA	NA	61.758	3.83E-08	7.4172	1.30E-07	ctrl - P10; ctrl - P11; P2 - ctrl; ctrl - P3; ctrl - P8; P2 - P10; P3 - P10; P10 - P8; P2 - P11; P11 - P8; P2 - P3; P2 - P8; P3 - P8
TC1200012708.hg.1	TC1200012708.hg.1	NM_001032409	OAS1	4938	61.68	3.85E-08	7.4141	1.31E-07	P10 - ctrl; ctrl - P2; P3 - ctrl; P8 - ctrl; P10 - P11; P10 - P2; P3 - P10; P8 - P10; P11 - P2; P3 - P11; P8 - P11; P3 - P2; P8 - P2
TC0600006762.hg.1	TC0600006762.hg.1	NR_106988_7	MIR7641-2		61.644	3.87E-08	7.4126	1.31E-07	P10 - ctrl; P11 - ctrl; P2 - ctrl; P3 - ctrl; P8 - ctrl; P11 - P10; P2 - P10; P3 - P10; P8 - P10; P2 - P11; P3 - P11; P3 - P2; P2 - P8; P3 - P8
TC0400009258.hg.1	TC0400009258.hg.1	NM_001166108	PALLD	23022	61.608	3.88E-08	7.4112	1.31E-07	P10 - ctrl; ctrl - P11; P8 - ctrl; P10 - P11; P10 - P2; P10 - P3; P2 - P11; P3 - P11; P8 - P11; P3 - P2; P8 - P2; P8 - P3
TC1200011251.hg.1	TC1200011251.hg.1	NM_004616	TSPAN8	7103	61.31	3.99E-08	7.3991	1.35E-07	P10 - ctrl; ctrl - P2; P3 - ctrl; P8 - ctrl; P10 - P11; P10 - P2; P3 - P11; P8 - P11; P3 - P2; P8 - P2
TC0800011148.hg.1	TC0800011148.hg.1	NM_057749	CCNE2	9134	61.256	4.01E-08	7.3969	1.35E-07	P10 - ctrl; ctrl - P11; P3 - ctrl; P8 - ctrl; P10 - P11; P10 - P2; P3 - P10; P8 - P10; P2 - P11; P3 - P11; P8 - P11; P3 - P2; P8 - P2
TC0100009307.hg.1	TC0100009307.hg.1	NM_001113226	NTNG1	22854	61.206	4.03E-08	7.3949	1.35E-07	ctrl - P10; P11 - ctrl; ctrl - P2; P3 - ctrl; P11 - P10; P2 - P10; P3 - P10; P8 - P10; P11 - P2; P11 - P8; P3 - P2; P3 - P8
TC2000009887.hg.1	TC2000009887.hg.1	NM_015192	PLCB1	23236	61.07	4.08E-08	7.3894	1.37E-07	ctrl - P10; P11 - ctrl; ctrl - P2; ctrl - P3; ctrl - P8; P11 - P10; P2 - P10; P11 - P2; P11 - P3; P11 - P8; P2 - P3; P2 - P8
TC0800008512.hg.1	TC0800008512.hg.1	glydarby.aAug10-unspliced	glydarby	NA	61.057	4.08E-08	7.3888	1.37E-07	ctrl - P10; ctrl - P11; P2 - ctrl; ctrl - P3; ctrl - P8; P2 - P10; P3 - P10; P2 - P11; P3 - P11; P2 - P3; P2 - P8; P3 - P8
TC0200014704.hg.1	TC0200014704.hg.1	ENST00000463588	RN7SL393P		61.018	4.10E-08	7.3872	1.37E-07	ctrl - P10; ctrl - P11; ctrl - P3; ctrl - P8; P11 - P10; P2 - P10; P10 - P8; P2 - P11; P11 - P3; P11 - P8; P2 - P3; P2 - P8; P3 - P8
TC1500007160.hg.1	TC1500007160.hg.1	NA	NA	NA	61.006	4.10E-08	7.3868	1.37E-07	ctrl - P10; ctrl - P2; ctrl - P3; ctrl - P8; P11 - P10; P10 - P8; P11 - P2; P11 - P3; P11 - P8; P2 - P3; P2 - P8
TC2100007424.hg.1	TC2100007424.hg.1	NA	NA	NA	60.982	4.11E-08	7.3858	1.37E-07	ctrl - P10; ctrl - P11; ctrl - P2; ctrl - P3; ctrl - P8; P2 - P10; P3 - P10; P2 - P11; P3 - P11; P2 - P3; P2 - P8; P3 - P8
TC0700012140.hg.1	TC0700012140.hg.1	NR_027374	LHFPL3-AS2	723809	60.964	4.12E-08	7.3851	1.37E-07	ctrl - P10; P11 - ctrl; P3 - ctrl; ctrl - P8; P11 - P10; P2 - P10; P3 - P10; P11 - P2; P11 - P3; P11 - P8; P3 - P2; P2 - P8; P3 - P8
TC1200011959.hg.1	TC1200011959.hg.1	NA	NA	NA	60.892	4.15E-08	7.3821	1.38E-07	ctrl - P10; ctrl - P11; ctrl - P3; ctrl - P8; P11 - P10; P2 - P10; P3 - P10; P2 - P11; P11 - P3; P11 - P8; P2 - P3; P2 - P8; P3 - P8
TC0100015550.hg.1	TC0100015550.hg.1	NM_001287385	FAM72C	554282	60.879	4.15E-08	7.3816	1.38E-07	P10 - ctrl; ctrl - P11; P8 - ctrl; P10 - P11; P10 - P2; P10 - P3; P8 - P10; P3 - P11; P8 - P11; P3 - P2; P8 - P2; P8 - P3
TC0100017634.hg.1	TC0100017634.hg.1	NR_023371	RNA5S9	1E+08	60.786	4.19E-08	7.3778	1.39E-07	P10 - ctrl; P11 - ctrl; P2 - ctrl; P3 - ctrl; P8 - ctrl; P11 - P10; P2 - P10; P3 - P10; P8 - P10; P2 - P11; P3 - P11; P3 - P2; P2 - P8; P3 - P8
TC0200014088.hg.1	TC0200014088.hg.1	ronemo.aAug10-unspliced	ronemo	NA	60.764	4.20E-08	7.3769	1.39E-07	ctrl - P10; ctrl - P11; ctrl - P2; ctrl - P3; ctrl - P8; P11 - P10; P2 - P10; P3 - P10; P2 - P11; P3 - P11; P11 - P8; P2 - P3; P2 - P8; P3 - P8
TC1600006949.hg.1	TC1600006949.hg.1	ENST00000561528.1	RP11-165M1.2		60.725	4.21E-08	7.3753	1.40E-07	ctrl - P10; P2 - ctrl; ctrl - P3; ctrl - P8; P11 - P10; P2 - P10; P3 - P10; P2 - P11; P11 - P3; P11 - P8; P2 - P3; P2 - P8; P3 - P8
TC1200007352.hg.1	TC1200007352.hg.1	NM_198578	LRRK2	120892	60.656	4.24E-08	7.3725	1.40E-07	ctrl - P10; ctrl - P3; ctrl - P8; P11 - P10; P2 - P10; P3 - P10; P10 - P8; P11 - P8; P2 - P3; P2 - P8; P3 - P8
TC1600011496.hg.1	TC1600011496.hg.1	NM_001304771	GPRC5B	51704	60.382	4.35E-08	7.3612	1.44E-07	P10 - ctrl; P11 - ctrl; ctrl - P2; ctrl - P3; P8 - ctrl; P11 - P10; P10 - P2; P10 - P3; P11 - P2; P11 - P3; P8 - P2; P8 - P3

TC1700007216.hg.1	TC1700007216.hg.1	NR_023380	CCDC144CP	348254	60.303	4.39E-08	7.358	1.45E-07	ctrl - P10; ctrl - P11; ctrl - P3; ctrl - P8; P11 - P10; P2 - P10; P3 - P10; P2 - P11; P11 - P3; P11 - P8; P2 - P3; P2 - P8; P3 - P8
TC2100008521.hg.1	TC2100008521.hg.1	NM_001278650	B3GALT5	10317	60.191	4.43E-08	7.3534	1.46E-07	ctrl - P10; ctrl - P2; ctrl - P3; ctrl - P8; P11 - P10; P2 - P10; P3 - P10; P11 - P2; P11 - P3; P11 - P8; P2 - P8; P3 - P8
TC0800012319.hg.1	TC0800012319.hg.1	NR_105006	LINC01111	1.02E+08	60.158	4.45E-08	7.352	1.46E-07	ctrl - P10; ctrl - P11; ctrl - P2; ctrl - P3; ctrl - P8; P2 - P10; P10 - P3; P10 - P8; P2 - P11; P11 - P3; P11 - P8; P2 - P3; P2 - P8; P8 - P3
TC1000012480.hg.1	TC1000012480.hg.1	NM_000772	CYP2C18	1562	59.839	4.58E-08	7.3388	1.51E-07	P10 - ctrl; P11 - ctrl; P3 - ctrl; P8 - ctrl; P10 - P11; P10 - P2; P10 - P3; P10 - P8; P8 - P11; P8 - P2; P8 - P3
TC1100013081.hg.1	TC1100013081.hg.1	NM_002033	FUT4	2526	59.836	4.59E-08	7.3386	1.51E-07	P10 - ctrl; P3 - ctrl; P8 - ctrl; P10 - P11; P10 - P2; P10 - P3; P8 - P11; P8 - P2; P8 - P3
TC0700012998.hg.1	TC0700012998.hg.1	NR_027788	ZNF767P	79970	59.825	4.59E-08	7.3382	1.51E-07	ctrl - P10; ctrl - P11; ctrl - P3; ctrl - P8; P11 - P10; P2 - P10; P3 - P10; P2 - P11; P11 - P8; P2 - P3; P2 - P8; P3 - P8
TC1000008226.hg.1	TC1000008226.hg.1	NR_038464	LINC00857	439990	59.643	4.67E-08	7.3306	1.53E-07	P10 - ctrl; P11 - ctrl; P3 - ctrl; P8 - ctrl; P10 - P11; P10 - P2; P10 - P3; P8 - P11; P3 - P2; P8 - P2; P8 - P3
TC0200009902.hg.1	TC0200009902.hg.1	NM_001171631	NOSTRIN	115677	59.589	4.69E-08	7.3284	1.54E-07	ctrl - P10; ctrl - P2; P3 - ctrl; ctrl - P8; P11 - P10; P2 - P10; P3 - P10; P11 - P2; P3 - P11; P11 - P8; P3 - P2; P2 - P8; P3 - P8
TC0700008107.hg.1	TC0700008107.hg.1	klorpla.aAug10-unspliced	klorpla	NA	59.475	4.75E-08	7.3236	1.55E-07	ctrl - P10; ctrl - P11; P2 - ctrl; ctrl - P3; ctrl - P8; P11 - P10; P2 - P10; P3 - P10; P10 - P8; P2 - P11; P11 - P8; P2 - P3; P2 - P8; P3 - P8
TC0800007731.hg.1	TC0800007731.hg.1	kehare.aAug10-unspliced	kehare	NA	59.359	4.80E-08	7.3188	1.57E-07	ctrl - P10; ctrl - P11; ctrl - P3; ctrl - P8; P11 - P10; P2 - P10; P3 - P10; P10 - P8; P2 - P11; P11 - P8; P2 - P3; P2 - P8; P3 - P8
TC1500010712.hg.1	TC1500010712.hg.1	ENST00000448387	GOLGA8N	643699	59.204	4.87E-08	7.3123	1.59E-07	ctrl - P10; ctrl - P11; ctrl - P2; ctrl - P3; ctrl - P8; P11 - P10; P2 - P10; P3 - P10; P2 - P11; P11 - P8; P2 - P3; P2 - P8; P3 - P8
TC0400010284.hg.1	TC0400010284.hg.1	jorko.aAug10-unspliced	jorko	NA	59.189	4.88E-08	7.3116	1.59E-07	ctrl - P10; P2 - ctrl; ctrl - P8; P11 - P10; P2 - P10; P3 - P10; P10 - P8; P2 - P11; P11 - P8; P2 - P3; P2 - P8; P3 - P8
TC1000007641.hg.1	TC1000007641.hg.1	NM_012242	DKK1	22943	59.136	4.90E-08	7.3094	1.59E-07	P10 - ctrl; P3 - ctrl; P8 - ctrl; P10 - P11; P10 - P2; P3 - P10; P8 - P10; P3 - P11; P8 - P11; P3 - P2; P8 - P2; P3 - P8
TC0600011491.hg.1	TC0600011491.hg.1	NM_002125	HLA-DRB5	3127	59.05	4.95E-08	7.3058	1.60E-07	P10 - ctrl; P8 - ctrl; P10 - P11; P10 - P2; P10 - P3; P8 - P11; P8 - P2; P8 - P3
TC0400008328.hg.1	TC0400008328.hg.1	teesnarby.aAug10-unspliced	teesnarby	NA	59.031	4.95E-08	7.305	1.61E-07	ctrl - P10; ctrl - P11; ctrl - P3; ctrl - P8; P11 - P10; P2 - P10; P10 - P8; P2 - P11; P11 - P3; P11 - P8; P2 - P3; P2 - P8; P3 - P8
TC0100010397.hg.1	TC0100010397.hg.1	NM_031423	NUF2	83540	58.978	4.98E-08	7.3028	1.61E-07	P10 - ctrl; ctrl - P2; P8 - ctrl; P10 - P11; P10 - P2; P10 - P3; P8 - P10; P3 - P11; P8 - P11; P3 - P2; P8 - P2; P8 - P3
TC0700007198.hg.1	TC0700007198.hg.1	NM_001284301	ANLN	54443	58.848	5.04E-08	7.2973	1.63E-07	P10 - ctrl; ctrl - P11; ctrl - P2; P8 - ctrl; P10 - P11; P10 - P2; P10 - P3; P8 - P10; P3 - P11; P8 - P11; P3 - P2; P8 - P2; P8 - P3
TC0200012633.hg.1	TC0200012633.hg.1	seelar.aAug10-unspliced	seelar	NA	58.808	5.06E-08	7.2956	1.63E-07	ctrl - P10; ctrl - P11; ctrl - P3; ctrl - P8; P11 - P10; P2 - P10; P10 - P8; P2 - P11; P11 - P3; P11 - P8; P2 - P3; P2 - P8; P3 - P8
TC0200012281.hg.1	TC0200012281.hg.1	ENST00000450854.1	AC009229.5		58.651	5.14E-08	7.289	1.66E-07	ctrl - P10; P11 - ctrl; P3 - ctrl; ctrl - P8; P11 - P10; P2 - P10; P3 - P10; P11 - P2; P11 - P8; P3 - P2; P2 - P8; P3 - P8
TC0100015445.hg.1	TC0100015445.hg.1	NM_152380	TBX15	6913	58.556	5.19E-08	7.285	1.67E-07	P10 - ctrl; ctrl - P11; P2 - ctrl; P3 - ctrl; P8 - ctrl; P10 - P11; P3 - P10; P8 - P10; P2 - P11; P3 - P11; P8 - P11; P3 - P2; P8 - P2
TC0200013903.hg.1	TC0200013903.hg.1	teyder.aAug10-unspliced	teyder	NA	58.498	5.22E-08	7.2825	1.68E-07	ctrl - P10; ctrl - P11; ctrl - P3; ctrl - P8; P11 - P10; P2 - P10; P3 - P10; P10 - P8; P2 - P11; P11 - P8; P2 - P3; P2 - P8; P3 - P8
TC0X00008724.hg.1	TC0X00008724.hg.1	NM_001301228	HMGB3	3149	58.37	5.28E-08	7.2771	1.70E-07	P10 - ctrl; P3 - ctrl; P8 - ctrl; P10 - P11; P10 - P2; P10 - P3; P3 - P11; P8 - P11; P3 - P2; P8 - P2; P8 - P3
TC0900008442.hg.1	TC0900008442.hg.1	temore.aAug10-unspliced	temore	NA	58.345	5.30E-08	7.276	1.70E-07	ctrl - P10; ctrl - P11; P2 - ctrl; ctrl - P3; ctrl - P8; P2 - P10; P3 - P10; P2 - P11; P11 - P8; P2 - P3; P2 - P8; P3 - P8
TC1400010647.hg.1	TC1400010647.hg.1	NM_000623	BDKRB2	624	58.273	5.33E-08	7.2729	1.71E-07	ctrl - P10; ctrl - P11; ctrl - P2; ctrl - P3; P10 - P2; P10 - P3; P8 - P10; P11 - P2; P11 - P3; P8 - P11; P8 - P2; P8 - P3
TC1600010100.hg.1	TC1600010100.hg.1	NA	NA	NA	58.262	5.34E-08	7.2725	1.71E-07	ctrl - P10; ctrl - P11; P2 - ctrl; ctrl - P3; ctrl - P8; P2 - P10; P3 - P10; P2 - P11; P3 - P11; P2 - P3; P2 - P8; P3 - P8
TC0100008101.hg.1	TC0100008101.hg.1	NM_001297655	KIF2C	11004	58.234	5.35E-08	7.2713	1.71E-07	P10 - ctrl; ctrl - P11; ctrl - P2; P8 - ctrl; P10 - P11; P10 - P2; P10 - P3; P8 - P10; P3 - P11; P8 - P11; P3 - P2; P8 - P2; P8 - P3
TC0200013311.hg.1	TC0200013311.hg.1	NM_001164730	REEP1	65055	58.136	5.41E-08	7.2671	1.72E-07	P10 - ctrl; P11 - ctrl; P3 - ctrl; P8 - ctrl; P10 - P11; P10 - P2; P11 - P2; P3 - P11; P8 - P11; P3 - P2; P8 - P2; P8 - P3
TC1500006571.hg.1	TC1500006571.hg.1	NA	NA	NA	58.133	5.41E-08	7.267	1.72E-07	ctrl - P10; ctrl - P3; ctrl - P8; P11 - P10; P2 - P10; P3 - P10; P2 - P11; P11 - P3; P11 - P8; P2 - P3; P2 - P8; P3 - P8
TC0900011850.hg.1	TC0900011850.hg.1	NM_001145099	SLC2A6	11182	58.007	5.48E-08	7.2616	1.74E-07	P10 - ctrl; ctrl - P11; ctrl - P2; P3 - ctrl; P8 - ctrl; P10 - P11; P10 - P2; P10 - P3; P8 - P10; P3 - P11; P8 - P11; P3 - P2; P8 - P2; P8 - P3
TC0800011129.hg.1	TC0800011129.hg.1	NM_001144663	CDH17	1015	57.956	5.50E-08	7.2594	1.75E-07	P10 - ctrl; P11 - ctrl; P2 - ctrl; P3 - ctrl; P8 - ctrl; P11 - P10; P10 - P2; P3 - P10; P11 - P2; P3 - P11; P11 - P8; P3 - P2; P8 - P2; P3 - P8
TC0300013733.hg.1	TC0300013733.hg.1	NM_005929	MFI2	4241	57.845	5.56E-08	7.2547	1.77E-07	P10 - ctrl; P11 - ctrl; P3 - ctrl; P8 - ctrl; P10 - P11; P10 - P2; P8 - P10; P11 - P2; P3 - P11; P8 - P11; P3 - P2; P8 - P2; P8 - P3
TC0800009961.hg.1	TC0800009961.hg.1	NM_001831	CLU	1191	57.834	5.57E-08	7.2542	1.77E-07	P10 - ctrl; ctrl - P11; ctrl - P2; P3 - ctrl; P8 - ctrl; P10 - P11; P10 - P2; P3 - P10; P11 - P2; P3 - P11; P8 - P11; P3 - P2; P8 - P2; P3 - P8
TC0X00008034.hg.1	TC0X00008034.hg.1	NM_198465	NRK	203447	57.788	5.60E-08	7.2522	1.77E-07	ctrl - P10; ctrl - P11; ctrl - P2; ctrl - P3; ctrl - P8; P11 - P10; P2 - P10; P3 - P10; P2 - P11; P3 - P11; P11 - P8; P3 - P2; P2 - P8; P3 - P8

TC1200011246.hg.1	TC1200011246.hg.1	NM_001207015	PTPRR	5801	57.708	5.64E-08	7.2488	1.78E-07	P10 - ctrl; P11 - ctrl; P3 - ctrl; P8 - ctrl; P10 - P11; P10 - P2; P10 - P3; P3 - P11; P8 - P11; P3 - P2; P8 - P2; P8 - P3
TC1400009962.hg.1	TC1400009962.hg.1	ENST00000555853.1	RP11-471B22.2		57.646	5.67E-08	7.2461	1.79E-07	P10 - ctrl; P11 - ctrl; P3 - ctrl; P8 - ctrl; P10 - P11; P10 - P2; P10 - P3; P11 - P2; P3 - P11; P8 - P11; P3 - P2; P8 - P2; P8 - P3
TC1900011963.hg.1	TC1900011963.hg.1	NM_001031850	PSG6	5675	57.614	5.69E-08	7.2447	1.80E-07	P10 - ctrl; ctrl - P2; P3 - ctrl; P8 - ctrl; P10 - P11; P10 - P2; P11 - P2; P3 - P11; P8 - P11; P3 - P2; P8 - P2
TC2100007810.hg.1	TC2100007810.hg.1	NA	NA	NA	57.58	5.71E-08	7.2433	1.80E-07	ctrl - P10; ctrl - P11; P2 - ctrl; ctrl - P3; ctrl - P8; P2 - P10; P3 - P10; P2 - P11; P3 - P11; P11 - P8; P2 - P3; P2 - P8; P3 - P8
TC0700010289.hg.1	TC0700010289.hg.1	NA	NA	NA	57.536	5.74E-08	7.2414	1.81E-07	ctrl - P10; ctrl - P11; ctrl - P2; ctrl - P3; ctrl - P8; P11 - P10; P2 - P10; P3 - P10; P2 - P11; P11 - P3; P11 - P8; P2 - P3; P2 - P8; P3 - P8
TC1000007704.hg.1	TC1000007704.hg.1	NA	NA	NA	57.421	5.80E-08	7.2364	1.82E-07	ctrl - P10; ctrl - P11; P2 - ctrl; ctrl - P3; ctrl - P8; P2 - P10; P3 - P10; P2 - P11; P3 - P11; P2 - P3; P2 - P8; P3 - P8
TC1600007107.hg.1	TC1600007107.hg.1	NM_001105248	TMC5	79838	57.414	5.81E-08	7.2361	1.82E-07	P10 - ctrl; P2 - ctrl; P8 - ctrl; P10 - P11; P10 - P2; P10 - P3; P8 - P11; P8 - P2; P8 - P3
TC1900009970.hg.1	TC1900009970.hg.1	NM_004335	BST2	684	57.401	5.81E-08	7.2356	1.82E-07	ctrl - P11; ctrl - P2; P3 - ctrl; P8 - ctrl; P10 - P11; P10 - P2; P3 - P10; P8 - P10; P11 - P2; P3 - P11; P8 - P11; P3 - P2; P8 - P2
TC0800009529.hg.1	TC0800009529.hg.1	NM_001201329	PPP1R3B	79660	57.4	5.81E-08	7.2355	1.82E-07	P10 - ctrl; ctrl - P2; P3 - ctrl; P8 - ctrl; P10 - P11; P10 - P2; P10 - P3; P10 - P8; P3 - P11; P8 - P11; P3 - P2; P8 - P2; P8 - P3
TC0700012787.hg.1	TC0700012787.hg.1	smeyperby.aAug10-unspliced	smeyperby	NA	57.365	5.83E-08	7.234	1.82E-07	ctrl - P10; ctrl - P11; P2 - ctrl; ctrl - P8; P11 - P10; P2 - P10; P3 - P10; P10 - P8; P2 - P11; P3 - P11; P11 - P8; P2 - P3; P2 - P8; P3 - P8
TC1800006602.hg.1	TC1800006602.hg.1	ENST00000608943.1	RP11-376P6.3		57.333	5.85E-08	7.2326	1.83E-07	ctrl - P10; ctrl - P11; P2 - ctrl; ctrl - P3; ctrl - P8; P2 - P10; P3 - P10; P10 - P8; P2 - P11; P3 - P11; P2 - P3; P2 - P8; P3 - P8
TC1100010410.hg.1	TC1100010410.hg.1	NM_001143805	BDNF	627	57.308	5.87E-08	7.2315	1.83E-07	P10 - ctrl; ctrl - P2; P3 - ctrl; P8 - ctrl; P10 - P11; P10 - P2; P10 - P3; P11 - P2; P3 - P11; P8 - P11; P3 - P2; P8 - P2; P8 - P3
TC0600008547.hg.1	TC0600008547.hg.1	ENST00000421318.1	RP11-554D15.4		57.249	5.90E-08	7.229	1.84E-07	P10 - ctrl; P2 - ctrl; P3 - ctrl; P8 - ctrl; P10 - P11; P10 - P3; P2 - P11; P3 - P11; P8 - P11; P2 - P3; P2 - P8; P8 - P3
TC1100011259.hg.1	TC1100011259.hg.1	NM_001300844	FOSL1	8061	57.186	5.94E-08	7.2263	1.85E-07	P10 - ctrl; ctrl - P2; P3 - ctrl; P8 - ctrl; P10 - P11; P10 - P2; P3 - P10; P8 - P10; P11 - P2; P3 - P11; P8 - P11; P3 - P2; P8 - P2
TC0300012112.hg.1	TC0300012112.hg.1	NM_003778	B4GALT4	8702	57.153	5.96E-08	7.2248	1.85E-07	P10 - ctrl; ctrl - P11; P3 - ctrl; P8 - ctrl; P10 - P11; P10 - P2; P3 - P10; P8 - P10; P2 - P11; P3 - P11; P8 - P11; P3 - P2; P8 - P2
TC0200014796.hg.1	TC0200014796.hg.1	kynorbo.aAug10-unspliced	kynorbo	NA	57.149	5.96E-08	7.2246	1.85E-07	ctrl - P10; ctrl - P11; ctrl - P2; ctrl - P3; ctrl - P8; P11 - P10; P2 - P10; P10 - P8; P2 - P11; P11 - P8; P2 - P3; P2 - P8; P3 - P8
TC0400009087.hg.1	TC0400009087.hg.1	skoyfley.aAug10-unspliced	skoyfley	NA	57.116	5.98E-08	7.2232	1.85E-07	ctrl - P10; P2 - ctrl; ctrl - P3; ctrl - P8; P11 - P10; P2 - P10; P3 - P10; P2 - P11; P11 - P3; P11 - P8; P2 - P3; P2 - P8; P3 - P8
TC0800010275.hg.1	TC0800010275.hg.1	NM_000930	PLAT	5327	56.914	6.10E-08	7.2145	1.89E-07	P3 - ctrl; P8 - ctrl; P10 - P2; P3 - P10; P8 - P10; P3 - P11; P8 - P11; P3 - P2; P8 - P2; P3 - P8
TC1100010529.hg.1	TC1100010529.hg.1	NA	NA	NA	56.827	6.16E-08	7.2107	1.90E-07	P10 - ctrl; P11 - ctrl; P3 - ctrl; P8 - ctrl; P10 - P11; P10 - P2; P3 - P11; P8 - P11; P3 - P2; P8 - P2
TC1200008595.hg.1	TC1200008595.hg.1	NM_001206977	NR1H4	9971	56.716	6.23E-08	7.2058	1.92E-07	ctrl - P10; ctrl - P11; ctrl - P2; ctrl - P3; ctrl - P8; P11 - P10; P2 - P10; P3 - P10; P11 - P3; P11 - P8; P2 - P3; P2 - P8; P3 - P8
HTA2-pos-PSR02023979.hg.1	23151072	NA	NA	NA	56.578	6.31E-08	7.1998	1.95E-07	ctrl - P10; ctrl - P11; ctrl - P3; ctrl - P8; P11 - P10; P2 - P10; P3 - P10; P2 - P11; P11 - P8; P2 - P3; P2 - P8; P3 - P8
TC0200009402.hg.1	TC0200009402.hg.1	NM_002410	MGAT5	4249	56.537	6.34E-08	7.198	1.95E-07	P10 - ctrl; P11 - ctrl; ctrl - P2; P3 - ctrl; P8 - ctrl; P10 - P11; P10 - P2; P10 - P3; P11 - P2; P3 - P11; P8 - P11; P3 - P2; P8 - P2; P8 - P3
TC0400006887.hg.1	TC0400006887.hg.1	ENST00000508998.1	RP11-281P23.2		56.461	6.39E-08	7.1946	1.97E-07	P10 - ctrl; P3 - ctrl; P8 - ctrl; P10 - P11; P10 - P2; P2 - P11; P3 - P11; P8 - P11; P3 - P2; P8 - P2; P3 - P8
TC1500010617.hg.1	TC1500010617.hg.1	wopoy.aAug10-unspliced	wopoy	NA	56.362	6.45E-08	7.1903	1.98E-07	ctrl - P10; ctrl - P11; ctrl - P3; ctrl - P8; P11 - P10; P2 - P10; P3 - P10; P2 - P11; P11 - P8; P2 - P3; P2 - P8; P3 - P8
TC0800011591.hg.1	TC0800011591.hg.1	snorla.aAug10-unspliced	snorla	NA	56.157	6.59E-08	7.1813	2.02E-07	ctrl - P10; ctrl - P11; P2 - ctrl; ctrl - P3; ctrl - P8; P10 - P11; P2 - P10; P3 - P10; P10 - P8; P2 - P11; P3 - P11; P2 - P3; P2 - P8; P3 - P8
TC1900007688.hg.1	TC1900007688.hg.1	NM_001238	CCNE1	898	56.079	6.64E-08	7.1778	2.03E-07	P10 - ctrl; ctrl - P11; ctrl - P2; P3 - ctrl; P8 - ctrl; P10 - P11; P10 - P2; P8 - P10; P3 - P11; P8 - P11; P3 - P2; P8 - P2; P8 - P3
TC1900011112.hg.1	TC1900011112.hg.1	uc061ayc.1	AC026803.1	NA	56.077	6.64E-08	7.1778	2.03E-07	P10 - ctrl; P11 - ctrl; ctrl - P2; P3 - ctrl; P8 - ctrl; P10 - P2; P10 - P8; P11 - P2; P11 - P8; P3 - P2; P8 - P2; P3 - P8
TC0300012137.hg.1	TC0300012137.hg.1	uc062mwe.1	AC092910.1	NA	55.97	6.71E-08	7.173	2.05E-07	ctrl - P10; ctrl - P11; P2 - ctrl; ctrl - P8; P11 - P10; P2 - P10; P3 - P10; P2 - P11; P3 - P11; P11 - P8; P2 - P3; P2 - P8; P3 - P8
TC0500010847.hg.1	TC0500010847.hg.1	nypluby.aAug10-unspliced	nypluby	NA	55.953	6.73E-08	7.1723	2.05E-07	ctrl - P10; ctrl - P3; ctrl - P8; P11 - P10; P2 - P10; P3 - P10; P11 - P8; P2 - P3; P2 - P8; P3 - P8
TC1400007223.hg.1	TC1400007223.hg.1	cheyslor.aAug10-unspliced	cheyslor	NA	55.642	6.94E-08	7.1585	2.12E-07	ctrl - P10; ctrl - P11; ctrl - P3; ctrl - P8; P2 - P10; P3 - P10; P10 - P8; P2 - P11; P3 - P11; P11 - P8; P2 - P3; P2 - P8; P3 - P8
TC0100009797.hg.1	TC0100009797.hg.1	blawda.aAug10	blawda		55.633	6.95E-08	7.1581	2.12E-07	ctrl - P10; P3 - ctrl; ctrl - P8; P11 - P10; P2 - P10; P3 - P10; P3 - P11; P11 - P8; P3 - P2; P2 - P8; P3 - P8
TC1200012616.hg.1	TC1200012616.hg.1	NA	NA	NA	55.587	6.98E-08	7.156	2.13E-07	ctrl - P10; ctrl - P11; P2 - ctrl; ctrl - P3; ctrl - P8; P2 - P10; P3 - P10; P2 - P11; P11 - P8; P2 - P3; P2 - P8; P3 - P8
TC0100007645.hg.1	TC0100007645.hg.1	NM_001204414	TINAGL1	64129	55.576	6.99E-08	7.1555	2.13E-07	P10 - ctrl; ctrl - P2; P3 - ctrl; P8 - ctrl; P10 - P11; P10 - P2; P8 - P10; P11 - P2; P3 - P11; P8 - P11; P3 - P2; P8 - P2; P8 - P3

TC0100007746.hg.1	TC0100007746.hg.1	NM_153212	GJB4	127534	55.445	7.08E-08	7.1497	2.15E-07	P10 - ctrl; P3 - ctrl; P8 - ctrl; P10 - P11; P10 - P2; P11 - P2; P3 - P11; P8 - P11; P3 - P2; P8 - P2
TC0X00010076.hg.1	TC0X00010076.hg.1	teti.aAug10-unspliced	teti	NA	55.407	7.11E-08	7.148	2.16E-07	ctrl - P10; ctrl - P11; P2 - ctrl; P3 - ctrl; ctrl - P8; P2 - P10; P3 - P10; P10 - P8; P2 - P11; P3 - P11; P11 - P8; P2 - P3; P2 - P8; P3 - P8
TC0900010664.hg.1	TC0900010664.hg.1	NA	NA	NA	55.268	7.22E-08	7.1418	2.19E-07	P10 - ctrl; P3 - ctrl; P8 - ctrl; P10 - P11; P10 - P2; P10 - P3; P2 - P11; P3 - P11; P8 - P11; P3 - P2; P8 - P2; P8 - P3
TC0700012107.hg.1	TC0700012107.hg.1	feysmeebu.aAug10-unspliced	feysmeebu	NA	55.243	7.23E-08	7.1407	2.19E-07	ctrl - P10; ctrl - P11; P2 - ctrl; ctrl - P3; ctrl - P8; P2 - P10; P3 - P10; P10 - P8; P2 - P11; P11 - P8; P2 - P3; P2 - P8; P3 - P8
TC0700008624.hg.1	TC0700008624.hg.1	NA	NA	NA	55.211	7.26E-08	7.1392	2.19E-07	ctrl - P10; ctrl - P11; ctrl - P3; ctrl - P8; P11 - P10; P2 - P10; P3 - P10; P2 - P11; P3 - P11; P11 - P8; P2 - P3; P2 - P8; P3 - P8
TC2000007938.hg.1	TC2000007938.hg.1	gleytu.aAug10-unspliced	gleytu	NA	55.039	7.39E-08	7.1315	2.23E-07	ctrl - P10; ctrl - P11; ctrl - P3; ctrl - P8; P11 - P10; P2 - P10; P3 - P10; P10 - P8; P2 - P11; P11 - P3; P11 - P8; P2 - P3; P2 - P8; P3 - P8
TC0700009352.hg.1	TC0700009352.hg.1	NM_001061	TBXAS1	6916	54.987	7.43E-08	7.1292	2.24E-07	P10 - ctrl; P3 - ctrl; P8 - ctrl; P10 - P11; P10 - P2; P3 - P10; P3 - P11; P8 - P11; P3 - P2; P8 - P2; P3 - P8
TC1000008482.hg.1	TC1000008482.hg.1	NM_001127182	CEP55	55165	54.984	7.43E-08	7.129	2.24E-07	P10 - ctrl; ctrl - P11; ctrl - P2; P8 - ctrl; P10 - P11; P10 - P2; P10 - P3; P8 - P10; P3 - P11; P8 - P11; P3 - P2; P8 - P2; P8 - P3
TC0300006465.hg.1	TC0300006465.hg.1	NM_020873	LRRN1	57633	54.963	7.45E-08	7.1281	2.24E-07	ctrl - P11; ctrl - P2; P3 - ctrl; P8 - ctrl; P10 - P11; P10 - P2; P3 - P10; P8 - P10; P3 - P11; P8 - P11; P3 - P2; P8 - P2
TC1600008159.hg.1	TC1600008159.hg.1	NM_000196	HSD11B2	3291	54.951	7.46E-08	7.1275	2.24E-07	ctrl - P10; P11 - ctrl; ctrl - P2; ctrl - P3; ctrl - P8; P11 - P10; P11 - P2; P11 - P3; P11 - P8
TC1000012052.hg.1	TC1000012052.hg.1	moyshu.aAug10-unspliced	moyshu		54.924	7.48E-08	7.1263	2.24E-07	ctrl - P10; ctrl - P11; ctrl - P3; ctrl - P8; P11 - P10; P2 - P10; P2 - P11; P11 - P3; P11 - P8; P2 - P3; P2 - P8
TC0600011057.hg.1	TC0600011057.hg.1	NA	NA	NA	54.918	7.48E-08	7.1261	2.24E-07	ctrl - P10; ctrl - P11; P2 - ctrl; ctrl - P3; ctrl - P8; P11 - P10; P2 - P10; P3 - P10; P10 - P8; P2 - P11; P11 - P8; P2 - P3; P2 - P8; P3 - P8
TC0300013844.hg.1	TC0300013844.hg.1	NA	NA	NA	54.9	7.49E-08	7.1253	2.24E-07	ctrl - P10; ctrl - P2; ctrl - P3; ctrl - P8; P11 - P10; P2 - P10; P11 - P2; P11 - P3; P11 - P8; P2 - P3; P2 - P8
TC0800011717.hg.1	TC0800011717.hg.1	shofarby.aAug10-unspliced	shofarby	NA	54.828	7.55E-08	7.122	2.26E-07	ctrl - P10; ctrl - P11; P2 - ctrl; ctrl - P8; P11 - P10; P2 - P10; P3 - P10; P2 - P11; P11 - P8; P2 - P3; P2 - P8; P3 - P8
TC0100013700.hg.1	TC0100013700.hg.1	NM_001190481	CLSPN	63967	54.764	7.60E-08	7.1191	2.27E-07	P10 - ctrl; ctrl - P11; P3 - ctrl; P8 - ctrl; P10 - P11; P10 - P2; P8 - P10; P2 - P11; P3 - P11; P8 - P11; P3 - P2; P8 - P2; P8 - P3
TC1600009922.hg.1	TC1600009922.hg.1	NA	NA	NA	54.73	7.63E-08	7.1176	2.27E-07	ctrl - P10; ctrl - P11; P2 - ctrl; ctrl - P3; ctrl - P8; P11 - P10; P2 - P10; P3 - P10; P10 - P8; P2 - P11; P11 - P8; P2 - P3; P2 - P8; P3 - P8
TC1800007014.hg.1	TC1800007014.hg.1	NM_001943	DSG2	1829	54.689	7.66E-08	7.1157	2.28E-07	P10 - ctrl; P3 - ctrl; P8 - ctrl; P10 - P11; P10 - P2; P10 - P3; P11 - P2; P3 - P11; P8 - P11; P3 - P2; P8 - P2; P8 - P3
TC0600013884.hg.1	TC0600013884.hg.1	glawsnerbu.aAug10-unspliced	glawsnerbu	NA	54.666	7.68E-08	7.1147	2.28E-07	ctrl - P10; ctrl - P11; ctrl - P2; ctrl - P3; ctrl - P8; P11 - P10; P2 - P10; P3 - P10; P10 - P8; P11 - P3; P11 - P8; P2 - P3; P2 - P8; P3 - P8
TC0400007556.hg.1	TC0400007556.hg.1	NM_024592	SRD5A3	79644	54.385	7.91E-08	7.1019	2.35E-07	P10 - ctrl; ctrl - P2; P8 - ctrl; P10 - P11; P10 - P2; P10 - P3; P11 - P2; P8 - P11; P3 - P2; P8 - P2; P8 - P3
TC0200007098.hg.1	TC0200007098.hg.1	ENST00000605056.1	RP11-373D23.3		54.325	7.96E-08	7.0992	2.36E-07	P10 - ctrl; ctrl - P11; P3 - ctrl; P8 - ctrl; P10 - P11; P10 - P2; P2 - P11; P3 - P11; P8 - P11; P3 - P2; P8 - P2
TC1700010790.hg.1	TC1700010790.hg.1	NM_001079675	ETV4	2118	54.301	7.98E-08	7.0981	2.37E-07	P10 - ctrl; P11 - ctrl; ctrl - P2; P3 - ctrl; P8 - ctrl; P10 - P2; P3 - P10; P8 - P10; P11 - P2; P3 - P11; P8 - P11; P3 - P2; P8 - P2
TC0600008670.hg.1	TC0600008670.hg.1	NM_001170423	PRSS35	167681	54.176	8.08E-08	7.0924	2.39E-07	ctrl - P11; ctrl - P2; ctrl - P3; ctrl - P8; P10 - P11; P10 - P2; P10 - P3; P10 - P8; P11 - P2; P11 - P3; P11 - P8; P8 - P3
TC0600011124.hg.1	TC0600011124.hg.1	NM_003537	HIST1H3B	8358	53.758	8.45E-08	7.0733	2.50E-07	P10 - ctrl; ctrl - P2; P3 - ctrl; P8 - ctrl; P10 - P11; P10 - P2; P10 - P3; P3 - P11; P8 - P11; P3 - P2; P8 - P2; P8 - P3
TC0200011192.hg.1	TC0200011192.hg.1	skydu.aAug10-unspliced	skydu	NA	53.649	8.55E-08	7.0683	2.52E-07	ctrl - P10; ctrl - P11; ctrl - P3; ctrl - P8; P11 - P10; P2 - P10; P3 - P10; P10 - P8; P2 - P11; P11 - P8; P2 - P3; P2 - P8; P3 - P8
TC0X00010958.hg.1	TC0X00010958.hg.1	skeeler.aAug10-unspliced	skeeler	NA	53.597	8.59E-08	7.0659	2.54E-07	ctrl - P10; ctrl - P3; ctrl - P8; P11 - P10; P2 - P10; P11 - P3; P11 - P8; P2 - P3; P2 - P8; P3 - P8
TC1600008993.hg.1	TC1600008993.hg.1	stozubu.aAug10-unspliced	stozubu	NA	53.566	8.62E-08	7.0644	2.54E-07	ctrl - P10; ctrl - P11; ctrl - P3; ctrl - P8; P11 - P10; P2 - P10; P3 - P10; P10 - P8; P2 - P11; P11 - P3; P11 - P8; P2 - P3; P2 - P8; P3 - P8
TC0300006545.hg.1	TC0300006545.hg.1	fudawbo.aAug10-unspliced	fudawbo	NA	53.546	8.64E-08	7.0635	2.54E-07	ctrl - P10; ctrl - P11; P2 - ctrl; ctrl - P3; ctrl - P8; P2 - P10; P3 - P10; P2 - P11; P3 - P11; P11 - P8; P2 - P3; P2 - P8; P3 - P8
TC0700012551.hg.1	TC0700012551.hg.1	ENST00000469533	RP11-309L24.6	NA	53.375	8.80E-08	7.0556	2.59E-07	P10 - ctrl; P3 - ctrl; P8 - ctrl; P10 - P11; P10 - P2; P8 - P10; P2 - P11; P3 - P11; P8 - P11; P3 - P2; P8 - P2; P8 - P3
TC0200014798.hg.1	TC0200014798.hg.1	NA	NA	NA	53.355	8.82E-08	7.0547	2.59E-07	ctrl - P10; ctrl - P11; ctrl - P3; ctrl - P8; P2 - P10; P10 - P8; P2 - P11; P11 - P8; P2 - P3; P2 - P8; P3 - P8
TC0900008504.hg.1	TC0900008504.hg.1	NM_032888	COL27A1	85301	53.222	8.94E-08	7.0485	2.62E-07	ctrl - P10; ctrl - P11; ctrl - P3; ctrl - P8; P11 - P10; P2 - P10; P3 - P10; P2 - P11; P11 - P3; P11 - P8; P2 - P3; P2 - P8; P3 - P8
TC0X00009069.hg.1	TC0X00009069.hg.1	NM_001287242	ARHGAP6	395	53.161	9.00E-08	7.0457	2.64E-07	ctrl - P10; ctrl - P2; ctrl - P3; ctrl - P8; P11 - P10; P2 - P10; P3 - P10; P11 - P2; P11 - P3; P11 - P8; P2 - P8; P3 - P8
TC1000008347.hg.1	TC1000008347.hg.1	taklarbu.aAug10-unspliced	taklarbu	NA	52.985	9.17E-08	7.0375	2.68E-07	ctrl - P10; ctrl - P11; P2 - ctrl; ctrl - P3; ctrl - P8; P11 - P10; P2 - P10; P3 - P10; P10 - P8; P2 - P11; P11 - P8; P2 - P3; P2 - P8; P3 - P8
TC0600011058.hg.1	TC0600011058.hg.1	NA	NA	NA	52.98	9.18E-08	7.0373	2.68E-07	ctrl - P10; ctrl - P11; P2 - ctrl; ctrl - P3; ctrl - P8; P2 - P10; P10 - P8; P2 - P11; P11 - P8; P2 - P3; P2 - P8; P3 - P8

TC0500011010.hg.1	TC0500011010.hg.1	bystawbu.aAug10-unspliced	bystawbu	NA	52.866	9.29E-08	7.0319	2.71E-07	ctrl - P10; ctrl - P11; ctrl - P3; ctrl - P8; P11 - P10; P2 - P10; P3 - P10; P10 - P8; P2 - P11; P11 - P8; P2 - P3; P2 - P8; P3 - P8
TC0500010852.hg.1	TC0500010852.hg.1	kuflu.bAug10-unspliced	kuflu	NA	52.802	9.35E-08	7.029	2.73E-07	ctrl - P10; P3 - ctrl; ctrl - P8; P11 - P10; P2 - P10; P3 - P10; P3 - P11; P11 - P8; P3 - P2; P2 - P8; P3 - P8
TC1700009422.hg.1	TC1700009422.hg.1	ENST00000623339	RP11-667K14.9		52.783	9.37E-08	7.0281	2.73E-07	ctrl - P10; ctrl - P11; P2 - ctrl; ctrl - P3; ctrl - P8; P2 - P10; P3 - P10; P2 - P11; P3 - P11; P11 - P8; P2 - P3; P2 - P8; P3 - P8
TC1300007796.hg.1	TC1300007796.hg.1	ENST00000595998.1	RP11-573N10.1		52.735	9.42E-08	7.0259	2.74E-07	ctrl - P10; ctrl - P11; P2 - ctrl; ctrl - P3; ctrl - P8; P2 - P10; P10 - P8; P2 - P11; P3 - P11; P11 - P8; P2 - P3; P2 - P8; P3 - P8
TC0100007747.hg.1	TC0100007747.hg.1	NM_001005752	GJB3	2707	52.583	9.58E-08	7.0187	2.78E-07	P10 - ctrl; P3 - ctrl; P8 - ctrl; P10 - P11; P10 - P2; P3 - P11; P8 - P11; P3 - P2; P8 - P2
TC0100012675.hg.1	TC0100012675.hg.1	NM_007274	ACOT7	11332	52.457	9.71E-08	7.0128	2.82E-07	P10 - ctrl; ctrl - P2; P3 - ctrl; P8 - ctrl; P10 - P11; P10 - P2; P3 - P10; P8 - P10; P11 - P2; P3 - P11; P8 - P11; P3 - P2; P8 - P2
TC0200015620.hg.1	TC0200015620.hg.1	tuha.aAug10-unspliced	tuha	NA	52.389	9.78E-08	7.0096	2.84E-07	ctrl - P10; ctrl - P3; ctrl - P8; P11 - P10; P2 - P10; P10 - P3; P10 - P8; P11 - P3; P11 - P8; P2 - P3; P2 - P8
TC0100013498.hg.1	TC0100013498.hg.1	ENST00000611385	uc_338		52.355	9.82E-08	7.008	2.84E-07	ctrl - P10; ctrl - P11; P2 - ctrl; ctrl - P3; ctrl - P8; P2 - P10; P3 - P10; P10 - P8; P2 - P11; P11 - P8; P2 - P3; P2 - P8; P3 - P8
TC1500006963.hg.1	TC1500006963.hg.1	NM_144508	CASC5	57082	52.319	9.86E-08	7.0063	2.85E-07	P10 - ctrl; ctrl - P11; ctrl - P2; P3 - ctrl; P8 - ctrl; P10 - P11; P10 - P2; P10 - P3; P8 - P10; P3 - P11; P8 - P11; P3 - P2; P8 - P2; P8 - P3
TC0200008000.hg.1	TC0200008000.hg.1	roydawbu.aAug10-unspliced	roydawbu	NA	52.187	1.00E-07	7.0001	2.89E-07	ctrl - P10; ctrl - P3; ctrl - P8; P11 - P10; P2 - P10; P3 - P10; P2 - P11; P11 - P3; P11 - P8; P2 - P3; P2 - P8; P3 - P8
TC0600014184.hg.1	TC0600014184.hg.1	smufla.aAug10	smufla	NA	52.107	1.01E-07	6.9963	2.91E-07	P10 - ctrl; P11 - ctrl; P2 - ctrl; P3 - ctrl; P8 - ctrl; P10 - P11; P10 - P2; P10 - P3; P2 - P11; P8 - P11; P2 - P3; P8 - P2; P8 - P3
TC0200010625.hg.1	TC0200010625.hg.1	tikore.aAug10-unspliced	tikore	NA	51.845	1.04E-07	6.9839	2.99E-07	ctrl - P10; ctrl - P11; ctrl - P3; ctrl - P8; P11 - P10; P2 - P10; P3 - P10; P10 - P8; P2 - P11; P11 - P8; P2 - P3; P2 - P8; P3 - P8
TC0700013385.hg.1	TC0700013385.hg.1	NR_108104	LINC01372	1.02E+08	51.816	1.04E-07	6.9825	3.00E-07	ctrl - P10; ctrl - P11; ctrl - P3; ctrl - P8; P11 - P10; P2 - P10; P2 - P11; P11 - P8; P2 - P3; P2 - P8; P3 - P8
TC1900011419.hg.1	TC1900011419.hg.1	NA	NA	NA	51.77	1.05E-07	6.9803	3.01E-07	ctrl - P10; ctrl - P11; P2 - ctrl; ctrl - P3; ctrl - P8; P11 - P10; P2 - P10; P3 - P10; P10 - P8; P2 - P11; P11 - P8; P2 - P3; P2 - P8; P3 - P8
TC1100010105.hg.1	TC1100010105.hg.1	NM_198516	GALNT18	374378	51.758	1.05E-07	6.9797	3.01E-07	ctrl - P10; ctrl - P2; ctrl - P3; ctrl - P8; P11 - P10; P3 - P10; P11 - P2; P11 - P3; P11 - P8; P3 - P2; P2 - P8; P3 - P8
TC1700008757.hg.1	TC1700008757.hg.1	NM_002758	MAP2K6	5608	51.719	1.05E-07	6.9779	3.02E-07	ctrl - P10; ctrl - P11; ctrl - P2; ctrl - P3; ctrl - P8; P11 - P10; P2 - P10; P3 - P10; P8 - P10; P11 - P2; P11 - P3; P11 - P8; P2 - P8; P3 - P8
TC0600014307.hg.1	TC0600014307.hg.1	ENST00000625013	RP3-331H24.7		51.609	1.07E-07	6.9726	3.05E-07	ctrl - P10; ctrl - P11; P2 - ctrl; ctrl - P3; ctrl - P8; P10 - P11; P2 - P10; P10 - P8; P2 - P11; P3 - P11; P2 - P3; P2 - P8; P3 - P8
TC1400007201.hg.1	TC1400007201.hg.1	NM_001130851	CDKN3	1033	51.589	1.07E-07	6.9717	3.06E-07	P10 - ctrl; ctrl - P2; P8 - ctrl; P10 - P11; P10 - P2; P10 - P3; P8 - P10; P11 - P2; P3 - P11; P8 - P11; P3 - P2; P8 - P2; P8 - P3
TC0200011062.hg.1	TC0200011062.hg.1	NM_145236	B3GNT7	93010	51.577	1.07E-07	6.9711	3.06E-07	P10 - ctrl; P11 - ctrl; P3 - ctrl; P8 - ctrl; P10 - P11; P10 - P2; P10 - P3; P8 - P10; P11 - P2; P8 - P11; P3 - P2; P8 - P2; P8 - P3
TC0X00009075.hg.1	TC0X00009075.hg.1	NA	NA	NA	51.534	1.07E-07	6.969	3.07E-07	ctrl - P10; ctrl - P11; ctrl - P3; ctrl - P8; P11 - P10; P2 - P10; P3 - P10; P2 - P11; P11 - P3; P11 - P8; P2 - P3; P2 - P8; P3 - P8
TC1600010849.hg.1	TC1600010849.hg.1	ploserbo.aAug10-unspliced	ploserbo	NA	51.498	1.08E-07	6.9673	3.08E-07	ctrl - P10; ctrl - P11; ctrl - P3; ctrl - P8; P11 - P10; P2 - P10; P3 - P10; P2 - P11; P11 - P8; P2 - P3; P2 - P8; P3 - P8
TC0800007367.hg.1	TC0800007367.hg.1	NM_003816	ADAM9	8754	51.484	1.08E-07	6.9666	3.08E-07	P10 - ctrl; ctrl - P11; P8 - ctrl; P10 - P11; P10 - P2; P10 - P3; P3 - P11; P8 - P11; P3 - P2; P8 - P2; P8 - P3
TC0200014703.hg.1	TC0200014703.hg.1	NM_001301684	CCDC148	130940	51.459	1.08E-07	6.9654	3.08E-07	ctrl - P10; ctrl - P3; ctrl - P8; P11 - P10; P2 - P10; P3 - P10; P11 - P3; P11 - P8; P2 - P3; P2 - P8; P3 - P8
TC2100006627.hg.1	TC2100006627.hg.1	NR_027790	MIR99AHG	388815	51.373	1.09E-07	6.9613	3.11E-07	ctrl - P10; ctrl - P11; ctrl - P2; ctrl - P3; ctrl - P8; P11 - P10; P2 - P10; P3 - P10; P11 - P2; P11 - P3; P11 - P8; P2 - P3; P2 - P8
TC0200012339.hg.1	TC0200012339.hg.1	NM_001112800	SLC8A1	2p22.1	51.358	1.10E-07	6.9606	3.11E-07	P10 - ctrl; P11 - ctrl; P2 - ctrl; P8 - ctrl; P10 - P11; P10 - P2; P10 - P3; P10 - P8; P2 - P11; P11 - P3; P8 - P11; P2 - P3; P8 - P3
TC1100009110.hg.1	TC1100009110.hg.1	NM_006169	NNMT	4837	51.323	1.10E-07	6.9589	3.12E-07	P10 - ctrl; P3 - ctrl; P8 - ctrl; P10 - P11; P10 - P2; P3 - P11; P8 - P11; P3 - P2; P8 - P2
TC0500009863.hg.1	TC0500009863.hg.1	NM_024830	LPCAT1	79888	51.209	1.11E-07	6.9534	3.15E-07	P10 - ctrl; ctrl - P2; P3 - ctrl; P8 - ctrl; P10 - P11; P10 - P2; P8 - P10; P11 - P2; P3 - P11; P8 - P11; P3 - P2; P8 - P2
TC1000008388.hg.1	TC1000008388.hg.1	NM_000043	FAS	355	51.187	1.12E-07	6.9524	3.16E-07	P10 - ctrl; ctrl - P2; P3 - ctrl; P8 - ctrl; P10 - P11; P10 - P2; P3 - P11; P8 - P11; P3 - P2; P8 - P2
TC0900007129.hg.1	TC0900007129.hg.1	NM_001256685	MELK	9833	50.969	1.14E-07	6.9418	3.23E-07	P10 - ctrl; ctrl - P11; ctrl - P2; P3 - ctrl; P8 - ctrl; P10 - P11; P10 - P2; P8 - P10; P3 - P11; P8 - P11; P3 - P2; P8 - P2; P8 - P3
TC1600008235.hg.1	TC1600008235.hg.1	OTTHUMT00000474147	RP11-140H17.2		50.925	1.15E-07	6.9397	3.24E-07	ctrl - P10; ctrl - P11; P2 - ctrl; ctrl - P3; ctrl - P8; P11 - P10; P2 - P10; P3 - P10; P2 - P11; P11 - P8; P2 - P3; P2 - P8; P3 - P8
TC0200014766.hg.1	TC0200014766.hg.1	wadey.aAug10-unspliced	wadey	NA	50.909	1.15E-07	6.939	3.25E-07	ctrl - P10; ctrl - P8; P11 - P10; P2 - P10; P3 - P10; P10 - P8; P11 - P8; P2 - P8; P3 - P8
TC0600011144.hg.1	TC0600011144.hg.1	NA	NA	NA	50.804	1.16E-07	6.9338	3.28E-07	P10 - ctrl; P3 - ctrl; P8 - ctrl; P10 - P11; P10 - P2; P3 - P10; P11 - P2; P3 - P11; P8 - P11; P3 - P2; P8 - P2

TC0500013323.hg.1	TC0500013323.hg.1	hsa_circ_0072870	TCONS_l2_00023773		50.775	1.17E-07	6.9325	3.29E-07	ctrl - P10; ctrl - P11; ctrl - P3; ctrl - P8; P11 - P10; P2 - P10; P3 - P10; P2 - P11; P11 - P8; P2 - P3; P2 - P8; P3 - P8
TC0200015645.hg.1	TC0200015645.hg.1	NM_015657	ABCA12	26154	50.723	1.18E-07	6.9299	3.30E-07	P11 - ctrl; ctrl - P2; P3 - ctrl; P8 - ctrl; P11 - P10; P10 - P2; P3 - P10; P8 - P10; P11 - P2; P11 - P8; P3 - P2; P8 - P2; P3 - P8
TC0200009071.hg.1	TC0200009071.hg.1	NM_001271049	CFAP221	200373	50.627	1.19E-07	6.9253	3.34E-07	ctrl - P10; ctrl - P2; ctrl - P3; ctrl - P8; P11 - P10; P2 - P10; P10 - P3; P10 - P8; P11 - P2; P11 - P3; P11 - P8; P2 - P3; P2 - P8
TSUnmapped00000284.hg.1	TSUnmapped00000284.hg.1	ENST00000628182	LRP6	4040	50.585	1.19E-07	6.9232	3.35E-07	ctrl - P10; ctrl - P2; ctrl - P3; ctrl - P8; P11 - P10; P2 - P10; P8 - P10; P11 - P3; P11 - P8; P2 - P3; P2 - P8; P8 - P3
TC0300011868.hg.1	TC0300011868.hg.1	NA	NA	NA	50.509	1.20E-07	6.9195	3.37E-07	P10 - ctrl; P2 - ctrl; P3 - ctrl; P8 - ctrl; P10 - P11; P10 - P2; P10 - P3; P10 - P8; P2 - P11; P3 - P11; P8 - P11; P8 - P2; P8 - P3
TC2000006675.hg.1	TC2000006675.hg.1	swywar.aAug10-unspliced	swywar	NA	50.503	1.20E-07	6.9192	3.37E-07	ctrl - P10; ctrl - P11; ctrl - P3; ctrl - P8; P2 - P10; P3 - P10; P2 - P11; P3 - P11; P2 - P3; P2 - P8; P3 - P8
TC1600011361.hg.1	TC1600011361.hg.1	ENST00000614721	ACSM3	6296	50.409	1.22E-07	6.9147	3.40E-07	P10 - ctrl; P3 - ctrl; P8 - ctrl; P10 - P11; P10 - P2; P2 - P11; P3 - P11; P8 - P11; P3 - P2; P8 - P2; P8 - P3
TC1000012428.hg.1	TC1000012428.hg.1	NM_001253908	AKR1C3	8644	50.322	1.23E-07	6.9104	3.43E-07	P10 - ctrl; P11 - ctrl; P3 - ctrl; P8 - ctrl; P10 - P11; P10 - P2; P11 - P2; P3 - P11; P8 - P11; P3 - P2; P8 - P2; P8 - P3
TC1500006569.hg.1	TC1500006569.hg.1	NR_001292	SNORD108	15q11.2	50.226	1.24E-07	6.9057	3.47E-07	ctrl - P10; ctrl - P3; ctrl - P8; P11 - P10; P2 - P10; P3 - P10; P11 - P3; P11 - P8; P2 - P3; P2 - P8; P3 - P8
TC0900007518.hg.1	TC0900007518.hg.1	NM_153267	MAMDC2	256691	50.139	1.25E-07	6.9014	3.50E-07	ctrl - P10; ctrl - P11; ctrl - P2; ctrl - P8; P3 - P10; P8 - P10; P3 - P11; P8 - P11; P3 - P2; P8 - P2; P3 - P8
TC1100012425.hg.1	TC1100012425.hg.1	teyjeeby.aAug10-unspliced	teyjeeby	NA	50.116	1.26E-07	6.9003	3.50E-07	ctrl - P10; ctrl - P11; ctrl - P3; ctrl - P8; P11 - P10; P2 - P10; P3 - P10; P10 - P8; P2 - P11; P11 - P8; P2 - P3; P2 - P8; P3 - P8
TC1400008780.hg.1	TC1400008780.hg.1	NM_002515	NOVA1	4857	50.097	1.26E-07	6.8993	3.50E-07	ctrl - P10; ctrl - P11; ctrl - P2; ctrl - P8; P11 - P10; P2 - P10; P3 - P10; P3 - P11; P11 - P8; P3 - P2; P2 - P8; P3 - P8
TSUnmapped00000123.hg.1	TSUnmapped00000123.hg.1	ENST00000626271	SLC2A6	NA	50.08	1.26E-07	6.8985	3.51E-07	P10 - ctrl; ctrl - P11; ctrl - P2; P3 - ctrl; P8 - ctrl; P10 - P11; P10 - P2; P8 - P10; P3 - P11; P8 - P11; P3 - P2; P8 - P2; P8 - P3
TC0100013072.hg.1	TC0100013072.hg.1	NM_001141973	ATP13A2	23400	50.071	1.26E-07	6.8981	3.51E-07	P10 - ctrl; ctrl - P2; P3 - ctrl; P8 - ctrl; P10 - P11; P10 - P2; P11 - P2; P3 - P11; P8 - P11; P3 - P2; P8 - P2; P8 - P3
TC0700006584.hg.1	TC0700006584.hg.1	flerka.aAug10-unspliced	flerka	NA	50.004	1.27E-07	6.8948	3.53E-07	ctrl - P10; ctrl - P11; ctrl - P3; ctrl - P8; P2 - P10; P3 - P10; P2 - P11; P3 - P11; P11 - P8; P2 - P3; P2 - P8; P3 - P8
TSUnmapped00000268.hg.1	TSUnmapped00000268.hg.1	ENST00000627310	CCDC84	338657	49.991	1.28E-07	6.8941	3.53E-07	ctrl - P10; ctrl - P11; ctrl - P3; ctrl - P8; P2 - P10; P3 - P10; P2 - P11; P3 - P11; P2 - P3; P2 - P8; P3 - P8
TC1600007379.hg.1	TC1600007379.hg.1	NA	NA	NA	49.869	1.29E-07	6.8881	3.58E-07	ctrl - P10; ctrl - P11; ctrl - P3; ctrl - P8; P11 - P10; P2 - P10; P3 - P10; P2 - P11; P11 - P8; P2 - P3; P2 - P8; P3 - P8
TC1500006583.hg.1	TC1500006583.hg.1	NR_003297	SNORD115-5	15q11.2	49.84	1.30E-07	6.8867	3.58E-07	P10 - ctrl; P11 - ctrl; P2 - ctrl; P3 - ctrl; P8 - ctrl; P10 - P11; P10 - P2; P3 - P10; P3 - P11; P8 - P11; P3 - P2; P8 - P2; P3 - P8
TC1500006586.hg.1	TC1500006586.hg.1	ENST00000362912	SNORD115-9	15q11.2	49.84	1.30E-07	6.8867	3.58E-07	P10 - ctrl; P11 - ctrl; P2 - ctrl; P3 - ctrl; P8 - ctrl; P10 - P11; P10 - P2; P3 - P10; P3 - P11; P8 - P11; P3 - P2; P8 - P2; P3 - P8
TC1500006589.hg.1	TC1500006589.hg.1	ENST00000362583	SNORD115-12	15q11.2	49.84	1.30E-07	6.8867	3.58E-07	P10 - ctrl; P11 - ctrl; P2 - ctrl; P3 - ctrl; P8 - ctrl; P10 - P11; P10 - P2; P3 - P10; P3 - P11; P8 - P11; P3 - P2; P8 - P2; P3 - P8
TC0700012439.hg.1	TC0700012439.hg.1	NM_005763	AASS	10157	49.775	1.31E-07	6.8835	3.60E-07	ctrl - P10; ctrl - P2; ctrl - P3; ctrl - P8; P11 - P10; P2 - P10; P3 - P10; P11 - P3; P11 - P8; P2 - P3; P2 - P8
TC0100017315.hg.1	TC0100017315.hg.1	sheeply.aAug10-unspliced	sheeply	NA	49.741	1.31E-07	6.8818	3.61E-07	ctrl - P10; ctrl - P3; ctrl - P8; P11 - P10; P2 - P10; P11 - P3; P11 - P8; P2 - P3; P2 - P8; P3 - P8
TC0100018451.hg.1	TC0100018451.hg.1	NM_004120	GBP2	2634	49.669	1.32E-07	6.8782	3.63E-07	P10 - ctrl; P2 - ctrl; P3 - ctrl; P8 - ctrl; P10 - P11; P10 - P2; P10 - P3; P3 - P11; P8 - P11; P3 - P2; P8 - P2; P8 - P3
TC0400009003.hg.1	TC0400009003.hg.1	ENST00000517210	RN7SKP35	NA	49.669	1.32E-07	6.8782	3.63E-07	ctrl - P10; ctrl - P11; ctrl - P3; ctrl - P8; P11 - P10; P2 - P10; P3 - P10; P2 - P11; P11 - P8; P2 - P3; P2 - P8; P3 - P8
TC0200016353.hg.1	TC0200016353.hg.1	jawklo.aAug10-unspliced	jawklo	NA	49.654	1.33E-07	6.8775	3.63E-07	ctrl - P10; ctrl - P11; P2 - ctrl; ctrl - P3; ctrl - P8; P11 - P10; P2 - P10; P3 - P10; P2 - P11; P3 - P11; P11 - P8; P2 - P3; P2 - P8; P3 - P8
TC0600009597.hg.1	TC0600009597.hg.1	NM_001270507	TNFAIP3	7128	49.557	1.34E-07	6.8727	3.67E-07	P10 - ctrl; P11 - ctrl; P3 - ctrl; P8 - ctrl; P10 - P11; P10 - P2; P10 - P3; P3 - P11; P8 - P11; P3 - P2; P8 - P2; P8 - P3
TC1600011372.hg.1	TC1600011372.hg.1	NM_001017390	SULT1A4	445329	49.443	1.36E-07	6.867	3.71E-07	P10 - ctrl; P3 - ctrl; P8 - ctrl; P10 - P11; P10 - P2; P10 - P3; P3 - P11; P8 - P11; P3 - P2; P8 - P2; P8 - P3
TC1800008895.hg.1	TC1800008895.hg.1	stasky.aAug10-unspliced	stasky	NA	49.407	1.36E-07	6.8652	3.73E-07	ctrl - P10; ctrl - P11; P2 - ctrl; ctrl - P3; ctrl - P8; P2 - P10; P10 - P8; P2 - P11; P11 - P8; P2 - P3; P2 - P8; P3 - P8
TC1500006590.hg.1	TC1500006590.hg.1	NR_003306	SNORD115-14	15q11.2	49.314	1.38E-07	6.8606	3.76E-07	P10 - ctrl; P11 - ctrl; P2 - ctrl; P3 - ctrl; P8 - ctrl; P10 - P11; P10 - P2; P3 - P10; P3 - P11; P8 - P11; P3 - P2; P8 - P2; P3 - P8
TC0X00008080.hg.1	TC0X00008080.hg.1	NM_001170553	VSIG1	340547	49.142	1.41E-07	6.852	3.83E-07	P10 - ctrl; ctrl - P2; P3 - ctrl; P8 - ctrl; P10 - P11; P10 - P2; P10 - P3; P3 - P11; P8 - P11; P3 - P2; P8 - P2; P8 - P3
TC0200009759.hg.1	TC0200009759.hg.1	smuneebo.aAug10-unspliced	smuneebo	NA	49.118	1.41E-07	6.8508	3.84E-07	ctrl - P10; P2 - ctrl; ctrl - P3; ctrl - P8; P11 - P10; P2 - P10; P3 - P10; P10 - P8; P2 - P11; P11 - P8; P2 - P3; P2 - P8; P3 - P8
TC0100009938.hg.1	TC0100009938.hg.1	NM_006818	MLLT11	1q21	49.113	1.41E-07	6.8505	3.84E-07	ctrl - P11; ctrl - P2; P3 - ctrl; P8 - ctrl; P10 - P11; P10 - P2; P3 - P10; P8 - P10; P11 - P2; P3 - P11; P8 - P11; P3 - P2; P8 - P2; P8 - P3
TC0100012172.hg.1	TC0100012172.hg.1	NM_003686	EXO1	9156	49.104	1.41E-07	6.8501	3.84E-07	P10 - ctrl; ctrl - P11; ctrl - P2; P3 - ctrl; P8 - ctrl; P10 - P11; P10 - P2; P8 - P10; P3 - P11; P8 - P11; P3 - P2; P8 - P2; P8 - P3

TC0200014791.hg.1	TC0200014791.hg.1	voykla.aAug10-unspliced	voykla	NA	49.068	1.42E-07	6.8483	3.85E-07	ctrl - P10; ctrl - P11; P2 - ctrl; ctrl - P3; ctrl - P8; P2 - P10; P3 - P10; P10 - P8; P2 - P11; P3 - P11; P11 - P8; P2 - P3; P2 - P8; P3 - P8
TC0800010800.hg.1	TC0800010800.hg.1	NM_153225	SBSPON	157869	49.009	1.43E-07	6.8453	3.87E-07	ctrl - P10; ctrl - P11; ctrl - P2; ctrl - P8; P11 - P10; P2 - P10; P3 - P10; P8 - P10; P11 - P8; P3 - P2; P2 - P8; P3 - P8
TC1500010800.hg.1	TC1500010800.hg.1	NM_000693	ALDH1A3	220	48.974	1.43E-07	6.8436	3.88E-07	P10 - ctrl; ctrl - P2; P3 - ctrl; P8 - ctrl; P10 - P11; P10 - P2; P8 - P10; P11 - P2; P3 - P11; P8 - P11; P3 - P2; P8 - P2; P8 - P3
TC0600012744.hg.1	TC0600012744.hg.1	uc063qjl.1	RNU6-117P	NA	48.938	1.44E-07	6.8418	3.89E-07	ctrl - P10; ctrl - P11; ctrl - P3; ctrl - P8; P2 - P10; P2 - P11; P2 - P3; P2 - P8
TC0800008423.hg.1	TC0800008423.hg.1	ENST00000518749.1	KB-1107E3.1		48.837	1.46E-07	6.8367	3.93E-07	P10 - ctrl; P8 - ctrl; P10 - P11; P10 - P2; P10 - P3; P10 - P8; P8 - P11; P8 - P2; P8 - P3
TC1700006630.hg.1	TC1700006630.hg.1	NM_001114974	SMTNL2	342527	48.781	1.47E-07	6.8339	3.96E-07	ctrl - P10; ctrl - P2; ctrl - P3; ctrl - P8; P11 - P10; P2 - P10; P10 - P8; P11 - P2; P11 - P3; P11 - P8; P2 - P3; P2 - P8
TC0500013392.hg.1	TC0500013392.hg.1	NM_001308173	CCNJL	79616	48.354	1.54E-07	6.8123	4.15E-07	P10 - ctrl; P11 - ctrl; ctrl - P2; P3 - ctrl; P8 - ctrl; P10 - P11; P10 - P2; P10 - P3; P11 - P2; P8 - P11; P3 - P2; P8 - P2; P8 - P3
TC0X00008335.hg.1	TC0X00008335.hg.1	poyly.aAug10-unspliced	poyly	NA	48.265	1.56E-07	6.8077	4.19E-07	ctrl - P10; ctrl - P11; P2 - ctrl; ctrl - P8; P2 - P10; P3 - P10; P2 - P11; P3 - P11; P11 - P8; P2 - P3; P2 - P8; P3 - P8
TC0500007684.hg.1	TC0500007684.hg.1	hsa_circ_0072797	TCONS_l2_00023407	NA	48.191	1.57E-07	6.804	4.22E-07	ctrl - P10; ctrl - P11; ctrl - P3; ctrl - P8; P11 - P10; P2 - P10; P3 - P10; P2 - P11; P11 - P8; P2 - P3; P2 - P8; P3 - P8
TC0400007751.hg.1	TC0400007751.hg.1	ENST00000507775	RP13-644M16.5	NA	48.135	1.58E-07	6.8011	4.25E-07	ctrl - P10; ctrl - P11; P2 - ctrl; ctrl - P8; P2 - P10; P3 - P10; P10 - P8; P2 - P11; P3 - P11; P11 - P8; P2 - P3; P2 - P8; P3 - P8
TC2000009970.hg.1	TC2000009970.hg.1	NM_001083910	SIRPB1	10326	47.87	1.63E-07	6.7876	4.38E-07	P10 - ctrl; P3 - ctrl; P8 - ctrl; P10 - P11; P10 - P2; P3 - P10; P2 - P11; P3 - P11; P8 - P11; P3 - P2; P8 - P2
TC0300013513.hg.1	TC0300013513.hg.1	NM_001134418	P3H2	55214	47.86	1.63E-07	6.7871	4.38E-07	ctrl - P10; ctrl - P2; ctrl - P3; ctrl - P8; P11 - P10; P3 - P10; P11 - P2; P11 - P3; P11 - P8; P3 - P2; P3 - P8
TC1000008495.hg.1	TC1000008495.hg.1	NM_001165979	PLCE1	51196	47.8	1.64E-07	6.784	4.40E-07	P10 - ctrl; P2 - ctrl; P3 - ctrl; P8 - ctrl; P10 - P11; P10 - P2; P10 - P3; P8 - P10; P2 - P11; P3 - P11; P8 - P11; P8 - P2; P8 - P3
TC1200012452.hg.1	TC1200012452.hg.1	ENST00000542821.1	RP11-989F5.3		47.792	1.65E-07	6.7836	4.40E-07	ctrl - P10; ctrl - P11; ctrl - P3; ctrl - P8; P2 - P10; P3 - P10; P10 - P8; P2 - P11; P3 - P11; P2 - P3; P2 - P8; P3 - P8
TC1100011611.hg.1	TC1100011611.hg.1	NM_173582	PGM2L1	283209	47.719	1.66E-07	6.7798	4.44E-07	P10 - ctrl; ctrl - P11; P3 - ctrl; P8 - ctrl; P10 - P11; P10 - P2; P2 - P11; P3 - P11; P8 - P11; P3 - P2; P8 - P2; P8 - P3
TC0300013071.hg.1	TC0300013071.hg.1	zyjeebu.aAug10-unspliced	zyjeebu	NA	47.653	1.67E-07	6.7764	4.47E-07	ctrl - P10; ctrl - P11; ctrl - P3; ctrl - P8; P11 - P10; P2 - P10; P10 - P8; P2 - P11; P11 - P3; P11 - P8; P2 - P3; P2 - P8; P3 - P8
TC0200009220.hg.1	TC0200009220.hg.1	snerveybo.aAug10-unspliced	snerveybo	NA	47.63	1.68E-07	6.7753	4.47E-07	ctrl - P10; ctrl - P11; ctrl - P3; ctrl - P8; P11 - P10; P2 - P10; P10 - P3; P10 - P8; P11 - P3; P11 - P8; P2 - P3; P2 - P8
TC0600007241.hg.1	TC0600007241.hg.1	LRRC16A.lAug10-unspliced	LRRC16A	NA	47.603	1.68E-07	6.7738	4.48E-07	ctrl - P10; ctrl - P11; ctrl - P3; ctrl - P8; P11 - P10; P2 - P10; P3 - P10; P2 - P11; P3 - P11; P11 - P8; P2 - P3; P2 - P8; P3 - P8
TC0200008628.hg.1	TC0200008628.hg.1	ENST00000430586.1	AC092168.2		47.595	1.68E-07	6.7734	4.48E-07	ctrl - P11; P2 - ctrl; P3 - ctrl; ctrl - P8; P10 - P11; P2 - P10; P3 - P10; P10 - P8; P2 - P11; P3 - P11; P2 - P8; P3 - P8
TC0100017314.hg.1	TC0100017314.hg.1	blydy.aAug10-unspliced	blydy	NA	47.559	1.69E-07	6.7716	4.50E-07	ctrl - P10; ctrl - P11; ctrl - P2; ctrl - P3; ctrl - P8; P11 - P10; P2 - P10; P11 - P2; P11 - P3; P11 - P8; P2 - P3; P2 - P8; P3 - P8
TC2200007980.hg.1	TC2200007980.hg.1	noheru.aAug10-unspliced	noheru	NA	47.525	1.70E-07	6.7698	4.51E-07	ctrl - P10; ctrl - P11; ctrl - P3; ctrl - P8; P2 - P10; P3 - P10; P8 - P10; P2 - P11; P3 - P11; P8 - P11; P2 - P3; P2 - P8
TC0100016891.hg.1	TC0100016891.hg.1	NM_001305792	KIF14	9928	47.477	1.71E-07	6.7674	4.53E-07	P10 - ctrl; ctrl - P11; P3 - ctrl; P8 - ctrl; P10 - P11; P10 - P2; P8 - P10; P3 - P11; P8 - P11; P3 - P2; P8 - P2; P8 - P3
TC1100009402.hg.1	TC1100009402.hg.1	NR_103862	LOC100507283	1.01E+08	47.415	1.72E-07	6.7641	4.56E-07	ctrl - P10; ctrl - P2; ctrl - P3; ctrl - P8; P11 - P10; P2 - P10; P3 - P10; P8 - P10; P11 - P2; P11 - P3; P11 - P8; P2 - P8
TC0100012421.hg.1	TC0100012421.hg.1	NR_027055	FAM41C	284593	47.412	1.72E-07	6.764	4.56E-07	ctrl - P10; ctrl - P11; P2 - ctrl; ctrl - P3; ctrl - P8; P11 - P10; P2 - P10; P3 - P10; P2 - P11; P11 - P8; P2 - P3; P2 - P8; P3 - P8
TC1200009726.hg.1	TC1200009726.hg.1	swawchu.aAug10-unspliced	swawchu	NA	47.345	1.74E-07	6.7605	4.59E-07	ctrl - P10; ctrl - P11; ctrl - P3; ctrl - P8; P11 - P10; P2 - P10; P3 - P10; P2 - P11; P11 - P8; P2 - P3; P2 - P8; P3 - P8
TC2000007239.hg.1	TC2000007239.hg.1	NM_001258329	EPB41L1	2036	47.14	1.78E-07	6.7499	4.70E-07	ctrl - P10; ctrl - P2; ctrl - P3; ctrl - P8; P11 - P10; P10 - P3; P11 - P2; P11 - P3; P11 - P8
TC0300013843.hg.1	TC0300013843.hg.1	NR_038264	ADAMTS9-AS2	1.01E+08	47.003	1.81E-07	6.7428	4.77E-07	ctrl - P10; ctrl - P2; ctrl - P3; ctrl - P8; P11 - P10; P2 - P10; P11 - P2; P11 - P3; P11 - P8; P2 - P3; P2 - P8
TC0100014228.hg.1	TC0100014228.hg.1	NM_001018054	LRP8	7804	46.761	1.86E-07	6.7301	4.91E-07	P10 - ctrl; ctrl - P2; P3 - ctrl; P8 - ctrl; P10 - P11; P10 - P2; P3 - P10; P8 - P10; P11 - P2; P3 - P11; P8 - P11; P3 - P2; P8 - P2
TC0600009999.hg.1	TC0600009999.hg.1	ENST00000405341	HSPE1P26	NA	46.722	1.87E-07	6.7281	4.93E-07	ctrl - P10; ctrl - P11; P2 - ctrl; ctrl - P3; ctrl - P8; P10 - P11; P2 - P10; P10 - P8; P2 - P11; P3 - P11; P11 - P8; P2 - P3; P2 - P8; P3 - P8
TC0X00007936.hg.1	TC0X00007936.hg.1	NM_006733	CENPI	2491	46.708	1.87E-07	6.7273	4.93E-07	P10 - ctrl; ctrl - P11; ctrl - P2; P3 - ctrl; P8 - ctrl; P10 - P11; P10 - P2; P8 - P10; P3 - P11; P8 - P11; P3 - P2; P8 - P2; P8 - P3
TC0X00007507.hg.1	TC0X00007507.hg.1	NM_004429	EFNB1	1947	46.699	1.88E-07	6.7268	4.93E-07	P10 - ctrl; P11 - ctrl; P3 - ctrl; P8 - ctrl; P10 - P11; P10 - P2; P8 - P10; P3 - P11; P8 - P11; P3 - P2; P8 - P2; P8 - P3
TC1800006590.hg.1	TC1800006590.hg.1	weeso.aAug10-unspliced	weeso	NA	46.694	1.88E-07	6.7265	4.93E-07	ctrl - P10; ctrl - P11; P2 - ctrl; ctrl - P3; ctrl - P8; P2 - P10; P3 - P10; P10 - P8; P2 - P11; P3 - P11; P11 - P8; P2 - P3; P2 - P8; P3 - P8
TC1100008489.hg.1	TC1100008489.hg.1	smyglawbu.aAug10-unspliced	smyglawbu	NA	46.679	1.88E-07	6.7258	4.93E-07	ctrl - P10; ctrl - P11; P2 - ctrl; ctrl - P3; ctrl - P8; P11 - P10; P2 - P10; P3 - P10; P2 - P11; P11 - P8; P2 - P3; P2 - P8; P3 - P8
TC1000011923.hg.1	TC1000011923.hg.1	NM_001145453	GFRA1	2674	46.604	1.90E-07	6.7218	4.97E-07	ctrl - P10; ctrl - P2; P3 - ctrl; P8 - ctrl; P3 - P10; P8 - P10; P3 - P11; P8 - P11; P3 - P2; P8 - P2; P3 - P8

TC1100008121.hg.1	TC1100008121.hg.1	hakora.aAug10-unspliced	hakora	NA	46.569	1.91E-07	6.72	4.98E-07	ctrl - P10; ctrl - P11; P2 - ctrl; ctrl - P3; ctrl - P8; P11 - P10; P2 - P10; P10 - P8; P2 - P11; P11 - P3; P11 - P8; P2 - P3; P2 - P8; P3 - P8
TC0800009429.hg.1	TC0800009429.hg.1	NM_005218	DEFB1	1672	46.56	1.91E-07	6.7195	4.98E-07	P10 - ctrl; P11 - ctrl; P2 - ctrl; P8 - ctrl; P10 - P2; P10 - P3; P11 - P2; P11 - P3; P8 - P2; P8 - P3
TC1700011456.hg.1	TC1700011456.hg.1	satama.aAug10-unspliced	satama	NA	46.558	1.91E-07	6.7194	4.98E-07	ctrl - P10; ctrl - P11; ctrl - P3; ctrl - P8; P2 - P10; P2 - P11; P11 - P8; P2 - P3; P2 - P8; P3 - P8
TC1500010108.hg.1	TC1500010108.hg.1	ENST00000602975.1	RP11-797A18.6		46.522	1.92E-07	6.7175	5.00E-07	ctrl - P10; ctrl - P11; ctrl - P3; ctrl - P8; P2 - P10; P3 - P10; P2 - P11; P11 - P8; P2 - P3; P2 - P8; P3 - P8
TC1200008849.hg.1	TC1200008849.hg.1	uc058tfd.1	AC144522.1	NA	46.501	1.92E-07	6.7164	5.01E-07	ctrl - P10; ctrl - P11; ctrl - P3; ctrl - P8; P2 - P10; P3 - P10; P2 - P11; P11 - P8; P2 - P3; P2 - P8; P3 - P8
TC2100007663.hg.1	TC2100007663.hg.1	NM_001130914	BTG3	10950	46.474	1.93E-07	6.715	5.02E-07	P10 - ctrl; ctrl - P2; P3 - ctrl; P8 - ctrl; P10 - P11; P10 - P2; P3 - P10; P8 - P10; P11 - P2; P3 - P11; P8 - P11; P3 - P2; P8 - P2
TC0400007901.hg.1	TC0400007901.hg.1	NM_020859	SHROOM3	57619	46.407	1.94E-07	6.7114	5.05E-07	P10 - ctrl; P11 - ctrl; ctrl - P2; P3 - ctrl; P8 - ctrl; P10 - P11; P10 - P2; P10 - P3; P11 - P2; P8 - P11; P3 - P2; P8 - P2; P8 - P3
TC0300006437.hg.1	TC0300006437.hg.1	NM_001253387	CHL1	10752	46.367	1.95E-07	6.7093	5.07E-07	ctrl - P10; ctrl - P11; ctrl - P2; ctrl - P3; ctrl - P8; P11 - P10; P11 - P2; P11 - P3; P11 - P8
TC1100012957.hg.1	TC1100012957.hg.1	NM_001199573	TRIM22	10346	46.334	1.96E-07	6.7076	5.09E-07	P10 - ctrl; ctrl - P11; ctrl - P2; P3 - ctrl; P8 - ctrl; P10 - P11; P10 - P2; P3 - P11; P8 - P11; P3 - P2; P8 - P2
TC0800011057.hg.1	TC0800011057.hg.1	swaly.aAug10-unspliced	swaly	NA	46.33	1.96E-07	6.7074	5.09E-07	ctrl - P10; ctrl - P11; P2 - ctrl; ctrl - P8; P2 - P10; P3 - P10; P10 - P8; P2 - P11; P3 - P11; P2 - P3; P2 - P8; P3 - P8
TC2200007795.hg.1	TC2200007795.hg.1	uc062fle.1	AL022328.1	NA	46.232	1.99E-07	6.7022	5.14E-07	P10 - ctrl; P11 - ctrl; P3 - ctrl; P8 - ctrl; P10 - P11; P10 - P2; P10 - P3; P10 - P8; P11 - P2; P3 - P2; P8 - P2; P8 - P3
TC1400009364.hg.1	TC1400009364.hg.1	NR_110416	LOC101927780	1.02E+08	46.073	2.02E-07	6.6937	5.24E-07	P10 - ctrl; ctrl - P11; ctrl - P2; P3 - ctrl; P8 - ctrl; P10 - P11; P10 - P2; P8 - P10; P3 - P11; P8 - P11; P3 - P2; P8 - P2; P8 - P3
TC0100011472.hg.1	TC0100011472.hg.1	NM_001122834	HHAT	55733	46.02	2.04E-07	6.691	5.26E-07	P10 - ctrl; P3 - ctrl; P8 - ctrl; P10 - P11; P10 - P2; P3 - P10; P8 - P10; P11 - P2; P3 - P11; P8 - P11; P3 - P2; P8 - P2
TC1500010463.hg.1	TC1500010463.hg.1	NM_001267580	PRC1	9055	45.934	2.06E-07	6.6863	5.32E-07	P10 - ctrl; ctrl - P11; ctrl - P2; P8 - ctrl; P10 - P11; P10 - P2; P10 - P3; P8 - P10; P3 - P11; P8 - P11; P3 - P2; P8 - P2; P8 - P3
TC1200010620.hg.1	TC1200010620.hg.1	ENST00000550468.2	RP11-161H23.5		45.867	2.08E-07	6.6827	5.35E-07	P10 - ctrl; P11 - ctrl; P3 - ctrl; P8 - ctrl; P10 - P11; P10 - P2; P10 - P3; P11 - P2; P3 - P11; P8 - P11; P3 - P2; P8 - P2; P8 - P3
TC1300007795.hg.1	TC1300007795.hg.1	NA	NA	NA	45.816	2.09E-07	6.6801	5.38E-07	ctrl - P10; ctrl - P11; ctrl - P3; ctrl - P8; P11 - P10; P2 - P10; P3 - P10; P2 - P11; P3 - P11; P11 - P8; P2 - P3; P2 - P8; P3 - P8
TC1200007567.hg.1	TC1200007567.hg.1	NM_012284	KCNH3	23416	45.815	2.09E-07	6.68	5.38E-07	ctrl - P10; ctrl - P11; ctrl - P2; ctrl - P3; ctrl - P8; P11 - P10; P2 - P10; P3 - P10; P11 - P2; P11 - P3; P11 - P8; P2 - P8; P3 - P8
TC0Y00006645.hg.1	TC0Y00006645.hg.1	stuby.aAug10-unspliced	stuby	NA	45.707	2.12E-07	6.6742	5.44E-07	ctrl - P10; ctrl - P11; ctrl - P3; ctrl - P8; P11 - P10; P2 - P10; P3 - P10; P2 - P11; P3 - P11; P11 - P8; P2 - P8; P3 - P8
TC0200008538.hg.1	TC0200008538.hg.1	leekorbu.aAug10-unspliced	leekorbu	NA	45.654	2.13E-07	6.6714	5.47E-07	ctrl - P10; ctrl - P11; P2 - ctrl; ctrl - P8; P11 - P10; P2 - P10; P3 - P10; P2 - P11; P3 - P11; P11 - P8; P2 - P3; P2 - P8; P3 - P8
TC1300009729.hg.1	TC1300009729.hg.1	reenoy.aAug10-unspliced	reenoy	NA	45.605	2.14E-07	6.6687	5.50E-07	ctrl - P10; ctrl - P11; P2 - ctrl; ctrl - P8; P11 - P10; P2 - P10; P3 - P10; P2 - P11; P11 - P8; P2 - P3; P2 - P8; P3 - P8
TC0X00010098.hg.1	TC0X00010098.hg.1	NM_001008537	KIAA2022	340533	45.286	2.23E-07	6.6516	5.72E-07	ctrl - P10; ctrl - P2; ctrl - P3; ctrl - P8; P11 - P10; P8 - P10; P11 - P2; P11 - P3; P11 - P8; P3 - P2; P8 - P2
TC1200011574.hg.1	TC1200011574.hg.1	NM_018351	FGD6	55785	45.276	2.23E-07	6.651	5.72E-07	P10 - ctrl; P3 - ctrl; P8 - ctrl; P10 - P11; P10 - P2; P3 - P10; P8 - P10; P3 - P11; P8 - P11; P3 - P2; P8 - P2; P3 - P8
TC1800008899.hg.1	TC1800008899.hg.1	smeebor.aAug10-unspliced	smeebor	NA	45.219	2.25E-07	6.6479	5.75E-07	ctrl - P10; ctrl - P11; P2 - ctrl; ctrl - P3; ctrl - P8; P2 - P10; P10 - P8; P2 - P11; P11 - P8; P2 - P3; P2 - P8; P3 - P8
TC0800008080.hg.1	TC0800008080.hg.1	NM_001444	FABP5	2171	45.214	2.25E-07	6.6477	5.75E-07	ctrl - P10; ctrl - P11; P3 - ctrl; ctrl - P8; P11 - P10; P2 - P10; P3 - P10; P2 - P11; P3 - P11; P3 - P2; P2 - P8; P3 - P8
TC0700013428.hg.1	TC0700013428.hg.1	NM_178238	PILRB	29990	45.153	2.27E-07	6.6444	5.79E-07	ctrl - P10; ctrl - P11; ctrl - P2; ctrl - P3; ctrl - P8; P11 - P10; P2 - P10; P3 - P10; P2 - P11; P11 - P8; P2 - P3; P2 - P8; P3 - P8
TC0100015705.hg.1	TC0100015705.hg.1	NA	NA	NA	45.096	2.28E-07	6.6413	5.82E-07	P10 - ctrl; P11 - ctrl; P8 - ctrl; P10 - P11; P10 - P2; P10 - P3; P11 - P2; P11 - P3; P3 - P2; P8 - P2; P8 - P3
TC0900011148.hg.1	TC0900011148.hg.1	NM_001286974	CTNNAL1	8727	45.086	2.29E-07	6.6407	5.83E-07	P10 - ctrl; ctrl - P2; P3 - ctrl; P8 - ctrl; P10 - P11; P10 - P2; P3 - P10; P8 - P10; P11 - P2; P3 - P11; P8 - P11; P3 - P2; P8 - P2
TC1200011753.hg.1	TC1200011753.hg.1	NA	NA	NA	44.925	2.33E-07	6.632	5.94E-07	P10 - ctrl; P11 - ctrl; P3 - ctrl; P8 - ctrl; P10 - P11; P10 - P2; P10 - P3; P11 - P2; P8 - P11; P3 - P2; P8 - P2; P8 - P3
TC0400008487.hg.1	TC0400008487.hg.1	spoklawby.cAug10-unspliced	spoklawby	NA	44.92	2.34E-07	6.6317	5.94E-07	ctrl - P10; ctrl - P11; ctrl - P3; ctrl - P8; P11 - P10; P2 - P10; P2 - P11; P11 - P3; P11 - P8; P2 - P3; P2 - P8
TC0100017732.hg.1	TC0100017732.hg.1	ENST00000416221.1	RP11-295G20.2		44.883	2.35E-07	6.6297	5.96E-07	P10 - ctrl; P11 - ctrl; P3 - ctrl; P8 - ctrl; P10 - P11; P10 - P2; P3 - P10; P8 - P10; P11 - P2; P3 - P11; P8 - P11; P3 - P2; P8 - P2
TC0400006478.hg.1	TC0400006478.hg.1	NA	NA	NA	44.809	2.37E-07	6.6257	6.00E-07	ctrl - P10; ctrl - P11; ctrl - P2; ctrl - P3; ctrl - P8; P2 - P10; P3 - P10; P2 - P11; P2 - P3; P2 - P8; P3 - P8
TC0800011912.hg.1	TC0800011912.hg.1	NA	NA	NA	44.808	2.37E-07	6.6256	6.00E-07	ctrl - P10; ctrl - P11; ctrl - P3; ctrl - P8; P11 - P10; P2 - P10; P2 - P11; P11 - P3; P11 - P8; P2 - P3; P2 - P8; P3 - P8
TC1100013201.hg.1	TC1100013201.hg.1	ENST00000542515	ORAOV1	220064	44.797	2.37E-07	6.625	6.00E-07	P10 - ctrl; P11 - ctrl; ctrl - P2; P3 - ctrl; P8 - ctrl; P10 - P11; P10 - P2; P11 - P2; P3 - P11; P8 - P11; P3 - P2; P8 - P2

TC0600012434.hg.1	TC0600012434.hg.1	NM_002395	ME1	4199	44.701	2.40E-07	6.6198	6.07E-07	P10 - ctrl; ctrl - P11; ctrl - P2; P3 - ctrl; P8 - ctrl; P10 - P11; P10 - P2; P11 - P2; P3 - P11; P8 - P11; P3 - P2; P8 - P2
TC0800007362.hg.1	TC0800007362.hg.1	NM_021623	PLEKHA2	59339	44.67	2.41E-07	6.6181	6.09E-07	P10 - ctrl; ctrl - P2; P8 - ctrl; P10 - P11; P10 - P2; P10 - P3; P11 - P2; P8 - P11; P3 - P2; P8 - P2; P8 - P3
TC0300008740.hg.1	TC0300008740.hg.1	zerborbu.aAug10-unspliced	zerborbu	NA	44.647	2.42E-07	6.6168	6.10E-07	ctrl - P10; ctrl - P11; P2 - ctrl; ctrl - P3; ctrl - P8; P2 - P10; P3 - P10; P2 - P11; P3 - P11; P11 - P8; P2 - P3; P2 - P8; P3 - P8
TC0300013665.hg.1	TC0300013665.hg.1	ENST00000308466	MUC4	4585	44.618	2.43E-07	6.6152	6.12E-07	P10 - ctrl; P8 - ctrl; P10 - P11; P10 - P2; P10 - P3; P8 - P11; P8 - P2; P8 - P3
TC1400007585.hg.1	TC1400007585.hg.1	NR_125769	LINC01269	1.04E+08	44.618	2.43E-07	6.6148	6.12E-07	P10 - ctrl; P3 - ctrl; P8 - ctrl; P10 - P11; P10 - P2; P3 - P10; P8 - P10; P3 - P11; P8 - P11; P3 - P2; P8 - P2; P3 - P8
TC0200012082.hg.1	TC0200012082.hg.1	NM_015662	IFT172	26160	44.576	2.44E-07	6.6129	6.14E-07	ctrl - P10; ctrl - P2; ctrl - P3; ctrl - P8; P11 - P10; P2 - P10; P10 - P3; P11 - P2; P11 - P3; P11 - P8; P2 - P3; P2 - P8
TC0200015618.hg.1	TC0200015618.hg.1	veygorbo.aAug10-unspliced	veygorbo	NA	44.445	2.48E-07	6.6057	6.23E-07	ctrl - P10; ctrl - P3; ctrl - P8; P11 - P10; P2 - P10; P10 - P8; P11 - P3; P11 - P8; P2 - P3; P2 - P8
TC0500013185.hg.1	TC0500013185.hg.1	ENST00000425529	RP11-497H16.7		43.946	2.64E-07	6.5781	6.63E-07	ctrl - P10; ctrl - P11; ctrl - P3; ctrl - P8; P11 - P10; P2 - P10; P3 - P10; P2 - P11; P11 - P8; P2 - P3; P2 - P8; P3 - P8
TC0300011979.hg.1	TC0300011979.hg.1	NM_024508	ZBED2	79413	43.917	2.65E-07	6.5765	6.65E-07	P10 - ctrl; P3 - ctrl; P8 - ctrl; P10 - P11; P10 - P2; P3 - P11; P8 - P11; P3 - P2; P8 - P2
TC1500009610.hg.1	TC1500009610.hg.1	veysorbo.aAug10-unspliced	veysorbo	NA	43.812	2.69E-07	6.5707	6.74E-07	ctrl - P10; ctrl - P11; P2 - ctrl; ctrl - P8; P10 - P11; P2 - P10; P3 - P10; P10 - P8; P2 - P11; P3 - P11; P11 - P8; P2 - P3; P2 - P8; P3 - P8
TC1300008930.hg.1	TC1300008930.hg.1	chorwarby.aAug10-unspliced	chorwarby	NA	43.744	2.71E-07	6.5669	6.79E-07	ctrl - P10; ctrl - P11; ctrl - P8; P2 - P10; P3 - P10; P10 - P8; P2 - P11; P3 - P11; P11 - P8; P2 - P3; P2 - P8; P3 - P8
TC1000012227.hg.1	TC1000012227.hg.1	NM_152311	CLRN3	119467	43.725	2.72E-07	6.5659	6.80E-07	P10 - ctrl; P11 - ctrl; P2 - ctrl; P3 - ctrl; P8 - ctrl; P10 - P11; P10 - P2; P10 - P3; P10 - P8; P11 - P3; P11 - P8; P2 - P3; P2 - P8; P8 - P3
TC0200007399.hg.1	TC0200007399.hg.1	NM_172069	PLEKHH2	130271	43.628	2.75E-07	6.5604	6.87E-07	ctrl - P10; ctrl - P11; ctrl - P3; ctrl - P8; P2 - P10; P2 - P11; P2 - P3; P2 - P8
TC0400008772.hg.1	TC0400008772.hg.1	NR_038380	SLC7A11-AS1	641364	43.624	2.75E-07	6.5602	6.87E-07	P10 - ctrl; P3 - ctrl; P8 - ctrl; P10 - P11; P10 - P2; P3 - P10; P11 - P2; P3 - P11; P8 - P11; P3 - P2; P8 - P2; P3 - P8
TC0100009850.hg.1	TC0100009850.hg.1	ENST00000466343.2	RP4-791M13.3		43.6	2.76E-07	6.5589	6.89E-07	ctrl - P10; ctrl - P11; ctrl - P3; ctrl - P8; P11 - P10; P2 - P10; P3 - P10; P2 - P11; P11 - P3; P11 - P8; P2 - P3; P2 - P8; P3 - P8
TC0100007841.hg.1	TC0100007841.hg.1	NM_001256875	CDCA8	55143	43.571	2.77E-07	6.5572	6.91E-07	P10 - ctrl; ctrl - P2; P3 - ctrl; P8 - ctrl; P10 - P11; P10 - P2; P10 - P3; P8 - P10; P3 - P11; P8 - P11; P3 - P2; P8 - P2; P8 - P3
TC1500007511.hg.1	TC1500007511.hg.1	garstaw.aAug10-unspliced	garstaw	NA	43.534	2.79E-07	6.5552	6.93E-07	ctrl - P10; ctrl - P11; ctrl - P3; ctrl - P8; P10 - P11; P2 - P10; P3 - P10; P2 - P11; P3 - P11; P2 - P3; P2 - P8; P3 - P8
TC0200009626.hg.1	TC0200009626.hg.1	NM_001195685	LYPD6	2q23.2	43.51	2.79E-07	6.5538	6.95E-07	ctrl - P10; P11 - ctrl; ctrl - P2; ctrl - P3; ctrl - P8; P11 - P10; P8 - P10; P11 - P2; P11 - P3; P11 - P8; P8 - P2; P8 - P3
TC1800008997.hg.1	TC1800008997.hg.1	NR_038325	LINC01541	1.01E+08	43.418	2.83E-07	6.5486	7.02E-07	P10 - ctrl; P3 - ctrl; P8 - ctrl; P10 - P11; P10 - P2; P10 - P3; P2 - P11; P3 - P11; P8 - P11; P8 - P2; P8 - P3
TC0400012972.hg.1	TC0400012972.hg.1	NM_020724	RNF150	57484	43.416	2.83E-07	6.5486	7.02E-07	ctrl - P10; ctrl - P11; ctrl - P2; ctrl - P3; ctrl - P8; P11 - P10; P3 - P10; P11 - P2; P11 - P3; P11 - P8
TC0800009782.hg.1	TC0800009782.hg.1	NM_001130518	CSGALNACT1	55790	43.396	2.84E-07	6.5474	7.02E-07	P10 - ctrl; P11 - ctrl; P2 - ctrl; P3 - ctrl; P8 - ctrl; P10 - P11; P10 - P2; P10 - P3; P11 - P2; P8 - P11; P3 - P2; P8 - P2; P8 - P3
TC0100011099.hg.1	TC0100011099.hg.1	kleyplu.aAug10-unspliced	kleyplu	NA	43.396	2.84E-07	6.5474	7.02E-07	ctrl - P10; ctrl - P11; P2 - ctrl; ctrl - P3; ctrl - P8; P11 - P10; P2 - P10; P3 - P10; P10 - P8; P2 - P11; P11 - P8; P2 - P3; P2 - P8; P3 - P8
TC1600009427.hg.1	TC1600009427.hg.1	LOC729978.dAug10	LOC729978		43.271	2.88E-07	6.5404	7.13E-07	ctrl - P10; ctrl - P11; ctrl - P3; ctrl - P8; P11 - P10; P2 - P10; P3 - P10; P8 - P10; P2 - P11; P11 - P8; P2 - P3; P2 - P8; P3 - P8
TC0600011460.hg.1	TC0600011460.hg.1	LOC100287365.aAug10-unspliced	LOC100287365	NA	43.24	2.89E-07	6.5386	7.15E-07	P10 - ctrl; P11 - ctrl; ctrl - P2; P3 - ctrl; P8 - ctrl; P10 - P11; P10 - P2; P10 - P3; P10 - P8; P11 - P2; P11 - P3; P3 - P2; P8 - P2; P8 - P3
TC1500006568.hg.1	TC1500006568.hg.1	jeezaw.aAug10-unspliced	jeezaw	NA	43.234	2.90E-07	6.5383	7.15E-07	ctrl - P10; ctrl - P11; P2 - ctrl; ctrl - P3; ctrl - P8; P2 - P10; P10 - P8; P2 - P11; P11 - P8; P2 - P3; P2 - P8; P3 - P8
TC1900011960.hg.1	TC1900011960.hg.1	NM_021016	PSG3	5671	43.143	2.93E-07	6.5331	7.23E-07	P10 - ctrl; P3 - ctrl; P8 - ctrl; P10 - P11; P10 - P2; P3 - P10; P8 - P10; P3 - P11; P8 - P11; P3 - P2; P8 - P2
TC0400011646.hg.1	TC0400011646.hg.1	smusnawby.aAug10-unspliced	smusnawby	NA	43.086	2.95E-07	6.5299	7.27E-07	ctrl - P10; ctrl - P11; ctrl - P3; ctrl - P8; P11 - P10; P2 - P10; P3 - P10; P2 - P11; P3 - P11; P11 - P8; P2 - P3; P2 - P8; P3 - P8
TC0200014765.hg.1	TC0200014765.hg.1	DPP4.nAug10-unspliced	DPP4	NA	42.958	3.00E-07	6.5227	7.39E-07	ctrl - P10; ctrl - P3; ctrl - P8; P11 - P10; P2 - P10; P3 - P10; P10 - P8; P11 - P8; P2 - P8; P3 - P8
TC0X00010083.hg.1	TC0X00010083.hg.1	NR_030398	MIR421	693122	42.955	3.00E-07	6.5225	7.39E-07	ctrl - P10; ctrl - P11; P2 - ctrl; ctrl - P3; ctrl - P8; P2 - P10; P3 - P10; P2 - P11; P11 - P8; P2 - P3; P2 - P8; P3 - P8
TC0100014852.hg.1	TC0100014852.hg.1	NM_002053	GBP1	2633	42.913	3.02E-07	6.5201	7.41E-07	P10 - ctrl; ctrl - P11; ctrl - P2; P8 - ctrl; P10 - P11; P10 - P2; P10 - P3; P3 - P11; P8 - P11; P3 - P2; P8 - P2; P8 - P3
TC1600011465.hg.1	TC1600011465.hg.1	NR_040023	ERVK13-1	1.01E+08	42.912	3.02E-07	6.52	7.41E-07	ctrl - P10; ctrl - P11; P2 - ctrl; ctrl - P3; ctrl - P8; P11 - P10; P2 - P10; P2 - P11; P11 - P3; P11 - P8; P2 - P3; P2 - P8; P3 - P8
TC1800009245.hg.1	TC1800009245.hg.1	NM_001031848	SERPINB8	5271	42.9	3.02E-07	6.5193	7.42E-07	P10 - ctrl; P3 - ctrl; P8 - ctrl; P10 - P11; P10 - P2; P3 - P11; P8 - P11; P3 - P2; P8 - P2
TC0200008502.hg.1	TC0200008502.hg.1	NM_001281710	NCAPH	23397	42.883	3.03E-07	6.5184	7.43E-07	P10 - ctrl; ctrl - P11; ctrl - P2; P8 - ctrl; P10 - P11; P10 - P2; P10 - P3; P8 - P10; P3 - P11; P8 - P11; P3 - P2; P8 - P2; P8 - P3
TC0500012373.hg.1	TC0500012373.hg.1	basteyby.aAug10-unspliced	basteyby	NA	42.869	3.04E-07	6.5176	7.43E-07	ctrl - P10; ctrl - P11; ctrl - P3; ctrl - P8; P11 - P10; P2 - P10; P3 - P10; P2 - P11; P11 - P3; P11 - P8; P2 - P3; P2 - P8

TC1500009071.hg.1	TC1500009071.hg.1	ENST00000560769.1	CTD-2033D15.1		42.833	3.05E-07	6.5156	7.46E-07	P10 - ctrl; ctrl - P2; P3 - ctrl; P8 - ctrl; P10 - P11; P10 - P2; P8 - P10; P3 - P11; P8 - P11; P3 - P2; P8 - P2
TC0500007050.hg.1	TC0500007050.hg.1	NM_178140	PDZD2	23037	42.806	3.06E-07	6.514	7.48E-07	P10 - ctrl; ctrl - P2; P8 - ctrl; P10 - P11; P10 - P2; P10 - P3; P11 - P2; P8 - P11; P8 - P2; P8 - P3
TC0200014718.hg.1	TC0200014718.hg.1	meydo.aAug10-unspliced	meydo	NA	42.769	3.08E-07	6.5119	7.51E-07	ctrl - P10; ctrl - P11; ctrl - P3; ctrl - P8; P11 - P10; P2 - P10; P2 - P11; P11 - P3; P11 - P8; P2 - P3; P2 - P8
TC0900008395.hg.1	TC0900008395.hg.1	NA	NA	NA	42.615	3.14E-07	6.5031	7.66E-07	ctrl - P10; ctrl - P11; ctrl - P3; ctrl - P8; P11 - P10; P2 - P10; P3 - P10; P10 - P8; P2 - P11; P11 - P8; P2 - P3; P2 - P8; P3 - P8
TC0600007610.hg.1	TC0600007610.hg.1	NM_002441	MSH5	4439	42.585	3.15E-07	6.5014	7.68E-07	ctrl - P10; ctrl - P11; ctrl - P3; ctrl - P8; P11 - P10; P2 - P10; P3 - P10; P8 - P10; P2 - P11; P11 - P8; P2 - P3; P2 - P8; P3 - P8
TC0X00008056.hg.1	TC0X00008056.hg.1	NM_001171092	CLDN2	9075	42.58	3.15E-07	6.5011	7.68E-07	ctrl - P10; P2 - ctrl; P3 - ctrl; ctrl - P8; P11 - P10; P2 - P10; P3 - P10; P8 - P10; P2 - P11; P11 - P8; P2 - P8; P3 - P8
TC1500006785.hg.1	TC1500006785.hg.1	NM_001039841	ARHGAP11B	89839	42.559	3.16E-07	6.4999	7.69E-07	ctrl - P11; ctrl - P2; ctrl - P3; P8 - ctrl; P10 - P11; P10 - P2; P10 - P3; P8 - P10; P2 - P11; P3 - P11; P8 - P11; P8 - P2; P8 - P3
TC0800009178.hg.1	TC0800009178.hg.1	NR_126398	C8orf31	286122	42.541	3.17E-07	6.4989	7.70E-07	P10 - ctrl; P11 - ctrl; P3 - ctrl; P8 - ctrl; P10 - P11; P10 - P2; P11 - P2; P3 - P11; P8 - P11; P3 - P2; P8 - P2; P8 - P3
TC1200009179.hg.1	TC1200009179.hg.1	paforby.aAug10-unspliced	paforby	NA	42.384	3.24E-07	6.4899	7.85E-07	P10 - ctrl; P11 - ctrl; P2 - ctrl; P3 - ctrl; P8 - ctrl; P10 - P11; P10 - P2; P10 - P3; P11 - P2; P8 - P11; P3 - P2; P8 - P2; P8 - P3
TC1000008995.hg.1	TC1000008995.hg.1	NM_001276282	ATRNL1	26033	42.314	3.27E-07	6.4859	7.92E-07	P10 - ctrl; ctrl - P11; ctrl - P3; P8 - ctrl; P10 - P11; P10 - P2; P10 - P3; P10 - P8; P2 - P11; P8 - P11; P2 - P3; P8 - P2; P8 - P3
TC0300007190.hg.1	TC0300007190.hg.1	swerfawbo.aAug10-unspliced	swerfawbo	NA	42.273	3.28E-07	6.4835	7.95E-07	ctrl - P10; ctrl - P11; ctrl - P3; ctrl - P8; P11 - P10; P2 - P10; P3 - P10; P2 - P11; P11 - P8; P2 - P3; P2 - P8; P3 - P8
TC1100012787.hg.1	TC1100012787.hg.1	staglybu.aAug10-unspliced	staglybu	NA	42.208	3.31E-07	6.4797	8.02E-07	ctrl - P10; ctrl - P2; ctrl - P3; ctrl - P8; P11 - P10; P10 - P3; P11 - P2; P11 - P3; P11 - P8; P2 - P3
TC1500009348.hg.1	TC1500009348.hg.1	NM_000138	FBN1	2200	42.155	3.34E-07	6.4767	8.06E-07	P10 - ctrl; P3 - ctrl; P8 - ctrl; P10 - P11; P10 - P2; P3 - P10; P8 - P10; P11 - P2; P3 - P11; P8 - P11; P3 - P2; P8 - P2
TC1100011766.hg.1	TC1100011766.hg.1	NA	NA	NA	42.146	3.34E-07	6.4761	8.07E-07	ctrl - P10; ctrl - P11; ctrl - P8; P11 - P10; P2 - P10; P3 - P10; P3 - P11; P11 - P8; P2 - P8; P3 - P8
TC0700008617.hg.1	TC0700008617.hg.1	NM_020979	SH2B2	10603	42.136	3.35E-07	6.4756	8.07E-07	P10 - ctrl; P11 - ctrl; P3 - ctrl; P8 - ctrl; P10 - P11; P10 - P2; P10 - P3; P11 - P2; P8 - P11; P3 - P2; P8 - P2; P8 - P3
TC1500010018.hg.1	TC1500010018.hg.1	NM_001146029	SEMA7A	8482	42.042	3.39E-07	6.4701	8.16E-07	P10 - ctrl; P3 - ctrl; P8 - ctrl; P10 - P11; P10 - P2; P8 - P10; P3 - P11; P8 - P11; P3 - P2; P8 - P2; P8 - P3
TC0100012299.hg.1	TC0100012299.hg.1	NA	NA	NA	42.034	3.39E-07	6.4697	8.16E-07	ctrl - P10; ctrl - P11; P2 - ctrl; ctrl - P3; ctrl - P8; P2 - P10; P10 - P8; P2 - P11; P11 - P8; P2 - P3; P2 - P8
TC0500009035.hg.1	TC0500009035.hg.1	NM_001040129	SPINK13	153218	41.992	3.41E-07	6.4672	8.20E-07	ctrl - P10; ctrl - P11; ctrl - P2; P3 - ctrl; ctrl - P8; P3 - P10; P3 - P11; P3 - P2; P3 - P8
TC0700007001.hg.1	TC0700007001.hg.1	NR_122069	HOXA-AS2	285943	41.943	3.43E-07	6.4644	8.25E-07	ctrl - P10; ctrl - P2; ctrl - P3; ctrl - P8; P11 - P10; P10 - P3; P11 - P2; P11 - P3; P11 - P8; P2 - P3; P8 - P3
TC0200012917.hg.1	TC0200012917.hg.1	NA	NA	NA	41.936	3.44E-07	6.464	8.25E-07	ctrl - P10; ctrl - P2; P3 - ctrl; ctrl - P8; P11 - P10; P2 - P10; P3 - P10; P11 - P2; P11 - P8; P3 - P2; P2 - P8; P3 - P8
TC2000009295.hg.1	TC2000009295.hg.1	sperpey.aAug10-unspliced	sperpey	NA	41.895	3.45E-07	6.4616	8.29E-07	ctrl - P10; ctrl - P11; ctrl - P3; ctrl - P8; P2 - P10; P3 - P10; P10 - P8; P2 - P11; P3 - P11; P11 - P8; P2 - P3; P2 - P8; P3 - P8
TC0500012567.hg.1	TC0500012567.hg.1	NM_032385	FAXDC2	10826	41.867	3.47E-07	6.46	8.30E-07	ctrl - P10; ctrl - P2; ctrl - P3; ctrl - P8; P11 - P10; P2 - P10; P3 - P10; P8 - P10; P11 - P2; P11 - P3; P11 - P8; P2 - P3; P2 - P8
TC0600007700.hg.1	TC0600007700.hg.1	NM_002263	KIFC1	3833	41.863	3.47E-07	6.4598	8.30E-07	P10 - ctrl; ctrl - P11; ctrl - P2; P8 - ctrl; P10 - P11; P10 - P2; P10 - P3; P8 - P10; P3 - P11; P8 - P11; P3 - P2; P8 - P2; P8 - P3
TC1400006437.hg.1	TC1400006437.hg.1	NA	NA	NA	41.849	3.48E-07	6.4589	8.31E-07	ctrl - P11; P2 - ctrl; P3 - ctrl; ctrl - P8; P10 - P11; P2 - P10; P3 - P10; P10 - P8; P2 - P11; P3 - P11; P2 - P3; P2 - P8; P3 - P8
TC0700008503.hg.1	TC0700008503.hg.1	uc032zyw.2	AC069294.1	NA	41.803	3.50E-07	6.4562	8.36E-07	ctrl - P10; ctrl - P11; P2 - ctrl; ctrl - P3; ctrl - P8; P11 - P10; P2 - P10; P3 - P10; P2 - P11; P11 - P8; P2 - P3; P2 - P8
TC1600007880.hg.1	TC1600007880.hg.1	uc059uez.1	snoU13	NA	41.677	3.56E-07	6.4489	8.49E-07	ctrl - P10; ctrl - P11; ctrl - P3; ctrl - P8; P11 - P10; P2 - P10; P10 - P3; P2 - P11; P11 - P3; P11 - P8; P2 - P3; P2 - P8
TC0100008517.hg.1	TC0100008517.hg.1	NM_001134673	NFIA	1p31.3-p31.2	41.609	3.59E-07	6.4449	8.56E-07	ctrl - P10; P11 - ctrl; ctrl - P2; ctrl - P3; ctrl - P8; P11 - P10; P2 - P10; P3 - P10; P8 - P10; P11 - P2; P11 - P3; P11 - P8; P2 - P8; P3 - P8
TC0600011515.hg.1	TC0600011515.hg.1	ENST00000580587.1	XXbac-BPG181M17.6		41.532	3.63E-07	6.4404	8.64E-07	P10 - ctrl; P11 - ctrl; P3 - ctrl; P8 - ctrl; P10 - P2; P10 - P3; P11 - P2; P11 - P3; P3 - P2; P8 - P2; P8 - P3
TC0500010540.hg.1	TC0500010540.hg.1	NM_001127671	LIFR	3977	41.478	3.65E-07	6.4372	8.70E-07	ctrl - P10; ctrl - P11; ctrl - P2; ctrl - P3; ctrl - P8; P11 - P10; P2 - P10; P3 - P10; P10 - P8; P11 - P3; P11 - P8; P2 - P8; P3 - P8
TC1500007815.hg.1	TC1500007815.hg.1	gawzoybu.aAug10-unspliced	gawzoybu	NA	41.424	3.68E-07	6.4341	8.75E-07	ctrl - P10; ctrl - P11; P2 - ctrl; ctrl - P3; ctrl - P8; P11 - P10; P2 - P10; P3 - P10; P10 - P8; P2 - P11; P11 - P8; P2 - P3; P2 - P8; P3 - P8
TC0200016583.hg.1	TC0200016583.hg.1	NR_131917	LOC105747689	1.06E+08	41.365	3.71E-07	6.4306	8.81E-07	ctrl - P10; ctrl - P11; ctrl - P8; P2 - P10; P3 - P10; P2 - P11; P3 - P11; P2 - P3; P2 - P8; P3 - P8
TC2200007496.hg.1	TC2200007496.hg.1	jeybar.aAug10-unspliced	jeybar	NA	41.284	3.75E-07	6.4259	8.90E-07	ctrl - P10; ctrl - P11; ctrl - P3; ctrl - P8; P11 - P10; P2 - P10; P3 - P10; P10 - P8; P2 - P11; P11 - P8; P2 - P3; P2 - P8; P3 - P8
TC1100011933.hg.1	TC1100011933.hg.1	TUBA1B.1.aAug10	TUBA1B.1	NA	41.275	3.76E-07	6.4254	8.90E-07	P10 - ctrl; P11 - ctrl; P3 - ctrl; P8 - ctrl; P10 - P11; P10 - P2; P10 - P3; P8 - P11; P8 - P2; P8 - P3

TC0300013336.hg.1	TC0300013336.hg.1	NM_014398	LAMP3	27074	41.17	3.81E-07	6.4191	9.02E-07	P10 - ctrl; P11 - ctrl; P3 - ctrl; P8 - ctrl; P10 - P2; P8 - P10; P11 - P2; P3 - P11; P8 - P11; P3 - P2; P8 - P2
TC0200011902.hg.1	TC0200011902.hg.1	chaneebo.aAug10-unspliced	chaneebo	NA	41.159	3.82E-07	6.4185	9.03E-07	ctrl - P10; ctrl - P11; ctrl - P3; ctrl - P8; P11 - P10; P2 - P10; P10 - P8; P2 - P11; P11 - P8; P2 - P3; P2 - P8; P3 - P8
TC0400007806.hg.1	TC0400007806.hg.1	NM_001144756	NPFFR2	10886	41.153	3.82E-07	6.4181	9.03E-07	P10 - ctrl; ctrl - P2; P3 - ctrl; P8 - ctrl; P10 - P11; P10 - P2; P8 - P10; P11 - P2; P3 - P11; P8 - P11; P3 - P2; P8 - P2; P8 - P3
TC0700008073.hg.1	TC0700008073.hg.1	NM_000941	POR	5447	41.1	3.85E-07	6.415	9.08E-07	P10 - ctrl; P11 - ctrl; ctrl - P2; P3 - ctrl; P8 - ctrl; P10 - P11; P10 - P2; P10 - P3; P10 - P8; P11 - P2; P8 - P11; P3 - P2; P8 - P2; P8 - P3
TC1100010747.hg.1	TC1100010747.hg.1	gawny.aAug10-unspliced	gawny	NA	41.098	3.85E-07	6.4148	9.08E-07	ctrl - P10; ctrl - P11; ctrl - P3; ctrl - P8; P2 - P10; P3 - P10; P2 - P11; P3 - P11; P2 - P3; P2 - P8; P3 - P8
TC1500006580.hg.1	TC1500006580.hg.1	NR_003294	SNORD115-2	15q11.2	41.063	3.87E-07	6.4128	9.11E-07	P10 - ctrl; P3 - ctrl; P8 - ctrl; P10 - P11; P10 - P2; P3 - P10; P3 - P11; P8 - P11; P3 - P2; P8 - P2; P3 - P8
TC0500011072.hg.1	TC0500011072.hg.1	chostarby.aAug10-unspliced	chostarby	NA	41.06	3.87E-07	6.4126	9.11E-07	ctrl - P10; ctrl - P11; ctrl - P3; ctrl - P8; P11 - P10; P2 - P10; P2 - P11; P11 - P8; P2 - P3; P2 - P8; P3 - P8
TC0600011112.hg.1	TC0600011112.hg.1	NM_005074	SLC17A1	6568	40.987	3.91E-07	6.4083	9.19E-07	ctrl - P10; ctrl - P11; P2 - ctrl; P3 - ctrl; ctrl - P8; P11 - P10; P2 - P10; P3 - P10; P2 - P11; P3 - P11; P2 - P8; P3 - P8
TC2000009922.hg.1	TC2000009922.hg.1	NM_001199264	SGK2	20q13.2	40.889	3.96E-07	6.4025	9.31E-07	ctrl - P10; P11 - ctrl; ctrl - P2; ctrl - P3; ctrl - P8; P11 - P10; P2 - P10; P3 - P10; P11 - P2; P11 - P3; P11 - P8; P3 - P2; P3 - P8
HTA2-pos-2985912_st	23150249	NA	NA	NA	40.815	4.00E-07	6.3981	9.39E-07	ctrl - P10; ctrl - P11; ctrl - P3; ctrl - P8; P11 - P10; P2 - P10; P3 - P10; P2 - P11; P11 - P8; P2 - P3; P2 - P8; P3 - P8
TC0700010370.hg.1	TC0700010370.hg.1	NA	NA	NA	40.721	4.05E-07	6.3925	9.50E-07	ctrl - P11; P2 - ctrl; P3 - ctrl; ctrl - P8; P10 - P11; P2 - P10; P3 - P10; P10 - P8; P2 - P11; P3 - P11; P2 - P3; P2 - P8; P3 - P8
TC0200009344.hg.1	TC0200009344.hg.1	NM_001258304	CCDC74A	90557	40.632	4.10E-07	6.3871	9.61E-07	ctrl - P10; ctrl - P2; ctrl - P3; ctrl - P8; P11 - P10; P10 - P3; P11 - P2; P11 - P3; P11 - P8; P8 - P2; P8 - P3
TC0100009670.hg.1	TC0100009670.hg.1	NA	NA	NA	40.598	4.12E-07	6.3851	9.65E-07	ctrl - P10; ctrl - P11; ctrl - P3; ctrl - P8; P10 - P11; P2 - P10; P3 - P10; P10 - P8; P2 - P11; P3 - P11; P2 - P3; P2 - P8; P3 - P8
TC2100007806.hg.1	TC2100007806.hg.1	smuvee.aAug10-unspliced	smuvee	NA	40.501	4.18E-07	6.3793	9.77E-07	ctrl - P10; ctrl - P11; ctrl - P3; ctrl - P8; P2 - P10; P3 - P10; P2 - P11; P3 - P11; P2 - P3; P2 - P8; P3 - P8
TC0900010570.hg.1	TC0900010570.hg.1	NM_001244959	FRMD3	257019	40.448	4.21E-07	6.3761	9.83E-07	ctrl - P10; ctrl - P11; ctrl - P2; ctrl - P8; P11 - P10; P2 - P10; P3 - P10; P8 - P10; P3 - P11; P11 - P8; P3 - P2; P2 - P8; P3 - P8
TC0600007282.hg.1	TC0600007282.hg.1	NM_003522	HIST1H2BF	8343	40.441	4.21E-07	6.3757	9.83E-07	P10 - ctrl; ctrl - P2; P8 - ctrl; P10 - P11; P10 - P2; P10 - P3; P11 - P2; P8 - P11; P3 - P2; P8 - P2; P8 - P3
TC1100012687.hg.1	TC1100012687.hg.1	NM_001308027	MSANTD2	11q24.2	40.377	4.25E-07	6.3719	9.91E-07	ctrl - P10; ctrl - P11; ctrl - P3; ctrl - P8; P11 - P10; P2 - P10; P2 - P11; P11 - P3; P11 - P8; P2 - P3; P2 - P8
TC1300009783.hg.1	TC1300009783.hg.1	mimeri.aAug10-unspliced	mimeri	NA	40.351	4.26E-07	6.3703	9.94E-07	ctrl - P10; ctrl - P11; ctrl - P3; ctrl - P8; P2 - P10; P3 - P10; P2 - P11; P11 - P8; P2 - P3; P2 - P8; P3 - P8
TC1200011992.hg.1	TC1200011992.hg.1	vubler.aAug10-unspliced	vubler	NA	40.233	4.33E-07	6.3632	1.01E-06	P10 - ctrl; P11 - ctrl; P3 - ctrl; P8 - ctrl; P10 - P11; P10 - P2; P11 - P2; P8 - P11; P3 - P2; P8 - P2
TC1000011952.hg.1	TC1000011952.hg.1	porpoyby.aAug10-unspliced	porpoyby	NA	40.14	4.39E-07	6.3576	1.02E-06	ctrl - P10; ctrl - P11; ctrl - P3; ctrl - P8; P11 - P10; P2 - P10; P3 - P10; P2 - P11; P11 - P3; P11 - P8; P2 - P3; P2 - P8; P3 - P8
TC2200006540.hg.1	TC2200006540.hg.1	NM_017414	USP18	11274	40.081	4.43E-07	6.354	1.03E-06	P10 - ctrl; P3 - ctrl; P8 - ctrl; P10 - P11; P10 - P2; P3 - P11; P8 - P11; P3 - P2; P8 - P2
TC1900011382.hg.1	TC1900011382.hg.1	hsa_circ_0000954	MYADM	NA	40.042	4.45E-07	6.3516	1.03E-06	P10 - ctrl; P11 - ctrl; P3 - ctrl; P8 - ctrl; P10 - P11; P10 - P2; P10 - P3; P11 - P2; P8 - P11; P3 - P2; P8 - P2; P8 - P3
HTA2-pos-PSR07002495.hg.1	23151383	NA	NA	NA	39.988	4.48E-07	6.3484	1.04E-06	ctrl - P10; ctrl - P11; ctrl - P3; ctrl - P8; P11 - P10; P2 - P10; P3 - P10; P2 - P11; P11 - P8; P2 - P3; P2 - P8; P3 - P8
TC0300011888.hg.1	TC0300011888.hg.1	NA	NA	NA	39.878	4.55E-07	6.3417	1.06E-06	P10 - ctrl; P8 - ctrl; P10 - P11; P10 - P2; P10 - P3; P10 - P8; P8 - P11; P8 - P2; P8 - P3
TC0200009895.hg.1	TC0200009895.hg.1	NM_001256126	CERS6	253782	39.701	4.67E-07	6.3309	1.08E-06	ctrl - P10; ctrl - P2; ctrl - P8; P11 - P10; P2 - P10; P3 - P10; P8 - P10; P11 - P2; P11 - P8; P3 - P2; P3 - P8
TC0200012989.hg.1	TC0200012989.hg.1	wuklar.aAug10-unspliced	wuklar	NA	39.657	4.70E-07	6.3282	1.09E-06	ctrl - P10; ctrl - P11; ctrl - P3; ctrl - P8; P11 - P10; P2 - P10; P3 - P10; P2 - P11; P11 - P8; P2 - P3; P2 - P8; P3 - P8
TC0900011662.hg.1	TC0900011662.hg.1	kortuby.aAug10-unspliced	kortuby	NA	39.579	4.75E-07	6.3234	1.10E-06	P10 - ctrl; P11 - ctrl; P3 - ctrl; P8 - ctrl; P10 - P11; P10 - P2; P10 - P3; P11 - P2; P3 - P11; P8 - P11; P3 - P2; P8 - P2; P8 - P3
TC1500007593.hg.1	TC1500007593.hg.1	NR_002756	RNU5A-1	26831	39.556	4.76E-07	6.322	1.10E-06	P11 - ctrl; P2 - ctrl; P11 - P10; P2 - P10; P2 - P11; P11 - P3; P11 - P8; P2 - P3; P2 - P8
TC0100016693.hg.1	TC0100016693.hg.1	ENST00000623474	RP4-635A23.6		39.486	4.81E-07	6.3177	1.11E-06	ctrl - P10; ctrl - P11; ctrl - P3; ctrl - P8; P2 - P10; P2 - P11; P2 - P3; P2 - P8
TC1500006585.hg.1	TC1500006585.hg.1	NR_003300	SNORD115-8	15q11.2	39.452	4.83E-07	6.3156	1.11E-06	P10 - ctrl; P11 - ctrl; P3 - ctrl; P8 - ctrl; P10 - P11; P10 - P2; P3 - P10; P3 - P11; P8 - P11; P3 - P2; P8 - P2; P3 - P8
TC1100010456.hg.1	TC1100010456.hg.1	NM_000280	PAX6	5080	39.386	4.88E-07	6.3116	1.12E-06	P11 - ctrl; ctrl - P2; P3 - ctrl; P11 - P10; P10 - P2; P3 - P10; P11 - P2; P11 - P8; P3 - P2; P3 - P8
TC0200012343.hg.1	TC0200012343.hg.1	steelo.aAug10-unspliced	steelo	NA	39.302	4.94E-07	6.3064	1.14E-06	P10 - ctrl; P2 - ctrl; P10 - P11; P2 - P10; P10 - P3; P10 - P8; P2 - P11; P2 - P3; P2 - P8
TC0100018300.hg.1	TC0100018300.hg.1	NM_001261464	ADAM15	8751	39.248	4.98E-07	6.3031	1.14E-06	P10 - ctrl; ctrl - P2; P3 - ctrl; P8 - ctrl; P10 - P11; P10 - P2; P11 - P2; P3 - P11; P8 - P11; P3 - P2; P8 - P2; P8 - P3

TC0400010128.hg.1	TC0400010128.hg.1	smusmawby.aAug10-unspliced	smusmawby	NA	39.231	4.99E-07	6.302	1.15E-06	ctrl - P10; ctrl - P11; ctrl - P3; ctrl - P8; P11 - P10; P2 - P10; P3 - P10; P2 - P11; P11 - P8; P2 - P3; P2 - P8; P3 - P8
TC0100009149.hg.1	TC0100009149.hg.1	PTBP2.oAug10-unspliced	PTBP2	NA	39.177	5.03E-07	6.2986	1.15E-06	ctrl - P10; ctrl - P11; P2 - ctrl; ctrl - P3; ctrl - P8; P2 - P10; P3 - P10; P2 - P11; P11 - P8; P2 - P3; P2 - P8; P3 - P8
TC1100008008.hg.1	TC1100008008.hg.1	NM_002689	POLA2	23649	39.172	5.03E-07	6.2983	1.15E-06	ctrl - P11; ctrl - P2; P3 - ctrl; P8 - ctrl; P10 - P11; P10 - P2; P3 - P10; P8 - P10; P3 - P11; P8 - P11; P3 - P2; P8 - P2
TC1100010131.hg.1	TC1100010131.hg.1	ENST00000527288.1	RP11-265D17.2		39.108	5.08E-07	6.2944	1.16E-06	P10 - ctrl; P8 - ctrl; P10 - P11; P10 - P2; P10 - P3; P8 - P11; P8 - P2; P8 - P3
TC0X00009917.hg.1	TC0X00009917.hg.1	NM_001199687	EDA2R	60401	39.019	5.14E-07	6.2889	1.18E-06	P10 - ctrl; P11 - ctrl; P3 - ctrl; P8 - ctrl; P10 - P11; P10 - P2; P10 - P3; P11 - P2; P8 - P11; P3 - P2; P8 - P2; P8 - P3
TC0800011305.hg.1	TC0800011305.hg.1	NM_001040624	NCALD	83988	38.982	5.17E-07	6.2866	1.18E-06	P10 - ctrl; P3 - ctrl; P8 - ctrl; P10 - P11; P10 - P2; P10 - P3; P3 - P11; P8 - P11; P3 - P2; P8 - P2; P8 - P3
TC0900006657.hg.1	TC0900006657.hg.1	sposhaw.aAug10-unspliced	sposhaw	NA	38.814	5.30E-07	6.2761	1.21E-06	ctrl - P10; ctrl - P11; P2 - ctrl; ctrl - P8; P2 - P10; P3 - P10; P10 - P8; P2 - P11; P3 - P11; P11 - P8; P2 - P3; P2 - P8; P3 - P8
TC1400008189.hg.1	TC1400008189.hg.1	tamami.aAug10-unspliced	tamami	NA	38.732	5.36E-07	6.271	1.22E-06	ctrl - P10; ctrl - P11; ctrl - P3; ctrl - P8; P2 - P10; P3 - P10; P10 - P8; P2 - P11; P11 - P8; P2 - P3; P2 - P8; P3 - P8
TC0600014191.hg.1	TC0600014191.hg.1	NM_001030060	SAMD5	389432	38.725	5.36E-07	6.2706	1.22E-06	P10 - ctrl; P2 - ctrl; P3 - ctrl; P8 - ctrl; P10 - P11; P10 - P2; P10 - P3; P2 - P11; P3 - P11; P8 - P11; P2 - P3; P8 - P3
TC0700007694.hg.1	TC0700007694.hg.1	NA	NA	NA	38.641	5.43E-07	6.2653	1.24E-06	P2 - ctrl; P3 - ctrl; ctrl - P8; P10 - P11; P2 - P10; P3 - P10; P10 - P8; P2 - P11; P3 - P11; P2 - P8; P3 - P8
TC1200012298.hg.1	TC1200012298.hg.1	ENST00000602741.1	RP11-214K3.19		38.63	5.44E-07	6.2646	1.24E-06	ctrl - P10; ctrl - P11; P2 - ctrl; ctrl - P3; ctrl - P8; P2 - P10; P3 - P10; P2 - P11; P11 - P8; P2 - P3; P2 - P8; P3 - P8
TC1700010440.hg.1	TC1700010440.hg.1	NM_001032278	MMP28	79148	38.501	5.54E-07	6.2565	1.26E-06	P10 - ctrl; P11 - ctrl; P3 - ctrl; P8 - ctrl; P10 - P2; P3 - P10; P11 - P2; P3 - P11; P8 - P11; P3 - P2; P8 - P2; P3 - P8
TC0500011579.hg.1	TC0500011579.hg.1	NM_005668	ST8SIA4	7903	38.487	5.55E-07	6.2556	1.26E-06	P11 - ctrl; P2 - ctrl; P11 - P10; P2 - P10; P2 - P11; P11 - P3; P11 - P8; P2 - P3; P2 - P8
TC0200014733.hg.1	TC0200014733.hg.1	NA	NA	NA	38.437	5.59E-07	6.2525	1.27E-06	ctrl - P10; ctrl - P11; P2 - ctrl; ctrl - P3; ctrl - P8; P2 - P10; P2 - P11; P2 - P3; P2 - P8; P3 - P8
TC0300013555.hg.1	TC0300013555.hg.1	NM_178496	MB21D2	151963	38.283	5.72E-07	6.2428	1.30E-06	P10 - ctrl; ctrl - P2; ctrl - P3; P8 - ctrl; P10 - P11; P10 - P2; P10 - P3; P11 - P2; P11 - P3; P8 - P11; P3 - P2; P8 - P2; P8 - P3
TC1700007167.hg.1	TC1700007167.hg.1	snovybu.aAug10-unspliced	snovybu	NA	38.241	5.75E-07	6.2402	1.30E-06	ctrl - P10; ctrl - P11; P2 - ctrl; ctrl - P3; ctrl - P8; P11 - P10; P2 - P10; P3 - P10; P2 - P11; P11 - P8; P2 - P3; P2 - P8; P3 - P8
TC0900012180.hg.1	TC0900012180.hg.1	NM_001100876	PHYHD1	254295	38.235	5.76E-07	6.2398	1.30E-06	ctrl - P10; P11 - ctrl; ctrl - P2; ctrl - P8; P11 - P10; P3 - P10; P11 - P2; P11 - P3; P11 - P8; P3 - P2; P3 - P8
TC0200011507.hg.1	TC0200011507.hg.1	NA	NA	NA	38.196	5.79E-07	6.2372	1.31E-06	ctrl - P10; ctrl - P11; ctrl - P2; ctrl - P8; P2 - P10; P3 - P10; P2 - P11; P3 - P11; P3 - P2; P2 - P8; P3 - P8
TC1000010851.hg.1	TC1000010851.hg.1	chawmyby.aAug10-unspliced	chawmyby	NA	38.186	5.80E-07	6.2367	1.31E-06	ctrl - P10; ctrl - P11; ctrl - P3; ctrl - P8; P2 - P10; P10 - P8; P2 - P11; P3 - P11; P2 - P3; P2 - P8; P3 - P8
TC0X00008468.hg.1	TC0X00008468.hg.1	NM_001101357	CCDC160	347475	38.139	5.84E-07	6.2337	1.32E-06	ctrl - P10; ctrl - P11; ctrl - P2; ctrl - P3; ctrl - P8; P11 - P10; P2 - P10; P3 - P10; P11 - P3; P11 - P8; P2 - P3; P2 - P8; P3 - P8
HTA2-pos-47421982_st	23150845	NA	NA	NA	38.129	5.85E-07	6.233	1.32E-06	ctrl - P10; ctrl - P11; ctrl - P3; ctrl - P8; P11 - P10; P2 - P10; P3 - P10; P2 - P11; P2 - P3; P2 - P8
TC1400006436.hg.1	TC1400006436.hg.1	NA	NA	NA	38.127	5.85E-07	6.2329	1.32E-06	ctrl - P11; P2 - ctrl; P3 - ctrl; P10 - P11; P2 - P10; P3 - P10; P10 - P8; P2 - P11; P3 - P11; P2 - P3; P2 - P8; P3 - P8
TC0800007672.hg.1	TC0800007672.hg.1	NM_052898	XKR4	114786	38.115	5.86E-07	6.2322	1.32E-06	ctrl - P11; P2 - ctrl; P3 - ctrl; ctrl - P8; P10 - P11; P2 - P10; P10 - P8; P2 - P11; P3 - P11; P2 - P8; P3 - P8
TC0900008053.hg.1	TC0900008053.hg.1	gawlar.aAug10-unspliced	gawlar	NA	38.11	5.86E-07	6.2318	1.32E-06	ctrl - P10; ctrl - P11; P2 - ctrl; ctrl - P3; ctrl - P8; P2 - P10; P10 - P8; P2 - P11; P11 - P8; P2 - P3; P2 - P8; P3 - P8
TC0200007908.hg.1	TC0200007908.hg.1	NM_018153	ANTXR1	84168	38.026	5.94E-07	6.2265	1.33E-06	ctrl - P10; ctrl - P2; ctrl - P3; ctrl - P8; P11 - P10; P10 - P8; P11 - P2; P11 - P3; P11 - P8; P2 - P8
TC1700010652.hg.1	TC1700010652.hg.1	NM_033032	KRTAP2-2	728279	38.007	5.95E-07	6.2253	1.34E-06	P10 - ctrl; P3 - ctrl; P8 - ctrl; P10 - P11; P10 - P2; P10 - P3; P8 - P10; P3 - P11; P8 - P11; P3 - P2; P8 - P2; P8 - P3
TC0700008354.hg.1	TC0700008354.hg.1	ENST00000415536.1	AC003092.1		37.957	6.00E-07	6.2221	1.35E-06	P10 - ctrl; P3 - ctrl; P8 - ctrl; P10 - P11; P10 - P2; P3 - P10; P8 - P10; P3 - P11; P8 - P11; P3 - P2; P8 - P2
TC0400006931.hg.1	TC0400006931.hg.1	gloygu.aAug10-unspliced	gloygu	NA	37.903	6.04E-07	6.2187	1.35E-06	ctrl - P10; ctrl - P11; ctrl - P3; ctrl - P8; P11 - P10; P2 - P10; P3 - P10; P2 - P11; P11 - P3; P11 - P8; P2 - P3; P2 - P8; P3 - P8
TC0X00006804.hg.1	TC0X00006804.hg.1	NA	NA	NA	37.892	6.05E-07	6.2179	1.36E-06	ctrl - P10; ctrl - P11; ctrl - P3; ctrl - P8; P2 - P10; P3 - P10; P2 - P11; P3 - P11; P2 - P3; P2 - P8; P3 - P8
TC1000009674.hg.1	TC1000009674.hg.1	ENST00000478294	LOC105376382	1.05E+08	37.887	6.06E-07	6.2176	1.36E-06	P3 - ctrl; P8 - ctrl; P10 - P2; P3 - P10; P8 - P10; P3 - P11; P8 - P11; P3 - P2; P8 - P2
TC1000008395.hg.1	TC1000008395.hg.1	ENST00000354541.3	RP11-149I23.3		37.863	6.08E-07	6.2161	1.36E-06	ctrl - P10; ctrl - P2; ctrl - P3; ctrl - P8; P11 - P10; P2 - P10; P11 - P2; P11 - P3; P11 - P8; P2 - P3; P2 - P8
TC0800007051.hg.1	TC0800007051.hg.1	NM_001105541	NEFM	4741	37.835	6.10E-07	6.2143	1.36E-06	ctrl - P10; ctrl - P11; ctrl - P2; ctrl - P8; P11 - P10; P2 - P10; P3 - P10; P11 - P2; P3 - P11; P11 - P8; P3 - P2; P2 - P8; P3 - P8
TC0300006705.hg.1	TC0300006705.hg.1	NR_046084	SH3BP5-AS1	1.01E+08	37.803	6.13E-07	6.2122	1.37E-06	ctrl - P10; ctrl - P11; ctrl - P3; ctrl - P8; P11 - P10; P2 - P10; P3 - P10; P2 - P11; P11 - P8; P2 - P3; P2 - P8; P3 - P8

TC1600009927.hg.1	TC1600009927.hg.1	NA	NA	NA	37.767	6.17E-07	6.21	1.37E-06	ctrl - P10; ctrl - P11; ctrl - P3; ctrl - P8; P2 - P10; P2 - P11; P11 - P8; P2 - P3; P2 - P8; P3 - P8
TC1600009960.hg.1	TC1600009960.hg.1	NA	NA	NA	37.767	6.17E-07	6.21	1.37E-06	ctrl - P10; ctrl - P11; ctrl - P3; ctrl - P8; P2 - P10; P2 - P11; P11 - P8; P2 - P3; P2 - P8; P3 - P8
TC0500011013.hg.1	TC0500011013.hg.1	vostarby.aAug10-unspliced	vostarby	NA	37.739	6.19E-07	6.2081	1.38E-06	ctrl - P10; ctrl - P11; ctrl - P3; ctrl - P8; P11 - P10; P2 - P10; P3 - P10; P2 - P11; P11 - P8; P2 - P3; P2 - P8; P3 - P8
TC0200012826.hg.1	TC0200012826.hg.1	ENST00000561559.1	RP11-568N6.1		37.708	6.22E-07	6.2062	1.38E-06	ctrl - P10; ctrl - P11; ctrl - P2; ctrl - P3; ctrl - P8; P11 - P10; P2 - P10; P10 - P3; P11 - P3; P11 - P8; P2 - P3; P2 - P8; P8 - P3
TC0100014027.hg.1	TC0100014027.hg.1	rerparbu.aAug10-unspliced	rerparbu	NA	37.691	6.24E-07	6.2051	1.39E-06	ctrl - P10; ctrl - P11; P2 - ctrl; ctrl - P3; ctrl - P8; P11 - P10; P2 - P10; P3 - P10; P2 - P11; P11 - P8; P2 - P3; P2 - P8; P3 - P8
TC1700011421.hg.1	TC1700011421.hg.1	rawwey.aAug10-unspliced	rawwey	NA	37.593	6.33E-07	6.1988	1.40E-06	ctrl - P10; ctrl - P11; P2 - ctrl; ctrl - P3; ctrl - P8; P2 - P10; P3 - P10; P2 - P11; P3 - P11; P2 - P3; P2 - P8; P3 - P8
TC0800007641.hg.1	TC0800007641.hg.1	NM_001286673	RGS20	8601	37.505	6.41E-07	6.1932	1.42E-06	P10 - ctrl; P3 - ctrl; P8 - ctrl; P10 - P11; P10 - P2; P3 - P11; P8 - P11; P3 - P2; P8 - P2; P8 - P3
TC2200008036.hg.1	TC2200008036.hg.1	OTTHUMT00000075837	USP41	373856	37.433	6.48E-07	6.1885	1.44E-06	P10 - ctrl; P3 - ctrl; P8 - ctrl; P10 - P11; P10 - P2; P3 - P11; P8 - P11; P3 - P2; P8 - P2
TC0600007585.hg.1	TC0600007585.hg.1	NM_001289161	MICB	4277	37.42	6.49E-07	6.1877	1.44E-06	P10 - ctrl; ctrl - P2; P3 - ctrl; P8 - ctrl; P10 - P11; P10 - P2; P10 - P3; P8 - P10; P11 - P2; P3 - P11; P8 - P11; P3 - P2; P8 - P2; P8 - P3
TC0900011875.hg.1	TC0900011875.hg.1	NR_023344	RNU6ATAC	1E+08	37.413	6.50E-07	6.1872	1.44E-06	ctrl - P10; P2 - ctrl; ctrl - P8; P11 - P10; P2 - P10; P3 - P10; P2 - P11; P11 - P8; P2 - P3; P2 - P8; P3 - P8
TC0100014952.hg.1	TC0100014952.hg.1	NR_034089	CCDC18-AS1	1E+08	37.405	6.51E-07	6.1867	1.44E-06	ctrl - P10; ctrl - P11; ctrl - P3; ctrl - P8; P11 - P10; P2 - P10; P3 - P10; P11 - P8; P2 - P3; P2 - P8; P3 - P8
TC1200009297.hg.1	TC1200009297.hg.1	feeforby.aAug10-unspliced	feeforby		37.353	6.56E-07	6.1833	1.45E-06	P10 - ctrl; P11 - ctrl; P3 - ctrl; P8 - ctrl; P10 - P11; P10 - P2; P10 - P3; P11 - P2; P8 - P11; P3 - P2; P8 - P2; P8 - P3
TC0500009377.hg.1	TC0500009377.hg.1	NM_017785	SPDL1	54908	37.257	6.65E-07	6.1771	1.47E-06	P10 - ctrl; P3 - ctrl; P8 - ctrl; P10 - P11; P10 - P2; P8 - P10; P3 - P11; P8 - P11; P3 - P2; P8 - P2; P8 - P3
TC0X00007243.hg.1	TC0X00007243.hg.1	uc022bwf.2	AC231645.1	NA	37.206	6.70E-07	6.1738	1.48E-06	ctrl - P10; ctrl - P11; ctrl - P2; ctrl - P3; ctrl - P8; P11 - P10; P2 - P10; P11 - P3; P11 - P8; P2 - P3; P2 - P8
TC0700010537.hg.1	TC0700010537.hg.1	NA	NA	NA	37.179	6.73E-07	6.1721	1.48E-06	ctrl - P10; ctrl - P11; ctrl - P3; ctrl - P8; P11 - P10; P2 - P10; P3 - P10; P2 - P11; P11 - P8; P2 - P3; P2 - P8; P3 - P8
TC0400006665.hg.1	TC0400006665.hg.1	zawku.aAug10-unspliced	zawku	NA	37.118	6.79E-07	6.1681	1.49E-06	ctrl - P10; P11 - ctrl; ctrl - P3; ctrl - P8; P11 - P10; P2 - P10; P3 - P10; P11 - P2; P11 - P3; P11 - P8; P2 - P3; P2 - P8; P3 - P8
TC1700006647.hg.1	TC1700006647.hg.1	lywaw.aAug10-unspliced	lywaw	NA	37.089	6.82E-07	6.1662	1.50E-06	ctrl - P10; ctrl - P11; ctrl - P3; ctrl - P8; P2 - P10; P3 - P10; P2 - P11; P11 - P8; P2 - P3; P2 - P8; P3 - P8
TC1500008525.hg.1	TC1500008525.hg.1	poychubu.aAug10-unspliced	poychubu	NA	37.07	6.84E-07	6.165	1.50E-06	ctrl - P10; ctrl - P11; P2 - ctrl; ctrl - P3; ctrl - P8; P11 - P10; P2 - P10; P3 - P10; P2 - P11; P11 - P8; P2 - P3; P2 - P8; P3 - P8
TC0500011852.hg.1	TC0500011852.hg.1	NM_001178102	LOX	4015	37.049	6.86E-07	6.1636	1.51E-06	P10 - ctrl; P3 - ctrl; P8 - ctrl; P10 - P11; P10 - P2; P3 - P11; P8 - P11; P3 - P2; P8 - P2
TC0200008981.hg.1	TC0200008981.hg.1	doswawby.aAug10-unspliced	doswawby	NA	36.977	6.94E-07	6.1589	1.52E-06	ctrl - P10; ctrl - P11; ctrl - P2; ctrl - P3; ctrl - P8; P11 - P10; P2 - P10; P3 - P10; P2 - P11; P11 - P8; P2 - P3; P2 - P8; P3 - P8
TC1100008401.hg.1	TC1100008401.hg.1	zeekarby.aAug10	zeekarby	NA	36.932	6.98E-07	6.156	1.53E-06	P10 - ctrl; P3 - ctrl; P8 - ctrl; P10 - P11; P10 - P2; P10 - P3; P3 - P11; P8 - P11; P3 - P2; P8 - P2; P8 - P3
TC0100017849.hg.1	TC0100017849.hg.1	NM_019891	ERO1B	56605	36.928	6.99E-07	6.1557	1.53E-06	ctrl - P10; ctrl - P2; ctrl - P3; ctrl - P8; P11 - P10; P3 - P10; P11 - P2; P11 - P3; P11 - P8; P3 - P2; P3 - P8
TC1000010102.hg.1	TC1000010102.hg.1	joymorby.aAug10-unspliced	joymorby	NA	36.917	7.00E-07	6.155	1.53E-06	ctrl - P10; ctrl - P11; ctrl - P3; ctrl - P8; P11 - P10; P2 - P10; P10 - P8; P2 - P11; P11 - P8; P2 - P3; P2 - P8; P3 - P8
TC0100015417.hg.1	TC0100015417.hg.1	NM_001253849	VTCN1	79679	36.914	7.00E-07	6.1548	1.53E-06	P10 - ctrl; P11 - ctrl; ctrl - P2; P3 - ctrl; P8 - ctrl; P10 - P11; P10 - P2; P10 - P3; P11 - P2; P3 - P2; P8 - P2; P8 - P3
TC0600011973.hg.1	TC0600011973.hg.1	gleebloy.aAug10-unspliced	gleebloy	NA	36.888	7.03E-07	6.1531	1.53E-06	P10 - ctrl; ctrl - P11; P2 - ctrl; P3 - ctrl; P8 - ctrl; P10 - P11; P2 - P10; P3 - P10; P2 - P11; P3 - P11; P8 - P11; P2 - P8; P3 - P8
TC1200010130.hg.1	TC1200010130.hg.1	NM_001261414	SOX5	6660	36.876	7.04E-07	6.1523	1.54E-06	ctrl - P10; ctrl - P3; ctrl - P8; P11 - P10; P2 - P10; P10 - P3; P11 - P3; P11 - P8; P2 - P3; P2 - P8
TC0X00008826.hg.1	TC0X00008826.hg.1	hsa_circ_0002730	FLNA	NA	36.766	7.16E-07	6.1451	1.56E-06	P10 - ctrl; P11 - ctrl; P3 - ctrl; P8 - ctrl; P10 - P11; P10 - P2; P10 - P3; P11 - P2; P8 - P11; P3 - P2; P8 - P2; P8 - P3
TC0400010503.hg.1	TC0400010503.hg.1	NR_039966	MIR4802	1.01E+08	36.753	7.17E-07	6.1443	1.56E-06	ctrl - P10; P2 - ctrl; ctrl - P3; ctrl - P8; P11 - P10; P2 - P10; P10 - P3; P2 - P11; P11 - P3; P11 - P8; P2 - P3; P2 - P8
TC0100007907.hg.1	TC0100007907.hg.1	MACF1andKIAA0754.vpAug10-unspliced	MACF1andKIAA0754	NA	36.7	7.23E-07	6.1408	1.57E-06	ctrl - P10; ctrl - P11; ctrl - P3; ctrl - P8; P2 - P10; P3 - P10; P2 - P11; P3 - P11; P2 - P3; P2 - P8; P3 - P8
TC0X00008526.hg.1	TC0X00008526.hg.1	NM_001159699	FHL1	2273	36.7	7.23E-07	6.1407	1.57E-06	P10 - ctrl; P3 - ctrl; P8 - ctrl; P10 - P11; P10 - P2; P8 - P10; P3 - P11; P8 - P11; P3 - P2; P8 - P2; P8 - P3
TC1000012438.hg.1	TC1000012438.hg.1	NR_036438	SVILP1	645954	36.68	7.25E-07	6.1394	1.57E-06	P10 - ctrl; P3 - ctrl; P8 - ctrl; P10 - P11; P10 - P2; P3 - P10; P8 - P10; P3 - P11; P8 - P11; P3 - P2; P8 - P2
TC0200012980.hg.1	TC0200012980.hg.1	NM_152792	ASPRV1	151516	36.658	7.28E-07	6.138	1.58E-06	ctrl - P10; P11 - ctrl; ctrl - P3; ctrl - P8; P11 - P10; P2 - P10; P11 - P2; P11 - P3; P11 - P8; P2 - P3; P2 - P8; P3 - P8
TC1600010641.hg.1	TC1600010641.hg.1	ENST00000563083.1	CTD-2012K14.3		36.655	7.28E-07	6.1378	1.58E-06	P10 - ctrl; P11 - ctrl; P3 - ctrl; P8 - ctrl; P10 - P11; P10 - P2; P10 - P3; P10 - P8; P11 - P2; P8 - P11; P3 - P2; P8 - P2; P8 - P3

TC1300007110.hg.1	TC1300007110.hg.1	OTTHUMT00000474841	RP11-165D6.1		36.625	7.31E-07	6.1358	1.58E-06	ctrl - P10; ctrl - P11; ctrl - P8; P11 - P10; P2 - P10; P3 - P10; P2 - P11; P3 - P11; P11 - P8; P2 - P8; P3 - P8
TC0800012464.hg.1	TC0800012464.hg.1	NR_024393	CASC8	727677	36.592	7.35E-07	6.1336	1.59E-06	P10 - ctrl; P3 - ctrl; P8 - ctrl; P10 - P11; P10 - P2; P3 - P10; P3 - P11; P8 - P11; P3 - P2; P8 - P2
TC0200013021.hg.1	TC0200013021.hg.1	NM_020459	PAIP2B	400961	36.58	7.36E-07	6.1328	1.59E-06	ctrl - P10; ctrl - P2; ctrl - P3; ctrl - P8; P11 - P10; P2 - P10; P11 - P2; P11 - P3; P11 - P8; P2 - P3; P2 - P8
TC0100009476.hg.1	TC0100009476.hg.1	hunura.aAug10-unspliced	hunura	NA	36.516	7.44E-07	6.1286	1.61E-06	ctrl - P10; P2 - ctrl; ctrl - P3; ctrl - P8; P11 - P10; P2 - P10; P3 - P10; P2 - P11; P11 - P3; P11 - P8; P2 - P3; P2 - P8; P3 - P8
TC0100014531.hg.1	TC0100014531.hg.1	NM_001002292	WLS	79971	36.475	7.48E-07	6.1259	1.61E-06	ctrl - P10; ctrl - P2; ctrl - P3; ctrl - P8; P11 - P10; P2 - P10; P3 - P10; P8 - P10; P11 - P2; P11 - P3; P11 - P8
TC0600008509.hg.1	TC0600008509.hg.1	NM_001160130	KCNQ5	56479	36.473	7.48E-07	6.1258	1.61E-06	ctrl - P10; ctrl - P11; ctrl - P2; ctrl - P3; ctrl - P8; P11 - P10; P2 - P10; P3 - P10; P2 - P11; P3 - P11; P2 - P3; P2 - P8; P3 - P8
TC1500008797.hg.1	TC1500008797.hg.1	yoharu.aAug10-unspliced	yoharu	NA	36.425	7.54E-07	6.1227	1.62E-06	ctrl - P10; ctrl - P11; ctrl - P3; ctrl - P8; P2 - P10; P3 - P10; P10 - P8; P2 - P11; P3 - P11; P2 - P3; P2 - P8; P3 - P8
TC1900007992.hg.1	TC1900007992.hg.1	reture.aAug10-unspliced	reture	NA	36.4	7.57E-07	6.1209	1.63E-06	ctrl - P10; ctrl - P11; ctrl - P8; P10 - P11; P2 - P10; P3 - P10; P2 - P11; P3 - P11; P8 - P11; P2 - P8; P3 - P8
TC0100017094.hg.1	TC0100017094.hg.1	NM_001123168	FAM72A	729533	36.359	7.62E-07	6.1182	1.64E-06	P10 - ctrl; P8 - ctrl; P10 - P11; P10 - P2; P10 - P3; P8 - P10; P3 - P11; P8 - P11; P3 - P2; P8 - P2; P8 - P3
TC1200007154.hg.1	TC1200007154.hg.1	smyguby.aAug10-unspliced	smyguby	NA	36.294	7.69E-07	6.1139	1.65E-06	ctrl - P10; ctrl - P11; ctrl - P3; ctrl - P8; P2 - P10; P10 - P8; P2 - P11; P11 - P8; P2 - P3; P2 - P8; P3 - P8
TSUnmapped00000137.hg.1	TSUnmapped00000137.hg.1	ENST00000626615	ABO	NA	36.271	7.72E-07	6.1124	1.66E-06	ctrl - P10; ctrl - P2; ctrl - P8; P11 - P10; P3 - P10; P11 - P2; P11 - P8; P3 - P2; P3 - P8
TC1500010725.hg.1	TC1500010725.hg.1	NM_000070	CAPN3	825	36.238	7.76E-07	6.1103	1.66E-06	ctrl - P10; ctrl - P11; ctrl - P2; ctrl - P3; ctrl - P8; P11 - P10; P2 - P10; P3 - P10; P11 - P3; P11 - P8; P2 - P3; P2 - P8; P3 - P8
TC2100008297.hg.1	TC2100008297.hg.1	ENST00000270162	SIK1	150094	36.195	7.81E-07	6.1074	1.67E-06	P10 - ctrl; P11 - ctrl; ctrl - P2; P3 - ctrl; P8 - ctrl; P10 - P11; P10 - P2; P10 - P3; P11 - P2; P8 - P11; P3 - P2; P8 - P2; P8 - P3
TC0900008954.hg.1	TC0900008954.hg.1	soshaw.aAug10-unspliced	soshaw	NA	36.143	7.87E-07	6.1039	1.69E-06	ctrl - P10; ctrl - P11; ctrl - P3; ctrl - P8; P2 - P10; P10 - P8; P2 - P11; P11 - P3; P11 - P8; P2 - P3; P2 - P8
TC1500008894.hg.1	TC1500008894.hg.1	uc059hck.1	RNU6-466P	NA	36.127	7.89E-07	6.1028	1.69E-06	ctrl - P10; ctrl - P11; P2 - ctrl; ctrl - P3; ctrl - P8; P2 - P10; P3 - P10; P2 - P11; P11 - P8; P2 - P3; P2 - P8; P3 - P8
TC0500009212.hg.1	TC0500009212.hg.1	klaflo.aAug10-unspliced	klaflo	NA	36.101	7.92E-07	6.1011	1.69E-06	ctrl - P10; ctrl - P3; ctrl - P8; P11 - P10; P2 - P10; P11 - P2; P11 - P3; P11 - P8; P2 - P3; P2 - P8; P3 - P8
TC0300009164.hg.1	TC0300009164.hg.1	klardorbu.aAug10-unspliced	klardorbu	NA	36.091	7.94E-07	6.1004	1.69E-06	ctrl - P10; ctrl - P11; ctrl - P3; ctrl - P8; P11 - P10; P2 - P10; P3 - P10; P2 - P11; P11 - P8; P2 - P3; P2 - P8; P3 - P8
TC0100009144.hg.1	TC0100009144.hg.1	skoroybo.aAug10-unspliced	skoroybo	NA	36.09	7.94E-07	6.1004	1.69E-06	ctrl - P10; ctrl - P11; P2 - ctrl; ctrl - P3; ctrl - P8; P2 - P10; P3 - P10; P10 - P8; P2 - P11; P3 - P11; P11 - P8; P2 - P3; P2 - P8; P3 - P8
TC0500009320.hg.1	TC0500009320.hg.1	NM_001142556	HMMR	3161	35.989	8.06E-07	6.0936	1.72E-06	P10 - ctrl; P3 - ctrl; P8 - ctrl; P10 - P11; P10 - P2; P8 - P10; P3 - P11; P8 - P11; P3 - P2; P8 - P2; P8 - P3
TC0100018248.hg.1	TC0100018248.hg.1	NM_017619	RNPC3	55599	35.954	8.10E-07	6.0913	1.73E-06	ctrl - P10; ctrl - P11; ctrl - P3; ctrl - P8; P11 - P10; P2 - P10; P2 - P11; P11 - P3; P11 - P8; P2 - P3; P2 - P8; P3 - P8
TC0100012764.hg.1	TC0100012764.hg.1	gomabo.aAug10-unspliced	gomabo	NA	35.916	8.15E-07	6.0887	1.74E-06	ctrl - P10; ctrl - P11; ctrl - P3; ctrl - P8; P11 - P10; P2 - P10; P10 - P8; P2 - P11; P11 - P3; P11 - P8; P2 - P3; P2 - P8; P3 - P8
TC0600011943.hg.1	TC0600011943.hg.1	NM_001290072	ENPP5	6p21.1-p11.2	35.895	8.18E-07	6.0873	1.74E-06	ctrl - P10; ctrl - P2; ctrl - P3; ctrl - P8; P11 - P10; P10 - P2; P10 - P3; P10 - P8; P11 - P2; P11 - P3; P11 - P8; P8 - P2
TC0600010131.hg.1	TC0600010131.hg.1	derfleeby.aAug10-unspliced	derfleeby	NA	35.728	8.39E-07	6.0761	1.78E-06	ctrl - P10; ctrl - P11; ctrl - P3; ctrl - P8; P11 - P10; P2 - P10; P2 - P11; P11 - P8; P2 - P3; P2 - P8
TC0200010446.hg.1	TC0200010446.hg.1	meygybu.aAug10-unspliced	meygybu	NA	35.626	8.53E-07	6.0692	1.81E-06	ctrl - P10; ctrl - P11; ctrl - P3; ctrl - P8; P11 - P10; P2 - P10; P10 - P8; P2 - P11; P11 - P3; P11 - P8; P2 - P3; P2 - P8; P3 - P8
TC1900006816.hg.1	TC1900006816.hg.1	NA	NA	NA	35.57	8.60E-07	6.0654	1.82E-06	P10 - ctrl; P8 - ctrl; P10 - P11; P10 - P2; P10 - P3; P8 - P11; P3 - P2; P8 - P2; P8 - P3
TC0100014966.hg.1	TC0100014966.hg.1	NM_001308253	GCLM	2730	35.55	8.63E-07	6.0641	1.83E-06	P10 - ctrl; P3 - ctrl; P8 - ctrl; P10 - P11; P10 - P2; P3 - P11; P8 - P11; P3 - P2; P8 - P2
TC1200012547.hg.1	TC1200012547.hg.1	ENST00000623177	RP11-46H11.11		35.517	8.67E-07	6.0619	1.84E-06	ctrl - P10; ctrl - P11; ctrl - P3; ctrl - P8; P2 - P10; P3 - P10; P2 - P11; P3 - P11; P2 - P3; P2 - P8; P3 - P8
TC0600011887.hg.1	TC0600011887.hg.1	ENST00000607600.1	RP1-261G23.7		35.458	8.75E-07	6.0578	1.85E-06	P10 - ctrl; P11 - ctrl; ctrl - P2; ctrl - P3; P8 - ctrl; P10 - P2; P10 - P3; P11 - P2; P11 - P3; P3 - P2; P8 - P2; P8 - P3
TC2000007242.hg.1	TC2000007242.hg.1	OTTHUMT00000473783	RP11-234K24.6		35.451	8.76E-07	6.0573	1.85E-06	ctrl - P10; ctrl - P11; ctrl - P2; ctrl - P3; ctrl - P8; P11 - P10; P2 - P10; P11 - P3; P11 - P8; P2 - P3; P2 - P8
TC0X00009073.hg.1	TC0X00009073.hg.1	NA	NA	NA	35.432	8.79E-07	6.0561	1.86E-06	ctrl - P10; ctrl - P11; ctrl - P3; ctrl - P8; P11 - P10; P2 - P10; P10 - P8; P2 - P11; P11 - P8; P2 - P3; P2 - P8; P3 - P8
TC1200011496.hg.1	TC1200011496.hg.1	NM_001920	DCN	1634	35.406	8.82E-07	6.0543	1.86E-06	P10 - ctrl; P3 - ctrl; P8 - ctrl; P10 - P11; P10 - P2; P3 - P10; P3 - P11; P8 - P11; P3 - P2; P8 - P2; P3 - P8
TC0800012011.hg.1	TC0800012011.hg.1	spazyby.aAug10	spazyby	NA	35.389	8.85E-07	6.0531	1.87E-06	ctrl - P10; ctrl - P11; P2 - ctrl; ctrl - P3; ctrl - P8; P2 - P10; P3 - P10; P2 - P11; P3 - P11; P11 - P8; P2 - P3; P2 - P8; P3 - P8
TC1000011420.hg.1	TC1000011420.hg.1	NM_001178137	CPEB3	22849	35.298	8.97E-07	6.047	1.89E-06	ctrl - P10; ctrl - P2; ctrl - P3; ctrl - P8; P11 - P10; P2 - P10; P11 - P2; P11 - P3; P11 - P8

TC0500007891.hg.1	TC0500007891.hg.1	torstawby.aAug10-unspliced	torstawby	NA	35.261	9.03E-07	6.0445	1.90E-06	ctrl - P10; ctrl - P11; ctrl - P3; ctrl - P8; P2 - P10; P10 - P8; P2 - P11; P11 - P8; P2 - P3; P2 - P8; P3 - P8
TC1500009478.hg.1	TC1500009478.hg.1	NM_001277176	WDR72	256764	35.204	9.11E-07	6.0406	1.92E-06	ctrl - P10; ctrl - P3; ctrl - P8; P11 - P10; P2 - P10; P11 - P3; P11 - P8; P2 - P3; P2 - P8
TC0400008054.hg.1	TC0400008054.hg.1	soflor.aAug10-unspliced	soflor	NA	35.193	9.12E-07	6.0398	1.92E-06	ctrl - P10; ctrl - P11; P2 - ctrl; ctrl - P3; ctrl - P8; P2 - P10; P3 - P10; P10 - P8; P2 - P11; P3 - P11; P11 - P8; P2 - P3; P2 - P8; P3 - P8
TC0600008231.hg.1	TC0600008231.hg.1	NM_003221	TFAP2B	7021	35.159	9.17E-07	6.0375	1.93E-06	ctrl - P10; ctrl - P2; ctrl - P3; ctrl - P8; P11 - P10; P2 - P10; P11 - P2; P11 - P3; P11 - P8; P2 - P8
TC0500008890.hg.1	TC0500008890.hg.1	NM_002588	PCDHGC3	5098	34.934	9.51E-07	6.022	1.99E-06	P10 - ctrl; P11 - ctrl; ctrl - P2; P3 - ctrl; P8 - ctrl; P10 - P11; P10 - P2; P10 - P3; P11 - P2; P3 - P11; P8 - P11; P3 - P2; P8 - P2; P8 - P3
TC0300010476.hg.1	TC0300010476.hg.1	NM_001012409	SGOL1	151648	34.93	9.51E-07	6.0218	1.99E-06	P10 - ctrl; ctrl - P11; ctrl - P2; ctrl - P3; P8 - ctrl; P10 - P11; P10 - P2; P10 - P3; P8 - P10; P8 - P11; P8 - P2; P8 - P3
TC0200016651.hg.1	TC0200016651.hg.1	NM_000104	CYP1B1	1545	34.904	9.55E-07	6.02	2.00E-06	ctrl - P10; P11 - ctrl; ctrl - P8; P11 - P10; P2 - P10; P3 - P10; P11 - P2; P11 - P3; P11 - P8; P2 - P8; P3 - P8
TC0900007808.hg.1	TC0900007808.hg.1	parteyby.aAug10-unspliced	parteyby	NA	34.897	9.56E-07	6.0195	2.00E-06	ctrl - P10; ctrl - P11; P2 - ctrl; ctrl - P3; ctrl - P8; P2 - P10; P2 - P11; P2 - P3; P2 - P8; P3 - P8
TC0700007043.hg.1	TC0700007043.hg.1	kluky.aAug10-unspliced	kluky	NA	34.876	9.59E-07	6.018	2.01E-06	ctrl - P10; ctrl - P8; P11 - P10; P2 - P10; P3 - P10; P11 - P8; P2 - P8; P3 - P8
TC1300010011.hg.1	TC1300010011.hg.1	NM_001278674	LINC00452	643365	34.86	9.62E-07	6.0169	2.01E-06	P10 - ctrl; P2 - ctrl; P3 - ctrl; P8 - ctrl; P10 - P11; P10 - P3; P10 - P8; P2 - P11; P3 - P11; P8 - P11; P2 - P8
TC0100009147.hg.1	TC0100009147.hg.1	swawplo.aAug10-unspliced	swawplo	NA	34.858	9.62E-07	6.0168	2.01E-06	ctrl - P10; ctrl - P11; P2 - ctrl; ctrl - P3; ctrl - P8; P11 - P10; P2 - P10; P2 - P11; P11 - P8; P2 - P3; P2 - P8
TC0700006605.hg.1	TC0700006605.hg.1	NA	NA	NA	34.847	9.64E-07	6.0161	2.01E-06	ctrl - P10; ctrl - P11; ctrl - P3; ctrl - P8; P11 - P10; P2 - P10; P3 - P10; P10 - P8; P2 - P11; P11 - P8; P2 - P3; P2 - P8; P3 - P8
TC0300012145.hg.1	TC0300012145.hg.1	NM_007085	FSTL1	11167	34.822	9.68E-07	6.0143	2.02E-06	ctrl - P10; ctrl - P11; ctrl - P2; ctrl - P3; P10 - P2; P10 - P3; P8 - P10; P11 - P2; P8 - P11; P8 - P2; P8 - P3
TC0100007969.hg.1	TC0100007969.hg.1	NR_029846	MIR30E	407034	34.808	9.70E-07	6.0134	2.02E-06	ctrl - P10; ctrl - P11; ctrl - P3; ctrl - P8; P11 - P10; P2 - P10; P10 - P8; P2 - P11; P11 - P3; P11 - P8; P2 - P3; P2 - P8; P3 - P8
TC0600009146.hg.1	TC0600009146.hg.1	ENST00000607386.1	RP5-1112D6.7		34.776	9.75E-07	6.0112	2.03E-06	ctrl - P10; ctrl - P11; P2 - ctrl; ctrl - P8; P11 - P10; P2 - P10; P3 - P10; P2 - P11; P3 - P11; P2 - P3; P2 - P8; P3 - P8
TC0400010211.hg.1	TC0400010211.hg.1	NR_107018	MIR7978	1.02E+08	34.704	9.86E-07	6.0062	2.05E-06	ctrl - P10; ctrl - P11; ctrl - P2; ctrl - P3; ctrl - P8; P11 - P10; P2 - P10; P11 - P2; P11 - P3; P11 - P8; P2 - P8
TC0300007274.hg.1	TC0300007274.hg.1	nuhira.aAug10-unspliced	nuhira	NA	34.67	9.91E-07	6.0038	2.06E-06	ctrl - P10; ctrl - P11; ctrl - P2; ctrl - P3; ctrl - P8; P2 - P10; P2 - P3; P2 - P8
TC1700010232.hg.1	TC1700010232.hg.1	slarsmy.aAug10-unspliced	slarsmy	NA	34.632	9.97E-07	6.0012	2.07E-06	ctrl - P10; ctrl - P11; ctrl - P3; ctrl - P8; P11 - P10; P2 - P10; P3 - P10; P8 - P10; P2 - P11; P2 - P3; P2 - P8; P3 - P8
TC0200014991.hg.1	TC0200014991.hg.1	NM_001033045	GPR155	151556	34.555	1.01E-06	5.9958	2.09E-06	ctrl - P10; ctrl - P2; ctrl - P3; ctrl - P8; P11 - P10; P2 - P10; P3 - P10; P11 - P2; P11 - P3; P11 - P8; P2 - P8; P3 - P8
TC1100012438.hg.1	TC1100012438.hg.1	skeylerby.aAug10-unspliced	skeylerby	NA	34.525	1.01E-06	5.9938	2.10E-06	ctrl - P10; ctrl - P11; ctrl - P3; ctrl - P8; P11 - P10; P2 - P10; P3 - P10; P10 - P8; P2 - P11; P11 - P8; P2 - P3; P2 - P8; P3 - P8
TC0700012586.hg.1	TC0700012586.hg.1	sheebler.aAug10-unspliced	sheebler	NA	34.52	1.02E-06	5.9934	2.10E-06	ctrl - P10; ctrl - P11; P2 - ctrl; ctrl - P8; P10 - P11; P2 - P10; P10 - P8; P2 - P11; P3 - P11; P2 - P3; P2 - P8; P3 - P8
TC0900009675.hg.1	TC0900009675.hg.1	NR_039685	MIR4474	1.01E+08	34.478	1.02E-06	5.9905	2.11E-06	ctrl - P10; ctrl - P11; P2 - ctrl; ctrl - P8; P2 - P10; P3 - P10; P10 - P8; P2 - P11; P3 - P11; P11 - P8; P2 - P3; P2 - P8; P3 - P8
TC1800006594.hg.1	TC1800006594.hg.1	ENST00000584036.1	RP11-678G15.1		34.465	1.02E-06	5.9896	2.12E-06	ctrl - P10; ctrl - P11; ctrl - P3; ctrl - P8; P2 - P10; P3 - P10; P2 - P11; P3 - P11; P11 - P8; P2 - P3; P2 - P8; P3 - P8
TC1500008131.hg.1	TC1500008131.hg.1	NA	NA	NA	34.416	1.03E-06	5.9862	2.13E-06	ctrl - P10; ctrl - P3; ctrl - P8; P11 - P10; P2 - P10; P11 - P3; P11 - P8; P2 - P3; P2 - P8; P3 - P8
TC1400007540.hg.1	TC1400007540.hg.1	swortoy.aAug10	swortoy	NA	34.409	1.03E-06	5.9857	2.13E-06	P10 - ctrl; P11 - ctrl; P3 - ctrl; P8 - ctrl; P10 - P11; P10 - P2; P10 - P3; P10 - P8; P11 - P2; P11 - P3; P3 - P2; P8 - P2; P8 - P3
TC1400008632.hg.1	TC1400008632.hg.1	NR_003029	SNORD9	14q11.2	34.385	1.04E-06	5.984	2.14E-06	P11 - ctrl; P2 - ctrl; P3 - ctrl; P11 - P10; P2 - P10; P3 - P10; P11 - P8; P2 - P8; P3 - P8
TC0900007387.hg.1	TC0900007387.hg.1	ENST00000432011	RP11-236F9.2		34.329	1.05E-06	5.9801	2.16E-06	P10 - ctrl; P3 - ctrl; P8 - ctrl; P10 - P11; P10 - P2; P3 - P10; P3 - P11; P8 - P11; P3 - P2; P8 - P2
TC1800007420.hg.1	TC1800007420.hg.1	tawry.aAug10-unspliced	tawry	NA	34.293	1.05E-06	5.9775	2.17E-06	ctrl - P10; ctrl - P11; ctrl - P3; ctrl - P8; P11 - P10; P2 - P10; P2 - P11; P11 - P3; P11 - P8; P2 - P3; P2 - P8; P3 - P8
TC0700008292.hg.1	TC0700008292.hg.1	NM_012449	STEAP1	26872	34.291	1.05E-06	5.9775	2.17E-06	P10 - ctrl; P3 - ctrl; P8 - ctrl; P10 - P11; P10 - P2; P3 - P10; P8 - P10; P3 - P11; P8 - P11; P3 - P2; P8 - P2
TC0300013424.hg.1	TC0300013424.hg.1	NR_049838	MIR548AQ	1.01E+08	34.253	1.06E-06	5.9748	2.18E-06	ctrl - P10; ctrl - P11; P2 - ctrl; ctrl - P3; ctrl - P8; P2 - P10; P3 - P10; P2 - P11; P2 - P3; P2 - P8; P3 - P8
TC0400008240.hg.1	TC0400008240.hg.1	NM_001306151	DAPP1	27071	34.195	1.07E-06	5.9707	2.20E-06	P10 - ctrl; ctrl - P11; ctrl - P2; P8 - ctrl; P10 - P11; P10 - P2; P10 - P3; P11 - P2; P3 - P11; P8 - P11; P3 - P2; P8 - P2; P8 - P3
TC1500006587.hg.1	TC1500006587.hg.1	ENST00000365073	SNORD115-10	15q11.2	34.168	1.07E-06	5.9688	2.20E-06	P10 - ctrl; P11 - ctrl; P2 - ctrl; P3 - ctrl; P8 - ctrl; P10 - P11; P10 - P2; P3 - P10; P3 - P11; P8 - P11; P3 - P2; P8 - P2; P3 - P8
TC0800011542.hg.1	TC0800011542.hg.1	klobly.aAug10-unspliced	klobly	NA	34.121	1.08E-06	5.9655	2.22E-06	ctrl - P10; ctrl - P11; P2 - ctrl; ctrl - P8; P2 - P10; P3 - P10; P10 - P8; P2 - P11; P3 - P11; P11 - P8; P2 - P3; P2 - P8; P3 - P8

TC0200009757.hg.1	TC0200009757.hg.1	NA	NA	NA	34.092	1.09E-06	5.9635	2.23E-06	ctrl - P10; ctrl - P11; P2 - ctrl; ctrl - P3; ctrl - P8; P11 - P10; P2 - P10; P3 - P10; P2 - P11; P11 - P8; P2 - P3; P2 - P8; P3 - P8
TC1300009731.hg.1	TC1300009731.hg.1	hsa_circ_0002531	FAM155A	NA	34.03	1.10E-06	5.9591	2.25E-06	ctrl - P10; ctrl - P11; P2 - ctrl; ctrl - P8; P11 - P10; P2 - P10; P3 - P10; P10 - P8; P2 - P11; P3 - P11; P11 - P8; P2 - P3; P2 - P8; P3 - P8
TC0800007312.hg.1	TC0800007312.hg.1	ENST00000622942	RP11-205M5.3		34.001	1.10E-06	5.9571	2.26E-06	P10 - ctrl; P11 - ctrl; P8 - ctrl; P10 - P2; P10 - P3; P11 - P2; P11 - P3; P8 - P2; P8 - P3
TC1200009526.hg.1	TC1200009526.hg.1	nychy.aAug10-unspliced	nychy	NA	33.997	1.10E-06	5.9568	2.26E-06	ctrl - P10; ctrl - P11; ctrl - P3; ctrl - P8; P11 - P10; P2 - P10; P3 - P10; P2 - P11; P11 - P8; P2 - P3; P2 - P8; P3 - P8
TC0900012047.hg.1	TC0900012047.hg.1	NA	NA	NA	33.993	1.11E-06	5.9565	2.26E-06	P10 - ctrl; P11 - ctrl; P3 - ctrl; P8 - ctrl; P10 - P11; P10 - P2; P10 - P3; P11 - P2; P8 - P11; P3 - P2; P8 - P2; P8 - P3
TC1000010305.hg.1	TC1000010305.hg.1	zunoyby.aAug10-unspliced	zunoyby	NA	33.91	1.12E-06	5.9506	2.29E-06	ctrl - P10; ctrl - P11; P2 - ctrl; ctrl - P8; P2 - P10; P3 - P10; P2 - P11; P3 - P11; P2 - P3; P2 - P8; P3 - P8
TC1000010294.hg.1	TC1000010294.hg.1	TRNAL44P.aAug10-unspliced	TRNAL44P	NA	33.871	1.13E-06	5.9479	2.30E-06	ctrl - P10; ctrl - P11; P2 - ctrl; ctrl - P3; ctrl - P8; P2 - P10; P3 - P10; P2 - P11; P3 - P11; P11 - P8; P2 - P3; P2 - P8; P3 - P8
TC0900008865.hg.1	TC0900008865.hg.1	NM_001286760	CERCAM	51148	33.871	1.13E-06	5.9479	2.30E-06	P10 - ctrl; ctrl - P2; P3 - ctrl; P8 - ctrl; P10 - P11; P10 - P2; P3 - P11; P8 - P11; P3 - P2; P8 - P2
TC0800007232.hg.1	TC0800007232.hg.1	jerbeyby.bAug10-unspliced	jerbeyby	NA	33.863	1.13E-06	5.9473	2.30E-06	ctrl - P10; ctrl - P11; ctrl - P3; ctrl - P8; P11 - P10; P2 - P10; P3 - P10; P2 - P11; P11 - P8; P2 - P3; P2 - P8; P3 - P8
TC0800011719.hg.1	TC0800011719.hg.1	zokor.aAug10-unspliced	zokor	NA	33.85	1.13E-06	5.9464	2.30E-06	ctrl - P10; ctrl - P11; P2 - ctrl; ctrl - P8; P11 - P10; P2 - P10; P3 - P10; P2 - P11; P3 - P11; P11 - P8; P2 - P3; P2 - P8; P3 - P8
TC1300006601.hg.1	TC1300006601.hg.1	NM_001166271	SPATA13	221178	33.811	1.14E-06	5.9436	2.31E-06	ctrl - P10; ctrl - P3; ctrl - P8; P11 - P10; P2 - P10; P11 - P3; P11 - P8; P2 - P3; P2 - P8
TC0200007415.hg.1	TC0200007415.hg.1	feela.aAug10-unspliced	feela	NA	33.766	1.15E-06	5.9404	2.33E-06	ctrl - P10; ctrl - P11; ctrl - P3; ctrl - P8; P2 - P10; P10 - P8; P2 - P11; P11 - P3; P11 - P8; P2 - P3; P2 - P8; P3 - P8
TC0400007232.hg.1	TC0400007232.hg.1	NM_001104629	C4orf19	55286	33.754	1.15E-06	5.9396	2.33E-06	P10 - ctrl; ctrl - P2; ctrl - P3; P8 - ctrl; P10 - P11; P10 - P2; P10 - P3; P11 - P2; P11 - P3; P8 - P11; P8 - P2; P8 - P3
TC0700008351.hg.1	TC0700008351.hg.1	NM_004126	GNG11	2791	33.737	1.15E-06	5.9384	2.34E-06	P10 - ctrl; P3 - ctrl; P8 - ctrl; P10 - P11; P10 - P2; P8 - P10; P3 - P11; P8 - P11; P3 - P2; P8 - P2; P8 - P3
TC1200009739.hg.1	TC1200009739.hg.1	ENST00000602946.1	RP5-940J5.9		33.73	1.15E-06	5.9379	2.34E-06	P10 - ctrl; P11 - ctrl; P3 - ctrl; P8 - ctrl; P10 - P11; P10 - P2; P10 - P3; P10 - P8; P11 - P2; P3 - P2; P8 - P2
TC1300008093.hg.1	TC1300008093.hg.1	lafeyby.aAug10-unspliced	lafeyby	NA	33.711	1.16E-06	5.9366	2.34E-06	ctrl - P10; ctrl - P11; ctrl - P3; ctrl - P8; P11 - P10; P2 - P10; P2 - P11; P11 - P8; P2 - P3; P2 - P8
TC1300009993.hg.1	TC1300009993.hg.1	NM_001159596	BIVM	54841	33.701	1.16E-06	5.9358	2.34E-06	ctrl - P10; ctrl - P2; ctrl - P3; ctrl - P8; P11 - P10; P2 - P10; P3 - P10; P11 - P3; P11 - P8; P2 - P3; P2 - P8; P3 - P8
TC0400011506.hg.1	TC0400011506.hg.1	perglu.aAug10-unspliced	perglu	NA	33.66	1.17E-06	5.9329	2.36E-06	ctrl - P10; ctrl - P11; ctrl - P3; ctrl - P8; P11 - P10; P2 - P10; P2 - P11; P11 - P8; P2 - P3; P2 - P8; P3 - P8
TC2000006756.hg.1	TC2000006756.hg.1	NM_001033087	MACROD2	140733	33.583	1.18E-06	5.9274	2.39E-06	P10 - ctrl; P11 - ctrl; ctrl - P2; ctrl - P3; P8 - ctrl; P10 - P11; P10 - P2; P10 - P3; P11 - P2; P11 - P3; P8 - P2; P8 - P3
TC1100011761.hg.1	TC1100011761.hg.1	zorgleebu.aAug10-unspliced	zorgleebu	NA	33.574	1.18E-06	5.9268	2.39E-06	ctrl - P10; ctrl - P8; P11 - P10; P2 - P10; P3 - P10; P11 - P8; P2 - P8; P3 - P8
TC0500007454.hg.1	TC0500007454.hg.1	snerplyby.aAug10-unspliced	snerplyby	NA	33.57	1.18E-06	5.9265	2.39E-06	ctrl - P10; ctrl - P11; P2 - ctrl; ctrl - P3; ctrl - P8; P2 - P10; P10 - P8; P2 - P11; P11 - P8; P2 - P3; P2 - P8; P3 - P8
TC0800008324.hg.1	TC0800008324.hg.1	NM_002380	MATN2	4147	33.567	1.19E-06	5.9263	2.39E-06	P10 - ctrl; P3 - ctrl; P8 - ctrl; P10 - P11; P10 - P2; P3 - P10; P8 - P10; P3 - P11; P8 - P11; P3 - P2; P8 - P2
TC0500011652.hg.1	TC0500011652.hg.1	lyju.aAug10-unspliced	lyju	NA	33.565	1.19E-06	5.9261	2.39E-06	ctrl - P11; P2 - ctrl; ctrl - P8; P10 - P11; P2 - P10; P10 - P8; P2 - P11; P3 - P11; P2 - P3; P2 - P8; P3 - P8
TC1700012436.hg.1	TC1700012436.hg.1	ENST00000577267.1	RP11-159D12.2		33.559	1.19E-06	5.9257	2.39E-06	ctrl - P10; ctrl - P11; ctrl - P3; ctrl - P8; P11 - P10; P2 - P10; P3 - P10; P2 - P11; P11 - P8; P2 - P3; P2 - P8; P3 - P8
TC1400006438.hg.1	TC1400006438.hg.1	NA	NA	NA	33.524	1.19E-06	5.9232	2.40E-06	ctrl - P11; P2 - ctrl; P3 - ctrl; ctrl - P8; P10 - P11; P2 - P10; P3 - P10; P10 - P8; P2 - P11; P3 - P11; P2 - P3; P2 - P8; P3 - P8
TC0600013642.hg.1	TC0600013642.hg.1	peefla.aAug10-unspliced	peefla	NA	33.514	1.20E-06	5.9225	2.40E-06	ctrl - P10; ctrl - P11; P2 - ctrl; ctrl - P8; P11 - P10; P2 - P10; P3 - P10; P2 - P11; P3 - P11; P11 - P8; P2 - P3; P2 - P8; P3 - P8
TC0200007023.hg.1	TC0200007023.hg.1	swawgloy.aAug10-unspliced	swawgloy	NA	33.474	1.20E-06	5.9196	2.41E-06	ctrl - P10; ctrl - P11; ctrl - P3; ctrl - P8; P11 - P10; P2 - P10; P2 - P11; P11 - P8; P2 - P3; P2 - P8; P3 - P8
TC1700008862.hg.1	TC1700008862.hg.1	NM_001006638	RAB37	326624	33.473	1.20E-06	5.9195	2.41E-06	P10 - ctrl; P3 - ctrl; P8 - ctrl; P10 - P11; P10 - P2; P10 - P3; P3 - P11; P8 - P11; P3 - P2; P8 - P2; P8 - P3
TC0900011755.hg.1	TC0900011755.hg.1	NM_001145106	FIBCD1	84929	33.461	1.21E-06	5.9187	2.41E-06	P10 - ctrl; ctrl - P2; P3 - ctrl; P8 - ctrl; P10 - P11; P10 - P2; P3 - P10; P3 - P11; P8 - P11; P3 - P2; P8 - P2; P3 - P8
TC1700010653.hg.1	TC1700010653.hg.1	NM_001165252	KRTAP2-3	730755	33.453	1.21E-06	5.9182	2.42E-06	P10 - ctrl; P3 - ctrl; P8 - ctrl; P10 - P11; P10 - P2; P10 - P3; P8 - P10; P3 - P11; P8 - P11; P3 - P2; P8 - P2; P8 - P3
TC0800007668.hg.1	TC0800007668.hg.1	NA	NA	NA	33.332	1.23E-06	5.9095	2.46E-06	ctrl - P11; ctrl - P8; P10 - P11; P3 - P10; P10 - P8; P2 - P11; P3 - P11; P8 - P11; P3 - P2; P2 - P8; P3 - P8
TC2000007240.hg.1	TC2000007240.hg.1	NA	NA	NA	33.314	1.24E-06	5.9082	2.47E-06	ctrl - P10; ctrl - P11; ctrl - P3; ctrl - P8; P11 - P10; P2 - P10; P3 - P10; P2 - P11; P11 - P3; P11 - P8; P2 - P3; P2 - P8; P3 - P8
TC0300006662.hg.1	TC0300006662.hg.1	NA	NA	NA	33.287	1.24E-06	5.9062	2.48E-06	P10 - ctrl; P3 - ctrl; P8 - ctrl; P10 - P11; P10 - P2; P10 - P3; P3 - P11; P8 - P11; P3 - P2; P8 - P2; P8 - P3
TC0200012166.hg.1	TC0200012166.hg.1	feyfa.aAug10-unspliced	feyfa	NA	33.166	1.27E-06	5.8975	2.53E-06	P2 - ctrl; P3 - ctrl; P2 - P10; P3 - P10; P2 - P11; P3 - P11; P3 - P2; P2 - P8; P3 - P8

TC1500010546.hg.1	TC1500010546.hg.1	OTTHUMT00000474289	RP11-327J17.9		33.152	1.27E-06	5.8965	2.53E-06	ctrl - P10; ctrl - P11; ctrl - P3; ctrl - P8; P11 - P10; P2 - P10; P11 - P3; P11 - P8; P2 - P3; P2 - P8
TC1000011114.hg.1	TC1000011114.hg.1	NM_001014797	KCNMA1	3778	33.139	1.27E-06	5.8956	2.53E-06	ctrl - P10; P11 - ctrl; ctrl - P2; P3 - ctrl; ctrl - P8; P11 - P10; P3 - P10; P11 - P2; P3 - P11; P11 - P8; P3 - P2; P3 - P8
TC0900012075.hg.1	TC0900012075.hg.1	kuwaya.aAug10-unspliced	kuwaya	NA	33.04	1.29E-06	5.8884	2.57E-06	ctrl - P10; ctrl - P11; P2 - ctrl; ctrl - P3; ctrl - P8; P2 - P10; P3 - P10; P2 - P11; P11 - P8; P2 - P3; P2 - P8; P3 - P8
TC0100017534.hg.1	TC0100017534.hg.1	NM_002221	ITPKB	3707	33.031	1.30E-06	5.8877	2.57E-06	ctrl - P10; P11 - ctrl; ctrl - P2; ctrl - P3; ctrl - P8; P11 - P10; P11 - P2; P11 - P3; P11 - P8
TC1800008237.hg.1	TC1800008237.hg.1	sorplaw.aAug10-unspliced	sorplaw	NA	32.998	1.30E-06	5.8854	2.59E-06	ctrl - P10; ctrl - P11; ctrl - P3; ctrl - P8; P11 - P10; P2 - P10; P2 - P11; P11 - P8; P2 - P3; P2 - P8
TC0800008033.hg.1	TC0800008033.hg.1	slarkor.aAug10-unspliced	slarkor	NA	32.913	1.32E-06	5.8792	2.62E-06	ctrl - P10; ctrl - P11; ctrl - P3; ctrl - P8; P2 - P10; P3 - P10; P2 - P11; P2 - P3; P2 - P8; P3 - P8
TC0300012975.hg.1	TC0300012975.hg.1	NM_001038628	B3GALNT1	8706	32.863	1.33E-06	5.8756	2.64E-06	P10 - ctrl; P3 - ctrl; P8 - ctrl; P10 - P11; P10 - P2; P10 - P3; P8 - P10; P3 - P11; P8 - P11; P3 - P2; P8 - P2; P8 - P3
TC0400008063.hg.1	TC0400008063.hg.1	zyswuby.aAug10-unspliced	zyswuby	NA	32.846	1.34E-06	5.8743	2.65E-06	ctrl - P10; ctrl - P11; ctrl - P3; ctrl - P8; P2 - P10; P3 - P10; P10 - P8; P2 - P11; P3 - P11; P2 - P3; P2 - P8; P3 - P8
TC0400010789.hg.1	TC0400010789.hg.1	pergaw.aAug10-unspliced	pergaw	NA	32.823	1.34E-06	5.8727	2.65E-06	ctrl - P10; ctrl - P11; ctrl - P8; P2 - P10; P3 - P10; P10 - P8; P2 - P11; P3 - P11; P11 - P8; P2 - P8; P3 - P8
TC0Y00007293.hg.1	TC0Y00007293.hg.1	NA	NA	NA	32.796	1.35E-06	5.8707	2.66E-06	ctrl - P10; ctrl - P11; ctrl - P2; ctrl - P8; P11 - P10; P3 - P10; P11 - P2; P3 - P11; P3 - P2; P3 - P8
TC1100007017.hg.1	TC1100007017.hg.1	NM_153347	TMEM86A	144110	32.78	1.35E-06	5.8695	2.67E-06	ctrl - P10; ctrl - P2; ctrl - P3; ctrl - P8; P11 - P10; P10 - P3; P11 - P2; P11 - P3; P11 - P8; P2 - P3; P8 - P3
TC1100007049.hg.1	TC1100007049.hg.1	NM_001098520	HTATIP2	10553	32.76	1.36E-06	5.868	2.68E-06	P10 - ctrl; ctrl - P11; ctrl - P2; P8 - ctrl; P10 - P11; P10 - P2; P10 - P3; P3 - P11; P8 - P11; P3 - P2; P8 - P2; P8 - P3
TC1000011951.hg.1	TC1000011951.hg.1	NR_002791	EMX2OS	10q26.1	32.749	1.36E-06	5.8673	2.68E-06	ctrl - P10; ctrl - P2; ctrl - P3; ctrl - P8; P11 - P10; P2 - P10; P10 - P8; P11 - P2; P11 - P3; P11 - P8; P2 - P3; P2 - P8; P3 - P8
TC1700008101.hg.1	TC1700008101.hg.1	ENST00000582765	RP11-995C19.2	NA	32.723	1.36E-06	5.8654	2.69E-06	ctrl - P10; ctrl - P11; ctrl - P3; ctrl - P8; P2 - P10; P3 - P10; P2 - P11; P11 - P8; P2 - P3; P2 - P8; P3 - P8
TC0700013384.hg.1	TC0700013384.hg.1	ENST00000609770.1	RP11-166O4.6		32.688	1.37E-06	5.8628	2.70E-06	ctrl - P10; ctrl - P11; ctrl - P3; ctrl - P8; P11 - P10; P2 - P10; P2 - P11; P11 - P3; P11 - P8; P2 - P3; P2 - P8
TC1200011961.hg.1	TC1200011961.hg.1	NM_001193453	TMEM116	89894	32.599	1.39E-06	5.8563	2.74E-06	ctrl - P10; ctrl - P2; ctrl - P3; ctrl - P8; P11 - P10; P10 - P3; P11 - P2; P11 - P3; P11 - P8
TC0600014366.hg.1	TC0600014366.hg.1	NM_001130690	PDE10A	10846	32.493	1.42E-06	5.8485	2.79E-06	P10 - ctrl; P3 - ctrl; P8 - ctrl; P10 - P11; P10 - P2; P8 - P10; P3 - P11; P8 - P11; P3 - P2; P8 - P2; P8 - P3
TC1100011540.hg.1	TC1100011540.hg.1	darmoy.aAug10-unspliced	darmoy	NA	32.485	1.42E-06	5.848	2.79E-06	ctrl - P10; ctrl - P11; ctrl - P3; ctrl - P8; P11 - P10; P2 - P10; P3 - P10; P2 - P11; P11 - P8; P2 - P3; P2 - P8; P3 - P8
TC1100010418.hg.1	TC1100010418.hg.1	NM_031217	KIF18A	81930	32.438	1.43E-06	5.8445	2.81E-06	P10 - ctrl; ctrl - P2; P3 - ctrl; P8 - ctrl; P10 - P11; P10 - P2; P8 - P10; P11 - P2; P3 - P11; P8 - P11; P3 - P2; P8 - P2; P8 - P3
TC1300007164.hg.1	TC1300007164.hg.1	NM_001160308	SETDB2	83852	32.424	1.43E-06	5.8434	2.81E-06	ctrl - P10; P11 - ctrl; ctrl - P2; ctrl - P3; ctrl - P8; P11 - P10; P2 - P10; P11 - P2; P11 - P3; P11 - P8; P2 - P3; P2 - P8
TC0X00010375.hg.1	TC0X00010375.hg.1	NM_001011657	ZMAT1	84460	32.421	1.43E-06	5.8432	2.81E-06	ctrl - P10; ctrl - P11; ctrl - P3; ctrl - P8; P11 - P10; P2 - P10; P3 - P10; P2 - P11; P11 - P8; P2 - P3; P2 - P8; P3 - P8
TC0200011748.hg.1	TC0200011748.hg.1	uc061gsa.1	AC018463.1	NA	32.402	1.44E-06	5.8418	2.82E-06	ctrl - P10; ctrl - P11; P2 - ctrl; ctrl - P8; P2 - P10; P3 - P10; P10 - P8; P2 - P11; P3 - P11; P2 - P3; P2 - P8; P3 - P8
TC0400011000.hg.1	TC0400011000.hg.1	soso.aAug10-unspliced	soso	NA	32.376	1.45E-06	5.8399	2.83E-06	ctrl - P10; ctrl - P11; ctrl - P3; ctrl - P8; P2 - P10; P3 - P10; P2 - P11; P11 - P8; P2 - P3; P2 - P8; P3 - P8
TC0600008347.hg.1	TC0600008347.hg.1	NA	NA	NA	32.368	1.45E-06	5.8393	2.83E-06	ctrl - P10; ctrl - P11; ctrl - P3; ctrl - P8; P11 - P10; P2 - P10; P3 - P10; P10 - P8; P2 - P11; P11 - P8; P2 - P3; P2 - P8; P3 - P8
TC0600011116.hg.1	TC0600011116.hg.1	NM_001098486	SLC17A3	10786	32.314	1.46E-06	5.8353	2.86E-06	ctrl - P10; ctrl - P11; P2 - ctrl; P3 - ctrl; P2 - P10; P3 - P10; P2 - P11; P3 - P11; P3 - P2; P2 - P8; P3 - P8
TSUnmapped00000012.hg.1	TSUnmapped00000012.hg.1	ENST00000625211	LRP6	4040	32.302	1.46E-06	5.8344	2.86E-06	ctrl - P10; ctrl - P2; ctrl - P3; ctrl - P8; P11 - P10; P2 - P10; P11 - P3; P11 - P8; P2 - P3; P2 - P8
TC1300008944.hg.1	TC1300008944.hg.1	shafarby.aAug10-unspliced	shafarby	NA	32.284	1.47E-06	5.8331	2.87E-06	ctrl - P10; P2 - ctrl; ctrl - P8; P11 - P10; P2 - P10; P3 - P10; P10 - P8; P2 - P11; P11 - P8; P2 - P3; P2 - P8; P3 - P8
TC0100017292.hg.1	TC0100017292.hg.1	smabo.aAug10-unspliced	smabo	NA	32.255	1.48E-06	5.8309	2.88E-06	ctrl - P10; ctrl - P11; ctrl - P8; P2 - P10; P3 - P10; P2 - P11; P3 - P11; P2 - P3; P2 - P8; P3 - P8
TC0400012639.hg.1	TC0400012639.hg.1	ENST00000384502	Y_RNA		32.222	1.48E-06	5.8285	2.89E-06	ctrl - P10; ctrl - P11; ctrl - P3; ctrl - P8; P11 - P10; P2 - P10; P3 - P10; P2 - P11; P11 - P8; P2 - P3; P2 - P8; P3 - P8
TC0900008633.hg.1	TC0900008633.hg.1	uc064vli.1	AL161784.1	NA	32.19	1.49E-06	5.8262	2.91E-06	P10 - ctrl; P11 - ctrl; P3 - ctrl; P8 - ctrl; P10 - P11; P10 - P2; P10 - P3; P11 - P2; P8 - P11; P3 - P2; P8 - P2; P8 - P3
TC0300011413.hg.1	TC0300011413.hg.1	kasore.aAug10-unspliced	kasore	NA	32.173	1.50E-06	5.8249	2.91E-06	ctrl - P10; ctrl - P11; ctrl - P3; ctrl - P8; P2 - P10; P2 - P11; P11 - P8; P2 - P3; P2 - P8; P3 - P8
TC0800009302.hg.1	TC0800009302.hg.1	ENST00000520139	AC144568.4	NA	32.172	1.50E-06	5.8248	2.91E-06	ctrl - P10; ctrl - P11; P2 - ctrl; ctrl - P8; P11 - P10; P2 - P10; P3 - P10; P2 - P11; P11 - P8; P2 - P3; P2 - P8; P3 - P8
TC0100009621.hg.1	TC0100009621.hg.1	NM_006623	PHGDH	26227	32.168	1.50E-06	5.8245	2.91E-06	ctrl - P10; ctrl - P2; ctrl - P3; ctrl - P8; P11 - P10; P3 - P10; P11 - P2; P11 - P3; P11 - P8; P3 - P2; P3 - P8

TC1600008212.hg.1	TC1600008212.hg.1	glusmer.aAug10-unspliced	glusmer	NA	32.077	1.52E-06	5.8178	2.95E-06	ctrl - P10; ctrl - P11; ctrl - P3; ctrl - P8; P2 - P10; P10 - P8; P2 - P11; P11 - P8; P2 - P3; P2 - P8; P3 - P8
TC1500008038.hg.1	TC1500008038.hg.1	NM_014862	ARNT2	9915	32.07	1.52E-06	5.8172	2.96E-06	ctrl - P10; ctrl - P2; ctrl - P3; ctrl - P8; P11 - P10; P2 - P10; P3 - P10; P11 - P2; P11 - P3; P11 - P8; P2 - P3; P2 - P8; P3 - P8
TC0800011414.hg.1	TC0800011414.hg.1	NA	NA	NA	32.064	1.52E-06	5.8168	2.96E-06	ctrl - P10; P2 - ctrl; ctrl - P3; ctrl - P8; P11 - P10; P2 - P10; P10 - P3; P10 - P8; P2 - P11; P11 - P3; P11 - P8; P2 - P3; P2 - P8
TC1800006595.hg.1	TC1800006595.hg.1	uc060nds.1	AP005118.1	NA	32.012	1.54E-06	5.8129	2.98E-06	ctrl - P10; ctrl - P11; ctrl - P3; ctrl - P8; P2 - P10; P10 - P8; P2 - P11; P3 - P11; P2 - P3; P2 - P8; P3 - P8
TC0600014111.hg.1	TC0600014111.hg.1	NM_006772	SYNGAP1	8831	31.961	1.55E-06	5.8091	3.00E-06	ctrl - P10; ctrl - P11; ctrl - P2; ctrl - P3; ctrl - P8; P11 - P10; P2 - P10; P3 - P10; P11 - P3; P11 - P8; P2 - P3; P2 - P8
TC0400007133.hg.1	TC0400007133.hg.1	NA	NA	NA	31.931	1.56E-06	5.8068	3.02E-06	ctrl - P10; ctrl - P11; P2 - ctrl; ctrl - P8; P2 - P10; P3 - P10; P10 - P8; P2 - P11; P3 - P11; P2 - P3; P2 - P8; P3 - P8
TC0800011583.hg.1	TC0800011583.hg.1	slobly.aAug10-unspliced	slobly	NA	31.922	1.56E-06	5.8062	3.02E-06	ctrl - P10; ctrl - P11; P2 - ctrl; ctrl - P8; P2 - P10; P3 - P10; P10 - P8; P2 - P11; P3 - P11; P2 - P3; P2 - P8; P3 - P8
TC0200007999.hg.1	TC0200007999.hg.1	ZNF638.uAug10-unspliced	ZNF638	NA	31.908	1.57E-06	5.8051	3.02E-06	ctrl - P10; ctrl - P11; P2 - ctrl; ctrl - P3; ctrl - P8; P11 - P10; P2 - P10; P3 - P10; P2 - P11; P11 - P8; P2 - P3; P2 - P8; P3 - P8
TC1300006992.hg.1	TC1300006992.hg.1	NA	NA	NA	31.853	1.58E-06	5.8011	3.05E-06	ctrl - P10; ctrl - P11; ctrl - P2; ctrl - P3; ctrl - P8; P11 - P10; P2 - P10; P11 - P3; P11 - P8; P2 - P3; P2 - P8
TC0800010234.hg.1	TC0800010234.hg.1	NM_003012	SFRP1	6422	31.839	1.59E-06	5.8	3.06E-06	ctrl - P10; ctrl - P11; ctrl - P2; ctrl - P3; ctrl - P8; P11 - P10; P2 - P10; P3 - P10; P3 - P11; P11 - P8; P3 - P2; P2 - P8; P3 - P8
TC1500010929.hg.1	TC1500010929.hg.1	NM_207446	FAM174B	400451	31.833	1.59E-06	5.7995	3.06E-06	P10 - ctrl; P3 - ctrl; P8 - ctrl; P10 - P11; P10 - P2; P3 - P11; P8 - P11; P3 - P2; P8 - P2; P8 - P3
TC0100016495.hg.1	TC0100016495.hg.1	tari.aAug10-unspliced	tari	NA	31.828	1.59E-06	5.7992	3.06E-06	ctrl - P10; ctrl - P11; P2 - ctrl; ctrl - P3; ctrl - P8; P11 - P10; P2 - P10; P2 - P11; P11 - P3; P11 - P8; P2 - P3; P2 - P8
TC1000008344.hg.1	TC1000008344.hg.1	NA	NA	NA	31.812	1.59E-06	5.798	3.06E-06	ctrl - P10; ctrl - P11; P2 - ctrl; ctrl - P8; P2 - P10; P3 - P10; P2 - P11; P3 - P11; P11 - P8; P2 - P3; P2 - P8; P3 - P8
TC0600011235.hg.1	TC0600011235.hg.1	NM_003514	HIST1H2AM	8336	31.754	1.61E-06	5.7936	3.09E-06	P10 - ctrl; ctrl - P2; P8 - ctrl; P10 - P11; P10 - P2; P10 - P3; P11 - P2; P8 - P11; P3 - P2; P8 - P2; P8 - P3
TC0200009761.hg.1	TC0200009761.hg.1	loydo.aAug10-unspliced	loydo	NA	31.729	1.62E-06	5.7917	3.10E-06	ctrl - P10; ctrl - P3; ctrl - P8; P11 - P10; P2 - P10; P3 - P10; P2 - P11; P11 - P3; P11 - P8; P2 - P3; P2 - P8; P3 - P8
TC1600010741.hg.1	TC1600010741.hg.1	blawsnee.aAug10-unspliced	blawsnee	NA	31.694	1.63E-06	5.7891	3.12E-06	ctrl - P10; ctrl - P11; ctrl - P3; ctrl - P8; P11 - P10; P2 - P10; P3 - P10; P2 - P11; P11 - P8; P2 - P3; P2 - P8; P3 - P8
TC1500010549.hg.1	TC1500010549.hg.1	NA	NA	NA	31.627	1.64E-06	5.7841	3.15E-06	ctrl - P10; ctrl - P11; ctrl - P3; ctrl - P8; P11 - P10; P2 - P10; P2 - P11; P11 - P3; P11 - P8; P2 - P3; P2 - P8
TC1900006668.hg.1	TC1900006668.hg.1	dorko.aAug10-unspliced	dorko	NA	31.606	1.65E-06	5.7825	3.16E-06	P10 - ctrl; P11 - ctrl; P3 - ctrl; P8 - ctrl; P10 - P2; P10 - P3; P11 - P2; P11 - P3; P11 - P8; P8 - P2; P8 - P3
TC0600008166.hg.1	TC0600008166.hg.1	NM_001204051	SLC25A27	9481	31.603	1.65E-06	5.7823	3.16E-06	ctrl - P10; ctrl - P11; ctrl - P3; ctrl - P8; P11 - P10; P2 - P10; P3 - P10; P2 - P11; P11 - P3; P11 - P8; P2 - P3; P2 - P8; P3 - P8
TC0100015644.hg.1	TC0100015644.hg.1	NA	NA	NA	31.506	1.68E-06	5.775	3.21E-06	ctrl - P10; ctrl - P11; ctrl - P3; ctrl - P8; P11 - P10; P2 - P10; P3 - P10; P2 - P11; P11 - P8; P2 - P3; P2 - P8; P3 - P8
TC0300012065.hg.1	TC0300012065.hg.1	storbobu.aAug10-unspliced	storbobu	NA	31.486	1.68E-06	5.7734	3.22E-06	ctrl - P10; P2 - ctrl; ctrl - P3; ctrl - P8; P11 - P10; P2 - P10; P3 - P10; P10 - P8; P2 - P11; P11 - P8; P2 - P3; P2 - P8; P3 - P8
TC0600008548.hg.1	TC0600008548.hg.1	NA	NA	NA	31.47	1.69E-06	5.7722	3.23E-06	P10 - ctrl; P2 - ctrl; P8 - ctrl; P10 - P11; P2 - P10; P10 - P3; P10 - P8; P2 - P11; P8 - P11; P2 - P3; P2 - P8; P8 - P3
TC0200009607.hg.1	TC0200009607.hg.1	vudu.aAug10-unspliced	vudu	NA	31.457	1.69E-06	5.7712	3.23E-06	ctrl - P10; ctrl - P11; P2 - ctrl; ctrl - P8; P11 - P10; P2 - P10; P3 - P10; P2 - P11; P3 - P11; P11 - P8; P2 - P3; P2 - P8; P3 - P8
TC1600009425.hg.1	TC1600009425.hg.1	snoywor.aAug10-unspliced	snoywor	NA	31.44	1.70E-06	5.7699	3.24E-06	ctrl - P10; ctrl - P11; ctrl - P3; ctrl - P8; P2 - P10; P2 - P11; P11 - P8; P2 - P3; P2 - P8; P3 - P8
TC0300009676.hg.1	TC0300009676.hg.1	shermubu.aAug10-unspliced	shermubu	NA	31.438	1.70E-06	5.7697	3.24E-06	ctrl - P10; ctrl - P11; ctrl - P3; ctrl - P8; P11 - P10; P2 - P10; P2 - P11; P11 - P3; P11 - P8; P2 - P3; P2 - P8
TC2100006634.hg.1	TC2100006634.hg.1	flotar.aAug10-unspliced	flotar	NA	31.434	1.70E-06	5.7695	3.24E-06	ctrl - P10; ctrl - P11; ctrl - P3; ctrl - P8; P11 - P10; P2 - P10; P3 - P10; P11 - P3; P11 - P8; P2 - P3; P2 - P8
TC0X00009074.hg.1	TC0X00009074.hg.1	NA	NA	NA	31.423	1.70E-06	5.7686	3.24E-06	ctrl - P10; ctrl - P11; ctrl - P3; ctrl - P8; P11 - P10; P2 - P10; P2 - P11; P11 - P3; P11 - P8; P2 - P3; P2 - P8
TC0700009530.hg.1	TC0700009530.hg.1	dersmarbu.aAug10-unspliced	dersmarbu	NA	31.415	1.71E-06	5.768	3.24E-06	ctrl - P10; ctrl - P11; P2 - ctrl; ctrl - P3; ctrl - P8; P2 - P10; P2 - P11; P11 - P8; P2 - P3; P2 - P8; P3 - P8
TC1200012296.hg.1	TC1200012296.hg.1	ENST00000602988.1	RP11-214K3.21		31.397	1.71E-06	5.7667	3.25E-06	ctrl - P10; ctrl - P11; ctrl - P3; ctrl - P8; P11 - P10; P2 - P10; P3 - P10; P10 - P8; P2 - P11; P11 - P8; P2 - P3; P2 - P8; P3 - P8
TC0Y00007288.hg.1	TC0Y00007288.hg.1	wubo.aAug10-unspliced	wubo	NA	31.369	1.72E-06	5.7645	3.25E-06	ctrl - P10; ctrl - P11; ctrl - P2; ctrl - P3; ctrl - P8; P11 - P10; P3 - P10; P8 - P10; P11 - P2; P3 - P11; P3 - P2; P3 - P8
TC0Y00007296.hg.1	TC0Y00007296.hg.1	sorbo.aAug10-unspliced	sorbo	NA	31.369	1.72E-06	5.7645	3.25E-06	ctrl - P10; ctrl - P11; ctrl - P2; ctrl - P3; ctrl - P8; P11 - P10; P3 - P10; P8 - P10; P11 - P2; P3 - P11; P3 - P2; P3 - P8
TC0Y00007297.hg.1	TC0Y00007297.hg.1	snubar.aAug10-unspliced	snubar	NA	31.369	1.72E-06	5.7645	3.25E-06	ctrl - P10; ctrl - P11; ctrl - P2; ctrl - P3; ctrl - P8; P11 - P10; P3 - P10; P8 - P10; P11 - P2; P3 - P11; P3 - P2; P3 - P8
TC0Y00007301.hg.1	TC0Y00007301.hg.1	shabo.aAug10-unspliced	shabo	NA	31.369	1.72E-06	5.7645	3.25E-06	ctrl - P10; ctrl - P11; ctrl - P2; ctrl - P3; ctrl - P8; P11 - P10; P3 - P10; P8 - P10; P11 - P2; P3 - P11; P3 - P2; P3 - P8

TC0Y00007307.hg.1	TC0Y00007307.hg.1	korbo.aAug10-unspliced	korbo	NA	31.369	1.72E-06	5.7645	3.25E-06	ctrl - P10; ctrl - P11; ctrl - P2; ctrl - P3; ctrl - P8; P11 - P10; P3 - P10; P8 - P10; P11 - P2; P3 - P11; P3 - P2; P3 - P8
TC0700012917.hg.1	TC0700012917.hg.1	NM_001042482	TPK1	27010	31.364	1.72E-06	5.7642	3.25E-06	P10 - ctrl; P8 - ctrl; P10 - P11; P10 - P2; P10 - P3; P8 - P11; P8 - P2; P8 - P3
TC1100006999.hg.1	TC1100006999.hg.1	vycher.aAug10-unspliced	vycher	NA	31.358	1.72E-06	5.7637	3.25E-06	ctrl - P10; ctrl - P11; ctrl - P3; ctrl - P8; P2 - P10; P3 - P10; P2 - P11; P3 - P11; P2 - P3; P2 - P8; P3 - P8
TC0900010217.hg.1	TC0900010217.hg.1	NR_046175	LOC286297	286297	31.352	1.72E-06	5.7632	3.25E-06	P10 - ctrl; P3 - ctrl; P8 - ctrl; P10 - P11; P10 - P2; P3 - P10; P3 - P11; P8 - P11; P3 - P2; P8 - P2
TC1500010183.hg.1	TC1500010183.hg.1	OTTHUMT00000433312	RP11-81A1.6		31.351	1.73E-06	5.7632	3.25E-06	ctrl - P10; ctrl - P11; ctrl - P2; ctrl - P3; ctrl - P8; P11 - P10; P2 - P10; P3 - P10; P2 - P11; P11 - P8; P2 - P3; P2 - P8; P3 - P8
TC0200015307.hg.1	TC0200015307.hg.1	NM_018897	DNAH7	56171	31.35	1.73E-06	5.7631	3.25E-06	ctrl - P10; ctrl - P2; ctrl - P3; ctrl - P8; P11 - P10; P2 - P10; P11 - P2; P11 - P3; P11 - P8; P2 - P3; P2 - P8
TC1200011313.hg.1	TC1200011313.hg.1	hsa_circ_0000422	NAP1L1	NA	31.348	1.73E-06	5.763	3.25E-06	ctrl - P10; ctrl - P11; ctrl - P2; ctrl - P3; ctrl - P8; P11 - P10; P2 - P10; P3 - P10; P2 - P11; P3 - P11; P2 - P3; P2 - P8; P3 - P8
TC1800008624.hg.1	TC1800008624.hg.1	ENST00000625002	RP11-380M21.3		31.34	1.73E-06	5.7623	3.25E-06	ctrl - P10; ctrl - P11; P2 - ctrl; ctrl - P3; ctrl - P8; P2 - P10; P10 - P8; P2 - P11; P11 - P8; P2 - P3; P2 - P8; P3 - P8
TC0700013295.hg.1	TC0700013295.hg.1	lorsleebu.aAug10-unspliced	lorsleebu	NA	31.336	1.73E-06	5.7621	3.25E-06	ctrl - P10; ctrl - P11; P2 - ctrl; ctrl - P3; ctrl - P8; P2 - P10; P2 - P11; P11 - P8; P2 - P3; P2 - P8; P3 - P8
TC0100011630.hg.1	TC0100011630.hg.1	spawabo.aAug10-unspliced	spawabo	NA	31.335	1.73E-06	5.762	3.25E-06	ctrl - P10; ctrl - P11; P2 - ctrl; ctrl - P3; ctrl - P8; P11 - P10; P2 - P10; P3 - P10; P2 - P11; P11 - P8; P2 - P3; P2 - P8; P3 - P8
TC2000008763.hg.1	TC2000008763.hg.1	NA	NA	NA	31.32	1.73E-06	5.7608	3.26E-06	ctrl - P10; ctrl - P11; P2 - ctrl; ctrl - P3; ctrl - P8; P2 - P10; P2 - P11; P2 - P3; P2 - P8
TC2000006736.hg.1	TC2000006736.hg.1	NM_018327	SPTLC3	55304	31.253	1.76E-06	5.7557	3.30E-06	ctrl - P10; ctrl - P11; ctrl - P2; ctrl - P3; ctrl - P8; P11 - P10; P2 - P10; P10 - P3; P10 - P8; P11 - P3; P11 - P8; P2 - P3; P2 - P8
TC0600013416.hg.1	TC0600013416.hg.1	swawspeebu.aAug10-unspliced	swawspeebu	NA	31.237	1.76E-06	5.7545	3.30E-06	ctrl - P10; ctrl - P11; P2 - ctrl; ctrl - P3; ctrl - P8; P2 - P10; P3 - P10; P2 - P11; P11 - P8; P2 - P3; P2 - P8; P3 - P8
TC1400007780.hg.1	TC1400007780.hg.1	snarbeyby.aAug10-unspliced	snarbeyby	NA	31.191	1.77E-06	5.751	3.33E-06	ctrl - P10; ctrl - P11; ctrl - P3; ctrl - P8; P2 - P10; P10 - P8; P2 - P11; P11 - P8; P2 - P3; P2 - P8; P3 - P8
TC0300009524.hg.1	TC0300009524.hg.1	NM_001258315	ECT2	1894	31.171	1.78E-06	5.7495	3.34E-06	P10 - ctrl; ctrl - P11; ctrl - P2; P8 - ctrl; P10 - P11; P10 - P2; P10 - P3; P3 - P11; P8 - P11; P3 - P2; P8 - P2; P8 - P3
TC1700008252.hg.1	TC1700008252.hg.1	NA	NA	NA	31.161	1.78E-06	5.7487	3.34E-06	P10 - ctrl; P3 - ctrl; P8 - ctrl; P10 - P11; P10 - P2; P10 - P3; P3 - P11; P8 - P11; P3 - P2; P8 - P2; P8 - P3
TC0100015563.hg.1	TC0100015563.hg.1	plawmeybo.aAug10-unspliced	plawmeybo	NA	31.137	1.79E-06	5.7469	3.35E-06	ctrl - P10; ctrl - P11; P2 - ctrl; ctrl - P3; ctrl - P8; P11 - P10; P2 - P10; P3 - P10; P2 - P11; P11 - P8; P2 - P3; P2 - P8; P3 - P8
TC0900007847.hg.1	TC0900007847.hg.1	fleyteyby.aAug10-unspliced	fleyteyby	NA	31.125	1.79E-06	5.746	3.35E-06	ctrl - P10; ctrl - P11; ctrl - P3; ctrl - P8; P2 - P10; P2 - P11; P2 - P3; P2 - P8; P3 - P8
TC0700012270.hg.1	TC0700012270.hg.1	wusmoybu.aAug10-unspliced	wusmoybu	NA	31.075	1.81E-06	5.7421	3.38E-06	ctrl - P10; ctrl - P11; P2 - ctrl; ctrl - P8; P11 - P10; P2 - P10; P3 - P10; P2 - P11; P11 - P8; P2 - P3; P2 - P8; P3 - P8
TC0900008052.hg.1	TC0900008052.hg.1	slawtoby.aAug10-unspliced	slawtoby	NA	31.06	1.82E-06	5.741	3.39E-06	ctrl - P10; ctrl - P11; ctrl - P3; ctrl - P8; P2 - P10; P10 - P8; P2 - P11; P3 - P11; P2 - P3; P2 - P8; P3 - P8
TC1800008898.hg.1	TC1800008898.hg.1	NA	NA	NA	31.037	1.82E-06	5.7392	3.40E-06	ctrl - P10; ctrl - P11; ctrl - P3; ctrl - P8; P2 - P10; P10 - P3; P10 - P8; P2 - P11; P11 - P8; P2 - P3; P2 - P8
TC1600008128.hg.1	TC1600008128.hg.1	NM_144601	CMTM3	123920	31.024	1.83E-06	5.7382	3.41E-06	P10 - ctrl; P3 - ctrl; P8 - ctrl; P10 - P11; P10 - P2; P3 - P10; P8 - P10; P3 - P11; P8 - P11; P3 - P2; P8 - P2
TC1900008925.hg.1	TC1900008925.hg.1	hsa_circ_0000961	U2AF2	NA	30.778	1.91E-06	5.7193	3.55E-06	ctrl - P10; ctrl - P11; ctrl - P3; ctrl - P8; P11 - P10; P2 - P10; P3 - P10; P2 - P11; P11 - P8; P2 - P3; P2 - P8; P3 - P8
TC0600014194.hg.1	TC0600014194.hg.1	NM_001010927	TIAM2	26230	30.678	1.94E-06	5.7116	3.62E-06	P10 - ctrl; P8 - ctrl; P10 - P11; P10 - P2; P10 - P3; P8 - P11; P8 - P2; P8 - P3
TC0500013328.hg.1	TC0500013328.hg.1	NM_015566	FAM169A	26049	30.646	1.95E-06	5.709	3.63E-06	ctrl - P10; ctrl - P11; ctrl - P2; ctrl - P3; ctrl - P8; P11 - P10; P2 - P10; P3 - P10; P11 - P2; P11 - P8; P3 - P2; P2 - P8; P3 - P8
TC0700010157.hg.1	TC0700010157.hg.1	sunaya.aAug10-unspliced	sunaya	NA	30.566	1.98E-06	5.7029	3.68E-06	ctrl - P10; ctrl - P11; ctrl - P3; ctrl - P8; P2 - P10; P10 - P8; P2 - P11; P2 - P3; P2 - P8; P3 - P8
TC1800008896.hg.1	TC1800008896.hg.1	gleesar.aAug10-unspliced	gleesar	NA	30.566	1.98E-06	5.7029	3.68E-06	ctrl - P10; ctrl - P11; ctrl - P3; ctrl - P8; P2 - P10; P2 - P11; P2 - P3; P2 - P8
TC1600007201.hg.1	TC1600007201.hg.1	NA	NA	NA	30.5	2.01E-06	5.6977	3.72E-06	ctrl - P10; ctrl - P11; ctrl - P3; ctrl - P8; P2 - P10; P2 - P11; P11 - P8; P2 - P3; P2 - P8
TC1600008218.hg.1	TC1600008218.hg.1	NM_001199280	HAS3	3038	30.391	2.05E-06	5.6892	3.79E-06	P10 - ctrl; P3 - ctrl; P8 - ctrl; P10 - P11; P10 - P2; P8 - P10; P11 - P2; P3 - P11; P8 - P11; P3 - P2; P8 - P2
TC1900009890.hg.1	TC1900009890.hg.1	ENST00000587970	LOC105372290	1.05E+08	30.345	2.06E-06	5.6856	3.82E-06	P10 - ctrl; P3 - ctrl; P8 - ctrl; P10 - P11; P10 - P2; P10 - P3; P3 - P11; P8 - P11; P3 - P2; P8 - P2; P8 - P3
TC1000006683.hg.1	TC1000006683.hg.1	NR_038291	LINC00707	1.01E+08	30.339	2.06E-06	5.6852	3.82E-06	ctrl - P2; P3 - ctrl; P8 - ctrl; P10 - P2; P3 - P10; P8 - P10; P11 - P2; P3 - P11; P8 - P11; P3 - P2; P8 - P2; P3 - P8
TC0400006844.hg.1	TC0400006844.hg.1	storsweybu.aAug10-unspliced	storsweybu	NA	30.328	2.07E-06	5.6843	3.83E-06	ctrl - P10; ctrl - P11; ctrl - P8; P11 - P10; P2 - P10; P3 - P10; P2 - P11; P3 - P11; P11 - P8; P2 - P3; P2 - P8; P3 - P8
TC1100007307.hg.1	TC1100007307.hg.1	NM_001276722	C11orf74	119710	30.261	2.09E-06	5.6791	3.87E-06	ctrl - P10; ctrl - P11; ctrl - P2; ctrl - P3; ctrl - P8; P11 - P10; P11 - P2; P11 - P3; P11 - P8; P2 - P3; P2 - P8
TC0200015193.hg.1	TC0200015193.hg.1	NM_001271751	CALCRL	10203	30.216	2.11E-06	5.6755	3.90E-06	ctrl - P10; ctrl - P2; ctrl - P3; ctrl - P8; P11 - P10; P11 - P2; P11 - P3; P11 - P8

TC1700012204.hg.1	TC1700012204.hg.1	ENST00000507171	AC107982.4	NA	30.204	2.12E-06	5.6746	3.90E-06	ctrl - P10; ctrl - P11; ctrl - P3; ctrl - P8; P11 - P10; P2 - P10; P3 - P10; P10 - P8; P11 - P8; P2 - P3; P2 - P8; P3 - P8
TC1500008627.hg.1	TC1500008627.hg.1	NR_027992	NBEAP1	606	30.18	2.12E-06	5.6727	3.92E-06	ctrl - P10; ctrl - P3; ctrl - P8; P11 - P10; P2 - P10; P3 - P10; P11 - P8; P2 - P3; P2 - P8; P3 - P8
TC0200009933.hg.1	TC0200009933.hg.1	NM_001289947	ERICH2	285141	30.149	2.14E-06	5.6703	3.94E-06	ctrl - P10; P11 - ctrl; ctrl - P2; ctrl - P3; ctrl - P8; P11 - P10; P2 - P10; P3 - P10; P11 - P2; P11 - P3; P11 - P8; P3 - P8
TC1100006798.hg.1	TC1100006798.hg.1	NR_073431	TMEM9B-AS1	493900	30.092	2.16E-06	5.6658	3.98E-06	ctrl - P10; ctrl - P2; ctrl - P3; ctrl - P8; P11 - P10; P2 - P10; P3 - P10; P11 - P2; P11 - P3; P11 - P8; P2 - P8; P3 - P8
TC0900008986.hg.1	TC0900008986.hg.1	swolu.aAug10-unspliced	swolu	NA	30.064	2.17E-06	5.6635	3.99E-06	ctrl - P10; ctrl - P11; P2 - ctrl; ctrl - P3; ctrl - P8; P2 - P10; P3 - P10; P2 - P11; P11 - P8; P2 - P3; P2 - P8; P3 - P8
TCUn_GL000218v100006435.hg.1	TCUn_GL000218v100006435.hg.1	hsa_circ_0089767	LOC100233156	NA	30.023	2.19E-06	5.6603	4.02E-06	ctrl - P10; ctrl - P11; P2 - ctrl; ctrl - P3; ctrl - P8; P11 - P10; P2 - P10; P3 - P10; P2 - P11; P11 - P8; P2 - P3; P2 - P8; P3 - P8
TC1400008822.hg.1	TC1400008822.hg.1	NA	NA	NA	30.009	2.19E-06	5.6592	4.03E-06	ctrl - P10; ctrl - P11; ctrl - P3; ctrl - P8; P2 - P10; P3 - P10; P10 - P8; P2 - P11; P11 - P8; P2 - P3; P2 - P8; P3 - P8
TC0100010649.hg.1	TC0100010649.hg.1	smawlybu.aAug10-unspliced	smawlybu	NA	29.992	2.20E-06	5.6579	4.04E-06	ctrl - P10; ctrl - P11; ctrl - P3; ctrl - P8; P2 - P10; P3 - P10; P2 - P11; P11 - P8; P2 - P3; P2 - P8; P3 - P8
TC0200013568.hg.1	TC0200013568.hg.1	hinomo.aAug10-unspliced	hinomo	NA	29.933	2.22E-06	5.6532	4.08E-06	ctrl - P10; ctrl - P11; P2 - ctrl; ctrl - P8; P11 - P10; P2 - P10; P3 - P10; P2 - P11; P11 - P8; P2 - P3; P2 - P8; P3 - P8
TC0700010897.hg.1	TC0700010897.hg.1	yahore.aAug10-unspliced	yahore	NA	29.841	2.26E-06	5.6459	4.14E-06	ctrl - P10; ctrl - P11; ctrl - P3; ctrl - P8; P11 - P10; P2 - P10; P3 - P10; P2 - P11; P11 - P8; P2 - P3; P2 - P8; P3 - P8
TC0500007985.hg.1	TC0500007985.hg.1	blogey.aAug10	blogey		29.827	2.27E-06	5.6448	4.15E-06	ctrl - P10; ctrl - P11; P2 - ctrl; ctrl - P3; ctrl - P8; P11 - P10; P2 - P10; P3 - P10; P2 - P11; P11 - P8; P2 - P3; P2 - P8; P3 - P8
TC0600011125.hg.1	TC0600011125.hg.1	NM_003513	HIST1H2AB	8335	29.811	2.27E-06	5.6435	4.16E-06	P10 - ctrl; ctrl - P11; P8 - ctrl; P10 - P11; P10 - P2; P10 - P3; P2 - P11; P3 - P11; P8 - P11; P8 - P2; P8 - P3
TC1700006649.hg.1	TC1700006649.hg.1	skyvarbu.aAug10-unspliced	skyvarbu	NA	29.777	2.29E-06	5.6408	4.18E-06	ctrl - P10; ctrl - P11; ctrl - P3; ctrl - P8; P2 - P10; P2 - P11; P2 - P3; P2 - P8; P3 - P8
TC0300012196.hg.1	TC0300012196.hg.1	klybeebu.aAug10-unspliced	klybeebu	NA	29.765	2.29E-06	5.6399	4.19E-06	ctrl - P10; ctrl - P3; ctrl - P8; P11 - P10; P2 - P10; P10 - P8; P11 - P3; P11 - P8; P2 - P3; P2 - P8; P3 - P8
TC0400008327.hg.1	TC0400008327.hg.1	koyswoby.aAug10-unspliced	koyswoby	NA	29.74	2.30E-06	5.6378	4.21E-06	ctrl - P10; ctrl - P11; ctrl - P3; ctrl - P8; P11 - P10; P2 - P10; P2 - P11; P11 - P8; P2 - P3; P2 - P8
TC0200014599.hg.1	TC0200014599.hg.1	fuder.aAug10-unspliced	fuder	NA	29.733	2.30E-06	5.6373	4.21E-06	ctrl - P10; ctrl - P11; ctrl - P3; ctrl - P8; P2 - P10; P10 - P8; P2 - P11; P3 - P11; P11 - P8; P2 - P3; P2 - P8; P3 - P8
TC0700012910.hg.1	TC0700012910.hg.1	reeblaw.aAug10-unspliced	reeblaw	NA	29.66	2.34E-06	5.6315	4.26E-06	ctrl - P10; ctrl - P11; P2 - ctrl; ctrl - P3; ctrl - P8; P2 - P10; P10 - P8; P2 - P11; P11 - P8; P2 - P3; P2 - P8; P3 - P8
TC0600009969.hg.1	TC0600009969.hg.1	swyjee.aAug10-unspliced	swyjee	NA	29.551	2.38E-06	5.6228	4.34E-06	ctrl - P10; ctrl - P11; P2 - ctrl; ctrl - P3; ctrl - P8; P2 - P10; P3 - P10; P2 - P11; P11 - P8; P2 - P3; P2 - P8; P3 - P8
TC0100008796.hg.1	TC0100008796.hg.1	NA	NA	NA	29.531	2.39E-06	5.6211	4.36E-06	ctrl - P10; ctrl - P11; P2 - ctrl; ctrl - P8; P11 - P10; P2 - P10; P3 - P10; P2 - P11; P11 - P8; P2 - P3; P2 - P8; P3 - P8
TC0400011223.hg.1	TC0400011223.hg.1	chorgu.aAug10-unspliced	chorgu	NA	29.511	2.40E-06	5.6196	4.37E-06	ctrl - P10; ctrl - P11; P2 - ctrl; ctrl - P3; ctrl - P8; P2 - P10; P10 - P8; P2 - P11; P11 - P8; P2 - P3; P2 - P8; P3 - P8
HTA2-pos-47421981_st	23150844	NA	NA	NA	29.501	2.41E-06	5.6188	4.38E-06	ctrl - P10; ctrl - P11; ctrl - P3; ctrl - P8; P11 - P10; P2 - P10; P3 - P10; P2 - P11; P11 - P8; P2 - P3; P2 - P8; P3 - P8
TC0100013227.hg.1	TC0100013227.hg.1	farworbo.aAug10-unspliced	farworbo	NA	29.464	2.42E-06	5.6158	4.40E-06	ctrl - P10; ctrl - P11; ctrl - P3; ctrl - P8; P2 - P10; P3 - P10; P2 - P11; P3 - P11; P2 - P3; P2 - P8; P3 - P8
TC1100010743.hg.1	TC1100010743.hg.1	peechee.aAug10-unspliced	peechee	NA	29.447	2.43E-06	5.6144	4.41E-06	ctrl - P10; ctrl - P11; ctrl - P3; ctrl - P8; P2 - P10; P2 - P11; P2 - P3; P2 - P8
TC1600006659.hg.1	TC1600006659.hg.1	nazobu.aAug10-unspliced	nazobu	NA	29.415	2.44E-06	5.6118	4.44E-06	P10 - ctrl; P3 - ctrl; P8 - ctrl; P10 - P11; P10 - P2; P8 - P10; P3 - P11; P8 - P11; P3 - P2; P8 - P2; P8 - P3
TC0400012992.hg.1	TC0400012992.hg.1	NM_001009554	MFAP3L	9848	29.391	2.46E-06	5.6099	4.45E-06	ctrl - P10; ctrl - P2; ctrl - P3; ctrl - P8; P11 - P10; P3 - P10; P11 - P2; P11 - P3; P11 - P8; P3 - P2; P3 - P8
TC0300009217.hg.1	TC0300009217.hg.1	ENST00000364347	Y_RNA		29.345	2.48E-06	5.6062	4.49E-06	ctrl - P10; ctrl - P11; ctrl - P3; ctrl - P8; P11 - P10; P2 - P10; P3 - P10; P2 - P11; P11 - P8; P2 - P3; P2 - P8; P3 - P8
TC1500006606.hg.1	TC1500006606.hg.1	NR_003353	SNORD115-38	15q11.2	29.337	2.48E-06	5.6056	4.49E-06	P10 - ctrl; P3 - ctrl; P8 - ctrl; P10 - P11; P10 - P2; P3 - P10; P3 - P11; P8 - P11; P3 - P2; P8 - P2; P3 - P8
TC0200011702.hg.1	TC0200011702.hg.1	ENST00000607181.1	RP11-254F7.3		29.332	2.48E-06	5.6051	4.49E-06	ctrl - P10; ctrl - P11; ctrl - P3; ctrl - P8; P11 - P10; P2 - P10; P11 - P3; P11 - P8; P2 - P3; P2 - P8
TC1700008911.hg.1	TC1700008911.hg.1	NA	NA	NA	29.303	2.50E-06	5.6028	4.51E-06	ctrl - P10; ctrl - P11; ctrl - P2; ctrl - P3; ctrl - P8; P11 - P10; P2 - P10; P11 - P3; P11 - P8; P2 - P3; P2 - P8; P3 - P8
TC1500008523.hg.1	TC1500008523.hg.1	darzee.aAug10-unspliced	darzee	NA	29.301	2.50E-06	5.6026	4.51E-06	ctrl - P10; ctrl - P11; ctrl - P3; ctrl - P8; P2 - P10; P3 - P10; P2 - P11; P2 - P3; P2 - P8; P3 - P8
TC0X00007078.hg.1	TC0X00007078.hg.1	flujor.aAug10-unspliced	flujor	NA	29.294	2.50E-06	5.6021	4.51E-06	ctrl - P10; ctrl - P11; ctrl - P3; ctrl - P8; P11 - P10; P2 - P10; P3 - P10; P2 - P11; P11 - P8; P2 - P3; P2 - P8; P3 - P8
TC0700009089.hg.1	TC0700009089.hg.1	NM_001134336	STRIP2	57464	29.224	2.53E-06	5.5964	4.57E-06	P10 - ctrl; P3 - ctrl; P8 - ctrl; P10 - P11; P10 - P2; P3 - P11; P8 - P11; P3 - P2; P8 - P2
TC0600008504.hg.1	TC0600008504.hg.1	NA	NA	NA	29.215	2.54E-06	5.5957	4.57E-06	ctrl - P10; ctrl - P11; ctrl - P8; P10 - P11; P2 - P10; P3 - P10; P2 - P11; P3 - P11; P8 - P11; P2 - P8; P3 - P8

TC0500010461.hg.1	TC0500010461.hg.1	NA	NA	NA	29.175	2.56E-06	5.5925	4.60E-06	ctrl - P10; ctrl - P11; P2 - ctrl; ctrl - P3; ctrl - P8; P2 - P10; P10 - P8; P2 - P11; P11 - P8; P2 - P3; P2 - P8; P3 - P8
TC1600006942.hg.1	TC1600006942.hg.1	boysmor.aAug10-unspliced	boysmor	NA	29.173	2.56E-06	5.5923	4.60E-06	ctrl - P10; ctrl - P11; ctrl - P3; ctrl - P8; P11 - P10; P2 - P10; P2 - P11; P11 - P3; P11 - P8; P2 - P3; P2 - P8
TC1400007235.hg.1	TC1400007235.hg.1	kluslor.aAug10-unspliced	kluslor	NA	29.162	2.56E-06	5.5914	4.61E-06	ctrl - P10; ctrl - P11; P2 - ctrl; ctrl - P3; ctrl - P8; P2 - P10; P10 - P8; P2 - P11; P11 - P8; P2 - P3; P2 - P8; P3 - P8
TC0400010002.hg.1	TC0400010002.hg.1	NA	NA	NA	29.154	2.57E-06	5.5907	4.61E-06	ctrl - P10; ctrl - P11; ctrl - P2; ctrl - P3; ctrl - P8; P11 - P10; P2 - P10; P3 - P10; P11 - P3; P11 - P8; P2 - P3; P2 - P8; P3 - P8
TC0100009143.hg.1	TC0100009143.hg.1	swuplo.aAug10-unspliced	swuplo	NA	29.14	2.57E-06	5.5896	4.62E-06	ctrl - P10; ctrl - P11; P2 - ctrl; ctrl - P3; ctrl - P8; P2 - P10; P3 - P10; P2 - P11; P2 - P3; P2 - P8; P3 - P8
TC0200009619.hg.1	TC0200009619.hg.1	NM_177964	LYPD6B	2q23.2	29.128	2.58E-06	5.5886	4.63E-06	ctrl - P10; ctrl - P2; ctrl - P3; ctrl - P8; P11 - P10; P11 - P2; P11 - P3; P11 - P8; P2 - P3
TC2000009137.hg.1	TC2000009137.hg.1	votor.aAug10-unspliced	votor	NA	29.104	2.59E-06	5.5867	4.65E-06	ctrl - P10; ctrl - P11; ctrl - P3; ctrl - P8; P11 - P10; P2 - P10; P3 - P10; P2 - P11; P11 - P8; P2 - P3; P2 - P8; P3 - P8
TC1000008093.hg.1	TC1000008093.hg.1	snamu.aAug10-unspliced	snamu	NA	29.091	2.60E-06	5.5856	4.65E-06	ctrl - P10; ctrl - P11; ctrl - P3; ctrl - P8; P2 - P10; P3 - P10; P2 - P11; P3 - P11; P2 - P3; P2 - P8; P3 - P8
TC0200010417.hg.1	TC0200010417.hg.1	smormybo.aAug10-unspliced	smormybo	NA	29.09	2.60E-06	5.5855	4.65E-06	ctrl - P10; ctrl - P11; ctrl - P3; ctrl - P8; P11 - P10; P2 - P10; P3 - P10; P10 - P8; P2 - P11; P11 - P8; P2 - P8; P3 - P8
TC1200010647.hg.1	TC1200010647.hg.1	NR_110589	LOC101927318	1.02E+08	28.958	2.66E-06	5.5748	4.77E-06	P10 - ctrl; P3 - ctrl; P8 - ctrl; P10 - P11; P10 - P2; P3 - P11; P8 - P11; P3 - P2; P8 - P2; P8 - P3
TC0500011194.hg.1	TC0500011194.hg.1	NM_001256566	F2RL2	2151	28.843	2.72E-06	5.5654	4.86E-06	ctrl - P10; ctrl - P2; ctrl - P8; P11 - P10; P3 - P10; P11 - P2; P11 - P8; P3 - P2; P3 - P8
TC0200007155.hg.1	TC0200007155.hg.1	NM_014600	EHD3	30845	28.842	2.72E-06	5.5653	4.86E-06	P10 - ctrl; ctrl - P2; P3 - ctrl; P8 - ctrl; P10 - P11; P10 - P2; P3 - P10; P3 - P11; P8 - P11; P3 - P2; P8 - P2
TC0200015609.hg.1	TC0200015609.hg.1	tawgobu.aAug10-unspliced	tawgobu	NA	28.84	2.72E-06	5.5652	4.86E-06	ctrl - P10; ctrl - P11; ctrl - P3; ctrl - P8; P11 - P10; P2 - P10; P2 - P11; P11 - P8; P2 - P3; P2 - P8; P3 - P8
TC0200012744.hg.1	TC0200012744.hg.1	blarklee.aAug10-unspliced	blarklee	NA	28.798	2.74E-06	5.5617	4.90E-06	ctrl - P10; ctrl - P11; ctrl - P3; ctrl - P8; P2 - P10; P10 - P8; P2 - P11; P11 - P8; P2 - P3; P2 - P8; P3 - P8
TC0200012919.hg.1	TC0200012919.hg.1	ENST00000419809.1	AC010987.5		28.764	2.76E-06	5.5589	4.93E-06	ctrl - P10; ctrl - P2; ctrl - P8; P11 - P10; P2 - P10; P3 - P10; P11 - P2; P11 - P8; P2 - P8; P3 - P8
TC0X00010382.hg.1	TC0X00010382.hg.1	NM_001012978	BEX5	340542	28.744	2.77E-06	5.5573	4.94E-06	ctrl - P10; P11 - ctrl; P2 - ctrl; ctrl - P8; P11 - P10; P2 - P10; P11 - P3; P11 - P8; P2 - P3; P2 - P8; P3 - P8
TC0500011020.hg.1	TC0500011020.hg.1	blystarby.aAug10-unspliced	blystarby	NA	28.727	2.78E-06	5.5559	4.95E-06	ctrl - P10; ctrl - P11; P2 - ctrl; ctrl - P8; P11 - P10; P2 - P10; P3 - P10; P2 - P11; P11 - P8; P2 - P3; P2 - P8; P3 - P8
TC0500011043.hg.1	TC0500011043.hg.1	lawstarby.aAug10-unspliced	lawstarby	NA	28.727	2.78E-06	5.5559	4.95E-06	ctrl - P10; ctrl - P11; P2 - ctrl; ctrl - P8; P11 - P10; P2 - P10; P3 - P10; P2 - P11; P11 - P8; P2 - P3; P2 - P8; P3 - P8
TC1300008090.hg.1	TC1300008090.hg.1	stysto.aAug10-unspliced	stysto	NA	28.721	2.78E-06	5.5554	4.95E-06	ctrl - P10; ctrl - P11; ctrl - P3; ctrl - P8; P2 - P10; P10 - P8; P2 - P11; P11 - P8; P2 - P3; P2 - P8; P3 - P8
TC1000007148.hg.1	TC1000007148.hg.1	NA	NA	NA	28.699	2.80E-06	5.5536	4.97E-06	ctrl - P10; ctrl - P11; P2 - ctrl; ctrl - P3; ctrl - P8; P2 - P10; P3 - P10; P2 - P11; P2 - P3; P2 - P8; P3 - P8
TC1700009938.hg.1	TC1700009938.hg.1	NM_001199989	RASD1	51655	28.673	2.81E-06	5.5514	4.99E-06	P10 - ctrl; P8 - ctrl; P10 - P11; P10 - P2; P10 - P3; P3 - P11; P8 - P11; P8 - P2; P8 - P3
TC1600008428.hg.1	TC1600008428.hg.1	churaw.aAug10-unspliced	churaw	NA	28.631	2.83E-06	5.548	5.03E-06	ctrl - P10; ctrl - P11; P2 - ctrl; ctrl - P8; P2 - P10; P10 - P8; P2 - P11; P3 - P11; P11 - P8; P2 - P3; P2 - P8; P3 - P8
TC0X00010559.hg.1	TC0X00010559.hg.1	NM_001243963	LRCH2	57631	28.621	2.84E-06	5.5471	5.03E-06	ctrl - P10; ctrl - P2; ctrl - P3; ctrl - P8; P11 - P10; P2 - P10; P3 - P10; P11 - P2; P11 - P3; P11 - P8; P3 - P8
TC1200010408.hg.1	TC1200010408.hg.1	snufo.aAug10-unspliced	snufo	NA	28.577	2.86E-06	5.5435	5.07E-06	ctrl - P10; ctrl - P11; ctrl - P3; ctrl - P8; P2 - P10; P2 - P11; P11 - P8; P2 - P3; P2 - P8; P3 - P8
TC0700013531.hg.1	TC0700013531.hg.1	NM_024014	HOXA6	3203	28.558	2.87E-06	5.5419	5.09E-06	ctrl - P10; ctrl - P2; ctrl - P3; ctrl - P8; P11 - P10; P2 - P10; P11 - P2; P11 - P3; P11 - P8; P2 - P3
HTA2-pos-47421984_st	23150847	NA	NA	NA	28.542	2.88E-06	5.5406	5.10E-06	ctrl - P10; ctrl - P11; ctrl - P3; ctrl - P8; P2 - P10; P3 - P10; P10 - P8; P2 - P11; P11 - P8; P2 - P8; P3 - P8
TC0700008102.hg.1	TC0700008102.hg.1	lerblaby.aAug10-unspliced	lerblaby	NA	28.539	2.88E-06	5.5403	5.10E-06	ctrl - P10; ctrl - P11; ctrl - P3; ctrl - P8; P2 - P10; P2 - P11; P11 - P8; P2 - P3; P2 - P8; P3 - P8
TC0500008811.hg.1	TC0500008811.hg.1	sporspybu.aAug10-unspliced	sporspybu	NA	28.506	2.90E-06	5.5376	5.12E-06	ctrl - P10; ctrl - P11; ctrl - P3; ctrl - P8; P2 - P10; P10 - P8; P2 - P11; P11 - P8; P2 - P3; P2 - P8; P3 - P8
TC1700007174.hg.1	TC1700007174.hg.1	NA	NA	NA	28.504	2.90E-06	5.5374	5.12E-06	P10 - ctrl; P11 - ctrl; P3 - ctrl; P8 - ctrl; P10 - P2; P10 - P3; P10 - P8; P11 - P2; P11 - P3; P11 - P8; P3 - P2; P8 - P2
TC1200007029.hg.1	TC1200007029.hg.1	ENST00000535764.1	RP11-405A12.2		28.421	2.95E-06	5.5305	5.20E-06	ctrl - P10; ctrl - P11; ctrl - P3; ctrl - P8; P11 - P10; P2 - P10; P3 - P10; P2 - P11; P11 - P8; P2 - P3; P2 - P8; P3 - P8
TC1100010948.hg.1	TC1100010948.hg.1	NM_005838	GLYAT	10249	28.391	2.96E-06	5.5281	5.23E-06	ctrl - P10; P2 - ctrl; P3 - ctrl; P11 - P10; P2 - P10; P3 - P10; P2 - P11; P3 - P11; P11 - P8; P2 - P8; P3 - P8
TC0100008645.hg.1	TC0100008645.hg.1	skernubu.cAug10-unspliced	skernubu	NA	28.386	2.97E-06	5.5277	5.23E-06	ctrl - P10; ctrl - P11; P2 - ctrl; ctrl - P3; ctrl - P8; P2 - P10; P3 - P10; P2 - P11; P3 - P11; P2 - P3; P2 - P8; P3 - P8
TC0200008536.hg.1	TC0200008536.hg.1	ANKRD36.veAug10-unspliced	ANKRD36	NA	28.377	2.97E-06	5.5269	5.23E-06	ctrl - P10; ctrl - P11; ctrl - P8; P11 - P10; P2 - P10; P3 - P10; P8 - P10; P2 - P11; P11 - P8; P2 - P3; P2 - P8; P3 - P8

TC0200009684.hg.1	TC0200009684.hg.1	flawdawbu.aAug10-unspliced	flawdawbu	NA	28.356	2.98E-06	5.5251	5.25E-06	ctrl - P10; ctrl - P11; P2 - ctrl; ctrl - P8; P2 - P10; P3 - P10; P10 - P8; P2 - P11; P3 - P11; P11 - P8; P2 - P3; P2 - P8; P3 - P8
TC1500006666.hg.1	TC1500006666.hg.1	NR_033351	GOLGA8F	1E+08	28.345	2.99E-06	5.5243	5.26E-06	ctrl - P10; ctrl - P11; ctrl - P3; ctrl - P8; P11 - P10; P2 - P10; P2 - P11; P11 - P8; P2 - P3; P2 - P8
TC0200011885.hg.1	TC0200011885.hg.1	NM_001008237	TTC32	130502	28.322	3.00E-06	5.5224	5.28E-06	ctrl - P10; ctrl - P2; ctrl - P3; ctrl - P8; P11 - P10; P2 - P10; P3 - P10; P11 - P2; P11 - P3; P11 - P8; P2 - P8; P3 - P8
TC0400011994.hg.1	TC0400011994.hg.1	NM_001101669	INPP4B	8821	28.303	3.01E-06	5.5208	5.29E-06	P10 - ctrl; P2 - ctrl; P3 - ctrl; P8 - ctrl; P10 - P11; P10 - P2; P10 - P3; P2 - P11; P3 - P11; P8 - P11; P8 - P3
TC0600013131.hg.1	TC0600013131.hg.1	blerklyby.aAug10-unspliced	blerklyby	NA	28.23	3.06E-06	5.5147	5.37E-06	ctrl - P10; ctrl - P11; P2 - ctrl; ctrl - P3; ctrl - P8; P2 - P10; P3 - P10; P2 - P11; P3 - P11; P11 - P8; P2 - P3; P2 - P8; P3 - P8
TC0600013119.hg.1	TC0600013119.hg.1	NM_001012279	SOGA3	387104	28.19	3.08E-06	5.5113	5.40E-06	ctrl - P10; ctrl - P2; P3 - ctrl; ctrl - P8; P11 - P10; P3 - P10; P11 - P2; P3 - P11; P11 - P8; P3 - P2; P3 - P8
TC1000007705.hg.1	TC1000007705.hg.1	zoygleybu.aAug10-unspliced	zoygleybu	NA	28.185	3.08E-06	5.5109	5.40E-06	ctrl - P10; ctrl - P11; ctrl - P8; P2 - P10; P3 - P10; P2 - P11; P3 - P11; P2 - P3; P2 - P8; P3 - P8
TC0900007729.hg.1	TC0900007729.hg.1	uc064tyu.1	RNU4-29P	NA	28.163	3.10E-06	5.5091	5.42E-06	ctrl - P10; ctrl - P11; ctrl - P3; ctrl - P8; P2 - P10; P3 - P10; P2 - P11; P3 - P11; P11 - P8; P2 - P3; P2 - P8; P3 - P8
TC0100017449.hg.1	TC0100017449.hg.1	NM_001143962	CAPN8	388743	28.152	3.10E-06	5.5081	5.43E-06	ctrl - P10; ctrl - P11; ctrl - P3; ctrl - P8; P11 - P10; P2 - P10; P2 - P11; P11 - P8; P2 - P3; P2 - P8
TC0600008093.hg.1	TC0600008093.hg.1	NA	NA	NA	28.106	3.13E-06	5.5043	5.48E-06	ctrl - P10; ctrl - P11; ctrl - P3; ctrl - P8; P11 - P10; P2 - P10; P3 - P10; P2 - P11; P11 - P8; P2 - P3; P2 - P8; P3 - P8
TC0600013885.hg.1	TC0600013885.hg.1	sneejo.aAug10-unspliced	sneejo	NA	28.092	3.14E-06	5.5031	5.48E-06	ctrl - P10; ctrl - P11; ctrl - P2; ctrl - P3; ctrl - P8; P11 - P10; P2 - P10; P11 - P3; P11 - P8; P2 - P3; P2 - P8; P3 - P8
TC0100015783.hg.1	TC0100015783.hg.1	NM_000449	RFX5	5993	28.09	3.14E-06	5.503	5.48E-06	P10 - ctrl; ctrl - P11; ctrl - P2; P8 - ctrl; P10 - P11; P10 - P2; P10 - P3; P11 - P2; P3 - P11; P8 - P11; P3 - P2; P8 - P2; P8 - P3
TC1800006591.hg.1	TC1800006591.hg.1	peykler.aAug10-unspliced	peykler	NA	28.09	3.14E-06	5.5029	5.48E-06	ctrl - P10; ctrl - P11; P2 - ctrl; ctrl - P8; P2 - P10; P3 - P10; P2 - P11; P3 - P11; P2 - P3; P2 - P8; P3 - P8
TC0700011984.hg.1	TC0700011984.hg.1	uc064gcw.1	AC005071.3	NA	28.023	3.18E-06	5.4973	5.55E-06	ctrl - P10; ctrl - P11; ctrl - P3; ctrl - P8; P2 - P10; P3 - P10; P2 - P11; P2 - P3; P2 - P8; P3 - P8
TC0400007270.hg.1	TC0400007270.hg.1	NM_138389	FAM114A1	92689	28.019	3.18E-06	5.4969	5.55E-06	P3 - ctrl; P8 - ctrl; P10 - P11; P10 - P2; P3 - P10; P8 - P10; P3 - P11; P8 - P11; P3 - P2; P8 - P2; P3 - P8
TC0500009414.hg.1	TC0500009414.hg.1	NA	NA	NA	28.018	3.19E-06	5.4969	5.55E-06	ctrl - P10; ctrl - P11; P2 - ctrl; ctrl - P3; ctrl - P8; P2 - P10; P2 - P11; P2 - P3; P2 - P8
TC1400007089.hg.1	TC1400007089.hg.1	NA	NA	NA	27.977	3.21E-06	5.4934	5.59E-06	ctrl - P10; ctrl - P11; ctrl - P3; ctrl - P8; P11 - P10; P2 - P10; P3 - P10; P2 - P11; P11 - P8; P2 - P3; P2 - P8; P3 - P8
TC1200012299.hg.1	TC1200012299.hg.1	ENST00000602558.1	RP11-214K3.18		27.96	3.22E-06	5.492	5.60E-06	ctrl - P10; ctrl - P11; ctrl - P3; ctrl - P8; P11 - P10; P2 - P10; P10 - P8; P2 - P11; P11 - P8; P2 - P3; P2 - P8; P3 - P8
TC0400007803.hg.1	TC0400007803.hg.1	nokime.aAug10-unspliced	nokime	NA	27.934	3.24E-06	5.4898	5.63E-06	ctrl - P10; ctrl - P3; ctrl - P8; P11 - P10; P2 - P10; P11 - P3; P11 - P8; P2 - P3; P2 - P8
TC1100012426.hg.1	TC1100012426.hg.1	ENST00000454832.1	SIK3-IT1		27.921	3.25E-06	5.4887	5.63E-06	ctrl - P10; ctrl - P11; ctrl - P3; ctrl - P8; P11 - P10; P2 - P10; P3 - P10; P2 - P11; P11 - P8; P2 - P3; P2 - P8; P3 - P8
TC0800012463.hg.1	TC0800012463.hg.1	NR_108049	CCAT1	1.01E+08	27.917	3.25E-06	5.4884	5.63E-06	P10 - ctrl; P3 - ctrl; P8 - ctrl; P10 - P11; P10 - P2; P8 - P10; P3 - P11; P8 - P11; P3 - P2; P8 - P2
TC0800011679.hg.1	TC0800011679.hg.1	NM_014109	ATAD2	29028	27.914	3.25E-06	5.4881	5.63E-06	P10 - ctrl; ctrl - P11; P3 - ctrl; P8 - ctrl; P10 - P11; P10 - P2; P8 - P10; P3 - P11; P8 - P11; P3 - P2; P8 - P2; P8 - P3
TC1600007897.hg.1	TC1600007897.hg.1	chaworbu.aAug10-unspliced	chaworbu	NA	27.913	3.25E-06	5.488	5.63E-06	ctrl - P10; ctrl - P3; ctrl - P8; P11 - P10; P2 - P10; P3 - P10; P2 - P11; P11 - P8; P2 - P3; P2 - P8; P3 - P8
TC0900009427.hg.1	TC0900009427.hg.1	NA	NA	NA	27.891	3.26E-06	5.4862	5.65E-06	ctrl - P10; ctrl - P11; P2 - ctrl; ctrl - P3; ctrl - P8; P2 - P10; P2 - P11; P11 - P8; P2 - P3; P2 - P8; P3 - P8
TC0600011231.hg.1	TC0600011231.hg.1	uc063mkg.1	Z98744.2	NA	27.845	3.29E-06	5.4823	5.70E-06	P10 - ctrl; P11 - ctrl; P8 - ctrl; P10 - P11; P10 - P2; P10 - P3; P11 - P2; P11 - P3; P8 - P11; P8 - P2; P8 - P3
TC0700010992.hg.1	TC0700010992.hg.1	forblaw.aAug10-unspliced	forblaw		27.842	3.30E-06	5.482	5.70E-06	ctrl - P10; ctrl - P11; ctrl - P3; ctrl - P8; P11 - P10; P2 - P10; P3 - P10; P2 - P11; P11 - P8; P2 - P3; P2 - P8; P3 - P8
TC0200006527.hg.1	TC0200006527.hg.1	NA	NA	NA	27.836	3.30E-06	5.4815	5.70E-06	ctrl - P10; ctrl - P11; ctrl - P2; ctrl - P8; P11 - P10; P2 - P10; P3 - P10; P11 - P8; P2 - P8; P3 - P8
TC0500012806.hg.1	TC0500012806.hg.1	goyshaby.aAug10-unspliced	goyshaby	NA	27.798	3.32E-06	5.4783	5.74E-06	P10 - ctrl; P11 - ctrl; P3 - ctrl; P8 - ctrl; P10 - P11; P10 - P2; P11 - P2; P3 - P11; P8 - P11; P3 - P2; P8 - P2
TC0500007467.hg.1	TC0500007467.hg.1	floystobu.aAug10-unspliced	floystobu	NA	27.786	3.33E-06	5.4773	5.75E-06	ctrl - P10; ctrl - P11; ctrl - P3; ctrl - P8; P11 - P10; P2 - P10; P2 - P11; P11 - P8; P2 - P3; P2 - P8; P3 - P8
TC0700013514.hg.1	TC0700013514.hg.1	NA	NA	NA	27.759	3.35E-06	5.475	5.77E-06	ctrl - P10; ctrl - P11; ctrl - P2; ctrl - P3; ctrl - P8; P11 - P10; P2 - P10; P3 - P10; P11 - P8; P2 - P8; P3 - P8
TC1100012963.hg.1	TC1100012963.hg.1	NR_034137	ZBED5-AS1	729013	27.758	3.35E-06	5.4749	5.77E-06	ctrl - P10; P11 - ctrl; ctrl - P2; ctrl - P3; ctrl - P8; P11 - P10; P10 - P3; P11 - P2; P11 - P3; P11 - P8; P2 - P3; P8 - P3
TC0300008941.hg.1	TC0300008941.hg.1	nekoyu.aAug10-unspliced	nekoyu	NA	27.728	3.37E-06	5.4724	5.80E-06	ctrl - P10; ctrl - P11; ctrl - P3; ctrl - P8; P2 - P10; P3 - P10; P2 - P11; P11 - P8; P2 - P3; P2 - P8; P3 - P8
TC0100014857.hg.1	TC0100014857.hg.1	NM_001134486	GBP5	115362	27.678	3.40E-06	5.4681	5.86E-06	P10 - ctrl; P3 - ctrl; P8 - ctrl; P10 - P11; P10 - P2; P8 - P10; P3 - P11; P8 - P11; P3 - P2; P8 - P2
TC0800008833.hg.1	TC0800008833.hg.1	NR_109833	PRNCR1	1.02E+08	27.658	3.42E-06	5.4664	5.88E-06	ctrl - P10; P2 - ctrl; ctrl - P8; P11 - P10; P2 - P10; P3 - P10; P2 - P11; P3 - P11; P11 - P8; P2 - P3; P2 - P8; P3 - P8

TC1000010777.hg.1	TC1000010777.hg.1	versha.aAug10-unspliced	versha	NA	27.635	3.43E-06	5.4645	5.90E-06	ctrl - P10; ctrl - P11; P2 - ctrl; ctrl - P3; ctrl - P8; P2 - P10; P10 - P8; P2 - P11; P3 - P11; P11 - P8; P2 - P3; P2 - P8; P3 - P8
TC0700011774.hg.1	TC0700011774.hg.1	mawmer.aAug10-unspliced	mawmer	NA	27.582	3.47E-06	5.46	5.95E-06	ctrl - P10; ctrl - P11; ctrl - P3; ctrl - P8; P11 - P10; P2 - P10; P3 - P10; P2 - P11; P11 - P8; P2 - P3; P2 - P8; P3 - P8
TC0400010243.hg.1	TC0400010243.hg.1	jarsmerby.aAug10-unspliced	jarsmerby	NA	27.582	3.47E-06	5.46	5.95E-06	ctrl - P10; ctrl - P11; ctrl - P3; ctrl - P8; P11 - P10; P2 - P10; P2 - P11; P11 - P3; P11 - P8; P2 - P3; P2 - P8
TC0100013293.hg.1	TC0100013293.hg.1	NM_002167	ID3	3399	27.5	3.52E-06	5.453	6.04E-06	ctrl - P10; P11 - ctrl; ctrl - P2; ctrl - P3; ctrl - P8; P11 - P10; P11 - P2; P11 - P3; P11 - P8
TC0X00008514.hg.1	TC0X00008514.hg.1	NM_182540	DDX26B	His) box polypeptide 26B	27.487	3.53E-06	5.4518	6.06E-06	ctrl - P10; ctrl - P11; ctrl - P2; ctrl - P3; ctrl - P8; P11 - P10; P2 - P10; P3 - P10; P11 - P3; P11 - P8; P2 - P8; P3 - P8
TC1200007613.hg.1	TC1200007613.hg.1	jarchar.aAug10-unspliced	jarchar	NA	27.462	3.55E-06	5.4497	6.08E-06	ctrl - P10; ctrl - P11; ctrl - P3; ctrl - P8; P11 - P10; P2 - P10; P2 - P11; P11 - P3; P11 - P8; P2 - P3; P2 - P8
TC1100010108.hg.1	TC1100010108.hg.1	stoyglor.aAug10-unspliced	stoyglor	NA	27.423	3.58E-06	5.4464	6.12E-06	ctrl - P10; ctrl - P11; ctrl - P3; ctrl - P8; P11 - P10; P2 - P10; P3 - P10; P11 - P3; P11 - P8; P2 - P3; P2 - P8; P3 - P8
TC0900011715.hg.1	TC0900011715.hg.1	bertybu.aAug10-unspliced	bertybu	NA	27.407	3.59E-06	5.445	6.14E-06	ctrl - P10; ctrl - P11; ctrl - P3; ctrl - P8; P2 - P10; P10 - P3; P10 - P8; P2 - P11; P11 - P3; P11 - P8; P2 - P3; P2 - P8
TC0400010787.hg.1	TC0400010787.hg.1	shyboybo.aAug10-unspliced	shyboybo	NA	27.393	3.60E-06	5.4438	6.15E-06	ctrl - P10; ctrl - P11; ctrl - P8; P2 - P10; P3 - P10; P2 - P11; P3 - P11; P2 - P3; P2 - P8; P3 - P8
TC1300006717.hg.1	TC1300006717.hg.1	sysheebu.aAug10-unspliced	sysheebu	NA	27.356	3.63E-06	5.4406	6.19E-06	ctrl - P10; ctrl - P11; ctrl - P3; ctrl - P8; P11 - P10; P2 - P10; P10 - P8; P2 - P11; P11 - P8; P2 - P3; P2 - P8; P3 - P8
TC1500008526.hg.1	TC1500008526.hg.1	soharu.aAug10-unspliced	soharu	NA	27.344	3.63E-06	5.4396	6.20E-06	ctrl - P10; ctrl - P11; ctrl - P3; ctrl - P8; P11 - P10; P2 - P10; P3 - P10; P2 - P11; P11 - P8; P2 - P3; P2 - P8; P3 - P8
TC0800011956.hg.1	TC0800011956.hg.1	ENST00000612268	MIR7641-1	1.02E+08	27.336	3.64E-06	5.4389	6.21E-06	P11 - ctrl; P2 - ctrl; P3 - ctrl; P11 - P10; P2 - P10; P3 - P10; P2 - P11; P3 - P11; P2 - P3; P2 - P8; P3 - P8
TC1600008887.hg.1	TC1600008887.hg.1	OTTHUMT00000474478	RP11-104N10.2		27.282	3.68E-06	5.4343	6.27E-06	ctrl - P10; ctrl - P11; ctrl - P3; ctrl - P8; P2 - P10; P2 - P11; P11 - P8; P2 - P3; P2 - P8; P3 - P8
TC0600011601.hg.1	TC0600011601.hg.1	sneeglyby.aAug10-unspliced	sneeglyby	NA	27.279	3.68E-06	5.434	6.27E-06	ctrl - P10; ctrl - P11; P2 - ctrl; ctrl - P3; ctrl - P8; P2 - P10; P3 - P10; P2 - P11; P11 - P8; P2 - P3; P2 - P8; P3 - P8
TC1700009890.hg.1	TC1700009890.hg.1	ENST00000583934.1	RP11-138I1.4		27.277	3.68E-06	5.4338	6.27E-06	P10 - ctrl; P11 - ctrl; P3 - ctrl; P8 - ctrl; P10 - P11; P10 - P2; P10 - P3; P10 - P8; P11 - P2; P3 - P2; P8 - P2
TC0400012828.hg.1	TC0400012828.hg.1	NM_001083907	BANK1	55024	27.262	3.69E-06	5.4325	6.28E-06	P10 - ctrl; P3 - ctrl; P8 - ctrl; P10 - P11; P10 - P2; P3 - P11; P8 - P11; P3 - P2; P8 - P2; P8 - P3
TC0100009271.hg.1	TC0100009271.hg.1	yoreha.aAug10-unspliced	yoreha	NA	27.229	3.72E-06	5.4296	6.32E-06	ctrl - P10; ctrl - P11; ctrl - P3; ctrl - P8; P11 - P10; P2 - P10; P2 - P11; P11 - P8; P2 - P3; P2 - P8
HTA2-pos-47421979_st	23150843	NA	NA	NA	27.226	3.72E-06	5.4294	6.32E-06	ctrl - P10; ctrl - P11; ctrl - P3; ctrl - P8; P11 - P10; P2 - P10; P3 - P10; P2 - P11; P11 - P8; P2 - P3; P2 - P8; P3 - P8
TC0600012866.hg.1	TC0600012866.hg.1	weesporby.aAug10-unspliced	weesporby	NA	27.222	3.72E-06	5.4291	6.32E-06	ctrl - P10; ctrl - P11; ctrl - P3; ctrl - P8; P2 - P10; P3 - P10; P2 - P11; P3 - P11; P2 - P3; P2 - P8; P3 - P8
TC0100017540.hg.1	TC0100017540.hg.1	shorobo.aAug10-unspliced	shorobo	NA	27.222	3.72E-06	5.429	6.32E-06	ctrl - P10; ctrl - P11; ctrl - P2; ctrl - P3; ctrl - P8; P11 - P10; P2 - P10; P11 - P3; P11 - P8; P2 - P3; P2 - P8
TC0900010088.hg.1	TC0900010088.hg.1	dawterbo.aAug10-unspliced	dawterbo		27.209	3.73E-06	5.428	6.33E-06	ctrl - P10; ctrl - P11; ctrl - P3; ctrl - P8; P11 - P10; P2 - P10; P3 - P10; P2 - P11; P11 - P8; P2 - P3; P2 - P8; P3 - P8
TC0200009072.hg.1	TC0200009072.hg.1	NA	NA	NA	27.204	3.74E-06	5.4275	6.33E-06	ctrl - P10; ctrl - P3; ctrl - P8; P11 - P10; P2 - P10; P10 - P8; P2 - P11; P11 - P3; P11 - P8; P2 - P3; P2 - P8
TC0600010935.hg.1	TC0600010935.hg.1	rurome.aAug10-unspliced	rurome	NA	27.159	3.77E-06	5.4236	6.38E-06	ctrl - P11; P2 - ctrl; ctrl - P8; P2 - P10; P3 - P10; P10 - P8; P2 - P11; P3 - P11; P11 - P8; P2 - P3; P2 - P8; P3 - P8
TC0700010406.hg.1	TC0700010406.hg.1	NA	NA	NA	27.15	3.78E-06	5.4228	6.39E-06	P10 - ctrl; P11 - ctrl; P3 - ctrl; P8 - ctrl; P10 - P11; P10 - P2; P10 - P3; P11 - P2; P8 - P11; P3 - P2; P8 - P2; P8 - P3
TC2100006826.hg.1	TC2100006826.hg.1	NR_024357	LINC00113	54088	27.135	3.79E-06	5.4216	6.41E-06	ctrl - P10; P11 - ctrl; ctrl - P3; ctrl - P8; P11 - P10; P2 - P10; P11 - P2; P11 - P3; P11 - P8; P2 - P3; P2 - P8
TC0900011343.hg.1	TC0900011343.hg.1	NA	NA	NA	27.119	3.80E-06	5.4201	6.42E-06	ctrl - P10; ctrl - P11; ctrl - P8; P2 - P10; P3 - P10; P2 - P11; P3 - P11; P2 - P3; P2 - P8; P3 - P8
TC0400008485.hg.1	TC0400008485.hg.1	NA	NA	NA	27.108	3.81E-06	5.4192	6.43E-06	ctrl - P10; ctrl - P11; ctrl - P3; ctrl - P8; P11 - P10; P2 - P10; P11 - P3; P11 - P8; P2 - P3; P2 - P8
TC0400008147.hg.1	TC0400008147.hg.1	joybubo.aAug10-unspliced	joybubo	NA	27.096	3.82E-06	5.4181	6.44E-06	ctrl - P10; P11 - ctrl; ctrl - P3; ctrl - P8; P11 - P10; P2 - P10; P11 - P2; P11 - P3; P11 - P8; P2 - P3; P2 - P8
TC1700008380.hg.1	TC1700008380.hg.1	jawserbo.aAug10-unspliced	jawserbo	NA	27.094	3.82E-06	5.418	6.44E-06	ctrl - P10; ctrl - P11; ctrl - P8; P2 - P10; P3 - P10; P2 - P11; P3 - P11; P11 - P8; P2 - P3; P2 - P8; P3 - P8
TC1600009680.hg.1	TC1600009680.hg.1	NA	NA	NA	27.042	3.86E-06	5.4135	6.50E-06	ctrl - P10; ctrl - P11; P2 - ctrl; ctrl - P3; ctrl - P8; P2 - P10; P2 - P11; P2 - P3; P2 - P8
TC0100011382.hg.1	TC0100011382.hg.1	NM_001004023	DYRK3	8444	27.016	3.88E-06	5.4112	6.53E-06	ctrl - P10; ctrl - P2; ctrl - P3; ctrl - P8; P11 - P10; P2 - P10; P3 - P10; P11 - P2; P11 - P3; P11 - P8; P2 - P8; P3 - P8
TC0200014089.hg.1	TC0200014089.hg.1	ronemo.bAug10-unspliced	ronemo	NA	26.998	3.89E-06	5.4097	6.55E-06	ctrl - P10; ctrl - P11; ctrl - P2; ctrl - P8; P11 - P10; P2 - P10; P3 - P10; P2 - P11; P3 - P11; P11 - P8; P2 - P8; P3 - P8

TC0800007871.hg.1	TC0800007871.hg.1	uc064nlz.1	RNA5SP268	NA	26.957	3.93E-06	5.4061	6.60E-06	ctrl - P10; ctrl - P11; P2 - ctrl; ctrl - P3; ctrl - P8; P2 - P10; P2 - P11; P2 - P3; P2 - P8
TC0800007676.hg.1	TC0800007676.hg.1	munumu.aAug10-unspliced	munumu	NA	26.932	3.95E-06	5.4039	6.63E-06	ctrl - P10; ctrl - P11; P2 - ctrl; ctrl - P8; P2 - P10; P3 - P10; P2 - P11; P3 - P11; P2 - P3; P2 - P8; P3 - P8
TC0600007899.hg.1	TC0600007899.hg.1	leegloby.aAug10-unspliced	leegloby	NA	26.932	3.95E-06	5.4039	6.63E-06	ctrl - P10; ctrl - P11; ctrl - P3; ctrl - P8; P2 - P10; P3 - P10; P2 - P11; P3 - P11; P2 - P3; P2 - P8; P3 - P8
TC0100008795.hg.1	TC0100008795.hg.1	NA	NA	NA	26.902	3.97E-06	5.4013	6.66E-06	ctrl - P10; ctrl - P3; ctrl - P8; P11 - P10; P2 - P10; P3 - P10; P2 - P11; P11 - P8; P2 - P3; P2 - P8; P3 - P8
TC1700009659.hg.1	TC1700009659.hg.1	NA	NA	NA	26.868	4.00E-06	5.3984	6.70E-06	ctrl - P10; ctrl - P11; ctrl - P3; ctrl - P8; P11 - P10; P2 - P10; P2 - P11; P11 - P3; P2 - P3; P2 - P8
TC0900010278.hg.1	TC0900010278.hg.1	NR_046175_2	LOC286297	NA	26.857	4.01E-06	5.3974	6.71E-06	P10 - ctrl; P3 - ctrl; P8 - ctrl; P10 - P11; P10 - P2; P3 - P10; P8 - P10; P3 - P11; P8 - P11; P3 - P2; P8 - P2
TC1700007382.hg.1	TC1700007382.hg.1	kercho.aAug10-unspliced	kercho	NA	26.708	4.13E-06	5.3843	6.91E-06	ctrl - P10; ctrl - P11; ctrl - P3; ctrl - P8; P2 - P10; P2 - P11; P11 - P8; P2 - P3; P2 - P8; P3 - P8
TC0100015113.hg.1	TC0100015113.hg.1	ENST00000438604	RP5-1108M17.5	NA	26.685	4.15E-06	5.3823	6.94E-06	ctrl - P10; ctrl - P11; P2 - ctrl; ctrl - P3; ctrl - P8; P2 - P10; P2 - P11; P11 - P3; P11 - P8; P2 - P3; P2 - P8
TC0200012340.hg.1	TC0200012340.hg.1	blyklaw.aAug10-unspliced	blyklaw	NA	26.656	4.17E-06	5.3797	6.97E-06	P10 - ctrl; P2 - ctrl; P10 - P11; P2 - P10; P10 - P3; P10 - P8; P2 - P11; P2 - P3; P2 - P8
TC1100007999.hg.1	TC1100007999.hg.1	heti.aAug10-unspliced	heti	NA	26.655	4.17E-06	5.3796	6.97E-06	ctrl - P10; ctrl - P11; ctrl - P3; ctrl - P8; P11 - P10; P2 - P10; P2 - P11; P11 - P8; P2 - P3; P2 - P8
TC1100012825.hg.1	TC1100012825.hg.1	uc058jdc.1	AP003041.1	NA	26.627	4.20E-06	5.3771	7.01E-06	P10 - ctrl; P11 - ctrl; P3 - ctrl; P8 - ctrl; P10 - P2; P10 - P3; P11 - P2; P11 - P3; P3 - P2; P8 - P2; P8 - P3
TC1100009818.hg.1	TC1100009818.hg.1	sawkeeby.aAug10-unspliced	sawkeeby	NA	26.619	4.20E-06	5.3765	7.02E-06	ctrl - P10; ctrl - P11; ctrl - P3; ctrl - P8; P2 - P10; P10 - P8; P2 - P11; P11 - P8; P2 - P3; P2 - P8; P3 - P8
TC1300007836.hg.1	TC1300007836.hg.1	jydeeby.aAug10-unspliced	jydeeby	NA	26.602	4.22E-06	5.375	7.04E-06	ctrl - P10; ctrl - P11; ctrl - P3; ctrl - P8; P11 - P10; P2 - P10; P2 - P11; P11 - P8; P2 - P3; P2 - P8; P3 - P8
TC0100015212.hg.1	TC0100015212.hg.1	NM_024526	EPS8L3	79574	26.561	4.25E-06	5.3713	7.09E-06	P10 - ctrl; P11 - ctrl; P2 - ctrl; P3 - ctrl; P8 - ctrl; P10 - P11; P10 - P2; P10 - P3; P8 - P11; P8 - P2; P8 - P3
TC2000008818.hg.1	TC2000008818.hg.1	deyroybu.bAug10-unspliced	deyroybu	NA	26.53	4.28E-06	5.3686	7.13E-06	ctrl - P11; P2 - ctrl; P3 - ctrl; ctrl - P8; P10 - P11; P2 - P10; P3 - P10; P10 - P8; P2 - P11; P3 - P11; P2 - P8; P3 - P8
TC0800011691.hg.1	TC0800011691.hg.1	NM_001003954	ANXA13	312	26.52	4.29E-06	5.3677	7.14E-06	ctrl - P10; P11 - ctrl; P3 - ctrl; P11 - P10; P2 - P10; P3 - P10; P3 - P11; P11 - P8; P3 - P2; P2 - P8; P3 - P8
TC0100007270.hg.1	TC0100007270.hg.1	ENST00000606754.1	RP1-224A6.9		26.507	4.30E-06	5.3666	7.15E-06	ctrl - P10; ctrl - P11; ctrl - P3; ctrl - P8; P2 - P10; P3 - P10; P2 - P11; P3 - P11; P2 - P3; P2 - P8; P3 - P8
TC1000011515.hg.1	TC1000011515.hg.1	NM_001114094	BLNK	29760	26.478	4.32E-06	5.3641	7.19E-06	ctrl - P10; ctrl - P2; ctrl - P3; ctrl - P8; P11 - P10; P10 - P3; P11 - P2; P11 - P3; P11 - P8; P2 - P3
TC0600011972.hg.1	TC0600011972.hg.1	meyjee.aAug10-unspliced	meyjee	NA	26.435	4.36E-06	5.3602	7.25E-06	P10 - ctrl; P2 - ctrl; P3 - ctrl; P8 - ctrl; P3 - P10; P8 - P10; P2 - P11; P3 - P11; P8 - P11; P3 - P2; P8 - P2
TC1500006584.hg.1	TC1500006584.hg.1	NR_003298	SNORD115-6	15q11.2	26.405	4.39E-06	5.3576	7.29E-06	P10 - ctrl; P3 - ctrl; P8 - ctrl; P10 - P11; P10 - P2; P3 - P10; P3 - P11; P8 - P11; P3 - P2; P8 - P2; P3 - P8
TC1500007739.hg.1	TC1500007739.hg.1	NM_001286429	THSD4	79875	26.383	4.41E-06	5.3556	7.32E-06	P10 - ctrl; ctrl - P2; P3 - ctrl; P8 - ctrl; P10 - P2; P3 - P10; P8 - P10; P11 - P2; P3 - P11; P8 - P11; P3 - P2; P8 - P2
TC0200010682.hg.1	TC0200010682.hg.1	NA	NA	NA	26.371	4.42E-06	5.3546	7.33E-06	ctrl - P10; ctrl - P3; ctrl - P8; P11 - P10; P2 - P10; P10 - P3; P11 - P3; P11 - P8; P2 - P3; P2 - P8
TC0200010412.hg.1	TC0200010412.hg.1	stagabu.aAug10-unspliced	stagabu	NA	26.364	4.43E-06	5.3539	7.34E-06	ctrl - P10; ctrl - P11; ctrl - P8; P11 - P10; P2 - P10; P3 - P10; P2 - P11; P3 - P11; P11 - P8; P2 - P3; P2 - P8; P3 - P8
TC2200008593.hg.1	TC2200008593.hg.1	NA	NA	NA	26.328	4.46E-06	5.3507	7.39E-06	P10 - ctrl; P11 - ctrl; P8 - ctrl; P10 - P11; P10 - P2; P10 - P3; P11 - P2; P8 - P11; P8 - P2; P8 - P3
TC0800010799.hg.1	TC0800010799.hg.1	NA	NA	NA	26.311	4.47E-06	5.3492	7.41E-06	ctrl - P10; ctrl - P11; ctrl - P8; P11 - P10; P2 - P10; P3 - P10; P8 - P10; P11 - P8; P2 - P8; P3 - P8
TC0600009147.hg.1	TC0600009147.hg.1	ENST00000607434.1	RP5-1112D6.8		26.254	4.53E-06	5.3441	7.49E-06	ctrl - P10; ctrl - P11; P2 - ctrl; ctrl - P8; P11 - P10; P2 - P10; P3 - P10; P2 - P11; P3 - P11; P11 - P8; P2 - P3; P2 - P8; P3 - P8
TC0900011306.hg.1	TC0900011306.hg.1	fyshor.aAug10-unspliced	fyshor	NA	26.243	4.54E-06	5.3432	7.50E-06	ctrl - P10; ctrl - P11; ctrl - P2; P3 - ctrl; ctrl - P8; P10 - P2; P3 - P10; P3 - P11; P3 - P2; P3 - P8
TC0900006760.hg.1	TC0900006760.hg.1	NA	NA	NA	26.22	4.56E-06	5.3411	7.53E-06	ctrl - P10; ctrl - P11; ctrl - P3; ctrl - P8; P11 - P10; P2 - P10; P3 - P10; P2 - P11; P11 - P8; P2 - P3; P2 - P8; P3 - P8
TC1100011845.hg.1	TC1100011845.hg.1	jerglerbu.aAug10-unspliced	jerglerbu	NA	26.218	4.56E-06	5.3409	7.53E-06	ctrl - P10; ctrl - P11; ctrl - P3; ctrl - P8; P2 - P10; P10 - P8; P2 - P11; P11 - P8; P2 - P3; P2 - P8; P3 - P8
TC1100012019.hg.1	TC1100012019.hg.1	NM_001271594	SESN3	143686	26.183	4.59E-06	5.3378	7.58E-06	ctrl - P10; ctrl - P11; ctrl - P8; P11 - P10; P2 - P10; P3 - P10; P3 - P11; P11 - P8; P3 - P2; P2 - P8; P3 - P8
TC0200009602.hg.1	TC0200009602.hg.1	blydeybu.aAug10-unspliced	blydeybu	NA	26.146	4.63E-06	5.3344	7.63E-06	ctrl - P10; ctrl - P11; ctrl - P3; ctrl - P8; P11 - P10; P2 - P10; P3 - P10; P10 - P8; P2 - P11; P11 - P8; P2 - P3; P2 - P8; P3 - P8
TC0X00010107.hg.1	TC0X00010107.hg.1	NM_001146256	ZDHHC15	158866	26.144	4.63E-06	5.3343	7.63E-06	ctrl - P10; ctrl - P11; ctrl - P2; ctrl - P8; P11 - P10; P3 - P10; P11 - P2; P3 - P11; P11 - P8; P3 - P2; P3 - P8
TC0200007097.hg.1	TC0200007097.hg.1	ENST00000604052.1	RP11-373D23.2		26.117	4.66E-06	5.3319	7.67E-06	ctrl - P10; ctrl - P11; ctrl - P3; ctrl - P8; P2 - P10; P3 - P10; P2 - P11; P3 - P11; P2 - P3; P2 - P8; P3 - P8

TC0200013759.hg.1	TC0200013759.hg.1	ENST00000427050.2	AC009505.2		26.051	4.72E-06	5.326	7.77E-06	P10 - ctrl; P11 - ctrl; P8 - ctrl; P10 - P2; P10 - P3; P11 - P2; P11 - P3; P8 - P2; P8 - P3
TC0400012082.hg.1	TC0400012082.hg.1	blarbeebo.aAug10-unspliced	blarbeebo	NA	26.038	4.73E-06	5.3248	7.78E-06	ctrl - P10; ctrl - P11; ctrl - P3; ctrl - P8; P11 - P10; P2 - P10; P2 - P11; P11 - P3; P11 - P8; P2 - P3; P2 - P8
TC0400012744.hg.1	TC0400012744.hg.1	NA	NA	NA	26.001	4.77E-06	5.3215	7.84E-06	ctrl - P10; ctrl - P11; ctrl - P3; ctrl - P8; P11 - P10; P2 - P10; P3 - P10; P2 - P11; P11 - P8; P2 - P3; P2 - P8; P3 - P8
TC0500011069.hg.1	TC0500011069.hg.1	cherstarby.aAug10-unspliced	cherstarby	NA	25.973	4.80E-06	5.3189	7.88E-06	ctrl - P10; ctrl - P11; ctrl - P3; ctrl - P8; P2 - P10; P10 - P8; P2 - P11; P11 - P8; P2 - P3; P2 - P8; P3 - P8
TC0800010283.hg.1	TC0800010283.hg.1	NA	NA	NA	25.855	4.92E-06	5.3083	8.07E-06	P10 - ctrl; P11 - ctrl; P3 - ctrl; P8 - ctrl; P10 - P11; P10 - P2; P10 - P3; P11 - P2; P11 - P3; P3 - P2; P8 - P2; P8 - P3
TC0300011415.hg.1	TC0300011415.hg.1	jeenarbo.aAug10-unspliced	jeenarbo	NA	25.85	4.92E-06	5.3079	8.07E-06	ctrl - P10; ctrl - P11; ctrl - P3; ctrl - P8; P11 - P10; P2 - P10; P3 - P10; P2 - P11; P11 - P8; P2 - P3; P2 - P8; P3 - P8
TC0400010788.hg.1	TC0400010788.hg.1	huke.aAug10-unspliced	huke	NA	25.82	4.95E-06	5.3051	8.12E-06	ctrl - P10; ctrl - P11; ctrl - P8; P2 - P10; P3 - P10; P10 - P8; P2 - P11; P3 - P11; P11 - P8; P2 - P8; P3 - P8
TC0900008055.hg.1	TC0900008055.hg.1	chershee.aAug10-unspliced	chershee	NA	25.812	4.96E-06	5.3044	8.13E-06	ctrl - P10; ctrl - P11; P2 - ctrl; ctrl - P3; ctrl - P8; P2 - P10; P2 - P11; P2 - P3; P2 - P8; P3 - P8
TC0600014308.hg.1	TC0600014308.hg.1	NR_029598	MIR30C2	407032	25.797	4.98E-06	5.3031	8.14E-06	ctrl - P10; ctrl - P11; ctrl - P3; ctrl - P8; P10 - P11; P2 - P10; P10 - P8; P2 - P11; P3 - P11; P2 - P3; P2 - P8; P3 - P8
TC2200007420.hg.1	TC2200007420.hg.1	soytey.aAug10-unspliced	soytey	NA	25.786	4.99E-06	5.3021	8.16E-06	ctrl - P10; ctrl - P11; ctrl - P3; ctrl - P8; P11 - P10; P2 - P10; P3 - P10; P2 - P11; P11 - P8; P2 - P3; P2 - P8; P3 - P8
TC0X00009094.hg.1	TC0X00009094.hg.1	bleyvy.bAug10	bleyvy		25.777	5.00E-06	5.3012	8.17E-06	P10 - ctrl; P11 - ctrl; P3 - ctrl; P8 - ctrl; P10 - P11; P10 - P2; P8 - P10; P3 - P11; P8 - P11; P3 - P2; P8 - P2; P8 - P3
TC0200013800.hg.1	TC0200013800.hg.1	NA	NA	NA	25.746	5.03E-06	5.2984	8.22E-06	ctrl - P10; ctrl - P2; ctrl - P3; ctrl - P8; P11 - P10; P2 - P10; P3 - P10; P11 - P2; P11 - P3; P11 - P8; P2 - P8; P3 - P8
TC0800010685.hg.1	TC0800010685.hg.1	NM_001080416	MYBL1	4603	25.715	5.06E-06	5.2956	8.26E-06	ctrl - P11; ctrl - P2; P3 - ctrl; P8 - ctrl; P10 - P11; P10 - P2; P3 - P10; P8 - P10; P3 - P11; P8 - P11; P3 - P2; P8 - P2
TC0400008146.hg.1	TC0400008146.hg.1	kasnuby.aAug10-unspliced	kasnuby	NA	25.71	5.07E-06	5.2951	8.26E-06	ctrl - P10; P11 - ctrl; ctrl - P3; ctrl - P8; P11 - P10; P2 - P10; P11 - P2; P11 - P3; P11 - P8; P2 - P3; P2 - P8
TC1100009822.hg.1	TC1100009822.hg.1	flucher.aAug10-unspliced	flucher	NA	25.71	5.07E-06	5.2951	8.26E-06	ctrl - P10; ctrl - P11; ctrl - P3; ctrl - P8; P2 - P10; P2 - P11; P11 - P8; P2 - P3; P2 - P8; P3 - P8
TC1500008188.hg.1	TC1500008188.hg.1	ENST00000559487	CTD-2262B20.1	NA	25.705	5.07E-06	5.2947	8.26E-06	ctrl - P10; ctrl - P11; P2 - ctrl; ctrl - P8; P2 - P10; P3 - P10; P2 - P11; P3 - P11; P2 - P8; P3 - P8
TC1600011547.hg.1	TC1600011547.hg.1	ENST00000563003.1	RP11-96H17.1		25.701	5.08E-06	5.2944	8.27E-06	ctrl - P10; ctrl - P2; ctrl - P3; ctrl - P8; P11 - P10; P2 - P10; P3 - P10; P11 - P2; P11 - P3; P11 - P8; P2 - P3; P2 - P8; P3 - P8
TC0500010772.hg.1	TC0500010772.hg.1	blarflar.aAug10-unspliced	blarflar	NA	25.673	5.11E-06	5.2918	8.31E-06	ctrl - P10; ctrl - P11; ctrl - P3; ctrl - P8; P11 - P10; P2 - P10; P3 - P10; P2 - P11; P2 - P3; P2 - P8; P3 - P8
TC0X00011227.hg.1	TC0X00011227.hg.1	NR_036049	MIR1184-1	1E+08	25.661	5.12E-06	5.2907	8.32E-06	P10 - ctrl; P11 - ctrl; P3 - ctrl; P8 - ctrl; P10 - P2; P10 - P3; P11 - P2; P11 - P3; P3 - P2; P8 - P2; P8 - P3
TC0X00011242.hg.1	TC0X00011242.hg.1	ENST00000408263	MIR1184-2	1E+08	25.661	5.12E-06	5.2907	8.32E-06	P10 - ctrl; P11 - ctrl; P3 - ctrl; P8 - ctrl; P10 - P2; P10 - P3; P11 - P2; P11 - P3; P3 - P2; P8 - P2; P8 - P3
TC1900008022.hg.1	TC1900008022.hg.1	derwu.aAug10-unspliced	derwu	NA	25.629	5.16E-06	5.2878	8.37E-06	ctrl - P10; ctrl - P11; ctrl - P3; ctrl - P8; P2 - P10; P3 - P10; P2 - P11; P11 - P8; P2 - P3; P2 - P8; P3 - P8
TC0X00006659.hg.1	TC0X00006659.hg.1	NA	NA	NA	25.621	5.16E-06	5.287	8.38E-06	ctrl - P10; ctrl - P11; ctrl - P3; ctrl - P8; P11 - P10; P2 - P10; P2 - P11; P2 - P3; P2 - P8
TC1200007053.hg.1	TC1200007053.hg.1	NM_030572	SPX	80763	25.571	5.22E-06	5.2825	8.46E-06	ctrl - P10; ctrl - P2; ctrl - P3; ctrl - P8; P11 - P10; P2 - P10; P3 - P10; P8 - P10; P11 - P2; P11 - P3; P11 - P8; P3 - P2; P3 - P8
TC1100011105.hg.1	TC1100011105.hg.1	uc058cnz.1	RNU6-118P	NA	25.55	5.24E-06	5.2806	8.49E-06	ctrl - P10; ctrl - P11; ctrl - P3; ctrl - P8; P11 - P10; P2 - P10; P3 - P10; P2 - P11; P11 - P8; P2 - P3; P2 - P8; P3 - P8
TC0700012276.hg.1	TC0700012276.hg.1	NM_014705	DOCK4	9732	25.549	5.24E-06	5.2805	8.49E-06	P10 - ctrl; P3 - ctrl; P8 - ctrl; P10 - P11; P10 - P2; P8 - P10; P3 - P11; P8 - P11; P3 - P2; P8 - P2
TC1700010258.hg.1	TC1700010258.hg.1	tarserbo.aAug10-unspliced	tarserbo	NA	25.506	5.29E-06	5.2765	8.56E-06	ctrl - P10; ctrl - P11; P2 - ctrl; ctrl - P8; P11 - P10; P2 - P10; P3 - P10; P2 - P11; P3 - P11; P11 - P8; P2 - P3; P2 - P8; P3 - P8
TC0300013740.hg.1	TC0300013740.hg.1	gerstuby.aAug10-unspliced	gerstuby	NA	25.459	5.34E-06	5.2723	8.64E-06	ctrl - P10; ctrl - P11; P2 - ctrl; ctrl - P8; P2 - P10; P3 - P10; P2 - P11; P3 - P11; P2 - P3; P2 - P8; P3 - P8
TC1000011977.hg.1	TC1000011977.hg.1	OTTHUMT00000473353	RP11-427L15.2		25.446	5.36E-06	5.271	8.66E-06	ctrl - P10; ctrl - P11; ctrl - P3; ctrl - P8; P2 - P10; P3 - P10; P10 - P8; P2 - P11; P11 - P8; P2 - P3; P2 - P8; P3 - P8
TC0200015619.hg.1	TC0200015619.hg.1	muhamo.aAug10-unspliced	muhamo	NA	25.41	5.40E-06	5.2678	8.72E-06	ctrl - P10; ctrl - P3; ctrl - P8; P11 - P10; P2 - P10; P10 - P3; P10 - P8; P11 - P3; P11 - P8; P2 - P3; P2 - P8
TC0400007496.hg.1	TC0400007496.hg.1	snospawby.aAug10-unspliced	snospawby	NA	25.357	5.46E-06	5.2629	8.81E-06	ctrl - P10; ctrl - P11; ctrl - P8; P2 - P10; P3 - P10; P2 - P11; P3 - P11; P11 - P8; P2 - P3; P2 - P8; P3 - P8
TC1300009727.hg.1	TC1300009727.hg.1	NA	NA	NA	25.298	5.53E-06	5.2574	8.92E-06	ctrl - P10; ctrl - P11; P2 - ctrl; ctrl - P8; P2 - P10; P3 - P10; P2 - P11; P3 - P11; P11 - P8; P2 - P8; P3 - P8
TC0300012459.hg.1	TC0300012459.hg.1	NA	NA	NA	25.293	5.53E-06	5.257	8.92E-06	ctrl - P10; ctrl - P2; ctrl - P3; ctrl - P8; P11 - P10; P2 - P10; P3 - P10; P11 - P2; P11 - P3; P11 - P8; P2 - P8; P3 - P8
TC1300009811.hg.1	TC1300009811.hg.1	wonoy.aAug10-unspliced	wonoy	NA	25.289	5.54E-06	5.2566	8.92E-06	ctrl - P10; ctrl - P11; ctrl - P3; ctrl - P8; P11 - P10; P2 - P10; P2 - P11; P11 - P8; P2 - P3; P2 - P8; P3 - P8
TC1000010302.hg.1	TC1000010302.hg.1	vamo.aAug10-unspliced	vamo	NA	25.257	5.58E-06	5.2537	8.98E-06	ctrl - P10; ctrl - P11; ctrl - P8; P2 - P10; P3 - P10; P2 - P11; P3 - P11; P11 - P8; P2 - P3; P2 - P8; P3 - P8

TC0100014661.hg.1	TC0100014661.hg.1	snerneebu.aAug10-unspliced	snerneebu	NA	25.227	5.61E-06	5.2509	9.03E-06	ctrl - P10; ctrl - P11; ctrl - P3; ctrl - P8; P11 - P10; P2 - P10; P3 - P10; P2 - P11; P11 - P8; P2 - P3; P2 - P8; P3 - P8
TC0300007201.hg.1	TC0300007201.hg.1	ENST00000568686.1	RP4-555D20.2		25.214	5.63E-06	5.2497	9.05E-06	ctrl - P10; ctrl - P11; ctrl - P2; ctrl - P8; P11 - P10; P2 - P10; P3 - P10; P3 - P11; P3 - P2; P2 - P8; P3 - P8
TC0800010248.hg.1	TC0800010248.hg.1	ENST00000581909.1	RP11-360L9.8		25.197	5.65E-06	5.2481	9.08E-06	P10 - ctrl; P11 - ctrl; P3 - ctrl; P8 - ctrl; P10 - P11; P10 - P2; P10 - P3; P11 - P2; P8 - P11; P3 - P2; P8 - P2; P8 - P3
HTA2-pos-2985890_st	23150247	NA	NA	NA	25.157	5.70E-06	5.2444	9.14E-06	ctrl - P10; ctrl - P11; P2 - ctrl; ctrl - P3; ctrl - P8; P11 - P10; P2 - P10; P3 - P10; P2 - P11; P11 - P8; P2 - P3; P2 - P8; P3 - P8
TC1000008434.hg.1	TC1000008434.hg.1	stawsho.aAug10-unspliced	stawsho	NA	25.153	5.70E-06	5.2441	9.14E-06	ctrl - P10; ctrl - P11; ctrl - P3; ctrl - P8; P2 - P10; P2 - P11; P11 - P8; P2 - P3; P2 - P8; P3 - P8
TC0300013070.hg.1	TC0300013070.hg.1	flojawbu.aAug10-unspliced	flojawbu	NA	25.153	5.70E-06	5.2441	9.14E-06	ctrl - P10; ctrl - P11; ctrl - P3; ctrl - P8; P11 - P10; P2 - P10; P10 - P8; P2 - P11; P11 - P3; P11 - P8; P2 - P3; P2 - P8; P3 - P8
TC0100014262.hg.1	TC0100014262.hg.1	NA	NA	NA	25.153	5.70E-06	5.244	9.14E-06	ctrl - P10; ctrl - P11; ctrl - P3; ctrl - P8; P2 - P10; P3 - P10; P10 - P8; P2 - P11; P3 - P11; P11 - P8; P2 - P8; P3 - P8
TC0100011095.hg.1	TC0100011095.hg.1	NA	NA	NA	25.103	5.76E-06	5.2394	9.23E-06	ctrl - P10; ctrl - P11; ctrl - P3; ctrl - P8; P11 - P10; P2 - P10; P2 - P11; P11 - P3; P11 - P8; P2 - P3; P2 - P8
TC0100018323.hg.1	TC0100018323.hg.1	NM_016545	IER5	51278	25.083	5.79E-06	5.2375	9.26E-06	P10 - ctrl; P11 - ctrl; P3 - ctrl; P8 - ctrl; P10 - P11; P10 - P2; P8 - P10; P11 - P2; P3 - P11; P8 - P11; P3 - P2; P8 - P2
TC1700010256.hg.1	TC1700010256.hg.1	ENST00000582881.1	RP11-68I3.11		25.081	5.79E-06	5.2374	9.26E-06	ctrl - P10; ctrl - P11; ctrl - P3; ctrl - P8; P11 - P10; P2 - P10; P2 - P11; P11 - P8; P2 - P3; P2 - P8; P3 - P8
TC1800007506.hg.1	TC1800007506.hg.1	NM_001270949	TNFRSF11A	8792	25.051	5.83E-06	5.2346	9.32E-06	P10 - ctrl; P3 - ctrl; P8 - ctrl; P10 - P11; P10 - P2; P3 - P11; P8 - P11; P3 - P2; P8 - P2
TC0700006585.hg.1	TC0700006585.hg.1	ENST00000430569	RP11-30B1.1		25.018	5.87E-06	5.2315	9.37E-06	ctrl - P10; ctrl - P11; ctrl - P3; ctrl - P8; P2 - P10; P3 - P10; P2 - P11; P11 - P8; P2 - P3; P2 - P8; P3 - P8
TC2000007324.hg.1	TC2000007324.hg.1	tatey.aAug10-unspliced	tatey	NA	25.016	5.87E-06	5.2313	9.37E-06	ctrl - P10; ctrl - P11; ctrl - P3; ctrl - P8; P2 - P10; P3 - P10; P2 - P11; P11 - P8; P2 - P3; P2 - P8; P3 - P8
TC1000008926.hg.1	TC1000008926.hg.1	NM_016234	ACSL5	51703	24.967	5.93E-06	5.2267	9.47E-06	ctrl - P2; P3 - ctrl; P8 - ctrl; P10 - P2; P3 - P10; P8 - P10; P11 - P2; P3 - P11; P8 - P11; P3 - P2; P8 - P2
TC0400012078.hg.1	TC0400012078.hg.1	NM_000901	NR3C2	4306	24.9	6.02E-06	5.2205	9.60E-06	ctrl - P10; ctrl - P2; ctrl - P3; ctrl - P8; P11 - P10; P2 - P10; P11 - P2; P11 - P3; P11 - P8; P2 - P3; P2 - P8
TC0400008055.hg.1	TC0400008055.hg.1	foygy.aAug10-unspliced	foygy	NA	24.861	6.07E-06	5.2169	9.67E-06	ctrl - P10; ctrl - P11; ctrl - P3; ctrl - P8; P2 - P10; P10 - P8; P2 - P11; P11 - P8; P2 - P3; P2 - P8; P3 - P8
TC0900007863.hg.1	TC0900007863.hg.1	NM_001827	CKS2	1164	24.825	6.12E-06	5.2135	9.74E-06	P10 - ctrl; P3 - ctrl; P8 - ctrl; P10 - P11; P10 - P2; P8 - P10; P3 - P11; P8 - P11; P3 - P2; P8 - P2; P8 - P3
TC0800009287.hg.1	TC0800009287.hg.1	fugorby.aAug10-unspliced	fugorby	NA	24.798	6.15E-06	5.211	9.79E-06	P10 - ctrl; P11 - ctrl; P3 - ctrl; P8 - ctrl; P10 - P2; P10 - P3; P11 - P2; P11 - P3; P3 - P2; P8 - P2; P8 - P3
TC0900011029.hg.1	TC0900011029.hg.1	NM_001127610	BAAT	570	24.782	6.17E-06	5.2094	9.82E-06	P10 - ctrl; P8 - ctrl; P10 - P11; P10 - P2; P10 - P3; P10 - P8; P8 - P11; P8 - P2; P8 - P3
TC0800008723.hg.1	TC0800008723.hg.1	sneyskerbu.aAug10-unspliced	sneyskerbu	NA	24.761	6.20E-06	5.2075	9.86E-06	ctrl - P10; ctrl - P11; P2 - ctrl; ctrl - P8; P10 - P11; P2 - P10; P10 - P8; P2 - P11; P3 - P11; P2 - P3; P2 - P8; P3 - P8
TC1600006603.hg.1	TC1600006603.hg.1	uc059pkt.1	AC009065.4		24.693	6.29E-06	5.201	1.00E-05	P10 - ctrl; P11 - ctrl; P8 - ctrl; P10 - P2; P10 - P3; P11 - P2; P11 - P3; P8 - P2; P8 - P3
TC1100012691.hg.1	TC1100012691.hg.1	dorchor.aAug10-unspliced	dorchor	NA	24.689	6.30E-06	5.2007	1.00E-05	ctrl - P10; ctrl - P11; ctrl - P3; ctrl - P8; P2 - P10; P2 - P11; P11 - P8; P2 - P3; P2 - P8
TC0500007894.hg.1	TC0500007894.hg.1	vystawby.aAug10-unspliced	vystawby	NA	24.614	6.40E-06	5.1936	1.02E-05	ctrl - P10; ctrl - P11; ctrl - P3; ctrl - P8; P2 - P10; P2 - P11; P2 - P3; P2 - P8
TC2000007060.hg.1	TC2000007060.hg.1	NA	NA	NA	24.588	6.44E-06	5.1912	1.02E-05	ctrl - P10; ctrl - P11; ctrl - P3; ctrl - P8; P11 - P10; P2 - P10; P2 - P11; P11 - P8; P2 - P3; P2 - P8
TC1000008271.hg.1	TC1000008271.hg.1	NM_001171971	CDHR1	92211	24.559	6.48E-06	5.1884	1.03E-05	ctrl - P11; ctrl - P2; P3 - ctrl; P8 - ctrl; P10 - P11; P3 - P10; P8 - P10; P3 - P11; P8 - P11; P3 - P2; P8 - P2
TC0100009989.hg.1	TC0100009989.hg.1	snoporbo.aAug10-unspliced	snoporbo	NA	24.482	6.59E-06	5.1811	1.04E-05	ctrl - P10; ctrl - P11; ctrl - P8; P2 - P10; P3 - P10; P10 - P8; P2 - P11; P3 - P11; P11 - P8; P2 - P8; P3 - P8
TC1300006861.hg.1	TC1300006861.hg.1	NM_001204197	NBEA	26960	24.444	6.65E-06	5.1775	1.05E-05	ctrl - P10; ctrl - P8; P11 - P10; P2 - P10; P3 - P10; P11 - P3; P11 - P8; P2 - P8; P3 - P8
TC0700007999.hg.1	TC0700007999.hg.1	NR_030321	MIR590	693175	24.413	6.69E-06	5.1746	1.06E-05	ctrl - P10; ctrl - P11; ctrl - P3; ctrl - P8; P2 - P10; P3 - P10; P2 - P11; P3 - P11; P2 - P3; P2 - P8; P3 - P8
TC1200011245.hg.1	TC1200011245.hg.1	NM_001109754	PTPRB	5787	24.389	6.73E-06	5.1723	1.06E-05	P10 - ctrl; P3 - ctrl; P8 - ctrl; P10 - P11; P10 - P2; P10 - P3; P3 - P11; P8 - P11; P3 - P2; P8 - P2; P8 - P3
TC1900008024.hg.1	TC1900008024.hg.1	kuriru.aAug10-unspliced	kuriru	NA	24.363	6.76E-06	5.1698	1.07E-05	ctrl - P10; ctrl - P11; ctrl - P8; P2 - P10; P3 - P10; P2 - P11; P3 - P11; P2 - P3; P2 - P8; P3 - P8
TC0200014908.hg.1	TC0200014908.hg.1	slerfeebu.aAug10-unspliced	slerfeebu	NA	24.362	6.77E-06	5.1697	1.07E-05	ctrl - P10; ctrl - P2; ctrl - P3; ctrl - P8; P11 - P10; P2 - P10; P3 - P10; P11 - P3; P11 - P8; P2 - P8; P3 - P8
TC1300008117.hg.1	TC1300008117.hg.1	NR_107042	MIR8075	1.02E+08	24.33	6.81E-06	5.1666	1.07E-05	ctrl - P10; ctrl - P11; ctrl - P3; ctrl - P8; P2 - P10; P2 - P11; P2 - P3; P2 - P8
TC0200012092.hg.1	TC0200012092.hg.1	NM_001142683	CCDC121	79635	24.312	6.84E-06	5.1649	1.08E-05	ctrl - P10; ctrl - P2; ctrl - P3; ctrl - P8; P11 - P10; P10 - P3; P11 - P2; P11 - P3; P11 - P8; P8 - P3

TC1400010101.hg.1	TC1400010101.hg.1	NA	NA	NA	24.311	6.84E-06	5.1649	1.08E-05	ctrl - P10; ctrl - P11; ctrl - P3; ctrl - P8; P2 - P10; P2 - P11; P11 - P8; P2 - P3; P2 - P8; P3 - P8
TC1200007014.hg.1	TC1200007014.hg.1	flafy.bAug10-unspliced	flafy	NA	24.272	6.90E-06	5.1611	1.09E-05	ctrl - P10; ctrl - P11; P2 - ctrl; ctrl - P8; P2 - P10; P3 - P10; P2 - P11; P3 - P11; P11 - P8; P2 - P3; P2 - P8; P3 - P8
TC1300008946.hg.1	TC1300008946.hg.1	gychy.aAug10-unspliced	gychy	NA	24.271	6.90E-06	5.161	1.09E-05	ctrl - P10; P2 - ctrl; ctrl - P3; ctrl - P8; P11 - P10; P2 - P10; P3 - P10; P2 - P11; P11 - P8; P2 - P3; P2 - P8; P3 - P8
TC0700006717.hg.1	TC0700006717.hg.1	chorshybu.aAug10-unspliced	chorshybu	NA	24.265	6.91E-06	5.1604	1.09E-05	ctrl - P10; P2 - ctrl; ctrl - P8; P11 - P10; P2 - P10; P3 - P10; P2 - P11; P11 - P8; P2 - P3; P2 - P8; P3 - P8
TC1600010292.hg.1	TC1600010292.hg.1	wuglu.aAug10-unspliced	wuglu	NA	24.252	6.93E-06	5.1592	1.09E-05	ctrl - P10; ctrl - P11; ctrl - P3; ctrl - P8; P11 - P10; P2 - P10; P3 - P10; P11 - P8; P2 - P3; P2 - P8; P3 - P8
TC0500010199.hg.1	TC0500010199.hg.1	NM_001034850	FAM134B	54463	24.235	6.96E-06	5.1575	1.09E-05	P10 - ctrl; P3 - ctrl; P8 - ctrl; P10 - P11; P10 - P2; P3 - P11; P8 - P11; P3 - P2; P8 - P2
TC0800008855.hg.1	TC0800008855.hg.1	NR_031610	MIR1205	1E+08	24.214	6.99E-06	5.1556	1.10E-05	P2 - ctrl; P3 - ctrl; P10 - P11; P2 - P10; P3 - P10; P2 - P11; P3 - P11; P8 - P11; P2 - P3; P2 - P8; P3 - P8
TC1100009126.hg.1	TC1100009126.hg.1	NA	NA	NA	24.199	7.01E-06	5.1542	1.10E-05	P10 - ctrl; P11 - ctrl; P8 - ctrl; P10 - P11; P10 - P2; P10 - P3; P11 - P2; P11 - P3; P8 - P2; P8 - P3
TC0100007899.hg.1	TC0100007899.hg.1	storkloy.aAug10-unspliced	storkloy	NA	24.192	7.02E-06	5.1535	1.10E-05	ctrl - P10; ctrl - P11; ctrl - P3; ctrl - P8; P2 - P10; P3 - P10; P2 - P11; P11 - P8; P2 - P3; P2 - P8; P3 - P8
TC0800011584.hg.1	TC0800011584.hg.1	sneela.aAug10-unspliced	sneela	NA	24.175	7.05E-06	5.1518	1.10E-05	ctrl - P10; ctrl - P11; ctrl - P8; P10 - P11; P2 - P10; P10 - P8; P2 - P11; P3 - P11; P2 - P3; P2 - P8; P3 - P8
TC0300006438.hg.1	TC0300006438.hg.1	skardubo.aAug10-unspliced	skardubo	NA	24.125	7.13E-06	5.147	1.12E-05	ctrl - P10; ctrl - P11; ctrl - P2; ctrl - P3; ctrl - P8; P11 - P10; P2 - P10; P11 - P3; P11 - P8; P2 - P3; P2 - P8
TC0100018274.hg.1	TC0100018274.hg.1	ENST00000495965	ATP1A1	1p21	24.105	7.16E-06	5.1451	1.12E-05	ctrl - P10; ctrl - P11; ctrl - P2; ctrl - P3; ctrl - P8; P2 - P10; P2 - P11; P2 - P3; P2 - P8
TC0300011261.hg.1	TC0300011261.hg.1	farnabo.aAug10-unspliced	farnabo	NA	24.051	7.25E-06	5.1398	1.13E-05	ctrl - P10; ctrl - P11; P2 - ctrl; ctrl - P3; ctrl - P8; P11 - P10; P2 - P10; P10 - P8; P2 - P11; P11 - P8; P2 - P3; P2 - P8; P3 - P8
TC0200006676.hg.1	TC0200006676.hg.1	ENST00000607140.1	RP11-254F7.4		23.996	7.34E-06	5.1346	1.15E-05	ctrl - P10; ctrl - P11; ctrl - P2; ctrl - P3; ctrl - P8; P11 - P10; P2 - P10; P11 - P3; P11 - P8; P2 - P3; P2 - P8
TC0100009385.hg.1	TC0100009385.hg.1	ENST00000609909.1	RP5-1074L1.4		23.978	7.37E-06	5.1328	1.15E-05	ctrl - P10; ctrl - P11; ctrl - P3; ctrl - P8; P2 - P10; P10 - P8; P2 - P11; P11 - P8; P2 - P3; P2 - P8; P3 - P8
TC2000009130.hg.1	TC2000009130.hg.1	ENST00000606979	RP3-511B24.5	NA	23.968	7.38E-06	5.1319	1.15E-05	ctrl - P10; ctrl - P11; P2 - ctrl; ctrl - P8; P2 - P10; P3 - P10; P2 - P11; P3 - P11; P2 - P3; P2 - P8; P3 - P8
TC1900007432.hg.1	TC1900007432.hg.1	seypey.aAug10-unspliced	seypey	NA	23.963	7.39E-06	5.1314	1.15E-05	ctrl - P10; ctrl - P11; ctrl - P3; ctrl - P8; P2 - P10; P2 - P11; P2 - P3; P2 - P8
TC1900008134.hg.1	TC1900008134.hg.1	NM_000767	CYP2B6	1555	23.925	7.45E-06	5.1277	1.16E-05	ctrl - P10; P11 - ctrl; ctrl - P2; ctrl - P3; ctrl - P8; P11 - P10; P11 - P2; P11 - P3; P11 - P8
TC1100007301.hg.1	TC1100007301.hg.1	NM_001160167	PRR5L	79899	23.904	7.49E-06	5.1256	1.17E-05	P10 - ctrl; ctrl - P11; ctrl - P2; P8 - ctrl; P10 - P11; P10 - P2; P11 - P2; P3 - P11; P8 - P11; P3 - P2; P8 - P2; P8 - P3
TC1800008894.hg.1	TC1800008894.hg.1	NA	NA	NA	23.9	7.49E-06	5.1252	1.17E-05	ctrl - P10; ctrl - P11; ctrl - P3; ctrl - P8; P2 - P10; P10 - P8; P2 - P11; P11 - P8; P2 - P3; P2 - P8
TC0400008106.hg.1	TC0400008106.hg.1	NM_016323	HERC5	51191	23.899	7.50E-06	5.1252	1.17E-05	P10 - ctrl; P3 - ctrl; P8 - ctrl; P10 - P11; P10 - P2; P3 - P11; P8 - P11; P3 - P2; P8 - P2; P8 - P3
TC0300009773.hg.1	TC0300009773.hg.1	NM_001308077	FETUB	26998	23.868	7.55E-06	5.1222	1.17E-05	P10 - ctrl; P3 - ctrl; P8 - ctrl; P10 - P11; P10 - P2; P3 - P10; P8 - P10; P3 - P11; P8 - P11; P3 - P2; P8 - P2
TC0100008525.hg.1	TC0100008525.hg.1	snawperbu.aAug10-unspliced	snawperbu	NA	23.853	7.57E-06	5.1207	1.18E-05	ctrl - P10; ctrl - P3; ctrl - P8; P11 - P10; P2 - P10; P3 - P10; P11 - P8; P2 - P8; P3 - P8
TC0X00010957.hg.1	TC0X00010957.hg.1	someru.aAug10-unspliced	someru	NA	23.804	7.66E-06	5.1159	1.19E-05	ctrl - P10; ctrl - P2; ctrl - P3; ctrl - P8; P11 - P10; P2 - P10; P11 - P3; P11 - P8; P2 - P3; P2 - P8
TC0700009727.hg.1	TC0700009727.hg.1	worko.aAug10-unspliced	worko	NA	23.781	7.70E-06	5.1137	1.19E-05	ctrl - P10; ctrl - P11; P2 - ctrl; ctrl - P3; ctrl - P8; P2 - P10; P2 - P11; P2 - P3; P2 - P8; P3 - P8
TC0200008634.hg.1	TC0200008634.hg.1	typobo.aAug10-unspliced	typobo	NA	23.717	7.81E-06	5.1075	1.21E-05	ctrl - P10; ctrl - P11; ctrl - P3; ctrl - P8; P11 - P10; P2 - P10; P3 - P10; P11 - P3; P11 - P8; P2 - P3; P2 - P8
TC0600011464.hg.1	TC0600011464.hg.1	ENST00000229729	SLC44A4	80736	23.671	7.89E-06	5.103	1.22E-05	P10 - ctrl; P11 - ctrl; P8 - ctrl; P10 - P11; P10 - P2; P10 - P3; P11 - P2; P11 - P3; P8 - P11; P8 - P2; P8 - P3
TC1200007616.hg.1	TC1200007616.hg.1	chawjorby.aAug10-unspliced	chawjorby	NA	23.618	7.98E-06	5.0978	1.24E-05	ctrl - P10; ctrl - P11; ctrl - P3; ctrl - P8; P2 - P10; P10 - P8; P2 - P11; P2 - P3; P2 - P8; P3 - P8
TC1500007337.hg.1	TC1500007337.hg.1	skaspee.aAug10-unspliced	skaspee	NA	23.602	8.01E-06	5.0963	1.24E-05	ctrl - P10; ctrl - P11; ctrl - P3; ctrl - P8; P2 - P10; P2 - P11; P2 - P3; P2 - P8; P3 - P8
TC1200012601.hg.1	TC1200012601.hg.1	NM_019844	SLCO1B3	28234	23.549	8.11E-06	5.091	1.25E-05	P10 - ctrl; P3 - ctrl; P8 - ctrl; P10 - P11; P10 - P2; P3 - P10; P8 - P10; P3 - P11; P8 - P11; P3 - P2; P8 - P2
TC0200007416.hg.1	TC0200007416.hg.1	reefa.aAug10-unspliced	reefa	NA	23.517	8.17E-06	5.0878	1.26E-05	ctrl - P10; ctrl - P11; ctrl - P3; ctrl - P8; P2 - P10; P10 - P8; P2 - P11; P11 - P8; P2 - P3; P2 - P8; P3 - P8
TC0400010244.hg.1	TC0400010244.hg.1	pogu.aAug10-unspliced	pogu	NA	23.512	8.18E-06	5.0874	1.26E-05	ctrl - P10; ctrl - P11; ctrl - P3; ctrl - P8; P11 - P10; P2 - P10; P10 - P8; P11 - P3; P11 - P8; P2 - P3; P2 - P8
TC0600006801.hg.1	TC0600006801.hg.1	NA	NA	NA	23.46	8.27E-06	5.0823	1.28E-05	ctrl - P10; ctrl - P11; P2 - ctrl; ctrl - P3; ctrl - P8; P2 - P10; P2 - P11; P3 - P11; P2 - P3; P2 - P8; P3 - P8

TC0600009216.hg.1	TC0600009216.hg.1	NA	NA	NA	23.417	8.36E-06	5.078	1.29E-05	P10 - ctrl; P3 - ctrl; P8 - ctrl; P10 - P11; P10 - P2; P8 - P10; P3 - P11; P8 - P11; P3 - P2; P8 - P2; P8 - P3
TC0X00009515.hg.1	TC0X00009515.hg.1	ENST00000602277.1	RP6-99M1.3		23.397	8.39E-06	5.0761	1.29E-05	ctrl - P10; ctrl - P11; ctrl - P8; P2 - P10; P3 - P10; P2 - P11; P3 - P11; P2 - P8; P3 - P8
TC0800006883.hg.1	TC0800006883.hg.1	swarslobu.aAug10-unspliced	swarslobu	NA	23.393	8.40E-06	5.0757	1.30E-05	ctrl - P10; ctrl - P11; P2 - ctrl; ctrl - P3; ctrl - P8; P2 - P10; P10 - P8; P2 - P11; P2 - P3; P2 - P8
TC1400007389.hg.1	TC1400007389.hg.1	rerzer.aAug10-unspliced	rerzer	NA	23.372	8.44E-06	5.0735	1.30E-05	ctrl - P11; ctrl - P8; P10 - P11; P2 - P10; P3 - P10; P10 - P8; P2 - P11; P3 - P11; P2 - P8; P3 - P8
TC0700007389.hg.1	TC0700007389.hg.1	blusheybu.bAug10-unspliced	blusheybu	NA	23.368	8.45E-06	5.0732	1.30E-05	P10 - ctrl; P11 - ctrl; P3 - ctrl; P8 - ctrl; P10 - P2; P10 - P3; P11 - P2; P11 - P3; P3 - P2; P8 - P2; P8 - P3
TC1100008272.hg.1	TC1100008272.hg.1	loysha.aAug10-unspliced	loysha	NA	23.304	8.57E-06	5.0668	1.32E-05	ctrl - P10; ctrl - P11; ctrl - P3; ctrl - P8; P2 - P10; P3 - P10; P2 - P11; P2 - P3; P2 - P8; P3 - P8
TC1300009928.hg.1	TC1300009928.hg.1	NA	NA	NA	23.295	8.59E-06	5.0659	1.32E-05	ctrl - P10; ctrl - P11; ctrl - P8; P2 - P10; P3 - P10; P2 - P11; P3 - P11; P2 - P8; P3 - P8
TC0200008535.hg.1	TC0200008535.hg.1	smeekybo.aAug10-unspliced	smeekybo	NA	23.284	8.61E-06	5.0648	1.32E-05	ctrl - P10; ctrl - P11; ctrl - P3; ctrl - P8; P11 - P10; P2 - P10; P3 - P10; P2 - P11; P11 - P8; P2 - P3; P2 - P8; P3 - P8
TC0300007058.hg.1	TC0300007058.hg.1	lorfarbo.aAug10-unspliced	lorfarbo	NA	23.247	8.69E-06	5.0612	1.33E-05	ctrl - P10; ctrl - P11; ctrl - P3; ctrl - P8; P11 - P10; P2 - P10; P3 - P10; P2 - P11; P11 - P8; P2 - P3; P2 - P8; P3 - P8
TC0500010498.hg.1	TC0500010498.hg.1	NM_001161429	RANBP3L	202151	23.19	8.80E-06	5.0555	1.35E-05	ctrl - P10; ctrl - P11; ctrl - P2; ctrl - P3; ctrl - P8; P11 - P10; P11 - P2; P11 - P3; P11 - P8
TC0500012392.hg.1	TC0500012392.hg.1	NM_003122	SPINK1	6690	23.187	8.81E-06	5.0552	1.35E-05	P10 - ctrl; P2 - ctrl; P8 - ctrl; P10 - P11; P10 - P2; P10 - P3; P2 - P11; P3 - P11; P8 - P11; P8 - P2; P8 - P3
TC1100009297.hg.1	TC1100009297.hg.1	rorkarby.aAug10-unspliced	rorkarby	NA	23.18	8.82E-06	5.0545	1.35E-05	ctrl - P10; ctrl - P11; ctrl - P3; ctrl - P8; P2 - P10; P10 - P8; P2 - P11; P11 - P8; P2 - P3; P2 - P8; P3 - P8
TC0800007675.hg.1	TC0800007675.hg.1	munumu.bAug10-unspliced	munumu	NA	23.14	8.90E-06	5.0506	1.36E-05	ctrl - P10; P2 - ctrl; P3 - ctrl; ctrl - P8; P2 - P10; P3 - P10; P2 - P11; P3 - P11; P2 - P3; P2 - P8; P3 - P8
TC0400008326.hg.1	TC0400008326.hg.1	NR_046840	ARHGEF38-IT1	1.01E+08	23.138	8.91E-06	5.0503	1.36E-05	ctrl - P10; ctrl - P11; ctrl - P3; ctrl - P8; P2 - P10; P10 - P8; P2 - P11; P11 - P8; P2 - P3; P2 - P8
TC1700007479.hg.1	TC1700007479.hg.1	ENST00000584382.1	RP11-142O6.1		23.109	8.96E-06	5.0475	1.37E-05	ctrl - P10; ctrl - P11; ctrl - P3; ctrl - P8; P2 - P10; P2 - P11; P2 - P3; P2 - P8
TC0900010894.hg.1	TC0900010894.hg.1	plartawby.aAug10-unspliced	plartawby	NA	23.087	9.01E-06	5.0452	1.38E-05	ctrl - P10; P2 - ctrl; ctrl - P3; ctrl - P8; P11 - P10; P2 - P10; P2 - P11; P11 - P3; P11 - P8; P2 - P3; P2 - P8
TC0Y00007303.hg.1	TC0Y00007303.hg.1	sharbo.aAug10-unspliced	sharbo	NA	23.049	9.09E-06	5.0414	1.39E-05	ctrl - P10; ctrl - P11; ctrl - P2; ctrl - P3; ctrl - P8; P11 - P10; P3 - P10; P11 - P2; P3 - P2; P8 - P2; P3 - P8
TC0Y00007308.hg.1	TC0Y00007308.hg.1	blerbo.aAug10-unspliced	blerbo	NA	23.049	9.09E-06	5.0414	1.39E-05	ctrl - P10; ctrl - P11; ctrl - P2; ctrl - P3; ctrl - P8; P11 - P10; P3 - P10; P11 - P2; P3 - P2; P8 - P2; P3 - P8
HTA2-pos-PSR02023992.hg.1	23151081	NA	NA	NA	23.039	9.11E-06	5.0404	1.39E-05	ctrl - P10; ctrl - P11; ctrl - P3; ctrl - P8; P11 - P10; P2 - P10; P3 - P10; P2 - P11; P2 - P3; P2 - P8; P3 - P8
TC1200011362.hg.1	TC1200011362.hg.1	NA	NA	NA	23.035	9.12E-06	5.04	1.39E-05	ctrl - P11; P2 - ctrl; ctrl - P8; P10 - P11; P2 - P10; P3 - P10; P10 - P8; P2 - P11; P3 - P11; P2 - P3; P2 - P8; P3 - P8
TC0900006759.hg.1	TC0900006759.hg.1	NA	NA	NA	22.954	9.29E-06	5.0319	1.42E-05	ctrl - P10; ctrl - P11; ctrl - P3; ctrl - P8; P2 - P10; P10 - P8; P2 - P11; P11 - P8; P2 - P3; P2 - P8; P3 - P8
TC1400008185.hg.1	TC1400008185.hg.1	bleezey.aAug10-unspliced	bleezey	NA	22.862	9.49E-06	5.0227	1.45E-05	ctrl - P10; ctrl - P11; P2 - ctrl; ctrl - P3; ctrl - P8; P2 - P10; P2 - P11; P2 - P3; P2 - P8
TC1600009426.hg.1	TC1600009426.hg.1	slyrar.aAug10-unspliced	slyrar	NA	22.858	9.50E-06	5.0222	1.45E-05	ctrl - P10; ctrl - P11; ctrl - P3; ctrl - P8; P11 - P10; P2 - P10; P2 - P11; P11 - P3; P11 - P8; P2 - P3; P2 - P8
TC0200013602.hg.1	TC0200013602.hg.1	NM_025244	TSGA10	80705	22.81	9.61E-06	5.0174	1.46E-05	ctrl - P10; ctrl - P3; ctrl - P8; P11 - P10; P2 - P10; P11 - P3; P11 - P8; P2 - P3; P2 - P8
TC0700007045.hg.1	TC0700007045.hg.1	NANOGP4.aAug10-unspliced	NANOGP4	NA	22.807	9.61E-06	5.0171	1.46E-05	ctrl - P10; ctrl - P11; ctrl - P8; P11 - P10; P2 - P10; P3 - P10; P2 - P11; P11 - P8; P2 - P8; P3 - P8
TC1200012176.hg.1	TC1200012176.hg.1	ENST00000535720.1	RP11-173P15.3		22.801	9.63E-06	5.0165	1.46E-05	P10 - ctrl; P11 - ctrl; P3 - ctrl; P8 - ctrl; P10 - P2; P11 - P2; P3 - P2; P8 - P2
TC0200014742.hg.1	TC0200014742.hg.1	wawly.aAug10-unspliced	wawly	NA	22.793	9.65E-06	5.0156	1.47E-05	ctrl - P10; ctrl - P11; ctrl - P3; ctrl - P8; P11 - P10; P2 - P10; P3 - P10; P2 - P11; P11 - P8; P2 - P3; P2 - P8; P3 - P8
TC0200010537.hg.1	TC0200010537.hg.1	spykeybo.aAug10-unspliced	spykeybo	NA	22.776	9.68E-06	5.0139	1.47E-05	ctrl - P10; P2 - ctrl; ctrl - P8; P11 - P10; P2 - P10; P3 - P10; P10 - P8; P11 - P8; P2 - P3; P2 - P8; P3 - P8
TC1100007695.hg.1	TC1100007695.hg.1	kise.aAug10-unspliced	kise	NA	22.762	9.72E-06	5.0126	1.47E-05	ctrl - P10; ctrl - P11; P2 - ctrl; ctrl - P8; P2 - P10; P3 - P10; P2 - P11; P3 - P11; P11 - P8; P2 - P3; P2 - P8; P3 - P8
TC0X00009072.hg.1	TC0X00009072.hg.1	NA	NA	NA	22.755	9.73E-06	5.0118	1.48E-05	ctrl - P10; ctrl - P11; ctrl - P3; ctrl - P8; P11 - P10; P2 - P10; P3 - P10; P2 - P11; P11 - P8; P2 - P3; P2 - P8; P3 - P8
TC1100009290.hg.1	TC1100009290.hg.1	blymey.aAug10-unspliced	blymey	NA	22.744	9.76E-06	5.0107	1.48E-05	ctrl - P10; ctrl - P11; ctrl - P3; ctrl - P8; P2 - P10; P3 - P10; P10 - P8; P2 - P11; P11 - P8; P2 - P3; P2 - P8; P3 - P8
HTA2-pos-PSR02023982.hg.1	23151075	NA	NA	NA	22.733	9.78E-06	5.0096	1.48E-05	ctrl - P10; ctrl - P11; ctrl - P8; P11 - P10; P2 - P10; P3 - P10; P2 - P11; P11 - P8; P2 - P3; P2 - P8; P3 - P8
TC0X00008336.hg.1	TC0X00008336.hg.1	spybu.aAug10-unspliced	spybu	NA	22.718	9.82E-06	5.0081	1.49E-05	ctrl - P10; ctrl - P11; ctrl - P3; ctrl - P8; P2 - P10; P3 - P10; P2 - P11; P3 - P11; P2 - P3; P2 - P8

TC1900009095.hg.1	TC1900009095.hg.1	NA	NA	NA	22.697	9.86E-06	5.0059	1.49E-05	P10 - ctrl; P11 - ctrl; ctrl - P2; P3 - ctrl; P8 - ctrl; P10 - P2; P10 - P3; P11 - P2; P11 - P3; P3 - P2; P8 - P2
TC0300011950.hg.1	TC0300011950.hg.1	NM_005459	GUCA1C	9626	22.684	9.89E-06	5.0046	1.50E-05	ctrl - P11; ctrl - P2; ctrl - P3; ctrl - P8; P10 - P11; P10 - P2; P10 - P3; P8 - P11; P8 - P2; P8 - P3
TC0200009955.hg.1	TC0200009955.hg.1	NM_001127383	CYBRD1	79901	22.675	9.92E-06	5.0037	1.50E-05	ctrl - P10; ctrl - P11; ctrl - P2; P3 - ctrl; P10 - P2; P3 - P10; P3 - P11; P8 - P11; P3 - P2; P8 - P2; P3 - P8
TC0900007622.hg.1	TC0900007622.hg.1	shyplorbu.aAug10-unspliced	shyplorbu	NA	22.657	9.96E-06	5.0018	1.50E-05	ctrl - P10; ctrl - P11; P2 - ctrl; ctrl - P3; ctrl - P8; P2 - P10; P10 - P8; P2 - P11; P11 - P8; P2 - P3; P2 - P8; P3 - P8
TC1900008727.hg.1	TC1900008727.hg.1	NM_001191055	ERVV-2	1E+08	22.651	9.97E-06	5.0013	1.50E-05	P10 - ctrl; P11 - ctrl; P3 - ctrl; P8 - ctrl; P10 - P2; P3 - P10; P8 - P10; P11 - P2; P3 - P11; P8 - P11; P3 - P2; P8 - P2
TC1300008769.hg.1	TC1300008769.hg.1	dorchaw.aAug10-unspliced	dorchaw	NA	22.65	9.97E-06	5.0012	1.50E-05	ctrl - P10; ctrl - P8; P11 - P10; P2 - P10; P3 - P10; P11 - P3; P11 - P8; P2 - P3; P2 - P8; P3 - P8
TC0100007444.hg.1	TC0100007444.hg.1	NM_001297647	CNKSR1	10256	22.647	9.98E-06	5.0008	1.50E-05	ctrl - P10; P11 - ctrl; ctrl - P2; ctrl - P8; P11 - P10; P3 - P10; P11 - P2; P11 - P3; P11 - P8; P3 - P2; P3 - P8
TC0100017979.hg.1	TC0100017979.hg.1	NA	NA	NA	22.642	9.99E-06	5.0004	1.51E-05	ctrl - P10; ctrl - P11; P2 - ctrl; ctrl - P8; P2 - P10; P3 - P10; P2 - P11; P3 - P11; P2 - P3; P2 - P8; P3 - P8
TC1500007322.hg.1	TC1500007322.hg.1	OTTHUMT00000473983	RP11-48G14.3		22.629	1.00E-05	4.999	1.51E-05	ctrl - P10; ctrl - P11; ctrl - P3; ctrl - P8; P11 - P10; P2 - P10; P2 - P11; P11 - P8; P2 - P3; P2 - P8
TC0900009418.hg.1	TC0900009418.hg.1	flupawbu.aAug10-unspliced	flupawbu	NA	22.615	1.01E-05	4.9976	1.51E-05	ctrl - P10; ctrl - P11; ctrl - P3; ctrl - P8; P2 - P10; P2 - P11; P11 - P3; P11 - P8; P2 - P3; P2 - P8
TC0600007039.hg.1	TC0600007039.hg.1	larfleyby.aAug10-unspliced	larfleyby	NA	22.606	1.01E-05	4.9966	1.52E-05	ctrl - P10; ctrl - P11; ctrl - P3; ctrl - P8; P2 - P10; P2 - P11; P2 - P3; P2 - P8
TC1600010318.hg.1	TC1600010318.hg.1	koysno.aAug10-unspliced	koysno	NA	22.603	1.01E-05	4.9964	1.52E-05	ctrl - P10; ctrl - P11; P2 - ctrl; ctrl - P3; ctrl - P8; P2 - P10; P3 - P10; P2 - P11; P3 - P11; P2 - P3; P2 - P8; P3 - P8
TC0200012285.hg.1	TC0200012285.hg.1	NA	NA	NA	22.593	1.01E-05	4.9954	1.52E-05	P10 - ctrl; P11 - ctrl; P3 - ctrl; P8 - ctrl; P10 - P2; P10 - P3; P11 - P2; P11 - P3; P3 - P2; P8 - P2
TC0100006504.hg.1	TC0100006504.hg.1	NA	NA	NA	22.592	1.01E-05	4.9953	1.52E-05	P10 - ctrl; P11 - ctrl; P8 - ctrl; P10 - P11; P10 - P2; P10 - P3; P10 - P8; P11 - P2; P11 - P3; P3 - P2; P8 - P2; P8 - P3
TC0X00008087.hg.1	TC0X00008087.hg.1	koho.aAug10-unspliced	koho	NA	22.591	1.01E-05	4.9951	1.52E-05	ctrl - P10; ctrl - P11; ctrl - P8; P11 - P10; P2 - P10; P3 - P10; P2 - P11; P11 - P8; P2 - P3; P2 - P8; P3 - P8
TC0400011169.hg.1	TC0400011169.hg.1	zeeko.aAug10-unspliced	zeeko	NA	22.576	1.01E-05	4.9937	1.52E-05	ctrl - P10; ctrl - P11; ctrl - P3; ctrl - P8; P11 - P10; P2 - P10; P2 - P11; P11 - P8; P2 - P3; P2 - P8
TC0300006567.hg.1	TC0300006567.hg.1	ENST00000602411.1	RP11-1020A11.1		22.574	1.02E-05	4.9934	1.52E-05	ctrl - P10; ctrl - P11; ctrl - P3; ctrl - P8; P11 - P10; P2 - P10; P2 - P11; P11 - P8; P2 - P3; P2 - P8; P3 - P8
TC0100018058.hg.1	TC0100018058.hg.1	lorleebo.aAug10-unspliced	lorleebo	NA	22.557	1.02E-05	4.9916	1.53E-05	ctrl - P10; ctrl - P11; ctrl - P8; P2 - P10; P3 - P10; P2 - P11; P11 - P8; P2 - P3; P2 - P8; P3 - P8
TC0400011645.hg.1	TC0400011645.hg.1	mygla.aAug10-unspliced	mygla	NA	22.538	1.02E-05	4.9898	1.53E-05	ctrl - P10; ctrl - P11; ctrl - P3; ctrl - P8; P2 - P10; P3 - P10; P2 - P11; P3 - P11; P2 - P8; P3 - P8
TC1800007189.hg.1	TC1800007189.hg.1	peywo.aAug10-unspliced	peywo	NA	22.53	1.03E-05	4.9889	1.53E-05	ctrl - P10; ctrl - P11; ctrl - P3; ctrl - P8; P11 - P10; P2 - P10; P3 - P10; P2 - P11; P11 - P8; P2 - P3; P2 - P8; P3 - P8
TC0400012081.hg.1	TC0400012081.hg.1	gasnoyby.aAug10-unspliced	gasnoyby	NA	22.515	1.03E-05	4.9874	1.54E-05	ctrl - P10; ctrl - P3; ctrl - P8; P11 - P10; P2 - P10; P11 - P3; P11 - P8; P2 - P3; P2 - P8; P3 - P8
TC0700010560.hg.1	TC0700010560.hg.1	shawky.aAug10-unspliced	shawky	NA	22.51	1.03E-05	4.9869	1.54E-05	ctrl - P10; ctrl - P11; ctrl - P3; ctrl - P8; P11 - P10; P2 - P10; P2 - P11; P11 - P3; P11 - P8; P2 - P3; P2 - P8
TC1100007512.hg.1	TC1100007512.hg.1	flawfloybu.aAug10-unspliced	flawfloybu	NA	22.487	1.04E-05	4.9845	1.55E-05	ctrl - P10; ctrl - P11; ctrl - P8; P10 - P11; P3 - P10; P10 - P8; P2 - P11; P3 - P11; P2 - P8; P3 - P8
TC0800011415.hg.1	TC0800011415.hg.1	ENST00000474907	RPL12P24	NA	22.473	1.04E-05	4.983	1.55E-05	ctrl - P10; ctrl - P11; P2 - ctrl; ctrl - P3; ctrl - P8; P2 - P10; P10 - P8; P2 - P11; P11 - P3; P11 - P8; P2 - P3; P2 - P8
TC0Y00007287.hg.1	TC0Y00007287.hg.1	zobo.aAug10-unspliced	zobo	NA	22.472	1.04E-05	4.983	1.55E-05	ctrl - P10; ctrl - P11; ctrl - P2; ctrl - P8; P11 - P10; P3 - P10; P8 - P10; P11 - P2; P3 - P11; P3 - P2; P3 - P8
TC0Y00007299.hg.1	TC0Y00007299.hg.1	skeybo.aAug10-unspliced	skeybo	NA	22.472	1.04E-05	4.983	1.55E-05	ctrl - P10; ctrl - P11; ctrl - P2; ctrl - P8; P11 - P10; P3 - P10; P8 - P10; P11 - P2; P3 - P11; P3 - P2; P3 - P8
TC0200014696.hg.1	TC0200014696.hg.1	ENST00000447019.1	AC019186.1		22.469	1.04E-05	4.9827	1.55E-05	ctrl - P10; ctrl - P3; ctrl - P8; P11 - P10; P2 - P10; P11 - P3; P11 - P8; P2 - P3; P2 - P8; P3 - P8
TC1400007531.hg.1	TC1400007531.hg.1	NA	NA	NA	22.44	1.05E-05	4.9797	1.56E-05	P10 - ctrl; P11 - ctrl; P3 - ctrl; P8 - ctrl; P10 - P2; P10 - P3; P11 - P2; P11 - P3; P3 - P2; P8 - P2
TC0500010858.hg.1	TC0500010858.hg.1	uc063dvx.1	RNU6-806P	NA	22.412	1.05E-05	4.9768	1.57E-05	P2 - ctrl; P3 - ctrl; P2 - P10; P3 - P10; P2 - P11; P3 - P11; P3 - P2; P2 - P8; P3 - P8
TC0400008064.hg.1	TC0400008064.hg.1	posnyby.aAug10-unspliced	posnyby	NA	22.379	1.06E-05	4.9734	1.58E-05	ctrl - P10; ctrl - P11; P2 - ctrl; ctrl - P3; ctrl - P8; P2 - P10; P10 - P8; P2 - P11; P11 - P8; P2 - P3; P2 - P8; P3 - P8
TC0700012155.hg.1	TC0700012155.hg.1	shasmorbu.aAug10-unspliced	shasmorbu	NA	22.346	1.07E-05	4.97	1.59E-05	ctrl - P10; ctrl - P11; ctrl - P3; ctrl - P8; P10 - P11; P2 - P10; P10 - P8; P2 - P11; P3 - P11; P2 - P3; P2 - P8; P3 - P8
TC1200008132.hg.1	TC1200008132.hg.1	ENST00000548895	C1GALT1P1	NA	22.301	1.08E-05	4.9654	1.61E-05	ctrl - P10; ctrl - P11; P2 - ctrl; ctrl - P3; ctrl - P8; P2 - P10; P2 - P11; P11 - P8; P2 - P3; P2 - P8
TC0100010325.hg.1	TC0100010325.hg.1	rarlee.aAug10	rarlee		22.292	1.09E-05	4.9645	1.61E-05	P10 - ctrl; P11 - ctrl; P8 - ctrl; P10 - P11; P10 - P2; P10 - P3; P11 - P2; P8 - P11; P8 - P2; P8 - P3

TC0100012762.hg.1	TC0100012762.hg.1	slernobu.aAug10-unspliced	slernobu	NA	22.276	1.09E-05	4.9628	1.62E-05	ctrl - P10; ctrl - P11; ctrl - P3; ctrl - P8; P11 - P10; P2 - P10; P2 - P11; P11 - P3; P11 - P8; P2 - P3; P2 - P8
TC1000011332.hg.1	TC1000011332.hg.1	chawdabu.aAug10-unspliced	chawdabu	NA	22.274	1.09E-05	4.9626	1.62E-05	ctrl - P10; ctrl - P11; ctrl - P3; ctrl - P8; P11 - P10; P2 - P10; P3 - P10; P2 - P11; P11 - P8; P2 - P3; P2 - P8; P3 - P8
TC0500007165.hg.1	TC0500007165.hg.1	fluflaw.aAug10-unspliced	fluflaw	NA	22.24	1.10E-05	4.9591	1.63E-05	ctrl - P10; ctrl - P11; ctrl - P3; ctrl - P8; P2 - P10; P2 - P11; P2 - P3; P2 - P8
TC0800011055.hg.1	TC0800011055.hg.1	slarzeeby.bAug10-unspliced	slarzeeby	NA	22.231	1.10E-05	4.9582	1.63E-05	ctrl - P11; P2 - ctrl; ctrl - P8; P10 - P11; P2 - P10; P10 - P8; P2 - P11; P3 - P11; P2 - P3; P2 - P8; P3 - P8
TC0500010463.hg.1	TC0500010463.hg.1	glorplarby.aAug10-unspliced	glorplarby	NA	22.23	1.10E-05	4.9581	1.63E-05	ctrl - P10; ctrl - P11; ctrl - P3; ctrl - P8; P2 - P10; P3 - P10; P2 - P11; P11 - P8; P2 - P3; P2 - P8; P3 - P8
TC1000008032.hg.1	TC1000008032.hg.1	NR_132103	PPP3CB-AS1	1.02E+08	22.168	1.12E-05	4.9517	1.65E-05	ctrl - P10; ctrl - P2; ctrl - P3; ctrl - P8; P11 - P10; P2 - P10; P11 - P2; P11 - P3; P11 - P8; P2 - P3; P2 - P8; P8 - P3
TC1200007269.hg.1	TC1200007269.hg.1	torblobu.aAug10-unspliced	torblobu	NA	22.15	1.12E-05	4.9498	1.66E-05	ctrl - P10; ctrl - P11; P2 - ctrl; ctrl - P3; ctrl - P8; P2 - P10; P10 - P8; P2 - P11; P2 - P3; P2 - P8; P3 - P8
TC0100012772.hg.1	TC0100012772.hg.1	ENST00000410579	Y_RNA		22.147	1.12E-05	4.9495	1.66E-05	ctrl - P10; ctrl - P11; ctrl - P3; ctrl - P8; P2 - P10; P10 - P8; P2 - P11; P2 - P3; P2 - P8
TC1800006603.hg.1	TC1800006603.hg.1	kitaru.aAug10-unspliced	kitaru	NA	22.137	1.13E-05	4.9484	1.66E-05	ctrl - P10; ctrl - P11; ctrl - P3; ctrl - P8; P2 - P10; P3 - P10; P2 - P11; P2 - P3; P2 - P8; P3 - P8
TC1900009933.hg.1	TC1900009933.hg.1	C19orf42.sAug10-unspliced	C19orf42	NA	22.126	1.13E-05	4.9473	1.67E-05	ctrl - P10; ctrl - P11; ctrl - P3; ctrl - P8; P2 - P10; P2 - P11; P11 - P8; P2 - P3; P2 - P8; P3 - P8
TC0800011816.hg.1	TC0800011816.hg.1	ENST00000363205	SNORA25	NA	22.107	1.13E-05	4.9453	1.67E-05	ctrl - P10; ctrl - P11; ctrl - P3; ctrl - P8; P2 - P10; P3 - P10; P10 - P8; P2 - P11; P11 - P8; P2 - P3; P2 - P8; P3 - P8
TC0100009671.hg.1	TC0100009671.hg.1	NA	NA	NA	22.074	1.14E-05	4.9419	1.68E-05	ctrl - P10; ctrl - P11; ctrl - P3; ctrl - P8; P2 - P10; P3 - P10; P2 - P11; P3 - P11; P2 - P3; P2 - P8; P3 - P8
TC0400007802.hg.1	TC0400007802.hg.1	nokime.bAug10-unspliced	nokime	NA	22.053	1.15E-05	4.9398	1.69E-05	ctrl - P10; ctrl - P3; ctrl - P8; P11 - P10; P2 - P10; P11 - P3; P11 - P8; P2 - P3; P2 - P8
TC0300008339.hg.1	TC0300008339.hg.1	ENST00000563632.1	RP11-757F18.5		22.049	1.15E-05	4.9393	1.69E-05	ctrl - P10; ctrl - P11; P2 - ctrl; ctrl - P8; P2 - P10; P3 - P10; P2 - P11; P3 - P11; P2 - P3; P2 - P8; P3 - P8
TC2200006890.hg.1	TC2200006890.hg.1	joyfer.aAug10-unspliced	joyfer	NA	21.938	1.18E-05	4.9277	1.74E-05	ctrl - P10; ctrl - P11; ctrl - P3; ctrl - P8; P11 - P10; P2 - P10; P2 - P11; P11 - P8; P2 - P3; P2 - P8; P3 - P8
TC0100014981.hg.1	TC0100014981.hg.1	vumeebo.aAug10-unspliced	vumeebo	NA	21.935	1.18E-05	4.9274	1.74E-05	ctrl - P10; ctrl - P11; ctrl - P3; ctrl - P8; P2 - P10; P3 - P10; P2 - P11; P3 - P11; P2 - P3; P2 - P8
TC1600010847.hg.1	TC1600010847.hg.1	ENST00000493458	AC009120.3	NA	21.897	1.19E-05	4.9234	1.75E-05	ctrl - P10; ctrl - P11; ctrl - P3; ctrl - P8; P11 - P10; P2 - P10; P2 - P11; P11 - P8; P2 - P3; P2 - P8; P3 - P8
TC0500011060.hg.1	TC0500011060.hg.1	deysmaby.aAug10-unspliced	deysmaby	NA	21.894	1.19E-05	4.9231	1.75E-05	ctrl - P10; ctrl - P3; ctrl - P8; P11 - P10; P2 - P10; P3 - P10; P2 - P11; P11 - P8; P2 - P3; P2 - P8; P3 - P8
TC0100018440.hg.1	TC0100018440.hg.1	NM_001048166	STIL	1p32	21.885	1.20E-05	4.9222	1.76E-05	P10 - ctrl; ctrl - P11; ctrl - P2; P8 - ctrl; P10 - P11; P10 - P2; P10 - P3; P3 - P11; P8 - P11; P3 - P2; P8 - P2; P8 - P3
TC0200011963.hg.1	TC0200011963.hg.1	NA	NA	NA	21.873	1.20E-05	4.9209	1.76E-05	ctrl - P10; ctrl - P8; P11 - P10; P2 - P10; P3 - P10; P11 - P8; P2 - P8; P3 - P8
TC0600009964.hg.1	TC0600009964.hg.1	vostaby.aAug10-unspliced	vostaby	NA	21.872	1.20E-05	4.9208	1.76E-05	ctrl - P10; ctrl - P11; ctrl - P8; P2 - P10; P3 - P10; P10 - P8; P2 - P11; P3 - P11; P11 - P8; P2 - P3; P2 - P8; P3 - P8
TC1600009946.hg.1	TC1600009946.hg.1	ENST00000567795.1	CTD-2574D22.4		21.849	1.21E-05	4.9184	1.77E-05	ctrl - P10; ctrl - P11; ctrl - P3; ctrl - P8; P2 - P10; P2 - P11; P11 - P8; P2 - P3; P2 - P8
TC0200010483.hg.1	TC0200010483.hg.1	NR_003031	SNORD11	2q33.1	21.846	1.21E-05	4.9181	1.77E-05	ctrl - P10; ctrl - P3; ctrl - P8; P11 - P10; P2 - P10; P11 - P3; P11 - P8; P2 - P3; P2 - P8
TC1500009584.hg.1	TC1500009584.hg.1	NA	NA	NA	21.827	1.21E-05	4.9161	1.77E-05	ctrl - P10; ctrl - P11; ctrl - P3; ctrl - P8; P2 - P10; P3 - P10; P2 - P11; P11 - P8; P2 - P3; P2 - P8; P3 - P8
TC0100013501.hg.1	TC0100013501.hg.1	puklor.aAug10-unspliced	puklor	NA	21.789	1.22E-05	4.9121	1.79E-05	ctrl - P10; ctrl - P11; ctrl - P3; ctrl - P8; P2 - P10; P3 - P10; P2 - P11; P3 - P11; P2 - P3; P2 - P8; P3 - P8
TC1200010743.hg.1	TC1200010743.hg.1	uc058ogq.1	AC021066.1	NA	21.749	1.24E-05	4.9079	1.81E-05	P10 - ctrl; P11 - ctrl; P3 - ctrl; P8 - ctrl; P10 - P11; P10 - P2; P10 - P3; P11 - P2; P8 - P11; P3 - P2; P8 - P2; P8 - P3
TC0200008799.hg.1	TC0200008799.hg.1	NR_037191	SULT1C2P1	151234	21.748	1.24E-05	4.9078	1.81E-05	ctrl - P10; P11 - ctrl; ctrl - P3; ctrl - P8; P11 - P10; P2 - P10; P11 - P2; P11 - P3; P11 - P8; P2 - P3; P2 - P8; P3 - P8
HTA2-pos-PSR02023984.hg.1	23151077	NA	NA	NA	21.731	1.24E-05	4.906	1.81E-05	ctrl - P10; ctrl - P11; ctrl - P3; ctrl - P8; P11 - P10; P2 - P10; P3 - P10; P2 - P11; P11 - P8; P2 - P3; P2 - P8; P3 - P8
HTA2-pos-3022516_st	23150290	NA	NA	NA	21.706	1.25E-05	4.9034	1.82E-05	ctrl - P10; ctrl - P11; ctrl - P3; ctrl - P8; P11 - P10; P2 - P10; P3 - P10; P2 - P11; P11 - P8; P2 - P3; P2 - P8; P3 - P8
TC1600009740.hg.1	TC1600009740.hg.1	blozu.aAug10-unspliced	blozu	NA	21.692	1.25E-05	4.9019	1.83E-05	ctrl - P10; ctrl - P11; ctrl - P3; ctrl - P8; P11 - P10; P2 - P10; P11 - P3; P11 - P8; P2 - P3; P2 - P8
TC0100017984.hg.1	TC0100017984.hg.1	NM_001206729	AKT3	10000	21.681	1.26E-05	4.9007	1.83E-05	ctrl - P10; ctrl - P11; ctrl - P2; ctrl - P8; P11 - P10; P2 - P10; P3 - P10; P11 - P8; P2 - P8; P3 - P8
TC0700009054.hg.1	TC0700009054.hg.1	ENST00000605836.1	RP11-274B21.10		21.666	1.26E-05	4.8992	1.84E-05	ctrl - P10; ctrl - P11; ctrl - P3; ctrl - P8; P2 - P10; P3 - P10; P2 - P11; P11 - P8; P2 - P3; P2 - P8; P3 - P8
TC1300008939.hg.1	TC1300008939.hg.1	pleyspor.aAug10-unspliced	pleyspor	NA	21.657	1.26E-05	4.8981	1.84E-05	ctrl - P10; ctrl - P11; ctrl - P3; ctrl - P8; P11 - P10; P2 - P10; P3 - P10; P2 - P11; P11 - P8; P2 - P3; P2 - P8; P3 - P8

TC0200007019.hg.1	TC0200007019.hg.1	swargloy.aAug10-unspliced	swargloy	NA	21.634	1.27E-05	4.8957	1.85E-05	ctrl - P10; ctrl - P11; ctrl - P3; ctrl - P8; P2 - P10; P2 - P11; P2 - P3; P2 - P8; P3 - P8
TC0800006873.hg.1	TC0800006873.hg.1	NM_006207	PDGFRL	5157	21.615	1.28E-05	4.8937	1.86E-05	P10 - ctrl; P11 - ctrl; P3 - ctrl; P8 - ctrl; P10 - P11; P10 - P2; P3 - P11; P8 - P11; P3 - P2; P8 - P2; P8 - P3
TC0200011189.hg.1	TC0200011189.hg.1	NR_131900	AGAP1-IT1	1.01E+08	21.612	1.28E-05	4.8934	1.86E-05	ctrl - P10; ctrl - P11; ctrl - P3; ctrl - P8; P11 - P10; P2 - P10; P3 - P10; P11 - P8; P2 - P3; P2 - P8; P3 - P8
TC1400009300.hg.1	TC1400009300.hg.1	mozer.aAug10-unspliced	mozer	NA	21.582	1.29E-05	4.8903	1.87E-05	ctrl - P10; ctrl - P11; ctrl - P2; ctrl - P3; ctrl - P8; P11 - P10; P2 - P10; P3 - P10; P11 - P8; P2 - P8; P3 - P8
TC1300008089.hg.1	TC1300008089.hg.1	skawzoby.aAug10-unspliced	skawzoby	NA	21.499	1.31E-05	4.8814	1.91E-05	ctrl - P10; ctrl - P11; ctrl - P3; ctrl - P8; P2 - P10; P10 - P8; P2 - P11; P11 - P8; P2 - P3; P2 - P8
TC1500008867.hg.1	TC1500008867.hg.1	plergler.aAug10-unspliced	plergler	NA	21.498	1.31E-05	4.8813	1.91E-05	ctrl - P10; ctrl - P11; ctrl - P3; ctrl - P8; P11 - P10; P2 - P10; P3 - P10; P2 - P11; P11 - P8; P2 - P3; P2 - P8; P3 - P8
TC1100011078.hg.1	TC1100011078.hg.1	ENST00000526045.1	CTD-2531D15.5		21.494	1.32E-05	4.8808	1.91E-05	P10 - ctrl; P11 - ctrl; P3 - ctrl; P8 - ctrl; P10 - P11; P10 - P2; P10 - P3; P11 - P2; P8 - P11; P3 - P2; P8 - P2; P8 - P3
TC0100011098.hg.1	TC0100011098.hg.1	zakorbo.aAug10-unspliced	zakorbo	NA	21.493	1.32E-05	4.8808	1.91E-05	ctrl - P10; P2 - ctrl; ctrl - P8; P11 - P10; P2 - P10; P3 - P10; P2 - P11; P11 - P8; P2 - P3; P2 - P8; P3 - P8
TC1200007011.hg.1	TC1200007011.hg.1	ENST00000501211.2	RP11-282K24.3		21.449	1.33E-05	4.8761	1.93E-05	ctrl - P10; ctrl - P11; ctrl - P3; ctrl - P8; P2 - P10; P2 - P11; P11 - P8; P2 - P3; P2 - P8; P3 - P8
TC0800011716.hg.1	TC0800011716.hg.1	kuhire.aAug10-unspliced	kuhire	NA	21.447	1.33E-05	4.8758	1.93E-05	ctrl - P10; ctrl - P11; ctrl - P8; P2 - P10; P3 - P10; P2 - P11; P3 - P11; P2 - P3; P2 - P8; P3 - P8
TC1500006575.hg.1	TC1500006575.hg.1	snychybu.aAug10-unspliced	snychybu	NA	21.429	1.34E-05	4.874	1.93E-05	ctrl - P10; ctrl - P11; ctrl - P3; ctrl - P8; P2 - P10; P2 - P11; P11 - P8; P2 - P3; P2 - P8; P3 - P8
TC0300007057.hg.1	TC0300007057.hg.1	kofarbo.aAug10-unspliced	kofarbo	NA	21.396	1.35E-05	4.8704	1.95E-05	ctrl - P10; P2 - ctrl; ctrl - P3; ctrl - P8; P11 - P10; P2 - P10; P3 - P10; P2 - P11; P11 - P8; P2 - P3; P2 - P8; P3 - P8
TC0100016940.hg.1	TC0100016940.hg.1	NA	NA	NA	21.333	1.37E-05	4.8637	1.98E-05	P10 - ctrl; P11 - ctrl; P8 - ctrl; P10 - P2; P10 - P3; P11 - P2; P11 - P3; P8 - P2; P8 - P3
TC0500012156.hg.1	TC0500012156.hg.1	chargor.aAug10-unspliced	chargor	NA	21.333	1.37E-05	4.8637	1.98E-05	ctrl - P10; ctrl - P11; P2 - ctrl; ctrl - P8; P11 - P10; P2 - P10; P3 - P10; P2 - P11; P11 - P8; P2 - P3; P2 - P8; P3 - P8
TC0100009145.hg.1	TC0100009145.hg.1	swoplo.aAug10-unspliced	swoplo	NA	21.307	1.38E-05	4.8609	1.99E-05	ctrl - P10; ctrl - P11; ctrl - P3; ctrl - P8; P2 - P10; P2 - P11; P2 - P3; P2 - P8; P3 - P8
TC1900008400.hg.1	TC1900008400.hg.1	ENST00000624074	CTD-2233K9.1		21.288	1.38E-05	4.8588	2.00E-05	ctrl - P10; ctrl - P11; ctrl - P3; ctrl - P8; P2 - P10; P2 - P11; P2 - P3; P2 - P8
TC0100012279.hg.1	TC0100012279.hg.1	NA	NA	NA	21.274	1.39E-05	4.8573	2.00E-05	ctrl - P10; ctrl - P11; ctrl - P3; ctrl - P8; P2 - P10; P3 - P10; P2 - P11; P2 - P3; P2 - P8; P3 - P8
TC1400009712.hg.1	TC1400009712.hg.1	gawsmer.aAug10-unspliced	gawsmer	NA	21.26	1.39E-05	4.8558	2.01E-05	ctrl - P10; ctrl - P11; ctrl - P3; ctrl - P8; P2 - P10; P2 - P11; P11 - P8; P2 - P3; P2 - P8
TC0200016551.hg.1	TC0200016551.hg.1	ENST00000604340.1	RP11-546J1.1		21.221	1.41E-05	4.8517	2.03E-05	ctrl - P10; ctrl - P11; ctrl - P3; ctrl - P8; P11 - P10; P2 - P10; P3 - P10; P2 - P11; P11 - P8; P2 - P3; P2 - P8; P3 - P8
TC1300008116.hg.1	TC1300008116.hg.1	uc058ymp.1	AL136221.3	NA	21.164	1.43E-05	4.8455	2.05E-05	ctrl - P10; ctrl - P11; ctrl - P3; ctrl - P8; P2 - P10; P2 - P11; P2 - P3; P2 - P8
TC1500008842.hg.1	TC1500008842.hg.1	ENST00000560740.1	RP11-680F8.4		21.145	1.43E-05	4.8434	2.06E-05	ctrl - P10; ctrl - P11; ctrl - P3; ctrl - P8; P2 - P10; P2 - P11; P11 - P8; P2 - P3; P2 - P8; P3 - P8
TC0100007304.hg.1	TC0100007304.hg.1	zyklor.aAug10-unspliced	zyklor	NA	21.129	1.44E-05	4.8417	2.07E-05	ctrl - P10; ctrl - P11; ctrl - P3; ctrl - P8; P2 - P10; P2 - P11; P11 - P8; P2 - P3; P2 - P8; P3 - P8
TC0600010129.hg.1	TC0600010129.hg.1	NA	NA	NA	21.126	1.44E-05	4.8414	2.07E-05	ctrl - P10; ctrl - P11; ctrl - P3; ctrl - P8; P11 - P10; P2 - P10; P2 - P11; P11 - P8; P2 - P3; P2 - P8; P3 - P8
TC1200011312.hg.1	TC1200011312.hg.1	lerchar.aAug10-unspliced	lerchar	NA	21.113	1.45E-05	4.84	2.07E-05	ctrl - P10; ctrl - P11; ctrl - P3; ctrl - P8; P2 - P10; P3 - P10; P2 - P11; P11 - P8; P2 - P3; P2 - P8; P3 - P8
TC1000010783.hg.1	TC1000010783.hg.1	dylor.aAug10-unspliced	dylor	NA	21.101	1.45E-05	4.8386	2.08E-05	ctrl - P10; ctrl - P11; P2 - ctrl; ctrl - P8; P2 - P10; P3 - P10; P10 - P8; P2 - P11; P3 - P11; P11 - P8; P2 - P3; P2 - P8; P3 - P8
TC0500011049.hg.1	TC0500011049.hg.1	ENST00000194097	NAIP	4671	21.084	1.46E-05	4.8369	2.09E-05	ctrl - P10; ctrl - P11; ctrl - P3; ctrl - P8; P11 - P10; P2 - P10; P3 - P10; P2 - P11; P11 - P8; P2 - P3; P2 - P8
TC0900008012.hg.1	TC0900008012.hg.1	borlar.aAug10-unspliced	borlar	NA	21.074	1.46E-05	4.8358	2.09E-05	ctrl - P10; P2 - ctrl; ctrl - P3; ctrl - P8; P11 - P10; P2 - P10; P2 - P11; P11 - P3; P11 - P8; P2 - P3; P2 - P8
TC1400008627.hg.1	TC1400008627.hg.1	pleepy.aAug10-unspliced	pleepy	NA	21.069	1.46E-05	4.8352	2.09E-05	ctrl - P10; ctrl - P11; P2 - ctrl; ctrl - P3; ctrl - P8; P2 - P10; P3 - P10; P2 - P11; P11 - P8; P2 - P3; P2 - P8; P3 - P8
TC0400010558.hg.1	TC0400010558.hg.1	NM_001105529	ATP8A1	10396	21.048	1.47E-05	4.8329	2.10E-05	ctrl - P10; ctrl - P3; ctrl - P8; P11 - P10; P2 - P10; P3 - P10; P10 - P8; P11 - P3; P11 - P8; P2 - P3; P2 - P8; P3 - P8
TC0300008341.hg.1	TC0300008341.hg.1	neebubu.aAug10-unspliced	neebubu	NA	21.023	1.48E-05	4.8303	2.11E-05	ctrl - P10; ctrl - P11; ctrl - P3; ctrl - P8; P2 - P10; P3 - P10; P2 - P11; P3 - P11; P2 - P3; P2 - P8; P3 - P8
TC0100010520.hg.1	TC0100010520.hg.1	feekloy.aAug10-unspliced	feekloy	NA	21.016	1.48E-05	4.8295	2.12E-05	ctrl - P10; ctrl - P11; ctrl - P3; ctrl - P8; P2 - P10; P3 - P10; P2 - P11; P11 - P8; P2 - P3; P2 - P8; P3 - P8
TC2000009441.hg.1	TC2000009441.hg.1	smeera.aAug10-unspliced	smeera	NA	21.008	1.48E-05	4.8285	2.12E-05	ctrl - P10; ctrl - P3; ctrl - P8; P2 - P10; P10 - P8; P2 - P11; P11 - P3; P11 - P8; P2 - P3; P2 - P8
TC0100006841.hg.1	TC0100006841.hg.1	forreybo.aAug10-unspliced	forreybo	NA	20.938	1.51E-05	4.821	2.16E-05	ctrl - P10; ctrl - P11; ctrl - P3; ctrl - P8; P11 - P10; P2 - P10; P3 - P10; P11 - P8; P2 - P3; P2 - P8; P3 - P8

TC1800008731.hg.1	TC1800008731.hg.1	parploy.aAug10-unspliced	parploy	NA	20.919	1.52E-05	4.8189	2.17E-05	ctrl - P10; ctrl - P11; ctrl - P3; ctrl - P8; P2 - P10; P10 - P8; P2 - P11; P3 - P11; P2 - P3; P2 - P8; P3 - P8
TC2100007117.hg.1	TC2100007117.hg.1	NA	NA	NA	20.896	1.53E-05	4.8164	2.18E-05	ctrl - P10; ctrl - P11; ctrl - P8; P2 - P10; P3 - P10; P10 - P8; P2 - P11; P3 - P11; P2 - P8; P3 - P8
TC0300008325.hg.1	TC0300008325.hg.1	dububu.aAug10-unspliced	dububu	NA	20.802	1.56E-05	4.8062	2.23E-05	ctrl - P10; ctrl - P3; ctrl - P8; P11 - P10; P2 - P10; P3 - P10; P2 - P11; P11 - P8; P2 - P3; P2 - P8; P3 - P8
TSUnmapped00000135.hg.1	TSUnmapped00000135.hg.1	ENST00000627169	INPP5D	3635	20.777	1.57E-05	4.8033	2.24E-05	P3 - ctrl; P8 - ctrl; P10 - P2; P3 - P10; P8 - P10; P11 - P2; P3 - P11; P8 - P11; P3 - P2; P8 - P2
TC1600007983.hg.1	TC1600007983.hg.1	ENST00000565861.1	RP11-325K4.3		20.775	1.57E-05	4.8032	2.24E-05	ctrl - P10; ctrl - P11; ctrl - P3; ctrl - P8; P2 - P10; P2 - P11; P2 - P3; P2 - P8
TC1600007172.hg.1	TC1600007172.hg.1	uc059rvx.1	RNU6-196P	NA	20.761	1.58E-05	4.8016	2.25E-05	ctrl - P10; ctrl - P11; ctrl - P3; ctrl - P8; P11 - P10; P2 - P10; P2 - P11; P11 - P8; P2 - P3; P2 - P8; P3 - P8
TC0200014738.hg.1	TC0200014738.hg.1	hsa_circ_0001077	RBMS1	NA	20.73	1.59E-05	4.7982	2.26E-05	ctrl - P10; ctrl - P11; P2 - ctrl; ctrl - P8; P11 - P10; P2 - P10; P3 - P10; P2 - P11; P11 - P8; P2 - P3; P2 - P8; P3 - P8
TC0Y00007295.hg.1	TC0Y00007295.hg.1	sybo.aAug10-unspliced	sybo	NA	20.658	1.62E-05	4.7902	2.30E-05	ctrl - P10; ctrl - P11; ctrl - P2; ctrl - P8; P11 - P10; P3 - P10; P8 - P10; P11 - P2; P3 - P11; P3 - P2; P3 - P8
TC0Y00007300.hg.1	TC0Y00007300.hg.1	shorbo.aAug10-unspliced	shorbo	NA	20.658	1.62E-05	4.7902	2.30E-05	ctrl - P10; ctrl - P11; ctrl - P2; ctrl - P8; P11 - P10; P3 - P10; P8 - P10; P11 - P2; P3 - P11; P3 - P2; P3 - P8
TC0Y00007302.hg.1	TC0Y00007302.hg.1	pleybo.aAug10-unspliced	pleybo	NA	20.658	1.62E-05	4.7902	2.30E-05	ctrl - P10; ctrl - P11; ctrl - P2; ctrl - P8; P11 - P10; P3 - P10; P8 - P10; P11 - P2; P3 - P11; P3 - P2; P3 - P8
TC0Y00007304.hg.1	TC0Y00007304.hg.1	pabo.aAug10-unspliced	pabo	NA	20.658	1.62E-05	4.7902	2.30E-05	ctrl - P10; ctrl - P11; ctrl - P2; ctrl - P8; P11 - P10; P3 - P10; P8 - P10; P11 - P2; P3 - P11; P3 - P2; P3 - P8
TC0300012378.hg.1	TC0300012378.hg.1	charvawbo.aAug10-unspliced	charvawbo	NA	20.658	1.62E-05	4.7902	2.30E-05	ctrl - P10; ctrl - P11; ctrl - P3; ctrl - P8; P2 - P10; P10 - P8; P2 - P11; P11 - P8; P2 - P3; P2 - P8; P3 - P8
TC1000007708.hg.1	TC1000007708.hg.1	NR_027508	FAM133CP	728640	20.653	1.62E-05	4.7897	2.30E-05	ctrl - P10; ctrl - P11; ctrl - P3; ctrl - P8; P2 - P10; P3 - P10; P10 - P8; P2 - P11; P3 - P11; P2 - P3; P2 - P8; P3 - P8
TC1100011839.hg.1	TC1100011839.hg.1	borchoy.aAug10-unspliced	borchoy	NA	20.65	1.62E-05	4.7894	2.30E-05	ctrl - P10; ctrl - P11; ctrl - P3; ctrl - P8; P2 - P10; P3 - P10; P2 - P11; P11 - P8; P2 - P3; P2 - P8; P3 - P8
TC0700012263.hg.1	TC0700012263.hg.1	NA	NA	NA	20.645	1.63E-05	4.7889	2.30E-05	ctrl - P10; P2 - ctrl; ctrl - P8; P11 - P10; P2 - P10; P3 - P10; P2 - P11; P11 - P8; P2 - P3; P2 - P8; P3 - P8
TC1000011989.hg.1	TC1000011989.hg.1	NR_002917	SNORA19	10q26	20.601	1.64E-05	4.784	2.33E-05	ctrl - P10; ctrl - P11; P2 - ctrl; ctrl - P3; ctrl - P8; P11 - P10; P2 - P10; P2 - P11; P2 - P3; P2 - P8
TC0400010704.hg.1	TC0400010704.hg.1	NM_001126328	LNX1	84708	20.597	1.65E-05	4.7835	2.33E-05	ctrl - P10; ctrl - P2; ctrl - P3; ctrl - P8; P11 - P10; P11 - P2; P11 - P3; P11 - P8
TC0100012763.hg.1	TC0100012763.hg.1	gamabo.aAug10-unspliced	gamabo	NA	20.593	1.65E-05	4.7831	2.33E-05	ctrl - P10; ctrl - P11; ctrl - P3; ctrl - P8; P2 - P10; P2 - P11; P11 - P8; P2 - P3; P2 - P8
TC0400011170.hg.1	TC0400011170.hg.1	lerflor.aAug10-unspliced	lerflor	NA	20.572	1.66E-05	4.7807	2.34E-05	ctrl - P10; ctrl - P11; ctrl - P3; ctrl - P8; P11 - P10; P2 - P10; P2 - P11; P11 - P8; P2 - P3; P2 - P8
TC1700008129.hg.1	TC1700008129.hg.1	ENST00000384007	Y_RNA		20.569	1.66E-05	4.7804	2.34E-05	ctrl - P10; ctrl - P11; ctrl - P3; ctrl - P8; P2 - P10; P10 - P8; P2 - P11; P3 - P11; P2 - P3; P2 - P8; P3 - P8
TC0100011631.hg.1	TC0100011631.hg.1	lyber.aAug10-unspliced	lyber	NA	20.494	1.69E-05	4.7722	2.38E-05	ctrl - P10; ctrl - P11; ctrl - P3; ctrl - P8; P11 - P10; P2 - P10; P3 - P10; P2 - P11; P11 - P8; P2 - P3; P2 - P8; P3 - P8
TC0700010449.hg.1	TC0700010449.hg.1	NR_131935	LOC541472	541472	20.489	1.69E-05	4.7716	2.39E-05	P10 - ctrl; P3 - ctrl; P8 - ctrl; P10 - P11; P10 - P2; P3 - P11; P8 - P11; P3 - P2; P8 - P2
TC0200013343.hg.1	TC0200013343.hg.1	ENST00000608142.1	RP11-1399P15.1		20.479	1.70E-05	4.7705	2.39E-05	ctrl - P10; ctrl - P11; ctrl - P2; ctrl - P3; ctrl - P8; P11 - P10; P11 - P2; P11 - P3; P11 - P8
TC1000008436.hg.1	TC1000008436.hg.1	kyparby.aAug10-unspliced	kyparby	NA	20.477	1.70E-05	4.7703	2.39E-05	ctrl - P10; ctrl - P11; ctrl - P3; ctrl - P8; P2 - P10; P3 - P10; P2 - P11; P3 - P11; P2 - P3; P2 - P8; P3 - P8
HTA2-pos-3145791_st	23150341	NA	NA	NA	20.379	1.74E-05	4.7594	2.45E-05	ctrl - P10; ctrl - P11; ctrl - P3; ctrl - P8; P11 - P10; P2 - P10; P3 - P10; P2 - P11; P2 - P3; P2 - P8; P3 - P8
TC1500007556.hg.1	TC1500007556.hg.1	shorzo.aAug10-unspliced	shorzo	NA	20.355	1.75E-05	4.7566	2.46E-05	ctrl - P10; ctrl - P11; ctrl - P3; ctrl - P8; P10 - P11; P2 - P10; P10 - P8; P2 - P11; P3 - P11; P2 - P3; P2 - P8; P3 - P8
TC1900007796.hg.1	TC1900007796.hg.1	rycho.aAug10-unspliced	rycho	NA	20.348	1.75E-05	4.7558	2.47E-05	ctrl - P10; ctrl - P11; P2 - ctrl; ctrl - P8; P11 - P10; P2 - P10; P3 - P10; P2 - P11; P11 - P8; P2 - P3; P2 - P8; P3 - P8
TC0X00010164.hg.1	TC0X00010164.hg.1	NR_110646	CHMP1B2P	1.01E+08	20.272	1.79E-05	4.7474	2.51E-05	ctrl - P10; ctrl - P3; ctrl - P8; P11 - P10; P2 - P10; P3 - P10; P2 - P11; P11 - P8; P2 - P3; P2 - P8; P3 - P8
TC1200008127.hg.1	TC1200008127.hg.1	cheeno.aAug10-unspliced	cheeno	NA	20.266	1.79E-05	4.7467	2.52E-05	ctrl - P10; ctrl - P11; ctrl - P3; ctrl - P8; P11 - P10; P2 - P10; P3 - P10; P2 - P11; P11 - P8; P2 - P3; P2 - P8
TC2000007158.hg.1	TC2000007158.hg.1	NA	NA	NA	20.198	1.82E-05	4.7391	2.56E-05	ctrl - P10; ctrl - P11; ctrl - P3; ctrl - P8; P2 - P10; P10 - P8; P2 - P11; P3 - P11; P2 - P3; P2 - P8; P3 - P8
TC0100015904.hg.1	TC0100015904.hg.1	ronira.aAug10-unspliced	ronira	NA	20.151	1.85E-05	4.7338	2.59E-05	ctrl - P10; ctrl - P11; ctrl - P3; ctrl - P8; P11 - P10; P2 - P10; P3 - P10; P2 - P11; P2 - P3; P2 - P8; P3 - P8
TC0200015602.hg.1	TC0200015602.hg.1	NA	NA	NA	20.129	1.86E-05	4.7313	2.60E-05	ctrl - P10; ctrl - P11; ctrl - P3; ctrl - P8; P2 - P10; P2 - P11; P11 - P8; P2 - P3; P2 - P8
TC1400007233.hg.1	TC1400007233.hg.1	jybuby.aAug10-unspliced	jybuby	NA	20.094	1.87E-05	4.7274	2.63E-05	ctrl - P10; ctrl - P11; ctrl - P3; ctrl - P8; P2 - P10; P3 - P10; P2 - P11; P11 - P8; P2 - P3; P2 - P8; P3 - P8

TC0400007800.hg.1	TC0400007800.hg.1	nusmuby.aAug10-unspliced	nusmuby	NA	20.086	1.88E-05	4.7265	2.63E-05	ctrl - P10; ctrl - P11; ctrl - P3; ctrl - P8; P11 - P10; P2 - P10; P11 - P3; P11 - P8; P2 - P3; P2 - P8
TC0600010124.hg.1	TC0600010124.hg.1	slajo.aAug10-unspliced	slajo	NA	20.079	1.88E-05	4.7257	2.63E-05	ctrl - P10; ctrl - P11; ctrl - P3; ctrl - P8; P11 - P10; P2 - P10; P2 - P11; P11 - P8; P2 - P3; P2 - P8
TC0500010431.hg.1	TC0500010431.hg.1	rorfler.aAug10-unspliced	rorfler	NA	20.077	1.88E-05	4.7254	2.63E-05	ctrl - P10; ctrl - P11; P2 - ctrl; ctrl - P3; ctrl - P8; P2 - P10; P10 - P8; P2 - P11; P11 - P8; P2 - P3; P2 - P8; P3 - P8
TC0200010538.hg.1	TC0200010538.hg.1	mogubu.aAug10-unspliced	mogubu	NA	20.044	1.90E-05	4.7217	2.65E-05	ctrl - P10; ctrl - P11; ctrl - P3; ctrl - P8; P11 - P10; P2 - P10; P2 - P11; P11 - P3; P11 - P8; P2 - P3; P2 - P8
TC1200011940.hg.1	TC1200011940.hg.1	uc058thw.1	U7	NA	20.017	1.91E-05	4.7187	2.67E-05	ctrl - P10; ctrl - P11; ctrl - P8; P10 - P11; P2 - P10; P10 - P8; P2 - P11; P3 - P11; P2 - P3; P2 - P8; P3 - P8
TC0X00006704.hg.1	TC0X00006704.hg.1	NM_001080975	REPS2	9185	19.997	1.92E-05	4.7164	2.68E-05	P10 - ctrl; P11 - ctrl; P3 - ctrl; P8 - ctrl; P10 - P11; P10 - P2; P3 - P11; P8 - P11; P3 - P2; P8 - P2
TC1100008900.hg.1	TC1100008900.hg.1	NM_001195005	C11orf70	85016	19.982	1.93E-05	4.7147	2.69E-05	ctrl - P10; ctrl - P11; ctrl - P2; ctrl - P3; ctrl - P8; P11 - P10; P2 - P10; P3 - P10; P11 - P8; P2 - P8; P3 - P8
TC0300007650.hg.1	TC0300007650.hg.1	NA	NA	NA	19.928	1.96E-05	4.7085	2.73E-05	ctrl - P10; ctrl - P11; ctrl - P3; ctrl - P8; P2 - P10; P2 - P11; P11 - P8; P2 - P3; P2 - P8; P3 - P8
TC1300007848.hg.1	TC1300007848.hg.1	versharbu.aAug10-unspliced	versharbu	NA	19.928	1.96E-05	4.7085	2.73E-05	ctrl - P10; ctrl - P11; ctrl - P3; ctrl - P8; P11 - P10; P2 - P10; P3 - P10; P11 - P8; P2 - P3; P2 - P8; P3 - P8
TC0100013274.hg.1	TC0100013274.hg.1	meeloybu.aAug10-unspliced	meeloybu	NA	19.925	1.96E-05	4.7082	2.73E-05	ctrl - P10; ctrl - P11; ctrl - P3; ctrl - P8; P2 - P10; P3 - P10; P2 - P11; P11 - P8; P2 - P3; P2 - P8; P3 - P8
TC0900008444.hg.1	TC0900008444.hg.1	NR_039814	MIR4668	1.01E+08	19.898	1.97E-05	4.7051	2.75E-05	ctrl - P10; ctrl - P11; ctrl - P3; ctrl - P8; P2 - P10; P2 - P11; P2 - P3; P2 - P8
TC1700012102.hg.1	TC1700012102.hg.1	jersker.aAug10-unspliced	jersker	NA	19.878	1.98E-05	4.7028	2.76E-05	ctrl - P10; ctrl - P11; P2 - ctrl; ctrl - P3; ctrl - P8; P2 - P10; P2 - P11; P2 - P3; P2 - P8; P3 - P8
TC0600011662.hg.1	TC0600011662.hg.1	snawspuby.aAug10-unspliced	snawspuby	NA	19.878	1.98E-05	4.7028	2.76E-05	ctrl - P10; ctrl - P11; ctrl - P3; ctrl - P8; P2 - P10; P2 - P11; P11 - P8; P2 - P3; P2 - P8
TC0700012837.hg.1	TC0700012837.hg.1	ENST00000602609.1	RP5-894A10.6		19.877	1.98E-05	4.7028	2.76E-05	ctrl - P10; ctrl - P11; ctrl - P3; ctrl - P8; P11 - P10; P2 - P10; P3 - P10; P2 - P11; P11 - P8; P2 - P3; P2 - P8; P3 - P8
TC1100009296.hg.1	TC1100009296.hg.1	rorkarby.bAug10-unspliced	rorkarby	NA	19.862	1.99E-05	4.701	2.77E-05	ctrl - P10; ctrl - P11; ctrl - P3; ctrl - P8; P11 - P10; P2 - P10; P3 - P10; P2 - P11; P11 - P8; P2 - P3; P2 - P8; P3 - P8
TC2100007085.hg.1	TC2100007085.hg.1	dorbaw.aAug10-unspliced	dorbaw	NA	19.802	2.02E-05	4.6942	2.81E-05	ctrl - P10; ctrl - P11; ctrl - P3; ctrl - P8; P2 - P10; P2 - P11; P11 - P8; P2 - P3; P2 - P8; P3 - P8
TC0300011414.hg.1	TC0300011414.hg.1	gunarbo.aAug10-unspliced	gunarbo	NA	19.78	2.03E-05	4.6916	2.82E-05	ctrl - P10; ctrl - P11; ctrl - P3; ctrl - P8; P2 - P10; P2 - P11; P11 - P8; P2 - P3; P2 - P8
TC0200012278.hg.1	TC0200012278.hg.1	ENST00000623854	RP11-314C9.1		19.776	2.04E-05	4.6912	2.83E-05	P11 - ctrl; P3 - ctrl; P11 - P10; P3 - P10; P11 - P2; P11 - P8; P3 - P2; P3 - P8
TC0500008831.hg.1	TC0500008831.hg.1	sleyjy.aAug10-unspliced	sleyjy	NA	19.761	2.04E-05	4.6895	2.83E-05	ctrl - P11; P2 - ctrl; ctrl - P8; P10 - P11; P2 - P10; P2 - P11; P3 - P11; P2 - P3; P2 - P8; P3 - P8
TC1700012284.hg.1	TC1700012284.hg.1	NA	NA	NA	19.748	2.05E-05	4.6879	2.84E-05	ctrl - P10; ctrl - P11; ctrl - P3; ctrl - P8; P11 - P10; P2 - P10; P2 - P11; P11 - P3; P11 - P8; P2 - P3; P2 - P8
TC0200015240.hg.1	TC0200015240.hg.1	NA	NA	NA	19.729	2.06E-05	4.6858	2.86E-05	ctrl - P10; ctrl - P3; ctrl - P8; P11 - P10; P2 - P10; P3 - P10; P11 - P3; P11 - P8; P2 - P3; P2 - P8; P3 - P8
TC0600011060.hg.1	TC0600011060.hg.1	lafloyby.aAug10-unspliced	lafloyby	NA	19.702	2.08E-05	4.6827	2.87E-05	ctrl - P10; ctrl - P11; P2 - ctrl; ctrl - P3; ctrl - P8; P2 - P10; P2 - P11; P2 - P3; P2 - P8
TC1000007525.hg.1	TC1000007525.hg.1	romare.aAug10-unspliced	romare	NA	19.691	2.08E-05	4.6815	2.88E-05	ctrl - P10; ctrl - P11; ctrl - P8; P11 - P10; P2 - P10; P3 - P10; P2 - P11; P3 - P11; P11 - P8; P2 - P8; P3 - P8
TC1100007808.hg.1	TC1100007808.hg.1	hsa_circ_0000312	SDHAF2	NA	19.681	2.09E-05	4.6803	2.89E-05	ctrl - P10; ctrl - P11; P2 - ctrl; ctrl - P8; P2 - P10; P3 - P10; P2 - P11; P11 - P8; P2 - P3; P2 - P8; P3 - P8
TC1000010242.hg.1	TC1000010242.hg.1	gloynorby.aAug10-unspliced	gloynorby	NA	19.678	2.09E-05	4.6799	2.89E-05	ctrl - P10; ctrl - P11; ctrl - P3; ctrl - P8; P2 - P10; P3 - P10; P2 - P11; P11 - P8; P2 - P3; P2 - P8; P3 - P8
TC0600010130.hg.1	TC0600010130.hg.1	kasoya.aAug10-unspliced	kasoya	NA	19.671	2.09E-05	4.6791	2.89E-05	ctrl - P10; ctrl - P11; ctrl - P3; ctrl - P8; P11 - P10; P2 - P10; P3 - P10; P2 - P11; P11 - P8; P2 - P3; P2 - P8; P3 - P8
TC0200013797.hg.1	TC0200013797.hg.1	ENST00000453431	AC009963.3	NA	19.635	2.11E-05	4.675	2.92E-05	ctrl - P10; ctrl - P3; ctrl - P8; P11 - P10; P2 - P10; P11 - P3; P11 - P8; P2 - P3; P2 - P8; P3 - P8
TC0800008830.hg.1	TC0800008830.hg.1	meyskeybu.aAug10-unspliced	meyskeybu	NA	19.618	2.12E-05	4.6731	2.93E-05	P2 - ctrl; P3 - ctrl; P2 - P10; P3 - P10; P2 - P11; P3 - P11; P2 - P8; P3 - P8
TC0100018332.hg.1	TC0100018332.hg.1	NA	NA	NA	19.614	2.13E-05	4.6725	2.93E-05	ctrl - P10; ctrl - P3; ctrl - P8; P11 - P10; P2 - P10; P11 - P3; P11 - P8; P2 - P3; P2 - P8
TC0600006799.hg.1	TC0600006799.hg.1	floyklorby.aAug10-unspliced	floyklorby	NA	19.602	2.13E-05	4.6712	2.94E-05	ctrl - P10; ctrl - P11; P2 - ctrl; ctrl - P3; ctrl - P8; P2 - P10; P2 - P11; P2 - P3; P2 - P8
TC1100011842.hg.1	TC1100011842.hg.1	duchoy.aAug10-unspliced	duchoy	NA	19.593	2.14E-05	4.6702	2.94E-05	ctrl - P10; P2 - ctrl; ctrl - P8; P2 - P10; P3 - P10; P10 - P8; P2 - P11; P11 - P8; P2 - P3; P2 - P8; P3 - P8
TC0200015918.hg.1	TC0200015918.hg.1	NA	NA	NA	19.586	2.14E-05	4.6694	2.95E-05	ctrl - P10; ctrl - P2; ctrl - P3; ctrl - P8; P11 - P10; P2 - P10; P11 - P2; P11 - P3; P11 - P8; P2 - P3; P2 - P8
TC0600008550.hg.1	TC0600008550.hg.1	NA	NA	NA	19.499	2.19E-05	4.6593	3.02E-05	P10 - ctrl; P2 - ctrl; P8 - ctrl; P10 - P11; P10 - P3; P2 - P11; P8 - P11; P2 - P3; P8 - P3

TC0600007233.hg.1	TC0600007233.hg.1	NA	NA	NA	19.495	2.19E-05	4.6589	3.02E-05	ctrl - P10; ctrl - P11; ctrl - P3; ctrl - P8; P2 - P10; P3 - P10; P2 - P11; P2 - P3; P2 - P8; P3 - P8
TC0600010128.hg.1	TC0600010128.hg.1	bawfleeby.aAug10-unspliced	bawfleeby	NA	19.487	2.20E-05	4.6579	3.02E-05	ctrl - P10; ctrl - P11; ctrl - P3; ctrl - P8; P11 - P10; P2 - P10; P2 - P11; P11 - P3; P11 - P8; P2 - P3; P2 - P8; P3 - P8
TC1200010909.hg.1	TC1200010909.hg.1	uc058pnx.1	RNU7-40P	NA	19.464	2.21E-05	4.6552	3.04E-05	ctrl - P10; ctrl - P11; ctrl - P8; P2 - P10; P3 - P10; P2 - P11; P3 - P11; P2 - P8; P3 - P8
TC2000007241.hg.1	TC2000007241.hg.1	joyvoy.aAug10-unspliced	joyvoy	NA	19.453	2.22E-05	4.654	3.05E-05	ctrl - P10; ctrl - P11; ctrl - P2; ctrl - P3; ctrl - P8; P11 - P10; P2 - P10; P3 - P10; P11 - P3; P11 - P8; P2 - P8; P3 - P8
TC0100010594.hg.1	TC0100010594.hg.1	uc057nhr.1	Z98750.1	NA	19.451	2.22E-05	4.6537	3.05E-05	ctrl - P10; ctrl - P11; P2 - ctrl; ctrl - P3; ctrl - P8; P2 - P10; P2 - P11; P11 - P8; P2 - P3; P2 - P8; P3 - P8
TC0100014051.hg.1	TC0100014051.hg.1	NA	NA	NA	19.444	2.22E-05	4.6529	3.05E-05	ctrl - P11; P2 - ctrl; ctrl - P8; P10 - P11; P2 - P10; P2 - P11; P3 - P11; P8 - P11; P2 - P8; P3 - P8
TC1300008293.hg.1	TC1300008293.hg.1	EFHA1.jAug10-unspliced	EFHA1	NA	19.433	2.23E-05	4.6516	3.06E-05	ctrl - P10; ctrl - P11; ctrl - P3; ctrl - P8; P11 - P10; P2 - P10; P2 - P11; P11 - P8; P2 - P3; P2 - P8
TC0100016970.hg.1	TC0100016970.hg.1	ENST00000362601	Y_RNA		19.415	2.24E-05	4.6495	3.07E-05	ctrl - P10; ctrl - P11; ctrl - P3; ctrl - P8; P2 - P10; P3 - P10; P2 - P11; P2 - P3; P2 - P8; P3 - P8
TC0400011825.hg.1	TC0400011825.hg.1	meyku.aAug10-unspliced	meyku	NA	19.384	2.26E-05	4.6459	3.09E-05	ctrl - P10; ctrl - P3; ctrl - P8; P11 - P10; P2 - P10; P3 - P10; P2 - P11; P11 - P8; P2 - P3; P2 - P8; P3 - P8
TC1700006558.hg.1	TC1700006558.hg.1	ENST00000622996	RP11-135N5.3		19.377	2.26E-05	4.6451	3.10E-05	ctrl - P10; ctrl - P11; ctrl - P3; ctrl - P8; P2 - P10; P2 - P11; P11 - P8; P2 - P3; P2 - P8; P3 - P8
TC1700008264.hg.1	TC1700008264.hg.1	blymee.aAug10-unspliced	blymee	NA	19.377	2.26E-05	4.6451	3.10E-05	ctrl - P10; ctrl - P11; ctrl - P3; ctrl - P8; P2 - P10; P2 - P11; P2 - P3; P2 - P8; P3 - P8
TC0100007242.hg.1	TC0100007242.hg.1	warrarbo.aAug10-unspliced	warrarbo	NA	19.354	2.28E-05	4.6424	3.11E-05	ctrl - P10; ctrl - P11; ctrl - P3; ctrl - P8; P11 - P10; P2 - P10; P2 - P11; P11 - P3; P11 - P8; P2 - P3; P2 - P8
TC0200009760.hg.1	TC0200009760.hg.1	loydo.bAug10-unspliced	loydo	NA	19.351	2.28E-05	4.642	3.12E-05	ctrl - P10; ctrl - P3; ctrl - P8; P11 - P10; P2 - P10; P3 - P10; P2 - P11; P11 - P8; P2 - P3; P2 - P8; P3 - P8
TC2200007916.hg.1	TC2200007916.hg.1	ENST00000441167.1	XXbac-B476C20.13		19.347	2.28E-05	4.6416	3.12E-05	ctrl - P10; ctrl - P11; ctrl - P3; ctrl - P8; P2 - P10; P3 - P10; P2 - P11; P11 - P8; P2 - P3; P2 - P8; P3 - P8
TC0700010688.hg.1	TC0700010688.hg.1	neegloyby.aAug10-unspliced	neegloyby	NA	19.342	2.29E-05	4.641	3.12E-05	ctrl - P10; ctrl - P11; ctrl - P3; ctrl - P8; P2 - P10; P10 - P8; P2 - P11; P2 - P3; P2 - P8; P3 - P8
TC0200013564.hg.1	TC0200013564.hg.1	NR_103732_2	LOC100506076		19.323	2.30E-05	4.6388	3.13E-05	ctrl - P10; ctrl - P11; ctrl - P8; P2 - P10; P3 - P10; P2 - P11; P3 - P11; P8 - P11; P2 - P8; P3 - P8
TC1000010833.hg.1	TC1000010833.hg.1	ENST00000435889	RP11-474D14.2	NA	19.306	2.31E-05	4.6368	3.15E-05	ctrl - P10; ctrl - P11; ctrl - P8; P2 - P10; P3 - P10; P10 - P8; P2 - P11; P3 - P11; P11 - P8; P2 - P3; P2 - P8; P3 - P8
TC0600007889.hg.1	TC0600007889.hg.1	flarjaw.aAug10-unspliced	flarjaw	NA	19.276	2.33E-05	4.6333	3.17E-05	ctrl - P10; ctrl - P11; ctrl - P8; P2 - P10; P3 - P10; P2 - P11; P3 - P11; P2 - P3; P2 - P8; P3 - P8
TC1300008559.hg.1	TC1300008559.hg.1	wawcha.aAug10-unspliced	wawcha	NA	19.275	2.33E-05	4.6332	3.17E-05	ctrl - P10; ctrl - P11; ctrl - P3; ctrl - P8; P2 - P10; P10 - P8; P2 - P11; P11 - P8; P2 - P3; P2 - P8; P3 - P8
TC0200010697.hg.1	TC0200010697.hg.1	cherklu.aAug10-unspliced	cherklu	NA	19.243	2.35E-05	4.6295	3.19E-05	ctrl - P10; ctrl - P11; ctrl - P3; ctrl - P8; P11 - P10; P2 - P10; P2 - P11; P11 - P8; P2 - P3; P2 - P8; P3 - P8
TC0300012059.hg.1	TC0300012059.hg.1	yamara.aAug10-unspliced	yamara	NA	19.24	2.35E-05	4.6291	3.19E-05	ctrl - P10; ctrl - P11; P2 - ctrl; ctrl - P8; P2 - P10; P3 - P10; P2 - P11; P3 - P11; P11 - P8; P2 - P3; P2 - P8; P3 - P8
TC0300007138.hg.1	TC0300007138.hg.1	koyfawbo.aAug10-unspliced	koyfawbo	NA	19.239	2.35E-05	4.629	3.19E-05	ctrl - P10; ctrl - P11; ctrl - P8; P11 - P10; P2 - P10; P3 - P10; P2 - P11; P11 - P8; P2 - P3; P2 - P8; P3 - P8
TC1000011301.hg.1	TC1000011301.hg.1	zoyloy.aAug10-unspliced	zoyloy	NA	19.22	2.36E-05	4.6267	3.21E-05	ctrl - P10; ctrl - P11; ctrl - P3; ctrl - P8; P2 - P10; P10 - P8; P2 - P11; P11 - P8; P2 - P3; P2 - P8; P3 - P8
TC0200014855.hg.1	TC0200014855.hg.1	NA	NA	NA	19.194	2.38E-05	4.6237	3.23E-05	ctrl - P10; ctrl - P3; ctrl - P8; P11 - P10; P2 - P10; P3 - P10; P11 - P3; P11 - P8; P2 - P3; P2 - P8
TC0100015491.hg.1	TC0100015491.hg.1	remema.aAug10-unspliced	remema		19.13	2.42E-05	4.6162	3.28E-05	ctrl - P10; ctrl - P11; P2 - ctrl; ctrl - P8; P2 - P10; P3 - P10; P2 - P11; P2 - P3; P2 - P8; P3 - P8
TC0800011580.hg.1	TC0800011580.hg.1	varger.aAug10-unspliced	varger	NA	19.128	2.42E-05	4.616	3.28E-05	ctrl - P10; ctrl - P11; ctrl - P8; P10 - P11; P2 - P10; P10 - P8; P2 - P11; P3 - P11; P2 - P3; P2 - P8; P3 - P8
TC1200012241.hg.1	TC1200012241.hg.1	doyfu.aAug10-unspliced	doyfu	NA	19.118	2.43E-05	4.6147	3.29E-05	ctrl - P10; ctrl - P11; ctrl - P3; ctrl - P8; P2 - P10; P2 - P11; P2 - P3; P2 - P8
TC0200008001.hg.1	TC0200008001.hg.1	zeyklar.aAug10-unspliced	zeyklar	NA	19.097	2.44E-05	4.6123	3.31E-05	ctrl - P10; ctrl - P11; ctrl - P3; ctrl - P8; P11 - P10; P2 - P10; P3 - P10; P2 - P11; P11 - P8; P2 - P3; P2 - P8; P3 - P8
TC0100009447.hg.1	TC0100009447.hg.1	NA	NA	NA	19.078	2.45E-05	4.6101	3.32E-05	ctrl - P10; ctrl - P11; ctrl - P3; ctrl - P8; P10 - P11; P2 - P10; P10 - P8; P2 - P11; P2 - P3; P2 - P8
TC1600008234.hg.1	TC1600008234.hg.1	staglo.aAug10-unspliced	staglo	NA	19.076	2.46E-05	4.6098	3.32E-05	ctrl - P10; ctrl - P11; ctrl - P3; ctrl - P8; P2 - P10; P2 - P11; P11 - P8; P2 - P3; P2 - P8; P3 - P8
TC0100009931.hg.1	TC0100009931.hg.1	gapybu.aAug10-unspliced	gapybu	NA	19.074	2.46E-05	4.6096	3.32E-05	ctrl - P10; ctrl - P11; ctrl - P3; ctrl - P8; P2 - P10; P2 - P11; P2 - P3; P2 - P8
TC0100008931.hg.1	TC0100008931.hg.1	harero.aAug10-unspliced	harero	NA	19.068	2.46E-05	4.6088	3.33E-05	ctrl - P10; P2 - ctrl; ctrl - P8; P11 - P10; P2 - P10; P3 - P10; P2 - P11; P3 - P11; P11 - P8; P2 - P8; P3 - P8
TC0300009731.hg.1	TC0300009731.hg.1	NM_001242314	MAP3K13	9175	19.067	2.46E-05	4.6088	3.33E-05	P10 - ctrl; P11 - ctrl; P3 - ctrl; P8 - ctrl; P10 - P11; P10 - P2; P10 - P3; P11 - P2; P8 - P11; P3 - P2; P8 - P2; P8 - P3

TC0700011487.hg.1	TC0700011487.hg.1	GTF2IRD2P1.bAug10	GTF2IRD2P1		19.055	2.47E-05	4.6074	3.34E-05	ctrl - P10; ctrl - P2; ctrl - P3; ctrl - P8; P11 - P10; P10 - P3; P11 - P2; P11 - P3; P11 - P8; P2 - P3; P2 - P8
TC0300006832.hg.1	TC0300006832.hg.1	spudoybo.aAug10-unspliced	spudoybo	NA	19.052	2.47E-05	4.607	3.34E-05	ctrl - P10; ctrl - P11; P2 - ctrl; ctrl - P8; P11 - P10; P2 - P10; P3 - P10; P2 - P11; P3 - P11; P2 - P3; P2 - P8; P3 - P8
TC1200006688.hg.1	TC1200006688.hg.1	NM_001297698	NANOG	79923	19.05	2.47E-05	4.6067	3.34E-05	ctrl - P10; ctrl - P8; P11 - P10; P2 - P10; P3 - P10; P2 - P11; P11 - P8; P2 - P3; P2 - P8; P3 - P8
TC1000007371.hg.1	TC1000007371.hg.1	skeyglerbu.aAug10-unspliced	skeyglerbu	NA	19.027	2.49E-05	4.604	3.36E-05	P10 - ctrl; ctrl - P11; ctrl - P2; P10 - P11; P10 - P2; P10 - P3; P10 - P8; P3 - P11; P8 - P11; P3 - P2; P8 - P2
TC1200012297.hg.1	TC1200012297.hg.1	ENST00000602601.1	RP11-214K3.20		19.026	2.49E-05	4.6039	3.36E-05	ctrl - P10; ctrl - P11; ctrl - P3; ctrl - P8; P11 - P10; P2 - P10; P3 - P10; P2 - P11; P11 - P8; P2 - P3; P2 - P8; P3 - P8
TC0600012033.hg.1	TC0600012033.hg.1	peyjee.aAug10-unspliced	peyjee	NA	18.966	2.53E-05	4.5968	3.41E-05	ctrl - P10; ctrl - P11; ctrl - P3; ctrl - P8; P11 - P10; P2 - P10; P3 - P10; P2 - P11; P11 - P8; P2 - P3; P2 - P8; P3 - P8
TC0300013069.hg.1	TC0300013069.hg.1	serjawbu.aAug10-unspliced	serjawbu	NA	18.956	2.54E-05	4.5955	3.42E-05	ctrl - P10; P2 - ctrl; ctrl - P3; ctrl - P8; P11 - P10; P2 - P10; P2 - P11; P11 - P8; P2 - P3; P2 - P8; P3 - P8
TC1300007111.hg.1	TC1300007111.hg.1	swoycha.aAug10-unspliced	swoycha	NA	18.933	2.55E-05	4.5928	3.44E-05	ctrl - P10; ctrl - P11; ctrl - P8; P11 - P10; P2 - P10; P3 - P10; P2 - P11; P11 - P8; P2 - P8; P3 - P8
TC0300012057.hg.1	TC0300012057.hg.1	glorbobu.aAug10-unspliced	glorbobu	NA	18.921	2.56E-05	4.5914	3.45E-05	ctrl - P10; ctrl - P11; ctrl - P8; P2 - P10; P3 - P10; P2 - P11; P11 - P8; P2 - P3; P2 - P8; P3 - P8
TC0600009965.hg.1	TC0600009965.hg.1	storjee.aAug10-unspliced	storjee	NA	18.889	2.58E-05	4.5876	3.47E-05	ctrl - P10; ctrl - P11; P2 - ctrl; ctrl - P8; P2 - P10; P3 - P10; P2 - P11; P11 - P8; P2 - P3; P2 - P8; P3 - P8
TC2200007424.hg.1	TC2200007424.hg.1	tawtey.aAug10-unspliced	tawtey	NA	18.739	2.69E-05	4.5697	3.62E-05	ctrl - P10; ctrl - P11; ctrl - P3; ctrl - P8; P2 - P10; P2 - P11; P11 - P8; P2 - P3; P2 - P8
TC0200011265.hg.1	TC0200011265.hg.1	NA	NA	NA	18.736	2.70E-05	4.5694	3.62E-05	ctrl - P10; ctrl - P11; ctrl - P3; ctrl - P8; P11 - P10; P2 - P10; P3 - P10; P11 - P3; P11 - P8; P2 - P3; P2 - P8
TC1100011840.hg.1	TC1100011840.hg.1	makami.aAug10-unspliced	makami	NA	18.669	2.75E-05	4.5613	3.69E-05	ctrl - P10; ctrl - P11; ctrl - P3; ctrl - P8; P2 - P10; P2 - P11; P2 - P3; P2 - P8; P3 - P8
TC0600011171.hg.1	TC0600011171.hg.1	bysnoybu.aAug10-unspliced	bysnoybu	NA	18.664	2.75E-05	4.5607	3.69E-05	ctrl - P10; ctrl - P11; ctrl - P3; ctrl - P8; P11 - P10; P2 - P10; P2 - P11; P11 - P3; P11 - P8; P2 - P3; P2 - P8; P3 - P8
TC1700007095.hg.1	TC1700007095.hg.1	NM_001130090	DRC3	83450	18.647	2.76E-05	4.5586	3.70E-05	ctrl - P10; ctrl - P2; ctrl - P3; ctrl - P8; P11 - P10; P10 - P3; P11 - P2; P11 - P3; P11 - P8; P2 - P3; P8 - P3
TC0200012253.hg.1	TC0200012253.hg.1	uc061icv.1	RNU6-577P	NA	18.646	2.76E-05	4.5585	3.70E-05	ctrl - P10; ctrl - P11; ctrl - P3; ctrl - P8; P11 - P10; P2 - P10; P3 - P10; P2 - P11; P11 - P8; P2 - P3; P2 - P8; P3 - P8
TC1000009714.hg.1	TC1000009714.hg.1	NM_001242413	PRKCQ	5588	18.578	2.82E-05	4.5502	3.77E-05	ctrl - P10; ctrl - P2; ctrl - P3; ctrl - P8; P11 - P10; P2 - P10; P3 - P10; P11 - P2; P11 - P8; P2 - P8; P3 - P8
TC1100010270.hg.1	TC1100010270.hg.1	ENST00000538408	RP11-613F22.6	NA	18.565	2.83E-05	4.5488	3.78E-05	ctrl - P10; ctrl - P11; ctrl - P3; ctrl - P8; P11 - P10; P2 - P10; P2 - P11; P11 - P8; P2 - P3; P2 - P8
TC0700012616.hg.1	TC0700012616.hg.1	flybler.aAug10-unspliced	flybler	NA	18.535	2.85E-05	4.5451	3.81E-05	ctrl - P10; ctrl - P11; ctrl - P8; P10 - P11; P2 - P10; P3 - P10; P10 - P8; P2 - P11; P3 - P11; P2 - P8; P3 - P8
TC0100013551.hg.1	TC0100013551.hg.1	gleeplo.aAug10-unspliced	gleeplo	NA	18.513	2.87E-05	4.5424	3.84E-05	ctrl - P10; ctrl - P11; ctrl - P3; ctrl - P8; P2 - P10; P2 - P11; P2 - P3; P2 - P8; P3 - P8
TC0200015877.hg.1	TC0200015877.hg.1	verdee.aAug10-unspliced	verdee	NA	18.507	2.87E-05	4.5417	3.84E-05	P2 - ctrl; P3 - ctrl; P2 - P10; P3 - P10; P2 - P11; P3 - P11; P3 - P2; P2 - P8; P3 - P8
TC1000008720.hg.1	TC1000008720.hg.1	pykleebu.aAug10-unspliced	pykleebu	NA	18.423	2.94E-05	4.5314	3.93E-05	ctrl - P10; ctrl - P11; ctrl - P8; P2 - P10; P3 - P10; P2 - P11; P11 - P8; P2 - P3; P2 - P8; P3 - P8
TC1400008411.hg.1	TC1400008411.hg.1	ENST00000602827.1	RP11-73M18.6		18.421	2.94E-05	4.5312	3.93E-05	ctrl - P10; ctrl - P11; ctrl - P3; ctrl - P8; P2 - P10; P2 - P11; P3 - P11; P2 - P3; P2 - P8; P3 - P8
TC0200015316.hg.1	TC0200015316.hg.1	sihomo.aAug10-unspliced	sihomo	NA	18.4	2.96E-05	4.5287	3.95E-05	ctrl - P10; ctrl - P11; ctrl - P3; ctrl - P8; P11 - P10; P2 - P10; P3 - P10; P2 - P11; P11 - P8; P2 - P3; P2 - P8; P3 - P8
TC0X00008877.hg.1	TC0X00008877.hg.1	ENST00000408676	MIR1184-3	1E+08	18.377	2.98E-05	4.5258	3.97E-05	P10 - ctrl; P11 - ctrl; ctrl - P2; P8 - ctrl; P10 - P2; P10 - P3; P11 - P2; P11 - P3; P3 - P2; P8 - P2; P8 - P3
TC0100014206.hg.1	TC0100014206.hg.1	chaplo.aAug10-unspliced	chaplo	NA	18.374	2.98E-05	4.5255	3.97E-05	ctrl - P10; P2 - ctrl; ctrl - P3; ctrl - P8; P11 - P10; P2 - P10; P2 - P11; P11 - P8; P2 - P3; P2 - P8
TC0400007881.hg.1	TC0400007881.hg.1	klustybu.aAug10-unspliced	klustybu	NA	18.362	2.99E-05	4.524	3.99E-05	ctrl - P10; ctrl - P11; ctrl - P3; ctrl - P8; P2 - P10; P3 - P10; P2 - P11; P3 - P11; P2 - P8; P3 - P8
TC0200011668.hg.1	TC0200011668.hg.1	ENST00000569008.1	RP11-434B12.1		18.36	2.99E-05	4.5239	3.99E-05	ctrl - P10; ctrl - P11; ctrl - P3; ctrl - P8; P11 - P10; P2 - P10; P11 - P3; P11 - P8; P2 - P3; P2 - P8
TC1000009279.hg.1	TC1000009279.hg.1	steeruby.aAug10-unspliced	steeruby	NA	18.284	3.06E-05	4.5145	4.07E-05	ctrl - P10; ctrl - P11; ctrl - P3; ctrl - P8; P2 - P10; P10 - P8; P2 - P11; P11 - P8; P2 - P3; P2 - P8; P3 - P8
TC0900007814.hg.1	TC0900007814.hg.1	vorteyby.aAug10-unspliced	vorteyby	NA	18.263	3.08E-05	4.5119	4.09E-05	ctrl - P10; ctrl - P11; ctrl - P3; ctrl - P8; P2 - P10; P2 - P11; P11 - P8; P2 - P3; P2 - P8
TC0400010129.hg.1	TC0400010129.hg.1	NA	NA	NA	18.24	3.10E-05	4.5091	4.12E-05	ctrl - P10; ctrl - P11; ctrl - P3; ctrl - P8; P11 - P10; P2 - P10; P2 - P11; P11 - P8; P2 - P3; P2 - P8; P3 - P8
TC0400008025.hg.1	TC0400008025.hg.1	NR_015359	WDFY3-AS2	404201	18.198	3.13E-05	4.504	4.16E-05	ctrl - P10; ctrl - P2; ctrl - P3; ctrl - P8; P11 - P10; P2 - P10; P11 - P3; P11 - P8; P2 - P3; P2 - P8
TC0100007366.hg.1	TC0100007366.hg.1	jawrubu.aAug10-unspliced	jawrubu	NA	18.168	3.16E-05	4.5003	4.20E-05	ctrl - P10; ctrl - P11; ctrl - P3; ctrl - P8; P2 - P10; P3 - P10; P2 - P11; P3 - P11; P2 - P3; P2 - P8; P3 - P8

TC1500007794.hg.1	TC1500007794.hg.1	chaper.aAug10-unspliced	chaper	NA	18.155	3.17E-05	4.4987	4.21E-05	ctrl - P10; ctrl - P11; ctrl - P3; ctrl - P8; P2 - P10; P2 - P11; P2 - P3; P2 - P8; P3 - P8
TC1300008085.hg.1	TC1300008085.hg.1	shupa.aAug10-unspliced	shupa	NA	18.153	3.17E-05	4.4984	4.21E-05	ctrl - P10; ctrl - P11; ctrl - P3; ctrl - P8; P10 - P11; P2 - P10; P10 - P3; P10 - P8; P2 - P11; P2 - P3; P2 - P8
TC0700007850.hg.1	TC0700007850.hg.1	NA	NA	NA	18.079	3.24E-05	4.4893	4.30E-05	P10 - ctrl; P11 - ctrl; P3 - ctrl; P8 - ctrl; P10 - P2; P10 - P3; P11 - P2; P11 - P3; P3 - P2; P8 - P2
TC0800011413.hg.1	TC0800011413.hg.1	NA	NA	NA	18.062	3.26E-05	4.4872	4.32E-05	ctrl - P10; P2 - ctrl; ctrl - P3; ctrl - P8; P11 - P10; P2 - P10; P10 - P8; P11 - P3; P11 - P8; P2 - P3; P2 - P8; P3 - P8
TC0600007112.hg.1	TC0600007112.hg.1	NR_030312	MIR548A1	693125	18.001	3.31E-05	4.4797	4.39E-05	P10 - ctrl; P2 - ctrl; P3 - ctrl; P8 - ctrl; P10 - P11; P10 - P2; P10 - P8; P2 - P11; P3 - P11; P8 - P11; P3 - P2; P3 - P8
TC0200011267.hg.1	TC0200011267.hg.1	mihi.aAug10-unspliced	mihi	NA	17.991	3.32E-05	4.4783	4.40E-05	ctrl - P10; ctrl - P11; ctrl - P3; ctrl - P8; P11 - P10; P2 - P10; P3 - P10; P2 - P11; P11 - P8; P2 - P8; P3 - P8
TC1200007457.hg.1	TC1200007457.hg.1	smorflu.aAug10-unspliced	smorflu	NA	17.959	3.35E-05	4.4744	4.44E-05	ctrl - P10; P2 - ctrl; ctrl - P8; P11 - P10; P2 - P10; P3 - P10; P2 - P11; P11 - P8; P2 - P3; P2 - P8; P3 - P8
TC0700007058.hg.1	TC0700007058.hg.1	NR_036482	DPY19L2P3	442524	17.95	3.36E-05	4.4733	4.45E-05	ctrl - P10; ctrl - P3; ctrl - P8; P11 - P10; P2 - P10; P11 - P3; P11 - P8; P2 - P3; P2 - P8
TC0100011700.hg.1	TC0100011700.hg.1	jernobu.aAug10-unspliced	jernobu	NA	17.947	3.37E-05	4.4729	4.45E-05	ctrl - P10; ctrl - P11; P2 - ctrl; ctrl - P8; P2 - P10; P2 - P11; P11 - P8; P2 - P3; P2 - P8; P3 - P8
TC0X00010780.hg.1	TC0X00010780.hg.1	studo.aAug10-unspliced	studo	NA	17.904	3.41E-05	4.4676	4.50E-05	ctrl - P10; ctrl - P11; P2 - ctrl; ctrl - P8; P2 - P10; P3 - P10; P2 - P11; P3 - P11; P2 - P3; P2 - P8; P3 - P8
TC2000008984.hg.1	TC2000008984.hg.1	jyseebu.aAug10-unspliced	jyseebu	NA	17.902	3.41E-05	4.4673	4.50E-05	ctrl - P10; ctrl - P11; ctrl - P3; ctrl - P8; P2 - P10; P2 - P11; P2 - P3; P2 - P8
TC0600006796.hg.1	TC0600006796.hg.1	dorjoy.aAug10-unspliced	dorjoy	NA	17.9	3.41E-05	4.467	4.50E-05	ctrl - P10; ctrl - P11; ctrl - P3; ctrl - P8; P2 - P10; P2 - P11; P2 - P3; P2 - P8; P3 - P8
TC1100007248.hg.1	TC1100007248.hg.1	spoymaw.aAug10-unspliced	spoymaw	NA	17.872	3.44E-05	4.4636	4.53E-05	ctrl - P10; ctrl - P11; ctrl - P3; ctrl - P8; P10 - P11; P2 - P10; P10 - P8; P2 - P11; P3 - P11; P2 - P3; P2 - P8
TC0200015397.hg.1	TC0200015397.hg.1	NM_001162407	CLK1	1195	17.869	3.44E-05	4.4631	4.54E-05	ctrl - P10; ctrl - P2; ctrl - P3; ctrl - P8; P11 - P10; P2 - P10; P11 - P3; P11 - P8; P2 - P3; P2 - P8
TC1400010335.hg.1	TC1400010335.hg.1	sworshubu.aAug10-unspliced	sworshubu	NA	17.85	3.46E-05	4.4609	4.56E-05	ctrl - P10; ctrl - P11; ctrl - P3; ctrl - P8; P2 - P10; P3 - P10; P2 - P11; P11 - P8; P2 - P3; P2 - P8; P3 - P8
TC1300008555.hg.1	TC1300008555.hg.1	goyvawby.aAug10-unspliced	goyvawby	NA	17.824	3.49E-05	4.4576	4.59E-05	ctrl - P10; ctrl - P11; ctrl - P3; ctrl - P8; P2 - P10; P2 - P11; P2 - P3; P2 - P8
TC1900008256.hg.1	TC1900008256.hg.1	NA	NA	NA	17.795	3.52E-05	4.4539	4.63E-05	ctrl - P10; ctrl - P3; ctrl - P8; P11 - P10; P2 - P10; P2 - P11; P11 - P3; P11 - P8; P2 - P3; P2 - P8
TC1700008067.hg.1	TC1700008067.hg.1	ENST00000587960.1	RP11-798G7.8		17.78	3.53E-05	4.452	4.65E-05	ctrl - P10; ctrl - P11; ctrl - P3; ctrl - P8; P11 - P10; P2 - P10; P3 - P10; P2 - P11; P11 - P8; P2 - P3; P2 - P8; P3 - P8
TC1500009811.hg.1	TC1500009811.hg.1	veestee.aAug10-unspliced	veestee	NA	17.712	3.60E-05	4.4435	4.73E-05	ctrl - P10; ctrl - P11; P2 - ctrl; ctrl - P3; ctrl - P8; P2 - P10; P10 - P8; P2 - P11; P2 - P3; P2 - P8; P3 - P8
TC1800008897.hg.1	TC1800008897.hg.1	moni.aAug10-unspliced	moni	NA	17.694	3.62E-05	4.4412	4.76E-05	ctrl - P10; ctrl - P11; ctrl - P3; ctrl - P8; P2 - P10; P10 - P8; P2 - P11; P2 - P3; P2 - P8
TC1800007419.hg.1	TC1800007419.hg.1	volo.aAug10-unspliced	volo	NA	17.63	3.69E-05	4.4332	4.84E-05	ctrl - P10; ctrl - P11; ctrl - P3; ctrl - P8; P2 - P10; P2 - P11; P2 - P3; P2 - P8
HTA2-pos-47424481_st	23150857	NA	NA	NA	17.62	3.70E-05	4.4319	4.86E-05	ctrl - P10; ctrl - P11; ctrl - P3; ctrl - P8; P11 - P10; P2 - P10; P2 - P11; P2 - P3; P2 - P8
TC1400009030.hg.1	TC1400009030.hg.1	ENST00000557067.1	CTD-2298J14.2		17.613	3.71E-05	4.431	4.86E-05	ctrl - P10; ctrl - P3; ctrl - P8; P11 - P10; P2 - P10; P11 - P2; P11 - P3; P11 - P8; P2 - P3; P2 - P8
TC1800006905.hg.1	TC1800006905.hg.1	NM_001308231	CABYR	26256	17.611	3.71E-05	4.4307	4.86E-05	P10 - ctrl; P2 - ctrl; P3 - ctrl; P8 - ctrl; P10 - P11; P10 - P2; P2 - P11; P3 - P11; P8 - P11; P8 - P2; P8 - P3
TC0100018367.hg.1	TC0100018367.hg.1	NM_000740	CHRM3	1131	17.608	3.71E-05	4.4304	4.86E-05	ctrl - P10; ctrl - P3; ctrl - P8; P11 - P10; P2 - P10; P3 - P10; P11 - P3; P11 - P8; P2 - P8; P3 - P8
TC1700012218.hg.1	TC1700012218.hg.1	NM_015077	SARM1	23098	17.599	3.72E-05	4.4293	4.87E-05	ctrl - P10; P11 - ctrl; ctrl - P2; ctrl - P8; P11 - P10; P3 - P10; P11 - P2; P11 - P3; P11 - P8; P3 - P8
TC0600007901.hg.1	TC0600007901.hg.1	sneykleebu.aAug10-unspliced	sneykleebu	NA	17.58	3.74E-05	4.4268	4.90E-05	ctrl - P10; ctrl - P11; ctrl - P8; P2 - P10; P3 - P10; P2 - P11; P3 - P11; P11 - P8; P2 - P3; P2 - P8; P3 - P8
TC1200006452.hg.1	TC1200006452.hg.1	ENST00000408512	RNU4ATAC16P	NA	17.558	3.77E-05	4.4241	4.93E-05	ctrl - P10; ctrl - P11; ctrl - P3; ctrl - P8; P2 - P10; P3 - P10; P2 - P11; P3 - P11; P2 - P3; P2 - P8; P3 - P8
TC0700010898.hg.1	TC0700010898.hg.1	veeku.aAug10-unspliced	veeku	NA	17.409	3.94E-05	4.405	5.15E-05	ctrl - P10; ctrl - P11; ctrl - P3; ctrl - P8; P11 - P10; P2 - P10; P2 - P11; P11 - P8; P2 - P3; P2 - P8
TC1500009262.hg.1	TC1500009262.hg.1	hekiru.aAug10-unspliced	hekiru	NA	17.402	3.94E-05	4.4042	5.15E-05	ctrl - P10; ctrl - P11; ctrl - P3; ctrl - P8; P2 - P10; P2 - P11; P11 - P8; P2 - P3; P2 - P8
TC0100018249.hg.1	TC0100018249.hg.1	NM_020978	AMY2B	280	17.395	3.95E-05	4.4032	5.16E-05	ctrl - P10; ctrl - P11; ctrl - P3; ctrl - P8; P2 - P10; P2 - P11; P11 - P3; P11 - P8; P2 - P3; P2 - P8
TC0100013500.hg.1	TC0100013500.hg.1	steypeybo.aAug10-unspliced	steypeybo	NA	17.394	3.95E-05	4.4031	5.16E-05	ctrl - P10; ctrl - P11; ctrl - P3; ctrl - P8; P2 - P10; P2 - P11; P2 - P3; P2 - P8; P3 - P8
TC0800011541.hg.1	TC0800011541.hg.1	klarverby.aAug10-unspliced	klarverby	NA	17.369	3.98E-05	4.4	5.20E-05	ctrl - P10; ctrl - P11; P2 - ctrl; ctrl - P8; P2 - P10; P3 - P10; P2 - P11; P3 - P11; P11 - P8; P2 - P3; P2 - P8; P3 - P8
TC0100008272.hg.1	TC0100008272.hg.1	snoboy.aAug10-unspliced	snoboy	NA	17.351	4.00E-05	4.3977	5.22E-05	ctrl - P10; ctrl - P11; ctrl - P3; ctrl - P8; P2 - P10; P3 - P10; P2 - P11; P3 - P11; P2 - P3; P2 - P8; P3 - P8

TC0600010132.hg.1	TC0600010132.hg.1	wusmoyby.aAug10-unspliced	wusmoyby	NA	17.334	4.02E-05	4.3954	5.24E-05	ctrl - P10; ctrl - P11; ctrl - P3; ctrl - P8; P2 - P10; P3 - P10; P2 - P11; P11 - P8; P2 - P3; P2 - P8; P3 - P8
TC1700008229.hg.1	TC1700008229.hg.1	smyslee.aAug10-unspliced	smyslee	NA	17.305	4.06E-05	4.3917	5.29E-05	ctrl - P10; ctrl - P11; ctrl - P8; P2 - P10; P3 - P10; P2 - P11; P3 - P11; P3 - P2; P2 - P8; P3 - P8
TC0300006720.hg.1	TC0300006720.hg.1	NA	NA	NA	17.302	4.06E-05	4.3914	5.29E-05	ctrl - P10; ctrl - P2; ctrl - P3; ctrl - P8; P11 - P10; P2 - P10; P11 - P2; P11 - P3; P11 - P8; P2 - P3
TC0300013113.hg.1	TC0300013113.hg.1	borkybu.aAug10-unspliced	borkybu	NA	17.298	4.07E-05	4.3908	5.29E-05	ctrl - P10; ctrl - P11; P2 - ctrl; P3 - ctrl; ctrl - P8; P2 - P10; P3 - P10; P2 - P11; P3 - P11; P2 - P8; P3 - P8
TC0300006943.hg.1	TC0300006943.hg.1	sworjeebo.aAug10-unspliced	sworjeebo	NA	17.251	4.12E-05	4.3848	5.36E-05	ctrl - P10; ctrl - P11; ctrl - P3; ctrl - P8; P2 - P10; P10 - P8; P2 - P11; P11 - P8; P2 - P3; P2 - P8; P3 - P8
TC0800011538.hg.1	TC0800011538.hg.1	klyverby.aAug10-unspliced	klyverby	NA	17.206	4.18E-05	4.379	5.43E-05	ctrl - P10; ctrl - P11; P2 - ctrl; ctrl - P8; P2 - P10; P2 - P11; P11 - P8; P2 - P3; P2 - P8; P3 - P8
TC1300006758.hg.1	TC1300006758.hg.1	NA	NA	NA	17.196	4.19E-05	4.3778	5.45E-05	ctrl - P10; ctrl - P11; ctrl - P3; ctrl - P8; P2 - P10; P3 - P10; P2 - P11; P2 - P3; P2 - P8
TC1200007898.hg.1	TC1200007898.hg.1	NA	NA	NA	17.181	4.21E-05	4.3758	5.47E-05	P10 - ctrl; P11 - ctrl; P8 - ctrl; P10 - P2; P10 - P3; P11 - P2; P11 - P3; P8 - P2; P8 - P3
TC0600013731.hg.1	TC0600013731.hg.1	beeflawby.aAug10-unspliced	beeflawby	NA	17.124	4.28E-05	4.3684	5.56E-05	ctrl - P10; ctrl - P11; ctrl - P8; P2 - P10; P3 - P10; P2 - P11; P3 - P11; P2 - P8; P3 - P8
TC0200016511.hg.1	TC0200016511.hg.1	NM_000577	IL1RN	3557	17.099	4.31E-05	4.3651	5.60E-05	P10 - ctrl; P3 - ctrl; P8 - ctrl; P10 - P11; P10 - P2; P8 - P10; P8 - P11; P3 - P2; P8 - P2; P8 - P3
TC1100007235.hg.1	TC1100007235.hg.1	NA	NA	NA	17.09	4.32E-05	4.3641	5.61E-05	P10 - ctrl; P11 - ctrl; P3 - ctrl; P8 - ctrl; P10 - P2; P11 - P2; P3 - P2; P8 - P2
TC0500011650.hg.1	TC0500011650.hg.1	chawsloby.aAug10-unspliced	chawsloby	NA	17.061	4.36E-05	4.3602	5.66E-05	ctrl - P11; P2 - ctrl; ctrl - P8; P10 - P11; P2 - P10; P3 - P10; P10 - P8; P2 - P11; P3 - P11; P2 - P8; P3 - P8
TC0X00007080.hg.1	TC0X00007080.hg.1	uc064ysa.1	AL138744.1	NA	17.009	4.43E-05	4.3535	5.74E-05	ctrl - P10; ctrl - P11; ctrl - P3; ctrl - P8; P2 - P10; P2 - P11; P2 - P3; P2 - P8
TC0600009997.hg.1	TC0600009997.hg.1	barjey.aAug10-unspliced	barjey	NA	16.98	4.47E-05	4.3497	5.79E-05	ctrl - P10; ctrl - P11; P2 - ctrl; ctrl - P8; P2 - P10; P10 - P8; P2 - P11; P3 - P11; P2 - P3; P2 - P8; P3 - P8
TC1500009183.hg.1	TC1500009183.hg.1	flyzawbu.aAug10-unspliced	flyzawbu	NA	16.963	4.49E-05	4.3475	5.81E-05	ctrl - P10; ctrl - P11; ctrl - P3; ctrl - P8; P2 - P10; P2 - P11; P2 - P3; P2 - P8
TC0500011343.hg.1	TC0500011343.hg.1	blagey.aAug10	blagey	NA	16.944	4.52E-05	4.345	5.84E-05	P10 - ctrl; P2 - ctrl; P3 - ctrl; P8 - ctrl; P10 - P11; P2 - P11; P3 - P11; P8 - P11
TC0600009145.hg.1	TC0600009145.hg.1	ENST00000444259.1	RP5-1112D6.4		16.931	4.54E-05	4.3433	5.86E-05	ctrl - P10; ctrl - P11; ctrl - P8; P2 - P10; P3 - P10; P2 - P11; P3 - P11; P2 - P8; P3 - P8
TC0200014176.hg.1	TC0200014176.hg.1	NA	NA	NA	16.924	4.55E-05	4.3423	5.87E-05	ctrl - P10; ctrl - P3; ctrl - P8; P11 - P10; P2 - P10; P3 - P10; P11 - P8; P2 - P8; P3 - P8
HTA2-neg-47419569_st	23149578	NA	NA	NA	16.921	4.55E-05	4.3419	5.88E-05	ctrl - P10; ctrl - P11; ctrl - P3; ctrl - P8; P2 - P10; P10 - P8; P2 - P11; P11 - P8; P2 - P3; P2 - P8; P3 - P8
TC0500010774.hg.1	TC0500010774.hg.1	NR_049869	MIR5687	1.01E+08	16.9	4.58E-05	4.3393	5.91E-05	ctrl - P10; ctrl - P11; P2 - ctrl; ctrl - P3; ctrl - P8; P2 - P10; P3 - P10; P2 - P11; P2 - P3; P2 - P8
TC1200012275.hg.1	TC1200012275.hg.1	zyblu.aAug10-unspliced	zyblu	NA	16.876	4.61E-05	4.3361	5.95E-05	ctrl - P10; ctrl - P11; ctrl - P3; ctrl - P8; P2 - P10; P3 - P10; P2 - P11; P2 - P3; P2 - P8; P3 - P8
TC1200007552.hg.1	TC1200007552.hg.1	ENST00000547387.1	RP11-386G11.10		16.862	4.63E-05	4.3342	5.97E-05	P10 - ctrl; P11 - ctrl; P3 - ctrl; P8 - ctrl; P10 - P11; P10 - P2; P11 - P2; P8 - P11; P3 - P2; P8 - P2
TC1700012361.hg.1	TC1700012361.hg.1	NM_001282540	CDRT1	374286	16.85	4.65E-05	4.3327	5.99E-05	ctrl - P11; P3 - ctrl; P8 - ctrl; P10 - P11; P10 - P2; P8 - P10; P3 - P11; P8 - P11; P3 - P2; P8 - P2
TC0300011412.hg.1	TC0300011412.hg.1	banarbo.aAug10-unspliced	banarbo	NA	16.85	4.65E-05	4.3326	5.99E-05	ctrl - P10; ctrl - P11; ctrl - P3; ctrl - P8; P2 - P10; P2 - P11; P11 - P8; P2 - P3; P2 - P8; P3 - P8
TC0500010844.hg.1	TC0500010844.hg.1	juflu.aAug10-unspliced	juflu	NA	16.843	4.66E-05	4.3318	6.00E-05	ctrl - P10; ctrl - P11; ctrl - P3; ctrl - P8; P11 - P10; P2 - P10; P3 - P10; P11 - P8; P2 - P8; P3 - P8
TC0700008851.hg.1	TC0700008851.hg.1	yukemu.aAug10-unspliced	yukemu	NA	16.813	4.70E-05	4.3278	6.05E-05	P2 - ctrl; P3 - ctrl; ctrl - P8; P11 - P10; P2 - P10; P3 - P10; P2 - P11; P11 - P8; P2 - P3; P2 - P8; P3 - P8
TC0500010846.hg.1	TC0500010846.hg.1	jawflu.aAug10-unspliced	jawflu	NA	16.793	4.73E-05	4.3251	6.08E-05	ctrl - P10; ctrl - P3; ctrl - P8; P11 - P10; P2 - P10; P3 - P10; P11 - P8; P2 - P8; P3 - P8
TC0100007216.hg.1	TC0100007216.hg.1	nokumo.aAug10-unspliced	nokumo	NA	16.741	4.80E-05	4.3183	6.18E-05	ctrl - P10; P2 - ctrl; ctrl - P3; ctrl - P8; P11 - P10; P2 - P10; P2 - P11; P11 - P3; P11 - P8; P2 - P3; P2 - P8
TC0100017311.hg.1	TC0100017311.hg.1	rerrerbo.aAug10-unspliced	rerrerbo	NA	16.698	4.87E-05	4.3126	6.26E-05	ctrl - P10; ctrl - P2; ctrl - P3; ctrl - P8; P11 - P10; P2 - P10; P11 - P3; P11 - P8; P2 - P3; P2 - P8
TC0X00006816.hg.1	TC0X00006816.hg.1	NM_001142386	PDK3	5165	16.693	4.88E-05	4.312	6.26E-05	ctrl - P10; ctrl - P2; ctrl - P8; P11 - P10; P2 - P10; P3 - P10; P11 - P2; P11 - P8; P3 - P2; P3 - P8
TC0800007316.hg.1	TC0800007316.hg.1	NM_004095	EIF4EBP1	1978	16.58	5.05E-05	4.297	6.48E-05	P10 - ctrl; P11 - ctrl; P3 - ctrl; P8 - ctrl; P11 - P10; P10 - P2; P8 - P10; P11 - P2; P11 - P3; P3 - P2; P8 - P2; P8 - P3
TC1500009205.hg.1	TC1500009205.hg.1	skoysu.aAug10-unspliced	skoysu	NA	16.424	5.30E-05	4.2761	6.79E-05	ctrl - P10; ctrl - P11; ctrl - P3; ctrl - P8; P2 - P10; P10 - P8; P2 - P11; P11 - P8; P2 - P8; P3 - P8
TC0500011032.hg.1	TC0500011032.hg.1	rani.aAug10-unspliced	rani	NA	16.414	5.31E-05	4.2748	6.81E-05	ctrl - P10; ctrl - P11; ctrl - P3; ctrl - P8; P11 - P10; P2 - P10; P2 - P11; P11 - P8; P2 - P3; P2 - P8; P3 - P8
TC1200007716.hg.1	TC1200007716.hg.1	veyjoyby.aAug10-unspliced	veyjoyby	NA	16.412	5.32E-05	4.2745	6.81E-05	ctrl - P10; ctrl - P11; ctrl - P3; ctrl - P8; P2 - P10; P2 - P11; P11 - P8; P2 - P3; P2 - P8; P3 - P8

TC0800010265.hg.1	TC0800010265.hg.1	glerkor.aAug10-unspliced	glerkor	NA	16.411	5.32E-05	4.2743	6.81E-05	ctrl - P10; ctrl - P11; P2 - ctrl; ctrl - P8; P2 - P10; P2 - P11; P11 - P8; P2 - P3; P2 - P8; P3 - P8
TC1100007461.hg.1	TC1100007461.hg.1	hsa_circ_0000297	ARHGAP1	NA	16.371	5.38E-05	4.269	6.89E-05	P10 - ctrl; P11 - ctrl; P2 - ctrl; P3 - ctrl; P8 - ctrl; P10 - P11; P10 - P2; P10 - P3; P11 - P2; P8 - P2; P8 - P3
TC1800006632.hg.1	TC1800006632.hg.1	ENST00000609701.1	RP11-888D10.4		16.342	5.43E-05	4.2651	6.95E-05	ctrl - P10; ctrl - P11; ctrl - P3; ctrl - P8; P11 - P10; P2 - P10; P3 - P10; P2 - P11; P11 - P8; P2 - P3; P2 - P8
TC0900011287.hg.1	TC0900011287.hg.1	NA	NA	NA	16.34	5.44E-05	4.2648	6.95E-05	ctrl - P10; ctrl - P11; ctrl - P3; ctrl - P8; P11 - P10; P2 - P10; P11 - P8; P2 - P3; P2 - P8
TC1700010948.hg.1	TC1700010948.hg.1	NA	NA	NA	16.331	5.45E-05	4.2636	6.97E-05	ctrl - P10; P2 - ctrl; ctrl - P3; ctrl - P8; P11 - P10; P2 - P10; P2 - P11; P11 - P3; P11 - P8; P2 - P3; P2 - P8
TC1200006506.hg.1	TC1200006506.hg.1	NA	NA	NA	16.317	5.47E-05	4.2617	6.99E-05	ctrl - P10; ctrl - P3; ctrl - P8; P11 - P10; P2 - P10; P11 - P3; P11 - P8; P2 - P3; P2 - P8
TC1400008696.hg.1	TC1400008696.hg.1	glorslu.aAug10-unspliced	glorslu	NA	16.26	5.57E-05	4.254	7.11E-05	ctrl - P10; ctrl - P11; ctrl - P3; ctrl - P8; P2 - P10; P3 - P10; P2 - P11; P2 - P3; P2 - P8; P3 - P8
TC0300011204.hg.1	TC0300011204.hg.1	bomoybo.aAug10-unspliced	bomoybo	NA	16.237	5.61E-05	4.251	7.16E-05	ctrl - P10; ctrl - P11; ctrl - P8; P11 - P10; P2 - P10; P3 - P10; P2 - P11; P2 - P8; P3 - P8
TC0600011974.hg.1	TC0600011974.hg.1	slaglawby.aAug10-unspliced	slaglawby	NA	16.171	5.73E-05	4.242	7.31E-05	P2 - ctrl; P3 - ctrl; P10 - P11; P2 - P10; P3 - P10; P2 - P11; P3 - P11; P8 - P11; P2 - P8; P3 - P8
TC0200013801.hg.1	TC0200013801.hg.1	NR_131251	LINC01594	1.03E+08	16.151	5.77E-05	4.2392	7.35E-05	ctrl - P10; ctrl - P3; ctrl - P8; P11 - P10; P2 - P10; P3 - P10; P11 - P3; P11 - P8; P2 - P8; P3 - P8
TC0200010126.hg.1	TC0200010126.hg.1	geydoy.vcAug10-unspliced	geydoy	NA	16.146	5.77E-05	4.2385	7.36E-05	ctrl - P10; ctrl - P3; ctrl - P8; P11 - P10; P2 - P10; P2 - P11; P11 - P8; P2 - P3; P2 - P8; P3 - P8
TC0100011365.hg.1	TC0100011365.hg.1	NA	NA	NA	16.119	5.82E-05	4.2348	7.42E-05	ctrl - P10; ctrl - P11; ctrl - P8; P2 - P10; P3 - P10; P2 - P11; P3 - P11; P2 - P3; P2 - P8; P3 - P8
TC1100010404.hg.1	TC1100010404.hg.1	serlaby.aAug10-unspliced	serlaby	NA	16.117	5.83E-05	4.2346	7.42E-05	ctrl - P10; ctrl - P11; ctrl - P3; ctrl - P8; P2 - P10; P10 - P8; P2 - P11; P11 - P8; P2 - P3; P2 - P8; P3 - P8
TC1100009808.hg.1	TC1100009808.hg.1	NR_002728	KCNQ1OT1	11p15	16.066	5.92E-05	4.2277	7.53E-05	ctrl - P10; ctrl - P11; ctrl - P3; ctrl - P8; P2 - P10; P10 - P8; P2 - P11; P2 - P3; P2 - P8
TC1000008357.hg.1	TC1000008357.hg.1	smorsho.aAug10-unspliced	smorsho	NA	16.035	5.98E-05	4.2234	7.60E-05	ctrl - P10; ctrl - P11; ctrl - P8; P11 - P10; P2 - P10; P3 - P10; P2 - P11; P3 - P11; P2 - P8; P3 - P8
TC1100009293.hg.1	TC1100009293.hg.1	ENST00000621414	7SK		15.966	6.11E-05	4.214	7.77E-05	ctrl - P10; ctrl - P11; ctrl - P8; P11 - P10; P2 - P10; P3 - P10; P2 - P11; P11 - P8; P2 - P3; P2 - P8; P3 - P8
TC0200010732.hg.1	TC0200010732.hg.1	starswyby.aAug10-unspliced	starswyby	NA	15.962	6.12E-05	4.2134	7.77E-05	ctrl - P10; ctrl - P11; ctrl - P8; P2 - P10; P3 - P10; P10 - P8; P2 - P11; P3 - P11; P11 - P8; P2 - P8; P3 - P8
TC2100007090.hg.1	TC2100007090.hg.1	NA	NA	NA	15.934	6.17E-05	4.2095	7.84E-05	ctrl - P10; ctrl - P11; ctrl - P3; ctrl - P8; P2 - P10; P2 - P11; P11 - P8; P2 - P3; P2 - P8
TC1200009218.hg.1	TC1200009218.hg.1	woytybo.aAug10	woytybo	NA	15.932	6.18E-05	4.2093	7.84E-05	ctrl - P10; ctrl - P11; ctrl - P3; ctrl - P8; P2 - P10; P2 - P11; P2 - P3; P2 - P8; P3 - P8
TC0300007402.hg.1	TC0300007402.hg.1	sharglee.aAug10-unspliced	sharglee	NA	15.916	6.21E-05	4.207	7.88E-05	ctrl - P10; ctrl - P11; ctrl - P3; ctrl - P8; P2 - P10; P3 - P10; P2 - P11; P11 - P8; P2 - P3; P2 - P8; P3 - P8
TC1500007381.hg.1	TC1500007381.hg.1	woysto.aAug10-unspliced	woysto	NA	15.911	6.22E-05	4.2064	7.88E-05	ctrl - P10; ctrl - P11; ctrl - P8; P2 - P10; P3 - P10; P2 - P11; P3 - P11; P2 - P3; P2 - P8; P3 - P8
TC2000006765.hg.1	TC2000006765.hg.1	swoygar.aAug10-unspliced	swoygar	NA	15.905	6.23E-05	4.2055	7.89E-05	ctrl - P10; ctrl - P3; ctrl - P8; P11 - P10; P2 - P10; P11 - P3; P11 - P8; P2 - P3; P2 - P8
TC0800011986.hg.1	TC0800011986.hg.1	deylu.aAug10-unspliced	deylu	NA	15.876	6.29E-05	4.2015	7.96E-05	P2 - ctrl; P2 - P10; P3 - P10; P2 - P11; P3 - P11; P2 - P3; P2 - P8; P3 - P8
TC1600009622.hg.1	TC1600009622.hg.1	NA	NA	NA	15.864	6.31E-05	4.1999	7.99E-05	ctrl - P10; ctrl - P11; ctrl - P3; ctrl - P8; P2 - P10; P2 - P11; P2 - P3; P2 - P8
TC0300013423.hg.1	TC0300013423.hg.1	skawmeybu.aAug10-unspliced	skawmeybu	NA	15.853	6.33E-05	4.1984	8.01E-05	ctrl - P10; ctrl - P11; ctrl - P3; ctrl - P8; P2 - P10; P2 - P11; P11 - P8; P2 - P3; P2 - P8; P3 - P8
TC1700007346.hg.1	TC1700007346.hg.1	glarwee.aAug10-unspliced	glarwee	NA	15.826	6.39E-05	4.1947	8.08E-05	ctrl - P10; ctrl - P11; ctrl - P3; ctrl - P8; P2 - P10; P2 - P11; P2 - P3; P2 - P8; P3 - P8
TC1200008709.hg.1	TC1200008709.hg.1	bojeeby.aAug10-unspliced	bojeeby	NA	15.802	6.44E-05	4.1913	8.14E-05	ctrl - P10; ctrl - P11; ctrl - P8; P2 - P10; P3 - P10; P2 - P11; P11 - P8; P2 - P3; P2 - P8; P3 - P8
TC0300012051.hg.1	TC0300012051.hg.1	nekeme.bAug10-unspliced	nekeme	NA	15.79	6.46E-05	4.1896	8.17E-05	ctrl - P10; ctrl - P11; ctrl - P8; P2 - P10; P3 - P10; P2 - P11; P3 - P11; P2 - P8; P3 - P8
TC0300007137.hg.1	TC0300007137.hg.1	keefawbo.aAug10-unspliced	keefawbo	NA	15.781	6.48E-05	4.1884	8.18E-05	ctrl - P10; ctrl - P11; ctrl - P3; ctrl - P8; P2 - P10; P3 - P10; P2 - P11; P2 - P3; P2 - P8; P3 - P8
TC1200009548.hg.1	TC1200009548.hg.1	NR_120482	LOC102723544	1.03E+08	15.76	6.53E-05	4.1854	8.24E-05	ctrl - P10; ctrl - P11; ctrl - P3; ctrl - P8; P11 - P10; P2 - P10; P11 - P3; P11 - P8; P2 - P3; P2 - P8; P3 - P8
TC0200011196.hg.1	TC0200011196.hg.1	sluneebo.aAug10-unspliced	sluneebo	NA	15.749	6.55E-05	4.184	8.26E-05	ctrl - P10; ctrl - P11; ctrl - P8; P11 - P10; P2 - P10; P3 - P10; P2 - P11; P11 - P8; P2 - P3; P2 - P8; P3 - P8
TC1700008736.hg.1	TC1700008736.hg.1	NA	NA	NA	15.727	6.59E-05	4.1808	8.31E-05	ctrl - P10; ctrl - P8; P11 - P10; P2 - P10; P3 - P10; P11 - P8; P2 - P8; P3 - P8
TC0900006544.hg.1	TC0900006544.hg.1	ENST00000428071	MTATP6P11	NA	15.706	6.64E-05	4.178	8.36E-05	ctrl - P10; P2 - ctrl; ctrl - P8; P11 - P10; P2 - P10; P3 - P10; P2 - P11; P11 - P8; P2 - P8; P3 - P8

TC1200011593.hg.1	TC1200011593.hg.1	NA	NA	NA	15.684	6.68E-05	4.1749	8.42E-05	ctrl - P10; ctrl - P11; ctrl - P3; ctrl - P8; P2 - P10; P2 - P11; P3 - P11; P2 - P3; P2 - P8; P3 - P8
TC1800006618.hg.1	TC1800006618.hg.1	flukey.aAug10-unspliced	flukey	NA	15.676	6.70E-05	4.1737	8.44E-05	ctrl - P10; ctrl - P11; ctrl - P8; P2 - P10; P3 - P10; P2 - P11; P3 - P11; P2 - P8; P3 - P8
TC0300012246.hg.1	TC0300012246.hg.1	NA	NA	NA	15.662	6.73E-05	4.1719	8.47E-05	ctrl - P10; ctrl - P11; ctrl - P3; ctrl - P8; P2 - P10; P3 - P10; P2 - P11; P2 - P3; P2 - P8; P3 - P8
TC2200008440.hg.1	TC2200008440.hg.1	ENST00000608952.1	RP4-539M6.21		15.646	6.77E-05	4.1695	8.51E-05	ctrl - P10; ctrl - P3; ctrl - P8; P11 - P10; P2 - P10; P2 - P11; P11 - P8; P2 - P3; P2 - P8; P3 - P8
TC0200008003.hg.1	TC0200008003.hg.1	klorporbo.aAug10-unspliced	klorporbo	NA	15.642	6.78E-05	4.169	8.52E-05	ctrl - P10; ctrl - P3; ctrl - P8; P11 - P10; P2 - P10; P2 - P11; P11 - P8; P2 - P3; P2 - P8
TC0100017636.hg.1	TC0100017636.hg.1	ENST00000362464	RNA5S11	1E+08	15.612	6.84E-05	4.1648	8.58E-05	P11 - ctrl; P2 - ctrl; P3 - ctrl; P11 - P10; P2 - P10; P3 - P10; P2 - P11; P11 - P8; P2 - P3; P2 - P8; P3 - P8
TC0100017637.hg.1	TC0100017637.hg.1	ENST00000362400	RNA5S12	1E+08	15.612	6.84E-05	4.1648	8.58E-05	P11 - ctrl; P2 - ctrl; P3 - ctrl; P11 - P10; P2 - P10; P3 - P10; P2 - P11; P11 - P8; P2 - P3; P2 - P8; P3 - P8
TC0100017638.hg.1	TC0100017638.hg.1	ENST00000365656	RNA5S13	1E+08	15.612	6.84E-05	4.1648	8.58E-05	P11 - ctrl; P2 - ctrl; P3 - ctrl; P11 - P10; P2 - P10; P3 - P10; P2 - P11; P11 - P8; P2 - P3; P2 - P8; P3 - P8
TC0100017639.hg.1	TC0100017639.hg.1	ENST00000364485	RNA5S14	1E+08	15.612	6.84E-05	4.1648	8.58E-05	P11 - ctrl; P2 - ctrl; P3 - ctrl; P11 - P10; P2 - P10; P3 - P10; P2 - P11; P11 - P8; P2 - P3; P2 - P8; P3 - P8
TC0100017640.hg.1	TC0100017640.hg.1	ENST00000365055	RNA5S15	1E+08	15.612	6.84E-05	4.1648	8.58E-05	P11 - ctrl; P2 - ctrl; P3 - ctrl; P11 - P10; P2 - P10; P3 - P10; P2 - P11; P11 - P8; P2 - P3; P2 - P8; P3 - P8
TC0100017641.hg.1	TC0100017641.hg.1	ENST00000365387	RNA5S16	1E+08	15.612	6.84E-05	4.1648	8.58E-05	P11 - ctrl; P2 - ctrl; P3 - ctrl; P11 - P10; P2 - P10; P3 - P10; P2 - P11; P11 - P8; P2 - P3; P2 - P8; P3 - P8
TC0100008644.hg.1	TC0100008644.hg.1	skernubu.aAug10-unspliced	skernubu	NA	15.609	6.85E-05	4.1644	8.58E-05	ctrl - P10; ctrl - P11; ctrl - P3; ctrl - P8; P2 - P10; P2 - P11; P2 - P3; P2 - P8
TC0100010515.hg.1	TC0100010515.hg.1	sloymabu.aAug10-unspliced	sloymabu	NA	15.597	6.87E-05	4.1628	8.61E-05	ctrl - P10; ctrl - P11; P2 - ctrl; ctrl - P8; P2 - P10; P3 - P10; P2 - P11; P3 - P11; P2 - P3; P2 - P8; P3 - P8
TC1100010689.hg.1	TC1100010689.hg.1	lucher.aAug10-unspliced	lucher	NA	15.57	6.94E-05	4.1589	8.68E-05	ctrl - P10; ctrl - P11; ctrl - P3; ctrl - P8; P2 - P10; P2 - P11; P2 - P3; P2 - P8
TC0100008520.hg.1	TC0100008520.hg.1	luzawbo.aAug10-unspliced	luzawbo	NA	15.553	6.97E-05	4.1565	8.72E-05	ctrl - P10; P2 - ctrl; ctrl - P3; ctrl - P8; P11 - P10; P2 - P10; P2 - P11; P11 - P8; P2 - P3; P2 - P8
TC0200009861.hg.1	TC0200009861.hg.1	NR_110574	LOC101929633	1.02E+08	15.537	7.01E-05	4.1543	8.76E-05	ctrl - P10; ctrl - P3; ctrl - P8; P11 - P10; P2 - P10; P11 - P2; P11 - P3; P11 - P8; P2 - P3; P2 - P8
TC1200008728.hg.1	TC1200008728.hg.1	keeney.aAug10-unspliced	keeney	NA	15.536	7.01E-05	4.1541	8.76E-05	ctrl - P10; ctrl - P3; ctrl - P8; P11 - P10; P2 - P10; P10 - P3; P11 - P3; P11 - P8; P2 - P3; P2 - P8
TC1200007013.hg.1	TC1200007013.hg.1	flafy.aAug10-unspliced	flafy	NA	15.515	7.06E-05	4.1513	8.82E-05	ctrl - P10; ctrl - P11; ctrl - P8; P2 - P10; P3 - P10; P2 - P11; P3 - P11; P2 - P8; P3 - P8
TC0600012337.hg.1	TC0600012337.hg.1	NA	NA	NA	15.511	7.07E-05	4.1507	8.82E-05	ctrl - P10; ctrl - P11; ctrl - P3; ctrl - P8; P2 - P10; P3 - P10; P2 - P11; P2 - P3; P2 - P8; P3 - P8
TC1500009637.hg.1	TC1500009637.hg.1	seyzo.aAug10-unspliced	seyzo	NA	15.481	7.14E-05	4.1464	8.91E-05	ctrl - P11; ctrl - P8; P10 - P11; P10 - P8; P2 - P11; P3 - P11; P2 - P8; P3 - P8
TC0200007083.hg.1	TC0200007083.hg.1	gukla.aAug10-unspliced	gukla	NA	15.476	7.15E-05	4.1458	8.91E-05	ctrl - P10; ctrl - P8; P11 - P10; P2 - P10; P3 - P10; P11 - P8; P2 - P8; P3 - P8
TC0600010134.hg.1	TC0600010134.hg.1	dawblor.aAug10-unspliced	dawblor	NA	15.466	7.17E-05	4.1444	8.94E-05	ctrl - P10; ctrl - P11; ctrl - P3; ctrl - P8; P2 - P10; P2 - P11; P2 - P3; P2 - P8
TC0500010064.hg.1	TC0500010064.hg.1	klerflu.aAug10-unspliced	klerflu	NA	15.422	7.28E-05	4.1381	9.07E-05	ctrl - P10; ctrl - P11; ctrl - P3; ctrl - P8; P2 - P10; P2 - P11; P2 - P3; P2 - P8
TC0400008065.hg.1	TC0400008065.hg.1	jugy.aAug10-unspliced	jugy	NA	15.408	7.31E-05	4.1361	9.10E-05	ctrl - P10; ctrl - P11; ctrl - P3; ctrl - P8; P2 - P10; P2 - P11; P2 - P3; P2 - P8; P3 - P8
TC0100014028.hg.1	TC0100014028.hg.1	jeyrobo.aAug10-unspliced	jeyrobo	NA	15.406	7.32E-05	4.1358	9.10E-05	ctrl - P10; ctrl - P11; P2 - ctrl; ctrl - P8; P11 - P10; P2 - P10; P3 - P10; P2 - P11; P11 - P8; P2 - P3; P2 - P8; P3 - P8
TC0300007403.hg.1	TC0300007403.hg.1	shaforbo.aAug10-unspliced	shaforbo	NA	15.393	7.35E-05	4.134	9.14E-05	ctrl - P10; P2 - ctrl; ctrl - P3; ctrl - P8; P2 - P10; P2 - P11; P11 - P8; P2 - P3; P2 - P8
TC0900010043.hg.1	TC0900010043.hg.1	kamamu.bAug10-unspliced	kamamu	NA	15.383	7.37E-05	4.1326	9.16E-05	ctrl - P10; ctrl - P11; ctrl - P8; P2 - P10; P3 - P10; P2 - P11; P3 - P11; P11 - P8; P2 - P8; P3 - P8
TC0200006526.hg.1	TC0200006526.hg.1	porlaw.aAug10-unspliced	porlaw	NA	15.354	7.44E-05	4.1285	9.24E-05	ctrl - P10; ctrl - P11; ctrl - P3; ctrl - P8; P2 - P10; P2 - P11; P2 - P3; P2 - P8
TC1200007649.hg.1	TC1200007649.hg.1	kuchu.aAug10-unspliced	kuchu	NA	15.337	7.48E-05	4.126	9.29E-05	ctrl - P10; ctrl - P11; ctrl - P3; ctrl - P8; P11 - P10; P2 - P10; P3 - P10; P2 - P11; P11 - P8; P2 - P3; P2 - P8; P3 - P8
TC2100006637.hg.1	TC2100006637.hg.1	seygor.aAug10-unspliced	seygor	NA	15.332	7.49E-05	4.1253	9.30E-05	ctrl - P10; ctrl - P11; ctrl - P3; ctrl - P8; P2 - P10; P2 - P11; P11 - P3; P11 - P8; P2 - P3; P2 - P8
TC1600009632.hg.1	TC1600009632.hg.1	ENST00000567236.1	RP11-626G11.3		15.324	7.51E-05	4.1242	9.32E-05	P10 - ctrl; P11 - ctrl; P8 - ctrl; P10 - P11; P10 - P2; P10 - P3; P11 - P2; P8 - P11; P8 - P2; P8 - P3
TC0300010681.hg.1	TC0300010681.hg.1	stofubo.aAug10-unspliced	stofubo	NA	15.323	7.52E-05	4.1241	9.32E-05	ctrl - P10; ctrl - P11; ctrl - P3; ctrl - P8; P2 - P10; P2 - P11; P11 - P8; P2 - P3; P2 - P8; P3 - P8
TC1600009203.hg.1	TC1600009203.hg.1	flusnor.aAug10-unspliced	flusnor	NA	15.311	7.54E-05	4.1224	9.35E-05	ctrl - P10; ctrl - P11; ctrl - P3; ctrl - P8; P2 - P10; P2 - P11; P2 - P3; P2 - P8

TC0900007769.hg.1	TC0900007769.hg.1	wawwyby.aAug10-unspliced	wawwyby	NA	15.311	7.55E-05	4.1223	9.35E-05	ctrl - P10; ctrl - P3; ctrl - P8; P11 - P10; P2 - P10; P2 - P11; P11 - P3; P11 - P8; P2 - P3; P2 - P8
TC0700012233.hg.1	TC0700012233.hg.1	sehura.aAug10-unspliced	sehura	NA	15.295	7.58E-05	4.1201	9.39E-05	P10 - ctrl; P3 - ctrl; P8 - ctrl; P10 - P11; P10 - P2; P3 - P10; P3 - P11; P8 - P11; P3 - P2; P3 - P8
TC0300008842.hg.1	TC0300008842.hg.1	smoyboybu.aAug10-unspliced	smoyboybu	NA	15.284	7.61E-05	4.1185	9.42E-05	ctrl - P10; ctrl - P11; ctrl - P3; ctrl - P8; P11 - P10; P2 - P10; P11 - P3; P11 - P8; P2 - P3; P2 - P8
TC1400007719.hg.1	TC1400007719.hg.1	tihuri.aAug10-unspliced	tihuri	NA	15.268	7.65E-05	4.1162	9.47E-05	ctrl - P10; ctrl - P11; P2 - ctrl; ctrl - P8; P2 - P10; P3 - P10; P2 - P11; P3 - P11; P2 - P8; P3 - P8
TC0X00008864.hg.1	TC0X00008864.hg.1	koymy.aAug10-unspliced	koymy	NA	15.26	7.67E-05	4.115	9.49E-05	ctrl - P10; P2 - ctrl; ctrl - P3; ctrl - P8; P2 - P10; P2 - P11; P11 - P8; P2 - P3; P2 - P8; P3 - P8
TC0200012741.hg.1	TC0200012741.hg.1	watuyo.aAug10-unspliced	watuyo	NA	15.238	7.73E-05	4.1119	9.55E-05	ctrl - P10; ctrl - P11; ctrl - P8; P2 - P10; P3 - P10; P10 - P8; P2 - P11; P3 - P11; P11 - P8; P2 - P8; P3 - P8
TC1800008675.hg.1	TC1800008675.hg.1	NR_036204	MIR4320	1E+08	15.233	7.74E-05	4.1113	9.56E-05	ctrl - P10; ctrl - P11; ctrl - P3; ctrl - P8; P2 - P10; P10 - P8; P2 - P11; P11 - P8; P2 - P3; P2 - P8; P3 - P8
TSUnmapped00000340.hg.1	TSUnmapped00000340.hg.1	ENST00000627983	SNORD24		15.219	7.78E-05	4.1092	9.60E-05	ctrl - P10; P11 - ctrl; P2 - ctrl; ctrl - P3; P11 - P10; P2 - P10; P11 - P3; P11 - P8; P2 - P3; P2 - P8
TSUnmapped00000845.hg.1	TSUnmapped00000845.hg.1	NA	NA	NA	15.219	7.78E-05	4.1092	9.60E-05	ctrl - P10; P11 - ctrl; P2 - ctrl; ctrl - P3; P11 - P10; P2 - P10; P11 - P3; P11 - P8; P2 - P3; P2 - P8
TC1200011796.hg.1	TC1200011796.hg.1	NA	NA	NA	15.182	7.87E-05	4.1039	9.71E-05	P10 - ctrl; P11 - ctrl; ctrl - P3; P10 - P2; P10 - P3; P10 - P8; P11 - P2; P11 - P3; P11 - P8; P2 - P3; P8 - P3
TC0300006744.hg.1	TC0300006744.hg.1	ENST00000607363.1	RP11-415F23.3		15.179	7.88E-05	4.1036	9.71E-05	ctrl - P10; ctrl - P11; ctrl - P3; ctrl - P8; P11 - P10; P2 - P10; P2 - P11; P11 - P3; P11 - P8; P2 - P3; P2 - P8
TC0700007273.hg.1	TC0700007273.hg.1	loyku.aAug10-unspliced	loyku	NA	15.167	7.91E-05	4.1017	9.75E-05	ctrl - P10; ctrl - P11; ctrl - P3; ctrl - P8; P2 - P10; P3 - P10; P2 - P11; P3 - P11; P2 - P3; P2 - P8; P3 - P8
TC0X00008109.hg.1	TC0X00008109.hg.1	dadu.aAug10-unspliced	dadu	NA	15.124	8.03E-05	4.0955	9.88E-05	ctrl - P10; ctrl - P3; ctrl - P8; P11 - P10; P2 - P10; P3 - P10; P11 - P8; P2 - P3; P2 - P8; P3 - P8
TC0400008524.hg.1	TC0400008524.hg.1	ENST00000419264	LOC105377390	1.05E+08	15.118	8.04E-05	4.0947	9.90E-05	ctrl - P10; ctrl - P11; ctrl - P3; ctrl - P8; P10 - P11; P2 - P10; P2 - P11; P2 - P3; P2 - P8
TC0600010125.hg.1	TC0600010125.hg.1	tonire.aAug10-unspliced	tonire	NA	15.103	8.08E-05	4.0925	9.94E-05	ctrl - P10; ctrl - P11; ctrl - P3; ctrl - P8; P11 - P10; P2 - P10; P2 - P11; P11 - P8; P2 - P3; P2 - P8
TC1200006664.hg.1	TC1200006664.hg.1	spoyzeeby.aAug10	spoyzeeby	NA	15.098	8.09E-05	4.0918	9.96E-05	P10 - ctrl; P11 - ctrl; ctrl - P2; P8 - ctrl; P10 - P2; P10 - P3; P11 - P2; P11 - P3; P3 - P2; P8 - P2; P8 - P3
TC0600007900.hg.1	TC0600007900.hg.1	blorspabu.aAug10-unspliced	blorspabu	NA	15.085	8.13E-05	4.09	9.99E-05	ctrl - P10; ctrl - P11; ctrl - P8; P2 - P10; P3 - P10; P2 - P11; P11 - P8; P2 - P3; P2 - P8; P3 - P8
TC1700008456.hg.1	TC1700008456.hg.1	ENST00000623355	RP11-567L7.3		15.079	8.15E-05	4.089	0.0001	ctrl - P10; ctrl - P11; ctrl - P3; ctrl - P8; P2 - P10; P2 - P11; P11 - P8; P2 - P3; P2 - P8; P3 - P8
TC0600011202.hg.1	TC0600011202.hg.1	NA	NA	NA	15.065	8.18E-05	4.0871	0.0001	P10 - ctrl; P3 - ctrl; P8 - ctrl; P10 - P11; P10 - P2; P3 - P11; P8 - P11; P3 - P2; P8 - P2
HTA2-pos-2978683_st	23150241	NA	NA	NA	15.064	8.19E-05	4.0869	0.0001	ctrl - P10; ctrl - P8; P11 - P10; P2 - P10; P3 - P10; P11 - P8; P2 - P8; P3 - P8
TC0400007423.hg.1	TC0400007423.hg.1	NA	NA	NA	15.037	8.26E-05	4.0831	0.000101	ctrl - P11; P2 - ctrl; P3 - ctrl; ctrl - P8; P2 - P10; P3 - P10; P2 - P11; P3 - P11; P2 - P8; P3 - P8
TC1200008131.hg.1	TC1200008131.hg.1	chuno.aAug10-unspliced	chuno	NA	15.003	8.35E-05	4.0781	0.000102	ctrl - P10; ctrl - P11; ctrl - P3; ctrl - P8; P11 - P10; P2 - P10; P11 - P3; P11 - P8; P2 - P3; P2 - P8
TC0X00008337.hg.1	TC0X00008337.hg.1	NA	NA	NA	14.994	8.38E-05	4.0768	0.000103	ctrl - P10; ctrl - P11; ctrl - P3; ctrl - P8; P2 - P10; P3 - P10; P2 - P11; P11 - P8; P2 - P3; P2 - P8; P3 - P8
TC1900010841.hg.1	TC1900010841.hg.1	ENST00000595748.1	CTC-490G23.2		14.986	8.40E-05	4.0756	0.000103	P10 - ctrl; P3 - ctrl; P8 - ctrl; P10 - P11; P10 - P2; P3 - P11; P8 - P11; P3 - P2; P8 - P2
TC0300012655.hg.1	TC0300012655.hg.1	chydobu.aAug10-unspliced	chydobu	NA	14.977	8.42E-05	4.0744	0.000103	ctrl - P10; ctrl - P11; ctrl - P8; P2 - P10; P3 - P10; P2 - P11; P11 - P8; P2 - P3; P2 - P8; P3 - P8
TC1400009549.hg.1	TC1400009549.hg.1	NA	NA	NA	14.945	8.52E-05	4.0698	0.000104	P10 - ctrl; P11 - ctrl; P8 - ctrl; P10 - P2; P10 - P3; P11 - P2; P11 - P3; P3 - P2; P8 - P2; P8 - P3
TC0100009559.hg.1	TC0100009559.hg.1	gloymarbu.aAug10-unspliced	gloymarbu	NA	14.94	8.53E-05	4.069	0.000104	ctrl - P10; ctrl - P11; ctrl - P3; ctrl - P8; P2 - P10; P3 - P10; P2 - P11; P2 - P3; P2 - P8; P3 - P8
TC0X00008052.hg.1	TC0X00008052.hg.1	NM_001184782	CXorf57	55086	14.876	8.72E-05	4.0597	0.000107	ctrl - P10; ctrl - P2; ctrl - P3; ctrl - P8; P11 - P10; P11 - P2; P11 - P3; P11 - P8; P3 - P8
TC0400012610.hg.1	TC0400012610.hg.1	blysparby.aAug10-unspliced	blysparby	NA	14.815	8.90E-05	4.0508	0.000109	ctrl - P10; ctrl - P11; P2 - ctrl; ctrl - P8; P2 - P10; P3 - P10; P2 - P11; P11 - P8; P2 - P3; P2 - P8; P3 - P8
TC1400008819.hg.1	TC1400008819.hg.1	NA	NA	NA	14.814	8.90E-05	4.0507	0.000109	ctrl - P10; ctrl - P11; ctrl - P3; ctrl - P8; P2 - P10; P2 - P11; P11 - P8; P2 - P3; P2 - P8; P3 - P8
TC1700012350.hg.1	TC1700012350.hg.1	NA	NA	NA	14.811	8.91E-05	4.0503	0.000109	ctrl - P10; ctrl - P11; ctrl - P3; ctrl - P8; P11 - P10; P2 - P10; P2 - P11; P11 - P8; P2 - P3; P2 - P8; P3 - P8
TC1200008737.hg.1	TC1200008737.hg.1	dadoby.aAug10-unspliced	dadoby	NA	14.736	9.14E-05	4.0392	0.000111	ctrl - P10; ctrl - P11; ctrl - P3; ctrl - P8; P2 - P10; P3 - P10; P2 - P11; P2 - P3; P2 - P8; P3 - P8
TC0100008524.hg.1	TC0100008524.hg.1	norbor.aAug10-unspliced	norbor	NA	14.735	9.14E-05	4.039	0.000111	ctrl - P10; ctrl - P3; ctrl - P8; P11 - P10; P2 - P10; P3 - P10; P8 - P10; P2 - P11; P11 - P8; P2 - P3; P2 - P8

TC0800011585.hg.1	TC0800011585.hg.1	wyskerbu.aAug10-unspliced	wyskerbu	NA	14.722	9.18E-05	4.0371	0.000112	ctrl - P10; ctrl - P11; ctrl - P3; ctrl - P8; P2 - P10; P2 - P11; P3 - P11; P2 - P3; P2 - P8; P3 - P8
TC2000009443.hg.1	TC2000009443.hg.1	bleyvey.aAug10-unspliced	bleyvey	NA	14.716	9.20E-05	4.0363	0.000112	ctrl - P10; ctrl - P11; ctrl - P3; ctrl - P8; P2 - P10; P3 - P10; P2 - P11; P2 - P3; P2 - P8; P3 - P8
TC0300011409.hg.1	TC0300011409.hg.1	NA	NA	NA	14.71	9.22E-05	4.0353	0.000112	ctrl - P10; ctrl - P11; ctrl - P3; ctrl - P8; P11 - P10; P2 - P10; P2 - P11; P11 - P8; P2 - P3; P2 - P8; P3 - P8
TC0600010925.hg.1	TC0600010925.hg.1	NA	NA	NA	14.693	9.27E-05	4.0329	0.000113	ctrl - P10; ctrl - P11; ctrl - P8; P2 - P10; P3 - P10; P2 - P11; P3 - P11; P2 - P8; P3 - P8
TC0600011424.hg.1	TC0600011424.hg.1	ENST00000606743.1	XXbac-BPG181B23.7		14.693	9.27E-05	4.0328	0.000113	ctrl - P10; P11 - ctrl; ctrl - P3; ctrl - P8; P11 - P10; P2 - P10; P11 - P2; P11 - P3; P11 - P8; P2 - P3; P2 - P8
TC0500011304.hg.1	TC0500011304.hg.1	blaployby.aAug10-unspliced	blaployby	NA	14.685	9.30E-05	4.0317	0.000113	ctrl - P10; P2 - ctrl; ctrl - P8; P11 - P10; P2 - P10; P2 - P11; P11 - P8; P2 - P3; P2 - P8; P3 - P8
TC1400006999.hg.1	TC1400006999.hg.1	ENST00000553366	RP11-1053O12.1	NA	14.653	9.40E-05	4.027	0.000114	ctrl - P10; ctrl - P3; ctrl - P8; P11 - P10; P2 - P10; P11 - P3; P11 - P8; P2 - P3; P2 - P8
TC0200012635.hg.1	TC0200012635.hg.1	sworkabu.aAug10-unspliced	sworkabu	NA	14.644	9.43E-05	4.0256	0.000115	ctrl - P10; ctrl - P11; ctrl - P3; ctrl - P8; P2 - P10; P2 - P11; P11 - P8; P2 - P3; P2 - P8; P3 - P8
TC1600007033.hg.1	TC1600007033.hg.1	klawserbo.aAug10-unspliced	klawserbo	NA	14.621	9.50E-05	4.0222	0.000115	ctrl - P10; ctrl - P11; ctrl - P3; ctrl - P8; P2 - P10; P2 - P11; P2 - P3; P2 - P8; P3 - P8
TC1500008090.hg.1	TC1500008090.hg.1	mochabu.aAug10-unspliced	mochabu		14.612	9.53E-05	4.0209	0.000116	ctrl - P10; ctrl - P11; ctrl - P3; ctrl - P8; P2 - P10; P2 - P11; P11 - P8; P2 - P3; P2 - P8
TC0700008824.hg.1	TC0700008824.hg.1	shorpaby.aAug10-unspliced	shorpaby	NA	14.612	9.53E-05	4.0209	0.000116	ctrl - P10; ctrl - P11; ctrl - P3; ctrl - P8; P2 - P10; P2 - P11; P2 - P3; P2 - P8
TC0400010127.hg.1	TC0400010127.hg.1	yatome.aAug10-unspliced	yatome	NA	14.607	9.55E-05	4.0202	0.000116	ctrl - P10; ctrl - P11; ctrl - P3; ctrl - P8; P2 - P10; P2 - P11; P2 - P3; P2 - P8
TC0700010684.hg.1	TC0700010684.hg.1	flublar.aAug10-unspliced	flublar	NA	14.602	9.56E-05	4.0195	0.000116	ctrl - P10; ctrl - P11; ctrl - P8; P2 - P10; P3 - P10; P2 - P11; P3 - P11; P2 - P8; P3 - P8
TC1000007565.hg.1	TC1000007565.hg.1	neymar.aAug10-unspliced	neymar	NA	14.601	9.56E-05	4.0194	0.000116	ctrl - P10; ctrl - P11; ctrl - P8; P2 - P10; P3 - P10; P2 - P11; P11 - P8; P2 - P3; P2 - P8; P3 - P8
TC0600008549.hg.1	TC0600008549.hg.1	NA	NA	NA	14.594	9.59E-05	4.0183	0.000116	P10 - ctrl; P2 - ctrl; P8 - ctrl; P10 - P11; P10 - P3; P2 - P11; P8 - P11; P2 - P3; P2 - P8; P8 - P3
TC0700007049.hg.1	TC0700007049.hg.1	soyja.aAug10-unspliced	soyja	NA	14.572	9.66E-05	4.0151	0.000117	ctrl - P10; ctrl - P8; P11 - P10; P2 - P10; P3 - P10; P11 - P8; P2 - P8; P3 - P8
TC0200009960.hg.1	TC0200009960.hg.1	padar.aAug10-unspliced	padar	NA	14.521	9.83E-05	4.0075	0.000119	ctrl - P10; ctrl - P8; P11 - P10; P2 - P10; P3 - P10; P2 - P11; P11 - P8; P2 - P8; P3 - P8
TC0800011251.hg.1	TC0800011251.hg.1	savawby.aAug10-unspliced	savawby	NA	14.491	9.93E-05	4.003	0.00012	ctrl - P10; ctrl - P11; ctrl - P3; ctrl - P8; P2 - P10; P2 - P11; P2 - P3; P2 - P8
TC1800006600.hg.1	TC1800006600.hg.1	kukori.aAug10-unspliced	kukori	NA	14.485	9.95E-05	4.0021	0.00012	ctrl - P10; ctrl - P11; ctrl - P8; P2 - P10; P3 - P10; P10 - P8; P2 - P11; P3 - P11; P11 - P8; P2 - P8; P3 - P8
TC1800009088.hg.1	TC1800009088.hg.1	glorploy.aAug10-unspliced	glorploy	NA	14.476	9.98E-05	4.0007	0.000121	ctrl - P10; ctrl - P11; ctrl - P8; P2 - P10; P10 - P8; P2 - P11; P3 - P11; P2 - P3; P2 - P8; P3 - P8
TC0500011034.hg.1	TC0500011034.hg.1	cheeju.aAug10-unspliced	cheeju	NA	14.468	0.0001	3.9995	0.000121	ctrl - P10; ctrl - P11; ctrl - P3; ctrl - P8; P2 - P10; P2 - P11; P11 - P8; P2 - P3; P2 - P8; P3 - P8
TC0500006812.hg.1	TC0500006812.hg.1	voyslarbu.aAug10-unspliced	voyslarbu	NA	14.465	0.0001	3.9991	0.000121	ctrl - P10; ctrl - P11; ctrl - P8; P2 - P10; P3 - P10; P10 - P8; P2 - P11; P3 - P11; P2 - P3; P2 - P8; P3 - P8
TC1500007321.hg.1	TC1500007321.hg.1	skeyklo.aAug10-unspliced	skeyklo	NA	14.46	0.0001	3.9983	0.000121	ctrl - P10; P2 - ctrl; ctrl - P8; P11 - P10; P2 - P10; P3 - P10; P2 - P11; P2 - P3; P2 - P8; P3 - P8
TC0600007356.hg.1	TC0600007356.hg.1	NA	NA	NA	14.459	0.0001	3.9982	0.000121	P10 - ctrl; P3 - ctrl; P8 - ctrl; P10 - P11; P10 - P2; P3 - P11; P8 - P11; P3 - P2; P8 - P2
TC0200007187.hg.1	TC0200007187.hg.1	slerjeebu.aAug10-unspliced	slerjeebu	NA	14.459	0.0001	3.9981	0.000121	ctrl - P10; ctrl - P11; ctrl - P3; ctrl - P8; P2 - P10; P3 - P10; P2 - P11; P2 - P3; P2 - P8
TC0300011411.hg.1	TC0300011411.hg.1	perverbo.aAug10-unspliced	perverbo	NA	14.455	0.000101	3.9976	0.000121	ctrl - P10; ctrl - P11; ctrl - P3; ctrl - P8; P2 - P10; P2 - P11; P11 - P8; P2 - P3; P2 - P8
TC0100015961.hg.1	TC0100015961.hg.1	romaro.aAug10-unspliced	romaro	NA	14.417	0.000102	3.9919	0.000123	ctrl - P10; P2 - ctrl; ctrl - P8; P11 - P10; P2 - P10; P2 - P11; P11 - P8; P2 - P3; P2 - P8; P3 - P8
TC0800011545.hg.1	TC0800011545.hg.1	NA	NA	NA	14.41	0.000102	3.9909	0.000123	ctrl - P10; ctrl - P11; P2 - ctrl; ctrl - P8; P2 - P10; P3 - P10; P2 - P11; P3 - P11; P2 - P3; P2 - P8; P3 - P8
TC0100013883.hg.1	TC0100013883.hg.1	jupla.aAug10-unspliced	jupla	NA	14.36	0.000104	3.9835	0.000125	ctrl - P10; ctrl - P11; ctrl - P3; ctrl - P8; P2 - P10; P3 - P10; P2 - P11; P2 - P3; P2 - P8; P3 - P8
TC0100011866.hg.1	TC0100011866.hg.1	NA	NA	NA	14.352	0.000104	3.9822	0.000125	ctrl - P10; ctrl - P11; ctrl - P3; ctrl - P8; P2 - P10; P3 - P10; P10 - P8; P2 - P11; P11 - P8; P2 - P8; P3 - P8
TC0600013904.hg.1	TC0600013904.hg.1	RNASET2andRPS6KA2.voAug10-unspliced	RNASET2andRPS6KA2	NA	14.339	0.000105	3.9803	0.000126	ctrl - P10; ctrl - P11; ctrl - P3; ctrl - P8; P10 - P11; P2 - P10; P2 - P11; P2 - P3; P2 - P8
HTA2-pos-47424483_st	23150859	NA	NA	NA	14.331	0.000105	3.9791	0.000126	ctrl - P10; ctrl - P11; ctrl - P8; P2 - P10; P3 - P10; P2 - P11; P2 - P3; P2 - P8; P3 - P8

HTA2-pos-3615330_st	23150705	NA	NA	NA	14.294	0.000106	3.9735	0.000128	P10 - ctrl; P11 - ctrl; ctrl - P2; P3 - ctrl; P8 - ctrl; P10 - P2; P11 - P2; P3 - P2; P8 - P2
TC0100011486.hg.1	TC0100011486.hg.1	NA	NA	NA	14.268	0.000107	3.9696	0.000129	ctrl - P10; ctrl - P11; ctrl - P3; ctrl - P8; P2 - P10; P2 - P11; P2 - P3; P2 - P8; P3 - P8
TC2200008362.hg.1	TC2200008362.hg.1	spopey.aAug10-unspliced	spopey	NA	14.221	0.000109	3.9624	0.000131	ctrl - P10; ctrl - P11; ctrl - P8; P2 - P10; P3 - P10; P2 - P11; P3 - P11; P2 - P8; P3 - P8
TC2100007809.hg.1	TC2100007809.hg.1	womu.aAug10-unspliced	womu	NA	14.207	0.00011	3.9604	0.000131	ctrl - P10; ctrl - P11; ctrl - P3; ctrl - P8; P2 - P10; P2 - P11; P2 - P3; P2 - P8
TC1500008024.hg.1	TC1500008024.hg.1	steezoybu.aAug10-unspliced	steezoybu	NA	14.204	0.00011	3.9599	0.000131	ctrl - P10; ctrl - P11; ctrl - P3; ctrl - P8; P2 - P10; P3 - P10; P2 - P11; P2 - P3; P2 - P8; P3 - P8
TC0900006541.hg.1	TC0900006541.hg.1	ENST00000456661	MTND1P11	NA	14.144	0.000112	3.9508	0.000134	ctrl - P10; ctrl - P8; P11 - P10; P2 - P10; P3 - P10; P11 - P8; P2 - P8; P3 - P8
TC1100006889.hg.1	TC1100006889.hg.1	bykorby.aAug10-unspliced	bykorby	NA	14.131	0.000112	3.9489	0.000135	ctrl - P10; ctrl - P11; ctrl - P8; P2 - P10; P2 - P11; P3 - P11; P2 - P3; P2 - P8; P3 - P8
TC1100009235.hg.1	TC1100009235.hg.1	ENST00000531886.1	RP11-110I1.6		14.098	0.000114	3.9438	0.000136	P10 - ctrl; P11 - ctrl; P3 - ctrl; P8 - ctrl; P10 - P2; P10 - P3; P11 - P2; P11 - P3; P8 - P2; P8 - P3
TC1700010985.hg.1	TC1700010985.hg.1	uc060guz.1	RNU6-1152P	NA	14.091	0.000114	3.9427	0.000136	ctrl - P10; ctrl - P11; ctrl - P8; P2 - P10; P3 - P10; P2 - P11; P11 - P8; P2 - P3; P2 - P8; P3 - P8
TC0X00008989.hg.1	TC0X00008989.hg.1	NM_001282145	NLGN4X	57502	14.07	0.000115	3.9396	0.000137	ctrl - P10; ctrl - P2; ctrl - P3; ctrl - P8; P11 - P10; P11 - P2; P11 - P3; P11 - P8; P3 - P2; P3 - P8
TC0500007535.hg.1	TC0500007535.hg.1	NA	NA	NA	14.068	0.000115	3.9392	0.000137	ctrl - P10; ctrl - P11; ctrl - P3; ctrl - P8; P2 - P10; P2 - P11; P2 - P3; P2 - P8
TC0600009289.hg.1	TC0600009289.hg.1	spuplawbu.aAug10-unspliced	spuplawbu	NA	14.035	0.000116	3.9342	0.000139	ctrl - P10; ctrl - P11; ctrl - P3; ctrl - P8; P11 - P10; P2 - P10; P2 - P11; P11 - P3; P11 - P8; P2 - P3; P2 - P8
TC0600013465.hg.1	TC0600013465.hg.1	NA	NA	NA	14.034	0.000116	3.9339	0.000139	ctrl - P10; ctrl - P11; ctrl - P3; ctrl - P8; P11 - P10; P2 - P10; P2 - P11; P11 - P8; P2 - P3; P2 - P8
TC0X00007705.hg.1	TC0X00007705.hg.1	skeybey.aAug10-unspliced	skeybey	NA	14.016	0.000117	3.9313	0.00014	ctrl - P10; ctrl - P11; ctrl - P3; ctrl - P8; P11 - P10; P2 - P10; P11 - P3; P11 - P8; P2 - P3; P2 - P8
TC1300009259.hg.1	TC1300009259.hg.1	NA	NA	NA	13.998	0.000118	3.9285	0.000141	ctrl - P10; ctrl - P11; ctrl - P3; ctrl - P8; P2 - P10; P2 - P11; P2 - P3; P2 - P8
TC0800011548.hg.1	TC0800011548.hg.1	snordorby.aAug10-unspliced	snordorby	NA	13.976	0.000119	3.9251	0.000142	ctrl - P10; P2 - ctrl; ctrl - P8; P2 - P10; P3 - P10; P2 - P11; P3 - P11; P2 - P3; P2 - P8; P3 - P8
TC1200012757.hg.1	TC1200012757.hg.1	ENST00000542513	TAS2R12		13.939	0.00012	3.9195	0.000143	ctrl - P10; ctrl - P8; P11 - P10; P2 - P10; P3 - P10; P2 - P11; P11 - P8; P2 - P3; P2 - P8; P3 - P8
TC1300006484.hg.1	TC1300006484.hg.1	teyare.aAug10-unspliced	teyare	NA	13.931	0.000121	3.9182	0.000144	ctrl - P10; ctrl - P11; ctrl - P3; ctrl - P8; P2 - P10; P2 - P11; P2 - P3; P2 - P8
TC1900009538.hg.1	TC1900009538.hg.1	NA	NA	NA	13.909	0.000122	3.9148	0.000145	P10 - ctrl; P11 - ctrl; P8 - ctrl; P10 - P2; P10 - P3; P10 - P8; P11 - P2; P11 - P3; P8 - P2; P8 - P3
TC1200010928.hg.1	TC1200010928.hg.1	NR_003046	SNORD59B	12q13.3	13.901	0.000122	3.9136	0.000145	ctrl - P10; ctrl - P3; ctrl - P8; P11 - P10; P2 - P10; P11 - P3; P11 - P8; P2 - P3; P2 - P8
TC0300012491.hg.1	TC0300012491.hg.1	shudabu.aAug10-unspliced	shudabu	NA	13.9	0.000122	3.9135	0.000145	ctrl - P10; ctrl - P11; P2 - ctrl; ctrl - P8; P2 - P10; P2 - P11; P2 - P3; P2 - P8; P3 - P8
TC0700006841.hg.1	TC0700006841.hg.1	ENST00000430859	LOC100506098	1.01E+08	13.892	0.000122	3.9122	0.000145	P11 - ctrl; P8 - ctrl; P11 - P10; P10 - P2; P10 - P3; P8 - P10; P11 - P2; P11 - P3; P8 - P2; P8 - P3
TC0200007420.hg.1	TC0200007420.hg.1	NA	NA	NA	13.888	0.000123	3.9117	0.000146	ctrl - P10; ctrl - P3; ctrl - P8; P11 - P10; P2 - P10; P3 - P10; P2 - P11; P11 - P8; P2 - P3; P2 - P8; P3 - P8
TC0500010395.hg.1	TC0500010395.hg.1	fuzyby.aAug10-unspliced	fuzyby	NA	13.879	0.000123	3.9102	0.000146	ctrl - P10; ctrl - P11; ctrl - P3; ctrl - P8; P2 - P10; P2 - P11; P2 - P3; P2 - P8; P3 - P8
HTA2-pos-3145790_st	23150340	NA	NA	NA	13.831	0.000125	3.9028	0.000148	ctrl - P10; ctrl - P3; ctrl - P8; P2 - P10; P2 - P11; P11 - P3; P11 - P8; P2 - P3; P2 - P8
TC0100014621.hg.1	TC0100014621.hg.1	sawmarbo.aAug10-unspliced	sawmarbo	NA	13.802	0.000126	3.8982	0.00015	P2 - ctrl; P3 - ctrl; P11 - P10; P2 - P10; P3 - P10; P2 - P11; P11 - P8; P2 - P3; P2 - P8; P3 - P8
TC0900011820.hg.1	TC0900011820.hg.1	TSC1.pAug10-unspliced	TSC1	NA	13.796	0.000127	3.8974	0.00015	ctrl - P10; ctrl - P11; ctrl - P3; ctrl - P8; P2 - P10; P2 - P11; P2 - P3; P2 - P8
TC0600011170.hg.1	TC0600011170.hg.1	NA	NA	NA	13.774	0.000128	3.894	0.000151	ctrl - P10; P2 - ctrl; ctrl - P3; ctrl - P8; P2 - P10; P10 - P8; P2 - P11; P11 - P8; P2 - P3; P2 - P8
TC0800011537.hg.1	TC0800011537.hg.1	gloverby.aAug10-unspliced	gloverby	NA	13.649	0.000134	3.8745	0.000158	ctrl - P10; ctrl - P11; ctrl - P3; ctrl - P8; P2 - P10; P2 - P11; P2 - P3; P2 - P8; P3 - P8
TC0600012336.hg.1	TC0600012336.hg.1	NA	NA	NA	13.646	0.000134	3.8741	0.000158	ctrl - P10; ctrl - P11; ctrl - P8; P2 - P10; P3 - P10; P2 - P11; P3 - P11; P11 - P8; P2 - P8; P3 - P8
TC0900006539.hg.1	TC0900006539.hg.1	hsa_circ_0001838	JAK2	NA	13.628	0.000135	3.8712	0.000159	ctrl - P10; ctrl - P11; ctrl - P8; P2 - P10; P3 - P10; P2 - P11; P11 - P8; P2 - P3; P2 - P8; P3 - P8
TC1000007709.hg.1	TC1000007709.hg.1	NA	NA	NA	13.624	0.000135	3.8706	0.000159	ctrl - P10; ctrl - P11; ctrl - P3; ctrl - P8; P2 - P10; P2 - P11; P2 - P3; P2 - P8; P3 - P8

TC1500007661.hg.1	TC1500007661.hg.1	slarzo.aAug10-unspliced	slarzo	NA	13.618	0.000135	3.8697	0.00016	ctrl - P10; ctrl - P11; ctrl - P3; ctrl - P8; P2 - P10; P10 - P8; P2 - P11; P11 - P8; P2 - P3; P2 - P8; P3 - P8
TC0200007133.hg.1	TC0200007133.hg.1	dawfa.aAug10-unspliced	dawfa	NA	13.608	0.000135	3.8681	0.00016	ctrl - P10; ctrl - P11; ctrl - P3; ctrl - P8; P2 - P10; P3 - P10; P2 - P11; P11 - P8; P2 - P3; P2 - P8; P3 - P8
TC2000007088.hg.1	TC2000007088.hg.1	NR_131907	ABALON	1.03E+08	13.602	0.000136	3.8671	0.00016	P10 - ctrl; P11 - ctrl; P3 - ctrl; P8 - ctrl; P10 - P11; P10 - P2; P10 - P3; P11 - P2; P3 - P2; P8 - P2
TC1800007686.hg.1	TC1800007686.hg.1	veywar.aAug10-unspliced	veywar	NA	13.601	0.000136	3.867	0.00016	ctrl - P10; ctrl - P11; ctrl - P3; ctrl - P8; P2 - P10; P3 - P10; P2 - P11; P2 - P3; P2 - P8; P3 - P8
TC1800008628.hg.1	TC1800008628.hg.1	NM_001039360	ZBTB7C	201501	13.508	0.00014	3.8523	0.000166	P11 - ctrl; ctrl - P2; P3 - ctrl; ctrl - P8; P11 - P10; P3 - P10; P11 - P2; P11 - P8; P3 - P2; P3 - P8
TC0400012223.hg.1	TC0400012223.hg.1	slorfley.aAug10-unspliced	slorfley	NA	13.505	0.000141	3.8519	0.000166	ctrl - P10; ctrl - P11; ctrl - P8; P2 - P10; P3 - P10; P2 - P11; P3 - P11; P2 - P3; P2 - P8; P3 - P8
TC0500006811.hg.1	TC0500006811.hg.1	shorskoby.aAug10-unspliced	shorskoby	NA	13.493	0.000141	3.85	0.000166	ctrl - P10; ctrl - P11; ctrl - P8; P2 - P10; P3 - P10; P2 - P11; P3 - P11; P2 - P3; P2 - P8; P3 - P8
TC0300012054.hg.1	TC0300012054.hg.1	blorbobu.aAug10-unspliced	blorbobu	NA	13.486	0.000142	3.8488	0.000167	ctrl - P10; ctrl - P11; ctrl - P8; P11 - P10; P2 - P10; P3 - P10; P2 - P11; P11 - P8; P2 - P3; P2 - P8; P3 - P8
TC0X00007542.hg.1	TC0X00007542.hg.1	plerby.aAug10-unspliced	plerby	NA	13.482	0.000142	3.8482	0.000167	ctrl - P10; ctrl - P11; ctrl - P3; ctrl - P8; P2 - P10; P2 - P11; P11 - P8; P2 - P3; P2 - P8; P3 - P8
TC0600012815.hg.1	TC0600012815.hg.1	NM_001111298	PPIL6	285755	13.464	0.000143	3.8454	0.000168	ctrl - P10; ctrl - P2; ctrl - P3; ctrl - P8; P11 - P10; P2 - P10; P11 - P2; P11 - P3; P11 - P8
TC0300011079.hg.1	TC0300011079.hg.1	tuforbo.aAug10-unspliced	tuforbo	NA	13.438	0.000144	3.8412	0.00017	ctrl - P10; ctrl - P11; ctrl - P3; ctrl - P8; P2 - P10; P3 - P10; P2 - P11; P2 - P3; P2 - P8
TC0200014856.hg.1	TC0200014856.hg.1	NA	NA	NA	13.424	0.000145	3.8391	0.00017	ctrl - P10; ctrl - P3; ctrl - P8; P11 - P10; P2 - P10; P11 - P3; P11 - P8; P2 - P3; P2 - P8
TC1300007800.hg.1	TC1300007800.hg.1	jatoby.aAug10-unspliced	jatoby	NA	13.422	0.000145	3.8387	0.00017	ctrl - P10; ctrl - P11; ctrl - P8; P2 - P10; P3 - P10; P2 - P11; P3 - P11; P2 - P3; P2 - P8; P3 - P8
TC1500009922.hg.1	TC1500009922.hg.1	OTTHUMT00000474076	RP11-138H8.8		13.375	0.000147	3.8313	0.000173	ctrl - P10; ctrl - P11; ctrl - P3; ctrl - P8; P2 - P10; P3 - P10; P2 - P11; P3 - P11; P2 - P3; P2 - P8; P3 - P8
HTA2-pos-PSR02023980.hg.1	23151073	NA	NA	NA	13.369	0.000148	3.8304	0.000174	ctrl - P10; ctrl - P8; P11 - P10; P2 - P10; P3 - P10; P2 - P11; P11 - P8; P2 - P3; P2 - P8; P3 - P8
TC0700012269.hg.1	TC0700012269.hg.1	hinare.aAug10-unspliced	hinare	NA	13.369	0.000148	3.8303	0.000174	ctrl - P10; ctrl - P8; P11 - P10; P2 - P10; P3 - P10; P2 - P11; P11 - P8; P2 - P3; P2 - P8; P3 - P8
TC1200012049.hg.1	TC1200012049.hg.1	humiri.aAug10-unspliced	humiri	NA	13.366	0.000148	3.8299	0.000174	ctrl - P10; ctrl - P11; ctrl - P3; ctrl - P8; P2 - P10; P2 - P11; P11 - P8; P2 - P3; P2 - P8; P3 - P8
TC1700007345.hg.1	TC1700007345.hg.1	ENST00000516474	Vault		13.315	0.000151	3.8218	0.000177	ctrl - P10; ctrl - P11; ctrl - P8; P2 - P10; P3 - P10; P2 - P11; P2 - P3; P2 - P8; P3 - P8
TC0X00007420.hg.1	TC0X00007420.hg.1	wardaw.aAug10-unspliced	wardaw	NA	13.295	0.000152	3.8185	0.000178	ctrl - P10; P2 - ctrl; ctrl - P8; P11 - P10; P2 - P10; P3 - P10; P2 - P11; P11 - P8; P2 - P3; P2 - P8; P3 - P8
TC1500009336.hg.1	TC1500009336.hg.1	MYEF2.jAug10-unspliced	MYEF2	NA	13.262	0.000154	3.8133	0.00018	ctrl - P10; ctrl - P11; ctrl - P3; ctrl - P8; P2 - P10; P2 - P11; P2 - P3; P2 - P8; P3 - P8
TC0300006575.hg.1	TC0300006575.hg.1	NM_001018115	FANCD2	2177	13.141	0.000161	3.7938	0.000188	P10 - ctrl; P8 - ctrl; P10 - P11; P10 - P2; P10 - P3; P8 - P11; P8 - P2; P8 - P3
TC0700013106.hg.1	TC0700013106.hg.1	leysmawbu.aAug10-unspliced	leysmawbu	NA	13.115	0.000162	3.7896	0.00019	ctrl - P10; ctrl - P11; ctrl - P3; ctrl - P8; P2 - P10; P2 - P11; P11 - P8; P2 - P3; P2 - P8
TC0X00010637.hg.1	TC0X00010637.hg.1	NA	NA	NA	13.111	0.000163	3.7889	0.00019	P11 - ctrl; ctrl - P2; ctrl - P3; P11 - P10; P10 - P2; P10 - P3; P11 - P2; P11 - P3; P8 - P2; P8 - P3
TC0900009462.hg.1	TC0900009462.hg.1	guroyby.aAug10-unspliced	guroyby	NA	13.103	0.000163	3.7876	0.000191	ctrl - P10; ctrl - P8; P11 - P10; P2 - P10; P3 - P10; P11 - P8; P2 - P8; P3 - P8
TC0100009448.hg.1	TC0100009448.hg.1	gawkoybu.aAug10-unspliced	gawkoybu	NA	13.101	0.000163	3.7874	0.000191	ctrl - P10; ctrl - P11; ctrl - P3; ctrl - P8; P2 - P10; P2 - P11; P2 - P3; P2 - P8; P3 - P8
TC0100012740.hg.1	TC0100012740.hg.1	reeplu.aAug10-unspliced	reeplu	NA	13.098	0.000163	3.7868	0.000191	ctrl - P10; ctrl - P11; ctrl - P8; P2 - P10; P3 - P10; P2 - P11; P3 - P11; P2 - P8; P3 - P8
TC1700010547.hg.1	TC1700010547.hg.1	NA	NA	NA	13.089	0.000164	3.7854	0.000191	ctrl - P10; ctrl - P11; ctrl - P3; ctrl - P8; P2 - P10; P2 - P11; P2 - P3; P2 - P8
TC0Y00007289.hg.1	TC0Y00007289.hg.1	rarsybo.aAug10-unspliced	rarsybo	NA	13.084	0.000164	3.7847	0.000192	ctrl - P10; ctrl - P11; ctrl - P2; ctrl - P3; ctrl - P8; P11 - P10; P3 - P10; P11 - P2; P3 - P2
TC0300008806.hg.1	TC0300008806.hg.1	NA	NA	NA	13.043	0.000167	3.7779	0.000195	ctrl - P10; ctrl - P8; P11 - P10; P2 - P10; P3 - P10; P2 - P11; P11 - P8; P2 - P3; P2 - P8; P3 - P8
TC1000010729.hg.1	TC1000010729.hg.1	NA	NA	NA	13.024	0.000168	3.7749	0.000196	ctrl - P10; ctrl - P11; ctrl - P3; ctrl - P8; P11 - P10; P2 - P10; P11 - P3; P11 - P8; P2 - P3; P2 - P8
TC0400006886.hg.1	TC0400006886.hg.1	vafloy.aAug10-unspliced	vafloy	NA	12.982	0.000171	3.768	0.000199	ctrl - P10; ctrl - P11; ctrl - P3; ctrl - P8; P2 - P10; P11 - P3; P11 - P8; P2 - P3; P2 - P8
HTA2-pos-47424482_st	23150858	NA	NA	NA	12.957	0.000172	3.764	0.000201	ctrl - P10; ctrl - P11; ctrl - P3; ctrl - P8; P2 - P10; P2 - P11; P2 - P3; P2 - P8

TC0100010613.hg.1	TC0100010613.hg.1	gerkerbo.aAug10-unspliced	gerkerbo	NA	12.952	0.000173	3.7631	0.000201	ctrl - P11; ctrl - P8; P2 - P10; P3 - P10; P10 - P8; P2 - P11; P3 - P11; P11 - P8; P2 - P8; P3 - P8
TC1200007451.hg.1	TC1200007451.hg.1	NA	NA	NA	12.915	0.000175	3.757	0.000204	ctrl - P10; ctrl - P11; ctrl - P3; ctrl - P8; P2 - P10; P2 - P11; P2 - P3; P2 - P8
TC0700008748.hg.1	TC0700008748.hg.1	tawkaw.aAug10-unspliced	tawkaw	NA	12.891	0.000177	3.7531	0.000205	ctrl - P10; ctrl - P11; ctrl - P3; ctrl - P8; P2 - P10; P2 - P11; P11 - P8; P2 - P3; P2 - P8; P3 - P8
TC1400006465.hg.1	TC1400006465.hg.1	ENST00000612637	CTD-2314B22.2	NA	12.879	0.000177	3.7511	0.000206	ctrl - P10; ctrl - P11; ctrl - P8; P11 - P10; P2 - P10; P3 - P10; P11 - P8; P2 - P8; P3 - P8
HTA2-neg-47424450_st	23150112	NA	NA	NA	12.855	0.000179	3.7473	0.000208	ctrl - P10; ctrl - P11; ctrl - P3; ctrl - P8; P2 - P10; P2 - P11; P11 - P3; P11 - P8; P2 - P3; P2 - P8
TC0500013077.hg.1	TC0500013077.hg.1	pleystoybu.aAug10-unspliced	pleystoybu	NA	12.838	0.00018	3.7445	0.000209	ctrl - P10; P2 - ctrl; ctrl - P8; P11 - P10; P2 - P10; P3 - P10; P2 - P11; P11 - P8; P2 - P3; P2 - P8; P3 - P8
TC0300012067.hg.1	TC0300012067.hg.1	swarbobu.aAug10-unspliced	swarbobu	NA	12.817	0.000182	3.7411	0.000211	ctrl - P10; P2 - ctrl; ctrl - P8; P11 - P10; P2 - P10; P2 - P11; P11 - P8; P2 - P3; P2 - P8
TC1300009254.hg.1	TC1300009254.hg.1	snorfeeby.aAug10-unspliced	snorfeeby	NA	12.817	0.000182	3.741	0.000211	ctrl - P10; P2 - ctrl; ctrl - P3; ctrl - P8; P2 - P10; P2 - P11; P11 - P8; P2 - P3; P2 - P8; P3 - P8
TC0300013686.hg.1	TC0300013686.hg.1	gleynarbo.aAug10-unspliced	gleynarbo	NA	12.812	0.000182	3.7402	0.000211	ctrl - P10; ctrl - P11; ctrl - P3; ctrl - P8; P2 - P10; P11 - P8; P2 - P3; P2 - P8
TC0300006842.hg.1	TC0300006842.hg.1	jorfabo.aAug10-unspliced	jorfabo	NA	12.804	0.000182	3.739	0.000212	ctrl - P10; ctrl - P11; ctrl - P8; P2 - P10; P3 - P10; P2 - P11; P3 - P11; P2 - P3; P2 - P8; P3 - P8
TC1200011477.hg.1	TC1200011477.hg.1	shawnar.aAug10-unspliced	shawnar	NA	12.732	0.000188	3.727	0.000217	ctrl - P10; ctrl - P11; ctrl - P3; ctrl - P8; P11 - P10; P2 - P10; P3 - P10; P11 - P8; P2 - P8; P3 - P8
TC0200009077.hg.1	TC0200009077.hg.1	NA	NA	NA	12.695	0.00019	3.7208	0.00022	ctrl - P10; ctrl - P11; ctrl - P3; ctrl - P8; P11 - P10; P2 - P10; P11 - P8; P2 - P3; P2 - P8
TC0X00010959.hg.1	TC0X00010959.hg.1	NA	NA	NA	12.59	0.000198	3.7034	0.000229	ctrl - P10; ctrl - P3; ctrl - P8; P11 - P10; P2 - P10; P11 - P3; P11 - P8; P2 - P3; P2 - P8
TC1500007336.hg.1	TC1500007336.hg.1	chuzerbu.aAug10-unspliced	chuzerbu	NA	12.565	0.0002	3.6993	0.000231	ctrl - P10; ctrl - P11; ctrl - P8; P2 - P10; P3 - P10; P2 - P11; P2 - P3; P2 - P8
TC0Y00006454.hg.1	TC0Y00006454.hg.1	spoybo.aAug10-unspliced	spoybo	NA	12.55	0.000201	3.6967	0.000233	P10 - ctrl; P11 - ctrl; P10 - P2; P10 - P3; P11 - P2; P11 - P3; P11 - P8; P8 - P2
TC0300008326.hg.1	TC0300008326.hg.1	NA	NA	NA	12.533	0.000202	3.6938	0.000234	ctrl - P10; ctrl - P11; ctrl - P3; ctrl - P8; P11 - P10; P2 - P10; P3 - P10; P2 - P11; P11 - P8; P2 - P3; P2 - P8; P3 - P8
TC1600008324.hg.1	TC1600008324.hg.1	teyzabu.aAug10-unspliced	teyzabu	NA	12.519	0.000203	3.6916	0.000235	ctrl - P10; ctrl - P3; ctrl - P8; P2 - P10; P2 - P11; P11 - P8; P2 - P3; P2 - P8
TC1100007694.hg.1	TC1100007694.hg.1	flerny.aAug10-unspliced	flerny	NA	12.514	0.000204	3.6907	0.000236	ctrl - P10; ctrl - P11; ctrl - P3; ctrl - P8; P2 - P10; P10 - P8; P2 - P11; P11 - P8; P2 - P3; P2 - P8; P3 - P8
TC1000011528.hg.1	TC1000011528.hg.1	NA	NA	NA	12.502	0.000205	3.6887	0.000236	P10 - ctrl; P11 - ctrl; P8 - ctrl; P10 - P2; P10 - P3; P11 - P2; P11 - P3; P8 - P2; P8 - P3
TC0300012214.hg.1	TC0300012214.hg.1	noberbu.aAug10-unspliced	noberbu	NA	12.499	0.000205	3.6881	0.000237	ctrl - P10; ctrl - P11; ctrl - P2; ctrl - P8; P3 - P10; P3 - P11; P3 - P2; P3 - P8
TC0300006725.hg.1	TC0300006725.hg.1	NA	NA	NA	12.494	0.000205	3.6872	0.000237	ctrl - P10; P2 - ctrl; ctrl - P8; P2 - P10; P2 - P11; P11 - P8; P2 - P3; P2 - P8; P3 - P8
TC0100009481.hg.1	TC0100009481.hg.1	wawleybo.aAug10-unspliced	wawleybo	NA	12.481	0.000207	3.6851	0.000238	ctrl - P10; ctrl - P11; ctrl - P3; ctrl - P8; P2 - P10; P2 - P11; P2 - P3; P2 - P8
TC0400010996.hg.1	TC0400010996.hg.1	vogy.aAug10-unspliced	vogy	NA	12.475	0.000207	3.6841	0.000239	ctrl - P10; ctrl - P11; ctrl - P3; ctrl - P8; P2 - P10; P2 - P11; P2 - P3; P2 - P8
TC0400008486.hg.1	TC0400008486.hg.1	spoklawby.bAug10-unspliced	spoklawby	NA	12.467	0.000208	3.6828	0.000239	ctrl - P10; ctrl - P3; ctrl - P8; P11 - P10; P2 - P10; P11 - P3; P11 - P8; P2 - P3; P2 - P8
TC1300008943.hg.1	TC1300008943.hg.1	pleychee.aAug10-unspliced	pleychee	NA	12.464	0.000208	3.6822	0.000239	ctrl - P10; ctrl - P3; ctrl - P8; P11 - P10; P2 - P10; P2 - P11; P11 - P8; P2 - P3; P2 - P8; P3 - P8
TC2000008426.hg.1	TC2000008426.hg.1	NA	NA	NA	12.432	0.00021	3.6768	0.000242	P11 - ctrl; P2 - ctrl; P11 - P10; P2 - P10; P11 - P3; P11 - P8; P2 - P3; P2 - P8
TC1000010412.hg.1	TC1000010412.hg.1	plokleybu.aAug10-unspliced	plokleybu	NA	12.43	0.000211	3.6765	0.000242	ctrl - P10; ctrl - P11; ctrl - P3; ctrl - P8; P2 - P10; P2 - P11; P2 - P3; P2 - P8
TC1200011149.hg.1	TC1200011149.hg.1	cheykuby.aAug10-unspliced	cheykuby	NA	12.413	0.000212	3.6737	0.000244	ctrl - P11; P2 - ctrl; ctrl - P8; P10 - P11; P2 - P10; P2 - P11; P3 - P11; P2 - P8; P3 - P8
TC1500007338.hg.1	TC1500007338.hg.1	meezo.aAug10-unspliced	meezo	NA	12.412	0.000212	3.6734	0.000244	ctrl - P10; ctrl - P11; P2 - ctrl; ctrl - P8; P2 - P10; P2 - P11; P2 - P3; P2 - P8
TC0100013972.hg.1	TC0100013972.hg.1	steypobu.aAug10-unspliced	steypobu	NA	12.382	0.000215	3.6684	0.000247	ctrl - P10; ctrl - P11; P2 - ctrl; ctrl - P3; ctrl - P8; P2 - P10; P2 - P11; P2 - P3; P2 - P8
TC1300006739.hg.1	TC1300006739.hg.1	NA	NA	NA	12.376	0.000215	3.6675	0.000247	P10 - ctrl; P11 - ctrl; P8 - ctrl; P10 - P2; P10 - P3; P11 - P2; P11 - P3; P8 - P2; P8 - P3
TC1100007369.hg.1	TC1100007369.hg.1	karure.aAug10-unspliced	karure	NA	12.359	0.000216	3.6646	0.000248	ctrl - P10; ctrl - P3; ctrl - P8; P11 - P10; P2 - P10; P2 - P11; P11 - P8; P2 - P3; P2 - P8

TC0700013296.hg.1	TC0700013296.hg.1	mazeyby.aAug10-unspliced	mazeyby	NA	12.317	0.00022	3.6575	0.000252	ctrl - P10; ctrl - P11; ctrl - P3; ctrl - P8; P2 - P10; P3 - P10; P2 - P11; P2 - P3; P2 - P8; P3 - P8
TC0Y00007290.hg.1	TC0Y00007290.hg.1	vubo.aAug10-unspliced	vubo	NA	12.307	0.000221	3.6558	0.000253	ctrl - P10; ctrl - P11; ctrl - P2; ctrl - P8; P11 - P10; P3 - P10; P3 - P11; P3 - P2; P3 - P8
TC1000010388.hg.1	TC1000010388.hg.1	NA	NA	NA	12.296	0.000222	3.6538	0.000254	P2 - ctrl; P3 - ctrl; P2 - P10; P3 - P10; P2 - P11; P3 - P11; P2 - P8; P3 - P8
TC0200008282.hg.1	TC0200008282.hg.1	PTCD3andSNORD94.vgAug10-unspliced	PTCD3andSNORD94	NA	12.265	0.000225	3.6485	0.000257	ctrl - P10; ctrl - P11; ctrl - P3; ctrl - P8; P11 - P10; P2 - P10; P11 - P8; P2 - P3; P2 - P8; P3 - P8
TC0800009770.hg.1	TC0800009770.hg.1	ENST00000623639	RP11-108A14.1		12.26	0.000225	3.6478	0.000258	ctrl - P10; ctrl - P8; P11 - P10; P2 - P10; P3 - P10; P2 - P11; P3 - P11; P11 - P8; P2 - P8; P3 - P8
TC0700010121.hg.1	TC0700010121.hg.1	blukeeby.aAug10-unspliced	blukeeby	NA	12.102	0.00024	3.6206	0.000274	ctrl - P10; ctrl - P8; P2 - P10; P3 - P10; P2 - P11; P3 - P11; P2 - P8; P3 - P8
TC0700010124.hg.1	TC0700010124.hg.1	klukeeby.aAug10-unspliced	klukeeby	NA	12.102	0.00024	3.6206	0.000274	ctrl - P10; ctrl - P8; P2 - P10; P3 - P10; P2 - P11; P3 - P11; P2 - P8; P3 - P8
TC0300013422.hg.1	TC0300013422.hg.1	romera.aAug10-unspliced	romera	NA	12.09	0.000241	3.6186	0.000275	ctrl - P10; ctrl - P11; ctrl - P3; ctrl - P8; P2 - P10; P10 - P8; P2 - P11; P11 - P8; P2 - P3; P2 - P8; P3 - P8
TC0500011302.hg.1	TC0500011302.hg.1	NA	NA	NA	12.055	0.000244	3.6126	0.000279	ctrl - P10; ctrl - P3; ctrl - P8; P11 - P10; P2 - P10; P2 - P11; P11 - P8; P2 - P3; P2 - P8
TC0200008540.hg.1	TC0200008540.hg.1	NR_103732	LOC100506076	1.01E+08	12.04	0.000246	3.6099	0.000281	ctrl - P10; ctrl - P11; ctrl - P8; P2 - P10; P3 - P10; P2 - P11; P3 - P11; P2 - P8; P3 - P8
TC1000007372.hg.1	TC1000007372.hg.1	NA	NA	NA	12.028	0.000247	3.6078	0.000282	P10 - ctrl; P2 - ctrl; P3 - ctrl; P10 - P11; P2 - P10; P10 - P8; P2 - P11; P3 - P11; P2 - P8; P3 - P8
TC0100009048.hg.1	TC0100009048.hg.1	hsa_circ_0000092	RPL5	NA	12.01	0.000248	3.6048	0.000284	ctrl - P10; ctrl - P11; ctrl - P3; ctrl - P8; P2 - P10; P2 - P11; P2 - P3; P2 - P8
TC0400007939.hg.1	TC0400007939.hg.1	lergar.aAug10-unspliced	lergar	NA	12.002	0.000249	3.6034	0.000284	ctrl - P10; ctrl - P11; ctrl - P8; P2 - P10; P2 - P11; P11 - P8; P2 - P3; P2 - P8; P3 - P8
TC1700010911.hg.1	TC1700010911.hg.1	NA	NA	NA	12.001	0.000249	3.6032	0.000284	ctrl - P10; ctrl - P11; ctrl - P3; ctrl - P8; P2 - P10; P2 - P11; P2 - P3; P2 - P8; P3 - P8
TC1800008623.hg.1	TC1800008623.hg.1	paplor.aAug10-unspliced	paplor	NA	11.945	0.000255	3.5934	0.000291	ctrl - P10; ctrl - P11; ctrl - P3; ctrl - P8; P2 - P10; P2 - P11; P2 - P3; P2 - P8
TC0100011367.hg.1	TC0100011367.hg.1	royaro.aAug10-unspliced	royaro	NA	11.942	0.000255	3.593	0.000291	ctrl - P10; ctrl - P11; ctrl - P3; ctrl - P8; P2 - P10; P3 - P10; P2 - P11; P2 - P8; P3 - P8
TC0100009846.hg.1	TC0100009846.hg.1	PDE4DIP.wgAug10-unspliced	PDE4DIP	NA	11.932	0.000256	3.5911	0.000292	ctrl - P10; ctrl - P11; ctrl - P3; ctrl - P8; P2 - P10; P3 - P10; P2 - P11; P2 - P3; P2 - P8; P3 - P8
TC2200007630.hg.1	TC2200007630.hg.1	slufer.aAug10-unspliced	slufer	NA	11.854	0.000265	3.5775	0.000301	ctrl - P10; ctrl - P11; P2 - ctrl; ctrl - P8; P2 - P10; P2 - P11; P2 - P3; P2 - P8
TC0400008145.hg.1	TC0400008145.hg.1	gerbubo.aAug10-unspliced	gerbubo	NA	11.829	0.000267	3.5732	0.000304	ctrl - P10; P11 - ctrl; ctrl - P3; P11 - P10; P2 - P10; P11 - P2; P11 - P3; P11 - P8; P2 - P3; P2 - P8
TC2100007087.hg.1	TC2100007087.hg.1	saver.aAug10-unspliced	saver	NA	11.806	0.00027	3.5691	0.000307	ctrl - P10; ctrl - P11; ctrl - P3; ctrl - P8; P2 - P10; P2 - P11; P2 - P3; P2 - P8
TC0900009842.hg.1	TC0900009842.hg.1	NA	NA	NA	11.802	0.00027	3.5685	0.000307	ctrl - P10; ctrl - P3; ctrl - P8; P11 - P10; P2 - P10; P2 - P11; P11 - P8; P2 - P3; P2 - P8; P3 - P8
TC1300008425.hg.1	TC1300008425.hg.1	sharnor.aAug10-unspliced	sharnor	NA	11.772	0.000273	3.5633	0.000311	ctrl - P10; ctrl - P11; ctrl - P3; ctrl - P8; P2 - P10; P3 - P10; P2 - P11; P2 - P8; P3 - P8
TC1200007996.hg.1	TC1200007996.hg.1	swyner.aAug10-unspliced	swyner	NA	11.764	0.000274	3.5617	0.000312	ctrl - P10; ctrl - P11; ctrl - P3; ctrl - P8; P2 - P10; P2 - P11; P11 - P3; P11 - P8; P2 - P3; P2 - P8
TC0300008317.hg.1	TC0300008317.hg.1	poysweyby.aAug10-unspliced	poysweyby	NA	11.758	0.000275	3.5608	0.000312	ctrl - P10; ctrl - P11; P2 - ctrl; ctrl - P3; ctrl - P8; P2 - P10; P2 - P11; P2 - P3; P2 - P8
TC0300012128.hg.1	TC0300012128.hg.1	NA	NA	NA	11.758	0.000275	3.5607	0.000312	ctrl - P10; ctrl - P11; ctrl - P3; ctrl - P8; P2 - P10; P2 - P11; P11 - P8; P2 - P3; P2 - P8
TC0200015226.hg.1	TC0200015226.hg.1	HIBCH.qAug10-unspliced	HIBCH	NA	11.756	0.000275	3.5603	0.000312	ctrl - P10; ctrl - P11; ctrl - P3; ctrl - P8; P11 - P10; P2 - P10; P3 - P10; P2 - P11; P11 - P8; P2 - P3; P2 - P8
TC1300006991.hg.1	TC1300006991.hg.1	plupa.aAug10-unspliced	plupa	NA	11.74	0.000277	3.5574	0.000314	ctrl - P10; ctrl - P11; ctrl - P8; P2 - P10; P2 - P11; P2 - P3; P2 - P8; P3 - P8
TC0700008968.hg.1	TC0700008968.hg.1	nukemu.aAug10-unspliced	nukemu	NA	11.721	0.000279	3.5542	0.000316	P10 - ctrl; P11 - ctrl; P8 - ctrl; P10 - P2; P10 - P3; P11 - P2; P11 - P3; P8 - P2; P8 - P3
TC0X00009141.hg.1	TC0X00009141.hg.1	beevar.aAug10-unspliced	beevar	NA	11.691	0.000283	3.5488	0.00032	ctrl - P10; ctrl - P11; ctrl - P3; ctrl - P8; P2 - P10; P2 - P11; P2 - P3; P2 - P8
TC0400007130.hg.1	TC0400007130.hg.1	gloygluby.aAug10-unspliced	gloygluby	NA	11.682	0.000284	3.5473	0.000321	ctrl - P11; P2 - ctrl; ctrl - P8; P2 - P10; P3 - P10; P2 - P11; P3 - P11; P2 - P8; P3 - P8
TC1700010966.hg.1	TC1700010966.hg.1	ENST00000580372.1	RP11-6N17.6		11.625	0.00029	3.5372	0.000329	P10 - ctrl; P11 - ctrl; P3 - ctrl; P8 - ctrl; P10 - P2; P11 - P2; P3 - P2; P8 - P2
TC0100011094.hg.1	TC0100011094.hg.1	wawkorbo.aAug10-unspliced	wawkorbo	NA	11.622	0.000291	3.5366	0.000329	ctrl - P10; ctrl - P3; ctrl - P8; P11 - P10; P2 - P10; P11 - P3; P11 - P8; P2 - P3; P2 - P8

TC1800007421.hg.1	TC1800007421.hg.1	neeser.aAug10-unspliced	neeser	NA	11.604	0.000293	3.5333	0.000331	ctrl - P10; ctrl - P11; ctrl - P3; ctrl - P8; P11 - P10; P2 - P10; P2 - P11; P11 - P3; P11 - P8; P2 - P3; P2 - P8
TC0700009340.hg.1	TC0700009340.hg.1	berbley.aAug10-unspliced	berbley	NA	11.529	0.000302	3.5201	0.000341	ctrl - P10; ctrl - P11; ctrl - P3; ctrl - P8; P2 - P10; P2 - P11; P2 - P3; P2 - P8; P3 - P8
TC0800009983.hg.1	TC0800009983.hg.1	shorker.aAug10-unspliced	shorker	NA	11.513	0.000304	3.5172	0.000343	ctrl - P10; ctrl - P11; ctrl - P3; ctrl - P8; P2 - P10; P2 - P11; P11 - P8; P2 - P3; P2 - P8; P3 - P8
TC2200008471.hg.1	TC2200008471.hg.1	ENST00000526089.1	RNF185-AS1		11.512	0.000304	3.517	0.000343	P10 - ctrl; P11 - ctrl; P8 - ctrl; P10 - P2; P10 - P3; P11 - P2; P11 - P3; P8 - P2; P8 - P3
TC1000009284.hg.1	TC1000009284.hg.1	borma.aAug10-unspliced	borma	NA	11.48	0.000308	3.5112	0.000348	ctrl - P10; ctrl - P8; P11 - P10; P2 - P10; P3 - P10; P11 - P8; P2 - P3; P2 - P8; P3 - P8
TC0100014024.hg.1	TC0100014024.hg.1	NA	NA	NA	11.473	0.000309	3.51	0.000349	ctrl - P10; ctrl - P11; ctrl - P3; ctrl - P8; P2 - P10; P2 - P11; P2 - P3; P2 - P8
TC0700007141.hg.1	TC0700007141.hg.1	NA	NA	NA	11.447	0.000312	3.5054	0.000352	ctrl - P10; ctrl - P3; ctrl - P8; P11 - P10; P2 - P10; P3 - P10; P11 - P8; P2 - P3; P2 - P8; P3 - P8
TC1900009179.hg.1	TC1900009179.hg.1	feezoy.aAug10-unspliced	feezoy	NA	11.44	0.000313	3.5041	0.000353	ctrl - P10; ctrl - P11; ctrl - P3; ctrl - P8; P2 - P10; P2 - P11; P2 - P3; P2 - P8
TC1200007155.hg.1	TC1200007155.hg.1	snawchar.aAug10-unspliced	snawchar	NA	11.434	0.000314	3.503	0.000354	ctrl - P10; ctrl - P11; ctrl - P3; ctrl - P8; P2 - P10; P2 - P11; P2 - P3; P2 - P8
TC0X00007403.hg.1	TC0X00007403.hg.1	beetoy.aAug10-unspliced	beetoy	NA	11.417	0.000316	3.4999	0.000356	ctrl - P10; ctrl - P11; P2 - ctrl; ctrl - P8; P2 - P10; P3 - P10; P2 - P11; P3 - P11; P2 - P8; P3 - P8
TC0500009416.hg.1	TC0500009416.hg.1	NA	NA	NA	11.381	0.000321	3.4934	0.000361	ctrl - P10; ctrl - P11; ctrl - P3; ctrl - P8; P2 - P10; P2 - P11; P11 - P8; P2 - P3; P2 - P8
TC0900007500.hg.1	TC0900007500.hg.1	fleyshee.aAug10-unspliced	fleyshee	NA	11.379	0.000321	3.4931	0.000362	ctrl - P10; ctrl - P11; ctrl - P8; P2 - P10; P3 - P10; P2 - P11; P3 - P11; P2 - P3; P2 - P8; P3 - P8
TC2200008352.hg.1	TC2200008352.hg.1	tovaw.aAug10-unspliced	tovaw	NA	11.371	0.000322	3.4915	0.000363	ctrl - P10; ctrl - P11; ctrl - P3; ctrl - P8; P11 - P10; P2 - P10; P2 - P11; P11 - P8; P2 - P3; P2 - P8
TC1100013095.hg.1	TC1100013095.hg.1	NR_126004	LOC283140	283140	11.358	0.000324	3.4892	0.000364	ctrl - P10; ctrl - P2; ctrl - P8; P11 - P10; P3 - P10; P11 - P2; P11 - P8; P3 - P2; P3 - P8
TC0500010850.hg.1	TC0500010850.hg.1	jeyflu.aAug10-unspliced	jeyflu	NA	11.331	0.000328	3.4844	0.000368	ctrl - P10; P3 - ctrl; ctrl - P8; P2 - P10; P3 - P10; P3 - P11; P3 - P2; P2 - P8; P3 - P8
TC0200014724.hg.1	TC0200014724.hg.1	voly.aAug10-unspliced	voly	NA	11.321	0.000329	3.4826	0.00037	ctrl - P10; ctrl - P11; ctrl - P8; P2 - P10; P3 - P10; P2 - P11; P2 - P3; P2 - P8; P3 - P8
TC0500009020.hg.1	TC0500009020.hg.1	NM_001112724	STK32A	5q32	11.304	0.000332	3.4795	0.000372	ctrl - P10; P11 - ctrl; P2 - ctrl; ctrl - P8; P11 - P10; P2 - P10; P3 - P10; P11 - P8; P2 - P8; P3 - P8
TC2000006722.hg.1	TC2000006722.hg.1	syto.aAug10-unspliced	syto	NA	11.296	0.000333	3.478	0.000373	ctrl - P10; ctrl - P11; ctrl - P3; ctrl - P8; P2 - P10; P2 - P11; P2 - P3; P2 - P8
TC0100013271.hg.1	TC0100013271.hg.1	rawabo.aAug10-unspliced	rawabo	NA	11.285	0.000334	3.4759	0.000375	ctrl - P10; ctrl - P11; ctrl - P8; P2 - P10; P3 - P10; P2 - P11; P2 - P3; P2 - P8; P3 - P8
TC0200007084.hg.1	TC0200007084.hg.1	tapubo.aAug10-unspliced	tapubo	NA	11.261	0.000338	3.4716	0.000379	ctrl - P10; ctrl - P8; P11 - P10; P2 - P10; P3 - P10; P11 - P8; P2 - P8; P3 - P8
TC0600009766.hg.1	TC0600009766.hg.1	NA	NA	NA	11.257	0.000338	3.4709	0.000379	P10 - ctrl; P2 - ctrl; P8 - ctrl; P2 - P10; P2 - P11; P2 - P3; P2 - P8
TC1500010548.hg.1	TC1500010548.hg.1	geetee.aAug10-unspliced	geetee	NA	11.243	0.00034	3.4682	0.000381	ctrl - P10; ctrl - P3; ctrl - P8; P11 - P10; P2 - P10; P11 - P3; P11 - P8; P2 - P3; P2 - P8
TC0400008062.hg.1	TC0400008062.hg.1	NA	NA	NA	11.166	0.000351	3.4543	0.000393	ctrl - P10; ctrl - P11; ctrl - P3; ctrl - P8; P2 - P10; P2 - P11; P2 - P3; P2 - P8
TC0400011109.hg.1	TC0400011109.hg.1	kygar.aAug10-unspliced	kygar	NA	11.157	0.000353	3.4526	0.000395	ctrl - P10; ctrl - P11; ctrl - P8; P2 - P10; P3 - P10; P2 - P11; P3 - P11; P2 - P3; P2 - P8; P3 - P8
TC0400006964.hg.1	TC0400006964.hg.1	NA	NA	NA	11.136	0.000356	3.4487	0.000398	ctrl - P10; ctrl - P11; ctrl - P3; ctrl - P8; P2 - P10; P3 - P10; P2 - P11; P11 - P8; P2 - P3; P2 - P8; P3 - P8
TC1200009979.hg.1	TC1200009979.hg.1	NA	NA	NA	11.128	0.000357	3.4472	0.000399	P10 - ctrl; P3 - ctrl; P8 - ctrl; P10 - P11; P10 - P2; P8 - P11; P3 - P2; P8 - P2; P8 - P3
TC1300007797.hg.1	TC1300007797.hg.1	zeyzoy.aAug10-unspliced	zeyzoy	NA	11.123	0.000358	3.4464	0.0004	ctrl - P10; ctrl - P11; ctrl - P3; ctrl - P8; P2 - P10; P10 - P8; P2 - P11; P11 - P8; P2 - P3; P2 - P8; P3 - P8
TC0100011195.hg.1	TC0100011195.hg.1	zorkobu.aAug10-unspliced	zorkobu	NA	11.109	0.00036	3.4438	0.000402	ctrl - P10; ctrl - P11; ctrl - P3; ctrl - P8; P2 - P10; P3 - P10; P2 - P11; P2 - P8
TC1200009256.hg.1	TC1200009256.hg.1	borgawby.aAug10-unspliced	borgawby	NA	11.079	0.000365	3.4382	0.000407	ctrl - P10; ctrl - P11; ctrl - P3; ctrl - P8; P2 - P10; P3 - P10; P2 - P11; P2 - P3; P2 - P8; P3 - P8
TC1200012809.hg.1	TC1200012809.hg.1	NA	NA	NA	11.013	0.000375	3.426	0.000419	ctrl - P10; ctrl - P11; ctrl - P3; ctrl - P8; P2 - P10; P2 - P11; P2 - P3; P2 - P8; P3 - P8
TC1200010932.hg.1	TC1200010932.hg.1	voychy.aAug10-unspliced	voychy	NA	10.978	0.000381	3.4195	0.000425	ctrl - P10; ctrl - P11; ctrl - P3; ctrl - P8; P2 - P10; P2 - P11; P11 - P8; P2 - P3; P2 - P8; P3 - P8
TC0100013196.hg.1	TC0100013196.hg.1	serklor.aAug10-unspliced	serklor	NA	10.938	0.000387	3.412	0.000432	ctrl - P10; ctrl - P8; P2 - P10; P3 - P10; P2 - P11; P2 - P3; P2 - P8; P3 - P8

TC1800007191.hg.1	TC1800007191.hg.1	ENST00000623380	RP11-693M3.1		10.929	0.000389	3.4104	0.000433	ctrl - P10; ctrl - P2; ctrl - P3; ctrl - P8; P11 - P10; P3 - P10; P11 - P2; P11 - P8; P2 - P8; P3 - P8
TC0400007719.hg.1	TC0400007719.hg.1	uc062wzu.1	SNORA62		10.858	0.000401	3.3972	0.000446	ctrl - P10; ctrl - P11; ctrl - P8; P2 - P10; P3 - P10; P2 - P11; P2 - P3; P2 - P8
TC0200011191.hg.1	TC0200011191.hg.1	NA	NA	NA	10.849	0.000402	3.3954	0.000448	ctrl - P10; ctrl - P3; ctrl - P8; P11 - P10; P2 - P10; P11 - P8; P2 - P3; P2 - P8
TC0100012765.hg.1	TC0100012765.hg.1	snawkubo.aAug10-unspliced	snawkubo	NA	10.846	0.000403	3.3948	0.000449	ctrl - P10; ctrl - P3; ctrl - P8; P11 - P10; P2 - P10; P2 - P11; P11 - P8; P2 - P3; P2 - P8; P3 - P8
TC0300010995.hg.1	TC0300010995.hg.1	speeswerby.aAug10-unspliced	speeswerby	NA	10.832	0.000405	3.3922	0.000451	ctrl - P10; ctrl - P11; ctrl - P8; P2 - P10; P3 - P10; P2 - P11; P3 - P11; P2 - P8; P3 - P8
TC0600007110.hg.1	TC0600007110.hg.1	NA	NA	NA	10.812	0.000409	3.3885	0.000455	P10 - ctrl; P2 - ctrl; P3 - ctrl; P8 - ctrl; P10 - P11; P2 - P11; P3 - P11; P8 - P11; P3 - P2
TC1900009545.hg.1	TC1900009545.hg.1	NA	NA	NA	10.751	0.00042	3.377	0.000467	P10 - ctrl; P11 - ctrl; P8 - ctrl; P10 - P2; P10 - P3; P11 - P2; P11 - P3; P8 - P2; P8 - P3
TC0200007158.hg.1	TC0200007158.hg.1	gleyjawbo.aAug10-unspliced	gleyjawbo	NA	10.748	0.00042	3.3765	0.000467	P10 - ctrl; P3 - ctrl; P8 - ctrl; P10 - P11; P10 - P2; P3 - P11; P8 - P11; P3 - P2; P8 - P2
TC1800008622.hg.1	TC1800008622.hg.1	shawsu.aAug10-unspliced	shawsu	NA	10.747	0.00042	3.3763	0.000467	ctrl - P10; ctrl - P3; ctrl - P8; P2 - P10; P2 - P11; P2 - P3; P2 - P8
TC2000006689.hg.1	TC2000006689.hg.1	blosobu.aAug10-unspliced	blosobu	NA	10.728	0.000424	3.3727	0.000471	P10 - ctrl; P3 - ctrl; P8 - ctrl; P10 - P11; P10 - P2; P3 - P11; P3 - P2; P8 - P2
TC1200007016.hg.1	TC1200007016.hg.1	kugyby.aAug10-unspliced	kugyby	NA	10.726	0.000424	3.3721	0.000471	ctrl - P10; P2 - ctrl; ctrl - P8; P11 - P10; P2 - P10; P3 - P10; P2 - P11; P11 - P8; P2 - P3; P2 - P8; P3 - P8
TC0900008687.hg.1	TC0900008687.hg.1	bleyplerbu.aAug10-unspliced	bleyplerbu	NA	10.723	0.000425	3.3717	0.000471	ctrl - P10; ctrl - P11; ctrl - P3; ctrl - P8; P2 - P10; P2 - P11; P2 - P3; P2 - P8
TC0100010118.hg.1	TC0100010118.hg.1	NR_030281	MIR92B	693235	10.675	0.000434	3.3625	0.000481	ctrl - P10; ctrl - P2; ctrl - P3; ctrl - P8; P11 - P10; P11 - P2; P11 - P3; P11 - P8
HTA2-pos-47420544_st	23150838	NA	NA	NA	10.649	0.000439	3.3577	0.000486	ctrl - P10; ctrl - P3; ctrl - P8; P2 - P10; P2 - P11; P11 - P8; P2 - P3; P2 - P8
TC1100012689.hg.1	TC1100012689.hg.1	spolorby.aAug10-unspliced	spolorby	NA	10.58	0.000452	3.3444	0.000501	ctrl - P10; ctrl - P3; ctrl - P8; P2 - P10; P2 - P11; P11 - P8; P2 - P3; P2 - P8
TC0300009039.hg.1	TC0300009039.hg.1	uc062opo.1	RNU7-47P	NA	10.566	0.000455	3.3418	0.000504	ctrl - P10; ctrl - P11; ctrl - P8; P2 - P10; P3 - P10; P2 - P11; P3 - P11; P2 - P8; P3 - P8
TC0400008057.hg.1	TC0400008057.hg.1	gagy.aAug10-unspliced	gagy	NA	10.557	0.000457	3.3401	0.000506	ctrl - P10; ctrl - P11; ctrl - P8; P2 - P10; P3 - P10; P2 - P11; P3 - P11; P2 - P3; P2 - P8; P3 - P8
TC0100014580.hg.1	TC0100014580.hg.1	NR_029707	MIR186	406962	10.552	0.000458	3.339	0.000507	ctrl - P10; ctrl - P11; ctrl - P3; ctrl - P8; P2 - P10; P2 - P11; P2 - P3; P2 - P8; P3 - P8
TC0300013282.hg.1	TC0300013282.hg.1	uc062qgt.1	RNA5SP149	NA	10.541	0.00046	3.3371	0.000509	P11 - ctrl; P2 - ctrl; P3 - ctrl; P11 - P10; P2 - P10; P3 - P10; P2 - P11; P11 - P8; P2 - P3; P2 - P8
TC0X00009868.hg.1	TC0X00009868.hg.1	sneegy.aAug10-unspliced	sneegy	NA	10.529	0.000463	3.3347	0.000511	ctrl - P10; ctrl - P11; ctrl - P3; ctrl - P8; P2 - P10; P2 - P11; P2 - P3; P2 - P8
TC0700013109.hg.1	TC0700013109.hg.1	wahira.aAug10-unspliced	wahira	NA	10.512	0.000466	3.3314	0.000515	ctrl - P10; ctrl - P11; ctrl - P8; P2 - P10; P3 - P10; P2 - P11; P2 - P3; P2 - P8; P3 - P8
TC0200008244.hg.1	TC0200008244.hg.1	hsa_circ_0001034	TGOLN2	NA	10.485	0.000472	3.3261	0.000521	P10 - ctrl; P11 - ctrl; P8 - ctrl; P10 - P2; P10 - P3; P11 - P2; P11 - P3; P8 - P2; P8 - P3
TC0600010865.hg.1	TC0600010865.hg.1	rarblor.aAug10-unspliced	rarblor	NA	10.442	0.000481	3.318	0.000531	ctrl - P10; ctrl - P11; ctrl - P8; P2 - P10; P3 - P10; P2 - P11; P11 - P8; P2 - P8; P3 - P8
TC0800011711.hg.1	TC0800011711.hg.1	gably.aAug10-unspliced	gably	NA	10.384	0.000494	3.3066	0.000544	ctrl - P10; ctrl - P11; ctrl - P3; ctrl - P8; P2 - P10; P2 - P11; P2 - P3; P2 - P8
TC0100008293.hg.1	TC0100008293.hg.1	siyuro.aAug10-unspliced	siyuro	NA	10.383	0.000494	3.3066	0.000544	ctrl - P10; ctrl - P11; ctrl - P3; ctrl - P8; P2 - P10; P2 - P11; P2 - P3; P2 - P8
TC0400011467.hg.1	TC0400011467.hg.1	swersnoby.aAug10-unspliced	swersnoby	NA	10.354	0.0005	3.3009	0.000551	ctrl - P10; ctrl - P11; ctrl - P3; ctrl - P8; P2 - P10; P2 - P11; P11 - P8; P2 - P3; P2 - P8; P3 - P8
TC0200011198.hg.1	TC0200011198.hg.1	lergubo.aAug10-unspliced	lergubo	NA	10.27	0.000519	3.2844	0.000572	ctrl - P10; ctrl - P11; ctrl - P3; ctrl - P8; P2 - P10; P2 - P11; P11 - P8; P2 - P8; P3 - P8
TC1700006669.hg.1	TC1700006669.hg.1	NR_034082	LOC100130950	1E+08	10.268	0.00052	3.284	0.000573	ctrl - P10; ctrl - P3; ctrl - P8; P11 - P10; P2 - P10; P11 - P3; P11 - P8; P2 - P3; P2 - P8
TC0600007890.hg.1	TC0600007890.hg.1	smarjaw.aAug10-unspliced	smarjaw	NA	10.26	0.000522	3.2825	0.000574	ctrl - P11; P2 - ctrl; ctrl - P8; P2 - P10; P2 - P11; P3 - P11; P2 - P3; P2 - P8; P3 - P8
TC0900011480.hg.1	TC0900011480.hg.1	jugar.aAug10-unspliced	jugar	NA	10.256	0.000523	3.2817	0.000575	ctrl - P10; ctrl - P11; ctrl - P3; ctrl - P8; P11 - P10; P2 - P10; P3 - P10; P11 - P8; P2 - P8; P3 - P8
TC0500008832.hg.1	TC0500008832.hg.1	rawsnobu.aAug10-unspliced	rawsnobu	NA	10.234	0.000528	3.2773	0.000581	ctrl - P11; P2 - ctrl; ctrl - P8; P10 - P11; P2 - P10; P2 - P11; P3 - P11; P2 - P3; P2 - P8
TC1700011916.hg.1	TC1700011916.hg.1	fleyskee.aAug10-unspliced	fleyskee	NA	10.219	0.000532	3.2744	0.000585	ctrl - P10; ctrl - P11; ctrl - P3; ctrl - P8; P2 - P10; P3 - P10; P2 - P11; P2 - P3; P2 - P8; P3 - P8

TC0300011340.hg.1	TC0300011340.hg.1	skeynybo.aAug10-unspliced	skeynybo	NA	10.211	0.000533	3.273	0.000586	ctrl - P10; ctrl - P11; ctrl - P8; P2 - P10; P3 - P10; P2 - P11; P2 - P3; P2 - P8; P3 - P8
TC1100012429.hg.1	TC1100012429.hg.1	klawchey.aAug10-unspliced	klawchey	NA	10.207	0.000535	3.272	0.000587	ctrl - P10; ctrl - P8; P11 - P10; P2 - P10; P3 - P10; P2 - P11; P11 - P8; P2 - P3; P2 - P8; P3 - P8
TC1200008164.hg.1	TC1200008164.hg.1	skoyskarby.aAug10-unspliced	skoyskarby	NA	10.194	0.000538	3.2695	0.00059	ctrl - P10; ctrl - P11; ctrl - P3; ctrl - P8; P2 - P10; P2 - P11; P2 - P3; P2 - P8
TC0600007898.hg.1	TC0600007898.hg.1	lugloby.aAug10-unspliced	lugloby	NA	10.189	0.000539	3.2686	0.000591	ctrl - P10; ctrl - P11; ctrl - P3; ctrl - P8; P2 - P10; P2 - P11; P2 - P3; P2 - P8; P3 - P8
TC0700008380.hg.1	TC0700008380.hg.1	smusheyby.aAug10-unspliced	smusheyby	NA	10.18	0.000541	3.2667	0.000594	ctrl - P10; ctrl - P11; ctrl - P8; P2 - P10; P3 - P10; P2 - P11; P3 - P11; P2 - P8; P3 - P8
TC0900010579.hg.1	TC0900010579.hg.1	muploybu.aAug10-unspliced	muploybu	NA	10.145	0.000553	3.2598	0.000603	ctrl - P10; ctrl - P11; ctrl - P8; P2 - P10; P3 - P10; P2 - P11; P2 - P8; P3 - P8
TC0700010990.hg.1	TC0700010990.hg.1	glawklarby.aAug10-unspliced	glawklarby	NA	10.11	0.000559	3.2529	0.000612	ctrl - P10; ctrl - P11; ctrl - P3; ctrl - P8; P2 - P10; P11 - P8; P2 - P3; P2 - P8
TC0300009744.hg.1	TC0300009744.hg.1	NA	NA	NA	10.017	0.000583	3.2345	0.000638	ctrl - P10; ctrl - P11; ctrl - P3; ctrl - P8; P2 - P10; P2 - P11; P2 - P3; P2 - P8
TC1300007776.hg.1	TC1300007776.hg.1	dawsharbu.aAug10-unspliced	dawsharbu	NA	10.005	0.000586	3.232	0.000642	ctrl - P10; ctrl - P11; ctrl - P3; ctrl - P8; P2 - P10; P2 - P11; P2 - P3; P2 - P8
TC0200010481.hg.1	TC0200010481.hg.1	NR_003058	SNORD70	2q33.1	10.001	0.000587	3.2312	0.000643	ctrl - P10; ctrl - P3; P11 - P10; P2 - P10; P11 - P3; P11 - P8; P2 - P3
TC2000008219.hg.1	TC2000008219.hg.1	statu.aAug10-unspliced	statu	NA	9.9795	0.000593	3.227	0.000649	ctrl - P10; ctrl - P11; ctrl - P3; ctrl - P8; P2 - P10; P2 - P11; P2 - P3; P2 - P8
TC0700012821.hg.1	TC0700012821.hg.1	skuku.aAug10-unspliced	skuku	NA	9.9788	0.000593	3.2268	0.000649	ctrl - P10; ctrl - P3; ctrl - P8; P2 - P10; P2 - P11; P11 - P8; P2 - P3; P2 - P8; P3 - P8
TC0100011422.hg.1	TC0100011422.hg.1	kyply.aAug10-unspliced	kyply	NA	9.9308	0.000606	3.2172	0.000663	ctrl - P10; ctrl - P11; ctrl - P3; ctrl - P8; P2 - P10; P2 - P11; P2 - P3; P2 - P8
TC0100009227.hg.1	TC0100009227.hg.1	dymerbo.aAug10-unspliced	dymerbo	NA	9.9258	0.000608	3.2162	0.000664	P2 - ctrl; ctrl - P8; P2 - P10; P3 - P10; P2 - P11; P11 - P8; P2 - P8; P3 - P8
TC0700012166.hg.1	TC0700012166.hg.1	snawblee.aAug10-unspliced	snawblee	NA	9.9182	0.00061	3.2147	0.000666	ctrl - P10; ctrl - P8; P11 - P10; P2 - P10; P2 - P11; P11 - P8; P2 - P3; P2 - P8; P3 - P8
TC1500007780.hg.1	TC1500007780.hg.1	blarta.aAug10-unspliced	blarta	NA	9.9142	0.000611	3.2139	0.000667	P10 - ctrl; P11 - ctrl; P3 - ctrl; P8 - ctrl; P10 - P2; P11 - P2; P3 - P2; P8 - P2
TC0700007871.hg.1	TC0700007871.hg.1	wergoy.aAug10-unspliced	wergoy	NA	9.9103	0.000612	3.2131	0.000668	ctrl - P10; ctrl - P11; ctrl - P3; ctrl - P8; P2 - P10; P2 - P11; P11 - P8; P2 - P3; P2 - P8
TC1200006564.hg.1	TC1200006564.hg.1	DYRK4_.vlAug10-unspliced	DYRK4_	NA	9.8903	0.000618	3.2091	0.000674	ctrl - P10; ctrl - P11; ctrl - P3; ctrl - P8; P2 - P10; P2 - P11; P2 - P8; P3 - P8
TC0200007037.hg.1	TC0200007037.hg.1	sweypybo.aAug10-unspliced	sweypybo	NA	9.89	0.000618	3.209	0.000674	ctrl - P10; ctrl - P8; P11 - P10; P2 - P10; P3 - P10; P11 - P8; P2 - P3; P2 - P8; P3 - P8
TC0100008067.hg.1	TC0100008067.hg.1	marpla.aAug10-unspliced	marpla	NA	9.8805	0.000621	3.2071	0.000676	ctrl - P10; ctrl - P11; ctrl - P3; ctrl - P8; P2 - P10; P2 - P11; P2 - P3; P2 - P8
TC1200010027.hg.1	TC1200010027.hg.1	sheefy.aAug10-unspliced	sheefy	NA	9.8235	0.000637	3.1956	0.000694	ctrl - P10; ctrl - P3; ctrl - P8; P11 - P10; P2 - P10; P11 - P3; P11 - P8; P2 - P8
TC0500006447.hg.1	TC0500006447.hg.1	juskyby.aAug10-unspliced	juskyby	NA	9.8175	0.000639	3.1944	0.000696	ctrl - P10; ctrl - P11; ctrl - P8; P2 - P10; P3 - P10; P2 - P11; P3 - P11; P2 - P8; P3 - P8
TC0900007418.hg.1	TC0900007418.hg.1	hsa_circ_0087128	LOC100133920		9.8173	0.000639	3.1943	0.000696	P10 - ctrl; P3 - ctrl; P8 - ctrl; P10 - P11; P3 - P11; P8 - P11; P3 - P2; P8 - P2
TC1400008344.hg.1	TC1400008344.hg.1	skerzey.aAug10-unspliced	skerzey	NA	9.7857	0.000649	3.1879	0.000706	ctrl - P10; ctrl - P11; ctrl - P3; ctrl - P8; P2 - P10; P2 - P11; P2 - P3; P2 - P8; P3 - P8
TC1100010385.hg.1	TC1100010385.hg.1	NA	NA	NA	9.7767	0.000651	3.1861	0.000708	P2 - ctrl; P10 - P11; P2 - P10; P2 - P11; P2 - P3; P2 - P8
TC2000006477.hg.1	TC2000006477.hg.1	teeja.aAug10-unspliced	teeja	NA	9.7705	0.000653	3.1848	0.00071	ctrl - P10; ctrl - P8; P11 - P10; P2 - P10; P3 - P10; P2 - P11; P2 - P3; P2 - P8; P3 - P8
TC0700008378.hg.1	TC0700008378.hg.1	uc064fmi.1	RNU4-16P	NA	9.7162	0.00067	3.1738	0.000728	ctrl - P10; ctrl - P11; ctrl - P8; P2 - P10; P2 - P11; P2 - P3; P2 - P8; P3 - P8
TC1800007413.hg.1	TC1800007413.hg.1	hohoma.aAug10-unspliced	hohoma	NA	9.7096	0.000672	3.1724	0.00073	P2 - ctrl; ctrl - P3; P2 - P10; P2 - P11; P11 - P3; P2 - P3; P2 - P8
TC0800011319.hg.1	TC0800011319.hg.1	NA	NA	NA	9.669	0.000685	3.1641	0.000744	ctrl - P10; ctrl - P11; ctrl - P8; P2 - P10; P2 - P11; P2 - P3; P2 - P8
TC0600013279.hg.1	TC0600013279.hg.1	fykloby.aAug10-unspliced	fykloby	NA	9.6498	0.000692	3.1602	0.00075	ctrl - P10; ctrl - P11; ctrl - P3; ctrl - P8; P2 - P10; P2 - P11; P11 - P8; P2 - P3; P2 - P8
TC0300010819.hg.1	TC0300010819.hg.1	smaswerby.aAug10	smaswerby	NA	9.644	0.000693	3.159	0.000752	P10 - ctrl; P11 - ctrl; P8 - ctrl; P10 - P2; P10 - P3; P11 - P2; P8 - P2; P8 - P3
TC1200008569.hg.1	TC1200008569.hg.1	teychu.aAug10-unspliced	teychu	NA	9.6388	0.000695	3.1579	0.000754	ctrl - P10; ctrl - P11; ctrl - P3; ctrl - P8; P2 - P10; P2 - P11; P2 - P3; P2 - P8

TC0900009027.hg.1	TC0900009027.hg.1	uc064wsf.1	RNU5D-2P	NA	9.6326	0.0006997	3.1567	0.0007556	P10 - ctrl; P11 - ctrl; P2 - ctrl; P2 - P10; P2 - P11; P11 - P3; P2 - P3; P2 - P8
TC0800006787.hg.1	TC0800006787.hg.1	uc033bcu.2	AC068587.2	NA	9.6215	0.0007011	3.1544	0.0007599	ctrl - P10; ctrl - P3; ctrl - P8; P2 - P10; P2 - P11; P11 - P8; P2 - P3; P2 - P8
TC0300008467.hg.1	TC0300008467.hg.1	NM_020754	ARHGAP31	57514	9.585	0.0007113	3.1469	0.0007772	ctrl - P10; ctrl - P11; ctrl - P2; P3 - P10; P8 - P10; P3 - P11; P8 - P11; P3 - P2; P8 - P2
TC1300007898.hg.1	TC1300007898.hg.1	ENST00000607072.1	RP11-255P5.2		9.5803	0.0007115	3.1459	0.0007773	ctrl - P10; ctrl - P11; ctrl - P8; P2 - P10; P3 - P10; P2 - P11; P2 - P8; P3 - P8
TC0300007208.hg.1	TC0300007208.hg.1	sorfeebo.aAug10-unspliced	sorfeebo	NA	9.5771	0.0007116	3.1452	0.0007774	ctrl - P10; ctrl - P3; ctrl - P8; P11 - P10; P2 - P10; P11 - P3; P11 - P8; P2 - P3; P2 - P8
TC0100007768.hg.1	TC0100007768.hg.1	smarbu.aAug10-unspliced	smarbu	NA	9.5511	0.0007225	3.1399	0.0007784	ctrl - P10; ctrl - P11; ctrl - P3; ctrl - P8; P2 - P10; P2 - P11; P2 - P3; P2 - P8
TC1800006596.hg.1	TC1800006596.hg.1	setaru.aAug10-unspliced	setaru	NA	9.5384	0.0007229	3.1372	0.0007788	ctrl - P10; ctrl - P11; ctrl - P3; ctrl - P8; P2 - P10; P3 - P10; P2 - P11; P2 - P3; P2 - P8; P3 - P8
TC0100015797.hg.1	TC0100015797.hg.1	POGZ.vfAug10-unspliced	POGZ	NA	9.4477	0.0007611	3.1184	0.0008223	ctrl - P10; ctrl - P11; ctrl - P8; P2 - P10; P3 - P10; P2 - P11; P3 - P11; P2 - P3; P2 - P8
TC0100017978.hg.1	TC0100017978.hg.1	sparmeebu.aAug10-unspliced	sparmeebu	NA	9.3299	0.0008066	3.0937	0.00087	ctrl - P10; ctrl - P11; ctrl - P8; P2 - P10; P2 - P11; P2 - P3; P2 - P8
TC0500009011.hg.1	TC0500009011.hg.1	klorflo.aAug10-unspliced	klorflo	NA	9.2869	0.0008223	3.0847	0.0008888	ctrl - P10; ctrl - P11; ctrl - P3; ctrl - P8; P2 - P10; P2 - P11; P2 - P3; P2 - P8
TC1500010107.hg.1	TC1500010107.hg.1	ENST00000624777	RP11-797A18.5		9.2682	0.00083	3.0807	0.0008896	ctrl - P10; ctrl - P11; ctrl - P3; ctrl - P8; P2 - P10; P2 - P11; P2 - P3; P2 - P8
TC0200007238.hg.1	TC0200007238.hg.1	floparbo.aAug10-unspliced	floparbo	NA	9.2384	0.0008443	3.0744	0.0009009	ctrl - P10; ctrl - P11; ctrl - P8; P2 - P10; P3 - P10; P2 - P11; P2 - P8; P3 - P8
TC0100017556.hg.1	TC0100017556.hg.1	geyklor.aAug10-unspliced	geyklor	NA	9.1945	0.0008661	3.0651	0.0009228	ctrl - P10; ctrl - P8; P11 - P10; P2 - P10; P3 - P10; P2 - P11; P11 - P8; P2 - P3; P2 - P8; P3 - P8
TC0600013353.hg.1	TC0600013353.hg.1	sparfla.aAug10-unspliced	sparfla	NA	9.1658	0.0008773	3.059	0.0009441	ctrl - P10; ctrl - P8; P11 - P10; P2 - P10; P3 - P10; P2 - P11; P11 - P8; P2 - P3; P2 - P8; P3 - P8
TC0300012039.hg.1	TC0300012039.hg.1	jubobu.aAug10-unspliced	jubobu	NA	9.1238	0.0008891	3.05	0.00096	ctrl - P10; ctrl - P11; ctrl - P3; ctrl - P8; P2 - P10; P2 - P11; P2 - P3; P2 - P8
TC0200012341.hg.1	TC0200012341.hg.1	NA	NA	NA	9.1129	0.0008966	3.0477	0.0009665	P10 - ctrl; P2 - ctrl; P10 - P11; P10 - P3; P10 - P8; P2 - P11; P2 - P3; P2 - P8
TC1800008330.hg.1	TC1800008330.hg.1	NA	NA	NA	9.112	0.0008966	3.0475	0.0009665	ctrl - P10; ctrl - P11; ctrl - P3; ctrl - P8; P2 - P10; P3 - P10; P2 - P11; P2 - P8; P3 - P8
TC0500012058.hg.1	TC0500012058.hg.1	glawswawbu.aAug10-unspliced	glawswawbu	NA	9.1079	0.0008998	3.0466	0.0009666	ctrl - P10; ctrl - P11; ctrl - P8; P2 - P10; P2 - P11; P2 - P8; P3 - P8
TC1500008846.hg.1	TC1500008846.hg.1	pargler.aAug10-unspliced	pargler	NA	9.0718	0.0009114	3.0389	0.0009883	ctrl - P10; P2 - ctrl; ctrl - P8; P2 - P10; P2 - P11; P11 - P8; P2 - P3; P2 - P8; P3 - P8
TC1100010293.hg.1	TC1100010293.hg.1	uc057ztu.1	RNA5SP335	NA	9.0268	0.0009335	3.0292	0.0010055	P11 - ctrl; P2 - ctrl; P2 - P10; P2 - P11; P11 - P8; P2 - P3; P2 - P8
TC1800008220.hg.1	TC1800008220.hg.1	stuwu.aAug10-unspliced	stuwu	NA	9.0099	0.0009443	3.0255	0.0010113	ctrl - P10; ctrl - P11; ctrl - P3; ctrl - P8; P2 - P10; P2 - P11; P2 - P3; P2 - P8
TC1500007391.hg.1	TC1500007391.hg.1	rarzo.aAug10-unspliced	rarzo	NA	8.9331	0.00098	3.0089	0.0010052	ctrl - P10; ctrl - P11; ctrl - P3; ctrl - P8; P2 - P10; P3 - P10; P2 - P11; P2 - P8; P3 - P8
TC0800008457.hg.1	TC0800008457.hg.1	NA	NA	NA	8.8559	0.0010018	2.9921	0.0010093	P10 - ctrl; P11 - ctrl; P8 - ctrl; P10 - P2; P10 - P3; P11 - P2; P3 - P2; P8 - P2; P8 - P3
TC0600013737.hg.1	TC0600013737.hg.1	NA	NA	NA	8.8485	0.0010022	2.9905	0.0010097	ctrl - P10; ctrl - P8; P11 - P10; P2 - P10; P2 - P11; P11 - P8; P2 - P3; P2 - P8; P3 - P8
TC0300013115.hg.1	TC0300013115.hg.1	mawkybu.aAug10-unspliced	mawkybu	NA	8.8458	0.0010024	2.9899	0.0010098	ctrl - P10; ctrl - P11; ctrl - P8; P2 - P10; P3 - P10; P2 - P11; P3 - P11; P2 - P8; P3 - P8
TC0800011316.hg.1	TC0800011316.hg.1	dershoy.aAug10-unspliced	dershoy	NA	8.8419	0.0010026	2.989	0.0011	ctrl - P10; ctrl - P11; ctrl - P8; P2 - P10; P2 - P11; P2 - P3; P2 - P8; P3 - P8
TC0900010085.hg.1	TC0900010085.hg.1	glaskybu.aAug10-unspliced	glaskybu	NA	8.8207	0.0010037	2.9844	0.0011111	ctrl - P10; ctrl - P8; P2 - P10; P2 - P11; P11 - P8; P2 - P3; P2 - P8; P3 - P8
TC1600009136.hg.1	TC1600009136.hg.1	NA	NA	NA	8.8144	0.00104	2.983	0.0011114	P10 - ctrl; P11 - ctrl; P10 - P2; P10 - P3; P11 - P2; P11 - P3; P8 - P2; P8 - P3
TC0200009872.hg.1	TC0200009872.hg.1	zeekla.aAug10-unspliced	zeekla	NA	8.8096	0.0010042	2.982	0.0011116	ctrl - P10; ctrl - P8; P11 - P10; P2 - P10; P3 - P10; P11 - P8; P2 - P8; P3 - P8
TC0500006712.hg.1	TC0500006712.hg.1	NR_003689	SNORD123	5p15.2	8.773	0.0010062	2.9739	0.0011137	ctrl - P10; ctrl - P2; ctrl - P8; P11 - P10; P3 - P10; P11 - P2; P11 - P8; P3 - P8
TC1900007800.hg.1	TC1900007800.hg.1	NA	NA	NA	8.7405	0.00108	2.9668	0.0011155	ctrl - P10; ctrl - P11; ctrl - P3; ctrl - P8; P2 - P10; P2 - P3; P2 - P8
TC1600007187.hg.1	TC1600007187.hg.1	lysny.aAug10-unspliced	lysny	NA	8.7384	0.0010081	2.9663	0.0011156	ctrl - P10; ctrl - P3; ctrl - P8; P2 - P10; P2 - P11; P2 - P3; P2 - P8

TC1000010300.hg.1	TC1000010300.hg.1	vonoyby.aAug10-unspliced	vonoyby	NA	8.7322	0.001084	2.9649	0.001159	ctrl - P10; ctrl - P11; ctrl - P8; P2 - P10; P3 - P10; P2 - P11; P2 - P8; P3 - P8
TC0Y00007305.hg.1	TC0Y00007305.hg.1	merbo.aAug10-unspliced	merbo	NA	8.7136	0.001094	2.9608	0.001169	ctrl - P10; ctrl - P11; ctrl - P2; ctrl - P8; P11 - P10; P3 - P10; P3 - P2; P3 - P8
TC0200009780.hg.1	TC0200009780.hg.1	darfarbu.aAug10-unspliced	darfarbu	NA	8.6834	0.001111	2.9541	0.001187	ctrl - P10; P3 - ctrl; ctrl - P8; P3 - P10; P3 - P11; P3 - P2; P2 - P8; P3 - P8
TC1800007417.hg.1	TC1800007417.hg.1	maser.aAug10-unspliced	maser	NA	8.6427	0.001135	2.9451	0.001211	ctrl - P10; ctrl - P11; ctrl - P3; ctrl - P8; P2 - P10; P11 - P8; P2 - P3; P2 - P8
TC2100007073.hg.1	TC2100007073.hg.1	ENST00000602568.1	AP001429.1		8.6373	0.001138	2.9439	0.001214	ctrl - P10; ctrl - P11; ctrl - P8; P2 - P10; P3 - P10; P2 - P11; P2 - P3; P2 - P8; P3 - P8
TC0300011335.hg.1	TC0300011335.hg.1	klawnybo.aAug10-unspliced	klawnybo	NA	8.6204	0.001148	2.9401	0.001224	ctrl - P10; ctrl - P3; ctrl - P8; P11 - P10; P2 - P10; P11 - P8; P2 - P3; P2 - P8
TC1000008069.hg.1	TC1000008069.hg.1	geemarby.aAug10-unspliced	geemarby	NA	8.6097	0.001154	2.9377	0.001231	ctrl - P10; ctrl - P11; ctrl - P8; P2 - P10; P3 - P10; P2 - P11; P3 - P11; P2 - P8; P3 - P8
TC1900009862.hg.1	TC1900009862.hg.1	stoytobu.aAug10-unspliced	stoytobu	NA	8.6074	0.001156	2.9372	0.001231	ctrl - P10; ctrl - P11; ctrl - P3; ctrl - P8; P2 - P10; P2 - P11; P2 - P3; P2 - P8
TC0300008340.hg.1	TC0300008340.hg.1	NA	NA	NA	8.5974	0.001162	2.935	0.001237	ctrl - P10; ctrl - P11; ctrl - P8; P2 - P10; P2 - P11; P3 - P11; P2 - P3; P2 - P8; P3 - P8
TC0200012315.hg.1	TC0200012315.hg.1	slerparbo.aAug10-unspliced	slerparbo	NA	8.5816	0.001171	2.9314	0.001247	ctrl - P10; ctrl - P3; ctrl - P8; P2 - P10; P2 - P11; P2 - P3; P2 - P8
TC2200006889.hg.1	TC2200006889.hg.1	boror.aAug10-unspliced	boror	NA	8.4774	0.001236	2.908	0.001315	ctrl - P10; ctrl - P8; P2 - P10; P2 - P11; P2 - P3; P2 - P8
TC2200008179.hg.1	TC2200008179.hg.1	C22orf43.vkAug10-unspliced	C22orf43	NA	8.4581	0.001248	2.9037	0.001328	ctrl - P10; ctrl - P11; ctrl - P3; ctrl - P8; P2 - P10; P3 - P10; P2 - P8; P3 - P8
TC0300012716.hg.1	TC0300012716.hg.1	ENST00000567714.1	RP11-274H2.5		8.447	0.001256	2.9012	0.001335	ctrl - P10; ctrl - P3; ctrl - P8; P11 - P10; P2 - P10; P11 - P3; P11 - P8; P2 - P3; P2 - P8
TC1600010550.hg.1	TC1600010550.hg.1	swerdu.aAug10-unspliced	swerdu	NA	8.4232	0.001271	2.8958	0.001351	ctrl - P10; ctrl - P3; ctrl - P8; P11 - P10; P2 - P10; P11 - P3; P11 - P8; P2 - P8
TC1200011939.hg.1	TC1200011939.hg.1	shofu.aAug10-unspliced	shofu	NA	8.4217	0.001272	2.8954	0.001352	P2 - ctrl; ctrl - P8; P2 - P10; P3 - P10; P2 - P11; P3 - P11; P2 - P8; P3 - P8
HTA2-pos-2999493_st	23150279	NA	NA	NA	8.4008	0.001286	2.8907	0.001366	ctrl - P11; ctrl - P3; ctrl - P8; P2 - P10; P10 - P3; P2 - P11; P2 - P3; P2 - P8; P8 - P3
TC0900010392.hg.1	TC0900010392.hg.1	NA	NA	NA	8.3134	0.001347	2.8708	0.00143	ctrl - P10; ctrl - P11; ctrl - P3; ctrl - P8; P2 - P10; P2 - P11; P2 - P3; P2 - P8
TC1200012052.hg.1	TC1200012052.hg.1	neygoyby.aAug10-unspliced	neygoyby	NA	8.3013	0.001355	2.868	0.001438	ctrl - P10; ctrl - P8; P11 - P10; P2 - P10; P3 - P10; P2 - P11; P2 - P3; P2 - P8
TC0200011383.hg.1	TC0200011383.hg.1	ruyura.aAug10-unspliced	ruyura	NA	8.2955	0.001359	2.8667	0.001442	ctrl - P10; ctrl - P3; ctrl - P8; P11 - P10; P2 - P10; P3 - P10; P11 - P8; P2 - P8; P3 - P8
TC1500009808.hg.1	TC1500009808.hg.1	larsor.aAug10-unspliced	larsor	NA	8.2882	0.001365	2.865	0.001447	ctrl - P10; ctrl - P11; ctrl - P3; ctrl - P8; P2 - P10; P2 - P11; P2 - P3; P2 - P8
TC0200014097.hg.1	TC0200014097.hg.1	sleyklo.aAug10-unspliced	sleyklo	NA	8.2833	0.001368	2.8639	0.00145	ctrl - P10; ctrl - P11; ctrl - P3; ctrl - P8; P2 - P10; P2 - P11; P2 - P3; P2 - P8
TC0400007494.hg.1	TC0400007494.hg.1	NA	NA	NA	8.2661	0.001381	2.8599	0.001463	P2 - ctrl; P3 - ctrl; P2 - P10; P3 - P10; P2 - P11; P3 - P11; P2 - P8; P3 - P8
TC1600008429.hg.1	TC1600008429.hg.1	swydo.aAug10-unspliced	swydo	NA	8.2158	0.001418	2.8483	0.001501	ctrl - P11; ctrl - P8; P2 - P10; P2 - P11; P2 - P3; P2 - P8
TC0100009197.hg.1	TC0100009197.hg.1	sayaro.aAug10-unspliced	sayaro	NA	8.1991	0.001431	2.8445	0.001514	ctrl - P11; P2 - ctrl; ctrl - P8; P2 - P10; P2 - P11; P3 - P11; P2 - P3; P2 - P8; P3 - P8
TC1100007754.hg.1	TC1100007754.hg.1	swyfer.aAug10-unspliced	swyfer	NA	8.1926	0.001436	2.843	0.001519	ctrl - P10; ctrl - P11; ctrl - P3; ctrl - P8; P2 - P10; P2 - P11; P2 - P3; P2 - P8
TC0300009674.hg.1	TC0300009674.hg.1	weymubu.aAug10-unspliced	weymubu	NA	8.1275	0.001486	2.8279	0.001572	ctrl - P3; ctrl - P8; P2 - P10; P11 - P3; P11 - P8; P2 - P3; P2 - P8
TC0400007296.hg.1	TC0400007296.hg.1	muglarby.aAug10-unspliced	muglarby	NA	8.0898	0.001517	2.8191	0.001603	ctrl - P10; ctrl - P11; ctrl - P8; P2 - P10; P2 - P11; P2 - P3; P2 - P8
TC0500010459.hg.1	TC0500010459.hg.1	NA	NA	NA	8.0772	0.001527	2.8162	0.001614	ctrl - P10; ctrl - P8; P11 - P10; P2 - P10; P11 - P8; P2 - P3; P2 - P8; P3 - P8
TC1800008406.hg.1	TC1800008406.hg.1	OTTHUMT00000473296	RP11-210K20.5		8.0527	0.001547	2.8104	0.001634	ctrl - P11; ctrl - P3; ctrl - P8; P2 - P10; P10 - P8; P2 - P11; P2 - P3; P2 - P8
TC0300006841.hg.1	TC0300006841.hg.1	garfabo.aAug10-unspliced	garfabo	NA	8.0521	0.001548	2.8103	0.001634	ctrl - P10; ctrl - P11; ctrl - P8; P2 - P10; P3 - P10; P2 - P11; P2 - P3; P2 - P8; P3 - P8
TC2100007567.hg.1	TC2100007567.hg.1	NA	NA	NA	8.0179	0.001577	2.8023	0.001664	P10 - ctrl; P2 - ctrl; P3 - ctrl; P2 - P10; P2 - P11; P2 - P3; P2 - P8; P3 - P8
TC1400007225.hg.1	TC1400007225.hg.1	sworcherbu.aAug10-unspliced	sworcherbu	NA	8.0139	0.00158	2.8013	0.001667	ctrl - P10; ctrl - P11; ctrl - P3; ctrl - P8; P2 - P10; P10 - P8; P2 - P11; P2 - P3; P2 - P8

TC1000008929.hg.1	TC1000008929.hg.1	lermu.aAug10-unspliced	lermu	NA	7.9692	0.001619	2.7908	0.001707	P2 - ctrl; ctrl - P8; P2 - P10; P3 - P10; P2 - P11; P3 - P11; P2 - P8; P3 - P8
TC0500012566.hg.1	TC0500012566.hg.1	vorsneebu.aAug10-unspliced	vorsneebu	NA	7.8999	0.001681	2.7744	0.001772	P10 - ctrl; P11 - ctrl; P3 - ctrl; P8 - ctrl; P10 - P2; P11 - P2; P8 - P2
TC0600011169.hg.1	TC0600011169.hg.1	NA	NA	NA	7.8785	0.001701	2.7694	0.001792	ctrl - P10; ctrl - P8; P2 - P10; P2 - P11; P11 - P8; P2 - P3; P2 - P8; P3 - P8
TC0800011603.hg.1	TC0800011603.hg.1	smobly.aAug10-unspliced	smobly	NA	7.874	0.001705	2.7683	0.001796	ctrl - P8; P2 - P10; P2 - P11; P11 - P8; P2 - P8; P3 - P8
TC1700006768.hg.1	TC1700006768.hg.1	NM_001406	EFNB3	1949	7.8458	0.001732	2.7616	0.001823	ctrl - P10; ctrl - P3; ctrl - P8; P11 - P10; P2 - P10; P11 - P3; P11 - P8; P2 - P3
TC1000008905.hg.1	TC1000008905.hg.1	spypawby.aAug10-unspliced	spypawby	NA	7.7933	0.001782	2.749	0.001875	ctrl - P10; ctrl - P11; ctrl - P3; ctrl - P8; P2 - P10; P2 - P11; P2 - P3; P2 - P8
TC1700008114.hg.1	TC1700008114.hg.1	chawwabu.aAug10-unspliced	chawwabu	NA	7.7377	0.001838	2.7357	0.001933	ctrl - P10; ctrl - P3; ctrl - P8; P2 - P10; P2 - P11; P11 - P8; P2 - P3; P2 - P8
TC0800009303.hg.1	TC0800009303.hg.1	NA	NA	NA	7.6817	0.001896	2.7222	0.001993	ctrl - P10; ctrl - P11; ctrl - P8; P2 - P10; P3 - P10; P2 - P11; P3 - P11; P2 - P8; P3 - P8
TC0200008885.hg.1	TC0200008885.hg.1	steymoybo.aAug10-unspliced	steymoybo	NA	7.6735	0.001905	2.7202	0.002002	ctrl - P10; ctrl - P2; ctrl - P3; ctrl - P8; P11 - P3; P11 - P8
TC0700009180.hg.1	TC0700009180.hg.1	plawbleeby.aAug10-unspliced	plawbleeby	NA	7.6206	0.001962	2.7073	0.002061	ctrl - P11; P2 - ctrl; ctrl - P8; P2 - P10; P2 - P11; P2 - P3; P2 - P8
TC0100015793.hg.1	TC0100015793.hg.1	POGZ.vjAug10-unspliced	POGZ	NA	7.6158	0.001967	2.7062	0.002066	ctrl - P10; ctrl - P3; ctrl - P8; P2 - P10; P2 - P11; P2 - P3; P2 - P8
TC0200010425.hg.1	TC0200010425.hg.1	NA	NA	NA	7.597	0.001988	2.7016	0.002087	ctrl - P10; ctrl - P8; P11 - P10; P2 - P10; P3 - P10; P2 - P11; P2 - P8; P3 - P8
TC0X00009556.hg.1	TC0X00009556.hg.1	nojy.aAug10-unspliced	nojy	NA	7.5694	0.002019	2.6948	0.002118	P10 - ctrl; P11 - ctrl; P8 - ctrl; P10 - P2; P10 - P3; P11 - P2; P11 - P3; P8 - P2; P8 - P3
TC0700007346.hg.1	TC0700007346.hg.1	skeychoyby.aAug10-unspliced	skeychoyby	NA	7.562	0.002028	2.693	0.002126	ctrl - P10; ctrl - P11; ctrl - P8; P3 - P10; P2 - P11; P3 - P11; P2 - P8; P3 - P8
TC0Y00007294.hg.1	TC0Y00007294.hg.1	tobo.aAug10-unspliced	tobo	NA	7.5571	0.002033	2.6918	0.002131	ctrl - P10; ctrl - P11; ctrl - P2; ctrl - P8; P11 - P10; P3 - P10; P3 - P2; P3 - P8
TC1200007263.hg.1	TC1200007263.hg.1	OTTHUMT00000474581	RP11-843B15.4		7.548	0.002044	2.6896	0.002141	ctrl - P10; ctrl - P11; P2 - P10; P3 - P10; P2 - P11; P3 - P11; P2 - P8; P3 - P8
TC1200008708.hg.1	TC1200008708.hg.1	flublorbu.aAug10-unspliced	flublorbu	NA	7.5152	0.002082	2.6816	0.00218	ctrl - P10; P2 - ctrl; ctrl - P8; P2 - P10; P3 - P10; P2 - P11; P2 - P3; P2 - P8; P3 - P8
TC1300008706.hg.1	TC1300008706.hg.1	ELF1.vdAug10-unspliced	ELF1	NA	7.4767	0.002128	2.6721	0.002228	ctrl - P10; ctrl - P3; ctrl - P8; P2 - P10; P2 - P11; P11 - P8; P2 - P3; P2 - P8
TC0500009611.hg.1	TC0500009611.hg.1	maplaby.aAug10-unspliced	maplaby	NA	7.4711	0.002135	2.6707	0.002234	ctrl - P10; ctrl - P11; ctrl - P3; ctrl - P8; P2 - P10; P2 - P11; P2 - P3; P2 - P8
TC0100018060.hg.1	TC0100018060.hg.1	fuvoybo.aAug10-unspliced	fuvoybo	NA	7.4667	0.00214	2.6696	0.002238	ctrl - P10; ctrl - P11; ctrl - P8; P2 - P10; P2 - P11; P2 - P8; P3 - P8
TC1600007501.hg.1	TC1600007501.hg.1	NA	NA	NA	7.4602	0.002148	2.668	0.002246	P2 - ctrl; P2 - P10; P2 - P11; P2 - P3; P2 - P8
TSUnmapped00000920.hg.1	TSUnmapped00000920.hg.1	NA	NA	NA	7.4377	0.002175	2.6625	0.002274	P11 - ctrl; P2 - ctrl; P11 - P10; P2 - P10; P11 - P8; P2 - P3; P2 - P8
TC0X00009774.hg.1	TC0X00009774.hg.1	merber.aAug10-unspliced	merber	NA	7.4138	0.002205	2.6565	0.002304	ctrl - P10; ctrl - P11; ctrl - P3; ctrl - P8; P2 - P10; P2 - P11; P2 - P3; P2 - P8
TC0200010909.hg.1	TC0200010909.hg.1	NA	NA	NA	7.4037	0.002218	2.654	0.002316	ctrl - P10; ctrl - P11; ctrl - P8; P2 - P10; P3 - P10; P11 - P8; P2 - P8; P3 - P8
TC0300006704.hg.1	TC0300006704.hg.1	kodeybo.aAug10-unspliced	kodeybo	NA	7.386	0.002241	2.6496	0.002339	ctrl - P10; ctrl - P8; P2 - P10; P11 - P8; P2 - P3; P2 - P8; P3 - P8
TC0700008327.hg.1	TC0700008327.hg.1	NA	NA	NA	7.3638	0.002269	2.6441	0.002368	ctrl - P10; ctrl - P3; ctrl - P8; P11 - P10; P2 - P10; P11 - P8; P2 - P3; P2 - P8
TC0200015170.hg.1	TC0200015170.hg.1	NA	NA	NA	7.3305	0.002313	2.6358	0.002413	ctrl - P10; ctrl - P8; P11 - P10; P2 - P10; P11 - P3; P11 - P8; P2 - P3; P2 - P8
TC0200008048.hg.1	TC0200008048.hg.1	rarkawbu.aAug10-unspliced	rarkawbu	NA	7.3052	0.002347	2.6295	0.002447	ctrl - P10; ctrl - P3; ctrl - P8; P11 - P10; P2 - P10; P11 - P3; P11 - P8; P2 - P8
TC1000009986.hg.1	TC1000009986.hg.1	NA	NA	NA	7.2609	0.002408	2.6184	0.002509	ctrl - P10; ctrl - P3; ctrl - P8; P11 - P10; P2 - P10; P11 - P8; P2 - P8
TC0400012258.hg.1	TC0400012258.hg.1	fluplawby.aAug10-unspliced	fluplawby	NA	7.2535	0.002418	2.6165	0.002519	P11 - ctrl; P11 - P10; P10 - P2; P10 - P3; P11 - P2; P11 - P3; P11 - P8
TC1100006886.hg.1	TC1100006886.hg.1	seysloyby.aAug10-unspliced	seysloyby	NA	7.25	0.002423	2.6156	0.002523	ctrl - P10; ctrl - P11; ctrl - P3; ctrl - P8; P2 - P10; P2 - P11; P2 - P3; P2 - P8
TC0700007027.hg.1	TC0700007027.hg.1	flerslawbu.aAug10-unspliced	flerslawbu	NA	7.219	0.002467	2.6078	0.002568	ctrl - P10; P2 - ctrl; ctrl - P8; P2 - P10; P2 - P11; P11 - P8; P2 - P3; P2 - P8; P3 - P8

TC0X00010424.hg.1	TC0X00010424.hg.1	ENST00000424887.1	TCEAL3-AS1		7.2144	0.002474	2.6066	0.002574	ctrl - P10; ctrl - P11; ctrl - P3; ctrl - P8; P2 - P10; P2 - P11; P2 - P3; P2 - P8
TC0Y00006885.hg.1	TC0Y00006885.hg.1	bybar.aAug10-unspliced	bybar	NA	7.1411	0.002582	2.588	0.002685	ctrl - P10; ctrl - P3; ctrl - P8; P2 - P10; P11 - P8; P2 - P3; P2 - P8
TC0800007703.hg.1	TC0800007703.hg.1	NA	NA	NA	7.1306	0.002598	2.5854	0.002701	P10 - ctrl; P11 - ctrl; P8 - ctrl; P10 - P2; P10 - P3; P11 - P2; P11 - P3; P8 - P2; P8 - P3
TC1700010263.hg.1	TC1700010263.hg.1	vawwybu.aAug10-unspliced	vawwybu	NA	7.0927	0.002656	2.5757	0.00276	ctrl - P10; ctrl - P11; ctrl - P8; P2 - P10; P2 - P11; P2 - P8; P3 - P8
TC0100010458.hg.1	TC0100010458.hg.1	zarorbo.aAug10-unspliced	zarorbo	NA	7.0648	0.002701	2.5686	0.002805	ctrl - P10; ctrl - P11; ctrl - P3; ctrl - P8; P2 - P10; P11 - P8; P2 - P8
TC0900010766.hg.1	TC0900010766.hg.1	shoyley.aAug10-unspliced	shoyley		7.0632	0.002703	2.5682	0.002806	ctrl - P10; ctrl - P3; ctrl - P8; P11 - P10; P2 - P10; P11 - P8; P2 - P3; P2 - P8
TC2000009054.hg.1	TC2000009054.hg.1	fleyroybu.aAug10-unspliced	fleyroybu	NA	6.9675	0.002861	2.5435	0.002969	ctrl - P10; ctrl - P8; P11 - P10; P2 - P10; P11 - P8; P2 - P3; P2 - P8; P3 - P8
HTA2-pos-2985881_st	23150245	NA	NA	NA	6.8664	0.003039	2.5172	0.003153	ctrl - P10; ctrl - P8; P11 - P10; P2 - P10; P3 - P10; P11 - P8; P2 - P8
TC0700012273.hg.1	TC0700012273.hg.1	cherkaw.aAug10-unspliced	cherkaw	NA	6.8626	0.003046	2.5162	0.003159	P2 - ctrl; ctrl - P8; P2 - P10; P3 - P10; P2 - P11; P2 - P8; P3 - P8
TC0300007877.hg.1	TC0300007877.hg.1	rawsnoyby.aAug10-unspliced	rawsnoyby	NA	6.8393	0.003089	2.5101	0.003202	ctrl - P10; ctrl - P3; ctrl - P8; P2 - P10; P2 - P11; P2 - P3; P2 - P8
TC0700010358.hg.1	TC0700010358.hg.1	NM_176813	AGR3	155465	6.7474	0.003266	2.486	0.003384	P10 - ctrl; P8 - ctrl; P10 - P2; P11 - P2; P8 - P11; P3 - P2; P8 - P2
TC0X00007082.hg.1	TC0X00007082.hg.1	NA	NA	NA	6.7334	0.003294	2.4823	0.003412	ctrl - P10; ctrl - P11; ctrl - P3; ctrl - P8; P2 - P10; P2 - P11; P2 - P3; P2 - P8
TC1600007273.hg.1	TC1600007273.hg.1	NA	NA	NA	6.6441	0.003479	2.4585	0.003602	ctrl - P10; ctrl - P3; ctrl - P8; P2 - P10; P2 - P11; P2 - P3; P2 - P8
TC1200010028.hg.1	TC1200010028.hg.1	NA	NA	NA	6.5747	0.003631	2.4399	0.003758	ctrl - P10; ctrl - P3; ctrl - P8; P11 - P10; P2 - P10; P11 - P3; P11 - P8; P2 - P3; P2 - P8
TC0900009426.hg.1	TC0900009426.hg.1	kyplybu.aAug10-unspliced	kyplybu	NA	6.5017	0.0038	2.4202	0.003931	ctrl - P10; ctrl - P11; ctrl - P8; P2 - P10; P2 - P11; P2 - P3; P2 - P8
TC0X00006592.hg.1	TC0X00006592.hg.1	pyva.aAug10-unspliced	pyva	NA	6.499	0.003806	2.4195	0.003936	ctrl - P10; ctrl - P8; P2 - P10; P3 - P10; P2 - P8; P3 - P8
TC0500007948.hg.1	TC0500007948.hg.1	muhome.aAug10-unspliced	muhome	NA	6.4898	0.003828	2.417	0.003956	ctrl - P8; P2 - P10; P2 - P11; P2 - P8; P3 - P8
TC0400012069.hg.1	TC0400012069.hg.1	snawsnorby.aAug10-unspliced	snawsnorby	NA	6.4605	0.003899	2.4091	0.004028	ctrl - P10; ctrl - P8; P2 - P10; P2 - P11; P11 - P8; P2 - P3; P2 - P8
TC1700006519.hg.1	TC1700006519.hg.1	shaler.aAug10-unspliced	shaler	NA	6.3964	0.004059	2.3916	0.004192	P10 - ctrl; P11 - ctrl; P8 - ctrl; P10 - P2; P11 - P2; P11 - P3; P8 - P2
TC1800008630.hg.1	TC1800008630.hg.1	LOC100127909.aAug10-unspliced	LOC100127909	NA	6.3614	0.00415	2.382	0.004284	ctrl - P10; ctrl - P3; ctrl - P8; P11 - P10; P2 - P10; P11 - P3; P11 - P8; P2 - P3; P2 - P8
TC0200007836.hg.1	TC0200007836.hg.1	NA	NA	NA	6.3356	0.004218	2.3749	0.004352	ctrl - P10; ctrl - P11; ctrl - P8; P2 - P10; P3 - P10; P2 - P11; P3 - P11; P2 - P8; P3 - P8
TC0800008919.hg.1	TC0800008919.hg.1	kinara.aAug10-unspliced	kinara	NA	6.2819	0.004365	2.36	0.004502	ctrl - P10; ctrl - P11; ctrl - P8; P2 - P10; P3 - P10; P2 - P11; P2 - P8
TC0700012151.hg.1	TC0700012151.hg.1	nerju.aAug10-unspliced	nerju	NA	6.271	0.004395	2.357	0.004531	ctrl - P10; ctrl - P11; ctrl - P8; P2 - P10; P2 - P11; P3 - P11; P2 - P8; P3 - P8
TC0500007704.hg.1	TC0500007704.hg.1	chosmarbu.aAug10-unspliced	chosmarbu	NA	6.266	0.004409	2.3556	0.004542	ctrl - P10; ctrl - P3; ctrl - P8; P11 - P10; P2 - P10; P11 - P3; P11 - P8; P2 - P3; P2 - P8
TC0500007712.hg.1	TC0500007712.hg.1	blorsmarbu.aAug10-unspliced	blorsmarbu	NA	6.266	0.004409	2.3556	0.004542	ctrl - P10; ctrl - P3; ctrl - P8; P11 - P10; P2 - P10; P11 - P3; P11 - P8; P2 - P3; P2 - P8
TC0100006869.hg.1	TC0100006869.hg.1	kawnawbu.aAug10-unspliced	kawnawbu	NA	6.2365	0.004493	2.3474	0.004627	ctrl - P10; ctrl - P11; ctrl - P3; ctrl - P8; P2 - P10; P2 - P11; P2 - P8
TC0200008871.hg.1	TC0200008871.hg.1	fleeswarby.aAug10-unspliced	fleeswarby	NA	6.2344	0.004499	2.3468	0.004631	ctrl - P10; ctrl - P3; ctrl - P8; P11 - P10; P2 - P10; P11 - P3; P11 - P8; P2 - P3; P2 - P8
TC0200007763.hg.1	TC0200007763.hg.1	studaw.aAug10-unspliced	studaw	NA	6.2056	0.004583	2.3388	0.004715	P2 - ctrl; ctrl - P8; P2 - P10; P3 - P10; P2 - P11; P11 - P8; P2 - P8; P3 - P8
TC0200007414.hg.1	TC0200007414.hg.1	teraro.aAug10-unspliced	teraro	NA	6.1802	0.004659	2.3317	0.004791	ctrl - P10; ctrl - P8; P2 - P10; P2 - P11; P2 - P8; P3 - P8
TC1700011455.hg.1	TC1700011455.hg.1	tuwaw.aAug10-unspliced	tuwaw	NA	6.154	0.004738	2.3244	0.004871	ctrl - P11; P2 - P10; P2 - P11; P3 - P11; P2 - P3; P2 - P8
TC0700007286.hg.1	TC0700007286.hg.1	soysmubu.aAug10-unspliced	soysmubu	NA	6.1399	0.004782	2.3204	0.004913	ctrl - P10; ctrl - P8; P2 - P10; P2 - P11; P2 - P3; P2 - P8
TC0700010552.hg.1	TC0700010552.hg.1	snarcheeby.aAug10-unspliced	snarcheeby	NA	6.1027	0.004899	2.3099	0.005031	ctrl - P10; ctrl - P8; P11 - P10; P2 - P10; P11 - P8; P2 - P8; P3 - P8

TC1600006981.hg.1	TC1600006981.hg.1	ferraw.aAug10-unspliced	ferraw	NA	6.0105	0.005202	2.2838	0.005341	ctrl - P10; ctrl - P11; ctrl - P8; P2 - P10; P2 - P11; P2 - P8
TC0300011206.hg.1	TC0300011206.hg.1	deemoybo.aAug10-unspliced	deemoybo	NA	5.9216	0.005516	2.2584	0.005661	ctrl - P10; ctrl - P8; P2 - P10; P2 - P11; P11 - P8; P2 - P8; P3 - P8
TC0500006615.hg.1	TC0500006615.hg.1	charspoybu.aAug10-unspliced	charspoybu	NA	5.8405	0.005822	2.235	0.005972	ctrl - P10; ctrl - P8; P2 - P10; P2 - P11; P2 - P3; P2 - P8
TC0300009501.hg.1	TC0300009501.hg.1	jorkarbu.aAug10-unspliced	jorkarbu	NA	5.7835	0.006048	2.2184	0.006202	ctrl - P11; ctrl - P8; P2 - P10; P2 - P11; P3 - P11; P2 - P8; P3 - P8
TC0600013128.hg.1	TC0600013128.hg.1	klarspawbu.aAug10-unspliced	klarspawbu	NA	5.7236	0.006298	2.2008	0.006455	ctrl - P8; P2 - P10; P3 - P10; P2 - P11; P3 - P11; P2 - P8; P3 - P8
TC1200008822.hg.1	TC1200008822.hg.1	NA	NA	NA	5.6132	0.006789	2.1682	0.006956	ctrl - P10; ctrl - P3; ctrl - P8; P2 - P10; P2 - P3; P2 - P8
TC0700012147.hg.1	TC0700012147.hg.1	NA	NA	NA	5.5996	0.006852	2.1642	0.007018	ctrl - P10; ctrl - P11; ctrl - P3; ctrl - P8; P2 - P10; P2 - P8
TC1700009418.hg.1	TC1700009418.hg.1	NA	NA	NA	5.5812	0.006939	2.1587	0.007104	ctrl - P10; ctrl - P11; ctrl - P8; P2 - P10; P2 - P11; P3 - P11; P2 - P8
TC0200014720.hg.1	TC0200014720.hg.1	joyfarbu.aAug10-unspliced	joyfarbu	NA	5.5048	0.007315	2.1358	0.007485	ctrl - P10; ctrl - P11; ctrl - P8; P2 - P10; P2 - P11; P2 - P3; P2 - P8
TC0100014897.hg.1	TC0100014897.hg.1	blubar.aAug10-unspliced	blubar	NA	5.4886	0.007398	2.1309	0.007567	P2 - ctrl; ctrl - P8; P2 - P10; P2 - P11; P2 - P3; P2 - P8; P3 - P8
TC0400007451.hg.1	TC0400007451.hg.1	chergy.aAug10-unspliced	chergy	NA	5.4612	0.00754	2.1227	0.007709	ctrl - P8; P2 - P10; P2 - P11; P2 - P3; P2 - P8
TC0900010926.hg.1	TC0900010926.hg.1	NA	NA	NA	5.4377	0.007664	2.1155	0.007833	ctrl - P10; ctrl - P8; P2 - P10; P2 - P11; P2 - P3; P2 - P8
TC0100008991.hg.1	TC0100008991.hg.1	pawkloy.aAug10-unspliced	pawkloy	NA	5.4336	0.007686	2.1143	0.007853	ctrl - P10; ctrl - P8; P11 - P10; P2 - P10; P3 - P10; P11 - P8; P2 - P8; P3 - P8
TC0600009713.hg.1	TC0600009713.hg.1	styjer.aAug10-unspliced	styjer	NA	5.3616	0.008084	2.0924	0.008256	ctrl - P11; ctrl - P8; P2 - P10; P2 - P11; P2 - P3; P2 - P8
TC1500009360.hg.1	TC1500009360.hg.1	speyzu.aAug10-unspliced	speyzu	NA	5.3439	0.008185	2.087	0.008356	ctrl - P8; P2 - P10; P2 - P11; P2 - P8; P3 - P8
TC0600012088.hg.1	TC0600012088.hg.1	ENST00000429053.1	RP13-476E20.1		5.2896	0.008506	2.0703	0.00868	ctrl - P10; ctrl - P2; ctrl - P3; ctrl - P8; P11 - P10
TC0200009433.hg.1	TC0200009433.hg.1	gorgabo.aAug10-unspliced	gorgabo	NA	5.2064	0.009026	2.0445	0.009206	ctrl - P10; ctrl - P8; P11 - P10; P2 - P10; P11 - P8; P2 - P8; P3 - P8
TC0300009210.hg.1	TC0300009210.hg.1	zeeforbo.aAug10-unspliced	zeeforbo	NA	5.1332	0.009513	2.0217	0.009699	ctrl - P10; ctrl - P11; ctrl - P8; P2 - P10; P2 - P11; P2 - P3; P2 - P8
TC1100010716.hg.1	TC1100010716.hg.1	charloby.aAug10-unspliced	charloby	NA	5.1075	0.009692	2.0136	0.009877	ctrl - P10; ctrl - P11; ctrl - P3; ctrl - P8; P2 - P10; P2 - P11; P2 - P8
HTA2-pos-2985916_st	23150251	NA	NA	NA	5.0855	0.009847	2.0067	0.010032	ctrl - P10; ctrl - P8; P2 - P10; P2 - P11; P2 - P8
HTA2-neg-47419654_st	23149597	NA	NA	NA	4.9047	0.011244	1.9491	0.011451	ctrl - P10; ctrl - P8; P2 - P10; P2 - P11; P2 - P8; P3 - P8
TC2100007807.hg.1	TC2100007807.hg.1	NA	NA	NA	4.8144	0.012027	1.9199	0.012242	ctrl - P10; ctrl - P11; ctrl - P8; P2 - P10; P2 - P11; P2 - P8
TC1200009922.hg.1	TC1200009922.hg.1	gloychu.aAug10-unspliced	gloychu	NA	4.8039	0.012122	1.9164	0.012334	ctrl - P10; ctrl - P8; P2 - P10; P2 - P11; P2 - P3; P2 - P8
TC2200008360.hg.1	TC2200008360.hg.1	bleyter.aAug10-unspliced	bleyter	NA	4.8027	0.012132	1.9161	0.01234	ctrl - P10; ctrl - P8; P2 - P10; P3 - P10; P2 - P11; P2 - P8; P3 - P8
TC1000007352.hg.1	TC1000007352.hg.1	NA	NA	NA	4.6554	0.013563	1.8676	0.01379	P2 - ctrl; P2 - P10; P2 - P11; P3 - P11; P2 - P8; P3 - P8
TC1100011571.hg.1	TC1100011571.hg.1	jymoy.aAug10-unspliced	jymoy	NA	4.5914	0.014245	1.8463	0.014477	ctrl - P10; ctrl - P11; ctrl - P8; P2 - P8; P3 - P8
HTA2-pos-3475282_st	23150564	NA	NA	NA	4.4902	0.015406	1.8123	0.01565	ctrl - P3; ctrl - P8; P2 - P3; P2 - P8
TC0X00008242.hg.1	TC0X00008242.hg.1	lorfey.aAug10-unspliced	lorfey	NA	4.4585	0.015792	1.8016	0.016036	ctrl - P10; ctrl - P11; ctrl - P8; P2 - P10; P2 - P8; P3 - P8
TC0100014611.hg.1	TC0100014611.hg.1	ENST00000608748.1	RP11-17E13.2		4.4098	0.016407	1.785	0.016654	ctrl - P10; ctrl - P8; P2 - P10; P3 - P10; P2 - P8
TC0600009592.hg.1	TC0600009592.hg.1	voyjer.aAug10-unspliced	voyjer	NA	4.3275	0.01751	1.7567	0.017765	P10 - ctrl; P2 - ctrl; P10 - P3; P2 - P11; P2 - P3
TC1600006898.hg.1	TC1600006898.hg.1	OTTHUMT00000473762	RP11-66H6.4		4.2093	0.019246	1.7157	0.01952	ctrl - P11; ctrl - P8; P2 - P8; P3 - P8
TC1800008211.hg.1	TC1800008211.hg.1	huhuma.aAug10-unspliced	huhuma	NA	4.1442	0.020286	1.6928	0.020566	P10 - P11; P2 - P11; P3 - P11; P3 - P8

TC1600007835.hg.1	TC1600007835.hg.1	NR_039672	MIR548AI	1.01E+08	4.125	0.020606	1.686	0.020882	ctrl - P3; ctrl - P8; P2 - P3; P2 - P8
TC0100018008.hg.1	TC0100018008.hg.1	NA	NA	NA	4.0737	0.021488	1.6678	0.021767	ctrl - P8; P2 - P10; P2 - P11; P2 - P8; P3 - P8
HTA2-neg-47419722_st	23149624	NA	NA	NA	4.0096	0.022652	1.6449	0.022937	ctrl - P10; ctrl - P11; ctrl - P8; P2 - P10; P2 - P11; P2 - P8
TC1100010690.hg.1	TC1100010690.hg.1	klomaw.aAug10-unspliced	klomaw	NA	3.9325	0.024151	1.6171	0.024444	ctrl - P10; ctrl - P11; ctrl - P8; P2 - P10; P2 - P11; P2 - P8
HTA2-pos-3581347_st	23150642	NA	NA	NA	3.8955	0.024908	1.6037	0.0252	P11 - P10; P11 - P2; P11 - P3; P8 - P2
TC1900008760.hg.1	TC1900008760.hg.1	NA	NA	NA	3.7686	0.027728	1.5571	0.028042	ctrl - P10; P11 - P10; P11 - P3; P11 - P8
TC2100006860.hg.1	TC2100006860.hg.1	bosy.aAug10-unspliced	bosy	NA	3.6663	0.03027	1.519	0.030601	ctrl - P10; ctrl - P11; ctrl - P3; ctrl - P8; P2 - P8
TC0100014777.hg.1	TC0100014777.hg.1	smornerbu.aAug10-unspliced	smornerbu	NA	3.5969	0.032148	1.4929	0.032486	ctrl - P10; ctrl - P11; ctrl - P8; P2 - P10; P2 - P11; P2 - P8
TC0500008234.hg.1	TC0500008234.hg.1	spoka.aAug10-unspliced	spoka	NA	3.5597	0.03321	1.4787	0.033545	ctrl - P10; ctrl - P11; ctrl - P8; P2 - P10; P2 - P11; P2 - P8
HTA2-neg-47424282_st	23150092	NA	NA	NA	3.4681	0.035997	1.4437	0.036347	ctrl - P10; P2 - P10; P3 - P10
TC0300013091.hg.1	TC0300013091.hg.1	NA	NA	NA	3.3982	0.038307	1.4167	0.038663	ctrl - P8; P11 - P8; P2 - P8
HTA2-pos-3371087_st	23150499	NA	NA	NA	3.2686	0.043056	1.366	0.043438	ctrl - P2; P10 - P2; P11 - P2; P3 - P2; P8 - P2
TC0600011223.hg.1	TC0600011223.hg.1	NA	NA	NA	3.1351	0.048662	1.3128	0.049075	P10 - ctrl; P3 - ctrl; P8 - ctrl

Supplementary Table 2A: Upregulated Gene Set Enrichment Analysis for 2478 probes identified by ANOVA with nominal p-value < 0.05.

GS	Size	ES	NES	NOM p-val	FDR q-val	FWER p-val	Rank at Max	Leading Edge
HES2_TARGET_GENES	81	0.96	1.12	0.001	1	1	62	tags=1%, list=3%, signal=1%
GOZGIT_ESR1_TARGETS_DN	103	0.95	1.11	0.002	1	1	45	tags=1%, list=2%, signal=1%
CREIGHTON_ENDOCRINE_THERAPY_RESISTANCE_1	51	0.96	1.12	0.003	1	1	62	tags=4%, list=3%, signal=4%
ISL1_TARGET_GENES	41	0.97	1.13	0.005	1	1	45	tags=2%, list=2%, signal=2%
MIR335_3P	50	0.96	1.12	0.007	1	1	62	tags=2%, list=3%, signal=2%
PCGF2_TARGET_GENES	43	0.96	1.13	0.008	1	1	45	tags=2%, list=2%, signal=2%
BLANCO_MELO_BRONCHIAL_EPITHELIAL_CELLS_INFLUENZA_A_DEL_NS1_INFECTION_UP	55	0.96	1.12	0.01	1	1	62	tags=2%, list=3%, signal=2%
SOX3_TARGET_GENES	24	0.97	1.15	0.022	1	1	45	tags=4%, list=2%, signal=4%
HNF4_Q6	17	0.98	1.16	0.023	1	1	45	tags=6%, list=2%, signal=6%
GRAESSMANN_APOPTOSIS_BY_SERUM_DEPRIVATION_UP	42	0.96	1.12	0.024	1	1	72	tags=2%, list=3%, signal=2%
WAMUNYOKOLI_OVARIAN_CANCER_LMP_UP	14	0.98	1.17	0.029	1	1	45	tags=7%, list=2%, signal=7%
HAND1E47_01	15	0.98	1.18	0.03	1	1	45	tags=7%, list=2%, signal=7%
GATA1_02	22	0.97	1.14	0.031	1	1	62	tags=5%, list=3%, signal=5%
SANSOM_APC_TARGETS	15	0.97	1.17	0.032	1	1	45	tags=7%, list=2%, signal=7%
GATAAGR_GATA_C	19	0.97	1.15	0.032	1	1	62	tags=5%, list=3%, signal=5%
ZNF610_TARGET_GENES	26	0.96	1.14	0.033	1	1	62	tags=4%, list=3%, signal=4%
SMID_BREAST_CANCER_BASAL_DN	57	0.94	1.09	0.036	1	1	62	tags=2%, list=3%, signal=2%
MIR4699_3P	31	0.96	1.12	0.036	1	1	62	tags=3%, list=3%, signal=3%
WAMUNYOKOLI_OVARIAN_CANCER_GRADES_1_2_UP	12	0.98	1.18	0.038	1	1	45	tags=8%, list=2%, signal=8%
MIR613	17	0.97	1.15	0.038	1	1	62	tags=6%, list=3%, signal=6%
MIR33A_3P	23	0.96	1.14	0.038	1	1	62	tags=4%, list=3%, signal=4%
TATAAA_TATA_01	68	0.93	1.09	0.038	1	1	62	tags=3%, list=3%, signal=3%
CDX2_Q5	14	0.98	1.17	0.039	1	1	45	tags=7%, list=2%, signal=7%
LIEN_BREAST_CARCINOMA_METAPLASTIC_VS_DUCTAL_DN	7	0.98	1.23	0.04	1	1	45	tags=14%, list=2%, signal=15%
POU2AF1_TARGET_GENES	38	0.95	1.11	0.04	1	1	62	tags=3%, list=3%, signal=3%
KUMAR_PATHOGEN_LOAD_BY_MACROPHAGES	9	0.97	1.22	0.042	1	1	45	tags=11%, list=2%, signal=11%
KIM_BIPOLAR_DISORDER_OLIGODENDROCYTE_DENSITY_CORR_UP	18	0.96	1.14	0.043	1	1	72	tags=6%, list=3%, signal=6%
NAB2_TARGET_GENES	36	0.95	1.11	0.043	1	1	62	tags=3%, list=3%, signal=3%
KIM_ALL_DISORDERS_OLIGODENDROCYTE_NUMBER_CORR_UP	24	0.96	1.14	0.045	1	1	72	tags=4%, list=3%, signal=4%
DLX2_TARGET_GENES	16	0.98	1.16	0.045	1	1	45	tags=6%, list=2%, signal=6%
TTTGTAG_MIR520D	18	0.96	1.15	0.046	1	1	62	tags=6%, list=3%, signal=6%
GTGCCTT_MIR506	30	0.95	1.12	0.047	1	1	72	tags=3%, list=3%, signal=3%
KIM_ALL_DISORDERS_CALB1_CORR_UP	27	0.96	1.13	0.048	1	1	72	tags=4%, list=3%, signal=4%
DOANE_RESPONSE_TO_ANDROGEN_DN	30	0.95	1.12	0.048	1	1	72	tags=3%, list=3%, signal=3%

HNF1_C	15	0.97	1.16	0.048	1	1	45	tags=7%, list=2%, signal=7%
MASSARWEH_TAMOXIFEN_RESISTANCE_DN	24	0.96	1.13	0.049	1	1	72	tags=4%, list=3%, signal=4%

Supplementary Table 2B: Downregulated Gene Set Enrichment Analysis for 2478 probes identified by ANOVA with nominal p-value < 0.05.

GS	Size	ES	NES	NOM p-val	FDR q-val	FWER p-val	Rank at Max	Leading Edge
RHEIN_ALL_GLUCOCORTICOID_THERAPY_UP	10	-0.847774	-1.8352331	0	0	0	329	tags=100%, list=16%, signal=118%
JOHANSSON_GLIOMAGENESIS_BY_PDGFB_UP	12	-0.8605654	-1.771203	0	0	0	304	tags=100%, list=14%, signal=116%
KHETCHOUMIAN_TRIM24_TARGETS_UP	7	-0.8585086	-1.7566353	0	0.0001782	0.001	304	tags=100%, list=14%, signal=117%
ODONNELL_TARGETS_OF_MYC_AND_TFRC_DN	12	-0.6847149	-1.7258455	0	0.00017692	0.001	671	tags=100%, list=32%, signal=146%
HINATA_NFKB_TARGETS_KERATINOCYTE_DN	9	-0.7129186	-1.6589348	0	0.00065208	0.004	610	tags=100%, list=29%, signal=140%
WP_HEPATITIS_C_AND_HEPATOCELLULAR_CARCINOMA	7	-0.8465583	-1.6513274	0	0.00077707	0.005	329	tags=100%, list=16%, signal=118%
LEE_LIVER_CANCER_HEPATOBLAST	6	-0.837076	-1.631133	0	0.00129259	0.008	348	tags=100%, list=17%, signal=120%
BLANCO_MELO_INFLUENZA_A_INFECTION_A594_CELLS_UP	6	-0.837076	-1.6310229	0	0.00128362	0.008	348	tags=100%, list=17%, signal=120%
DORN_ADENOVIRUS_INFECTION_24HR_DN	9	-0.7578948	-1.6240602	0	0.00195454	0.012	516	tags=100%, list=25%, signal=132%
XU_GH1_EXOGENOUS_TARGETS_DN	5	-0.8782235	-1.6160276	0	0.00209325	0.013	261	tags=100%, list=12%, signal=114%
KIM_LRRC3B_TARGETS	5	-0.8323782	-1.6126289	0	0.00223547	0.014	357	tags=100%, list=17%, signal=120%
GAURNIER_PSMD4_TARGETS	16	-0.6764283	-1.5920905	0	0.00325447	0.019	691	tags=100%, list=33%, signal=148%
PID_ATF2_PATHWAY	9	-0.7478469	-1.5909554	0	0.00323263	0.019	537	tags=100%, list=26%, signal=134%
REACTOME_SIGNALING_BY_FGFR2	6	-0.8136646	-1.5891135	0	0.00353156	0.021	397	tags=100%, list=19%, signal=123%
REACTOME_CONSTITUTIVE_SIGNALING_BY_ABERRANT_PI3K_IN_CANCER	6	-0.8232203	-1.584975	0	0.00382345	0.023	377	tags=100%, list=18%, signal=122%
PID_CMYB_PATHWAY	6	-0.8461539	-1.5846355	0	0.0037983	0.023	329	tags=100%, list=16%, signal=118%
BOHN_PRIMARY_IMMUNODEFICIENCY_SYNDROM_UP	7	-0.8111855	-1.5806632	0	0.0040669	0.025	403	tags=100%, list=19%, signal=123%
HENDRICKS_SMARCA4_TARGETS_UP	12	-0.767609	-1.5798817	0	0.00404049	0.025	498	tags=100%, list=24%, signal=130%
BURTON_ADIPOGENESIS_2	10	-0.6620393	-1.5778667	0	0.00414845	0.026	717	tags=100%, list=34%, signal=151%
AMIT_EGF_RESPONSE_60_HELA	6	-0.8103201	-1.5757201	0	0.00425502	0.027	404	tags=100%, list=19%, signal=123%
NAGASHIMA_NRG1_SIGNALING_DN	7	-0.7992352	-1.5748599	0	0.00422792	0.027	428	tags=100%, list=20%, signal=125%

JIANG_HYPOXIA_CANCER	6	-0.7998089	-1.5700128	0	0.00432853	0.027	426	tags=100%, list=20%, signal=125%
NGUYEN_NOTCH1_TARGETS_DN	6	-0.7873865	-1.5685278	0	0.00447007	0.028	452	tags=100%, list=22%, signal=127%
WP_PRIMARY_FOCAL_SEGMENTAL_GLOMERULOSCLEROSIS_FSGS	11	-0.6082375	-1.5659679	0	0.00470614	0.03	830	tags=100%, list=40%, signal=165%
REACTOME_CYCLIN_A_B1_B2_ASSOCIATED_EVENTS_DURING_G2_M_TRANSITION	6	-0.812709	-1.560894	0	0.00502649	0.033	399	tags=100%, list=19%, signal=123%
DORN_ADENOVIRUS_INFECTION_48HR_DN	7	-0.7657744	-1.5553398	0	0.00526194	0.035	498	tags=100%, list=24%, signal=131%
MEBARKI_HCC_PROGENITOR_WNT_UP_CTNNB1_DEPENDENT_BLOCKED_BY_FZD8CRD	5	-0.8271251	-1.5422379	0	0.00833559	0.054	368	tags=100%, list=18%, signal=121%
CAFFAREL_RESPONSE_TO_THC_24HR_5_DN	6	-0.8107979	-1.5406023	0	0.00869222	0.058	403	tags=100%, list=19%, signal=123%
MARZEC_IL2_SIGNALING_UP	11	-0.6570881	-1.53125	0	0.00931416	0.062	728	tags=100%, list=35%, signal=152%
DORN_ADENOVIRUS_INFECTION_12HR_DN	7	-0.7657744	-1.5304037	0	0.00937524	0.063	498	tags=100%, list=24%, signal=131%
HERNANDEZ_MITOTIC_ARREST_BY_DOCETAXEL_1_DN	8	-0.736968	-1.5285532	0	0.00957087	0.065	559	tags=100%, list=27%, signal=136%
ONDER_CDH1_TARGETS_3_DN	13	-0.7061362	-1.5280083	0	0.00951424	0.065	627	tags=100%, list=30%, signal=142%
REACTOME_SIGNALING_BY_FGFR	6	-0.8136646	-1.520943	0	0.01053488	0.073	397	tags=100%, list=19%, signal=123%
SATO_SILENCED_BY_DEACETYLATION_IN_PANCREATIC_CANCER	6	-0.7978978	-1.5100919	0	0.01219734	0.084	430	tags=100%, list=20%, signal=125%
BERENJENO_ROCK_SIGNALING_NOT_VIA_RHOA_DN	5	-0.8490927	-1.5082368	0	0.01257279	0.086	322	tags=100%, list=15%, signal=118%
VERHAAK_GLIOBLASTOMA_CLASSICAL	5	-0.8233047	-1.5068544	0	0.01275684	0.088	376	tags=100%, list=18%, signal=122%
SMID_BREAST_CANCER_RELAPSE_IN_PLEURA_DN	5	-0.8323782	-1.5065376	0	0.01268352	0.088	357	tags=100%, list=17%, signal=120%
REACTOME_THE_ROLE_OF_GTSE1_IN_G2_M_PROGRESSION_AFTER_G2_CHECKPOINT	6	-0.7845199	-1.5026147	0	0.01296883	0.091	458	tags=100%, list=22%, signal=128%
FERRARI_RESPONSE_TO_FENRETINIDE_UP	8	-0.7044476	-1.502448	0	0.01289514	0.091	627	tags=100%, list=30%, signal=142%
KRIGE_AMINO_ACID_DEPRIVATION	6	-0.7434305	-1.4983149	0	0.01385359	0.099	544	tags=100%, list=26%, signal=135%
GERHOLD_ADIPOGENESIS_DN	11	-0.6604406	-1.4962026	0	0.01405782	0.101	721	tags=100%, list=34%, signal=152%
CHANG_IMMORTALIZED_BY_HPV31_UP	7	-0.7538241	-1.4915903	0	0.01451218	0.105	523	tags=100%, list=25%, signal=133%
SHEPARD_CRASH_AND_BURN_MUTANT_UP	8	-0.7393591	-1.4915581	0	0.01443156	0.105	554	tags=100%, list=26%, signal=135%
ABBUD_LIF_SIGNALING_1_UP	6	-0.790731	-1.4909121	0	0.01463693	0.106	445	tags=100%, list=21%, signal=127%
PROVENZANI_METASTASIS_DN	11	-0.6681035	-1.4900663	0	0.01467852	0.107	705	tags=100%, list=34%, signal=150%
OLSSON_E2F3_TARGETS_DN	7	-0.7256214	-1.4825023	0	0.01588853	0.117	582	tags=100%, list=28%, signal=138%
REACTOME_UNFOLDED_PROTEIN_RESPONSE_UPR	5	-0.7975167	-1.4821783	0	0.01580218	0.117	430	tags=100%, list=20%, signal=125%

MORI_LARGE_PRE_BII_LYMPHOCYTE_DN	5	-0.8457497	-1.4798034	0	0.0165608 8	0.124	329	tags=100%, list=16%, signal=118%
BROWNE_HCMV_INFECTION_6HR_UP	8	-0.6881875	-1.4767629	0	0.0169019 7	0.127	661	tags=100%, list=31%, signal=145%
REACTOME_AMINO_ACID_TRANSPORT_ACROSS_THE_PLASMA_MEMBRANE	7	-0.75239	-1.4743195	0	0.0170792 2	0.129	526	tags=100%, list=25%, signal=133%
SCHOEN_NFKB_SIGNALING	8	-0.7044476	-1.4734421	0	0.0171152 5	0.13	627	tags=100%, list=30%, signal=142%
BROWNE_HCMV_INFECTION_4HR_UP	10	-0.6807085	-1.4725579	0	0.0173403 2	0.132	678	tags=100%, list=32%, signal=147%
GRAHAM_CML_QUIESCENT_VS_CML_DIVIDING_UP	7	-0.7041109	-1.4723938	0	0.0172490 6	0.132	627	tags=100%, list=30%, signal=142%
KEGG_HEMATOPOIETIC_CELL_LINEAGE	12	-0.6751317	-1.4716942	0	0.0174216 1	0.134	691	tags=100%, list=33%, signal=148%
REACTOME_CHEMOKINE_RECEPTORS_BIND_CHEMOKINES	9	-0.7023923	-1.4698899	0	0.0176631 6	0.136	632	tags=100%, list=30%, signal=142%
REACTOME_SENESCENCE_ASSOCIATED_SECRETORY_PHENOTYPE_SASP	5	-0.8104107	-1.465964	0	0.0180166 7	0.14	403	tags=100%, list=19%, signal=123%
PID_FGF_PATHWAY	5	-0.7960841	-1.4650205	0	0.0181658 5	0.142	433	tags=100%, list=21%, signal=126%
KOKKINAKIS_METHIONINE_DEPRIVATION_96HR_DN	9	-0.6866029	-1.460147	0	0.0188445 7	0.147	665	tags=100%, list=32%, signal=146%
FARMER_BREAST_CANCER_CLUSTER_3	5	-0.7707737	-1.4600102	0	0.0188977 8	0.147	486	tags=100%, list=23%, signal=130%
PID_FOXM1_PATHWAY	10	-0.6567736	-1.4549311	0	0.0199055 8	0.157	728	tags=100%, list=35%, signal=152%
REACTOME_SIGNALING_BY_INSULIN_RECEPTOR	6	-0.7434305	-1.454837	0	0.0198055 5	0.157	544	tags=100%, list=26%, signal=135%
PID_SYNDECAN_2_PATHWAY	6	-0.7606307	-1.4479946	0	0.0217408 8	0.174	508	tags=100%, list=24%, signal=132%
ZHONG_SECRETOME_OF_LUNG_CANCER_AND_MACROPHAGE	9	-0.6674641	-1.4473097	0	0.0218182 2	0.175	705	tags=100%, list=34%, signal=150%
KEGG_OOCYTE_MEIOSIS	9	-0.6564593	-1.4408737	0	0.0234571 9	0.188	728	tags=100%, list=35%, signal=152%
WP_OXIDATIVE_STRESS_RESPONSE	5	-0.7636103	-1.4401433	0	0.0233416 3	0.188	501	tags=100%, list=24%, signal=131%
KEGG_INTESTINAL_IMMUNE_NETWORK_FOR_IGA_PRODUCTION	9	-0.6741627	-1.4387338	0	0.0235618 9	0.191	691	tags=100%, list=33%, signal=148%
KOBAYASHI_EGFR_SIGNALING_6HR_DN	8	-0.661406	-1.4384456	0	0.0235482 9	0.192	717	tags=100%, list=34%, signal=151%
PID_P73PATHWAY	10	-0.6294878	-1.4382064	0	0.0235642 4	0.193	785	tags=100%, list=37%, signal=159%
GAUSSMANN_MLL_AF4_FUSION_TARGETS_A_DN	8	-0.6681014	-1.433997	0	0.0242391	0.197	703	tags=100%, list=33%, signal=150%
RICKMAN_HEAD_AND_NECK_CANCER_B	5	-0.7712512	-1.4245642	0	0.0267481 1	0.218	485	tags=100%, list=23%, signal=130%
HERNANDEZ_MITOTIC_ARREST_BY_DOCETAXEL_1_UP	5	-0.7712512	-1.4194202	0	0.0292766 3	0.236	485	tags=100%, list=23%, signal=130%
GROSS_HYPOXIA_VIA_ELK3_ONLY_UP	6	-0.7477306	-1.4168471	0	0.0300966 3	0.244	535	tags=100%, list=25%, signal=134%
WP_CELL_CYCLE	13	-0.6577181	-1.4151624	0	0.0303748 4	0.248	728	tags=100%, list=35%, signal=152%

WP_HIPPOMERLIN_SIGNALING_DYSREGULATION	9	-0.668421	-1.4149224	0	0.03034888	0.248	703	tags=100%, list=33%, signal=150%
REACTOME_L1CAM_INTERACTIONS	11	-0.5943487	-1.4112965	0	0.03188448	0.259	859	tags=100%, list=41%, signal=168%
PID_AVB3_INTEGRIN_PATHWAY	7	-0.7127151	-1.4096726	0	0.03243943	0.264	609	tags=100%, list=29%, signal=140%
LY_AGING_MIDDLE_DN	9	-0.669378	-1.4093351	0	0.03278426	0.267	701	tags=100%, list=33%, signal=149%
WP_ALLOGRAFT_REJECTION	11	-0.6297892	-1.408677	0	0.03299177	0.268	785	tags=100%, list=37%, signal=159%
KEGG_GRAFT_VERSUS_HOST_DISEASE	9	-0.6291866	-1.4077076	0	0.03335358	0.271	785	tags=100%, list=37%, signal=159%
LY_AGING_OLD_DN	12	-0.6042166	-1.407366	0	0.03346428	0.274	839	tags=100%, list=40%, signal=166%
KANG_IMMORTALIZED_BY_TERT_UP	11	-0.6312261	-1.4043688	0	0.03423474	0.281	782	tags=100%, list=37%, signal=159%
LEE_LIVER_CANCER_DENA_UP	11	-0.6604406	-1.404277	0	0.03408191	0.281	721	tags=100%, list=34%, signal=152%
MIKKELSEN_IPS_ICP_WITH_H3K4ME3_AND_H327ME3	9	-0.623445	-1.4015776	0	0.03496455	0.285	797	tags=100%, list=38%, signal=161%
REACTOME_NON_INTEGRIN_MEMBRANE_ECM_INTERACTIONS	8	-0.6915352	-1.4010395	0	0.0349116	0.285	654	tags=100%, list=31%, signal=145%
YAN_ESCAPE_FROM_ANOIKIS	7	-0.7017208	-1.3980953	0	0.03642494	0.297	632	tags=100%, list=30%, signal=143%
KUNINGER_IGF1_VS_PDGFB_TARGETS_DN	9	-0.6421053	-1.3976741	0	0.03626518	0.297	758	tags=100%, list=36%, signal=156%
HUPER_BREAST_BASAL_VS_LUMINAL_DN	9	-0.6751196	-1.3964152	0	0.0368994	0.303	689	tags=100%, list=33%, signal=148%
NABA_BASEMENT_MEMBRANES	7	-0.6854684	-1.3946561	0	0.03742466	0.307	666	tags=100%, list=32%, signal=146%
SESTO_RESPONSE_TO_UV_C3	5	-0.7382999	-1.3941249	0	0.03726265	0.307	554	tags=100%, list=26%, signal=136%
YAO_TEMPORAL_RESPONSE_TO_PROGESTERONE_CLUSTER_15	7	-0.7127151	-1.3919402	0	0.03803054	0.315	609	tags=100%, list=29%, signal=140%
WP_FOLATE_METABOLISM	10	-0.6601245	-1.3898875	0	0.03906613	0.325	721	tags=100%, list=34%, signal=152%
GROSS_HYPOXIA_VIA_ELK3_AND_HIF1A_DN	10	-0.6156055	-1.3894119	0	0.03942649	0.33	814	tags=100%, list=39%, signal=163%
WP_COMPLEMENT_AND_COAGULATION_CASCADES	7	-0.6591778	-1.3874403	0	0.04021879	0.337	721	tags=100%, list=34%, signal=152%
TIAN_TNF_SIGNALING_VIA_NFKB	11	-0.6178161	-1.3841202	0	0.04154407	0.348	810	tags=100%, list=39%, signal=162%
WP_MEASLES_VIRUS_INFECTION	11	-0.6178161	-1.3826367	0	0.04174328	0.352	810	tags=100%, list=39%, signal=162%
REACTOME_CELLULAR_SENESCENCE	8	-0.6346246	-1.378536	0	0.04365777	0.361	773	tags=100%, list=37%, signal=158%
COATES_MACROPHAGE_M1_VS_M2_UP	9	-0.6473684	-1.3778005	0	0.04380005	0.364	747	tags=100%, list=36%, signal=155%
WP_CYTOKINES_AND_INFLAMMATORY_RESPONSE	7	-0.6931166	-1.3737907	0	0.04534768	0.374	650	tags=100%, list=31%, signal=144%
KEGG_SYSTEMIC_LUPUS_ERYTHEMATOSUS	8	-0.6738403	-1.3732944	0	0.04572133	0.376	691	tags=100%, list=33%, signal=149%

WP_SPINAL_CORD_INJURY	9	-0.6626794	-1.3718693	0	0.0460526 5	0.381	715	tags=100%, list=34%, signal=151%
WP_PHOTODYNAMIC_THERAPYINDUCED_NFKB_SURVIVAL_SIGNALING	7	-0.6931166	-1.3684409	0	0.0475184 4	0.389	650	tags=100%, list=31%, signal=144%
ZHENG_IL22_SIGNALING_UP	7	-0.6591778	-1.3666997	0	0.0478517 2	0.392	721	tags=100%, list=34%, signal=152%
HOWLIN_PUBERTAL_MAMMARY_GLAND	7	-0.6931166	-1.3658628	0	0.0481321 5	0.394	650	tags=100%, list=31%, signal=144%
REACTOME_G0_AND_EARLY_G1	7	-0.6558318	-1.3653831	0	0.0482598	0.396	728	tags=100%, list=35%, signal=153%
DAZARD_UV_RESPONSE_CLUSTER_G28	7	-0.7041109	-1.3629875	0	0.0494415 7	0.408	627	tags=100%, list=30%, signal=142%
VANTVEER_BREAST_CANCER_POOR_PROGNOSIS	11	-0.5914751	-1.3616318	0	0.0497666 2	0.412	865	tags=100%, list=41%, signal=169%
WP_PROSTAGLANDIN_SYNTHESIS_AND_REGULATION	9	-0.6244019	-1.3591727	0	0.0506221 3	0.422	795	tags=100%, list=38%, signal=160%
DAZARD_UV_RESPONSE_CLUSTER_G1	5	-0.743553	-1.3573698	0	0.0514085 2	0.428	543	tags=100%, list=26%, signal=135%
REACTOME_APC_C_MEDIATED_DEGRADATION_OF_CELL_CYCLE_PROTEINS	8	-0.7063606	-1.3537152	0	0.0540239 7	0.445	623	tags=100%, list=30%, signal=142%
WP_VITAMIN_B12_METABOLISM	9	-0.6598086	-1.3504477	0	0.0555844 9	0.456	721	tags=100%, list=34%, signal=152%
DER_IFN_ALPHA_RESPONSE_UP	11	-0.5694444	-1.3496027	0	0.0561886 4	0.46	911	tags=100%, list=43%, signal=176%
WP_MIRNAS_INVOLVEMENT_IN_THE_IMMUNE_RESPONSE_IN_SEPSIS	6	-0.6927855	-1.3465049	0	0.0578229 8	0.472	650	tags=100%, list=31%, signal=144%
PETROVA_PROX1_TARGETS_UP	8	-0.6810139	-1.3457768	0	0.0578707 5	0.473	676	tags=100%, list=32%, signal=147%
GLASS_IGF2BP1_CLIP_TARGETS_KNOCKDOWN_DN	12	-0.5692381	-1.3442715	0	0.0581439 2	0.477	912	tags=100%, list=43%, signal=176%
WP_SARSCOV2_INNATE_IMMUNITY_EVASION_AND_CELLSPECIFIC_IMMUNE_RESPONSE	9	-0.6937799	-1.3399339	0	0.0600234 2	0.493	650	tags=100%, list=31%, signal=144%
KOKKINAKIS_METHIONINE_DEPRIVATION_48HR_DN	5	-0.7244508	-1.3352674	0	0.0626814 7	0.514	583	tags=100%, list=28%, signal=138%
KEGG_TYPE_I_DIABETES_MELLITUS	8	-0.6288857	-1.3315108	0	0.0653409 7	0.531	785	tags=100%, list=37%, signal=159%
HONRADO_BREAST_CANCER_BRCA1_VS_BRCA2	6	-0.6555184	-1.3304372	0	0.0657309 9	0.534	728	tags=100%, list=35%, signal=153%
GAJATE_RESPONSE_TO_TRABECTEDIN_UP	12	-0.6181121	-1.3271604	0	0.0675523 7	0.549	810	tags=100%, list=39%, signal=162%
CROMER_METASTASIS_DN	8	-0.6389288	-1.3248711	0	0.0690503 3	0.56	764	tags=100%, list=36%, signal=157%
WP_PROSTAGLANDIN_SIGNALING	7	-0.6931166	-1.3246197	0	0.0688973 8	0.56	650	tags=100%, list=31%, signal=144%
MARTIN_NFKB_TARGETS_UP	6	-0.6956522	-1.3154362	0	0.0735020 6	0.59	644	tags=100%, list=31%, signal=144%
NATSUME_RESPONSE_TO_INTERFERON_BETA_UP	9	-0.6291866	-1.31309	0	0.0753203 4	0.599	785	tags=100%, list=37%, signal=159%
ZHANG_PROLIFERATING_VS_QUIESCENT	6	-0.7037745	-1.3074113	0	0.0780762	0.615	627	tags=100%, list=30%, signal=142%
REACTOME_SIGNALING_BY_MET	7	-0.6419694	-1.3068113	0	0.0780917 7	0.617	757	tags=100%, list=36%, signal=156%

WESTON_VEGFA_TARGETS_3HR	10	-0.6309239	-1.3054675	0	0.0783155 7	0.62	782	tags=100%, list=37%, signal=159%
CROMER_TUMORIGENESIS_UP	9	-0.6172249	-1.3054478	0	0.0780464 5	0.62	810	tags=100%, list=39%, signal=162%
WP_ACUTE_VIRAL_MYOCARDITIS	6	-0.6927855	-1.3042728	0	0.0781012 1	0.622	650	tags=100%, list=31%, signal=144%
HOEBEKE_LYMPHOID_STEM_CELL_UP	8	-0.6169297	-1.3025918	0	0.0780369	0.626	810	tags=100%, list=39%, signal=162%
BROWN_MYELOID_CELL_DEVELOPMENT_DN	9	-0.6588517	-1.3012664	0	0.0788691	0.634	723	tags=100%, list=34%, signal=152%
IZADPANAH_STEM_CELL_ADIPOSE_VS_BONE_DN	11	-0.5215517	-1.3010753	0	0.0785118 6	0.635	1011	tags=100%, list=48%, signal=192%
HUANG_FOXA2_TARGETS_DN	9	-0.6598086	-1.3009434	0	0.0783280 4	0.635	721	tags=100%, list=34%, signal=152%
BOSCO_EPITHELIAL_DIFFERENTIATION_MODULE	11	-0.5823755	-1.2968361	0	0.0805127	0.649	884	tags=100%, list=42%, signal=172%
WANG_BARRETTS_ESOPHAGUS_AND_ESOPHAGUS_CANCER_DN	5	-0.6957975	-1.2948436	0	0.0811914 3	0.654	643	tags=100%, list=31%, signal=144%
PUIFFE_INVASION_INHIBITED_BY_ASCITES_UP	17	-0.5043228	-1.294698	0	0.0809975 2	0.655	1050	tags=100%, list=50%, signal=198%
BROWNE_INTERFERON_RESPONSIVE_GENES	11	-0.5675287	-1.288744	0	0.0843351 8	0.677	915	tags=100%, list=44%, signal=176%
REACTOME_TP53_REGULATES_TRANSCRIPTION_OF_CELL_CYCLE_GENES	8	-0.6561454	-1.2871752	0	0.0849525 9	0.682	728	tags=100%, list=35%, signal=153%
WP_PHOTODYNAMIC_THERAPYINDUCED_AP1_SURVIVAL_SIGNALING	8	-0.6288857	-1.2852556	0	0.0863722 2	0.688	785	tags=100%, list=37%, signal=159%
LINDVALL_IMMORTALIZED_BY_TERT_DN	11	-0.532567	-1.2840647	0	0.0867493 1	0.688	988	tags=100%, list=47%, signal=188%
REACTOME_INTERFERON_ALPHA_BETA_SIGNALING	10	-0.5466731	-1.2835066	0	0.0867003 5	0.688	958	tags=100%, list=46%, signal=183%
BUFFA_HYPOXIA_METAGENE	8	-0.6331899	-1.2823244	0	0.0865399 3	0.693	776	tags=100%, list=37%, signal=158%
SWEET_KRAS_TARGETS_DN	10	-0.6007659	-1.2797418	0	0.0887723 3	0.706	845	tags=100%, list=40%, signal=167%
TSAI_RESPONSE_TO_RADIATION_THERAPY	7	-0.6591778	-1.2792209	0	0.0887223 3	0.706	721	tags=100%, list=34%, signal=152%
PROVENZANI_METASTASIS_UP	9	-0.6244019	-1.276908	0	0.0905142 4	0.712	795	tags=100%, list=38%, signal=160%
PID_TAP63_PATHWAY	9	-0.6291866	-1.274688	0	0.0919105	0.719	785	tags=100%, list=37%, signal=159%
LEE_AGING_CEREBELLUM_DN	8	-0.6107126	-1.2734344	0	0.0923389 8	0.724	823	tags=100%, list=39%, signal=164%
REACTOME_MET_PROMOTES_CELL_MOTILITY	7	-0.6419694	-1.2730906	0	0.0923086	0.725	757	tags=100%, list=36%, signal=156%
WIEDERSCHAIN_TARGETS_OF_BMI1_AND_PCGF2	15	-0.5268714	-1.2708334	0	0.0946034 4	0.732	1002	tags=100%, list=48%, signal=190%
MIKKELSEN_ES_ICP_WITH_H3K4ME3_AND_H3K27ME3	11	-0.6757663	-1.2702557	0	0.0948770 9	0.736	689	tags=100%, list=33%, signal=148%
KEGG_ALLOGRAFT_REJECTION	8	-0.6288857	-1.2675776	0	0.0966451 3	0.746	785	tags=100%, list=37%, signal=159%
MORI_MATURE_B_LYMPHOCYTE_DN	8	-0.5973219	-1.2656112	0	0.0982641 9	0.759	851	tags=100%, list=41%, signal=168%

RADMACHER_AML_PROGNOSIS	9	-0.5856459	-1.2650229	0	0.0983394 4	0.759	876	tags=100%, list=42%, signal=171%
REACTOME_SIGNALING_BY_PTK6	6	-0.6555184	-1.264043	0	0.0985469 5	0.763	728	tags=100%, list=35%, signal=153%
KEGG_NOD_LIKE_RECEPTOR_SIGNALING_PATHWAY	9	-0.6172249	-1.2639314	0	0.0983208 6	0.763	810	tags=100%, list=39%, signal=162%
REICHERT_MITOSIS_LIN9_TARGETS	12	-0.5059894	-1.2639136	0	0.0980358 7	0.763	1044	tags=100%, list=50%, signal=198%
GRAHAM_NORMAL_QUIESCENT_VS_NORMAL_DIVIDING_UP	11	-0.6187739	-1.2633638	0	0.0981992 6	0.763	808	tags=100%, list=38%, signal=162%
SMID_BREAST_CANCER_LUMINAL_A_DN	9	-0.5684211	-1.263062	0	0.0981998 4	0.763	912	tags=100%, list=43%, signal=176%
REACTOME_TRANSPORT_TO_THE_GOLGI_AND_SUBSEQUENT_MODIFICATION	10	-0.6591671	-1.2621448	0	0.0983964 5	0.764	723	tags=100%, list=34%, signal=152%
SEMENZA_HIF1_TARGETS	7	-0.7036329	-1.2584424	0	0.1016250 5	0.78	628	tags=100%, list=30%, signal=142%
GAUSSMANN_MLL_AF4_FUSION_TARGETS_A_UP	12	-0.504552	-1.2580645	0	0.1016305 4	0.781	1047	tags=100%, list=50%, signal=198%
KEGG_AUTOIMMUNE_THYROID_DISEASE	7	-0.6285851	-1.2570844	0	0.1021604 2	0.782	785	tags=100%, list=37%, signal=159%
WEST_ADRENOCORTICAL_TUMOR_MARKERS_UP	9	-0.5909091	-1.2554739	0	0.1030323 1	0.786	865	tags=100%, list=41%, signal=169%
PID_INTEGRIN1_PATHWAY	11	-0.5627394	-1.2553418	0	0.1024534 6	0.786	925	tags=100%, list=44%, signal=178%
REACTOME_SCF_SKP2_MEDIATED_DEGRADATION_OF_P27_P21	5	-0.6552054	-1.2464771	0	0.1085261 2	0.806	728	tags=100%, list=35%, signal=153%
WANG_METHYLATED_IN_BREAST_CANCER	9	-0.5698565	-1.2449478	0	0.1103180 7	0.816	909	tags=100%, list=43%, signal=176%
BECKER_TAMOXIFEN_RESISTANCE_DN	9	-0.5837321	-1.2436291	0	0.1109045 9	0.821	880	tags=100%, list=42%, signal=171%
FRIDMAN_SENESCENCE_UP	14	-0.4661871	-1.2429668	0	0.1109413 8	0.821	1128	tags=100%, list=54%, signal=215%
FUJII_YBX1_TARGETS_UP	9	-0.592823	-1.2377623	0	0.1153972 7	0.834	861	tags=100%, list=41%, signal=169%
HOLLERN_EMT_BREAST_TUMOR_DN	10	-0.5945429	-1.2376682	0	0.1148359	0.834	858	tags=100%, list=41%, signal=168%
ZHU_CMV_ALL_UP	13	-0.5699904	-1.2308488	0	0.1199478 2	0.846	911	tags=100%, list=43%, signal=176%
REACTOME_G1_S_SPECIFIC_TRANSCRIPTION	6	-0.6555184	-1.2299608	0	0.1204919 3	0.85	728	tags=100%, list=35%, signal=153%
TAKEDA_TARGETS_OF_NUP98_HOXA9_FUSION_16D_UP	18	-0.541086	-1.2292576	0	0.1208011 4	0.85	974	tags=100%, list=46%, signal=185%
AMIT_SERUM_RESPONSE_240_MCF10A	10	-0.5691719	-1.2219938	0	0.1272387 8	0.865	911	tags=100%, list=43%, signal=176%
PID_AURORA_B_PATHWAY	9	-0.5684211	-1.2189617	0	0.1298735 1	0.875	912	tags=100%, list=43%, signal=176%
WP_COMPLEMENT_SYSTEM_IN_NEURONAL_DEVELOPMENT_AND_PLASTICITY	9	-0.6291866	-1.2183446	0	0.1299574 5	0.877	785	tags=100%, list=37%, signal=159%
WESTON_VEGFA_TARGETS_6HR	9	-0.630622	-1.2177395	0	0.1302919 8	0.88	782	tags=100%, list=37%, signal=159%
FONTAINE_FOLLICULAR_THYROID_ADENOMA_DN	10	-0.570608	-1.2167404	0	0.1309002 5	0.882	908	tags=100%, list=43%, signal=175%

WP_APOPTOSIS	6	-0.6282848	-1.2148101	0	0.13118456	0.888	785	tags=100%, list=37%, signal=159%
RIZ_ERYTHROID_DIFFERENTIATION	6	-0.6144291	-1.2086465	0	0.13613762	0.905	814	tags=100%, list=39%, signal=163%
NABA_ECM_REGULATORS	17	-0.5230547	-1.207317	0	0.13770057	0.908	1011	tags=100%, list=48%, signal=191%
MCBRYAN_PUBERTAL_BREAST_4_5WK_DN	13	-0.5172579	-1.2002225	0	0.14685811	0.921	1021	tags=100%, list=49%, signal=194%
NAKAMURA_METASTASIS_MODEL_DN	8	-0.5868006	-1.1999023	0	0.14692962	0.921	873	tags=100%, list=42%, signal=171%
REN_BOUND_BY_E2F	9	-0.5406699	-1.1909362	0	0.15516794	0.941	970	tags=100%, list=46%, signal=185%
WP_TGFBETA_SIGNALING_PATHWAY	13	-0.5091084	-1.1879195	0	0.15792254	0.943	1038	tags=100%, list=49%, signal=197%
SMITH_TERT_TARGETS_UP	11	-0.525862	-1.1878831	0	0.15765512	0.943	1002	tags=100%, list=48%, signal=190%
SHEDDEN_LUNG_CANCER_GOOD_SURVIVAL_A12	9	-0.6267943	-1.1870244	0	0.15832219	0.945	790	tags=100%, list=38%, signal=160%
KIM_BIPOLAR_DISORDER_OLIGODENDROCYTE_DENSITY_CORR_DN	11	-0.566092	-1.1851605	0	0.15817729	0.946	918	tags=100%, list=44%, signal=177%
KOKKINAKIS_METHIONINE_DEPRIVATION_48HR_UP	18	-0.502643	-1.184598	0	0.15771703	0.946	1054	tags=100%, list=50%, signal=199%
NAKAYAMA_SOFT_TISSUE_TUMORS_PCA1_UP	13	-0.5014381	-1.1832578	0	0.1591416	0.949	1054	tags=100%, list=50%, signal=200%
HUANG_GATA2_TARGETS_UP	10	-0.5361417	-1.1799409	0	0.16238935	0.955	980	tags=100%, list=47%, signal=187%
BYSTRYKH_HEMATOPOIESIS_STEM_CELL_AND_BRAIN_QTL_TRANS	8	-0.5868006	-1.1666522	0	0.1802113	0.972	873	tags=100%, list=42%, signal=171%
TSUNODA_CISPLATIN_RESISTANCE_DN	9	-0.562201	-1.1662532	0	0.18017624	0.972	925	tags=100%, list=44%, signal=178%
PLASARI_TGFB1_SIGNALING_VIA_NFIC_10HR_UP	11	-0.5043103	-1.1648229	0	0.18133463	0.973	1047	tags=100%, list=50%, signal=198%
GRUETZMANN_PANCREATIC_CANCER_DN	12	-0.5433637	-1.1612904	0	0.18504575	0.978	966	tags=100%, list=46%, signal=184%
REACTOME_ASPARAGINE_N_LINKED_GLYCOSYLATION	13	-0.5369127	-1.1576227	0	0.18920608	0.98	980	tags=100%, list=47%, signal=186%
HOLLERN_EMT_BREAST_TUMOR_UP	14	-0.4752998	-1.1563594	0	0.19007094	0.98	1109	tags=100%, list=53%, signal=211%
WP_G1_TO_S_CELL_CYCLE_CONTROL	8	-0.5404113	-1.1527084	0	0.19353563	0.981	970	tags=100%, list=46%, signal=185%
GHANDHI_BYSTANDER_IRRADIATION_UP	17	-0.5043228	-1.1525797	0	0.19278948	0.981	1050	tags=100%, list=50%, signal=198%
WANG_ESOPHAGUS_CANCER_VS_NORMAL_UP	14	-0.5472422	-1.1525253	0	0.1925398	0.981	959	tags=100%, list=46%, signal=183%
ZHANG_GATA6_TARGETS_DN	9	-0.5545455	-1.1486621	0	0.19818972	0.983	941	tags=100%, list=45%, signal=180%
GEISS_RESPONSE_TO_DSRNA_UP	9	-0.5688995	-1.1476834	0	0.19918889	0.984	911	tags=100%, list=43%, signal=176%
MEBARKI_HCC_PROGENITOR_WNT_UP_BLOCKED_BY_FZD8CRD	14	-0.4729017	-1.1465116	0	0.2009665	0.984	1114	tags=100%, list=53%, signal=212%
PID_FRA_PATHWAY	9	-0.5004785	-1.1456736	0	0.20227839	0.984	1054	tags=100%, list=50%, signal=200%

HESS_TARGETS_OF_HOXA9_AND_MEIS1_DN	8	-0.5456719	-1.1450896	0	0.2022695	0.984	959	tags=100%, list=46%, signal=183%
ZHU_CMV_8_HR_UP	8	-0.5686275	-1.1403909	0	0.20768361	0.987	911	tags=100%, list=43%, signal=176%
CROONQUIST_STROMAL_STIMULATION_UP	11	-0.5215517	-1.1397175	0	0.20838666	0.987	1011	tags=100%, list=48%, signal=192%
VANDESLUIS_COMMD1_TARGETS_GROUP_3_UP	13	-0.5671141	-1.1342282	0	0.21648875	0.989	917	tags=100%, list=44%, signal=176%
FRASOR_RESPONSE_TO_SERM_OR_FULVESTRANT_DN	9	-0.5406699	-1.1248258	0	0.2298857	0.993	970	tags=100%, list=46%, signal=185%
PEDERSEN_TARGETS_OF_611CTF_ISOFORM_OF_ERBB2	12	-0.4882607	-1.1213205	0	0.23208572	0.993	1081	tags=100%, list=52%, signal=205%
REACTOME_RESOLUTION_OF_SISTER_CHROMATID_COHESION	14	-0.4628297	-1.1188406	0	0.23582657	0.995	1135	tags=100%, list=54%, signal=216%
YAO_TEMPORAL_RESPONSE_TO_PROGESTERONE_CLUSTER_0	8	-0.5208035	-1.1152709	0	0.23996113	0.995	1011	tags=100%, list=48%, signal=192%
VANOEVELEN_MYOGENESIS_SIN3A_TARGETS	10	-0.5394926	-1.1119882	0	0.24389327	0.997	973	tags=100%, list=46%, signal=186%
WP_BURN_WOUND_HEALING	14	-0.5016787	-1.1045406	0	0.25548077	0.999	1054	tags=100%, list=50%, signal=200%
KATSANOU_ELAVL1_TARGETS_DN	16	-0.4891983	-1.094522	0	0.26725575	0.999	1081	tags=100%, list=52%, signal=205%
CHYLA_CBFA2T3_TARGETS_DN	15	-0.5158349	-1.0908169	0	0.2706672	1	1025	tags=100%, list=49%, signal=194%
REACTOME_CELL_CELL_COMMUNICATION	10	-0.5174725	-1.0897176	0	0.2725173	1	1019	tags=100%, list=49%, signal=193%
BILD_MYC_ONCOGENIC_SIGNATURE	12	-0.4729276	-1.089404	0	0.27279353	1	1113	tags=100%, list=53%, signal=212%
SESTO_RESPONSE_TO_UV_C7	10	-0.4968885	-1.0880502	0	0.27403447	1	1062	tags=100%, list=51%, signal=201%
REACTOME_M_PHASE	19	-0.4639423	-1.0879369	0	0.2736934	1	1135	tags=100%, list=54%, signal=216%
ACEVEDO_LIVER_CANCER_WITH_H3K9ME3_UP	10	-0.5390139	-1.0754536	0	0.29303846	1	974	tags=100%, list=46%, signal=186%
GRAHAM_CML_QUIESCENT_VS_NORMAL_QUIESCENT_DN	11	-0.5167624	-1.0662055	0	0.3062288	1	1021	tags=100%, list=49%, signal=194%
LEE_NEURAL_CREST_STEM_CELL_DN	13	-0.4209012	-1.065534	0	0.3066606	1	1222	tags=100%, list=58%, signal=238%
REACTOME_MITOTIC_G2_G2_M_PHASES	12	-0.5059894	-1.0634441	0	0.30974972	1	1044	tags=100%, list=50%, signal=198%
DELPUECH_FOXO3_TARGETS_UP	11	-0.5704023	-1.0577264	0	0.32034773	1	909	tags=100%, list=43%, signal=175%
RAO_BOUND_BY_SALL4_ISOFORM_A	12	-0.4628654	-1.0545852	0	0.3239162	1	1134	tags=100%, list=54%, signal=216%
WP_NEUROINFLAMMATION_AND_GLUTAMATERGIC_SIGNALING	11	-0.4583333	-1.0539647	0	0.32339194	1	1143	tags=100%, list=54%, signal=218%
IBRAHIM_NRF1_UP	13	-0.5033557	-1.0479041	0	0.33468735	1	1050	tags=100%, list=50%, signal=199%
ISSAEVA_MLL2_TARGETS	11	-0.4942529	-1.041898	0	0.3454207	1	1068	tags=100%, list=51%, signal=203%
REACTOME_SEPARATION_OF_SISTER_CHROMATIDS	12	-0.4623862	-1.0393107	0	0.34791702	1	1135	tags=100%, list=54%, signal=216%

BROWNE_HCMV_INFECTION_30MIN_DN	13	-0.4352829	-1.0347579	0	0.3558717 7	1	1192	tags=100%, list=57%, signal=230%
KAAB_HEART_ATRIUM_VS_VENTRICLE_DN	10	-0.5131642	-1.0312649	0	0.3630679 5	1	1028	tags=100%, list=49%, signal=195%
DAVICIONI_TARGETS_OF_PAX_FOXO1_FUSIONS_DN	11	-0.4415709	-1.0076503	0	0.4037818 6	1	1178	tags=100%, list=56%, signal=227%
FONTAINE_PAPILLARY_THYROID_CARCINOMA_UP	12	-0.5093436	-1.007583	0	0.4033043 4	1	1037	tags=100%, list=49%, signal=197%
THRA_TARGET_GENES	5	-0.8939828	-1.6503086	0	0.0015491 8	0.006	228	tags=100%, list=11%, signal=112%
MIR3913_3P	5	-0.8634193	-1.5859114	0	0.0031972 7	0.013	292	tags=100%, list=14%, signal=116%
MIR7150	7	-0.8202677	-1.567696	0	0.0051583 5	0.021	384	tags=100%, list=18%, signal=122%
MIR4778_5P	6	-0.8088868	-1.5386713	0	0.0105124 2	0.042	407	tags=100%, list=19%, signal=124%
STAT6_01	9	-0.7392345	-1.4946791	0	0.0216217 9	0.087	555	tags=100%, list=26%, signal=135%
NR1I2_TARGET_GENES	6	-0.7410416	-1.4775078	0	0.0253923 7	0.104	549	tags=100%, list=26%, signal=135%
PAX4_04	6	-0.7744864	-1.4550031	0	0.0354441 3	0.143	479	tags=100%, list=23%, signal=129%
MIR6894_3P	6	-0.7448639	-1.4477795	0	0.0409482 1	0.163	541	tags=100%, list=26%, signal=134%
MIR6840_3P	8	-0.6929699	-1.4470103	0	0.0410238	0.166	651	tags=100%, list=31%, signal=144%
MIR1825	8	-0.7450981	-1.445567	0	0.0409821 2	0.169	542	tags=100%, list=26%, signal=134%
MIR4772_3P	8	-0.7455763	-1.4451911	0	0.0404283 1	0.169	541	tags=100%, list=26%, signal=134%
MIR6854_5P	7	-0.7366157	-1.4439901	0	0.0403849 3	0.171	559	tags=100%, list=27%, signal=136%
MIR6845_3P	6	-0.7448639	-1.4335632	0	0.0451536 4	0.191	541	tags=100%, list=26%, signal=134%
MIR1261	6	-0.7477306	-1.4293743	0	0.0462682 9	0.198	535	tags=100%, list=25%, signal=134%
MIR150_5P	13	-0.6313519	-1.4253247	0	0.0479744 9	0.209	783	tags=100%, list=37%, signal=159%
MIR4693_5P	9	-0.6688995	-1.4152663	0	0.0527509 2	0.231	702	tags=100%, list=33%, signal=150%
MIR4432	8	-0.6637973	-1.4079119	0	0.0558934 6	0.251	712	tags=100%, list=34%, signal=151%
MIR136_5P	6	-0.6923077	-1.3922956	0	0.0663257 8	0.29	651	tags=100%, list=31%, signal=145%
MIR3679_5P	5	-0.7473735	-1.3844526	0	0.0711653 8	0.311	535	tags=100%, list=25%, signal=134%
MIR4762_5P	7	-0.7265775	-1.381051	0	0.0737562 3	0.324	580	tags=100%, list=28%, signal=138%
TCF1P_Q6	18	-0.5430082	-1.3797313	0	0.0744528 6	0.329	970	tags=100%, list=46%, signal=184%
MIR342_3P	6	-0.6923077	-1.3738939	0	0.0773816 1	0.343	651	tags=100%, list=31%, signal=145%

MIR6750_3P	6	-0.7209747	-1.346258	0	0.10303274	0.435	591	tags=100%, list=28%, signal=139%
MIR6732_3P	5	-0.7507163	-1.3437364	0	0.10459654	0.443	528	tags=100%, list=25%, signal=133%
MIR501_5P	7	-0.6945507	-1.337095	0	0.11135142	0.465	647	tags=100%, list=31%, signal=144%
MIR4441	9	-0.6339713	-1.326085	0	0.12071741	0.496	775	tags=100%, list=37%, signal=158%
MIR146A_3P	9	-0.5976077	-1.3202959	0	0.12577376	0.514	851	tags=100%, list=41%, signal=167%
MIER1_TARGET_GENES	9	-0.6229665	-1.3201522	0	0.12489023	0.516	798	tags=100%, list=38%, signal=161%
MIR1200	9	-0.5703349	-1.3189489	0	0.12525037	0.521	908	tags=100%, list=43%, signal=175%
HOXA10_TARGET_GENES	8	-0.6260163	-1.3164198	0	0.12764107	0.53	791	tags=100%, list=38%, signal=160%
MIR199A_5P	11	-0.6091954	-1.3129646	0	0.13057512	0.543	828	tags=100%, list=39%, signal=164%
MIR199B_5P	11	-0.6091954	-1.2900609	0	0.16410674	0.636	828	tags=100%, list=39%, signal=164%
MIR12122	10	-0.6673049	-1.2895468	0	0.16306613	0.638	706	tags=100%, list=34%, signal=150%
MIR4713_5P	9	-0.645933	-1.288783	0	0.16320845	0.642	750	tags=100%, list=36%, signal=155%
MIR6851_5P	7	-0.6075526	-1.2854614	0	0.16673319	0.658	829	tags=100%, list=39%, signal=165%
MIR4736	6	-0.667463	-1.2703369	0	0.18854454	0.711	703	tags=100%, list=33%, signal=150%
NKX62_Q2	9	-0.6110048	-1.2338164	0	0.24422297	0.809	823	tags=100%, list=39%, signal=164%
AACTGAC_MIR223	9	-0.5401914	-1.2290442	0	0.2439731	0.816	971	tags=100%, list=46%, signal=185%
OCT1_06	13	-0.5186961	-1.2235205	0	0.24544372	0.834	1018	tags=100%, list=48%, signal=193%
PHF21A_TARGET_GENES	12	-0.5467178	-1.2164179	0	0.25593317	0.854	959	tags=100%, list=46%, signal=183%
MIR1236_5P	8	-0.5829747	-1.2154641	0	0.256466	0.857	881	tags=100%, list=42%, signal=172%
MIR183_3P	10	-0.5457156	-1.2147043	0	0.25415137	0.858	960	tags=100%, list=46%, signal=183%
MIR504_3P	8	-0.5667145	-1.2144948	0	0.25269553	0.858	915	tags=100%, list=44%, signal=177%
GGCAGTG_MIR3243P	10	-0.5380565	-1.2097584	0	0.26462284	0.872	976	tags=100%, list=46%, signal=186%
AGGGCAG_MIR18A	8	-0.58967	-1.1936108	0	0.29858178	0.907	867	tags=100%, list=41%, signal=170%
H1_6_TARGET_GENES	15	-0.4702495	-1.1878787	0	0.31086534	0.914	1120	tags=100%, list=53%, signal=213%
MIR4513	7	-0.5664436	-1.1744947	0	0.33044213	0.94	915	tags=100%, list=44%, signal=177%
OCT1_03	14	-0.5016787	-1.1739619	0	0.32736698	0.94	1054	tags=100%, list=50%, signal=200%

MIR140_3P	11	-0.5943487	-1.1724138	0	0.32731876	0.941	859	tags=100%, list=41%, signal=168%
MIR199A_3P_MIR199B_3P	13	-0.4971237	-1.1708694	0	0.32419083	0.941	1063	tags=100%, list=51%, signal=201%
HSF1_01	10	-0.5165151	-1.169376	0	0.32125074	0.944	1021	tags=100%, list=49%, signal=194%
AAANWWTGC_UNKNOWN	12	-0.5184475	-1.1634408	0	0.33060342	0.949	1018	tags=100%, list=48%, signal=193%
MIR5703	8	-0.5461502	-1.1552857	0	0.350425	0.959	958	tags=100%, list=46%, signal=183%
SRF_Q5_01	11	-0.5177203	-1.1528617	0	0.35539836	0.959	1019	tags=100%, list=49%, signal=193%
GTCTTCC_MIR7	9	-0.5425837	-1.1518537	0	0.35610035	0.961	966	tags=100%, list=46%, signal=184%
MIR6853_3P	12	-0.4992813	-1.147156	0	0.3638958	0.964	1058	tags=100%, list=50%, signal=200%
STAT1_03	9	-0.5425837	-1.1431451	0	0.37242222	0.967	966	tags=100%, list=46%, signal=184%
RSRFC4_Q2	9	-0.5086125	-1.1430109	0	0.37057272	0.968	1037	tags=100%, list=49%, signal=197%
NGFIC_01	11	-0.5124521	-1.1421164	0	0.37111315	0.968	1030	tags=100%, list=49%, signal=195%
MIR1323	13	-0.4635666	-1.1416764	0	0.3692188	0.968	1133	tags=100%, list=54%, signal=216%
IPF1_Q4	14	-0.5016787	-1.140676	0	0.37055224	0.97	1054	tags=100%, list=50%, signal=200%
GCCNNNWTAAR_UNKNOWN	12	-0.5184475	-1.1385479	0	0.37228796	0.971	1018	tags=100%, list=48%, signal=193%
GATA_Q6	13	-0.4913711	-1.1342678	0	0.36779633	0.975	1075	tags=100%, list=51%, signal=204%
RSRFC4_01	11	-0.5090996	-1.1320554	0	0.3701384	0.977	1037	tags=100%, list=49%, signal=197%
AHRARNT_01	8	-0.5423242	-1.1310183	0	0.36872226	0.977	966	tags=100%, list=46%, signal=185%
AP3_Q6	13	-0.4947268	-1.125409	0	0.373987	0.982	1068	tags=100%, list=51%, signal=202%
MIR892C_3P	12	-0.4638237	-1.1225358	0	0.37731382	0.983	1132	tags=100%, list=54%, signal=216%
MIR212_3P	12	-0.4810733	-1.1224147	0	0.37557012	0.983	1096	tags=100%, list=52%, signal=208%
TATA_C	11	-0.5181992	-1.1208563	0	0.37560332	0.984	1018	tags=100%, list=48%, signal=193%
MIR7153_5P	9	-0.4779904	-1.116617	0	0.38515672	0.985	1101	tags=100%, list=52%, signal=209%
MIR6818_3P	12	-0.5098227	-1.1129707	0	0.3935358	0.987	1036	tags=100%, list=49%, signal=196%
MIR589_3P	18	-0.4425757	-1.1056423	0	0.40713933	0.988	1179	tags=100%, list=56%, signal=226%
MIR4533	15	-0.456334	-1.1045296	0	0.40422422	0.988	1149	tags=100%, list=55%, signal=219%
SRF_Q6	11	-0.5177203	-1.0963489	0	0.42011988	0.993	1019	tags=100%, list=49%, signal=193%

TFIII_Q6	13	-0.4674017	-1.0930494	0	0.42815736	0.993	1125	tags=100%, list=54%, signal=214%
MIR7151_3P	12	-0.5031145	-1.0903428	0	0.43047476	0.994	1050	tags=100%, list=50%, signal=199%
MIR21_5P	11	-0.4746169	-1.0899092	0	0.42983097	0.994	1109	tags=100%, list=53%, signal=211%
MSX1_01	9	-0.4966507	-1.0750906	0	0.44842035	0.998	1062	tags=100%, list=51%, signal=202%
TCF11_01	13	-0.5680729	-1.0694946	0	0.4568752	0.998	915	tags=100%, list=44%, signal=176%
GATA_C	10	-0.5007181	-1.0668026	0	0.46071562	0.999	1054	tags=100%, list=50%, signal=200%
MIR6885_3P	11	-0.4746169	-1.0667384	0	0.4588825	0.999	1109	tags=100%, list=53%, signal=211%
MIR3692_3P	16	-0.4320692	-1.0638299	0	0.46228766	0.999	1200	tags=100%, list=57%, signal=232%
HNF3B_01	15	-0.4702495	-1.0571737	0	0.4696712	0.999	1120	tags=100%, list=53%, signal=213%
MIR3663_3P	11	-0.4626437	-1.0545851	0	0.46767226	0.999	1134	tags=100%, list=54%, signal=216%
MIR4310	14	-0.4752998	-1.0387841	0	0.47374707	0.999	1109	tags=100%, list=53%, signal=211%
MIR6881_3P	13	-0.5057526	-1.0373647	0	0.47489518	0.999	1045	tags=100%, list=50%, signal=198%
TGTTTAC_MIR30A5P_MIR30C_MIR30D_MIR30B_MIR30E5P	21	-0.4104909	-1.0166866	0	0.5026224	0.999	1247	tags=100%, list=59%, signal=244%
HALLMARK_IL6_JAK_STAT3_SIGNALING	12	-0.630091	-1.5219908	0	0.00306373	0.001	785	tags=100%, list=37%, signal=159%
HALLMARK_KRAS_SIGNALING_DN	12	-0.4983229	-1.2121212	0	0.0375817	0.018	1060	tags=100%, list=51%, signal=201%
BIOCARTA_EICOSANOID_PATHWAY	5	-0.6499522	-1.2354403	0.01754386	0.1162506	0.837	739	tags=100%, list=35%, signal=154%
WILENSKY_RESPONSE_TO_DARAPLADIB	5	-0.8170965	-1.541144	0.01785714	0.00845549	0.056	389	tags=100%, list=19%, signal=122%
WP_FGFR3_SIGNALING_IN_CHONDROCYTE_PROLIFERATION_AND_TERMINAL_DIFFERENTIATION	5	-0.7335243	-1.3419032	0.01785714	0.05940973	0.487	564	tags=100%, list=27%, signal=136%
WP_TGFBETA_SIGNALING_IN_THYROID_CELLS_FOR_EPITHELIALMESENCHYMAL_TRANSITION	5	-0.7960841	-1.4315505	0.01886793	0.02477206	0.204	433	tags=100%, list=21%, signal=126%
BEGUM_TARGETS_OF_PAX3_FOXO1_FUSION_DN	5	-0.7922636	-1.4578946	0.01923077	0.01938678	0.152	441	tags=100%, list=21%, signal=126%
LUI_THYROID_CANCER_CLUSTER_4	5	-0.7187201	-1.3912255	0.02	0.03853601	0.32	595	tags=100%, list=28%, signal=139%
LINDSTEDT_DENDRITIC_CELL_MATURATION_D	5	-0.7559694	-1.3929641	0.0212766	0.03748606	0.31	517	tags=100%, list=25%, signal=132%
CHIN_BREAST_CANCER_COPY_NUMBER_UP	5	-0.7053486	-1.3217127	0.0212766	0.0698464	0.569	623	tags=100%, list=30%, signal=142%
MIR6729_3P	5	-0.787488	-1.4140785	0.02173913	0.05285579	0.235	451	tags=100%, list=21%, signal=127%
REACTOME_AMYLOID_FIBER_FORMATION	5	-0.7048711	-1.2990099	0.02272727	0.07860899	0.639	624	tags=100%, list=30%, signal=142%
MAHADEVAN_GIST_MORPHOLOGICAL_SWITCH	5	-0.7502388	-1.4159383	0.02325581	0.03018886	0.245	529	tags=100%, list=25%, signal=133%

KEGG_DRUG_METABOLISM_OTHER_ENZYMES	6	-0.689441	-1.3651545	0.02325581	0.04797144	0.397	657	tags=100%, list=31%, signal=145%
REACTOME_REGULATION_OF_PLK1_ACTIVITY_AT_G2_M_TRANSITION	5	-0.7053486	-1.3448312	0.02325581	0.05807296	0.474	623	tags=100%, list=30%, signal=142%
REACTOME_MET_ACTIVATES_PTK2_SIGNALING	6	-0.6416627	-1.26417	0.02380952	0.09835097	0.763	757	tags=100%, list=36%, signal=156%
REACTOME_LAMININ_INTERACTIONS	6	-0.6908743	-1.3210305	0.025	0.07019532	0.571	654	tags=100%, list=31%, signal=145%
MIR1227_5P	5	-0.6981853	-1.2925441	0.02564103	0.16143057	0.624	638	tags=100%, list=30%, signal=143%
MIR1233_5P	6	-0.6220736	-1.1961564	0.02702703	0.29482657	0.905	798	tags=100%, list=38%, signal=161%
WP_KYNURENINE_PATHWAY_AND_LINKS_TO_CELL_SENESCENCE	6	-0.6927855	-1.3229927	0.02857143	0.06942838	0.564	650	tags=100%, list=31%, signal=144%
KANNAN_TP53_TARGETS_UP	6	-0.6282848	-1.2602175	0.02941177	0.1004469	0.772	785	tags=100%, list=37%, signal=159%
BERNARD_PPAPDC1B_TARGETS_DN	6	-0.6067845	-1.2420918	0.03125	0.11150289	0.824	830	tags=100%, list=40%, signal=165%
PID_HIF1_TFPATHWAY	6	-0.7032967	-1.3803883	0.03333334	0.04285219	0.356	628	tags=100%, list=30%, signal=142%
MIR6801_5P	6	-0.7224081	-1.3635545	0.03333334	0.08670382	0.378	588	tags=100%, list=28%, signal=139%
WACKER_HYPOXIA_TARGETS_OF_VHL	5	-0.69532	-1.307539	0.03389831	0.07813466	0.614	644	tags=100%, list=31%, signal=144%
SCHAEFFER_PROSTATE_DEVELOPMENT_12HR_DN	6	-0.6588629	-1.2997173	0.03448276	0.07850675	0.637	721	tags=100%, list=34%, signal=152%
MIR6778_5P	6	-0.6220736	-1.1985798	0.03571429	0.28978163	0.9	798	tags=100%, list=38%, signal=161%
REACTOME_APC_C_CDH1_MEDIATED_DEGRADATION_OF_CDC20_AND_OTHER_APC_C_CDH1_TARGETED_PROTEINS_IN_LATE_MITOSIS_EARLY_G1	5	-0.7053486	-1.3274994	0.03636364	0.06739695	0.548	623	tags=100%, list=30%, signal=142%
ONO_FOXP3_TARGETS_DN	5	-0.717765	-1.3108405	0.03703704	0.07638785	0.603	597	tags=100%, list=28%, signal=139%
MIR4329	6	-0.6411849	-1.2526447	0.03703704	0.21463268	0.761	758	tags=100%, list=36%, signal=156%
MIR2682_5P	6	-0.6507406	-1.2364334	0.03703704	0.24063547	0.801	738	tags=100%, list=35%, signal=154%
PID_SYNDECAN_4_PATHWAY	5	-0.7602674	-1.3913044	0.03846154	0.03860241	0.319	508	tags=100%, list=24%, signal=132%
CUI_GLUCOSE_DEPRIVATION	6	-0.6722408	-1.2743232	0.03846154	0.09200636	0.721	693	tags=100%, list=33%, signal=149%
WP_PATHOGENESIS_OF_SARSCOV2_MEDIATED_BY_NSP9NSP10_COMPLEX	5	-0.6728749	-1.2701396	0.03846154	0.09466144	0.736	691	tags=100%, list=33%, signal=149%
KYNG_WERNER_SYNDROM_AND_NORMAL_AGING_DN	5	-0.6614136	-1.2317427	0.03846154	0.11979508	0.846	715	tags=100%, list=34%, signal=151%
PID_DELTA_NP63_PATHWAY	6	-0.6688963	-1.3033925	0.04	0.07815883	0.624	700	tags=100%, list=33%, signal=150%
SHIPP_DLBCL_VS_FOLLICULAR_LYMPHOMA_UP	6	-0.627807	-1.2530994	0.04	0.10370286	0.791	786	tags=100%, list=37%, signal=159%
BIOCARTA_CELLCYCLE_PATHWAY	6	-0.6555184	-1.2341237	0.04	0.11767662	0.84	728	tags=100%, list=35%, signal=153%
MIR92A_1_5P	5	-0.6862464	-1.2772425	0.04	0.17750496	0.684	663	tags=100%, list=32%, signal=146%

WP_LUNG_FIBROSIS	6	-0.6927855	-1.3010806	0.0416667	0.0787357	0.635	650	tags=100%, list=31%, signal=144%
AMIT_EGF_RESPONSE_240_MCF10A	5	-0.6977077	-1.2924579	0.0425319	0.0823817 6	0.665	639	tags=100%, list=30%, signal=143%
MIR3161	5	-0.6494747	-1.2372252	0.0425319	0.2414184 4	0.799	740	tags=100%, list=35%, signal=154%
MIR601	5	-0.6938873	-1.2738569	0.0434782 6	0.1829004 1	0.699	647	tags=100%, list=31%, signal=144%
REACTOME_METABOLISM_OF_NUCLEOTIDES	7	-0.7251434	-1.4328524	0.0454545 5	0.0243662 5	0.199	583	tags=100%, list=28%, signal=138%
BERENJENO_ROCK_SIGNALING_NOT_VIA_RHOA_UP	5	-0.7363897	-1.3600837	0.0461538 5	0.0508206 5	0.422	558	tags=100%, list=27%, signal=136%
REACTOME_ER_TO_GOLGI_ANTEROGRADE_TRANSPORT	5	-0.7841452	-1.4176489	0.0465116 3	0.0298511 1	0.241	458	tags=100%, list=22%, signal=128%
BAE_BRCA1_TARGETS_UP	5	-0.7120344	-1.3047274	0.0465116 3	0.0780824 9	0.62	609	tags=100%, list=29%, signal=141%
MIR3929	6	-0.6980411	-1.3313403	0.0487804 9	0.1165373 3	0.482	639	tags=100%, list=30%, signal=143%

Supplementary Table 3: An examination of the differential expression (p<0.05 and |FC| >2) between the control HRTPT cells and those exposed to 4.5μM iAs for 3, 8, and 10 passages identified 247, 363, and 304 genes, respectively.

**Control vs P3**	**Control vs P8**	**Control vs P10**	**CntP3,CntP8,CntP10**
**247 Total Genes**	**363 Total Genes**	**304 Total Genes**	**167 Total Genes**
Down (106)	Down (118)	Down (111)	Down (76)
ACPP	ACPP	ACPP	ACPP
ACSL1	ACSL1	ACSL1	ACSL1
ACSM3	ACSM3	ADAMTSL3	ADAMTSL3
ADAMTSL3	ADAMTSL3	ARHGAP24	ARHGAP24
ARHGAP24	ARHGAP24	ARHGEF38	ARHGEF38
ARHGEF38	ARHGEF38	ATP6V1B1	ATP6V1B1
ATP1A1	ATP6V1B1	B3GALT5	B3GALT5
ATP1B1	B3GALT5	BCAM	BCAM
ATP6V1B1	BCAM	BIVM	BIVM
B3GALT5	BIVM	CCDC84	CCDC84
BCAM	CCDC84	CCNL2	CCNL2
BCKDHB	CCNL2	CDH16	CLCN5
BIVM	CDH16	CLCN5	CLDN16
CBS	CLCN5	CLDN16	CREB5
CCDC84	CLDN16	CPVL	CRNDE
CCNL2	CPVL	CREB5	CYFIP2
CLCN5	CREB5	CRIM1	DAB2
CLDN16	CRNDE	CRNDE	DMTF1
CREB5	CYFIP2	CYFIP2	EFHD1
CREG1	DAB2	DAB2	EMX2
CRNDE	DBI	DMTF1	EPB41L1
CYFIP2	DMTF1	EDIL3	ERBB4
DAB2	EDIL3	EFHD1	ESRRG
DEPTOR	EFHD1	EMP1	FABP3
DMTF1	EMX2	EMX2	FGF13
EFHD1	EPB41L1	EPB41L1	FGF9
EMX2	ERBB4	ERBB4	FGFR2
EPB41L1	ESRP1	ESRP1	FRMD4A
ERBB4	ESRRG	ESRRG	FYB
ESRRG	FABP3	FABP3	GPNMB
FABP3	FGF13	FGF13	GUSBP3
FGF13	FGF9	FGF9	HOXA6
FGF9	FGFR2	FGFR2	KCNJ1
FGFR2	FKSG51	FRMD4A	KCP
FRMD4A	FKSG56	FYB	LDOC1
FYB	FKSG59	GAS5	LENG8
GOT1	FKSG61	GPNMB	LIX1

GPNMB	FKSG63	GTF2IP20	LRP6
GUSBP3	FRMD4A	GUSBP2	MAN1A1
HOXA10	FYB	GUSBP3	MAOA
HOXA6	GLS	GUSBP9	MEAF6P1
HPSE	GPNMB	HERC2P9	NBPF1
IVNS1ABP	GTF2IP20	HOXA6	NEAT1
KCNJ1	GUSBP2	HOXB3	NPIPL3
KCP	GUSBP3	KCNJ1	NREP
LDOC1	GUSBP3	KCP	OGT
LENG8	GUSBP9	LDOC1	PDZK1
LIX1	HERC2P9	LENG8	PILRB
LRP6	HOXA6	LIX1	POU3F3
LUC7L3	IVNS1ABP	LRP6	PPARGC1A
MAN1A1	KCNJ1	LUC7L3	PTH1R
MAOA	KCP	MAN1A1	RAB17
MEAF6P1	LDOC1	MAOA	RAP1GAP
MECOM	LENG8	MEAF6P1	SCD5
METTL7A	LIX1	NBPF1	SEMA4A
MPC2	LRP6	NBPF12	SLC12A1
NBPF1	MAN1A1	NBPF14	SLC3A1
NEAT1	MAOA	NEAT1	SLC4A4
NPIPL3	MEAF6P1	NKTR	SLC6A13
NREP	MECOM	NMNAT2	SLCO4C1
OCLN	METTL7A	NPIPA5	SMA4
OGT	NBPF1	NPIPB11	SMG1P1
PDZK1	NBPF12	NPIPB3	SMG1P5
PILRB	NBPF14	NPIPB4	SNORD41
POU3F3	NEAT1	NPIPB5	SOSTDC1
PPARGC1A	NMNAT2	NPIPB6	SPTLC3
PROM1	NPIPA5	NPIPB8	SRSF5
PTH1R	NPIPB11	NPIPL3	STAG3L1
PTPN4	NPIPB3	NREP	STAG3L2
RAB17	NPIPB4	OGT	STAG3L3
RAP1GAP	NPIPB5	P3H2	TIA1
RBM47	NPIPB6	PAQR5	TMEM37
RBM5	NPIPB8	PDZK1	UGT8
SCD5	NPIPL3	PHLDA1	VAV3
SEMA4A	NREP	PILRB	VEPH1
SIAE	OGT	PKP4	WSB1
SIM2	P3H2	PMS2P3	
SLC12A1	PAQR5	POU3F3	Up (91)
SLC3A1	PDZK1	PPARGC1A	AKAP12
SLC4A4	PILRB	PTH1R	ARK1C1

SLC6A13	PKP4	QKI	AKR1C3
SLCO4C1	PMS2P3	QPCT	AMIGO2
SMA4	POU3F3	RAB17	C3
SMG1P1	PPARGC1A	RAP1GAP	CALB1
SMG1P5	PROM1	S100A2	CCDC146
SNORD13	PTH1R	SCD5	CCL20
SNORD24	PTPN4	SEMA4A	CD55
SNORD41	QPCT	SLC12A1	CDH6
SOSTDC1	RAB17	SLC3A1	CDKN1A
SPTLC3	RAP1GAP	SLC4A4	CEACAM1
SRSF5	RBM5	SLC6A13	CEACAM5
STAG3L1	S100A2	SLCO4C1	CFB
STAG3L2	SCD5	SMA4	CHST15
STAG3L3	SCIN	SMG1P1	CLEC4E
SUCLG1	SEMA4A	SMG1P2	CP
TCP11L2	SIAE	SMG1P5	CSF1
TIA1	SLC12A1	SNORD41	CTSE
TMEM171	SLC3A1	SOSTDC1	CXCL1
TMEM37	SLC4A4	SPTLC3	CXCL3
TMEM47	SLC6A13	SRSF5	CXCL8
UBA1	SLCO4C1	STAG3L1	DUSP1
UGT8	SMA4	STAG3L2	DUSP4
VAV3	SMG1P1	STAG3L3	DUSP5
VEGFA	SMG1P2	SYTL2	EPS8
VEPH1	SMG1P5	TIA1	ERRFI1
WSB1	SNORD41	TMEM171	F3
	SOSTDC1	TMEM37	FAM83D
Up (141)	SPTLC3	UGT8	FGF18
ACOT7	SRSF5	VAV3	FGFBP1
AKR1C1	STAG3L1	VEPH1	FHL2
AKAP12	STAG3L2	WSB1	GCNT3
AKR1C3	STAG3L3		GDA
ALAS1	SYTL2	Up (193)	GPRC5A
AMIGO2	TIA1	ACTB	HLA-DMA
C3	TMEM37	ADAM9	HMOX1
CALB1	UGT8	AKAP12	IFI6
CCDC146	VAV3	AKR1C1	IL18
CCL20	VEPH1	AKR1C3	IL32
CCND1	WSB1	AMIGO2	IL6
CCND3		ANLN	ITGB3
CD44	Up (245)	ARHGAP1	KLK10
CD55	ACOT7	ASRGL1	KRT18P24
CD68	ACTB	ATP2B4	LCN2

CDH6	ADAM9	BMP1	LGALS1
CDKN1A	AKAP12	C3	LPCAT1
CEACAM1	AKR1C1	CALB1	LYPD1
CEACAM5	AKR1C3	CALM1	MALL
CES3	ALAS1	CCDC146	MAP1B
CFB	AMIGO2	CCL20	MFAP5
CHST15	ANLN	CD55	MFGE8
CLEC4E	APOL1	CD68	MUC13
CLU	APOL2	CD74	MX1
CP	ASRGL1	CDH6	NNMT
CSF1	ATP2B4	CDKN1A	NRCAM
CTSE	BMP1	CEACAM1	PCDH1
CXCL1	C3	CEACAM5	PDZK1IP1
CXCL3	CALB1	CFB	PERP
CXCL8	CALM1	CHST15	PIK3AP1
DCBLD2	CCDC146	CKMT1A	PLEC
DUSP1	CCL20	CLDN1	PLK2
DUSP4	CCND1	CLDN10	PPBP
DUSP5	CD44	CLEC4E	PTHLH
EMP3	CD55	CMPK1	PTPRU
EPHA2	CD74	CNN2	PYGO1
EPS8	CDH6	CP	RARRES3
ERRFI1	CDKN1A	CSF1	RXFP1
F3	CEACAM1	CTSB	SAA1
FAM83D	CEACAM5	CTSE	SAA2
FGF18	CFB	CXCL1	SH2D1B
FGFBP1	CHST15	CXCL3	SH3BGRL2
FHL2	CKMT1A	CXCL8	SLC16A3
FTL	CLDN1	CYB5R3	SLC1A1
GCNT3	CLDN10	CYP4V2	SLC7A11
GDA	CLEC4E	DEGS1	SLC7A7
GPRC5A	CNN2	DSG2	SLC9A3R1
HLA-DMA	CP	DUSP1	SLPI
HMGA1	CSF1	DUSP4	TFPI2
HMOX1	CTSB	DUSP5	TGM2
HPGD	CTSE	EPHX1	TMOD1
IFI6	CXCL1	EPS8	TNFAIP3
IL18	CXCL3	ERO1A	TRIM16L
IL32	CXCL8	ERRFI1	TRIM22
IL6	CYB5R3	ETHE1	TSTA3
ITGA3	DCBLD2	F3	TTC9
ITGB3	DEGS1	FAM83D	TXNRD1
KDELR3	DNAJB1	FGF18	UCA1

KLK10	DSG2	FGFBP1	UGT1A1
KRT18	DUSP1	FHL2	WNT7A
KRT18P24	DUSP4	GABARAPL1	ZNF486
LAMA3	DUSP5	GBP2	
LCN2	EPHA2	GCNT3	
LGALS1	EPHX1	GDA	
LPCAT1	EPS8	GPR143	
LRRC8A	ERO1A	GPRC5A	
LRRN4	ERRFI1	GPX3	
LYPD1	ETHE1	GSR	
MALL	F3	H2BFS	
MAP1B	FAM129B	HABP2	
MFAP5	FAM83D	HIST1H1B	
MFGE8	FGF18	HIST1H2AI	
MUC13	FGFBP1	HIST1H2BK	
MX1	FHL2	HIST2H2AA3	
NNMT	FSCN1	HIST2H2AA4	
NRCAM	GABARAPL1	HIST2H2BB	
NT5E	GBP2	HIST2H2BF	
PCDH1	GCNT3	HLA-DMA	
PDZK1IP1	GDA	HLA-DRA	
PERP	GPR143	HLA-DRB1	
PIK3AP1	GPRC5A	HMOX1	
PLEC	H2AFZ	HN1	
PLK2	H2BFS	IFI6	
PPBP	HIST1H1B	IGFBP3	
PTHLH	HIST1H2AI	IL18	
PTPRU	HIST1H2BK	IL1R1	
PYGO1	HIST2H2AA3	IL32	
RARRES3	HIST2H2AA4	IL6	
RNA5S1	HIST2H2BB	ITGB3	
RNA5S10	HIST2H2BF	KLK10	
RNA5S11	HLA-DMA	KPNA2	
RNA5S12	HLA-DRA	KRT18P24	
RNA5S13	HLA-DRB1	LAMB3	
RNA5S14	HM13	LCN2	
RNA5S15	HMOX1	LGALS1	
RNA5S16	HN1	LIPH	
RNA5S17	HPGD	LPCAT1	
RNA5S2	ICAM1	LRRC59	
RNA5S3	IFI6	LYPD1	
RNA5S4	IGFBP3	MALL	
RNA5S6	IL18	MAP1B	

RNA5S7	IL1R1	MAP1LC3B
RNA5S9	IL32	MFAP5
RUNX2	IL6	MFGE8
RXFP1	ITGA3	MFHAS1
S100A16	ITGB3	MGAT5
SAA1	KDELR3	MICAL2
SAA2	KLK10	MTRNR2L9
SAR1A	KPNA2	MTUS1
SCEL	KRT18	MUC13
SH2D1B	KRT18P24	MX1
SH3BGRL2	LAMA3	MYADM
SLC16A3	LAMB3	NEU1
SLC1A1	LAMC2	NFKBIA
SLC7A11	LCN2	NNMT
SLC7A7	LGALS1	NRCAM
SLC9A3R1	LIPH	ODC1
SLPI	LPCAT1	PCDH1
SPON1	LRRC59	PDP1
SQRDL	LRRC8A	PDZD2
SRM	LRRN4	PDZK1IP1
STEAP3	LYPD1	PERP
TCEB1	MALL	PIK3AP1
TFPI2	MAP1B	PLEC
TGFBR2	MFAP5	PLK1
TGM2	MFGE8	PLK2
TM4SF4	MFHAS1	PLLP
TMEM184B	MGAT5	PLPP2
TMOD1	MICAL2	PML
TNFAIP3	MMP14	POR
TNFRSF21	MTUS1	PORCN
TRIM16L	MUC13	PPBP
TRIM22	MX1	PPP1R3B
TSTA3	MYADM	PRKAR1A
TTC9	NET1	PRSS8
TXNRD1	NFKBIA	PSAT1
U2	NNMT	PTHLH
UCA1	NRCAM	PTPRU
UGT1A1	NT5E	PYGO1
WNT7A	ODC1	QSOX1
ZNF486	PCDH1	RARRES3
	PDLIM1	RDH10
	PDP1	RIMKLB
	PDZD2	RIPK2

	PDZK1IP1	RNF144B
	PERP	RXFP1
	PIK3AP1	SAA1
	PLEC	SAA2
	PLK1	SAT1
	PLK2	SERPINB1
	PLLP	SH2D1B
	PLPP2	SH3BGRL2
	PML	SHROOM3
	POR	SIK2
	PORCN	SLC15A1
	PPBP	SLC16A3
	PPP1R3B	SLC1A1
	PRKAG2	SLC20A2
	PRNP	SLC2A1
	PRSS8	SLC37A1
	PSAT1	SLC4A11
	PTHLH	SLC6A20
	PTPRU	SLC7A11
	PVRL2	SLC7A7
	PYGO1	SLC9A3R1
	QSOX1	SLPI
	RARRES3	SNX2
	RHOC	SOD2
	RHOF	SRD5A3
	RIMKLB	ST6GALNAC1
	RIPK2	SULT1A4
	RNA5S1	TFPI2
	RNA5S10	TGM2
	RNA5S11	TINAGL1
	RNA5S17	TM2D2
	RNA5S2	TM4SF4
	RNA5S3	TMOD1
	RNA5S4	TNFAIP3
	RNA5S6	TNFSF10
	RNA5S7	TPCN1
	RNF144B	TPK1
	RUNX2	TPM4
	RXFP1	TRIM16L
	S100A16	TRIM22
	SAA1	TSKU
	SAA2	TSPAN14
	SCEL	TSTA3

	SERPINB1	TTC9
	SFN	TUBB3
	SH2D1B	TXNDC12
	SH3BGRL2	TXNRD1
	SH3BGRL3	UCA1
	SHROOM3	UCP2
	SIK2	UGT1A1
	SLC15A1	UNC93B1
	SLC16A3	WNT7A
	SLC1A1	XBP1
	SLC20A2	ZNF486
	SLC2A1	
	SLC37A1	
	SLC39A6	
	SLC4A11	
	SLC6A20	
	SLC7A11	
	SLC7A7	
	SLC9A3R1	
	SLPI	
	SLX1A	
	SOD2	
	SORBS2	
	SPON1	
	SQRDL	
	SRD5A3	
	SRM	
	ST6GALNAC1	
	STEAP3	
	SULT1A4	
	TAGLN2	
	TALDO1	
	TCEB1	
	TFPI2	
	TGFBR2	
	TGM2	
	THBS1	
	TINAGL1	
	TM2D2	
	TMEM184B	
	TMEM63B	
	TMOD1	
	TNFAIP3	

	TNFRSF21
	TNFSF10
	TPCN1
	TPK1
	TPM4
	TPRA1
	TRAM2
	TRIM16L
	TRIM22
	TSKU
	TSPAN14
	TSTA3
	TTC9
	TUBA1C
	TUBB3
	TUBB4B
	TXN
	TXNDC12
	TXNRD1
	UCA1
	UCP2
	UGT1A1
	UNC93B1
	WARS
	WNT7A
	XBP1
	ZDHHC9
	ZNF486

Supplementary Table 4: Differential gene expression analysis between all HRTPT cells exposed to iAs passage (i.e., combined P3, P8, P10) and control.

probe	FC_iAS+VSctrl	log2(FC)	raw.pval	-LOG(p)	Gene Symbol
TC1500010739.hg.1	0.0064075	-7.286	8.02E-15	14.096	SLC12A1
TC1100012786.hg.1	0.0215	-5.5395	1.21E-14	13.918	KCNJ1
TC0800007715.hg.1	0.037656	-4.731	4.20E-13	12.377	
TC0300007273.hg.1	0.16649	-2.5865	9.25E-13	12.034	PTH1R
TC0100017310.hg.1	0.12226	-3.032	2.93E-12	11.534	ESRRG
TC0400011180.hg.1	0.11507	-3.1194	8.33E-12	11.08	SCD5
TC1400007562.hg.1	0.47681	-1.0685	8.38E-12	11.077	SRSF5
TC0300009853.hg.1	0.080953	-3.6268	1.10E-10	9.9573	CLDN16
TC0100015586.hg.1	0.37288	-1.4232	2.25E-10	9.6481	PDZK1
TC0700013623.hg.1	0.19783	-2.3377	2.78E-10	9.5565	KCP
TC1900008860.hg.1	0.36077	-1.4708	8.17E-10	9.0879	LENG8
TC0100015155.hg.1	0.22889	-2.1272	1.07E-09	8.9697	VAV3
TC1900008286.hg.1	0.28588	-1.8065	1.59E-09	8.798	BCAM
TC0400012829.hg.1	0.35883	-1.4786	2.01E-09	8.6962	ARHGEF38
TC0700013575.hg.1	0.37298	-1.4228	2.39E-09	8.6222	STAG3L3
TC1300008095.hg.1	0.31838	-1.6512	4.02E-09	8.3957	
TC1500010618.hg.1	0.22758	-2.1356	4.12E-09	8.3855	
TC0700013427.hg.1	0.21249	-2.2345	5.02E-09	8.2993	STAG3L5P-PVRIG2P-PILRB
TC1600010375.hg.1	0.39119	-1.3541	5.99E-09	8.2225	CRNDE
TC1600010602.hg.1	0.48616	-1.0405	7.41E-09	8.1301	CDH16
TC1200008675.hg.1	2.8042	1.4876	9.39E-09	8.0272	TXNRD1
TC0100010155.hg.1	0.14946	-2.7422	1.30E-08	7.8871	SEMA4A
TC0700013428.hg.1	0.25301	-1.9827	1.47E-08	7.8336	STAG3L5P
TC0700011519.hg.1	0.39518	-1.3394	1.47E-08	7.8319	STAG3L2
TC0500011528.hg.1	0.1398	-2.8386	1.64E-08	7.785	LIX1
TC0X00010989.hg.1	0.34424	-1.5385	1.94E-08	7.713	LDOC1
TC0700013388.hg.1	0.37325	-1.4218	2.13E-08	7.6707	STAG3L1
TC0400012621.hg.1	0.35525	-1.4931	2.86E-08	7.5437	ACSL1
TC0200013750.hg.1	2.6575	1.41	3.10E-08	7.5084	FHL2
TC0800010002.hg.1	3.2173	1.6858	3.37E-08	7.4718	DUSP4
TC1700010994.hg.1	0.27383	-1.8687	3.57E-08	7.4469	
TC2000009216.hg.1	5.897	2.56	4.25E-08	7.3711	SLPI
TC0600013326.hg.1	2.6285	1.3943	4.37E-08	7.3597	PERP
TC0300006925.hg.1	2.0059	1.0042	5.44E-08	7.2641	TGFBR2
HTA2.pos.2985914_st	0.22603	-2.1454	5.53E-08	7.2572	
TC0100011406.hg.1	2.6491	1.4055	5.78E-08	7.2383	CD55
TC1300008096.hg.1	0.27946	-1.8393	8.59E-08	7.0659	

TC0700010348.hg.1	0.20176	-2.3093	8.86E-08	7.0528	SOSTDC1
TC0100013445.hg.1	5.1539	2.3657	1.08E-07	6.9678	IFI6
TC0600007847.hg.1	2.739	1.4536	1.13E-07	6.9453	CDKN1A
TC2100006465.hg.1	0.31492	-1.667	1.29E-07	6.8892	
TC2100006481.hg.1	0.31492	-1.667	1.29E-07	6.8892	
TC22_KI270733v1_random00006438.hg.1	0.31492	-1.667	1.29E-07	6.8892	
TCUn_GL000220v100006438.hg.1	0.31492	-1.667	1.29E-07	6.8892	
TC1200007626.hg.1	0.42933	-1.2198	1.42E-07	6.8476	METTL7A
TC1200012859.hg.1	2.0255	1.0183	1.56E-07	6.8072	RHOF
TC1700007319.hg.1	0.3556	-1.4917	2.27E-07	6.6448	WSB1
TC1200009547.hg.1	0.3495	-1.5166	2.52E-07	6.5991	SLC6A13
TC1300008013.hg.1	0.34007	-1.5561	2.86E-07	6.5431	
TC0200011107.hg.1	0.3777	-1.4047	3.98E-07	6.3996	EFHD1
TC2000006736.hg.1	0.33479	-1.5787	4.25E-07	6.3712	SPTLC3
TC1000009025.hg.1	0.16796	-2.5738	4.44E-07	6.3525	EMX2
TC1200009834.hg.1	82.547	6.3671	4.52E-07	6.3446	MFAP5
TC0500013317.hg.1	0.32979	-1.6004	5.91E-07	6.2283	SMA4
TC0700006913.hg.1	0.12385	-3.0133	6.17E-07	6.21	GPNMB
TC1600011514.hg.1	0.42225	-1.2438	6.19E-07	6.2082	SMG1P2
TC0100016694.hg.1	0.47391	-1.0773	6.35E-07	6.1972	IVNS1ABP
TC1200008933.hg.1	2.1813	1.1252	7.14E-07	6.1461	TPCN1
TC0200013926.hg.1	0.41792	-1.2587	8.26E-07	6.0831	
TC0200009067.hg.1	0.26933	-1.8925	9.21E-07	6.0356	TMEM37
TC1100011185.hg.1	0.24793	-2.012	1.05E-06	5.9794	
TC1700012274.hg.1	2.8643	1.5182	1.06E-06	5.9762	ITGB3
TC0400010242.hg.1	0.3018	-1.7283	1.13E-06	5.9475	PPARGC1A
TC0500011596.hg.1	0.35447	-1.4962	1.18E-06	5.9276	SLCO4C1
TC0700006468.hg.1	0.32396	-1.6261	1.25E-06	5.9018	
TC0400011920.hg.1	12.813	3.6795	1.38E-06	5.86	SLC7A11
TC0500009768.hg.1	0.43435	-1.2031	1.42E-06	5.8468	
TC0600009862.hg.1	2.5179	1.3322	1.76E-06	5.7536	AKAP12
TC1500007409.hg.1	25.34	4.6633	1.83E-06	5.7378	GCNT3
TC0200009756.hg.1	0.45036	-1.1508	1.88E-06	5.7254	PKP4
TC1600007205.hg.1	0.36034	-1.4726	2.25E-06	5.6469	
TC0600011173.hg.1	0.43202	-1.2108	2.47E-06	5.6068	GUSBP2
TC1700008867.hg.1	2.641	1.4011	2.92E-06	5.5347	SLC9A3R1
TC1900007242.hg.1	16.052	4.0047	3.38E-06	5.4714	UCA1
TC1000012428.hg.1	2.3306	1.2207	3.82E-06	5.4182	AKR1C3
TC0500010835.hg.1	2.8982	1.5352	3.85E-06	5.4141	PLK2
TC0500013318.hg.1	0.41666	-1.2631	4.00E-06	5.3977	GUSBP3
TC2100006467.hg.1	0.31181	-1.6813	4.36E-06	5.36	

TC22_KI270733v1_random00006440.hg.1	0.31181	-1.6813	4.36E-06	5.36	
TC1100013118.hg.1	0.38537	-1.3757	4.58E-06	5.3394	LINC01001
TC0300013417.hg.1	2.0904	1.0638	5.13E-06	5.2897	LIPH
TC0200015616.hg.1	0.29676	-1.7526	6.29E-06	5.201	ERBB4
TC1100008018.hg.1	0.26538	-1.9139	6.78E-06	5.1685	NEAT1
TC0800012147.hg.1	2.6226	1.391	7.16E-06	5.1452	TSTA3
TC1100008236.hg.1	2.3218	1.2152	8.79E-06	5.0561	CCND1
TC0600010357.hg.1	0.44927	-1.1544	8.96E-06	5.0478	
TC0200007418.hg.1	0.29073	-1.7823	9.21E-06	5.0356	SLC3A1
TC1600011364.hg.1	0.45411	-1.1389	9.67E-06	5.0145	NPIPB5
TC0500012842.hg.1	3.6126	1.853	1.01E-05	4.9976	DUSP1
TC1200006896.hg.1	2.6934	1.4294	1.02E-05	4.991	GPRC5A
TC0300010086.hg.1	0.46327	-1.1101	1.13E-05	4.9463	
TC0100017456.hg.1	0.46728	-1.0976	1.16E-05	4.9347	GTF2IP20
TC0300009139.hg.1	2.3578	1.2374	1.40E-05	4.8553	TM4SF4
TC1000009522.hg.1	0.44954	-1.1535	1.40E-05	4.8541	
TC0500007761.hg.1	0.40569	-1.3016	1.57E-05	4.8028	TMEM171
TC0300010350.hg.1	2.5832	1.3692	1.80E-05	4.7457	WNT7A
TC0800010823.hg.1	2.0679	1.0482	1.99E-05	4.7008	TCEB1
TSUnmapped00000976.hg.1	16.059	4.0053	2.05E-05	4.688	
TC0700008182.hg.1	0.37659	-1.4089	2.43E-05	4.6147	
TC1500007833.hg.1	2.0934	1.0658	2.47E-05	4.6065	PML
TC0700010604.hg.1	0.40715	-1.2964	2.57E-05	4.5904	CPVL
TC0900006522.hg.1	2.4022	1.2644	2.61E-05	4.5829	SLC1A1
TC0200010630.hg.1	0.45002	-1.1519	2.73E-05	4.5642	
TC0300013513.hg.1	0.46821	-1.0948	2.91E-05	4.5363	P3H2
TC1600009958.hg.1	0.45609	-1.1326	2.98E-05	4.5264	LOC613037
TC0500009424.hg.1	4.6363	2.213	3.30E-05	4.4812	FGF18
TC1600011501.hg.1	0.44623	-1.1641	3.53E-05	4.4517	NPIPB3
TC0500011705.hg.1	0.21187	-2.2387	3.76E-05	4.4244	NREP
TC0100018403.hg.1	2.331	1.221	4.38E-05	4.3582	ERRFI1
TC0200013861.hg.1	7.6862	2.9423	4.41E-05	4.356	MALL
TC1700011097.hg.1	2.365	1.2418	4.60E-05	4.3375	LRRC59
TC0100018463.hg.1	2.0041	1.0029	5.76E-05	4.2396	RHOC
TC0800012176.hg.1	2.3328	1.2221	6.58E-05	4.1818	PLEC
TC0600011960.hg.1	2.0542	1.0386	7.93E-05	4.1005	TNFRSF21
TC0900010506.hg.1	2.3295	1.22	7.94E-05	4.1001	KRT18P24
TC1100006911.hg.1	2.196	1.1349	9.74E-05	4.0116	SPON1
TC1600007037.hg.1	0.45641	-1.1316	0.00011186	3.9513	NPIPA7
TC1600011505.hg.1	0.46716	-1.098	0.00013025	3.8852	NPIPB4
TC1100007899.hg.1	4.3076	2.1069	0.0001307	3.8837	RARRES3

TC0100018480.hg.1	0.47472	-1.0749	0.00014001	3.8538	NBPF14
TC0700008624.hg.1	0.46978	-1.0899	0.0001411	3.8505	
TC0500009863.hg.1	2.7543	1.4617	0.00014395	3.8418	LPCAT1
TC1700007135.hg.1	2.3486	1.2318	0.00016314	3.7874	TRIM16L
TC2100007316.hg.1	2.294	1.1979	0.00016469	3.7833	
TC0800007888.hg.1	5.6227	2.4913	0.00017145	3.7659	C8orf34
TC0600013684.hg.1	0.45473	-1.1369	0.0001811	3.7421	
TC0300013068.hg.1	0.47352	-1.0785	0.00019033	3.7205	MECOM
TC1000012117.hg.1	2.6462	1.4039	0.00019805	3.7032	CHST15
TC2000009058.hg.1	20.6	4.3646	0.000216	3.6655	TGM2
TC1100012318.hg.1	2.7145	1.4407	0.00023063	3.6371	IL18
TC1600009916.hg.1	0.4768	-1.0685	0.00023584	3.6274	NPIPB11
TC2100007208.hg.1	4.4369	2.1495	0.00023615	3.6268	MX1
TC1600009929.hg.1	0.45401	-1.1392	0.00024134	3.6174	
TC0700012230.hg.1	5.0991	2.3502	0.00026945	3.5695	NRCAM
TC0200011138.hg.1	16.964	4.0844	0.00028668	3.5426	UGT1A8
TC0100018478.hg.1	0.4572	-1.1291	0.00030028	3.5225	NBPF10
TC0100014081.hg.1	4.2223	2.078	0.0003083	3.511	PDZK1IP1
TC1200007686.hg.1	2.0359	1.0256	0.0003768	3.4239	KRT18
TC1100011833.hg.1	0.4898	-1.0297	0.00042113	3.3756	SYTL2
TC0300013520.hg.1	2.0367	1.0263	0.00043462	3.3619	CLDN1
TC0700006890.hg.1	12.643	3.6603	0.00044084	3.3557	IL6
TC0700012939.hg.1	0.48302	-1.0498	0.00045549	3.3415	
TC2100007485.hg.1	2.0813	1.0575	0.00048804	3.3115	
TC0100011267.hg.1	2.2419	1.1647	0.00051146	3.2912	ATP2B4
TC0800008263.hg.1	0.19375	-2.3677	0.00051449	3.2886	ESRP1
TC1500010369.hg.1	3.5502	1.8279	0.00051559	3.2877	MFGE8
TC0800009961.hg.1	2.1063	1.0747	0.00053081	3.2751	CLU
TC0100015871.hg.1	2.1078	1.0757	0.0005713	3.2431	S100A16
TC0900008891.hg.1	2.7627	1.4661	0.0006099	3.2147	LRRC8A
TC0600008146.hg.1	2.4865	1.3141	0.00068409	3.1649	RUNX2
TC0100009364.hg.1	6.4234	2.6833	0.00073715	3.1324	CSF1
TC1800007655.hg.1	2.3289	1.2197	0.00074711	3.1266	LOC100505817
TC1700008228.hg.1	2.1864	1.1286	0.00076112	3.1185	ITGA3
TC0600011185.hg.1	2.4208	1.2755	0.00081624	3.0882	HIST1H2BK
TC1500010700.hg.1	0.49015	-1.0287	0.00083058	3.0806	SNHG14
TC0600014217.hg.1	2.1942	1.1337	0.00091087	3.0405	SERPINB1
TC2200007356.hg.1	2.659	1.4109	0.00095398	3.0205	KDELR3
TC0100007584.hg.1	3.1258	1.6442	0.00095668	3.0192	PTPRU
TC1200010038.hg.1	2.6995	1.4327	0.0010155	2.9933	EPS8
TC0500007738.hg.1	17.418	4.1225	0.001119	2.9512	MAP1B

TC1800006897.hg.1	2.725	1.4462	0.001154	2.9378	LAMA3
TC1100011602.hg.1	2.3833	1.253	0.0011963	2.9222	UCP2
TC1000012427.hg.1	2.0005	1.0004	0.0012699	2.8962	AKR1C1
TC0600008622.hg.1	3.1417	1.6515	0.0013071	2.8837	SH3BGRL2
TC0100016206.hg.1	22.378	4.484	0.0013343	2.8748	SH2D1B
TC2200007204.hg.1	15.894	3.9904	0.0013361	2.8742	HMOX1
TC0400010125.hg.1	3.33	1.7355	0.0013585	2.8669	FGFBP1
TC0600009597.hg.1	4.6159	2.2066	0.0014889	2.8271	TNFAIP3
TC1900009095.hg.1	2.1634	1.1133	0.0015276	2.816	
TC0300012768.hg.1	34.482	5.1078	0.00154	2.8125	CP
TC1400008940.hg.1	2.2862	1.193	0.0017091	2.7672	NFKBIA
TC0300011815.hg.1	2.0927	1.0653	0.0017695	2.7521	DCBLD2
TC0800008243.hg.1	2.7481	1.4584	0.0017895	2.7473	PDP1
TC1200009829.hg.1	17.605	4.1379	0.0020188	2.6949	CLEC4E
TC1800007014.hg.1	2.0704	1.0499	0.0020979	2.6782	DSG2
TC1600009855.hg.1	0.49922	-1.0022	0.0021484	2.6679	NPIPB6
TC0400011015.hg.1	3.5044	1.8092	0.0021656	2.6644	CXCL3
TC0100013908.hg.1	2.0862	1.0609	0.0021756	2.6624	SLC2A1
TC1200010743.hg.1	4.9828	2.317	0.0022297	2.6518	
TC0100012675.hg.1	2.1983	1.1364	0.002294	2.6394	ACOT7
TC1200009739.hg.1	3.0936	1.6293	0.0023011	2.6381	
TC0400007836.hg.1	14.879	3.8952	0.0025503	2.5934	CXCL8
TC1100011375.hg.1	3.1456	1.6534	0.0026819	2.5716	C11orf24
TC0500007044.hg.1	4.1104	2.0393	0.0027779	2.5563	CDH6
TC1000010567.hg.1	0.4385	-1.1894	0.002796	2.5535	
TC0100017167.hg.1	2.7426	1.4555	0.0028746	2.5414	LAMB3
TC1000011186.hg.1	0.47259	-1.0813	0.0029134	2.5356	
TC2100008527.hg.1	2.0158	1.0114	0.0029539	2.5296	SLC37A1
TC0400007901.hg.1	3.2891	1.7177	0.0030019	2.5226	SHROOM3
TC1600006658.hg.1	6.9778	2.8028	0.0034162	2.4665	IL32
TC1400006659.hg.1	2.073	1.0517	0.0037051	2.4312	MMP14
TC0200009402.hg.1	2.557	1.3545	0.0040009	2.3978	MGAT5
TC1200010229.hg.1	6.34	2.6645	0.0044017	2.3564	PTHLH
TC0400007840.hg.1	3.4314	1.7788	0.0044375	2.3529	CXCL1
TC0800009287.hg.1	3.9358	1.9766	0.0046486	2.3327	
TC1100007273.hg.1	2.6919	1.4287	0.0047299	2.3252	CD44
TC0800009523.hg.1	2.1346	1.094	0.0049644	2.3041	MFHAS1
TC0400009129.hg.1	6.9784	2.8029	0.0050385	2.2977	RXFP1
TC0100010798.hg.1	3.3454	1.7422	0.0050545	2.2963	QSOX1
TC0300013146.hg.1	2.2661	1.1802	0.0051757	2.286	TNFSF10
TC0100007645.hg.1	2.4391	1.2864	0.0053887	2.2685	TINAGL1

TC0100018451.hg.1	3.0596	1.6133	0.0054078	2.267	GBP2
TC1200009297.hg.1	4.9212	2.299	0.0060305	2.2196	
TC1900010851.hg.1	2.0917	1.0647	0.0062866	2.2016	ETHE1
TC1300007561.hg.1	2.9978	1.5839	0.0064453	2.1908	SCEL
TC0600011938.hg.1	9.2472	3.209	0.0069224	2.1597	CLIC5
TC0900012047.hg.1	2.9186	1.5453	0.0072154	2.1417	
TC1900007465.hg.1	3.9411	1.9786	0.007801	2.1078	ZNF486
TC0700013394.hg.1	7.0241	2.8123	0.0079167	2.1015	CCDC146
TC1100008514.hg.1	2.0995	1.0701	0.0080694	2.0932	TSKU
TC2000008217.hg.1	2.222	1.1518	0.0081731	2.0876	SLC4A11
TC1400009186.hg.1	2.5892	1.3725	0.0082005	2.0862	ERO1A
TC0X00007190.hg.1	2.6144	1.3865	0.0082021	2.0861	PORCN
TC1900009443.hg.1	86.534	6.4352	0.0084834	2.0714	C3
TC0600007374.hg.1	2.5117	1.3287	0.0087172	2.0596	HIST1H2AI
TC1100011259.hg.1	3.7567	1.9095	0.0091786	2.0372	FOSL1
TC0100018482.hg.1	2.0263	1.0189	0.0094234	2.0258	HIST2H2AA3
TC0600014277.hg.1	5.4962	2.4584	0.0096522	2.0154	HLA-DMA
TC1600011453.hg.1	2.336	1.224	0.009814	2.0082	MC1R
TC1100013152.hg.1	14.257	3.8336	0.010179	1.9923	SAA2-SAA4
TC0900007667.hg.1	2.0836	1.0591	0.011182	1.9515	PSAT1
TC0800006975.hg.1	2.9564	1.5638	0.011475	1.9402	BMP1
TC0700010167.hg.1	2.0514	1.0366	0.011774	1.9291	ACTB
TC1600011546.hg.1	3.4303	1.7784	0.012041	1.9194	PLLP
TC1900009076.hg.1	2.6474	1.4046	0.013578	1.8672	PLPP2
TC1100006995.hg.1	16.157	4.0141	0.013658	1.8646	SAA1
TC2200007312.hg.1	4.0824	2.0294	0.014328	1.8438	LGALS1
TC1400007950.hg.1	2.092	1.0649	0.01479	1.83	CALM1
TC2200008370.hg.1	2.4207	1.2754	0.016148	1.7919	XBP1
TC1100012825.hg.1	4.2145	2.0754	0.016956	1.7707	
TC0700012917.hg.1	2.216	1.1479	0.017072	1.7677	TPK1
TC1500007695.hg.1	0.47601	-1.0709	0.017093	1.7672	PAQR5
TC1500009504.hg.1	5.8762	2.5549	0.017118	1.7666	PYGO1
TC0100014988.hg.1	15.17	3.9231	0.017381	1.7599	F3
TC0800009529.hg.1	2.5689	1.3612	0.01792	1.7467	PPP1R3B
TC1600008609.hg.1	2.7276	1.4476	0.018908	1.7234	HSD17B2
TC0600013757.hg.1	3.2075	1.6814	0.019713	1.7052	SOD2
TC1100012966.hg.1	2.863	1.5175	0.020106	1.6967	MICAL2
TC0600011232.hg.1	3.4485	1.786	0.02083	1.6813	HIST1H1B
TC1100012513.hg.1	3.0342	1.6013	0.021235	1.6729	H2AFX
TC0400009716.hg.1	2.1155	1.081	0.023857	1.6224	
TC2000007341.hg.1	4.4346	2.1488	0.024612	1.6088	FAM83D

TC1700008719.hg.1	2.1701	1.1178	0.025668	1.5906	KPNA2
TC1900011741.hg.1	18.554	4.2136	0.025677	1.5904	CEACAM5
TC0300012236.hg.1	48.703	5.6059	0.025862	1.5873	MUC13
TC1700011813.hg.1	4.2377	2.0833	0.025996	1.5851	ST6GALNAC1
TC0700010965.hg.1	4.7822	2.2577	0.02635	1.5792	IGFBP3
TC1400008677.hg.1	9.4859	3.2458	0.026612	1.5749	SLC7A7
TC0700008073.hg.1	2.5172	1.3318	0.026901	1.5702	POR
TC0900008847.hg.1	92.409	6.53	0.027097	1.5671	LCN2
TC0200008666.hg.1	7.542	2.9149	0.027286	1.5641	IL1R1
TC0400007556.hg.1	2.0711	1.0504	0.027352	1.563	SRD5A3
TC1900011382.hg.1	3.6654	1.874	0.028514	1.5449	
TC1200011470.hg.1	2.979	1.5748	0.029339	1.5326	DUSP6
TC1500007062.hg.1	5.3541	2.4206	0.029833	1.5253	CKMT1B
TC1500007067.hg.1	5.1968	2.3776	0.030109	1.5213	CKMT1A
TC0700007623.hg.1	2.3584	1.2378	0.032296	1.4908	
TC0100017084.hg.1	29.489	4.8821	0.032683	1.4857	CTSE
TC1600007537.hg.1	2.1939	1.1335	0.034092	1.4674	TGFB1I1
TC0300010930.hg.1	6.8064	2.7669	0.035989	1.4438	SLC6A20
TC0400009258.hg.1	3.3333	1.737	0.036151	1.4419	PALLD
TC0100015710.hg.1	2.8042	1.4876	0.03748	1.4262	
TC0900007552.hg.1	11.162	3.4805	0.038518	1.4143	GDA
TC0100015705.hg.1	2.4274	1.2794	0.039345	1.4051	
TC0600014106.hg.1	19.528	4.2875	0.040573	1.3918	CFB
TC0100015706.hg.1	2.8229	1.4972	0.042125	1.3755	
TC0500012470.hg.1	17.915	4.1631	0.045449	1.3425	CD74
TC1200006730.hg.1	2.0147	1.0106	0.046541	1.3322	RIMKLB
TC2100006834.hg.1	66.084	6.0462	0.048846	1.3112	LOC284825
TC0600014273.hg.1	18.562	4.2142	0.049117	1.3088	HLA-DRB1
TC1900006814.hg.1	30.376	4.9249	0.049738	1.3033	
TC1300009580.hg.1	8.2626	3.0466	0.052395	1.2807	SLC15A1
TC0600007650.hg.1	56.592	5.8225	0.056004	1.2518	HLA-DRA
TC1000007641.hg.1	6.6423	2.7317	0.061153	1.2136	DKK1
TC0200010980.hg.1	113.25	6.8234	0.064347	1.1915	CCL20
TC0300008123.hg.1	7.3328	2.8744	0.071996	1.1427	LINC00973
TC1900011742.hg.1	11.56	3.5311	0.072875	1.1374	CEACAM6
TC0100011402.hg.1	22.861	4.5148	0.089003	1.0506	C4BPA
TC0800011064.hg.1	17.268	4.11	0.089226	1.0495	CALB1
TC0400011013.hg.1	61.367	5.9394	0.098596	1.0061	PPBP

Supplementary Table 5A: Upregulated Gene Set Enrichment Analysis for 167 gene set with nominal p-value < 0.05.

GS	Size	ES	NES	NOM p-val	FDR q-val	FWER p-val	Rank at Max	Leading Edge
OCT1_02	5	0.88	2.12	0	0.231	0.156	24	tags=100%, list=14%, signal=113%
YTATTTTNR_MEF2_02	12	0.59	2.05	0.002	0.223	0.271	24	tags=50%, list=14%, signal=54%
WGGAATGY_TEF1_Q6	10	0.65	2.02	0.002	0.192	0.338	33	tags=70%, list=20%, signal=82%
CTATGCA_MIR153	6	0.75	1.97	0.002	0.226	0.476	29	tags=83%, list=17%, signal=97%
TAATAAT_MIR126	5	0.79	1.92	0.002	0.266	0.621	38	tags=100%, list=23%, signal=126%
SOX5_01	6	0.72	1.87	0.002	0.252	0.781	20	tags=67%, list=12%, signal=73%
BLANCO_MELO_RESPIRATORY_SYNCYTIAL_VIRUS_INFECTION_A594_CELLS_DN	5	0.74	1.77	0.005	0.969	0.977	26	tags=80%, list=16%, signal=92%
AMEF2_Q6	7	0.68	1.89	0.007	0.281	0.718	27	tags=71%, list=16%, signal=82%
MIR1827	7	0.64	1.78	0.007	0.276	0.949	64	tags=100%, list=39%, signal=156%
NKX62_Q2	5	0.75	1.82	0.009	0.255	0.894	44	tags=100%, list=27%, signal=132%
YCATTAA_UNKNOWN	12	0.52	1.82	0.01	0.272	0.887	30	tags=50%, list=18%, signal=57%
DELYS_THYROID_CANCER_DN	7	0.68	1.89	0.012	0.776	0.759	27	tags=71%, list=16%, signal=82%
AAAGACA_MIR511	5	0.76	1.89	0.012	0.243	0.722	24	tags=80%, list=14%, signal=91%
GGATTA_PITX2_Q2	8	0.61	1.78	0.012	0.295	0.949	23	tags=50%, list=14%, signal=55%
HNF1_C	8	0.61	1.83	0.013	0.275	0.868	33	tags=63%, list=20%, signal=74%
NKX61_01	5	0.71	1.76	0.014	0.286	0.96	15	tags=60%, list=9%, signal=64%
ZNF146_TARGET_GENES	8	0.62	1.85	0.015	0.27	0.832	67	tags=100%, list=40%, signal=160%
AAAYRNCTG_UNKNOWN	8	0.58	1.72	0.015	0.338	0.987	30	tags=63%, list=18%, signal=73%
P53_02	7	0.61	1.79	0.017	0.284	0.93	20	tags=57%, list=12%, signal=62%
POU6F1_01	7	0.62	1.73	0.02	0.34	0.983	30	tags=71%, list=18%, signal=84%
MIR8063	5	0.69	1.7	0.021	0.371	0.992	34	tags=80%, list=20%, signal=98%
HNF4ALPHA_Q6	5	0.65	1.59	0.021	0.467	1	16	tags=60%, list=10%, signal=64%
CEBP_01	5	0.68	1.64	0.023	0.451	1	24	tags=60%, list=14%, signal=68%
RODRIGUES_THYROID_CARCINOMA_ANAPLASTIC_DN	9	0.59	1.76	0.026	0.675	0.979	33	tags=67%, list=20%, signal=79%
FOXO4_01	5	0.66	1.6	0.026	0.47	1	58	tags=100%, list=35%, signal=149%
VDR_Q6	6	0.65	1.66	0.027	0.436	0.999	27	tags=67%, list=16%, signal=77%
MIR548N	6	0.64	1.64	0.027	0.466	1	63	tags=100%, list=38%, signal=155%
MIR5696	6	0.63	1.63	0.03	0.477	1	64	tags=100%, list=39%, signal=157%
TGGAAA_NFAT_Q4_01	29	0.33	1.55	0.03	0.48	1	38	tags=41%, list=23%, signal=44%
ER_Q6_01	7	0.58	1.6	0.031	0.484	1	58	tags=86%, list=35%, signal=126%
NFY_C	5	0.65	1.59	0.041	0.456	1	23	tags=60%, list=14%, signal=68%
COUP_01	5	0.67	1.58	0.041	0.444	1	16	tags=60%, list=10%, signal=64%
MIR10527_5P	7	0.58	1.62	0.043	0.463	1	32	tags=57%, list=19%, signal=68%
HAMAI_APOPTOSIS_VIA_TRAIL_UP	5	0.63	1.55	0.047	1	1	24	tags=60%, list=14%, signal=68%
MIR7157_5P	5	0.64	1.56	0.047	0.468	1	23	tags=80%, list=14%, signal=90%

MIR302C_5P	11	0. 48	1.6 1	0.048	0.493	1	33	tags=64%, list=20%, signal=74%
ZNF843_TARGET_GENES	9	0. 51	1.5 9	0.048	0.472	1	69	tags=89%, list=42%, signal=144%

Supplementary Table 5B: Downregulated Gene Set Enrichment Analysis for 167 gene set with nominal p-value < 0.05.

GS	Size	ES	NES	NOM p-val	FDR q-val	FWER p-val	Rank at Max	Leading Edge
NABA_MATRISOME_ASSOCIATED	21	-0.61	-2.29	0	0.012	0.009	34	tags=57%, list=20%, signal=63%
NABA_MATRISOME	24	-0.59	-2.28	0	0.007	0.01	34	tags=54%, list=20%, signal=58%
CREIGHTON_ENDOCRINE_THERAPY_RESISTANCE_3	17	-0.63	-2.19	0	0.009	0.021	29	tags=53%, list=17%, signal=58%
DELYS_THYROID_CANCER_UP	21	-0.58	-2.15	0	0.013	0.037	72	tags=90%, list=43%, signal=140%
NUYTTEN_EZH2_TARGETS_UP	28	-0.54	-2.13	0	0.014	0.052	52	tags=64%, list=31%, signal=78%
LEI_MYB_TARGETS	19	-0.58	-2.11	0	0.017	0.074	61	tags=79%, list=37%, signal=111%
ONDER_CDH1_TARGETS_2_DN	26	-0.53	-2.11	0	0.015	0.074	52	tags=65%, list=31%, signal=80%
BLANCO_MELO_COVID19_BRONCHIAL_EPITHELIAL_CELLS_SARS_COV_2_INFECTION_UP	14	-0.65	-2.1	0	0.014	0.081	56	tags=86%, list=34%, signal=118%
BLANCO_MELO_RESPIRATORY_SYNCYTIAL_VIRUS_INFECTION_A594_CELLS_UP	19	-0.58	-2.06	0	0.018	0.125	72	tags=89%, list=43%, signal=140%
WINTER_HYPOXIA_METAGENE	10	-0.7	-2.04	0	0.022	0.166	31	tags=70%, list=19%, signal=81%
REACTOME_REGULATION_OF_INSULIN_LIKE_GROWTH_FACTOR_IGF_TRANSPORT_AND_UPTAKE_BY_INSULIN_LIKE_GROWTH_FACTOR_BINDING_PROTEINS_IGFBPS	6	-0.81	-1.99	0	0.031	0.301	26	tags=83%, list=16%, signal=95%
WP_OVERVIEW_OF_PROINFLAMMATORY_AND_PROFIBROTIC_MEDIATORS	8	-0.7	-1.91	0	0.052	0.557	26	tags=63%, list=16%, signal=71%
MIYAGAWA_TARGETS_OF_EWSR1_ETS_FUSIONS_UP	5	-0.83	-1.91	0	0.051	0.569	34	tags=100%, list=20%, signal=122%
HALLMARK_EPITHELIAL_MESENCHYMAL_TRANSITION	14	-0.65	-2.14	0	0	0	56	tags=86%, list=34%, signal=118%
HARRIS_HYPOXIA	7	-0.78	-2.01	0.002	0.027	0.235	31	tags=86%, list=19%, signal=101%
SMID_BREAST_CANCER_LUMINAL_B_DN	19	-0.56	-2	0.002	0.03	0.275	52	tags=74%, list=31%, signal=95%
REACTOME_CYTOKINE_SIGNALING_IN_IMMUNE_SYSTEM	16	-0.59	-1.99	0.002	0.03	0.305	57	tags=75%, list=34%, signal=103%
MISSIAGLIA_REGULATED_BY_METHYLATION_UP	12	-0.63	-1.97	0.002	0.031	0.345	70	tags=100%, list=42%, signal=160%
PLASARI_TGFB1_TARGETS_10HR_UP	10	-0.66	-1.95	0.002	0.037	0.414	64	tags=100%, list=39%, signal=153%
ALTEMEIER_RESPONSE_TO_LPS_WITH_MECHANICAL_VENTILATION	10	-0.62	-1.85	0.002	0.067	0.771	71	tags=100%, list=43%, signal=164%
BLANCO_MELO_COVID19_SARS_COV_2_INFECTION_A594_CELLS_UP	5	-0.77	-1.74	0.002	0.105	0.984	27	tags=80%, list=16%, signal=93%

LIANG_SILENCED_BY_METHYLATION_2	9	-0.73	-2.07	0.003	0.019	0.12	52	tags=100%, list=31%, signal=138%
SENESE_HDAC1_AND_HDAC2_TARGETS_UP	13	-0.61	-2.03	0.003	0.023	0.19	20	tags=46%, list=12%, signal=48%
REACTOME_G_ALPHA_I_SIGNALLING_EVENTS	7	-0.72	-1.91	0.003	0.054	0.557	33	tags=71%, list=20%, signal=85%
WP_IL18_SIGNALING_PATHWAY	9	-0.68	-1.88	0.003	0.06	0.68	22	tags=56%, list=13%, signal=61%
LINDSTEDT_DENDRITIC_CELL_MATURATION_A	6	-0.75	-1.86	0.004	0.067	0.762	22	tags=67%, list=13%, signal=74%
GRAESSMANN_APOPTOSIS_BY_SERUM_DEPRIVATION_UP	12	-0.64	-1.97	0.005	0.032	0.34	69	tags=100%, list=42%, signal=159%
KEGG_CYTOKINE_CYTOKINE_RECEPTOR_INTERACTION	8	-0.7	-1.9	0.005	0.053	0.61	26	tags=63%, list=16%, signal=71%
REACTOME_SIGNALING_BY_INTERLEUKINS	13	-0.6	-1.89	0.005	0.059	0.659	57	tags=77%, list=34%, signal=108%
SENESE_HDAC1_TARGETS_UP	15	-0.55	-1.87	0.005	0.063	0.719	33	tags=47%, list=20%, signal=53%
MARTINEZ_RB1_AND_TP53_TARGETS_UP	15	-0.54	-1.84	0.005	0.066	0.818	58	tags=80%, list=35%, signal=112%
RICKMAN_HEAD_AND_NECK_CANCER_E	5	-0.76	-1.72	0.005	0.12	0.993	10	tags=60%, list=6%, signal=62%
NAKAMURA_TUMOR_ZONE_PERIPHERAL_VS_CENTRAL_DN	18	-0.51	-1.82	0.006	0.073	0.853	29	tags=39%, list=17%, signal=42%
FULCHER_INFLAMMATORY_RESPONSE_LECTIN_VS_LPS_UP	15	-0.54	-1.81	0.006	0.078	0.876	31	tags=47%, list=19%, signal=52%
GAURNIER_PSMD4_TARGETS	7	-0.7	-1.84	0.007	0.067	0.805	22	tags=57%, list=13%, signal=63%
REACTOME_POST_TRANSLATIONAL_PROTEIN_MODIFICATION	18	-0.52	-1.87	0.008	0.062	0.726	26	tags=44%, list=16%, signal=47%
ONDER_CDH1_TARGETS_3_DN	8	-0.64	-1.78	0.008	0.095	0.94	24	tags=50%, list=14%, signal=56%
CHARAFE_BREAST_CANCER_LUMINAL_VS_BASAL_DN	18	-0.53	-1.88	0.009	0.062	0.7	89	tags=100%, list=54%, signal=192%
REACTOME_INTERLEUKIN_10_SIGNALING	6	-0.73	-1.78	0.009	0.095	0.949	26	tags=67%, list=16%, signal=76%
REACTOME_INTERLEUKIN_4_AND_INTERLEUKIN_13_SIGNALING	8	-0.65	-1.77	0.009	0.093	0.957	33	tags=63%, list=20%, signal=74%
SWEET_LUNG_CANCER_KRAS_DN	8	-0.66	-1.79	0.01	0.095	0.933	7	tags=38%, list=4%, signal=37%
HINATA_NFKB_TARGETS_FIBROBLAST_UP	7	-0.67	-1.75	0.01	0.102	0.972	22	tags=57%, list=13%, signal=63%
DODD_NASOPHARYNGEAL_CARCINOMA_UP	29	-0.45	-1.84	0.011	0.07	0.789	58	tags=59%, list=35%, signal=74%
COLINA_TARGETS_OF_4EBP1_AND_4EBP2	10	-0.62	-1.84	0.011	0.069	0.805	34	tags=60%, list=20%, signal=71%
REACTOME_INNATE_IMMUNE_SYSTEM	19	-0.49	-1.78	0.011	0.095	0.946	33	tags=47%, list=20%, signal=52%
CAIRO_LIVER_DEVELOPMENT_DN	5	-0.75	-1.71	0.011	0.12	0.994	14	tags=60%, list=8%, signal=64%
REACTOME_PEPTIDE_LIGAND_BINDING_RECEPTORS	8	-0.66	-1.77	0.012	0.094	0.955	44	tags=75%, list=27%, signal=97%

REACTOME_CHEMOKINE_RECEPTORS_BIND_CHEMOKINES	5	-0.74	-1.71	0.012	0.12	0.996	18	tags=60%, list=11%, signal=65%
BUYTAERT_PHOTODYNAMIC_THERAPY_STRESS_DN	8	-0.63	-1.69	0.012	0.127	0.998	56	tags=88%, list=34%, signal=126%
HALLMARK_TNFA_SIGNALING_VIA_NFKB	13	-0.55	-1.79	0.012	0.064	0.075	67	tags=85%, list=40%, signal=131%
REACTOME_CLASS_A_1_RHODOPSIN_LIKE_RECEPTORS	8	-0.66	-1.76	0.013	0.094	0.963	44	tags=75%, list=27%, signal=97%
CHYLA_CBFA2T3_TARGETS_UP	6	-0.7	-1.71	0.013	0.12	0.995	29	tags=67%, list=17%, signal=78%
WP_NETWORK_MAP_OF_SARSCOV2_SIGNALING_PATHWAY	12	-0.54	-1.68	0.013	0.129	0.998	69	tags=83%, list=42%, signal=132%
HES2_TARGET_GENES	23	-0.46	-1.73	0.013	1	0.987	33	tags=39%, list=20%, signal=42%
SABATES_COLORECTAL_ADENOMA_UP	9	-0.6	-1.71	0.014	0.121	0.994	18	tags=44%, list=11%, signal=47%
GERY_CEBP_TARGETS	6	-0.67	-1.68	0.014	0.127	0.998	14	tags=50%, list=8%, signal=53%
MIR33A_3P	5	-0.72	-1.63	0.014	1	0.998	13	tags=60%, list=8%, signal=63%
NUYTTEN_NIPP1_TARGETS_DN	11	-0.59	-1.78	0.015	0.096	0.955	76	tags=100%, list=46%, signal=172%
PEDRIOLI_MIR31_TARGETS_DN	14	-0.54	-1.77	0.015	0.094	0.955	71	tags=86%, list=43%, signal=137%
MARTINEZ_TP53_TARGETS_UP	14	-0.53	-1.75	0.015	0.102	0.975	28	tags=50%, list=17%, signal=55%
ONDER_CDH1_TARGETS_1_DN	8	-0.64	-1.74	0.015	0.106	0.983	24	tags=50%, list=14%, signal=56%
TONKS_TARGETS_OF_RUNX1_RUNX1T1_FUSION_HSC_UP	10	-0.59	-1.71	0.015	0.12	0.995	37	tags=60%, list=22%, signal=73%
HINATA_NFKB_TARGETS_KERATINOCYTE_UP	8	-0.63	-1.74	0.016	0.106	0.985	67	tags=100%, list=40%, signal=160%
SMIRNOV_CIRCULATING_ENDOTHELIOCYTES_IN_CANCER_UP	8	-0.63	-1.72	0.016	0.12	0.994	18	tags=50%, list=11%, signal=53%
LEE_LIVER_CANCER_CIPROFIBRATE_UP	5	-0.73	-1.7	0.016	0.125	0.998	16	tags=60%, list=10%, signal=64%
WINZEN_DEGRADED_VIA_KHSRP	10	-0.62	-1.84	0.017	0.066	0.811	31	tags=60%, list=19%, signal=69%
WP_FERROPTOSIS	6	-0.68	-1.7	0.017	0.122	0.996	14	tags=50%, list=8%, signal=53%
JINESH_BLEBBISHIELD_VS_LIVE_CONTROL_DN	12	-0.57	-1.77	0.018	0.095	0.957	48	tags=67%, list=29%, signal=87%
SMID_BREAST_CANCER_BASAL_UP	18	-0.5	-1.79	0.019	0.095	0.936	51	tags=67%, list=31%, signal=86%
GRAESSMANN_RESPONSE_TO_MC_AND_SERUM_DEPRIVATION_UP	8	-0.62	-1.66	0.019	0.129	0.999	69	tags=100%, list=42%, signal=163%
ZNF513_TARGET_GENES	5	-0.72	-1.64	0.019	1	0.998	19	tags=60%, list=11%, signal=66%
BLANCO_MELO_COVID19_SARS_COV_2_POS_PATIENT_LUNG_TISSUE_UP	5	-0.73	-1.68	0.02	0.128	0.998	49	tags=100%, list=30%, signal=138%
REACTOME_GPCR_LIGAND_BINDING	12	-0.53	-1.68	0.021	0.125	0.998	33	tags=50%, list=20%, signal=58%

ZWANG_CLASS_3_TRANSIENTLY_INDUCED_BY_EGF	10	-0.58	-1.66	0.021	0.131	0.998	68	tags=90%, list=41%, signal=143%
REACTOME_HEMOSTASIS	9	-0.58	-1.65	0.021	0.131	1	31	tags=56%, list=19%, signal=65%
HELLEBREKERS_SILENCED_DURING_TUMOR_ANGIOGENESIS	8	-0.62	-1.68	0.022	0.126	0.998	57	tags=88%, list=34%, signal=127%
REACTOME_NEUTROPHIL_DEGRANULATION	9	-0.61	-1.72	0.025	0.122	0.994	29	tags=56%, list=17%, signal=64%
BLANCO_MELO_COVID19_SARS_COV_2_INFECTION_A594_ACE2_EXPRESSING_CELLS_UP	12	-0.54	-1.69	0.025	0.128	0.998	31	tags=50%, list=19%, signal=57%
BLANCO_MELO_COVID19_SARS_COV_2_INFECTION_CALU3_CELLS_UP	13	-0.52	-1.67	0.025	0.127	0.998	72	tags=85%, list=43%, signal=138%
BLANCO_MELO_BETA_INTERFERON_TREATED_BRONCHIAL_EPITHELIAL_CELLS_UP	19	-0.46	-1.66	0.026	0.129	0.999	54	tags=63%, list=33%, signal=83%
RUTELLA_RESPONSE_TO_HGF_UP	12	-0.52	-1.63	0.026	0.143	1	30	tags=42%, list=18%, signal=47%
HALLMARK_COMPLEMENT	10	-0.57	-1.68	0.026	0.109	0.185	69	tags=90%, list=42%, signal=145%
SMID_BREAST_CANCER_RELAPSE_IN_BONE_DN	14	-0.51	-1.69	0.027	0.129	0.998	51	tags=71%, list=31%, signal=94%
KOINUMA_TARGETS_OF_SMAD2_OR_SMAD3	17	-0.48	-1.69	0.027	0.128	0.998	74	tags=82%, list=45%, signal=133%
JINESH_BLEBBISHIELD_TO_IMMUNE_CELL_FUSION_PBSHMS_DN	17	-0.49	-1.68	0.027	0.13	0.998	30	tags=41%, list=18%, signal=45%
DAZARD_RESPONSE_TO_UV_NHEK_UP	10	-0.58	-1.67	0.027	0.125	0.998	28	tags=50%, list=17%, signal=57%
FOROUTAN_PRODRANK_TGFB_EMT_UP	7	-0.63	-1.65	0.027	0.132	1	56	tags=86%, list=34%, signal=124%
GROSS_HYPOXIA_VIA_ELK3_ONLY_UP	6	-0.68	-1.66	0.029	0.13	0.999	29	tags=67%, list=17%, signal=78%
PETROVA_ENDOTHELIUM_LYMPHATIC_VS_BLOOD_DN	16	-0.49	-1.64	0.03	0.138	1	60	tags=69%, list=36%, signal=97%
PHONG_TNF_RESPONSE_VIA_P38_PARTIAL	11	-0.55	-1.68	0.031	0.124	0.998	82	tags=100%, list=49%, signal=185%
DAZARD_RESPONSE_TO_UV_SCC_UP	5	-0.71	-1.65	0.031	0.133	1	22	tags=60%, list=13%, signal=67%
GROSS_HYPOXIA_VIA_ELK3_DN	9	-0.57	-1.63	0.032	0.144	1	78	tags=100%, list=47%, signal=178%
WESTON_VEGFA_TARGETS_3HR	6	-0.64	-1.61	0.032	0.149	1	47	tags=83%, list=28%, signal=112%
SENESE_HDAC2_TARGETS_UP	5	-0.67	-1.56	0.032	0.177	1	59	tags=100%, list=36%, signal=150%
SANA_TNF_SIGNALING_UP	7	-0.64	-1.66	0.033	0.13	0.999	52	tags=86%, list=31%, signal=120%
FOSTER_TOLERANT_MACROPHAGE_UP	10	-0.55	-1.62	0.033	0.149	1	21	tags=40%, list=13%, signal=43%
FOROUTAN_TGFB_EMT_UP	7	-0.63	-1.61	0.033	0.15	1	56	tags=86%, list=34%, signal=124%
MARTINEZ_RB1_TARGETS_UP	15	-0.49	-1.66	0.034	0.131	0.999	59	tags=73%, list=36%, signal=103%
RUTELLA_RESPONSE_TO_HGF_VS_CSF2RB_AND_IL4_UP	12	-0.52	-1.64	0.034	0.138	1	30	tags=42%, list=18%, signal=47%

SMIRNOV_RESPONSE_TO_IR_6HR_UP	5	-0.68	-1.57	0.034	0.174	1	58	tags=100%, list=35%, signal=149%
ROZANOV_MMP14_TARGETS_UP	10	-0.56	-1.62	0.035	0.15	1	50	tags=70%, list=30%, signal=94%
SCHUETZ_BREAST_CANCER_DUCTAL_INVASIVE_UP	15	-0.47	-1.62	0.036	0.148	1	25	tags=33%, list=15%, signal=36%
WP_VEGFAVEGFR2_SIGNALING_PATHWAY	6	-0.64	-1.58	0.036	0.175	1	64	tags=100%, list=39%, signal=157%
ONDER_CDH1_SIGNALING_VIA_CTNNB1	7	-0.61	-1.55	0.036	0.184	1	33	tags=57%, list=20%, signal=68%
CHICAS_RB1_TARGETS_SENESCENT	12	-0.5	-1.57	0.037	0.177	1	30	tags=42%, list=18%, signal=47%
HALLMARK_COAGULATION	7	-0.62	-1.65	0.037	0.106	0.234	69	tags=100%, list=42%, signal=164%
ENK_UV_RESPONSE_KERATINOCYTE_UP	13	-0.5	-1.61	0.038	0.151	1	67	tags=85%, list=40%, signal=131%
SMID_BREAST_CANCER_NORMAL_LIKE_UP	6	-0.64	-1.59	0.038	0.162	1	33	tags=83%, list=20%, signal=100%
ZHU_CMV_ALL_UP	7	-0.6	-1.57	0.038	0.178	1	72	tags=100%, list=43%, signal=169%
LIAO_METASTASIS	6	-0.65	-1.6	0.04	0.157	1	51	tags=83%, list=31%, signal=116%
KIM_WT1_TARGETS_UP	10	-0.56	-1.61	0.041	0.15	1	71	tags=90%, list=43%, signal=148%
MARTINEZ_RB1_TARGETS_DN	7	-0.6	-1.56	0.041	0.179	1	57	tags=86%, list=34%, signal=125%
NABA_SECRETED_FACTORS	13	-0.49	-1.56	0.041	0.178	1	34	tags=46%, list=20%, signal=53%
PHONG_TNF_TARGETS_UP	9	-0.57	-1.63	0.042	0.144	1	67	tags=89%, list=40%, signal=141%
BROWN_MYELOID_CELL_DEVELOPMENT_UP	5	-0.68	-1.56	0.042	0.181	1	28	tags=60%, list=17%, signal=70%
BOYLAN_MULTIPLE_MYELOMA_C_D_DN	9	-0.58	-1.65	0.043	0.133	1	29	tags=56%, list=17%, signal=64%
SMID_BREAST_CANCER_RELAPSE_IN_BONE_UP	5	-0.66	-1.53	0.043	0.193	1	60	tags=100%, list=36%, signal=152%
MIR5700	5	-0.65	-1.49	0.044	1	1	10	tags=40%, list=6%, signal=41%
HELLER_HDAC_TARGETS_SILENCED_BY_METHYLATION_UP	11	-0.52	-1.57	0.045	0.175	1	59	tags=73%, list=36%, signal=105%
WESTON_VEGFA_TARGETS	6	-0.64	-1.57	0.045	0.175	1	47	tags=83%, list=28%, signal=112%
TENEDINI_MEGAKARYOCYTE_MARKERS	5	-0.68	-1.56	0.045	0.179	1	22	tags=60%, list=13%, signal=67%
CREB3L4_TARGET_GENES	10	-0.52	-1.57	0.045	1	1	26	tags=40%, list=16%, signal=45%
BUYTAERT_PHOTODYNAMIC_THERAPY_STRESS_UP	17	-0.46	-1.58	0.046	0.168	1	74	tags=76%, list=45%, signal=124%
KEGG_CHEMOKINE_SIGNALING_PATHWAY	6	-0.63	-1.54	0.046	0.188	1	18	tags=50%, list=11%, signal=54%
WP_NRF2_PATHWAY	6	-0.62	-1.53	0.046	0.194	1	17	tags=50%, list=10%, signal=54%

HORIUCHI_WTAP_TARGETS_UP	11	-0.5	-1.53	0.046	0.192	1	69	tags=82%, list=42%, signal=131%
FOROUTAN_INTEGRATED_TGFB_EMT_UP	5	-0.68	-1.56	0.048	0.176	1	24	tags=60%, list=14%, signal=68%
BASAKI_YBX1_TARGETS_UP	5	-0.65	-1.5	0.048	0.21	1	46	tags=80%, list=28%, signal=107%
JOHNSTONE_PARVB_TARGETS_3_UP	11	-0.49	-1.52	0.049	0.198	1	26	tags=36%, list=16%, signal=40%

Supplementary Table 6: Reactome pathway analysis of 167 Common HRTPT cells exposed to iAs passage P3, P8, P10 Genes. * indicates p < 0.05, ** indicates p < 0.01.

Analysis of 167 Common iAS P3, P8, P10 Genes

FGFR2 ligand binding and activation 8.93e-08**
FGFR2 mutant receptor activation 2.05e-07 *
Interleukin-10 signaling 2.05e-06 **
SHC-mediated cascade: FGFR2 3.82e-06*
Interleukin-4 and Interleukin-13 signaling 7.91e-06 **
Activated point mutants of FGFR2 1.92e-05*
Signaling by FGFR2 IIIa TM 4.40e- 05*
FGFR2c ligand binding and activation 1.16e-04
Constitutive Signaling by Aberrant PI3K in Cancer 5.12e-04*
Interleukin-18 signaling 7.08e-04 **
Phospholipase C-mediated cascade; FGFR2 0.001*
FGFR2b ligand binding and activation 0.001
Chemokine receptors bind chemokines 0.002
Signaling by FGFR2 amplification mutants 0.002*
GRB2 events in ERBB2 signaling 0.002*
Post-translational protein phosphorylation 0.002 **
Signaling by FGFR2 in disease 0.003 *
RAF/MAP kinase cascade 0.006*
PI5P, PP2A and IER3 Regulate PI3K/AKT Signaling 0.011*
FGFR3b ligand binding and activation 0.011
MAPK1/MAPK3 signaling 0.011
ERBB2 Regulates Cell Motility 0.011*
Molecules associated with elastic fibres 0.013d
Negative regulation of the PI3K/AKT network 0.018*
Signaling by FGFR in disease 0.018*

Unique Pathways of 76 Down-regulated Genes Set

FGFR2c ligand binding and activation
ERBB2 Activates PTK6 Signaling
PI3K Cascade
FRS-mediated FGFR2 signaling
IRS-mediated signaling
Signaling by Insulin receptor
IRS-related events triggered by IGF1R
IGF1R signaling cascade
Signaling by Type 1 Insulin-like Growth Factor 1 Receptor (IGF1R)
SHC1 events in ERBB4 signaling

Unique Pathways of 91 Up-regulated Genes Set

Chemokine receptors bind chemokines6.96e-05
Post-translational protein phosphorylation9.99e-05
Senescence-Associated Secretory Phenotype (SASP) 0.002

Molecules associated with elastic fibres 0.003
Heme degradation 0.004
Peptide ligand-binding receptors 0.008
Glucuronidation 0.008
Regulation of Insulin-like Growth Factor (IGF) transport and uptake by Insulin-like Growth Factor Binding Proteins (IGFBPs) 0.008
Alternative complement activation 0.009
Elastic fibre formation 0.009
Transcriptional activation of cell cycle inhibitor p21 0.011
Transcriptional activation of p53 responsive genes 0.011
Neutrophil degranulation 0.015
RAF-independent MAPK1/3 activation 0.016
Metabolism of porphyrins 0.023
Aspirin ADME 0.025
Basigin interactions 0.025
Defective UGT1A4 causes hyperbilirubinemia 0.026
Defective UGT1A1 causes hyperbilirubinemia 0.026
Defective SLC40A1 causes hemochromatosis 4 (HFE4) (macrophages) 0.026
Activation of C3 and C5 0.032

Supplementary Table 7A: Upregulated Gene Set Enrichment Analysis for significant genes between the control vs iAs+ with nominal p-value < 0.05.

GS	Size	ES	NES	NOM p-val	FDR q-val	FWER p-val	Rank at Max	Leading Edge
REACTOME_ADAPTIVE_IMMUNE_SYSTEM	10	0.77	2.06	0	0.063	0.053	17	tags=60%, list=7%, signal=62%
SMID_BREAST_CANCER_NORMAL_LIKE_UP	12	0.72	2.05	0	0.037	0.063	36	tags=67%, list=15%, signal=75%
WP_NETWORK_MAP_OF_SARSCOV2_SIGNALING_PATHWAY	18	0.62	1.96	0	0.087	0.205	69	tags=72%, list=29%, signal=95%
CREIGHTON_ENDOCRINE_THERAPY_RESISTANCE_3	28	0.56	1.96	0	0.076	0.232	36	tags=43%, list=15%, signal=45%
HAN_JNK_SINGALING_DN	5	0.88	1.92	0	0.049	0.344	17	tags=80%, list=7%, signal=84%
REACTOME_CYTOKINE_SIGNALING_IN_IMMUNE_SYSTEM	25	0.56	1.91	0	0.057	0.422	99	tags=84%, list=42%, signal=130%
SENGUPTA_NASOPHARYNGEAL_CARCINOMA_WITH_LMP1_DN	9	0.73	1.9	0	0.05	0.439	31	tags=67%, list=13%, signal=74%
REACTOME_INNATE_IMMUNE_SYSTEM	28	0.54	1.88	0	0.056	0.522	30	tags=36%, list=13%, signal=36%
REACTOME_SIGNALING_BY_INTERLEUKINS	17	0.6	1.84	0	0.075	0.749	43	tags=53%, list=18%, signal=60%
KEGG_COMPLEMENT_AND_COAGULATION_CASCADES	5	0.84	1.78	0	0.091	0.95	25	tags=80%, list=11%, signal=88%
WP_ANTIVIRAL_AND_ANTIINFLAMMATORY_EFFECTS_OF_NRF2_ON_SARSCOV2_PATHWAY	5	0.81	1.76	0	0.096	0.968	29	tags=80%, list=12%, signal=89%
REACTOME_COMPLEMENT_CASCADE	5	0.81	1.73	0	0.114	0.994	14	tags=60%, list=6%, signal=62%
HSIAO_LIVER_SPECIFIC_GENES	9	0.75	1.94	0.001	0.062	0.274	22	tags=56%, list=9%, signal=59%
COLINA_TARGETS_OF_4EBP1_AND_4EBP2	12	0.68	1.94	0.001	0.055	0.284	25	tags=50%, list=11%, signal=53%
GAURNIER_PSMD4_TARGETS	9	0.73	1.93	0.001	0.051	0.296	29	tags=67%, list=12%, signal=73%
SMID_BREAST_CANCER_LUMINAL_B_DN	28	0.55	1.93	0.001	0.046	0.302	69	tags=61%, list=29%, signal=76%
HARRIS_HYPOXIA	9	0.73	1.9	0.001	0.049	0.456	29	tags=67%, list=12%, signal=73%
FOSTER_TOLERANT_MACROPHAGE_UP	8	0.75	1.87	0.001	0.059	0.575	28	tags=63%, list=12%, signal=69%
DELYS_THYROID_CANCER_UP	34	0.51	1.84	0.001	0.073	0.716	103	tags=79%, list=44%, signal=121%
REACTOME_PEPTIDE_LIGAND_BINDING_RECEPTORS	9	0.71	1.82	0.001	0.083	0.816	39	tags=67%, list=17%, signal=77%
KIM_GLIS2_TARGETS_UP	8	0.72	1.8	0.001	0.087	0.886	17	tags=50%, list=7%, signal=52%
HES2_TARGET_GENES	32	0.55	1.96	0.001	0.157	0.187	45	tags=44%, list=19%, signal=47%
E2F5_TARGET_GENES	6	0.78	1.76	0.001	1	0.945	38	tags=83%, list=16%, signal=97%

HALLMARK_EPITHELIAL_MESENCHYMAL_TRANSITION	18	0.59	1.89	0.001	0.027	0.021	99	tags=94%, list=42%, signal=151%
IWANAGA_CARCINOGENESIS_BY_KRAS_PTEN_UP	5	0.83	1.81	0.002	0.087	0.878	25	tags=80%, list=11%, signal=88%
REACTOME_HEMOSTASIS	16	0.58	1.78	0.002	0.09	0.938	32	tags=44%, list=14%, signal=47%
REACTOME_MHC_CLASS_II_ANTIGEN_PRESENTATION	5	0.82	1.76	0.002	0.097	0.975	17	tags=80%, list=7%, signal=84%
BOYLAN_MULTIPLE_MYELOMA_PCA1_UP	5	0.82	1.74	0.002	0.111	0.99	8	tags=60%, list=3%, signal=61%
LIANG_SILENCED_BY_METHYLATION_2	11	0.71	1.94	0.003	0.072	0.27	56	tags=82%, list=24%, signal=103%
WINZEN_DEGRADED_VIA_KHSRP	10	0.69	1.9	0.003	0.054	0.432	44	tags=70%, list=19%, signal=83%
REACTOME_INTERLEUKIN_4_AND_INTERLEUKIN_13_SIGNALING	8	0.73	1.83	0.003	0.081	0.794	29	tags=63%, list=12%, signal=69%
BLANCO_MELO_COVID19_SARS_COV_2_INFECTION_A594_CELLS_UP	5	0.81	1.76	0.003	0.097	0.975	29	tags=80%, list=12%, signal=89%
REACTOME_CELL_SURFACE_INTERACTIONS_AT_THE_VASCULAR_WALL	8	0.7	1.72	0.003	0.111	0.995	32	tags=63%, list=14%, signal=70%
JAATINEN_HEMATOPOIETIC_STEM_CELL_DN	5	0.77	1.65	0.003	0.169	1	12	tags=60%, list=5%, signal=62%
HORIUCHI_WTAP_TARGETS_UP	10	0.7	1.88	0.004	0.055	0.537	76	tags=100%, list=32%, signal=142%
JINESH_BLEBBISHIELD_VS_LIVE_CONTROL_DN	21	0.57	1.84	0.004	0.076	0.712	80	tags=71%, list=34%, signal=99%
REACTOME_CLASS_A_1_RHODOPSIN_LIKE_RECEPTORS	9	0.71	1.81	0.004	0.089	0.871	39	tags=67%, list=17%, signal=77%
SENESE_HDAC1_AND_HDAC2_TARGETS_UP	14	0.62	1.79	0.004	0.088	0.922	67	tags=71%, list=29%, signal=94%
CHIANG_LIVER_CANCER_SUBCLASS_CTNNB1_DN	9	0.7	1.78	0.004	0.092	0.946	45	tags=78%, list=19%, signal=93%
RICKMAN_HEAD_AND_NECK_CANCER_E	8	0.7	1.78	0.004	0.092	0.956	31	tags=63%, list=13%, signal=70%
REACTOME_REGULATION_OF_INSULIN_LIKE_GROWTH_FACTOR_IGF_TRANSPORT_AND_UPTAKE_BY_INSULIN_LIKE_GROWTH_FACTOR_BINDING_PROTEINS_IGFBPS	8	0.72	1.77	0.004	0.09	0.958	71	tags=100%, list=30%, signal=139%
REACTOME_G_ALPHA_I_SIGNALLING_EVENTS	9	0.73	1.85	0.005	0.076	0.693	26	tags=56%, list=11%, signal=60%
BIOCARTA_INFLAM_PATHWAY	5	0.83	1.8	0.005	0.086	0.898	43	tags=100%, list=18%, signal=120%
BLANCO_MELO_RESPIRATORY_SYNCYTIAL_VIRUS_INFECTION_A594_CELLS_UP	17	0.58	1.79	0.005	0.09	0.935	86	tags=82%, list=37%, signal=121%
BOQUEST_STEM_CELL_DN	10	0.64	1.71	0.005	0.124	0.999	48	tags=60%, list=21%, signal=72%
BLANCO_MELO_COVID19_BRONCHIAL_EPITHELIAL_CELLS_SARS_COV_2_INFECTION_UP	18	0.58	1.82	0.006	0.088	0.846	76	tags=72%, list=32%, signal=99%
GRAESSMANN_APOPTOSIS_BY_SERUM_DEPRIVATION_UP	18	0.56	1.73	0.006	0.113	0.994	85	tags=78%, list=36%, signal=113%
WINTER_HYPOXIA_METAGENE	14	0.59	1.73	0.006	0.111	0.994	71	tags=71%, list=30%, signal=96%

WP_COMPLEMENT_SYSTEM	5	0.79	1.69	0.006	0.133	1	14	tags=60%, list=6%, signal=62%
BLANCO_MELO_BETA_INTERFERON_TREATED_BRONCHIAL_EPITHELIAL_CELLS_UP	21	0.54	1.75	0.007	0.104	0.979	80	tags=76%, list=34%, signal=105%
JINESH_BLEBBISHIELD_TO_IMMUNE_CELL_FUSION_PBSHMS_DN	26	0.49	1.66	0.007	0.167	1	56	tags=46%, list=24%, signal=54%
NABA_MATRISOME	33	0.47	1.65	0.007	0.167	1	73	tags=55%, list=31%, signal=68%
PILON_KLF1_TARGETS_UP	9	0.69	1.81	0.008	0.091	0.862	24	tags=44%, list=10%, signal=48%
POOLA_INVASIVE_BREAST_CANCER_UP	10	0.66	1.74	0.008	0.108	0.985	40	tags=60%, list=17%, signal=69%
HALLMARK_COAGULATION	10	0.65	1.75	0.008	0.073	0.106	31	tags=50%, list=13%, signal=55%
DODD_NASOPHARYNGEAL_CARCINOMA_UP	42	0.46	1.71	0.009	0.12	0.999	105	tags=71%, list=45%, signal=106%
LEE_LIVER_CANCER_E2F1_UP	5	0.77	1.67	0.009	0.152	1	17	tags=60%, list=7%, signal=63%
MARTINEZ_TP53_TARGETS_UP	18	0.57	1.79	0.01	0.09	0.92	19	tags=33%, list=8%, signal=33%
NABA_MATRISOME_ASSOCIATED	24	0.53	1.76	0.01	0.096	0.972	73	tags=63%, list=31%, signal=82%
MARTINEZ_RB1_AND_TP53_TARGETS_UP	19	0.55	1.73	0.01	0.111	0.992	19	tags=32%, list=8%, signal=32%
WP_ALLOGRAFT_REJECTION	6	0.75	1.72	0.01	0.115	0.998	48	tags=83%, list=21%, signal=102%
HALLMARK_TNFA_SIGNALING_VIA_NFKB	18	0.55	1.73	0.01	0.061	0.129	99	tags=89%, list=42%, signal=142%
MIR3671	7	0.71	1.67	0.011	1	0.997	47	tags=71%, list=20%, signal=87%
MCLACHLAN_DENTAL_CARIES_UP	23	0.53	1.75	0.013	0.104	0.982	55	tags=52%, list=24%, signal=62%
NAKAMURA_TUMOR_ZONE_PERIPHERAL_VS_CENTRAL_DN	23	0.51	1.66	0.013	0.165	1	53	tags=43%, list=23%, signal=51%
WP_COMPLEMENT_AND_COAGULATION_CASCADES	5	0.75	1.64	0.013	0.177	1	25	tags=60%, list=11%, signal=66%
HALMOS_CEBPA_TARGETS_UP	6	0.73	1.64	0.013	0.175	1	67	tags=100%, list=29%, signal=137%
THUM_SYSTOLIC_HEART_FAILURE_UP	17	0.56	1.75	0.014	0.103	0.979	53	tags=53%, list=23%, signal=63%
ALTEMEIER_RESPONSE_TO_LPS_WITH_MECHANICAL_VENTILATION	12	0.61	1.7	0.014	0.12	0.999	80	tags=83%, list=34%, signal=120%
GRAESSMANN_APOPTOSIS_BY_DOXORUBICIN_UP	30	0.48	1.66	0.014	0.167	1	106	tags=80%, list=45%, signal=128%
REACTOME_GPCR_LIGAND_BINDING	13	0.59	1.73	0.015	0.108	0.995	44	tags=54%, list=19%, signal=63%
WP_OVERVIEW_OF_PROINFLAMMATORY_AND_PROFIBROTIC_MEDIATORS	8	0.67	1.7	0.015	0.122	0.999	69	tags=88%, list=29%, signal=120%
IGLESIAS_E2F_TARGETS_UP	5	0.76	1.63	0.015	0.175	1	17	tags=60%, list=7%, signal=63%
MIR493_5P	5	0.74	1.6	0.015	1	1	26	tags=60%, list=11%, signal=66%

BLANCO_MELO_COVID19_SARS_COV_2_INFECTION_A594_ACE2_EXPRESSING_CELLS_UP	15	0.58	1.73	0.016	0.11	0.995	69	tags=73%, list=29%, signal=97%
LEE_LIVER_CANCER_CIPROFIBRATE_UP	7	0.68	1.66	0.018	0.169	1	13	tags=43%, list=6%, signal=44%
LIN_SILENCED_BY_TUMOR_MICROENVIRONMENT	9	0.63	1.65	0.018	0.17	1	42	tags=56%, list=18%, signal=65%
SENGUPTA_NASOPHARYNGEAL_CARCINOMA_DN	12	0.57	1.63	0.018	0.175	1	69	tags=67%, list=29%, signal=90%
LINDSTEDT_DENDRITIC_CELL_MATURATION_A	7	0.67	1.62	0.018	0.193	1	29	tags=57%, list=12%, signal=63%
CAIRO_LIVER_DEVELOPMENT_DN	8	0.66	1.61	0.018	0.19	1	32	tags=50%, list=14%, signal=56%
ONDER_CDH1_TARGETS_2_DN	39	0.43	1.59	0.018	0.209	1	102	tags=67%, list=44%, signal=98%
ONDER_CDH1_TARGETS_1_DN	16	0.56	1.69	0.019	0.135	1	83	tags=75%, list=35%, signal=108%
REACTOME_INTERLEUKIN_10_SIGNALING	7	0.69	1.6	0.019	0.203	1	43	tags=71%, list=18%, signal=85%
LEE_LIVER_CANCER_DENA_UP	5	0.72	1.56	0.019	0.226	1	5	tags=40%, list=2%, signal=40%
BOSCO_EPITHELIAL_DIFFERENTIATION_MODULE	8	0.66	1.64	0.02	0.173	1	30	tags=50%, list=13%, signal=55%
BLANCO_MELO_COVID19_SARS_COV_2_LOW_MOI_INFECTION_A594_ACE2_EXPRESSING_CELLS_UP	7	0.66	1.61	0.02	0.191	1	69	tags=86%, list=29%, signal=118%
HALLMARK_COMPLEMENT	13	0.56	1.61	0.02	0.11	0.345	29	tags=38%, list=12%, signal=41%
KEGG_LEISHMANIA_INFECTION	5	0.76	1.64	0.021	0.171	1	15	tags=60%, list=6%, signal=63%
DAZARD_RESPONSE_TO_UV_NHEK_UP	11	0.58	1.62	0.021	0.192	1	69	tags=73%, list=29%, signal=98%
WP_SARSCOV2_INNATE_IMMUNITY_EVASION_AND_CELLSPECIFIC_IMMUNE_RESPONSE	6	0.72	1.64	0.022	0.177	1	69	tags=100%, list=29%, signal=138%
WP_CYTOKINES_AND_INFLAMMATORY_RESPONSE	5	0.73	1.58	0.022	0.208	1	43	tags=80%, list=18%, signal=96%
PICCALUGA_ANGIOIMMUNOBLASTIC_LYMPHOMA_UP	6	0.69	1.58	0.022	0.209	1	14	tags=50%, list=6%, signal=52%
ZHOU_INFLAMMATORY_RESPONSE_LIVE_UP	22	0.5	1.63	0.023	0.177	1	102	tags=82%, list=44%, signal=131%
PLASARI_TGFB1_TARGETS_10HR_UP	12	0.57	1.61	0.023	0.196	1	87	tags=83%, list=37%, signal=126%
PHONG_TNF_RESPONSE_VIA_P38_PARTIAL	15	0.54	1.6	0.023	0.202	1	99	tags=87%, list=42%, signal=141%
SCHUETZ_BREAST_CANCER_DUCTAL_INVASIVE_UP	22	0.51	1.67	0.024	0.157	1	53	tags=50%, list=23%, signal=59%
BOQUEST_STEM_CELL_UP	9	0.61	1.58	0.024	0.21	1	25	tags=44%, list=11%, signal=48%
HUPER_BREAST_BASAL_VS_LUMINAL_DN	7	0.66	1.58	0.024	0.21	1	53	tags=71%, list=23%, signal=90%
TENEDINI_MEGAKARYOCYTE_MARKERS	7	0.64	1.56	0.024	0.229	1	36	tags=57%, list=15%, signal=66%
MIR33A_3P	5	0.73	1.55	0.024	1	1	28	tags=60%, list=12%, signal=67%

HINATA_NFKB_TARGETS_FIBROBLAST_UP	10	0.61	1.64	0.025	0.177	1	76	tags=80%, list=32%, signal=113%
NAKAMURA_TUMOR_ZONE_PERIPHERAL_VS_CENTRAL_UP	10	0.6	1.58	0.025	0.209	1	25	tags=40%, list=11%, signal=43%
SWEET_LUNG_CANCER_KRAS_DN	10	0.61	1.62	0.026	0.186	1	8	tags=30%, list=3%, signal=30%
SMID_BREAST_CANCER_BASAL_UP	26	0.47	1.6	0.026	0.203	1	76	tags=58%, list=32%, signal=76%
REACTOME_CHEMOKINE_RECEPTORS_BIND_CHEMOKINES	5	0.72	1.55	0.026	0.23	1	26	tags=60%, list=11%, signal=66%
ZSCAN2_TARGET_GENES	9	0.63	1.63	0.026	1	1	14	tags=33%, list=6%, signal=34%
MIR3529_3P	5	0.73	1.57	0.026	1	1	31	tags=60%, list=13%, signal=68%
CHIARADONNA_NEOPLASTIC_TRANSFORMATION_CDC25_UP	6	0.7	1.57	0.027	0.217	1	43	tags=67%, list=18%, signal=80%
MISSIAGLIA_REGULATED_BY_METHYLATION_UP	13	0.57	1.65	0.028	0.166	1	106	tags=100%, list=45%, signal=173%
HELLER_SILENCED_BY_METHYLATION_UP	14	0.54	1.57	0.029	0.219	1	56	tags=57%, list=24%, signal=71%
NAKAYAMA_SOFT_TISSUE_TUMORS_PCA1_UP	6	0.69	1.54	0.029	0.237	1	14	tags=50%, list=6%, signal=52%
KEGG_CYTOKINE_CYTOKINE_RECEPTOR_INTERACTION	12	0.59	1.62	0.03	0.185	1	43	tags=50%, list=18%, signal=58%
HINATA_NFKB_TARGETS_KERATINOCYTE_UP	11	0.57	1.59	0.03	0.209	1	76	tags=73%, list=32%, signal=103%
NUYTTEN_EZH2_TARGETS_UP	40	0.42	1.55	0.031	0.231	1	99	tags=65%, list=42%, signal=93%
WP_CHEMOKINE_SIGNALING_PATHWAY	5	0.7	1.51	0.031	0.266	1	5	tags=40%, list=2%, signal=40%
GRAHAM_CML_QUIESCENT_VS_CML_DIVIDING_UP	5	0.69	1.49	0.031	0.276	1	76	tags=100%, list=32%, signal=145%
NABA_SECRETED_FACTORS	13	0.56	1.61	0.032	0.197	1	73	tags=69%, list=31%, signal=95%
SABATES_COLORECTAL_ADENOMA_UP	11	0.57	1.57	0.032	0.221	1	26	tags=36%, list=11%, signal=39%
MAHAJAN_RESPONSE_TO_IL1A_UP	6	0.69	1.57	0.033	0.22	1	76	tags=100%, list=32%, signal=144%
HALLMARK_ALLOGRAFT_REJECTION	7	0.67	1.62	0.034	0.134	0.334	48	tags=71%, list=21%, signal=87%
IVANOVA_HEMATOPOIESIS_MATURE_CELL	6	0.66	1.52	0.035	0.261	1	5	tags=33%, list=2%, signal=33%
WP_COMPLEMENT_SYSTEM_IN_NEURONAL_DEVELOPMENT_AND_PLASTICITY	7	0.67	1.59	0.036	0.206	1	14	tags=43%, list=6%, signal=44%
ZWANG_TRANSIENTLY_UP_BY_2ND_EGF_PULSE_ONLY	19	0.49	1.56	0.036	0.231	1	42	tags=42%, list=18%, signal=47%
ONDER_CDH1_TARGETS_3_DN	11	0.58	1.59	0.037	0.21	1	83	tags=82%, list=35%, signal=121%
ICHIBA_GRAFT_VERSUS_HOST_DISEASE_35D_UP	6	0.67	1.54	0.038	0.238	1	80	tags=100%, list=34%, signal=148%
ZNF318_TARGET_GENES	7	0.65	1.59	0.038	1	1	51	tags=71%, list=22%, signal=89%

BROWN_MYELOID_CELL_DEVELOPMENT_UP	6	0.68	1.55	0.039	0.232	1	2	tags=33%, list=1%, signal=33%
SANA_TNF_SIGNALING_UP	10	0.59	1.61	0.04	0.191	1	76	tags=80%, list=32%, signal=113%
ANDERSEN_CHOLANGIOCARCINOMA_CLASS2	17	0.51	1.56	0.04	0.226	1	102	tags=82%, list=44%, signal=135%
HALLMARK_INTERFERON_GAMMA_RESPONSE	12	0.55	1.55	0.04	0.12	0.477	56	tags=58%, list=24%, signal=73%
GRAESSMANN_RESPONSE_TO_MC_AND_SERUM_DEPRIVATION_UP	11	0.56	1.53	0.041	0.259	1	80	tags=73%, list=34%, signal=105%
LEE_LIVER_CANCER_ACOX1_UP	6	0.67	1.56	0.042	0.228	1	45	tags=67%, list=19%, signal=80%
REACTOME_POST_TRANSLATIONAL_PROTEIN_MODIFICATION	25	0.47	1.58	0.043	0.21	1	71	tags=52%, list=30%, signal=67%
WP_IL18_SIGNALING_PATHWAY	14	0.53	1.55	0.043	0.229	1	29	tags=36%, list=12%, signal=38%
SATO_SILENCED_BY_METHYLATION_IN_PANCREATIC_CANCER_1	23	0.48	1.59	0.044	0.208	1	56	tags=48%, list=24%, signal=57%
HOSHIDA_LIVER_CANCER_SUBCLASS_S3	10	0.56	1.53	0.044	0.258	1	45	tags=50%, list=19%, signal=59%
SMIRNOV_CIRCULATING_ENDOTHELIOCYTES_IN_CANCER_UP	11	0.55	1.49	0.044	0.282	1	26	tags=36%, list=11%, signal=39%
BILD_HRAS_ONCOGENIC_SIGNATURE	17	0.52	1.6	0.045	0.2	1	83	tags=71%, list=35%, signal=101%
JINESH_BLEBBISHIELD_TRANSFORMED_STEM_CELL_SPHERES_UP	13	0.53	1.54	0.045	0.246	1	99	tags=85%, list=42%, signal=139%
SWEET_KRAS_TARGETS_DN	5	0.69	1.51	0.045	0.268	1	75	tags=100%, list=32%, signal=144%
LENAOUR_DENDRITIC_CELL_MATURATION_UP	6	0.63	1.47	0.045	0.29	1	71	tags=83%, list=30%, signal=117%
HALLMARK_INFLAMMATORY_RESPONSE	14	0.53	1.55	0.045	0.138	0.471	43	tags=43%, list=18%, signal=49%
ZHOU_INFLAMMATORY_RESPONSE_FIMA_UP	20	0.48	1.52	0.046	0.262	1	69	tags=55%, list=29%, signal=71%
ROZANOV_MMP14_TARGETS_UP	11	0.54	1.52	0.046	0.261	1	56	tags=55%, list=24%, signal=68%
MIR3119	7	0.63	1.52	0.046	1	1	46	tags=57%, list=20%, signal=69%
KEGG_HEMATOPOIETIC_CELL_LINEAGE	9	0.61	1.56	0.047	0.229	1	43	tags=56%, list=18%, signal=65%
GERY_CEBP_TARGETS	7	0.65	1.54	0.047	0.24	1	24	tags=43%, list=10%, signal=46%
DAZARD_RESPONSE_TO_UV_SCC_UP	6	0.66	1.52	0.047	0.26	1	69	tags=83%, list=29%, signal=115%
ZHONG_SECRETOME_OF_LUNG_CANCER_AND_MACROPHAGE	5	0.69	1.51	0.047	0.272	1	14	tags=40%, list=6%, signal=42%
DLX2_TARGET_GENES	7	0.63	1.54	0.047	1	1	24	tags=43%, list=10%, signal=46%
ICHIBA_GRAFT_VERSUS_HOST_DISEASE_D7_UP	7	0.63	1.49	0.049	0.274	1	17	tags=43%, list=7%, signal=45%

Supplementary Table 7B: Downregulated Gene Set Enrichment Analysis for significant genes between the control vs iAs+ with nominal p-value < 0.05.

GS	Size	ES	NES	NOM p-val	FDR q-val	FWER p-val	Rank at Max	Leading Edge
BLANCO_MELO_COVID19_BRONCHIAL_EPITHELIAL_CELLS_SARS_COV_2_INFECTION_DN	5	-0.83	-2.05	0	0.307	0.404	45	tags=100%, list=19%, signal=121%
YTATTTTNR_MEF2_02	13	-0.58	-2.25	0	0.089	0.054	24	tags=46%, list=10%, signal=49%
AAAYRNCTG_UNKNOWN	8	-0.68	-2.09	0	0.231	0.248	23	tags=63%, list=10%, signal=67%
HNF1_C	7	-0.71	-2.09	0	0.16	0.256	20	tags=57%, list=9%, signal=61%
YCATTAA_UNKNOWN	14	-0.54	-2.07	0	0.146	0.295	11	tags=36%, list=5%, signal=35%
GGATTA_PITX2_Q2	9	-0.64	-2.01	0	0.182	0.413	8	tags=33%, list=3%, signal=33%
GATA4_Q3	5	-0.81	-2	0	0.168	0.437	24	tags=80%, list=10%, signal=87%
GATA_Q6	6	-0.72	-1.95	0.003	0.194	0.596	24	tags=67%, list=10%, signal=72%
MEF2_02	5	-0.7	-1.76	0.003	0.392	0.975	32	tags=80%, list=14%, signal=91%
E4BP4_01	5	-0.7	-1.71	0.003	0.435	0.993	15	tags=60%, list=6%, signal=63%
PCGF2_TARGET_GENES	11	-0.57	-1.97	0.004	0.179	0.518	56	tags=73%, list=24%, signal=91%
CEBP_01	7	-0.65	-1.9	0.004	0.262	0.757	15	tags=43%, list=6%, signal=44%
CTTTAAR_UNKNOWN	19	-0.4	-1.8	0.006	0.351	0.938	57	tags=58%, list=24%, signal=70%
PECE_MAMMARY_STEM_CELL_DN	7	-0.61	-1.79	0.007	1	0.972	97	tags=100%, list=41%, signal=166%
MIR3646	10	-0.57	-1.89	0.008	0.243	0.771	17	tags=50%, list=7%, signal=52%
ZNF843_TARGET_GENES	14	-0.47	-1.83	0.012	0.314	0.902	56	tags=64%, list=24%, signal=79%
CONCANNON_APOPTOSIS_BY_EPOXOMICIN_DN	5	-0.69	-1.75	0.015	0.85	0.986	45	tags=80%, list=19%, signal=97%
EVI1_02	5	-0.66	-1.67	0.015	0.544	1	24	tags=60%, list=10%, signal=65%
AAGCAAT_MIR137	8	-0.63	-1.89	0.017	0.226	0.779	31	tags=63%, list=13%, signal=70%
P53_02	8	-0.56	-1.73	0.018	0.42	0.99	21	tags=50%, list=9%, signal=53%
STAT4_01	6	-0.65	-1.77	0.02	0.398	0.967	56	tags=83%, list=24%, signal=107%
RTAAACA_FREAC2_01	12	-0.5	-1.75	0.021	0.387	0.98	30	tags=50%, list=13%, signal=54%
PEDERSEN_METASTASIS_BY_ERBB2_ISOFORM_7	12	-0.42	-1.56	0.023	1	1	142	tags=100%, list=61%, signal=241%
TAATTA_CHX10_01	15	-0.38	-1.56	0.023	0.633	1	32	tags=47%, list=14%, signal=51%
HNF1_01	8	-0.54	-1.66	0.028	0.552	1	22	tags=50%, list=9%, signal=53%
REACTOME_NEURONAL_SYSTEM	6	-0.61	-1.66	0.032	1	0.999	28	tags=50%, list=12%, signal=55%
REACTOME_METABOLISM_OF_LIPIDS	15	-0.4	-1.57	0.032	1	1	147	tags=100%, list=63%, signal=252%
CEBPA_01	6	-0.6	-1.62	0.034	0.62	1	32	tags=50%, list=14%, signal=56%
WGGAATGY_TEF1_Q6	11	-0.46	-1.6	0.037	0.611	1	21	tags=36%, list=9%, signal=38%
MIR3934_3P	6	-0.58	-1.58	0.037	0.636	1	102	tags=100%, list=44%, signal=173%
MIR8068	6	-0.58	-1.6	0.039	0.625	1	102	tags=100%, list=44%, signal=173%

YATTNATC_UNKNOWN	8	-0.52	-1.62	0.042	0.599	1	15	tags=38%, list=6%, signal=39%
PAX7_TARGET_GENES	6	-0.56	-1.53	0.042	0.649	1	19	tags=33%, list=8%, signal=35%
ARID5B_TARGET_GENES	6	-0.58	-1.58	0.043	0.616	1	103	tags=100%, list=44%, signal=174%

Supplementary Table 8: Ingenuity Pathway Analysis performed on 234 genes from Supplementary Table 4.

© 2000-2022 QIAGEN. All rights reserved.

Ingenuity Canonical Pathways	-log(p-value)	Ratio	Molecules
EIF2 Signaling	16.7	0.159	ACTB,CCND1,EIF3C,EIF4A1,EIF4G2,PPP1CA,RNA18SN5,RNA28SN5,RNA5-8SN5,RPL17,RPL28,RPL3,RPL30,RPL31,RPL35,RPL38,RPL7,RPL8,RPLP0,RPLP1,RPLP2,RPS11,RPS12,RPS15,RPS17,RPS18,RPS19,RPS2,RPS20,RPS24,RPS25,RPS26,RPSA,UBA52,VEGFA,WARS1
Phagosome Maturation	10.9	0.152	ATP6V0C,ATP6V0D1,ATP6V0E1,ATP6V1E1,CTSB,CTSE,CTSZ,DYNC1H1,DYNLL1,DYNLRB1,HLA-A,HLA-B,HLA-C,HLA-DRB1,PRDX1,PRDX2,RAB5C,RAB7A,TUBA1A,TUBA1B,TUBA1C,TUBA4A,TUBB,TUBB4B
Ferroptosis Signaling Pathway	10.9	0.167	ARF1,ARF4,CBS/LOC102724560,CDKN1A,CTSB,EMP1,FTH1,FTL,H2AC18/H2AC19,H2AX,H2AZ1,H2BC12,HMOX1,HSPB1,NFE2L2,PRKAG2,SAT1,SLC3A2,SLC7A11,STEAP3,TP53,TXNRD1
mTOR Signaling	10.3	0.126	CDC42,EIF3C,EIF4A1,EIF4B,EIF4G2,HIF1A,HMOX1,PPP2R2A,PRKAG2,RHOB,RHOC,RHOD,RHOF,RNA18SN5,RPS11,RPS12,RPS15,RPS17,RPS18,RPS19,RPS2,RPS20,RPS24,RPS25,RPS26,RPSA,VEGFA
RHOGDI Signaling	10	0.123	ACTB,ARHGAP1,ARHGDIA,CD44,CDC42,CDH16,CDH6,CFL1,EZR,GNAI1,GNAI2,GNB1,ITGA3,ITGB1,ITGB3,ITGB4,ITGB8,MSN,MYH10,MYL12A,MYL12B,MYL6,RHOB,RHOC,RHOD,RHOF,WASF2
Regulation of eIF4 and p70S6K Signaling	8.92	0.127	EIF3C,EIF4A1,EIF4G2,ITGA3,ITGB1,ITGB3,ITGB4,ITGB8,PAIP2,PPP2R2A,RNA18SN5,RPS11,RPS12,RPS15,RPS17,RPS18,RPS19,RPS2,RPS20,RPS24,RPS25,RPS26,RPSA
Coronavirus Pathogenesis Pathway	8.59	0.118	ADAM9,CCND1,CXCL8,EEF1A1,HIF1A,IL6,NFKBIA,RAB7A,RNA18SN5,RPS11,RPS12,RPS15,RPS17,RPS18,RPS19,RPS2,RPS20,RPS24,RPS25,RPS26,RPSA,SIGMAR1,TGFBR2,TP53
Hepatic Fibrosis Signaling Pathway	8.57	0.0851	CALM1 (includes others),CCND1,CDC42,CXCL8,FOXO1,FTH1,FTL,GNAI1,GNAI2,HIF1A,IL18,IL1R1,ITGA3,ITGB1,ITGB3,ITGB4,ITGB8,MYL12A,MYL12B,MYL6,MYLK,NFKBIA,PRKAG2,PRKAR1A,PTEN,RHOB,RHOC,RHOD,RHOF,SOD2,SPP1,TGFBR2,TIMP1,VEGFA,WNT7A,WNT9B
Regulation of Actin-based Motility by Rho	8.57	0.157	ACTB,ARHGDIA,CDC42,CFL1,ITGA3,ITGB1,ITGB3,ITGB4,ITGB8,MYL12A,MYL12B,MYL6,MYLK,PFN1,RHOB,RHOC,RHOD,RHOF
Sertoli Cell-Sertoli Cell Junction Signaling	8.51	0.117	ACTB,ACTN4,CDC42,CLDN1,CLDN10,CLDN16,ITGA3,ITGB1,ITGB3,ITGB4,ITGB8,NECTIN2,OCLN,PRKAG2,PRKAR1A,PTEN,TJP1,TUBA1A,TUBA1B,TUBA1C,TUBA4A,TUBB,TUBB4B,YBX3
ILK Signaling	8.03	0.114	ACTB,ACTN4,CCND1,CDC42,CFL1,HIF1A,ITGB1,ITGB3,ITGB4,ITGB8,KRT18,MYH10,MYL6,PPP2R2A,PTEN,RHOB,RHOC,RHOD,RHOF,TGFB1I1,TMSB10/TMSB4X,VEGFA,VIM
Sirtuin Signaling Pathway	7.87	0.0956	ATP5MC1,CXCL8,DUSP6,EPAS1,FOXO1,GABARAPL1,GLS,H1-5,HIF1A,MT-CYB,MT-ND1,NDUFB5,NDUFS5,NFE2L2,NQO1,PGAM1,PGK1,PPARGC1A,SLC2A1,SOD2,TP53,TUBA1A,TUBA1B,TUBA1C,TUBA4A,UCP2,VDAC1,VDAC3
Germ Cell-Sertoli Cell Junction Signaling	7.27	0.118	ACTB,ACTN4,CDC42,CFL1,IQGAP1,ITGA3,ITGB1,NECTIN2,RHOB,RHOC,RHOD,RHOF,TGFBR2,TJP1,TUBA1A,TUBA1B,TUBA1C,TUBA4A,TUBB,TUBB4B
Leukocyte Extravasation Signaling	7.01	0.109	ACTB,ACTN4,ARHGAP1,CD44,CDC42,CLDN1,CLDN10,CLDN16,CYBA,EDIL3,EZR,GNAI1,GNAI2,ITGA3,ITGB1,MMP14,MSN,MYL6,TIMP1,TIMP2,VAV3
Integrin Signaling	6.94	0.104	ACTB,ACTN4,ARF1,ARF4,CAV1,CDC42,ITGA3,ITGB1,ITGB3,ITGB4,ITGB8,MYL12A,MYL12B,MYLK,PFN1,PTEN,RHOB,RHOC,RHOD,RHOF,TSPAN1,TSPAN4
Signaling by Rho Family GTPases	6.92	0.0936	ACTB,CDC42,CDH16,CDH6,CFL1,EZR,GNAI1,GNAI2,GNB1,IQGAP1,ITGA3,ITGB1,ITGB3,ITGB4,ITGB8,MSN,MYL12A,MYL12B,MYL6,MYLK,RHOB,RHOC,RHOD,RHOF,VIM
Caveolar-mediated Endocytosis Signaling	6.9	0.173	ACTB,CAV1,CD55,HLA-A,HLA-B,HLA-C,ITGA3,ITGB1,ITGB3,ITGB4,ITGB8,PTPN1,RAB5C
Protein Kinase A Signaling	6.81	0.078	AKAP12,CALM1 (includes others),DUSP1,DUSP4,DUSP6,GNAI1,GNAI2,GNB1,GYS1,H1-5,ITPR3,KDELR1,KDELR3,MYH10,MYL12A,MYL12B,MYL6,MYLK,NFKBIA,PPP1CA,PRKAG2,PRKAR1A,PTEN,PTP4A1,PTPN1,PTPN3,PTPRM,PTPRU,SFN,TGFBR2,YWHAH,YWHAZ
Remodeling of Epithelial Adherens Junctions	6.5	0.176	ACTB,ACTN4,IQGAP1,MET,RAB5C,RAB7A,TUBA1A,TUBA1B,TUBA1C,TUBA4A,TUBB,TUBB4B
Estrogen Receptor Signaling	6.34	0.0758	ATP5MC1,CAV1,CCND1,CDKN1A,CFL1,CTBP2,FOXO1,GNAI1,GNAI2,GNB1,HIF1A,HNRNPD,HSP90AA1,HSP90AB1,MCU,MMP14,MT-CYB,MT-ND1,MYL12A,MYL12B,MYL6,NDUFB5,NDUFS5,PPARGC1A,PRKAG2,PRKAR1A,PTEN,RUNX2,SOD2,TP53,VEGFA
Semaphorin Signaling in Neurons	6.11	0.18	ARHGAP1,CDC42,CFL1,DPYSL2,ITGB1,MET,NRP1,RHOB,RHOC,RHOD,RHOF
Axonal Guidance Signaling	6.08	0.0688	ADAM9,ADAMTS1,BMP1,CDC42,CFL1,DPYSL2,GNAI1,GNAI2,GNB1,ITGA3,ITGB1,ITGB3,ITGB4,ITGB8,LNPEP,MET,MMP14,MYL12A,MYL12B,MYL6,NRP1,PFN1,PRKAG2,PRKAR1A,RHOD,SEMA4A,TUBA1A,TUBA1B,TUBA1C,TUBA4A,TUBB,TUBB4B,VEGFA,WNT7A,WNT9B
Actin Cytoskeleton Signaling	5.89	0.0902	ACTB,ACTN4,CDC42,CFL1,EZR,FGF18,IQGAP1,ITGA3,ITGB1,ITGB3,ITGB4,ITGB8,MSN,MYH10,MYL12A,MYL12B,MYL6,MYLK,PFN1,TMSB10/TMSB4X,VAV3,WASF2
NRF2-mediated Oxidative Stress Response	5.53	0.0886	ABCC4,ACTB,DNAJA1,DNAJB1,EPHX1,FOSL1,FTH1,FTL,GSR,HERPUD1,HMOX1,HSP90AA1,HSP90AB1,NFE2L2,NQO1,PRDX1,SOD2,TXN,TXNRD1,UBB,VCP
PI3K/AKT Signaling	5.5	0.095	CCND1,CDKN1A,FOXO1,GYS1,HSP90AA1,HSP90AB1,IL1R1,ITGA3,ITGB1,ITGB3,ITGB4,ITGB8,NFKBIA,PPP2R2A,PTEN,SFN,TP53,YWHAH,YWHAZ
Semaphorin Neuronal Repulsive Signaling Pathway	5.37	0.107	CD44,CFL1,DPYSL2,ITGA3,ITGB1,ITGB3,ITGB4,ITGB8,MYL12A,MYL12B,MYL6,NRP1,PRKAG2,PRKAR1A,TP53,VCAN
IL-8 Signaling	5.19	0.0905	CCND1,CCND3,CDC42,CSTB,CXCL1,CXCL8,GNAI1,GNAI2,GNB1,HMOX1,IQGAP1,ITGB3,MYL12B,NFKBIA,RHOB,RHOC,RHOD,RHOF,VEGFA
Iron homeostasis signaling pathway	5.18	0.109	ACO1,ATP6V0C,ATP6V0D1,ATP6V0E1,ATP6V1E1,BMP1,CP,EPAS1,FTH1,FTL,HIF1A,HMOX1,IL6,SKP1,STEAP3
Clathrin-mediated Endocytosis Signaling	5.13	0.0933	ACTB,APOL1,CDC42,CLU,FGF18,HSPA8,ITGB1,ITGB3,ITGB4,ITGB8,MET,MYO1E,RAB5C,RAB7A,UBA52,UBB,UBC,VEGFA
Hypoxia Signaling in the Cardiovascular System	5.13	0.145	HIF1A,HSP90AA1,HSP90AB1,NFKBIA,NQO1,P4HB,PTEN,TP53,UBE2L3,UBE2V1,VEGFA
Osteoarthritis Pathway	5.01	0.0847	ANXA2,ANXA5,CXCL8,EPAS1,FGF18,HIF1A,IL1R1,ITGA3,ITGB1,ITGB3,ITGB4,ITGB8,PPARGC1A,PRKAG2,PTH1R,PTHLH,RUNX2,SPP1,TGFBR2,VEGFA
Tight Junction Signaling	4.99	0.095	ACTB,CDC42,CLDN1,CLDN10,CLDN16,MYH10,MYL6,MYLK,NECTIN2,OCLN,PPP2R2A,PRKAG2,PRKAR1A,PTEN,TGFBR2,TJP1,YBX3
CLEAR Signaling Pathway	4.8	0.0772	ATP6V0C,ATP6V0D1,ATP6V0E1,ATP6V1E1,BLOC1S1,CTNS,CTSB,HIF1A,ITPR3,NEU1,PML,PPARGC1A,PPP2R2A,PRKAG2,PSAP,RAB7A,SFN,TGFBR2,TP53,VEGFA,YWHAH,YWHAZ
Role of Tissue Factor in Cancer	4.71	0.112	CDC42,CFL1,CSF1,CXCL1,CXCL8,F3,ITGA3,ITGB1,ITGB3,P4HB,PTEN,TP53,VEGFA
Epithelial Adherens Junction Signaling	4.5	0.0955	CDC42,CFL1,IQGAP1,MET,MYH10,MYL12A,MYL12B,NECTIN2,PPP2R2A,PRKAG2,PTEN,SFN,TGFBR2,YWHAH,YWHAZ
Glucocorticoid Receptor Signaling	4.41	0.0583	ACTB,ANXA1,ATP5MC1,CAV1,CDKN1A,CXCL3,CXCL8,DUSP1,HLA-A,HLA-B,HLA-C,HLA-DMA,HLA-DRB1,HSP90AA1,HSP90AB1,HSPA1A/HSPA1B,HSPA8,IL1R1,IL6,KRT18,KRT19,KRT7,KRT8,MT-CYB,MT-ND1,NDUFB5,NDUFS5,NFKBIA,POLR2C,PRKAG2,SLPI,SMARCC2,TGFBR2,YWHAH
Tumor Microenvironment Pathway	4.41	0.0894	CCND1,CD44,CSF1,CXCL8,FGF18,FOXO1,HIF1A,HLA-A,HLA-B,HLA-C,IL6,ITGB3,MMP14,SLC2A1,SPP1,VEGFA

Hereditary Breast Cancer Signaling	4.39	0.0986	ACTB,CCND1,CCND3,CDKN1A,H2AX,POLR2C,PTEN,RAD50,SFN,SMARCC2,TP53,UBA52,UBB,UBC
HIF1α Signaling	4.15	0.0817	CDKN1A,ELOC,HIF1A,HMOX1,HSP90AA1,HSPA1A/HSPA1B,HSPA8,IL6,MET,MMP14,PKM,RAN,SAT1,SLC2A1,TP53,VEGFA,VIM
Agranulocyte Adhesion and Diapedesis	4.1	0.081	ACTB,CLDN1,CLDN10,CLDN16,CXCL1,CXCL3,CXCL8,EZR,GNAI1,GNAI2,IL18,IL1R1,ITGB1,MMP14,MSN,MYH10,MYL6
Antigen Presentation Pathway	4.07	0.179	CD74,HLA-A,HLA-B,HLA-C,HLA-DMA,HLA-DRB1,TAPBP
BEX2 Signaling Pathway	4.07	0.122	CCND1,CDKN1A,HIF1A,LDB1,LGALS1,NFKBIA,PPP2R2A,PTEN,SPP1,VEGFA
Hepatic Fibrosis / Hepatic Stellate Cell Activation	3.99	0.0825	CSF1,CXCL3,CXCL8,ECE1,IFNGR2,IGFBP3,IL1R1,IL6,KLF6,MET,MYH10,MYL6,TGFBR2,TIMP1,TIMP2,VEGFA
Sumoylation Pathway	3.88	0.107	ARHGDIA,CDC42,CTBP2,NFKBIA,PML,RAN,RHOB,RHOC,RHOD,RHOF,TP53
Virus Entry via Endocytic Pathways	3.85	0.106	ACTB,CAV1,CD55,CDC42,HLA-A,HLA-B,HLA-C,ITGB1,ITGB3,ITGB4,ITGB8
Glioma Invasiveness Signaling	3.75	0.123	CD44,CDC42,ITGB3,RHOB,RHOC,RHOD,RHOF,TIMP1,TIMP2
RAR Activation	3.74	0.0784	ACTB,AKR1B1,AKR1C1/AKR1C2,AKR1C3,ALDH1A1,DUSP1,IGFBP3,NR2F2,PML,PPARGC1A,PRKAG2,PRKAR1A,PTEN,RDH10,SMARCC2,VEGFA
Crosstalk between Dendritic Cells and Natural Killer Cells	3.68	0.11	ACTB,FSCN1,HLA-A,HLA-B,HLA-C,HLA-DRB1,IL18,IL6,NECTIN2,TNFSF10
Coronavirus Replication Pathway	3.67	0.156	IFITM1,TUBA1A,TUBA1B,TUBA1C,TUBA4A,TUBB,TUBB4B
MicroRNA Biogenesis Signaling Pathway	3.64	0.0802	DDX17,DNAJB1,FUS,HMOX1,HNRNPA2B1,HNRNPD,HSP90AA1,HSP90AB1,HSPA8,ILF2,POLR2C,RAN,SRSF1,TP53,VEGFA
Protein Ubiquitination Pathway	3.62	0.0693	DNAJA1,DNAJB1,ELOC,HLA-A,HLA-B,HLA-C,HSP90AA1,HSP90AB1,HSPA1A/HSPA1B,HSPA8,HSPB1,PSMB2,PSMD8,SKP1,UBA52,UBB,UBC,UBE2L3,UBE2V1
Actin Nucleation by ARP-WASP Complex	3.61	0.108	CDC42,ITGA3,ITGB1,ITGB3,ITGB4,ITGB8,RHOB,RHOC,RHOD,RHOF
Mitochondrial Dysfunction	3.53	0.0819	ACO1,ATP5MC1,COX7A2L,CYB5R3,GSR,MT-CO1,MT-CYB,MT-ND1,NDUFB5,NDUFS5,SOD2,UCP2,VDAC1,VDAC3
Glioblastoma Multiforme Signaling	3.53	0.0819	CCND1,CCND3,CDC42,CDKN1A,FOXO1,ITPR3,PTEN,RHOB,RHOC,RHOD,RHOF,TP53,WNT7A,WNT9B
Molecular Mechanisms of Cancer	3.51	0.0583	BMP1,CCND1,CCND3,CDC42,CDKN1A,FOXO1,GNAI1,GNAI2,GNB1,HIF1A,ITGA3,ITGB1,ITGB3,ITGB4,ITGB8,NFKBIA,PRKAG2,PRKAR1A,RHOB,RHOC,RHOD,RHOF,TGFBR2,TP53,WNT7A,WNT9B
Renal Cell Carcinoma Signaling	3.49	0.114	CDC42,ELOC,HIF1A,MET,SLC2A1,UBA52,UBB,UBC,VEGFA
Cholesterol Biosynthesis I	3.47	0.308	CYP51A1,DHCR7,MSMO1,TM7SF2
Cholesterol Biosynthesis II (via 24,25-dihydrolanosterol)	3.47	0.308	CYP51A1,DHCR7,MSMO1,TM7SF2
Cholesterol Biosynthesis III (via Desmosterol)	3.47	0.308	CYP51A1,DHCR7,MSMO1,TM7SF2
Vitamin-C Transport	3.45	0.217	LRRC8A,SELENOT,SLC2A1,TXN,TXNRD1
Zymosterol Biosynthesis	3.4	0.5	CYP51A1,MSMO1,TM7SF2
PAK Signaling	3.4	0.094	CDC42,CFL1,ITGA3,ITGB1,ITGB3,ITGB4,ITGB8,MYL12A,MYL12B,MYL6,MYLK
PXR/RXR Activation	3.39	0.123	ALDH1A1,FOXO1,IL6,PPARGC1A,PRKAG2,PRKAR1A,SCD,UGT1A9 (includes others)
Gap Junction Signaling	3.38	0.0758	ACTB,CAV1,GNAI1,GNAI2,ITPR3,PRKAG2,PRKAR1A,SMARCC2,TJP1,TUBA1A,TUBA1B,TUBA1C,TUBA4A,TUBB,TUBB4B
AMPK Signaling	3.37	0.0702	ACTB,CCND1,CDKN1A,FOXO1,GNAI1,GNAI2,GNB1,GYS1,PPARGC1A,PPP2R2A,PRKAG2,PRKAR1A,RAB6A,RAB7A,RAB8A,SLC2A1,SMARCC2
Aryl Hydrocarbon Receptor Signaling	3.31	0.0818	AHR,ALDH1A1,CCND1,CCND3,CDKN1A,HSP90AA1,HSP90AB1,HSPB1,IL6,NFE2L2,NQO1,TGM2,TP53
BAG2 Signaling Pathway	3.29	0.107	ANXA2,CDKN1A,CTSB,HSP90AA1,HSPA1A/HSPA1B,HSPA8,PSMB2,PSMD8,TP53
Colorectal Cancer Metastasis Signaling	3.24	0.0664	CCND1,CDC42,GNAI1,GNAI2,GNB1,IL6,MMP14,PRKAG2,PRKAR1A,RHOB,RHOC,RHOD,RHOF,TGFBR2,TP53,VEGFA,WNT7A,WNT9B
Thioredoxin Pathway	3.17	0.429	SELENOT,TXN,TXNRD1
Wound Healing Signaling Pathway	3.17	0.0675	CXCL8,IFNGR2,IL18,IL1R1,IL6,ITGA3,ITGB1,ITGB4,LAMA3,LAMB3,LAMC1,LAMC2,NFKBIA,TGFBR2,TNFSF10,VEGFA,VIM
Glycolysis I	3.11	0.185	ALDOA,GAPDH,PGAM1,PGK1,PKM
Gluconeogenesis I	3.11	0.185	ALDOA,GAPDH,MDH2,PGAM1,PGK1
Regulation of Cellular Mechanics by Calpain Protease	3.1	0.101	ACTN4,CCND1,CCND3,EZR,ITGA3,ITGB1,ITGB3,ITGB4,ITGB8
14-3-3-mediated Signaling	3.09	0.0866	FOXO1,SFN,TUBA1A,TUBA1B,TUBA1C,TUBA4A,TUBB,TUBB4B,VIM,YWHAH,YWHAZ
Granulocyte Adhesion and Diapedesis	3.09	0.0741	CLDN1,CLDN10,CLDN16,CXCL1,CXCL3,CXCL8,EZR,GNAI1,GNAI2,IL18,IL1R1,ITGB1,MMP14,MSN
Immunogenic Cell Death Signaling Pathway	3.07	0.1	ANXA1,BCAP31,DAPK1,HSP90AA1,HSP90AB1,HSPA1A/HSPA1B,HSPA8,IFNGR2,IL6
Gαq Signaling	3.04	0.0765	CALM1 (includes others),CDC42,GNAI1,GNAI2,GNB1,GYS1,HMOX1,ITPR3,NFKBIA,RHOB,RHOC,RHOD,RHOF
PTEN Signaling	3.02	0.08	CCND1,CDC42,CDKN1A,FOXO1,ITGA3,ITGB1,ITGB3,ITGB4,ITGB8,PTEN,TGFBR2,YWHAH
ERK/MAPK Signaling	3.01	0.0698	DUSP1,DUSP4,DUSP6,HSPB1,ITGA3,ITGB1,ITGB3,ITGB4,ITGB8,PPP1CA,PPP2R2A,PRKAG2,PRKAR1A,YWHAH,YWHAZ
Cardiac Hypertrophy Signaling	3	0.0651	CALM1 (includes others),CDC42,GNAI1,GNAI2,GNB1,HSPB1,IL6,MYL12A,MYL12B,MYL6,PRKAG2,PRKAR1A,RHOB,RHOC,RHOD,RHOF,TGFBR2
NAD Signaling Pathway	2.99	0.0795	H1-5,H2BC12,HIF1A,NT5E,PARP14,POLR2C,PPARGC1A,PRKAG2,SOD2,TP53,TPCN1,VEGFA
Autophagy	2.99	0.0694	CALM1 (includes others),DAPK1,FOXO1,GABARAPL1,GORASP2,HIF1A,PPP2R2A,PRKAG2,PRKAR1A,PTEN,RAB7A,RIPK2,SLC3A2,TP53,VEGFA
Polyamine Regulation in Colon Cancer	2.98	0.121	EIF5A,OAZ1,ODC1,SAT1,SLC3A2,SRM,TP53
Macropinocytosis Signaling	2.93	0.105	ACTN4,CDC42,CSF1,ITGB1,ITGB3,ITGB4,ITGB8,MET
CDK5 Signaling	2.88	0.087	ITGA3,ITGB1,LAMA3,LAMB3,LAMC1,LAMC2,PPP1CA,PPP2R2A,PRKAG2,PRKAR1A
Multiple Sclerosis Signaling Pathway	2.87	0.0676	C3,CXCL1,CXCL8,DUSP1,DUSP6,HLA-A,HLA-B,HLA-C,HLA-DMA,HLA-DRB1,IFNGR2,IL18,IL6,PARP14,TNFSF10

Neuregulin Signaling	2.82	0.0855	ERBB4,ERRFI1,HSP90AA1,HSP90AB1,ITGA3,ITGB1,ITGB3,ITGB4,ITGB8,PTEN
p53 Signaling	2.81	0.0918	CCND1,CDKN1A,HIF1A,PERP,PML,PTEN,SFN,THBS1,TP53
HER-2 Signaling in Breast Cancer	2.77	0.0661	ARF1,ARF4,CCND1,CDC42,CDKN1A,COX7A2L,FOXO1,ITGB1,ITGB3,ITGB4,ITGB8,MT-CO1,NFKBIA,PTEN,TP53
Senescence Pathway	2.75	0.0602	CALM1 (includes others),CCND1,CCND3,CDKN1A,CXCL8,IL6,ITPR3,MCU,PML,PPP1CA,PPP2R2A,PTEN,RAD50,SAA1,SOD2,TGFBR2,TP53,ZFP36L1
MSP-RON Signaling In Cancer Cells Pathway	2.75	0.0786	CCND1,HIF1A,ITGB1,ITGB4,MET,NFKBIA,SFN,VEGFA,VIM,YWHAH,YWHAZ
Calcium Transport I	2.66	0.3	ANXA5,ATP2A2,ATP2B4
Inhibition of Angiogenesis by TSP1	2.65	0.147	HSPG2,TGFBR2,THBS1,TP53,VEGFA
RHOA Signaling	2.63	0.0806	ACTB,ARHGAP1,CFL1,EZR,MSN,MYL12A,MYL12B,MYL6,MYLK,PFN1
Macrophage Classical Activation Signaling Pathway	2.63	0.0688	CXCL8,GBP2,HIF1A,HLA-DMA,HLA-DRB1,IFNGR2,IL18,IL6,MT-CO1,NFKBIA,PARP14,PKM,TNFSF10
Role Of Osteoclasts In Rheumatoid Arthritis Signaling Pathway	2.61	0.0584	ADAM9,CALM1 (includes others),CCND1,CDC42,CSF1,FOXO1,IL18,IL1R1,ITGB3,MMP14,NFKBIA,PTPN1,RHOB,RHOC,RHOD,RHOF,SPP1,VAV3
Cell Cycle: G2/M DNA Damage Checkpoint Regulation	2.61	0.12	CDKN1A,SFN,SKP1,TP53,YWHAH,YWHAZ
CXCR4 Signaling	2.6	0.0714	CDC42,GNAI1,GNAI2,GNB1,ITPR3,MYL12A,MYL12B,MYL6,RHOB,RHOC,RHOD,RHOF
Androgen Signaling	2.58	0.071	CALM1 (includes others),CCND1,DNAJB1,GNAI1,GNAI2,GNB1,HSP90AA1,ITPR3,POLR2C,PRKAG2,PRKAR1A,TGFB1I1
Ribonucleotide Reductase Signaling Pathway	2.56	0.0706	ACTB,CCND1,CDKN1A,HIF1A,IL6,MET,PARP14,SKP1,SMARCC2,THBS1,TP53,VEGFA
Paxillin Signaling	2.54	0.0841	ACTB,ACTN4,ARF1,CDC42,ITGA3,ITGB1,ITGB3,ITGB4,ITGB8
Cellular Effects of Sildenafil (Viagra)	2.51	0.0733	ACTB,CALM1 (includes others),ITPR3,MYH10,MYL12A,MYL12B,MYL6,MYLK,PRKAG2,PRKAR1A,SLC4A11
Complement System	2.48	0.135	C3,CD55,CD59,CFB,CFI
Unfolded protein response	2.46	0.0889	DNAJA1,DNAJB1,HSPA1A/HSPA1B,HSPA8,NFE2L2,P4HB,VCP,XBP1
Natural Killer Cell Signaling	2.45	0.0657	CDC42,CFL1,HLA-A,HLA-B,HLA-C,HSPA1A/HSPA1B,HSPA8,IL18,ITGB1,NECTIN2,PVR,TNFSF10,VAV3
HGF Signaling	2.43	0.0758	CCND1,CDC42,CDKN1A,IL6,ITGA3,ITGB1,ITGB3,ITGB4,ITGB8,MET
Role of Osteoblasts, Osteoclasts and Chondrocytes in Rheumatoid Arthritis	2.41	0.0625	BMP1,CALM1 (includes others),CSF1,FOXO1,IL18,IL1R1,IL6,ITGB3,MMP14,NFKBIA,RUNX2,SPP1,WNT7A,WNT9B
ID1 Signaling Pathway	2.4	0.0647	BHLHA15,CAV1,CCND1,CDKN1A,HIF1A,IL6,PTEN,RHOC,TGFBR2,TGM2,TP53,VEGFA,VIM
Thrombin Signaling	2.39	0.0622	ARHGEF38,CDC42,GNAI1,GNAI2,GNB1,ITPR3,MYL12A,MYL12B,MYL6,MYLK,RHOB,RHOC,RHOD,RHOF
CSDE1 Signaling Pathway	2.36	0.107	CCND3,FUS,HNRNPC,HNRNPD,PTEN,VIM
Bladder Cancer Signaling	2.31	0.0776	CCND1,CDKN1A,CXCL8,DAPK1,FGF18,MMP14,THBS1,TP53,VEGFA
Oxytocin Signaling Pathway	2.26	0.0567	ATP2B4,CALM1 (includes others),CXCL8,GNAI1,GNAI2,GNB1,HSPB1,IL6,ITPR3,MYH10,MYL6,MYLK,NFE2L2,PRKAG2,PRKAR1A,SMARCC2
HOTAIR Regulatory Pathway	2.24	0.0675	CD44,CDKN1A,MET,MMP14,NFKBIA,PTEN,RHOC,SPP1,VIM,WNT7A,WNT9B
VDR/RXR Activation	2.24	0.0897	CDKN1A,FOXO1,HSD17B2,IGFBP3,RUNX2,SERPINB1,SPP1
Retinoate Biosynthesis I	2.24	0.119	AKR1B1,AKR1C1/AKR1C2,AKR1C3,ALDH1A1,RDH10
Role of IL-17A in Psoriasis	2.22	0.214	CXCL1,CXCL3,CXCL8
Nitric Oxide Signaling in the Cardiovascular System	2.21	0.075	ATP2A2,CALM1 (includes others),CAV1,HSP90AA1,HSP90AB1,ITPR3,PRKAG2,PRKAR1A,VEGFA
Apelin Cardiomyocyte Signaling Pathway	2.21	0.0808	ATP2A2,GNAI1,GNAI2,HIF1A,MYL12A,MYL12B,MYL6,MYLK
HMGB1 Signaling	2.17	0.0659	CDC42,CXCL8,IFNGR2,IL18,IL1R1,IL6,RHOB,RHOC,RHOD,RHOF,TNFSF10
Production of Nitric Oxide and Reactive Oxygen Species in Macrophages	2.16	0.0628	APOL1,CDC42,CLU,CYBA,IFNGR2,NFKBIA,PPP1CA,PPP2R2A,RHOB,RHOC,RHOD,RHOF
Aldosterone Signaling in Epithelial Cells	2.15	0.0655	DNAJA1,DNAJB1,DUSP1,HSP90AA1,HSP90AB1,HSPA1A/HSPA1B,HSPA8,HSPB1,ICMT,ITPR3,SLC12A1
LXR/RXR Activation	2.15	0.0732	APOL1,C3,CLU,CYP51A1,IL18,IL1R1,IL6,SAA1,SCD
Neuroinflammation Signaling Pathway	2.13	0.0536	CXCL8,GLS,HLA-A,HLA-B,HLA-C,HLA-DMA,HLA-DRB1,HMOX1,IFNGR2,IL18,IL1R1,IL6,MFGE8,NFE2L2,SLC6A13,SOD2,TGFBR2
Estrogen Biosynthesis	2.11	0.111	AKR1C1/AKR1C2,AKR1C3,CYP51A1,HSD17B2,POR
Endocannabinoid Cancer Inhibition Pathway	2.11	0.068	CCND1,CCND3,CDKN1A,GNAI1,GNAI2,HIF1A,PRKAG2,PRKAR1A,VEGFA,VIM
Superpathway of Cholesterol Biosynthesis	2.1	0.138	CYP51A1,DHCR7,MSMO1,TM7SF2
Pulmonary Fibrosis Idiopathic Signaling Pathway	2.02	0.0521	ACTB,ADAMTS1,AXL,CAV1,CCND1,FGF18,FOXO1,IL6,ITGB1,MMP14,PTEN,TGFBR2,THBS1,TP53,VIM,WNT7A,WNT9B
HIPPO signaling	2.01	0.0814	CD44,PPP1CA,PPP2R2A,SFN,SKP1,YWHAH,YWHAZ
α-Adrenergic Signaling	1.99	0.0741	CALM1 (includes others),GNAI1,GNAI2,GNB1,GYS1,ITPR3,PRKAG2,PRKAR1A
Apelin Muscle Signaling Pathway	1.99	0.104	GNAI1,GNAI2,GNB1,PPARGC1A,PRKAG2
Xenobiotic Metabolism AHR Signaling Pathway	1.99	0.0805	AHR,ALDH1A1,HSP90AA1,HSP90AB1,IL6,NQO1,UGT1A9 (includes others)
Stearate Biosynthesis I (Animals)	1.98	0.0896	ACOT7,ACSL1,ELOVL1,MBOAT7,PORCN,SRD5A3
Atherosclerosis Signaling	1.97	0.0687	APOL1,CLU,CSF1,CXCL8,F3,IL18,IL6,PLAAT4,TNFRSF12A
Pentose Phosphate Pathway (Non-oxidative Branch)	1.97	0.333	TALDO1,TKT

RAN Signaling	1.97	0.176	CSE1L,KPNA2,RAN
Cell Cycle: G1/S Checkpoint Regulation	1.95	0.0882	CCND1,CCND3,CDKN1A,FOXO1,SKP1,TP53
eNOS Signaling	1.94	0.0641	CALM1 (includes others),CAV1,HSP90AA1,HSP90AB1,HSPA1A/HSPA1B,HSPA8,ITPR3,PRKAG2,PRKAR1A,VEGFA
FAT10 Cancer Signaling Pathway	1.92	0.1	EEF1A1,IL6,NFKBIA,TGFBR2,TP53
D-myo-inositol (1,4,5,6)-Tetrakisphosphate Biosynthesis	1.89	0.0601	ATP1A1,DUSP1,DUSP4,DUSP6,G6PC3,HACD2,PPP1CA,PPP2R2A,PTEN,PTPN1,PTPRM
D-myo-inositol (3,4,5,6)-tetrakisphosphate Biosynthesis	1.89	0.0601	ATP1A1,DUSP1,DUSP4,DUSP6,G6PC3,HACD2,PPP1CA,PPP2R2A,PTEN,PTPN1,PTPRM
Apelin Adipocyte Signaling Pathway	1.89	0.0769	GNAI1,GNAI2,GPX1,HIF1A,PPARGC1A,PRKAG2,PRKAR1A
SNARE Signaling Pathway	1.88	0.0662	CALM1 (includes others),HSPA8,MYH10,MYL6,PRKAG2,PRKAR1A,RAB6A,RAB7A,RAB8A
Prostate Cancer Signaling	1.86	0.0702	CCND1,CDKN1A,FOXO1,HSP90AA1,HSP90AB1,NFKBIA,PTEN,TP53
RAC Signaling	1.86	0.0657	CD44,CDC42,CFL1,IQGAP1,ITGA3,ITGB1,ITGB3,ITGB4,ITGB8
Acute Phase Response Signaling	1.85	0.0595	C3,CFB,CP,FTL,HMOX1,IL18,IL1R1,IL6,NFKBIA,SAA1,SOD2
Dopamine-DARPP32 Feedback in cAMP Signaling	1.85	0.0595	ATP2A2,CALM1 (includes others),GNAI1,GNAI2,ITPR3,KCNJ1,KCNJ16,PPP1CA,PPP2R2A,PRKAG2,PRKAR1A
Triacylglycerol Biosynthesis	1.85	0.0962	ELOVL1,LPCAT1,MBOAT7,PLPP2,PORCN
Ephrin B Signaling	1.84	0.0833	CDC42,CFL1,GNAI1,GNAI2,GNB1,VAV3
Aspartate Degradation II	1.84	0.286	GOT1,MDH2
Inhibition of ARE-Mediated mRNA Degradation Pathway	1.83	0.0617	PPP2R2A,PRKAG2,PRKAR1A,PSMB2,PSMD8,SFN,TNFSF10,YWHAH,YWHAZ,ZFP36L1
IL-17 Signaling	1.82	0.0588	CXCL1,CXCL3,CXCL8,HSP90AA1,HSP90AB1,IL18,IL6,LCN2,SRSF1,TNFSF10,VEGFA
Cardiac Hypertrophy Signaling (Enhanced)	1.79	0.0443	ATP2A2,CALM1 (includes others),CXCL8,FGF18,GNAI1,GNAI2,GNB1,HSPB1,IL18,IL1R1,IL6,ITGA3,ITGB1,ITGB3,ITGB4,ITGB8, ITPR3,PRKAG2,PRKAR1A,PTEN,TGFBR2,TNFSF10,WNT7A,WNT9B
Role Of Chondrocytes In Rheumatoid Arthritis Signaling Pathway	1.78	0.0638	CXCL8,IL18,IL1R1,IL6,ITGB1,MMP14,PRKAG2,RUNX2,VEGFA
Airway Pathology in Chronic Obstructive Pulmonary Disease	1.78	0.0678	CXCL1,CXCL3,CXCL8,FGF18,IL18,IL6,LCN2,TNFSF10
Interferon Signaling	1.77	0.111	IFI6,IFITM1,IFNGR2,MX1
IL-1 Signaling	1.77	0.0729	GNAI1,GNAI2,GNB1,IL1R1,NFKBIA,PRKAG2,PRKAR1A
Superoxide Radicals Degradation	1.72	0.25	NQO1,SOD2
Calcium Signaling	1.72	0.0548	ASPH,ATP2A2,ATP2B4,CALM1 (includes others),ITPR3,MCU,MYH10,MYL6,PRKAG2,PRKAR1A,TPM3,TPM4
3-phosphoinositide Degradation	1.7	0.0564	ATP1A1,DUSP1,DUSP4,DUSP6,G6PC3,HACD2,PPP1CA,PPP2R2A,PTEN,PTPN1,PTPRM
Toll-like Receptor Signaling	1.68	0.0769	IL18,NFKBIA,TNFAIP3,UBA52,UBB,UBC
Inhibition of Matrix Metalloproteases	1.66	0.103	HSPG2,MMP14,TIMP1,TIMP2
Pulmonary Healing Signaling Pathway	1.64	0.0553	CCND1,CDC42,CFL1,HIF1A,MMP14,NFKBIA,PRKAG2,THBS1,VEGFA,WNT7A,WNT9B
D-myo-inositol-5-phosphate Metabolism	1.64	0.0553	ATP1A1,DUSP1,DUSP4,DUSP6,G6PC3,HACD2,PPP1CA,PPP2R2A,PTEN,PTPN1,PTPRM
WNT/β-catenin Signaling	1.64	0.0575	CCND1,CD44,PPP2R2A,TGFBR2,TP53,UBA52,UBB,UBC,WNT7A,WNT9B
Role of BRCA1 in DNA Damage Response	1.63	0.075	ACTB,BABAM2,CDKN1A,RAD50,SMARCC2,TP53
FXR/RXR Activation	1.62	0.0635	APOL1,C3,CLU,FOXO1,G6PC3,IL18,PPARGC1A,SAA1
GADD45 Signaling	1.61	0.0833	CCND1,CCND3,CDKN1A,TGFBR2,TP53
Ephrin Receptor Signaling	1.6	0.0545	CDC42,CFL1,GNAI1,GNAI2,GNB1,ITGA3,ITGB1,ITGB3,ITGB4,ITGB8,VEGFA
IGF-1 Signaling	1.6	0.0673	FOXO1,IGFBP3,PRKAG2,PRKAR1A,SFN,YWHAH,YWHAZ
Huntington's Disease Signaling	1.58	0.0495	ATP5MC1,DNAJB1,GLS,GNB1,HSPA1A/HSPA1B,HSPA8,POLR2C,PSMB2,PSMD8,TGM2,TP53,UBA 52,UBB,UBC
G Beta Gamma Signaling	1.57	0.062	CAV1,CDC42,GNAI1,GNAI2,GNB1,ITPR3,PRKAG2,PRKAR1A
Thiamin Salvage III	1.56	1	TPK1
PD-1, PD-L1 cancer immunotherapy pathway	1.56	0.066	HLA-A,HLA-B,HLA-C,HLA-DMA,HLA-DRB1,IFNGR2,PTEN
Relaxin Signaling	1.55	0.0581	GNAI1,GNAI2,GNB1,NFKBIA,PRKAG2,PRKAR1A,RXFP1,SMARCC2,VEGFA
TR/RXR Activation	1.54	0.0714	AKR1C1/AKR1C2,AKR1C3,HIF1A,PPARGC1A,SLC2A1,UCP2
Chronic Myeloid Leukemia Signaling	1.54	0.0654	CCND1,CCND3,CDKN1A,CTBP2,MECOM,TGFBR2,TP53
Cyclins and Cell Cycle Regulation	1.52	0.0706	CCND1,CCND3,CDKN1A,PPP2R2A,SKP1,TP53
GABA Receptor Signaling	1.52	0.0606	GNAI1,GNAI2,GNB1,ITPR3,SLC6A13,UBA52,UBB,UBC
3-phosphoinositide Biosynthesis	1.51	0.0526	ATP1A1,DUSP1,DUSP4,DUSP6,G6PC3,HACD2,PPP1CA,PPP2R2A,PTEN,PTPN1,PTPRM
Gα12/13 Signaling	1.5	0.0602	CDC42,CDH16,CDH6,MYL12A,MYL12B,MYL6,NFKBIA,VAV3
Ovarian Cancer Signaling	1.5	0.057	CCND1,CD44,PRKAG2,PRKAR1A,PTEN,TP53,VEGFA,WNT7A,WNT9B
Oxidative Phosphorylation	1.46	0.0631	ATP5MC1,COX7A2L,MT-CO1,MT-CYB,MT-ND1,NDUFB5,NDUFS5
Role of PKR in Interferon Induction and Antiviral Response	1.45	0.0588	HSP90AA1,HSP90AB1,HSPA1A/HSPA1B,HSPA8,IFNGR2,IL18,NFKBIA,TP53

Role Of Osteoblasts In Rheumatoid Arthritis Signaling Pathway	1.45	0.05	CTSB,CTSE,CTSZ,CXCL8,IL18,IL6,MMP14,RUNX2,TNFSF10,VEGFA,WNT7A,WNT9B
Pentose Phosphate Pathway	1.45	0.182	TALDO1,TKT
Antioxidant Action of Vitamin C	1.45	0.0625	HMOX1,NFKBIA,PLAAT4,SELENOT,SLC2A1,TXN,TXNRD1
IL-17A Signaling in Airway Cells	1.42	0.0746	CXCL1,CXCL3,IL6,NFKBIA,PTEN
B Cell Development	1.42	0.087	HLA-A,HLA-B,HLA-DMA,HLA-DRB1
iNOS Signaling	1.39	0.0851	CALM1 (includes others),HMGA1,IFNGR2,NFKBIA
Agrin Interactions at Neuromuscular Junction	1.38	0.0725	ACTB,CDC42,ERBB4,ITGB1,LAMC1
PFKFB4 Signaling Pathway	1.37	0.0833	PRKAG2,PRKAR1A,TKT,TP53
Fcγ Receptor-mediated Phagocytosis in Macrophages and Monocytes	1.34	0.0638	ACTB,CDC42,EZR,HMOX1,PTEN,VAV3
Cholecystokinin/Gastrin-mediated Signaling	1.33	0.0588	CDC42,IL18,ITPR3,RHOB,RHOC,RHOD,RHOF
Oleate Biosynthesis II (Animals)	1.31	0.154	SCD,SCD5
Guanosine Nucleotides Degradation III	1.31	0.154	GDA,NT5E
IL-10 Signaling	1.31	0.0694	HMOX1,IL18,IL1R1,IL6,NFKBIA
Melatonin Signaling	1.31	0.0694	CALM1 (includes others),GNAI1,GNAI2,PRKAG2,PRKAR1A
Melanoma Signaling	1.31	0.08	CCND1,CDKN1A,PTEN,TP53
Sphingosine-1-phosphate Signaling	1.31	0.0583	CDC42,GNAI1,GNAI2,RHOB,RHOC,RHOD,RHOF
Death Receptor Signaling	1.3	0.0625	ACTB,HSPB1,NFKBIA,PARP14,TNFRSF21,TNFSF10
Th1 Pathway	1.28	0.0574	HLA-A,HLA-B,HLA-DMA,HLA-DRB1,IFNGR2,IL18,IL6
CDX Gastrointestinal Cancer Signaling Pathway	1.26	0.0495	BMP1,CCND1,CXCL8,IL18,IL6,KLF5,PTEN,TNFSF10,WNT7A,WNT9B
Asparagine Degradation I	1.26	0.5	ASRGL1
L-cysteine Degradation III	1.26	0.5	GOT1
Spermidine Biosynthesis I	1.26	0.5	SRM
Cysteine Biosynthesis/Homocysteine Degradation	1.26	0.5	CBS/LOC102724560
GDP-L-fucose Biosynthesis I (from GDP-D-mannose)	1.26	0.5	GFUS
Putrescine Biosynthesis III	1.26	0.5	ODC1
Glutamine Degradation I	1.26	0.5	GLS
VEGF Signaling	1.25	0.0606	ACTB,ACTN4,FOXO1,HIF1A,SFN,VEGFA
GPCR-Mediated Integration of Enteroendocrine Signaling Exemplified by an L Cell	1.25	0.0667	GNAI1,GNAI2,ITPR3,PRKAG2,PRKAR1A
Dilated Cardiomyopathy Signaling Pathway	1.25	0.0533	ACTB,ATP2A2,DSG2,ITPR3,MYH10,MYL6,PRKAG2,PRKAR1A
Methylglyoxal Degradation III	1.24	0.0938	AKR1B1,AKR1C1/AKR1C2,AKR1C3
Erythropoietin Signaling Pathway	1.24	0.0508	CCND1,CXCL8,HIF1A,IL18,IL6,ITPR3,NFKBIA,TNFSF10,TP53
Corticotropin Releasing Hormone Signaling	1.23	0.053	CALM1 (includes others),GNAI1,GNAI2,ITPR3,PRKAG2,PRKAR1A,SMARCC2,VEGFA
ATM Signaling	1.23	0.06	CDKN1A,H2AX,NFKBIA,PPP2R2A,RAD50,TP53
Pancreatic Adenocarcinoma Signaling	1.22	0.0556	CCND1,CDC42,CDKN1A,HMOX1,TGFBR2,TP53,VEGFA
Xenobiotic Metabolism Signaling	1.21	0.0447	AHR,ALDH1A1,CHST15,FTL,HMOX1,HSP90AA1,HSP90AB1,IL6,NFE2L2,NQO1,PPARGC1A,PPP2R2A,UGT1A9 (includes others)
Role of Cytokines in Mediating Communication between Immune Cells	1.21	0.0741	CXCL8,IL18,IL32,IL6
TREM1 Signaling	1.21	0.0649	CXCL3,CXCL8,IL18,IL6,ITGB1
GP6 Signaling Pathway	1.2	0.0551	CALM1 (includes others),ITGB3,LAMA3,LAMB3,LAMC1,LAMC2,VAV3
Cardiac β-adrenergic Signaling	1.2	0.05	AKAP12,ATP2A2,GNAI1,GNAI2,GNB1,PPP1CA,PPP2R2A,PRKAG2,PRKAR1A
Telomere Extension by Telomerase	1.2	0.133	HNRNPA2B1,RAD50
IL-7 Signaling Pathway	1.19	0.0641	CCND1,CCND3,FOXO1,MET,SLC2A1
IL-6 Signaling	1.19	0.0547	CXCL8,HSPB1,IL18,IL1R1,IL6,NFKBIA,VEGFA
Superpathway of Inositol Phosphate Compounds	1.17	0.0462	ATP1A1,DUSP1,DUSP4,DUSP6,G6PC3,HACD2,PPP1CA,PPP2R2A,PTEN,PTPN1,PTPRM
Thyroid Cancer Signaling	1.17	0.0633	CCND1,CXCL8,FOXO1,PTEN,TP53
Extrinsic Prothrombin Activation Pathway	1.15	0.125	F3,TFPI
fMLP Signaling in Neutrophils	1.15	0.0534	CALM1 (includes others),CDC42,GNAI1,GNAI2,GNB1,ITPR3,NFKBIA
Role of IL-17A in Arthritis	1.14	0.0702	CXCL1,CXCL3,CXCL8,NFKBIA
Role of CHK Proteins in Cell Cycle Checkpoint Control	1.12	0.069	CDKN1A,PPP2R2A,RAD50,TP53
PPAR Signaling	1.12	0.0561	HSP90AA1,HSP90AB1,IL18,IL1R1,NFKBIA,PPARGC1A

CCR3 Signaling in Eosinophils	1.11	0.0522	CALM1 (includes others),CFL1,GNAI1,GNAI2,GNB1,ITPR3,MYLK
NADH Repair	1.09	0.333	GAPDH
Serine Biosynthesis	1.09	0.333	PSAT1
1,25-dihydroxyvitamin D3 Biosynthesis	1.09	0.333	POR
Hypusine Biosynthesis	1.09	0.333	EIF5A
Glutamate Degradation II	1.09	0.333	GOT1
Aspartate Biosynthesis	1.09	0.333	GOT1
p70S6K Signaling	1.09	0.0519	BCAP31,GNAI1,GNAI2,PPP2R2A,SFN,YWHAH,YWHAZ
Adipogenesis pathway	1.09	0.0519	CTBP2,FOXO1,HIF1A,KLF5,NR2F2,TP53,XBP1
Xenobiotic Metabolism PXR Signaling Pathway	1.07	0.0469	ALDH1A1,CHST15,HSP90AA1,HSP90AB1,PPARGC1A,PPP1CA,PRKAG2,PRKAR1A,UGT1A9 (includes others)
Regulation Of The Epithelial Mesenchymal Transition By Growth Factors Pathway	1.07	0.0469	CDC42,FGF18,FOXO1,IL6,MET,OCLN,TGFBR2,TNFSF10,VIM
IL-17A Signaling in Fibroblasts	1.06	0.0789	IL6,LCN2,NFKBIA
Differential Regulation of Cytokine Production in Macrophages and T Helper Cells by IL-17A and IL-17F	1.06	0.111	CXCL1,IL6
Bile Acid Biosynthesis, Neutral Pathway	1.06	0.111	AKR1C1/AKR1C2,AKR1C3
Role of Hypercytokinemia/hyperchemokinemia in the Pathogenesis of Influenza	1.05	0.0581	CXCL3,CXCL8,IL18,IL6,MX1
Human Embryonic Stem Cell Pluripotency	1.05	0.0482	BMP1,FOXO1,GNAI1,GNAI2,GNB1,TGFBR2,WNT7A,WNT9B
Role of MAPK Signaling in Promoting the Pathogenesis of Influenza	1.04	0.0536	ATP6V0C,ATP6V0D1,ATP6V0E1,ATP6V1E1,NFKBIA,PLAAT4
PPARα/RXRα Activation	1.04	0.0462	HSP90AA1,HSP90AB1,IL1R1,IL6,NFKBIA,PPARGC1A,PRKAG2,PRKAR1A,TGFBR2
BMP signaling pathway	1.03	0.0575	BMP1,PRKAG2,PRKAR1A,RUNX2,SOSTDC1
Pathogen Induced Cytokine Storm Signaling Pathway	1.03	0.0403	C3,CXCL1,CXCL3,CXCL8,FTH1,FTL,HLA-DMA,HLA-DRB1,IFNGR2,IL18,IL1R1,IL6,SLC2A1,TNFSF10,VEGFA
Insulin Receptor Signaling	1.03	0.05	FOXO1,GYS1,PPP1CA,PRKAG2,PRKAR1A,PTEN,PTPN1
γ-linolenate Biosynthesis II (Animals)	1.02	0.105	ACSL1,CYB5R3
Purine Nucleotides Degradation II (Aerobic)	1.02	0.105	GDA,NT5E
DNA damage-induced 14-3-3σ Signaling	1.02	0.105	SFN,TP53
Sperm Motility	1.01	0.0431	AXL,CALM1 (includes others),ERBB4,GNAI1,GNAI2,GNB1,ITPR3,MET,PLAAT4,PRKAG2,PRKAR1A
Adrenomedullin signaling pathway	0.996	0.0452	C3,CALM1 (includes others),HIF1A,IL18,ITPR3,MYLK,PRKAG2,PRKAR1A,SMARCC2
Inflammasome pathway	0.983	0.1	CTSB,IL18
Th1 and Th2 Activation Pathway	0.979	0.0465	HLA-A,HLA-B,HLA-DMA,HLA-DRB1,IFNGR2,IL18,IL6,TGFBR2
Heme Degradation	0.975	0.25	HMOX1
Spermine and Spermidine Degradation I	0.975	0.25	SAT1
L-cysteine Degradation I	0.975	0.25	GOT1
PI3K Signaling in B Lymphocytes	0.971	0.0483	C3,CALM1 (includes others),CD81,ITPR3,NFKBIA,PTEN,VAV3
Glutamate Receptor Signaling	0.963	0.0606	CALM1 (includes others),GLS,GNB1,SLC1A1
Androgen Biosynthesis	0.947	0.0952	AKR1C3,SRD5A3
Mitotic Roles of Polo-Like Kinase	0.943	0.0597	HSP90AA1,HSP90AB1,PLK2,PPP2R2A
Pyroptosis Signaling Pathway	0.939	0.0538	GBP2,IL18,IL1R1,PRKAG2,PRKAR1A
IL-4 Signaling	0.939	0.0538	HLA-A,HLA-B,HLA-DMA,HLA-DRB1,HMGA1
p38 MAPK Signaling	0.939	0.05	DUSP1,HSPB1,IL18,IL1R1,TGFBR2,TP53
Neuroprotective Role of THOP1 in Alzheimer's Disease	0.924	0.0496	ECE1,HLA-A,HLA-B,HLA-C,PRKAG2,PRKAR1A
MIF Regulation of Innate Immunity	0.917	0.0682	CD74,NFKBIA,TP53
G Protein Signaling Mediated by Tubby	0.917	0.0682	GNAI1,GNAI2,GNB1
Putrescine Degradation III	0.914	0.0909	ALDH1A1,SAT1
SPINK1 General Cancer Pathway	0.91	0.058	IL6,MT1F,MT1M,MT1X
Small Cell Lung Cancer Signaling	0.896	0.0521	CCND1,CCND3,NFKBIA,PTEN,TP53
Glioma Signaling	0.889	0.0484	CALM1 (includes others),CCND1,CCND3,CDKN1A,PTEN,TP53
Creatine-phosphate Biosynthesis	0.883	0.2	CKMT1A/CKMT1B
Superpathway of Serine and Glycine Biosynthesis I	0.883	0.2	PSAT1
CMP-N-acetylneuraminate Biosynthesis I (Eukaryotes)	0.883	0.2	GNE
Glutathione Redox Reactions II	0.883	0.2	GSR

Tyrosine Degradation I	0.883	0.2	HGD
Acetate Conversion to Acetyl-CoA	0.883	0.2	ACSL1
Differential Regulation of Cytokine Production in Intestinal Epithelial Cells by IL-17A and IL-17F	0.879	0.087	CXCL1,LCN2
The Visual Cycle	0.879	0.087	AKR1C3,RDH10
TCA Cycle II (Eukaryotic)	0.879	0.087	ACO1,MDH2
Apelin Cardiac Fibroblast Signaling Pathway	0.879	0.087	IL6,PRKAG2
Gαs Signaling	0.876	0.048	GNAI1,GNAI2,GNB1,PRKAG2,PRKAR1A,PTH1R
Role of OCT4 in Mammalian Embryonic Stem Cell Pluripotency	0.873	0.0652	NR2F2,SPP1,TP53
Basal Cell Carcinoma Signaling	0.86	0.0556	BMP1,TP53,WNT7A,WNT9B
Role of IL-17F in Allergic Inflammatory Airway Diseases	0.854	0.0638	CXCL1,CXCL8,IL6
Ephrin A Signaling	0.854	0.0638	CDC42,CFL1,VAV3
Endocannabinoid Developing Neuron Pathway	0.854	0.0472	CCND1,GNAI1,GNAI2,GNB1,PRKAG2,PRKAR1A
Necroptosis Signaling Pathway	0.848	0.0449	AXL,DAPK1,TNFSF10,TP53,UBC,VDAC1,VDAC3
ERK5 Signaling	0.83	0.0541	FOSL1,SFN,YWHAH,YWHAZ
Bupropion Degradation	0.821	0.08	CYP51A1,POR
CDP-diacylglycerol Biosynthesis I	0.821	0.08	LPCAT1,MBOAT7
Ethanol Degradation IV	0.821	0.08	ACSL1,ALDH1A1
Granzyme A Signaling	0.815	0.0533	H1-5,MT-ND1,NDUFB5,NDUFS5
Rapoport-Luebering Glycolytic Shunt	0.81	0.167	PGAM1
Hepatic Cholestasis	0.796	0.0419	CXCL8,IL18,IL1R1,IL6,NFKBIA,PRKAG2,PRKAR1A,TNFSF10
GNRH Signaling	0.796	0.0419	CALM1 (includes others),CDC42,GNAI1,GNAI2,GNB1,ITPR3,PRKAG2,PRKAR1A
MYC Mediated Apoptosis Signaling	0.796	0.06	PRKAG2,PRKAR1A,TP53
IL-17A Signaling in Gastric Cells	0.793	0.0769	CXCL1,CXCL8
Estrogen-mediated S-phase Entry	0.793	0.0769	CCND1,CDKN1A
Role of NFAT in Cardiac Hypertrophy	0.788	0.0405	CALM1 (includes others),GNAI1,GNAI2,GNB1,IL6,ITPR3,PRKAG2,PRKAR1A,TGFBR2
P2Y Purigenic Receptor Signaling Pathway	0.788	0.0451	GNAI1,GNAI2,GNB1,ITGB3,PRKAG2,PRKAR1A
Role of MAPK Signaling in Inhibiting the Pathogenesis of Influenza	0.785	0.0519	CXCL8,IL6,NFKBIA,PLAAT4
TNFR1 Signaling	0.78	0.0588	CDC42,NFKBIA,TNFAIP3
NF-κB Activation by Viruses	0.772	0.0513	ITGA3,ITGB1,ITGB3,NFKBIA
Phosphatidylglycerol Biosynthesis II (Non-plastidic)	0.77	0.0741	LPCAT1,MBOAT7
Maturity Onset Diabetes of Young (MODY) Signaling	0.759	0.0506	APOL1,APOL2,GAPDH,UCP2
Ceramide Biosynthesis	0.75	0.143	SPTLC3
Glycogen Biosynthesis II (from UDP-D-Glucose)	0.75	0.143	GYS1
Telomerase Signaling	0.747	0.0463	CDKN1A,HSP90AA1,HSP90AB1,PPP2R2A,TP53
Oxytocin In Brain Signaling Pathway	0.745	0.0406	CALM1 (includes others),GNAI1,GNAI2,GNB1,IL6,ITPR3,PLAAT4,PTPN1
Glutathione Redox Reactions I	0.745	0.0714	GPX1,GSR
Chemokine Signaling	0.745	0.05	CALM1 (includes others),CFL1,GNAI1,GNAI2
Dopamine Receptor Signaling	0.745	0.05	PPP1CA,PPP2R2A,PRKAG2,PRKAR1A
Reelin Signaling in Neurons	0.738	0.0435	ARHGEF38,CDC42,CFL1,ITGA3,ITGB1,MAP1B
White Adipose Tissue Browning Pathway	0.738	0.0435	CTBP2,ITPR3,PPARGC1A,PRKAG2,PRKAR1A,VEGFA
Phototransduction Pathway	0.728	0.0556	GNB1,PRKAG2,PRKAR1A
Sonic Hedgehog Signaling	0.721	0.069	PRKAG2,PRKAR1A
Gαi Signaling	0.719	0.0429	CAV1,GNAI1,GNAI2,GNB1,PRKAG2,PRKAR1A
Apelin Endothelial Signaling Pathway	0.71	0.0426	CALM1 (includes others),GNAI1,GNAI2,GNB1,HIF1A,PRKAG2
PEDF Signaling	0.695	0.0476	NFKBIA,SOD2,TP53,WASF2
cAMP-mediated signaling	0.69	0.0383	AKAP12,CALM1 (includes others),DUSP1,DUSP4,DUSP6,GNAI1,GNAI2,PRKAR1A,PTH1R
Xenobiotic Metabolism General Signaling Pathway	0.69	0.042	AHR,FTL,HMOX1,NFE2L2,NQO1,UGT1A9 (includes others)
Role of Macrophages, Fibroblasts and Endothelial Cells in Rheumatoid Arthritis	0.678	0.0361	CALM1 (includes others),CCND1,CSF1,CXCL8,IL18,IL1R1,IL32,IL6,NFKBIA,VEGFA,WNT7A,WNT9B
Nicotine Degradation III	0.668	0.0517	CYP51A1,POR,UGT1A9 (includes others)

Cancer Drug Resistance By Drug Efflux	0.668	0.0517	FOXO1,PTEN,TP53
Regulation Of The Epithelial Mesenchymal Transition In Development Pathway	0.658	0.046	HIF1A,PYGO1,WNT7A,WNT9B
TNFR2 Signaling	0.656	0.0625	NFKBIA,TNFAIP3
Citrulline Biosynthesis	0.652	0.111	GLS
GPCR-Mediated Nutrient Sensing in Enteroendocrine Cells	0.642	0.0424	GNAI1,GNAI2,ITPR3,PRKAG2,PRKAR1A
Endometrial Cancer Signaling	0.638	0.05	CCND1,PTEN,TP53
PCP (Planar Cell Polarity) Pathway	0.638	0.05	PFN1,WNT7A,WNT9B
Endocannabinoid Neuronal Synapse Pathway	0.638	0.0403	GNAI1,GNAI2,GNB1,ITPR3,PRKAG2,PRKAR1A
Airway Inflammation in Asthma	0.636	0.0606	CXCL8,IFNGR2
Opioid Signaling Pathway	0.613	0.0358	CALM1 (includes others),CDC42,GNAI1,GNAI2,GNB1,ITPR3,NFKBIA,PRKAG2,PRKAR1A,SIGMAR1
Melatonin Degradation I	0.611	0.0484	CYP51A1,POR,UGT1A9 (includes others)
Coagulation System	0.599	0.0571	F3,TFPI
Ethanol Degradation II	0.599	0.0571	ACSL1,ALDH1A1
Synaptogenesis Signaling Pathway	0.592	0.0349	CALM1 (includes others),CDC42,CDH16,CDH6,CFL1,HSPA8,MAP1B,PRKAG2,PRKAR1A,RAB5C,THBS1
Role of Pattern Recognition Receptors in Recognition of Bacteria and Viruses	0.582	0.0385	C3,CXCL8,IL18,IL6,RIPK2,TNFSF10
Non-Small Cell Lung Cancer Signaling	0.582	0.0426	CCND1,CCND3,ITPR3,TP53
MIF-mediated Glucocorticoid Regulation	0.582	0.0556	CD74,NFKBIA
Sucrose Degradation V (Mammalian)	0.577	0.0909	ALDOA
Dolichyl-diphosphooligosaccharide Biosynthesis	0.577	0.0909	ALG3
ERB2-ERBB3 Signaling	0.573	0.0462	CCND1,FOXO1,PTEN
TWEAK Signaling	0.565	0.0541	NFKBIA,TNFRSF12A
TGF-β Signaling	0.562	0.0417	CDC42,RUNX2,TGFBR2,TGIF1
Role of PI3K/AKT Signaling in the Pathogenesis of Influenza	0.561	0.0455	GNAI1,GNAI2,NFKBIA
Nicotine Degradation II	0.561	0.0455	CYP51A1,POR,UGT1A9 (includes others)
Xenobiotic Metabolism CAR Signaling Pathway	0.558	0.0366	ALDH1A1,CHST15,HSP90AA1,HSP90AB1,PPARGC1A,PPP2R2A,UGT1A9 (includes others)
Phagosome Formation	0.551	0.0317	C3,CDC42,CFL1,CLEC4E,GPRC5A,HMOX1,ITGA3,ITGB1,ITGB3,ITGB4,ITGB8,ITPR3,MYH10,MYL12A,MYL12B,MYL6,MYLK,PLAAT4,PTH1R,RXFP1,VAV3,WASF2
Superpathway of Melatonin Degradation	0.55	0.0448	CYP51A1,POR,UGT1A9 (includes others)
Cell Cycle Regulation by BTG Family Proteins	0.548	0.0526	CCND1,PPP2R2A
Hematopoiesis from Multipotent Stem Cells	0.545	0.0833	CSF1
Neurovascular Coupling Signaling Pathway	0.541	0.0354	CALM1 (includes others),ITPR3,KCNJ1,KCNJ16,PLAAT4,PRKAG2,PRKAR1A,SMARCC2
Breast Cancer Regulation by Stathmin1	0.541	0.032	CCND1,CCND3,GNB1,GPRC5A,HIF1A,PPP1CA,PPP2R2A,PRKAG2,PRKAR1A,PTH1R,RXFP1,TP53,TUBA1A,TUBA1B,TUBA1C,TUBA4A,TUBB,TUBB4B,VEGFA
tRNA Charging	0.533	0.0513	GARS1,WARS1
Regulation of the Epithelial-Mesenchymal Transition Pathway	0.532	0.0359	FGF18,HIF1A,MET,PYGO1,TGFBR2,WNT7A,WNT9B
Synaptic Long Term Potentiation	0.529	0.0382	CALM1 (includes others),ITPR3,PPP1CA,PRKAG2,PRKAR1A
Superpathway of Methionine Degradation	0.519	0.05	CBS/LOC102724560,GOT1
Netrin Signaling	0.495	0.0417	ITPR3,PRKAG2,PRKAR1A
Circadian Rhythm Signaling	0.491	0.0336	CIRBP,GNAI1,GNAI2,GNB1,HIF1A,ITPR3,PRKAG2,PRKAR1A,SMARCC2
IL-12 Signaling and Production in Macrophages	0.491	0.0368	APOL1,CLU,IL18,NFKBIA,RAB7A
DNA Double-Strand Break Repair by Homologous Recombination	0.489	0.0714	RAD50
DNA Double-Strand Break Repair by Non-Homologous End Joining	0.489	0.0714	RAD50
Acyl-CoA Hydrolysis	0.489	0.0714	ACOT7
Urate Biosynthesis/Inosine 5'-phosphate Degradation	0.489	0.0714	NT5E
Phenylalanine Degradation IV (Mammalian, via Side Chain)	0.489	0.0714	GOT1
Colanic Acid Building Blocks Biosynthesis	0.489	0.0714	GFUS
Th2 Pathway	0.484	0.0365	HLA-A,HLA-B,HLA-DMA,HLA-DRB1,TGFBR2
CDC42 Signaling	0.479	0.0312	CDC42,CDC42SE1,CFL1,HLA-A,HLA-B,HLA-C,HLA-DMA,HLA-DRB1,IQGAP1,ITGA3,ITGB1,ITGB3,ITGB4,ITGB8,MYL12A,MYL12B,MYL6,MYLK
Acetone Degradation I (to Methylglyoxal)	0.476	0.0465	CYP51A1,POR
TEC Kinase Signaling	0.476	0.0311	ACTB,CDC42,GNAI1,GNAI2,GNB1,GTF2I,ITGA3,ITGB1,ITGB3,ITGB4,ITGB8,RHOB,RHOC,RHOD,RHOF,TNFRSF21,TNFSF10,VAV3
Fatty Acid Activation	0.465	0.0667	ACSL1

Superpathway of Citrulline Metabolism	0.465	0.0667	GLS
Choline Biosynthesis III	0.465	0.0667	HMOX1
BER (Base Excision Repair) Pathway	0.463	0.0455	ARL6IP5,TP53
Leptin Signaling in Obesity	0.456	0.0395	FOXO1,PRKAG2,PRKAR1A
Antiproliferative Role of Somatostatin Receptor 2	0.446	0.039	CDKN1A,GNB1,SMARCC2
Adenosine Nucleotides Degradation II	0.442	0.0625	NT5E
Apelin Pancreas Signaling Pathway	0.439	0.0435	PRKAG2,PRKAR1A
IL-23 Signaling Pathway	0.439	0.0435	HIF1A,NFKBIA
Kinetochore Metaphase Signaling Pathway	0.435	0.036	H2AC18/H2AC19,H2AX,H2AZ1,PPP1CA
Retinol Biosynthesis	0.427	0.0426	AKR1C3,RDH10
D-myo-inositol (1,3,4)-trisphosphate Biosynthesis	0.421	0.0588	PTEN
nNOS Signaling in Skeletal Muscle Cells	0.416	0.0417	CALM1 (includes others),ITPR3
Estrogen-Dependent Breast Cancer Signaling	0.411	0.037	AKR1C1/AKR1C2,CCND1,HSD17B2
JAK/STAT Signaling	0.403	0.0366	CDKN1A,IL6,PTPN1
Amyotrophic Lateral Sclerosis Signaling	0.399	0.0345	GPX1,RAB5C,TP53,VEGFA
Factors Promoting Cardiogenesis in Vertebrates	0.387	0.0329	BMP1,CCND1,TGFBR2,WNT7A,WNT9B
Glutaryl-CoA Degradation	0.384	0.0526	HACD2
Mitochondrial L-carnitine Shuttle Pathway	0.384	0.0526	ACSL1
Amyloid Processing	0.384	0.0392	PRKAG2,PRKAR1A
Type II Diabetes Mellitus Signaling	0.381	0.0327	ACSL1,ITPR3,NFKBIA,PKM,PRKAG2
Role of NANOG in Mammalian Embryonic Stem Cell Pluripotency	0.373	0.0333	BMP1,TP53,WNT7A,WNT9B
Endothelin-1 Signaling	0.359	0.0312	ECE1,GNAI1,GNAI2,HMOX1,ITPR3,PLAAT4
IL-15 Production	0.355	0.0325	AXL,ERBB4,IL6,MET
Histamine Degradation	0.352	0.0476	ALDH1A1
Fatty Acid α-oxidation	0.352	0.0476	ALDH1A1
Endoplasmic Reticulum Stress Pathway	0.352	0.0476	XBP1
Lymphotoxin β Receptor Signaling	0.346	0.0364	CXCL1,NFKBIA
Synaptic Long Term Depression	0.34	0.0306	GNAI1,GNAI2,ITPR3,PLAAT4,PPP2R2A,SMARCC2
Superpathway of D-myo-inositol (1,4,5)-trisphosphate Metabolism	0.337	0.0455	PTEN
FAT10 Signaling Pathway	0.337	0.0357	PSMB2,PSMD8
Oxidative Ethanol Degradation III	0.329	0.0351	ACSL1,ALDH1A1
Pyrimidine Deoxyribonucleotides De Novo Biosynthesis I	0.323	0.0435	CMPK1
G-Protein Coupled Receptor Signaling	0.323	0.0285	CALM1 (includes others),CCND1,CDC42,DUSP1,DUSP4,DUSP6,FOXO1,GNAI1,GNAI2,GNB1,GPRC5A,MYL12A,MYL12B,MYL6,MYLK,NFKBIA,PRKAG2,PRKAR1A,PTH1R,RXFP1
ERBB Signaling	0.322	0.0323	CDC42,ERBB4,FOXO1
MSP-RON Signaling Pathway	0.321	0.0345	ACTB,CSF1
Retinoic acid Mediated Apoptosis Signaling	0.305	0.0333	PARP14,TNFSF10
SPINK1 Pancreatic Cancer Pathway	0.305	0.0333	CTSB,TGFBR2
Role of JAK family kinases in IL-6-type Cytokine Signaling	0.298	0.04	IL6
STAT3 Pathway	0.289	0.0296	CDKN1A,IL1R1,TGFBR2,VEGFA
Role of JAK1, JAK2 and TYK2 in Interferon Signaling	0.287	0.0385	IFNGR2
Tryptophan Degradation III (Eukaryotic)	0.287	0.0385	HACD2
Neuropathic Pain Signaling In Dorsal Horn Neurons	0.275	0.0297	ITPR3,PRKAG2,PRKAR1A
NAD Salvage Pathway II	0.275	0.037	NT5E
Tryptophan Degradation X (Mammalian, via Tryptamine)	0.275	0.037	ALDH1A1
Pregnenolone Biosynthesis	0.275	0.037	MICAL2
Cysteine Biosynthesis III (mammalia)	0.275	0.037	CBS/LOC102724560
Activation of IRF by Cytosolic Pattern Recognition Receptors	0.268	0.0308	IL6,NFKBIA
Induction of Apoptosis by HIV1	0.268	0.0308	NFKBIA,TP53
Histidine Degradation VI	0.264	0.0357	MICAL2

CD40 Signaling	0.255	0.0299	NFKBIA,TNFAIP3
Phospholipases	0.255	0.0299	HMOX1,PLAAT4
Eicosanoid Signaling	0.249	0.0294	AKR1C3,PLAAT4
Role of p14/p19ARF in Tumor Suppression	0.245	0.0333	TP53
Ubiquinol-10 Biosynthesis (Eukaryotic)	0.237	0.0323	MICAL2
Serotonin Degradation	0.231	0.0282	ALDH1A1,UGT1A9 (includes others)
Dopamine Degradation	0.228	0.0312	ALDH1A1
4-1BB Signaling in T Lymphocytes	0.212	0.0294	NFKBIA
Role of JAK2 in Hormone-like Cytokine Signaling	0.212	0.0294	PTPN1
Oxytocin In Spinal Neurons Signaling Pathway	0.204	0.0286	SMARCC2
Fatty Acid β-oxidation I	0.204	0.0286	ACSL1
Nucleotide Excision Repair Pathway	0.204	0.0286	POLR2C
IL-13 Signaling Pathway	0	0.0259	AGR2,DUSP1,TP53
Fc Epsilon RI Signaling	0	0.00847	VAV3
LPS/IL-1 Mediated Inhibition of RXR Function	0	0.0276	ABCC4,ACSL1,ALDH1A1,CHST15,IL18,IL1R1,PPARGC1A
Ceramide Signaling	0	0.011	PPP2R2A
Role of RIG1-like Receptors in Antiviral Innate Immunity	0	0.0217	NFKBIA
Role of NFAT in Regulation of the Immune Response	0	0.0186	CALM1 (includes others),GNAI1,GNAI2,GNB1,HLA-A,HLA-B,HLA-DMA,HLA-DRB1,ITPR3,KPNA2,NFKBIA
FcγRIIB Signaling in B Lymphocytes	0	0.0115	ITPR3
LPS-stimulated MAPK Signaling	0	0.0235	CDC42,NFKBIA
CCR5 Signaling in Macrophages	0	0.01	CALM1 (includes others),GNAI1,GNAI2,GNB1,ITPR3
Calcium-induced T Lymphocyte Apoptosis	0	0.0152	ATP2A2,CALM1 (includes others),HLA-A,HLA-B,HLA-DMA,HLA-DRB1,ITPR3
Cytotoxic T Lymphocyte-mediated Apoptosis of Target Cells	0	0.00706	HLA-A,HLA-B,HLA-C
CD27 Signaling in Lymphocytes	0	0.0175	NFKBIA
IL-3 Signaling	0	0.0127	FOXO1
CTLA4 Signaling in Cytotoxic T Lymphocytes	0	0.0197	AHR,ARF1,HLA-A,HLA-B,HLA-C,HLA-DMA,HLA-DRB1,HMOX1,ITGA3,ITGB1,PPP2R2A,VAV3
B Cell Activating Factor Signaling	0	0.0233	NFKBIA
T Helper Cell Differentiation	0	0.017	HLA-A,HLA-B,HLA-DMA,HLA-DRB1,IFNGR2,IL18,IL6,TGFBR2
Oncostatin M Signaling	0	0.0233	EPAS1
CD28 Signaling in T Helper Cells	0	0.0155	CALM1 (includes others),CDC42,HLA-A,HLA-B,HLA-DMA,HLA-DRB1,ITPR3,NFKBIA
IL-15 Signaling	0	0.00189	CXCL8
Dendritic Cell Maturation	0	0.0168	FSCN1,HLA-A,HLA-B,HLA-C,HLA-DMA,HLA-DRB1,IL18,IL32,IL6,NFKBIA
Mechanisms of Viral Exit from Host Cells	0	0.0244	ACTB
Angiopoietin Signaling	0	0.0263	FOXO1,NFKBIA
Renin-Angiotensin Signaling	0	0.0248	ITPR3,PRKAG2,PRKAR1A
Docosahexaenoic Acid (DHA) Signaling	0	0.0263	FOXO1
ICOS-ICOSL Signaling in T Helper Cells	0	0.0158	CALM1 (includes others),HLA-A,HLA-B,HLA-DMA,HLA-DRB1,ITPR3,NFKBIA,PTEN
Lipid Antigen Presentation by CD1	0	0.00241	PSAP
Melanocyte Development and Pigmentation Signaling	0	0.0204	PRKAG2,PRKAR1A
DNA Methylation and Transcriptional Repression Signaling	0	0.0179	MBD2,TP53
Growth Hormone Signaling	0	0.0141	IGFBP3
CREB Signaling in Neurons	0	0.0215	CALM1 (includes others),GNAI1,GNAI2,GNB1,GPRC5A,ITPR3,POLR2C,PRKAG2,PRKAR1A,PTH1R,RXFP1,TGFBR2,VEGFA
Type I Diabetes Mellitus Signaling	0	0.0157	HLA-A,HLA-B,HLA-C,HLA-DMA,HLA-DRB1,IFNGR2,IL1R1,NFKBIA
Allograft Rejection Signaling	0	0.0102	HLA-A,HLA-B,HLA-C,HLA-DMA,HLA-DRB1
Autoimmune Thyroid Disease Signaling	0	0.0109	HLA-A,HLA-B,HLA-C,HLA-DMA,HLA-DRB1
Acute Myeloid Leukemia Signaling	0	0.022	CCND1,PML
Graft-versus-Host Disease Signaling	0	0.0157	HLA-A,HLA-B,HLA-C,HLA-DMA,HLA-DRB1,IL18,IL6
Communication between Innate and Adaptive Immune Cells	0	0.0075	CXCL8,HLA-A,HLA-B,HLA-C,HLA-DRB1,IL18,IL6
Systemic Lupus Erythematosus Signaling	0	0.0222	HLA-A,HLA-B,HLA-C,HNRNPA2B1,HNRNPC,IL18,IL6,RNU1-2,RNU1-27P,RNU1-28P,RNU1-3,RNU1-4,RNVU1-18,RNVU1-7

FAK Signaling	0	0.0144	CCND1,GPRC5A,IL1R1,ITGA3,ITGB1,ITGB3,ITGB4,ITGB8,MET,MMP14,PTEN,PTH1R,RXFP1,TGFBR2,TP53
April Mediated Signaling	0	0.0238	NFKBIA
Phospholipase C Signaling	0	0.0264	CALM1 (includes others),CDC42,GNB1,HMOX1,ITGA3,ITGB1,ITGB3,ITGB4,ITGB8,ITPR3,MYL12A,MYL12B,MYL6,RHOB,RHOC,RHOD,RHOF,TGM2
Altered T Cell and B Cell Signaling in Rheumatoid Arthritis	0	0.0162	CSF1,HLA-A,HLA-B,HLA-DMA,HLA-DRB1,IL18,IL6,SPP1
RANK Signaling in Osteoclasts	0	0.022	CALM1 (includes others),NFKBIA
Regulation of IL-2 Expression in Activated and Anergic T Lymphocytes	0	0.0108	CALM1 (includes others),NFKBIA,TGFBR2,TOB1,VAV3
Role of WNT/GSK-3β Signaling in the Pathogenesis of Influenza	0	0.0256	WNT7A,WNT9B
NUR77 Signaling in T Lymphocytes	0	0.0136	CALM1 (includes others),HLA-A,HLA-B,HLA-C,HLA-DMA,HLA-DRB1,TNFSF10
PKCθ Signaling in T Lymphocytes	0	0.0125	HLA-A,HLA-B,HLA-DMA,HLA-DRB1,ITPR3,NFKBIA,VAV3
Role of MAPK Signaling in the Pathogenesis of Influenza	0	0.0122	PLAAT4
Antiproliferative Role of TOB in T Cell Signaling	0	0.00704	SKP1,TGFBR2,TOB1
OX40 Signaling Pathway	0	0.0125	HLA-A,HLA-B,HLA-C,HLA-DMA,HLA-DRB1,NFKBIA
Assembly of RNA Polymerase II Complex	0	0.02	POLR2C
Spliceosomal Cycle	0	0.0204	HSPA8
NGF Signaling	0	0.0167	CDC42,TP53
Mouse Embryonic Stem Cell Pluripotency	0	0.00962	TP53
Hematopoiesis from Pluripotent Stem Cells	0	0.00674	CSF1,CXCL8,IL6
nNOS Signaling in Neurons	0	0.0213	CALM1 (includes others)
VEGF Family Ligand-Receptor Interactions	0	0.0238	NRP1,VEGFA
ERBB4 Signaling	0	0.0147	ERBB4
GDNF Family Ligand-Receptor Interactions	0	0.0263	CDC42,ITPR3
Heparan Sulfate Biosynthesis	0	0.0114	CHST15
Thyroid Hormone Metabolism II (via Conjugation and/or Degradation)	0	0.025	UGT1A9 (includes others)
Heparan Sulfate Biosynthesis (Late Stages)	0	0.0123	CHST15
Pyrimidine Ribonucleotides De Novo Biosynthesis	0	0.025	CMPK1
Pyridoxal 5'-phosphate Salvage Pathway	0	0.0154	DAPK1
Chondroitin Sulfate Biosynthesis	0	0.0172	CHST15
Dermatan Sulfate Biosynthesis	0	0.0167	CHST15
Dermatan Sulfate Biosynthesis (Late Stages)	0	0.0213	CHST15
Salvage Pathways of Pyrimidine Ribonucleotides	0	0.0206	CMPK1,DAPK1
Pyrimidine Ribonucleotides Interconversion	0	0.027	CMPK1
Chondroitin Sulfate Biosynthesis (Late Stages)	0	0.02	CHST15
Noradrenaline and Adrenaline Degradation	0	0.027	ALDH1A1
UVA-Induced MAPK Signaling	0	0.0204	PARP14,TP53
UVB-Induced MAPK Signaling	0	0.0192	TP53
UVC-Induced MAPK Signaling	0	0.0196	TP53
EGF Signaling	0	0.0182	ITPR3
SAPK/JNK Signaling	0	0.00797	CDC42,DUSP4,GNB1,TP53
FGF Signaling	0	0.0233	FGF18,MET
B Cell Receptor Signaling	0	0.011	CALM1 (includes others),CDC42,CFL1,FOXO1,NFKBIA,PTEN,VAV3
Neurotrophin/TRK Signaling	0	0.0128	CDC42
Apoptosis Signaling	0	0.0192	NFKBIA,TP53
NF-κB Signaling	0	0.0105	IL18,IL1R1,NFKBIA,TGFBR2,TNFAIP3,UBE2V1
T Cell Receptor Signaling	0	0.0179	CALM1 (includes others),DUSP6,HLA-A,HLA-B,HLA-C,HLA-DMA,HLA-DRB1,ITGA3,ITGB1,NFKBIA,VAV3
PDGF Signaling	0	0.0116	CAV1
Gustation Pathway	0	0.0246	ITPR3,LIPH,LRRC8A,PRKAG2,PRKAR1A
Th17 Activation Pathway	0	0.0124	AHR,HIF1A,HSP90AA1,HSP90AB1,IL1R1,IL6
NER (Nucleotide Excision Repair, Enhanced Pathway)	0	0.0192	GPS1,POLR2C
T Cell Exhaustion Signaling Pathway	0	0.0194	FOXO1,HLA-A,HLA-B,HLA-C,HLA-DMA,HLA-DRB1,IL6,MGAT5,PPP2R2A,TGFBR2,VEGFA

Systemic Lupus Erythematosus In T Cell Signaling Pathway	0	0.0264	CD44,CDC42,EZR,GNAI1,GNAI2,HLA-A,HLA-B,HLA-C,HLA-DMA,HLA-DRB1,IL6,MSN,PPP2R2A,RHOB,RHOC,RHOD,RHOF
Systemic Lupus Erythematosus In B Cell Signaling Pathway	0	0.0138	CALM1 (includes others),CCND1,CCND3,CXCL8,FOXO1,IFNGR2,IL18,IL6,PLAAT4,TNFSF10
Insulin Secretion Signaling Pathway	0	0.0257	EIF4A1,EIF4G2,ITPR3,PRKAG2,PRKAR1A,SEC61A1,SLC2A1
MSP-RON Signaling In Macrophages Pathway	0	0.0252	HLA-DMA,HLA-DRB1,IFNGR2
GM-CSF Signaling	0	0.0143	CCND1

Supplementary Table 9: Common differentially expressed genes between P2, P11, and the control.

Control vs P2 (-iAs)	Control vs P11 (-iAs	Common ContP2 vs ContP11	
**166 Total Genes**	**69 Total Genes**	**36 Common Genes**	
136 Genes Down	31 Genes Down	ANLN	**Down regulated: 11genes**
ACO2	ANLN	CLDN16	TMEM173
ACPP	CCDC146	CTSE	THBS1
ACSL1	CCDC84	CYFIP2	PTH1R
ADIPOR1	CCNL2	IGFBP3	LIX1
ANLN	CDH6	KCP	SCD5
ARHGAP24	CLDN16	LIX1	TXNIP
ATP6V1B1	CXCL1	LRRN4	CLDN16
B3GALT5	HERC2P9	MFAP5	ANLN
BAG6	ITGB3	NMNAT2	LRRN4
BCAM	KCP	PLK1	PLK1
BHLHE40	LENG8	PTH1R	KCP
CBS	LIX1	RNA5S1	
CCND1	LRRN4	RNA5S10	**UP regulated: 22 genes**
CDH16	MALT1	RNA5S11	RNA5S4
CEACAM5	MAP1B	RNA5S12	Y_RNA
CKMT1A	MYLK	RNA5S13	RNA5S12
CLCN5	NEAT1	RNA5S14	MFAP5
CLDN16	NPIPL3	RNA5S15	SH2D1B
CNPPD1	PILRB	RNA5S16	RNA5S15
CREG1	PLK1	RNA5S17	RNA5S2
CRIM1	PTH1R	RNA5S2	RNA5S13
CTSL	SCD5	RNA5S3	RNA5SP149
CYFIP2	SMA4	RNA5S4	SNORD36C
CYP51A1	SNORD41	RNA5S6	RNA5S3
DAG1	STAG3L1	RNA5S7	RNA5S1
DHCR7	STAG3L3	RNA5SP149	RNA5S10
EFHD1	THBS1	SCD5	RNA5S7
EMP1	TIA1	SH2D1B	RNA5S14
EPB41L1	TMEM173	SNORA22	SNORA22
ERMP1	TXNIP	SNORD36C	RNA5S17
ESRP1	UCHL1	THBS1	U2
ESRRG	WSB1	TMEM173	RNA5S11
FABP3		TXNIP	CTSE
FGF13	38 Genes Up	U2	RNA5S16
FGFBP1	CD68	Y_RNA	RNA5S6
FOLR1	CDKN1A		
FYB	CLDN10		Three genes changed between the conditions
GLB1	CTSE		IGFBP3

GOT1	CYFIP2		CYFIP2
GPI	GCNT3		NMNAT2
GPNMB	IGFBP3		
GPX3	LCN2		
GRN	LGALS3		
HACD3	LYPD1		
HOXA6	MFAP5		
HOXA7	MUC13		
ICAM1	NMNAT2		
IGFBP3	RDH10		
IL6	RNA5S1		
ITM2C	RNA5S10		
KCNJ1	RNA5S11		
KCP	RNA5S12		
KLK10	RNA5S13		
L1CAM	RNA5S14		
LDLR	RNA5S15		
LDOC1	RNA5S16		
LIX1	RNA5S17		
LRRC8A	RNA5S2		
LRRN4	RNA5S3		
LSR	RNA5S4		
MAL	RNA5S6		
MALL	RNA5S7		
MAN1A1	RNA5SP149		
MAOA	RPPH1		
MBOAT7	SH2D1B		
MPC1	SLC7A11		
MSMO1	SNORA22		
MT1JP	SNORA47		
MYD88	SNORD36C		
NMNAT2	ST6GALNAC1		
NQO1	U2		
NUCB1	UGT1A1		
OXA1L	Y_RNA		
P3H2			
P4HB			
PAQR5			
PDIA3P2			
PHLDA1			
PLAU			
PLK1			
PNPLA6			

PORCN
PPP1CC
PRKCSH
PTH1R
PVR
PVRL2
PXDN
QPCT
QSOX1
RAB17
RAP1GAP
RBM47
RELL1
S100A2
SAA1
SAA2
SCD
SCD5
SDC4
SEMA4A
SERINC2
SIAE
SLC12A1
SLC25A4
SLC35A4
SLC4A11
SLC6A13
SLC7A1
SLC7A5
SLC7A5P2
SNORD1A
SRPR
SUCLG1
SULF2
TACSTD2
TAPBP
TCN2
THBS1
TINAGL1
TM7SF2
TMEM127
TMEM171
TMEM173

TMEM184B
TMEM45B
TRIOBP
TTYH3
TUBA4A
TXNIP
UBA1
UGT8
USP11
VEPH1
WBP2
WFDC2
30 Genes Up
BNIP3P3
BNIP3P4
BNIP3P7
CTSE
MFAP5
RIMKLB
RNA5S1
RNA5S10
RNA5S11
RNA5S12
RNA5S13
RNA5S14
RNA5S15
RNA5S16
RNA5S17
RNA5S2
RNA5S3
RNA5S4
RNA5S6
RNA5S7
RNA5S9
RNA5SP149
RXFP1
SH2D1B
SLC15A1
SNORA22
SNORD36C
U1
U2

Y_RNA

Supplementary Table 10: Differentially expressed genes between iAs- and the control.

probe	FC_iASnegVSctrl	log2(FC)	raw.pval	#NAME?	Gene Symbol
TC1500006925.hg.1	0.39778	-1.33	1.40E-08	7.8536	THBS1
TC0500012107.hg.1	0.13483	-2.8908	8.33E-08	7.0794	CXCL14
TC0800009358.hg.1	0.1653	-2.5969	3.98E-07	6.3998	
TC0500012210.hg.1	0.49195	-1.0234	8.43E-07	6.0744	TMEM173
TC0700013623.hg.1	0.40809	-1.293	5.42E-06	5.2662	KCP
TC0600010057.hg.1	0.40279	-1.3119	7.87E-06	5.1041	ACAT2
TC2000008316.hg.1	0.3051	-1.7126	1.20E-05	4.9195	LRRN4
TC0700007814.hg.1	2.6008	1.379	2.41E-05	4.6175	SNORA22
TC0700007796.hg.1	2.4548	1.2956	3.08E-05	4.5112	SNORA22
TC1700008162.hg.1	2.0942	1.0664	3.79E-05	4.4216	
TC0100015598.hg.1	0.32936	-1.6023	4.03E-05	4.3945	TXNIP
TC1200011009.hg.1	2.4323	1.2823	4.42E-05	4.3541	
TC2000009317.hg.1	0.41179	-1.28	9.22E-05	4.0352	SULF2
TC0800007715.hg.1	0.28487	-1.8116	0.000108	3.9653	
TSUnmapped00000501.hg.1	2.7051	1.4357	0.000116	3.9369	
TC0300009853.hg.1	0.22216	-2.1703	0.000121	3.9171	CLDN16
TC0200010468.hg.1	2.0108	1.0077	0.000151	3.8221	
TC0300007273.hg.1	0.3251	-1.621	0.000157	3.8038	PTH1R
TC1000011646.hg.1	3.2108	1.6829	0.000174	3.7582	RNU2-59P
TC0500012184.hg.1	5.3906	2.4304	0.000218	3.6615	SNORD63
TC0400011180.hg.1	0.28467	-1.8126	0.000252	3.5978	SCD5
TC0500011528.hg.1	0.33644	-1.5716	0.000419	3.3774	LIX1
TC0100017064.hg.1	0.473	-1.0801	0.000444	3.3526	NUAK2
TC2000008315.hg.1	0.45394	-1.1394	0.000464	3.3334	
TC1400008851.hg.1	2.4391	1.2864	0.000533	3.2734	
TC0100015582.hg.1	2.1975	1.1359	0.000944	3.0253	U1
TC0300012212.hg.1	0.49046	-1.0278	0.000982	3.008	MYLK
TC1900009438.hg.1	2.1706	1.1181	0.001501	2.8237	
TC0700013427.hg.1	0.4451	-1.1678	0.001733	2.7613	STAG3L5P-PVRIG2P-PILRB
TC1500006574.hg.1	2.4603	1.2989	0.002258	2.6464	
TC0700012967.hg.1	2.1632	1.1132	0.002408	2.6184	RNY1
TC2100007424.hg.1	0.36466	-1.4554	0.00241	2.6181	
TC1700010767.hg.1	3.8412	1.9416	0.002593	2.5861	
TC1700010768.hg.1	3.8412	1.9416	0.002593	2.5861	
TC1700010769.hg.1	3.8412	1.9416	0.002593	2.5861	
TC1700010770.hg.1	3.8412	1.9416	0.002593	2.5861	
TC1700010771.hg.1	3.8412	1.9416	0.002593	2.5861	
TC1700010772.hg.1	3.8412	1.9416	0.002593	2.5861	
TC1700010773.hg.1	3.8412	1.9416	0.002593	2.5861	
TC1700010774.hg.1	3.8412	1.9416	0.002593	2.5861	
TC1700010775.hg.1	3.8412	1.9416	0.002593	2.5861	

TC1700010776.hg.1	3.8412	1.9416	0.002593	2.5861	
TC1700010778.hg.1	3.8412	1.9416	0.002593	2.5861	
TC1700010779.hg.1	3.8412	1.9416	0.002593	2.5861	
TC1200006520.hg.1	0.49341	-1.0191	0.002759	2.5593	TSPAN9
TC1800007655.hg.1	2.8116	1.4914	0.003378	2.4713	LOC100505817
TC2100008391.hg.1	0.46359	-1.1091	0.003819	2.418	
TC1700010777.hg.1	3.6726	1.8768	0.004008	2.397	
TC1500008988.hg.1	0.44016	-1.1839	0.005073	2.2947	GOLGA8A
TC1500008995.hg.1	0.45469	-1.137	0.005299	2.2758	GOLGA8B
TC1100011514.hg.1	0.48962	-1.0303	0.006646	2.1774	DHCR7
TC1200006909.hg.1	0.42117	-1.2475	0.007424	2.1294	EMP1
TC0600006762.hg.1	3.3055	1.7249	0.021114	1.6754	RNA5SP202
TC0600012117.hg.1	2.1354	1.0945	0.021306	1.6715	
TC0100017626.hg.1	3.5405	1.8239	0.021629	1.665	RNA5S2
TC0100017627.hg.1	3.5405	1.8239	0.021629	1.665	RNA5S3
TC0100017628.hg.1	3.5405	1.8239	0.021629	1.665	RNA5S4
TC0100017629.hg.1	3.5405	1.8239	0.021629	1.665	RNA5S1
TC0100017630.hg.1	3.5405	1.8239	0.021629	1.665	RNA5S6
TC0100017631.hg.1	3.5405	1.8239	0.021629	1.665	RNA5S11
TC0100017632.hg.1	3.5405	1.8239	0.021629	1.665	RNA5S1
TC0100017633.hg.1	3.5405	1.8239	0.021629	1.665	RNA5S10
TC0100017635.hg.1	3.5405	1.8239	0.021629	1.665	RNA5S1
TC0100017642.hg.1	3.5405	1.8239	0.021629	1.665	RNA5S1
TC0100017634.hg.1	2.9065	1.5393	0.028447	1.546	RNA5S9
TC0300013282.hg.1	4.3562	2.1231	0.035269	1.4526	RNA5SP149
TC0100017636.hg.1	3.9951	1.9982	0.035431	1.4506	RNA5S11
TC0100017637.hg.1	3.9951	1.9982	0.035431	1.4506	RNA5S12
TC0100017638.hg.1	3.9951	1.9982	0.035431	1.4506	RNA5S13
TC0100017639.hg.1	3.9951	1.9982	0.035431	1.4506	RNA5S14
TC0100017640.hg.1	3.9951	1.9982	0.035431	1.4506	RNA5S15
TC0100017641.hg.1	3.9951	1.9982	0.035431	1.4506	RNA5S16
TC0100009542.hg.1	0.46982	-1.0898	0.036866	1.4334	
TSUnmapped00000920.hg.1	4.2077	2.073	0.044735	1.3494	
TC0100006873.hg.1	2.2368	1.1614	0.047516	1.3232	RNU5E-1
TC1200009834.hg.1	8.0835	3.015	0.0511	1.2916	MFAP5
TC0800011956.hg.1	4.1177	2.0418	0.071119	1.148	MIR7641-1

Supplementary Table 11: Differentially expressed genes between HRTPT cells recovery to iAs passage (P2, P11) vs control.

	t.stat	p.value	#NAME?	FDR
TC1500006925.hg.1	29.264	1.40E-08	7.8536	3.50E-05
TC0500012107.hg.1	22.63	8.33E-08	7.0794	0.000104
TC0800009358.hg.1	18.035	3.98E-07	6.3998	0.000332
TC1100011833.hg.1	16.677	6.81E-07	6.1666	0.000421
TC0500012210.hg.1	16.167	8.43E-07	6.0744	0.000421
TC0700009597.hg.1	-14.443	1.82E-06	5.7404	0.000757
TC1200011591.hg.1	13.137	3.46E-06	5.4615	0.001234
TC0700013623.hg.1	12.289	5.42E-06	5.2662	0.001675
TC0700006567.hg.1	12.064	6.14E-06	5.2121	0.001675
TC0500007804.hg.1	11.906	6.70E-06	5.1737	0.001675
TC0600010057.hg.1	11.624	7.87E-06	5.1041	0.001688
TC0200006891.hg.1	11.573	8.10E-06	5.0913	0.001688
TC2000008316.hg.1	10.907	1.20E-05	4.9195	0.002314
TC0500011203.hg.1	-9.867	2.34E-05	4.6314	0.004019
TC0700007814.hg.1	-9.8193	2.41E-05	4.6175	0.004019
TC2200009230.hg.1	9.4814	3.04E-05	4.5177	0.00453
TC0700007796.hg.1	-9.4596	3.08E-05	4.5112	0.00453
TC1700008162.hg.1	-9.1656	3.79E-05	4.4216	0.005259
TC0100015598.hg.1	9.0783	4.03E-05	4.3945	0.005303
TC1200011009.hg.1	-8.9494	4.42E-05	4.3541	0.005529
TC0500013205.hg.1	8.7915	4.97E-05	4.3039	0.005911
HTA2.pos.PSR01009094.hg.1	8.6926	5.34E-05	4.2721	0.006071
TC2000008479.hg.1	-8.4822	6.26E-05	4.2034	0.006316
TC0700012684.hg.1	8.4662	6.34E-05	4.198	0.006316
TSUnmapped00000340.hg.1	-8.4189	6.57E-05	4.1824	0.006316
TSUnmapped00000845.hg.1	-8.4189	6.57E-05	4.1824	0.006316
TC1200007387.hg.1	-8.2193	7.67E-05	4.1154	0.007031
TC0800008314.hg.1	-8.1845	7.88E-05	4.1036	0.007031
TC2000009317.hg.1	7.9855	9.22E-05	4.0352	0.007891
HTA2.pos.PSR05015343.hg.1	7.9518	9.47E-05	4.0235	0.007891
TC0200013925.hg.1	7.8358	0.000104	3.9829	0.008386
TC0800007715.hg.1	7.7861	0.000108	3.9653	0.00846
TC0600012072.hg.1	7.7405	0.000112	3.9491	0.008499
TSUnmapped00000501.hg.1	-7.7065	0.000116	3.9369	0.008499
TC0300009853.hg.1	7.6513	0.000121	3.9171	0.008641
TC0400012903.hg.1	7.5335	0.000134	3.8745	0.009019
TC0400011580.hg.1	7.5135	0.000136	3.8673	0.009019
TC0400012236.hg.1	-7.5014	0.000137	3.8628	0.009019
TC0700013584.hg.1	7.448	0.000143	3.8433	0.009094

TC0500013047.hg.1	-7.4308	0.000146	3.8369	0.009094
TC0200010468.hg.1	-7.3906	0.000151	3.8221	0.009181
TC0300007273.hg.1	7.3411	0.000157	3.8038	0.009349
TC0300011815.hg.1	7.3096	0.000161	3.792	0.009381
TC0600008985.hg.1	-7.2826	0.000165	3.7819	0.009384
TC1000011646.hg.1	-7.2195	0.000174	3.7582	0.00969
TC0700006629.hg.1	7.172	0.000182	3.7403	0.009878
TC0X00006799.hg.1	7.1235	0.00019	3.7219	0.010087
TC0100012278.hg.1	7.0745	0.000198	3.7032	0.010311
TC1900011756.hg.1	7.0051	0.000211	3.6765	0.01074
TC0500012184.hg.1	-6.9661	0.000218	3.6615	0.010764
TC0900009489.hg.1	6.9464	0.000222	3.6538	0.010764
TC0500012579.hg.1	6.936	0.000224	3.6498	0.010764
TC2100007821.hg.1	-6.8797	0.000236	3.6278	0.010784
HTA2.pos.PSR17024046.hg.1	6.8706	0.000238	3.6243	0.010784
TC0X00009356.hg.1	6.8579	0.00024	3.6193	0.010784
TC0500008157.hg.1	6.8515	0.000242	3.6168	0.010784
TC0400011180.hg.1	6.8032	0.000252	3.5978	0.011069
TC1700010693.hg.1	6.7762	0.000259	3.5871	0.011149
HTA2.pos.3686585_st	6.7284	0.00027	3.5682	0.011323
TC0X00010001.hg.1	-6.7221	0.000272	3.5656	0.011323
TC0400011931.hg.1	6.5645	0.000315	3.5023	0.012886
TC0500011528.hg.1	6.2618	0.000419	3.3774	0.016902
TC0100017064.hg.1	6.2028	0.000444	3.3526	0.017363
TC1900008824.hg.1	6.2013	0.000445	3.352	0.017363
TC2000008315.hg.1	6.1576	0.000464	3.3334	0.017842
TC1400008851.hg.1	-6.0176	0.000533	3.2734	0.019739
TC0M00006432.hg.1	-6.0154	0.000534	3.2725	0.019739
HTA2.pos.PSR06035602.hg.1	6.0095	0.000537	3.2699	0.019739
HTA2.pos.PSR02024033.hg.1	5.9676	0.00056	3.2517	0.020285
HTA2.pos.JUC04005577.hg.1	-5.8885	0.000607	3.2172	0.021652
HTA2.pos.3686576_st	5.8279	0.000645	3.1905	0.0227
TC0400007933.hg.1	5.8074	0.000659	3.1814	0.022856
TC1700012437.hg.1	5.7628	0.000689	3.1616	0.023151
TC0500011233.hg.1	-5.7448	0.000702	3.1536	0.023151
HTA2.pos.3686574_st	5.744	0.000703	3.1532	0.023151
TC0200010410.hg.1	5.7422	0.000704	3.1524	0.023151
HTA2.pos.2839528_st	5.7184	0.000722	3.1417	0.023171
TC1400009398.hg.1	-5.7161	0.000723	3.1407	0.023171
TC0300013520.hg.1	5.6841	0.000748	3.1264	0.023647
TC1300009757.hg.1	-5.6247	0.000795	3.0996	0.024133
TC0300011792.hg.1	-5.6241	0.000796	3.0993	0.024133
TC1000009536.hg.1	5.6227	0.000797	3.0986	0.024133

TC1800007431.hg.1	5.6171	0.000802	3.0961	0.024133
TC0500010635.hg.1	5.5956	0.00082	3.0864	0.024195
TC1100013022.hg.1	5.5918	0.000823	3.0846	0.024195
TC1600007521.hg.1	5.5679	0.000844	3.0737	0.024326
TC0300012718.hg.1	5.5645	0.000847	3.0722	0.024326
TC0400011349.hg.1	5.4627	0.000943	3.0254	0.026213
TC0100015582.hg.1	-5.4623	0.000944	3.0253	0.026213
TC0100015568.hg.1	-5.4618	0.000944	3.025	0.026213
TC0300012212.hg.1	5.4251	0.000982	3.008	0.02696
TC0200010443.hg.1	5.3855	0.001024	2.9896	0.027745
HTA2.pos.PSR6_cox_hap2002783.hg.1	5.3333	0.001084	2.9651	0.027745
HTA2.pos.PSR6_dbb_hap3002696.hg.1	5.3333	0.001084	2.9651	0.027745
HTA2.pos.PSR6_mann_hap4002311.hg.1	5.3333	0.001084	2.9651	0.027745
HTA2.pos.PSR6_mcf_hap5002453.hg.1	5.3333	0.001084	2.9651	0.027745
HTA2.pos.PSR6_qbl_hap6002764.hg.1	5.3333	0.001084	2.9651	0.027745
TC1900008860.hg.1	5.2941	0.001131	2.9467	0.027745
TC0800006760.hg.1	5.282	0.001146	2.941	0.027745
TC1100012226.hg.1	-5.2739	0.001156	2.9372	0.027745
TC1400008160.hg.1	-5.2506	0.001185	2.9261	0.027745
TC0100015643.hg.1	-5.2489	0.001188	2.9253	0.027745
HTA2.pos.PSR06017433.hg.1	5.2234	0.001221	2.9132	0.027745
HTA2.pos.PSR6_apd_hap1001142.hg.1	5.2234	0.001221	2.9132	0.027745
HTA2.pos.PSR6_cox_hap2002778.hg.1	5.2234	0.001221	2.9132	0.027745
HTA2.pos.PSR6_dbb_hap3002691.hg.1	5.2234	0.001221	2.9132	0.027745
HTA2.pos.PSR6_mann_hap4002306.hg.1	5.2234	0.001221	2.9132	0.027745
HTA2.pos.PSR6_mcf_hap5002448.hg.1	5.2234	0.001221	2.9132	0.027745
HTA2.pos.PSR6_qbl_hap6002759.hg.1	5.2234	0.001221	2.9132	0.027745
HTA2.pos.PSR6_ssto_hap7002447.hg.1	5.2234	0.001221	2.9132	0.027745
TC0100013046.hg.1	-5.1313	0.001352	2.8691	0.029149
TC0100013054.hg.1	-5.1313	0.001352	2.8691	0.029149
TC0100015533.hg.1	-5.1313	0.001352	2.8691	0.029149
TC1_KI270713v1_random00006433.hg.1	-5.1313	0.001352	2.8691	0.029149
TC1400008912.hg.1	-5.1313	0.001352	2.8691	0.029149
HTA2.pos.3080378_st	5.1304	0.001353	2.8687	0.029149
TC0700013205.hg.1	-5.0925	0.001411	2.8504	0.029788
TC0600010357.hg.1	5.0914	0.001413	2.8499	0.029788
TC2000008055.hg.1	5.0834	0.001426	2.846	0.029788
TSUnmapped00000499.hg.1	5.0762	0.001437	2.8425	0.029788
TC2000006736.hg.1	5.0723	0.001443	2.8406	0.029788
TC0400010785.hg.1	5.0656	0.001454	2.8374	0.029788
HTA2.pos.PSR06035604.hg.1	5.0532	0.001475	2.8313	0.029959
TC1900009438.hg.1	-5.0375	0.001501	2.8237	0.030243
HTA2.pos.2928795_st	-5.0234	0.001525	2.8169	0.030479

HTA2.pos.3686554_st	5.0082	0.001551	2.8094	0.030759
HTA2.pos.PSR05013909.hg.1	-4.9797	0.001602	2.7955	0.031512
TC1300007794.hg.1	4.9504	0.001655	2.7811	0.032319
TC1000008056.hg.1	4.9371	0.001681	2.7745	0.032557
HTA2.pos.2928788_st	-4.9156	0.001722	2.7639	0.03305
TC0700013427.hg.1	4.9104	0.001733	2.7613	0.03305
HTA2.pos.PSR17024065.hg.1	4.8798	0.001794	2.7462	0.033965
HTA2.pos.PSR17024054.hg.1	4.8685	0.001817	2.7406	0.034147
TC1400008633.hg.1	-4.8343	0.00189	2.7235	0.035247
TC0M00006444.hg.1	-4.8148	0.001933	2.7138	0.035781
TC1200009724.hg.1	4.793	0.001982	2.7029	0.036405
HTA2.pos.PSR04018328.hg.1	4.7871	0.001996	2.6999	0.036405
TC1400007562.hg.1	4.7105	0.002182	2.6612	0.0379
TC1500006574.hg.1	-4.6813	0.002258	2.6464	0.0379
HTA2.pos.PSR15015201.hg.1	-4.6687	0.002291	2.64	0.0379
HTA2.pos.PSR17024053.hg.1	4.6591	0.002317	2.6351	0.0379
HTA2.pos.PSR06017442.hg.1	4.6427	0.002362	2.6267	0.0379
HTA2.pos.PSR6_cox_hap2002785.hg.1	4.6427	0.002362	2.6267	0.0379
HTA2.pos.PSR6_dbb_hap3002698.hg.1	4.6427	0.002362	2.6267	0.0379
HTA2.pos.PSR6_mann_hap4002313.hg.1	4.6427	0.002362	2.6267	0.0379
HTA2.pos.PSR6_mcf_hap5002455.hg.1	4.6427	0.002362	2.6267	0.0379
HTA2.pos.PSR6_qbl_hap6002766.hg.1	4.6427	0.002362	2.6267	0.0379
HTA2.pos.PSR6_ssto_hap7002454.hg.1	4.6427	0.002362	2.6267	0.0379
TC0700012967.hg.1	-4.6264	0.002408	2.6184	0.0379
TC2100007424.hg.1	4.6258	0.00241	2.6181	0.0379
TC0600009310.hg.1	4.6255	0.002411	2.6179	0.0379
HTA2.pos.PSR05013907.hg.1	-4.6205	0.002425	2.6153	0.0379
TC0100007075.hg.1	-4.6115	0.002451	2.6107	0.0379
TC0100007085.hg.1	-4.6115	0.002451	2.6107	0.0379
TC0100009762.hg.1	-4.6115	0.002451	2.6107	0.0379
TC1400006877.hg.1	-4.6115	0.002451	2.6107	0.0379
TC0400011902.hg.1	-4.6103	0.002454	2.6101	0.0379
TC1500006995.hg.1	4.6061	0.002466	2.608	0.0379
TC0400007344.hg.1	4.5667	0.002584	2.5877	0.0379
TC1700010767.hg.1	-4.5637	0.002593	2.5861	0.0379
TC1700010768.hg.1	-4.5637	0.002593	2.5861	0.0379
TC1700010769.hg.1	-4.5637	0.002593	2.5861	0.0379
TC1700010770.hg.1	-4.5637	0.002593	2.5861	0.0379
TC1700010771.hg.1	-4.5637	0.002593	2.5861	0.0379
TC1700010772.hg.1	-4.5637	0.002593	2.5861	0.0379
TC1700010773.hg.1	-4.5637	0.002593	2.5861	0.0379
TC1700010774.hg.1	-4.5637	0.002593	2.5861	0.0379
TC1700010775.hg.1	-4.5637	0.002593	2.5861	0.0379

TC1700010776.hg.1	-4.5637	0.002593	2.5861	0.0379
TC1700010778.hg.1	-4.5637	0.002593	2.5861	0.0379
TC1700010779.hg.1	-4.5637	0.002593	2.5861	0.0379
TC0600014276.hg.1	-4.53	0.0027	2.5687	0.038139
HTA2.pos.PSR6_cox_hap2002787.hg.1	4.5235	0.002721	2.5653	0.038139
HTA2.pos.PSR6_dbb_hap3002700.hg.1	4.5235	0.002721	2.5653	0.038139
HTA2.pos.PSR6_mann_hap4002315.hg.1	4.5235	0.002721	2.5653	0.038139
HTA2.pos.PSR6_mcf_hap5002457.hg.1	4.5235	0.002721	2.5653	0.038139
HTA2.pos.PSR6_qbl_hap6002768.hg.1	4.5235	0.002721	2.5653	0.038139
HTA2.pos.PSR6_ssto_hap7002456.hg.1	4.5235	0.002721	2.5653	0.038139
HTA2.pos.JUC04005592.hg.1	-4.5201	0.002732	2.5635	0.038139
TC1200006520.hg.1	4.5119	0.002759	2.5593	0.0383
HTA2.pos.PSR17024051.hg.1	4.5054	0.00278	2.5559	0.038385
TC1900009833.hg.1	4.4877	0.00284	2.5467	0.038993
TC0300011273.hg.1	4.4636	0.002923	2.5341	0.039744
TC1700009694.hg.1	4.4627	0.002926	2.5337	0.039744
HTA2.pos.PSR17024059.hg.1	4.4446	0.002991	2.5242	0.040405
TC1800007655.hg.1	-4.3444	0.003378	2.4713	0.045386
TC0300012089.hg.1	4.3027	0.003555	2.4492	0.047506
TSUnmapped00000961.hg.1	-4.2935	0.003595	2.4443	0.047792
HTA2.pos.3597911_st	4.2574	0.003759	2.4249	0.0497
TC2100008391.hg.1	4.2445	0.003819	2.418	0.050231
TC0100014995.hg.1	4.2263	0.003906	2.4083	0.051105
HTA2.pos.3080369_st	4.212	0.003976	2.4006	0.05175
TC1700010777.hg.1	-4.2055	0.004008	2.397	0.0519
TC0100012063.hg.1	4.1988	0.004042	2.3934	0.052062
HTA2.pos.3597953_st	4.1484	0.004304	2.3662	0.055152
TC0100014276.hg.1	4.1054	0.004542	2.3428	0.057728
TC0X00006739.hg.1	4.1039	0.004551	2.3419	0.057728
HTA2.pos.2839524_st	4.0815	0.004681	2.3297	0.059081
TC0400008091.hg.1	-4.0511	0.004864	2.313	0.061081
TC1500008988.hg.1	4.0179	0.005073	2.2947	0.063386
TC0800010807.hg.1	-4.0116	0.005114	2.2913	0.063571
TC1000010776.hg.1	4.0078	0.005139	2.2892	0.063571
HTA2.pos.PSR10005986.hg.1	-4.003	0.00517	2.2865	0.063645
HTA2.pos.JUC05009409.hg.1	-3.9886	0.005266	2.2785	0.06427
TC2200007310.hg.1	3.986	0.005283	2.2771	0.06427
TC1500008995.hg.1	3.9837	0.005299	2.2758	0.06427
TC0800008783.hg.1	3.98	0.005324	2.2738	0.06427
TC0300006723.hg.1	-3.9577	0.005478	2.2614	0.065812
HTA2.pos.PSR04010430.hg.1	-3.9474	0.005551	2.2557	0.066368
TC0800010502.hg.1	-3.9228	0.005729	2.242	0.06794
HTA2.pos.PSR06017444.hg.1	3.9217	0.005736	2.2414	0.06794

TC2200008703.hg.1	3.8431	0.006349	2.1973	0.074843
TC1000007703.hg.1	3.8281	0.006474	2.1888	0.075608
HTA2.pos.3080367_st	3.8245	0.006504	2.1868	0.075608
TC1900009071.hg.1	3.8214	0.00653	2.1851	0.075608
TC0700010538.hg.1	3.8208	0.006535	2.1847	0.075608
TC1100011514.hg.1	3.8079	0.006646	2.1774	0.076538
TC0500008708.hg.1	3.7776	0.006914	2.1603	0.079252
TC0200010275.hg.1	3.7733	0.006953	2.1578	0.07934
TC2000007515.hg.1	3.7538	0.007132	2.1468	0.081015
TC0100010867.hg.1	3.7289	0.00737	2.1326	0.083334
TC1200006909.hg.1	3.7233	0.007424	2.1294	0.083569
HTA2.pos.PSR06035600.hg.1	3.6783	0.007877	2.1036	0.088272
TC0600011635.hg.1	3.6729	0.007934	2.1005	0.088509
TC0900009470.hg.1	3.6591	0.00808	2.0926	0.089141
TC0300010086.hg.1	3.6583	0.008089	2.0921	0.089141
TC0X00007573.hg.1	3.6575	0.008097	2.0917	0.089141
TC0500011668.hg.1	-3.6471	0.008209	2.0857	0.089975
HTA2.pos.PSR10005983.hg.1	-3.6421	0.008265	2.0828	0.090188
TC1400007085.hg.1	-3.6379	0.00831	2.0804	0.090292
TC0200011503.hg.1	3.623	0.008476	2.0718	0.091694
TC0700013428.hg.1	3.6154	0.008562	2.0674	0.092226
HTA2.pos.2839538_st	3.5906	0.00885	2.0531	0.094564
TC1700011449.hg.1	3.5902	0.008855	2.0528	0.094564
TC1100006494.hg.1	3.5863	0.008901	2.0506	0.094649
HTA2.pos.PSR09016040.hg.1	3.5784	0.008996	2.046	0.095253
TC1400010743.hg.1	-3.5748	0.009039	2.0439	0.095311
HTA2.pos.2891951_st	-3.5674	0.009129	2.0396	0.095856
TC2000009949.hg.1	3.5427	0.009435	2.0253	0.098655
TC1100013110.hg.1	3.5381	0.009494	2.0226	0.098854
TC0400010282.hg.1	-3.4727	0.010368	1.9843	0.10751
HTA2.pos.3045353_st	3.4607	0.010537	1.9773	0.10881
TC1700008886.hg.1	3.4544	0.010627	1.9736	0.10916
TC1000008031.hg.1	3.4433	0.010789	1.967	0.10916
TC1200008539.hg.1	3.4433	0.010789	1.967	0.10916
TC1700008713.hg.1	3.4433	0.010789	1.967	0.10916
TC2000006547.hg.1	3.4433	0.010789	1.967	0.10916
TC0500011596.hg.1	3.4395	0.010844	1.9648	0.10927
TC1400008586.hg.1	-3.4337	0.01093	1.9614	0.10969
HTA2.pos.PSR03004164.hg.1	-3.4206	0.011127	1.9536	0.11084
TC0100012754.hg.1	3.4202	0.011133	1.9534	0.11084
HTA2.pos.PSR08018865.hg.1	-3.4059	0.011351	1.945	0.11257
TC1700008704.hg.1	3.3967	0.011494	1.9395	0.11353
TC1400009732.hg.1	3.3863	0.011658	1.9334	0.1147

TC1200006604.hg.1	3.3756	0.011829	1.927	0.11576
TC0100013692.hg.1	3.3738	0.011858	1.926	0.11576
TC1700010414.hg.1	-3.3531	0.012198	1.9137	0.11859
HTA2.pos.2985932_st	3.3464	0.012311	1.9097	0.11859
TC1200008556.hg.1	-3.3393	0.012431	1.9055	0.11859
TC0800007957.hg.1	-3.3336	0.01253	1.9021	0.11859
HTA2.pos.PSR02024018.hg.1	3.3281	0.012624	1.8988	0.11859
TC0100006815.hg.1	-3.3227	0.012718	1.8956	0.11859
TC0300009631.hg.1	-3.3227	0.012718	1.8956	0.11859
TC1000006820.hg.1	-3.3227	0.012718	1.8956	0.11859
TC1400006852.hg.1	-3.3227	0.012718	1.8956	0.11859
TC1900006496.hg.1	-3.3227	0.012718	1.8956	0.11859
TC1900006506.hg.1	-3.3227	0.012718	1.8956	0.11859
TSUnmapped00000838.hg.1	-3.3227	0.012718	1.8956	0.11859
TC0600012754.hg.1	3.3116	0.012912	1.889	0.11995
TC0200014085.hg.1	3.3042	0.013045	1.8846	0.12074
TC0500009768.hg.1	3.2949	0.013214	1.879	0.12185
TC1400010745.hg.1	-3.2877	0.013345	1.8747	0.12219
TC0100010166.hg.1	3.2875	0.013348	1.8746	0.12219
TC1000008318.hg.1	-3.2731	0.013615	1.866	0.12418
TC0100013083.hg.1	3.2535	0.013989	1.8542	0.12712
TC1400007096.hg.1	-3.2381	0.01429	1.845	0.12939
TC0Y00007157.hg.1	3.2341	0.01437	1.8426	0.12964
TC1400009529.hg.1	3.2159	0.014737	1.8316	0.13217
TC1100007262.hg.1	3.215	0.014756	1.831	0.13217
TC1300007491.hg.1	-3.2113	0.014831	1.8288	0.13237
TC0400011297.hg.1	-3.1706	0.015694	1.8043	0.13957
TC1700010677.hg.1	3.1628	0.015867	1.7995	0.14061
TC0X00010849.hg.1	3.1536	0.016072	1.7939	0.14192
HTA2.pos.3686578_st	3.1499	0.016155	1.7917	0.14216
HTA2.pos.PSR02024013.hg.1	3.137	0.016447	1.7839	0.14421
TC1600011418.hg.1	3.1187	0.016874	1.7728	0.14696
TC1700008760.hg.1	3.1186	0.016877	1.7727	0.14696
HTA2.pos.3686564_st	3.0864	0.017655	1.7531	0.15319
TC1700007416.hg.1	3.0652	0.018191	1.7401	0.15625
TC22_KI270733v1_random00006443.hg.1	-3.065	0.018195	1.7401	0.15625
TCUn_GL000220v100006442.hg.1	-3.065	0.018195	1.7401	0.15625
HTA2.pos.PSR01009074.hg.1	3.0549	0.018455	1.7339	0.15794
TC0100011406.hg.1	-3.0456	0.018699	1.7282	0.15948
TC1000010368.hg.1	3.0287	0.019149	1.7179	0.15999
TC0600010690.hg.1	3.0219	0.019334	1.7137	0.15999
TC0600013613.hg.1	3.0219	0.019334	1.7137	0.15999
TC1200011072.hg.1	3.0219	0.019334	1.7137	0.15999

TC1300009866.hg.1	3.0219	0.019334	1.7137	0.15999
TC1600011095.hg.1	3.0219	0.019334	1.7137	0.15999
TC1900011893.hg.1	3.0219	0.019334	1.7137	0.15999
TC2000008178.hg.1	3.0219	0.019334	1.7137	0.15999
TC2200008809.hg.1	3.0219	0.019334	1.7137	0.15999
TC1100012121.hg.1	3.0155	0.019511	1.7097	0.16092
TC0100008145.hg.1	3.0049	0.019803	1.7033	0.16279
TC2100007491.hg.1	2.9924	0.020157	1.6956	0.16515
TC0200014994.hg.1	-2.9673	0.020889	1.6801	0.16682
TC0700012465.hg.1	-2.9673	0.020889	1.6801	0.16682
TC0X00010986.hg.1	-2.9673	0.020889	1.6801	0.16682
TC1400008874.hg.1	-2.9673	0.020889	1.6801	0.16682
TC1500009854.hg.1	-2.9673	0.020889	1.6801	0.16682
TC0X00009238.hg.1	2.9644	0.020974	1.6783	0.16682
TC0600006762.hg.1	-2.9597	0.021114	1.6754	0.16682
TC0500013229.hg.1	2.9551	0.021252	1.6726	0.16682
TC0600012117.hg.1	-2.9533	0.021306	1.6715	0.16682
TC0100017626.hg.1	-2.9427	0.021629	1.665	0.16682
TC0100017627.hg.1	-2.9427	0.021629	1.665	0.16682
TC0100017628.hg.1	-2.9427	0.021629	1.665	0.16682
TC0100017629.hg.1	-2.9427	0.021629	1.665	0.16682
TC0100017630.hg.1	-2.9427	0.021629	1.665	0.16682
TC0100017631.hg.1	-2.9427	0.021629	1.665	0.16682
TC0100017632.hg.1	-2.9427	0.021629	1.665	0.16682
TC0100017633.hg.1	-2.9427	0.021629	1.665	0.16682
TC0100017635.hg.1	-2.9427	0.021629	1.665	0.16682
TC0100017642.hg.1	-2.9427	0.021629	1.665	0.16682
TC1200007826.hg.1	-2.9385	0.02176	1.6623	0.16709
TC0100014934.hg.1	-2.9373	0.021797	1.6616	0.16709
TC0300012773.hg.1	2.9182	0.022397	1.6498	0.17116
TC1500009250.hg.1	2.9021	0.022919	1.6398	0.17411
HTA2.pos.PSR15015202.hg.1	-2.9019	0.022922	1.6397	0.17411
TC1700007319.hg.1	2.8972	0.023077	1.6368	0.17476
TC1200007626.hg.1	2.8841	0.023512	1.6287	0.17742
TC1900008878.hg.1	2.8824	0.02357	1.6276	0.17742
TC0400009221.hg.1	2.8779	0.023724	1.6248	0.17757
HTA2.pos.PSR15015197.hg.1	-2.8776	0.023733	1.6246	0.17757
TC1000008951.hg.1	-2.8719	0.023927	1.6211	0.17849
TC2000010027.hg.1	2.8621	0.024266	1.615	0.18007
TC0100013203.hg.1	-2.8616	0.024283	1.6147	0.18007
TC0800009519.hg.1	-2.8516	0.024632	1.6085	0.18212
TC1000006969.hg.1	-2.843	0.024937	1.6032	0.18381
TC0900007028.hg.1	2.841	0.025008	1.6019	0.18381

TC1000008159.hg.1	-2.8337	0.025272	1.5974	0.18472
TC1000008054.hg.1	2.8322	0.025327	1.5964	0.18472
HTA2.pos.3045314_st	2.8314	0.025354	1.5959	0.18472
TC1700010492.hg.1	2.8117	0.026083	1.5836	0.18931
HTA2.pos.PSR10005982.hg.1	-2.8103	0.026135	1.5828	0.18931
TC0200015242.hg.1	2.8	0.026523	1.5764	0.19156
TC0300010122.hg.1	2.7933	0.02678	1.5722	0.19286
TC0X00007191.hg.1	2.7847	0.027115	1.5668	0.19405
HTA2.pos.PSR07022143.hg.1	2.7839	0.027147	1.5663	0.19405
TC2000010026.hg.1	2.7831	0.027177	1.5658	0.19405
HTA2.pos.PSR02024022.hg.1	2.7777	0.027388	1.5624	0.195
TC1000011938.hg.1	2.773	0.027573	1.5595	0.19576
TC0500006476.hg.1	2.7709	0.027658	1.5582	0.1958
HTA2.pos.3271672_st	-2.7688	0.027741	1.5569	0.19583
TC1100010455.hg.1	2.7552	0.028289	1.5484	0.19914
TC0100017634.hg.1	-2.7514	0.028447	1.546	0.19969
TC0200015248.hg.1	2.7139	0.030031	1.5224	0.21021
HTA2.pos.2891949_st	-2.7114	0.030139	1.5209	0.21038
TC1800007680.hg.1	2.7085	0.030265	1.5191	0.21068
TC0900007033.hg.1	2.6958	0.030824	1.5111	0.21397
TC0200011720.hg.1	-2.6805	0.031515	1.5015	0.21816
TC2200007054.hg.1	-2.6782	0.031621	1.5	0.21829
TC0700006640.hg.1	2.6724	0.031889	1.4964	0.21954
HTA2.pos.PSR01009067.hg.1	2.6683	0.03208	1.4938	0.22024
TC1900010738.hg.1	2.665	0.032234	1.4917	0.22069
TC0600008379.hg.1	-2.6598	0.032476	1.4884	0.22129
TC1900007012.hg.1	2.6593	0.032498	1.4881	0.22129
TC1500010197.hg.1	2.6491	0.032987	1.4817	0.22401
HTA2.pos.JUC03002217.hg.1	-2.6466	0.033106	1.4801	0.22421
HTA2.pos.3255800_st	-2.6409	0.033378	1.4765	0.22544
TCUn_GL000220v100006441.hg.1	-2.6313	0.033851	1.4704	0.22801
TC1500006842.hg.1	-2.6206	0.034382	1.4637	0.23097
TC0700006648.hg.1	2.6098	0.034924	1.4569	0.23217
TC0300013282.hg.1	-2.603	0.035269	1.4526	0.23217
TC0200009857.hg.1	-2.6003	0.035412	1.4509	0.23217
TC0100017636.hg.1	-2.5999	0.035431	1.4506	0.23217
TC0100017637.hg.1	-2.5999	0.035431	1.4506	0.23217
TC0100017638.hg.1	-2.5999	0.035431	1.4506	0.23217
TC0100017639.hg.1	-2.5999	0.035431	1.4506	0.23217
TC0100017640.hg.1	-2.5999	0.035431	1.4506	0.23217
TC0100017641.hg.1	-2.5999	0.035431	1.4506	0.23217
TC0200007237.hg.1	2.5987	0.03549	1.4499	0.23217
TC0100007898.hg.1	2.5942	0.035725	1.447	0.2331

TC1300007752.hg.1	-2.5909	0.035897	1.4449	0.23361
TC1100006911.hg.1	-2.583	0.036312	1.44	0.2357
TC0600012753.hg.1	2.5786	0.036544	1.4372	0.23659
TC0100009542.hg.1	2.5726	0.036866	1.4334	0.23805
TC0X00010608.hg.1	-2.5676	0.037138	1.4302	0.2392
TC1500007510.hg.1	2.5587	0.037623	1.4245	0.2417
TC0400012829.hg.1	2.5568	0.03773	1.4233	0.24176
HTA2.pos.3525998_st	-2.5539	0.037888	1.4215	0.24215
TC0600009007.hg.1	2.5502	0.038091	1.4192	0.24283
HTA2.pos.PSR02004954.hg.1	-2.5368	0.038842	1.4107	0.2469
TC1600010357.hg.1	2.5354	0.038927	1.4097	0.2469
TC0300012216.hg.1	2.5264	0.039441	1.4041	0.24952
TC1700008986.hg.1	2.5162	0.040032	1.3976	0.25262
HTA2.pos.PSR08018863.hg.1	-2.5088	0.040469	1.3929	0.25474
HTA2.pos.PSR03029262.hg.1	-2.4964	0.041206	1.385	0.25873
TC1200010662.hg.1	2.494	0.041357	1.3835	0.25902
TC1200011527.hg.1	-2.4782	0.04232	1.3735	0.26439
TC1200007605.hg.1	2.4741	0.042579	1.3708	0.26496
TC1100010087.hg.1	-2.4734	0.042622	1.3704	0.26496
TSUnmapped00000831.hg.1	-2.4696	0.04286	1.3679	0.26578
TC0M00006441.hg.1	-2.4663	0.043066	1.3659	0.26639
HTA2.pos.2928786_st	-2.4635	0.043242	1.3641	0.26682
TC0500007761.hg.1	2.4496	0.04413	1.3553	0.27163
TSUnmapped00000920.hg.1	-2.4404	0.044735	1.3494	0.27468
TC0200016202.hg.1	2.4316	0.045312	1.3438	0.27753
TC0700007431.hg.1	2.4269	0.045627	1.3408	0.27878
TC0800008245.hg.1	2.4247	0.045775	1.3394	0.27881
TC0200015178.hg.1	2.4235	0.045854	1.3386	0.27881
TC0100007008.hg.1	2.4194	0.046132	1.336	0.27981
TC0700011835.hg.1	2.4109	0.04671	1.3306	0.28264
HTA2.pos.3045327_st	2.4056	0.047077	1.3272	0.28286
HTA2.pos.PSR05013906.hg.1	-2.4056	0.047078	1.3272	0.28286
TC1700010994.hg.1	2.4055	0.047087	1.3271	0.28286
TC0100006873.hg.1	-2.3993	0.047516	1.3232	0.28475
HTA2.pos.3045312_st	2.3949	0.047826	1.3203	0.28593
TC0500011775.hg.1	-2.39	0.048169	1.3172	0.28729
TC0100016694.hg.1	2.3882	0.048296	1.3161	0.28736
HTA2.pos.PSR09016043.hg.1	2.3864	0.048427	1.3149	0.28746
TC1900010452.hg.1	2.384	0.048598	1.3134	0.28779
HTA2.pos.3597957_st	2.382	0.048738	1.3121	0.28793
TC0400011560.hg.1	2.3767	0.049121	1.3087	0.28951
TC0700007897.hg.1	-2.3666	0.049855	1.3023	0.29293

Supplementary Table 12: Reactome and Panther pathway analysis of 36 Common gene HRTPT cells recovery to iAs passage P2, P11

Reactome Pathways

Pathway name p-value FDR
Molecules associated with elastic fibres 1.13e-04 0.065
Glucuronidation 3.69e-04 0.065
Elastic fibre formation 4.25e-04 0.065
Transcriptional activation of cell cycle inhibitor p21 5.31e-04 0.065
Transcriptional activation of p53 responsive genes 5.31e-04 0.065
Aspirin ADME 0.001 0.134
TP53 Regulates Transcription of Genes Involved in G1 Cell Cycle Arrest
0.003 0.289
Heme degradation 0.005 0.391
Interleukin-4 and Interleukin-13 signaling 0.009 0.468
Defective UGT1A4 causes hyperbilirubinemia 0.009 0.468
Defective UGT1A1 causes hyperbilirubinemia 0.009 0.468
Paracetamol ADME 0.009 0.468
Drug ADME 0.012 0.468
O-linked glycosylation of mucins 0.012 0.468
FOXO-mediated transcription of cell cycle genes 0.014 0.468
RUNX3 regulates CDKN1A transcription 0.014 0.468
p53-Dependent G1/S DNA damage checkpoint 0.018 0.468
p53-Dependent G1 DNA Damage Response 0.018 0.468

Panther

Biological Processes (>3 Genes)

Biological Regulation (7)
Cellular Process (9)
Developmental Process (4)
Multicellular Organismal Process (4)
Response to Stimulus (5)
Signaling (4)

Molecular Function (> 2 Genes)

Binding (9)
Molecular Function Regulator (3)
Molecular Transducer Activity (3)

Protein Class (> 2 genes)

None

Pathways (> 1 Gene)

None

Supplementary Table 13: Downregulated Gene Set Enrichment Analysis for significant genes between the control and iAs with nominal p-value < 0.05.

GS	Size	ES	NES	NOM p-val	FDR q-val	FWER p-val	Rank at Max	Leading Edge
TGANTCA_AP1_C	6	-0.72	-1.96	0.009	0.005	0.005	13	tags=83%, list=30%, signal=102%
RODRIGUES_THYROID_CARCINOMA_POORLY_DIFFERENTIATED_DN	5	-0.67	-1.7	0.019	0.047	0.017	19	tags=100%, list=43%, signal=156%

Supplementary Table 14: Ingenuity Pathway Analysis of Supplementary Table 10.

© 2000-2022 QIAGEN. All rights reserved.

Ingenuity Canonical Pathways	-log(p-value)	Ratio	z-score	Molecules
Superpathway of Cholesterol Biosynthesis	16.7	0.379	#NUM!	ACAT2,CYP51A1,DHCR24,DHCR7,EBP,FDFT1,HMGCR,HMGCS1,IDI1,MSMO1,SQLE
Cholesterol Biosynthesis I	12.1	0.538	#NUM!	CYP51A1,DHCR24,DHCR7,EBP,FDFT1,MSMO1,SQLE
Cholesterol Biosynthesis II (via 24,25-dihydrolanosterol)	12.1	0.538	#NUM!	CYP51A1,DHCR24,DHCR7,EBP,FDFT1,MSMO1,SQLE
Cholesterol Biosynthesis III (via Desmosterol)	12.1	0.538	#NUM!	CYP51A1,DHCR24,DHCR7,EBP,FDFT1,MSMO1,SQLE
Mevalonate Pathway I	5.78	0.286	#NUM!	ACAT2,HMGCR,HMGCS1,IDI1
Superpathway of Geranylgeranyldiphosphate Biosynthesis I (via Mevalonate)	5.3	0.222	#NUM!	ACAT2,HMGCR,HMGCS1,IDI1
Tight Junction Signaling	4.62	0.0447	#NUM!	CLDN1,CLDN10,CLDN16,MYH10,MYLK,RAC1,STX16,VCL
Epoxysqualene Biosynthesis	4.37	1	#NUM!	FDFT1,SQLE
Ferroptosis Signaling Pathway	3.63	0.0455	#NUM!	ARF4,CBS/LOC102724560,EMP1,FDFT1,HMGCR,SAT1
Coronavirus Pathogenesis Pathway	3.4	0.0343	#NUM!	RN7SL1,RN7SL2,RNU2-1,RPS20,RPS24,STAT1,STING1
Integrin Signaling	3.3	0.033	#NUM!	ACTN1,ARF4,MYLK,RAC1,RHOB,TSPAN1,VCL
Zymosterol Biosynthesis	3.2	0.333	#NUM!	CYP51A1,MSMO1
Systemic Lupus Erythematosus Signaling	3.04	0.019	#NUM!	HNRNPA2B1,RNU1-2,RNU1-27P,RNU1-28P,RNU1-3,RNU1-4,RNU2-1,RNU5E-1,RNU6-1,RNU6-15P,RNVU1-18,RNVU1-7
Leukocyte Extravasation Signaling	2.76	0.0311	#NUM!	ACTN1,CLDN1,CLDN10,CLDN16,RAC1,VCL
Ketogenesis	2.65	0.182	#NUM!	ACAT2,HMGCS1
Sertoli Cell-Sertoli Cell Junction Signaling	2.62	0.0291	#NUM!	ACTN1,CLDN1,CLDN10,CLDN16,RAC1,VCL
Oleate Biosynthesis II (Animals)	2.5	0.154	#NUM!	FADS2,SCD5
Acetyl-CoA Biosynthesis III (from Citrate)	2.18	1	#NUM!	ACLY
MicroRNA Biogenesis Signaling Pathway	2.11	0.0267	#NUM!	DDX17,DDX5,FUS,HNRNPA2B1,SRSF1
LXR/RXR Activation	2.06	0.0325	#NUM!	CYP51A1,FDFT1,HMGCR,LDLR
ILK Signaling	1.98	0.0249	#NUM!	ACTN1,MYH10,RAC1,RHOB,VCL
Glioma Invasiveness Signaling	1.91	0.0411	#NUM!	PLAU,RAC1,RHOB
Agranulocyte Adhesion and Diapedesis	1.9	0.0238	#NUM!	CLDN1,CLDN10,CLDN16,CXCL14,MYH10
Cysteine Biosynthesis/Homocysteine Degradation	1.89	0.5	#NUM!	CBS/LOC102724560
Putrescine Biosynthesis III	1.89	0.5	#NUM!	ODC1
Glutamine Degradation I	1.89	0.5	#NUM!	GLS
Regulation of Cellular Mechanics by Calpain Protease	1.68	0.0337	#NUM!	ACTN1,CAST,VCL
Actin Cytoskeleton Signaling	1.65	0.0205	#NUM!	ACTN1,MYH10,MYLK,RAC1,VCL
Complement System	1.61	0.0541	#NUM!	CD55,CFI
Germ Cell-Sertoli Cell Junction Signaling	1.59	0.0235	#NUM!	ACTN1,RAC1,RHOB,VCL
Spermine and Spermidine Degradation I	1.59	0.25	#NUM!	SAT1
Tumor Microenvironment Pathway	1.52	0.0223	#NUM!	CFLAR,PLAU,RAC1,SPP1
Retinoate Biosynthesis I	1.51	0.0476	#NUM!	AKR1B1,RDH10
Oncostatin M Signaling	1.49	0.0465	#NUM!	PLAU,STAT1
Trans, trans-farnesyl Diphosphate Biosynthesis	1.49	0.2	#NUM!	IDI1
Paxillin Signaling	1.48	0.028	#NUM!	ACTN1,RAC1,VCL
Granulocyte Adhesion and Diapedesis	1.45	0.0212	#NUM!	CLDN1,CLDN10,CLDN16,CXCL14
Glucocorticoid Receptor Signaling	1.42	0.0137	#NUM!	FKBP5,GJA1,HLA-DMB,KRT19,MT-ND1,PLAU,RAC1,STAT1
Glycine Cleavage Complex	1.41	0.167	#NUM!	GLDC
Regulation of Actin-based Motility by Rho	1.4	0.0261	#NUM!	MYLK,RAC1,RHOB
Sirtuin Signaling Pathway	1.36	0.0171	#NUM!	ACLY,GLS,MT-ND1,PDHA1,SCNN1A
Ceramide Biosynthesis	1.35	0.143	#NUM!	SPTLC3
Acetyl-CoA Biosynthesis I (Pyruvate Dehydrogenase Complex)	1.35	0.143	#NUM!	PDHA1
CSDE1 Signaling Pathway	1.28	0.0357	#NUM!	FUS,RAC1
mTOR Signaling	1.28	0.0187	#NUM!	RAC1,RHOB,RPS20,RPS24

Polyamine Regulation in Colon Cancer	1.26	0.0345	#NUM!	ODC1,SAT1
Synaptogenesis Signaling Pathway	1.25	0.0159	#NUM!	CDH3,FARP1,RAC1,STX16,THBS1
RHOGDI Signaling	1.25	0.0182	#NUM!	CDH3,MYH10,RAC1,RHOB
Citrulline Biosynthesis	1.24	0.111	#NUM!	GLS
Neuroinflammation Signaling Pathway	1.24	0.0158	#NUM!	CFLAR,GLS,HLA-DMB,RAC1,STAT1
Semaphorin Signaling in Neurons	1.22	0.0328	#NUM!	RAC1,RHOB
Thrombin Signaling	1.22	0.0178	#NUM!	ARHGEF38,MYLK,RAC1,RHOB
EIF2 Signaling	1.21	0.0176	#NUM!	RPL41,RPL7,RPS20,RPS24
Ketolysis	1.2	0.1	#NUM!	ACAT2
Remodeling of Epithelial Adherens Junctions	1.14	0.0294	#NUM!	ACTN1,VCL
Semaphorin Neuronal Repulsive Signaling Pathway	1.12	0.02	#NUM!	FARP1,RAC1,SEMA6A
Mineralocorticoid Biosynthesis	1.09	0.0769	#NUM!	EBP
Epithelial Adherens Junction Signaling	1.08	0.0191	#NUM!	MYH10,RAC1,VCL
Glucocorticoid Biosynthesis	1.06	0.0714	#NUM!	EBP
Telomere Extension by Telomerase	1.03	0.0667	#NUM!	HNRNPA2B1
Superpathway of Citrulline Metabolism	1.03	0.0667	#NUM!	GLS
IL-3 Signaling	1.02	0.0253	#NUM!	RAC1,STAT1
Pathogen Induced Cytokine Storm Signaling Pathway	1.01	0.0134	#NUM!	CXCL14,HLA-DMB,HMGCR,STAT1,STING1
Signaling by Rho Family GTPases	1.01	0.015	#NUM!	CDH3,MYLK,RAC1,RHOB
Parkinson's Signaling	1	0.0625	#NUM!	UCHL1
Insulin Secretion Signaling Pathway	0.983	0.0147	#NUM!	PDHA1,SCNN1A,STAT1,STX16
PEDF Signaling	0.979	0.0238	#NUM!	CFLAR,RAC1
Isoleucine Degradation I	0.955	0.0556	#NUM!	ACAT2
Immunogenic Cell Death Signaling Pathway	0.932	0.0222	#NUM!	CFLAR,STING1
Glutaryl-CoA Degradation	0.932	0.0526	#NUM!	ACAT2
γ-linolenate Biosynthesis II (Animals)	0.932	0.0526	#NUM!	FADS2
Actin Nucleation by ARP-WASP Complex	0.907	0.0215	#NUM!	RAC1,RHOB
Macrophage Classical Activation Signaling Pathway	0.896	0.0159	#NUM!	HLA-DMB,PDHA1,STAT1
Androgen Biosynthesis	0.889	0.0476	#NUM!	EBP
Production of Nitric Oxide and Reactive Oxygen Species in Macrophages	0.886	0.0157	#NUM!	RAC1,RHOB,STAT1
Putrescine Degradation III	0.873	0.0455	#NUM!	SAT1
VEGF Signaling	0.863	0.0202	#NUM!	ACTN1,VCL
The Visual Cycle	0.854	0.0435	#NUM!	RDH10
IL-22 Signaling	0.836	0.0417	#NUM!	STAT1
Sumoylation Pathway	0.836	0.0194	#NUM!	RAC1,RHOB
Virus Entry via Endocytic Pathways	0.83	0.0192	#NUM!	CD55,RAC1
RAR Activation	0.824	0.0147	#NUM!	AKR1B1,RAC1,RDH10
Role of JAK family kinases in IL-6-type Cytokine Signaling	0.821	0.04	#NUM!	STAT1
Bupropion Degradation	0.821	0.04	#NUM!	CYP51A1
Role of JAK1, JAK2 and TYK2 in Interferon Signaling	0.804	0.0385	#NUM!	STAT1
Tryptophan Degradation III (Eukaryotic)	0.804	0.0385	#NUM!	ACAT2
Cysteine Biosynthesis III (mammalia)	0.79	0.037	#NUM!	CBS/LOC102724560
Multiple Sclerosis Signaling Pathway	0.75	0.0135	#NUM!	HLA-DMB,PLAU,STAT1
PAK Signaling	0.75	0.0171	#NUM!	MYLK,RAC1
Role of p14/p19ARF in Tumor Suppression	0.747	0.0333	#NUM!	RAC1
Cholecystokinin/Gastrin-mediated Signaling	0.738	0.0168	#NUM!	RAC1,RHOB
MSP-RON Signaling In Macrophages Pathway	0.738	0.0168	#NUM!	HLA-DMB,STAT1
Sphingosine-1-phosphate Signaling	0.733	0.0167	#NUM!	RAC1,RHOB
Renin-Angiotensin Signaling	0.726	0.0165	#NUM!	RAC1,STAT1
Methylglyoxal Degradation III	0.724	0.0312	#NUM!	AKR1B1

Th1 Pathway	0.721	0.0164	#NUM!	HLA-DMB,STAT1
Pancreatic Adenocarcinoma Signaling	0.701	0.0159	#NUM!	RAC1,STAT1
Inhibition of Angiogenesis by TSP1	0.699	0.0294	#NUM!	THBS1
Role of JAK2 in Hormone-like Cytokine Signaling	0.699	0.0294	#NUM!	STAT1
Osteoarthritis Pathway	0.697	0.0127	#NUM!	PTH1R,RAC1,SPP1
GP6 Signaling Pathway	0.695	0.0157	#NUM!	LAMC2,RAC1
Coagulation System	0.688	0.0286	#NUM!	PLAU
IL-9 Signaling	0.688	0.0286	#NUM!	STAT1
Interferon Signaling	0.676	0.0278	#NUM!	STAT1
CCR3 Signaling in Eosinophils	0.66	0.0149	#NUM!	MYLK,RAC1
Adipogenesis pathway	0.656	0.0148	#NUM!	KLF5,TXNIP
SNARE Signaling Pathway	0.652	0.0147	#NUM!	MYH10,STX16
Antigen Presentation Pathway	0.646	0.0256	#NUM!	HLA-DMB
Wound Healing Signaling Pathway	0.642	0.0119	#NUM!	LAMC2,RAC1,STAT1
Reelin Signaling in Neurons	0.642	0.0145	#NUM!	ARHGEF38,RAC1
Superpathway of Methionine Degradation	0.636	0.025	#NUM!	CBS/LOC102724560
Insulin Receptor Signaling	0.633	0.0143	#NUM!	ACLY,SCNN1A
Role Of Chondrocytes In Rheumatoid Arthritis Signaling Pathway	0.629	0.0142	#NUM!	CFLAR,PLAU
Axonal Guidance Signaling	0.618	0.00982	#NUM!	ADAMTS1,LNPEP,NTN4,RAC1,SEMA6A
PI3K Signaling in B Lymphocytes	0.611	0.0138	#NUM!	MALT1,RAC1
Acetone Degradation I (to Methylglyoxal)	0.609	0.0233	#NUM!	CYP51A1
Estrogen Biosynthesis	0.592	0.0222	#NUM!	CYP51A1
Dilated Cardiomyopathy Signaling Pathway	0.59	0.0133	#NUM!	MYH10,TPM1
Cellular Effects of Sildenafil (Viagra)	0.59	0.0133	#NUM!	MYH10,MYLK
Role of OCT4 in Mammalian Embryonic Stem Cell Pluripotency	0.583	0.0217	#NUM!	SPP1
Colorectal Cancer Metastasis Signaling	0.583	0.0111	#NUM!	RAC1,RHOB,STAT1
B Cell Development	0.583	0.0217	#NUM!	HLA-DMB
iNOS Signaling	0.575	0.0213	#NUM!	STAT1
Ephrin A Signaling	0.575	0.0213	#NUM!	RAC1
Retinol Biosynthesis	0.575	0.0213	#NUM!	RDH10
Necroptosis Signaling Pathway	0.565	0.0128	#NUM!	CFLAR,STAT1
Phagosome Maturation	0.558	0.0127	#NUM!	RAC1,STX16
MYC Mediated Apoptosis Signaling	0.553	0.02	#NUM!	CFLAR
Oxytocin Signaling Pathway	0.551	0.0106	#NUM!	GJA1,MYH10,MYLK
HMGB1 Signaling	0.526	0.012	#NUM!	RAC1,RHOB
Hepatic Fibrosis Signaling Pathway	0.523	0.00946	#NUM!	MYLK,RAC1,RHOB,SPP1
CXCR4 Signaling	0.521	0.0119	#NUM!	RAC1,RHOB
EGF Signaling	0.519	0.0182	#NUM!	STAT1
Ribonucleotide Reductase Signaling Pathway	0.514	0.0118	#NUM!	CDH3,THBS1
Gαq Signaling	0.514	0.0118	#NUM!	RAC1,RHOB
Mitochondrial Dysfunction	0.511	0.0117	#NUM!	MT-ND1,PDHA1
Glioblastoma Multiforme Signaling	0.511	0.0117	#NUM!	RAC1,RHOB
Th1 and Th2 Activation Pathway	0.509	0.0116	#NUM!	HLA-DMB,STAT1
Senescence Pathway	0.507	0.01	#NUM!	DHCR24,PDHA1,STING1
CNTF Signaling	0.506	0.0175	#NUM!	STAT1
WNT/β-catenin Signaling	0.502	0.0115	#NUM!	CDH3,GJA1
Nicotine Degradation III	0.499	0.0172	#NUM!	CYP51A1
Retinoic acid Mediated Apoptosis Signaling	0.487	0.0167	#NUM!	CFLAR
PCP (Planar Cell Polarity) Pathway	0.487	0.0167	#NUM!	RAC1
Role Of Osteoclasts In Rheumatoid Arthritis Signaling Pathway	0.485	0.00974	#NUM!	RAC1,RHOB,SPP1

Regulation of eIF4 and p70S6K Signaling	0.479	0.011	#NUM!	RPS20,RPS24
Melatonin Degradation I	0.475	0.0161	#NUM!	CYP51A1
Thrombopoietin Signaling	0.47	0.0159	#NUM!	STAT1
Activation of IRF by Cytosolic Pattern Recognition Receptors	0.458	0.0154	#NUM!	STAT1
Nicotine Degradation II	0.453	0.0152	#NUM!	CYP51A1
Glutamate Receptor Signaling	0.453	0.0152	#NUM!	GLS
Superpathway of Melatonin Degradation	0.449	0.0149	#NUM!	CYP51A1
Stearate Biosynthesis I (Animals)	0.449	0.0149	#NUM!	DHCR24
Pulmonary Fibrosis Idiopathic Signaling Pathway	0.445	0.0092	#NUM!	ADAMTS1,PLAU,THBS1
Clathrin-mediated Endocytosis Signaling	0.444	0.0104	#NUM!	LDLR,RAC1
Hepatic Fibrosis / Hepatic Stellate Cell Activation	0.44	0.0103	#NUM!	MYH10,STAT1
Agrin Interactions at Neuromuscular Junction	0.438	0.0145	#NUM!	RAC1
Role of JAK1 and JAK3 in γc Cytokine Signaling	0.438	0.0145	#NUM!	STAT1
GM-CSF Signaling	0.433	0.0143	#NUM!	STAT1
Natural Killer Cell Signaling	0.429	0.0101	#NUM!	PVR,RAC1
Growth Hormone Signaling	0.428	0.0141	#NUM!	STAT1
Pulmonary Healing Signaling Pathway	0.427	0.0101	#NUM!	RAC1,THBS1
Ephrin B Signaling	0.424	0.0139	#NUM!	RAC1
Netrin Signaling	0.424	0.0139	#NUM!	RAC1
CDX Gastrointestinal Cancer Signaling Pathway	0.419	0.0099	#NUM!	KLF5,RAC1
Caveolar-mediated Endocytosis Signaling	0.41	0.0133	#NUM!	CD55
Granzyme A Signaling	0.41	0.0133	#NUM!	MT-ND1
GDNF Family Ligand-Receptor Interactions	0.406	0.0132	#NUM!	RAC1
Macropinocytosis Signaling	0.406	0.0132	#NUM!	RAC1
HIF1α Signaling	0.403	0.00962	#NUM!	RAC1,SAT1
IL-8 Signaling	0.398	0.00952	#NUM!	RAC1,RHOB
VDR/RXR Activation	0.397	0.0128	#NUM!	SPP1
IL-7 Signaling Pathway	0.397	0.0128	#NUM!	STAT1
Renal Cell Carcinoma Signaling	0.393	0.0127	#NUM!	RAC1
Role of BRCA1 in DNA Damage Response	0.388	0.0125	#NUM!	STAT1
ERK/MAPK Signaling	0.386	0.0093	#NUM!	RAC1,STAT1
FLT3 Signaling in Hematopoietic Progenitor Cells	0.38	0.0122	#NUM!	STAT1
JAK/STAT Signaling	0.38	0.0122	#NUM!	STAT1
BEX2 Signaling Pathway	0.38	0.0122	#NUM!	SPP1
Calcium Signaling	0.377	0.00913	#NUM!	MYH10,TPM1
TR/RXR Activation	0.373	0.0119	#NUM!	LDLR
LPS-stimulated MAPK Signaling	0.369	0.0118	#NUM!	RAC1
Role of Hypercytokinemia/hyperchemokinemia in the Pathogenesis of Influenza	0.365	0.0116	#NUM!	STAT1
FGF Signaling	0.365	0.0116	#NUM!	RAC1
PDGF Signaling	0.365	0.0116	#NUM!	STAT1
Prolactin Signaling	0.347	0.011	#NUM!	STAT1
Crosstalk between Dendritic Cells and Natural Killer Cells	0.347	0.011	#NUM!	FSCN1
Apelin Adipocyte Signaling Pathway	0.347	0.011	#NUM!	RAC1
Pyroptosis Signaling Pathway	0.34	0.0108	#NUM!	TXNIP
IL-4 Signaling	0.34	0.0108	#NUM!	HLA-DMB
Fcγ Receptor-mediated Phagocytosis in Macrophages and Monocytes	0.336	0.0106	#NUM!	RAC1
Death Receptor Signaling	0.33	0.0104	#NUM!	CFLAR
p53 Signaling	0.323	0.0102	#NUM!	THBS1
UVA-Induced MAPK Signaling	0.323	0.0102	#NUM!	STAT1
Phagosome Formation	0.322	0.00722	#NUM!	MALT1,MYH10,MYLK,PTH1R,RAC1

Apelin Cardiomyocyte Signaling Pathway	0.321	0.0101	#NUM!	MYLK
IGF-1 Signaling	0.305	0.00962	#NUM!	IGFBP7
PD-1, PD-L1 cancer immunotherapy pathway	0.299	0.00943	#NUM!	HLA-DMB
Cardiac Hypertrophy Signaling	0.292	0.00766	#NUM!	RAC1,RHOB
Oxidative Phosphorylation	0.286	0.00901	#NUM!	MT-ND1
CDK5 Signaling	0.275	0.0087	#NUM!	LAMC2
IL-13 Signaling Pathway	0.272	0.00862	#NUM!	STAT1
Amyotrophic Lateral Sclerosis Signaling	0.272	0.00862	#NUM!	RAC1
Bladder Cancer Signaling	0.272	0.00862	#NUM!	THBS1
Role of Tissue Factor in Cancer	0.272	0.00862	#NUM!	RAC1
Fc Epsilon RI Signaling	0.268	0.00847	#NUM!	RAC1
NGF Signaling	0.263	0.00833	#NUM!	RAC1
p38 MAPK Signaling	0.263	0.00833	#NUM!	STAT1
Huntington's Disease Signaling	0.256	0.00707	#NUM!	GLS,STX16
IL-15 Production	0.256	0.00813	#NUM!	STAT1
RHOA Signaling	0.253	0.00806	#NUM!	MYLK
Molecular Mechanisms of Cancer	0.251	0.00673	#NUM!	CFLAR,RAC1,RHOB
Gαs Signaling	0.251	0.008	#NUM!	PTH1R
Endocannabinoid Developing Neuron Pathway	0.247	0.00787	#NUM!	RAC1
fMLP Signaling in Neutrophils	0.238	0.00763	#NUM!	RAC1
HGF Signaling	0.237	0.00758	#NUM!	RAC1
Gα12/13 Signaling	0.234	0.00752	#NUM!	CDH3
STAT3 Pathway	0.23	0.00741	#NUM!	RAC1
IL-12 Signaling and Production in Macrophages	0.228	0.00735	#NUM!	STAT1
Role of PKR in Interferon Induction and Antiviral Response	0.228	0.00735	#NUM!	STAT1
RAC Signaling	0.226	0.0073	#NUM!	RAC1
Th2 Pathway	0.226	0.0073	#NUM!	HLA-DMB
PTEN Signaling	0.203	0.00667	#NUM!	RAC1
NAD Signaling Pathway	0.201	0.00662	#NUM!	GJA1
Neurovascular Coupling Signaling Pathway	0	0.00442	#NUM!	GJA1
Role Of Osteoblasts In Rheumatoid Arthritis Signaling Pathway	0	0.00417	#NUM!	STAT1
NRF2-mediated Oxidative Stress Response	0	0.00422	#NUM!	FKBP5
LPS/IL-1 Mediated Inhibition of RXR Function	0	0.00394	#NUM!	HMGCS1
Erythropoietin Signaling Pathway	0	0.00565	#NUM!	RAC1
Role of NFAT in Regulation of the Immune Response	0	0.00169	#NUM!	HLA-DMB
Calcium-induced T Lymphocyte Apoptosis	0	0.00217	#NUM!	HLA-DMB
IL-17 Signaling	0	0.00535	#NUM!	SRSF1
CTLA4 Signaling in Cytotoxic T Lymphocytes	0	0.00493	#NUM!	HLA-DMB,MALT1,RAC1
T Helper Cell Differentiation	0	0.00425	#NUM!	HLA-DMB,STAT1
CD28 Signaling in T Helper Cells	0	0.0058	#NUM!	HLA-DMB,MALT1,RAC1
Dendritic Cell Maturation	0	0.00503	#NUM!	FSCN1,HLA-DMB,STAT1
ICOS-ICOSL Signaling in T Helper Cells	0	0.00394	#NUM!	HLA-DMB,RAC1
GNRH Signaling	0	0.00524	#NUM!	RAC1
Aldosterone Signaling in Epithelial Cells	0	0.00595	#NUM!	SCNN1A
CREB Signaling in Neurons	0	0.00165	#NUM!	PTH1R
Type I Diabetes Mellitus Signaling	0	0.00393	#NUM!	HLA-DMB,STAT1
Allograft Rejection Signaling	0	0.00205	#NUM!	HLA-DMB
Autoimmune Thyroid Disease Signaling	0	0.00218	#NUM!	HLA-DMB
Graft-versus-Host Disease Signaling	0	0.00224	#NUM!	HLA-DMB
CDC42 Signaling	0	0.00347	#NUM!	HLA-DMB,MYLK

FAK Signaling	0	0.00192	#NUM!	PTH1R,RAC1
AMPK Signaling	0	0.00413	#NUM!	HMGCR
Role of Osteoblasts, Osteoclasts and Chondrocytes in Rheumatoid Arthritis	0	0.00446	#NUM!	SPP1
Phospholipase C Signaling	0	0.00293	#NUM!	RAC1,RHOB
Ovarian Cancer Signaling	0	0.00633	#NUM!	GJA1
HER-2 Signaling in Breast Cancer	0	0.00441	#NUM!	ARF4
Altered T Cell and B Cell Signaling in Rheumatoid Arthritis	0	0.00405	#NUM!	HLA-DMB,SPP1
Protein Kinase A Signaling	0	0.00488	#NUM!	MYH10,MYLK
Role of Macrophages, Fibroblasts and Endothelial Cells in Rheumatoid Arthritis	0	0.00301	#NUM!	RAC1
Breast Cancer Regulation by Stathmin1	0	0.00337	#NUM!	PTH1R,RAC1
Regulation of IL-2 Expression in Activated and Anergic T Lymphocytes	0	0.00433	#NUM!	MALT1,RAC1
NUR77 Signaling in T Lymphocytes	0	0.00195	#NUM!	HLA-DMB
PKCθ Signaling in T Lymphocytes	0	0.00538	#NUM!	HLA-DMB,MALT1,RAC1
OX40 Signaling Pathway	0	0.00209	#NUM!	HLA-DMB
Gap Junction Signaling	0	0.00505	#NUM!	GJA1
TEC Kinase Signaling	0	0.00519	#NUM!	RAC1,RHOB,STAT1
Estrogen Receptor Signaling	0	0.00489	#NUM!	DDX5,MT-ND1
SAPK/JNK Signaling	0	0.00199	#NUM!	RAC1
Protein Ubiquitination Pathway	0	0.00365	#NUM!	UCHL1
B Cell Receptor Signaling	0	0.00315	#NUM!	MALT1,RAC1
cAMP-mediated signaling	0	0.00426	#NUM!	PTH1R
NF-κB Signaling	0	0.00175	#NUM!	MALT1
T Cell Receptor Signaling	0	0.00487	#NUM!	HLA-DMB,MALT1,RAC1
G-Protein Coupled Receptor Signaling	0	0.00427	#NUM!	MYLK,PTH1R,RAC1
Gustation Pathway	0	0.00493	#NUM!	SCNN1A
Opioid Signaling Pathway	0	0.00358	#NUM!	RAC1
Adrenomedullin signaling pathway	0	0.00503	#NUM!	MYLK
T Cell Exhaustion Signaling Pathway	0	0.00353	#NUM!	HLA-DMB,STAT1
Systemic Lupus Erythematosus In T Cell Signaling Pathway	0	0.00466	#NUM!	HLA-DMB,RAC1,RHOB
Systemic Lupus Erythematosus In B Cell Signaling Pathway	0	0.00552	#NUM!	MALT1,RAC1,STAT1,STING1
HOTAIR Regulatory Pathway	0	0.00613	#NUM!	SPP1
Regulation Of The Epithelial Mesenchymal Transition By Growth Factors Pathway	0	0.00521	#NUM!	RAC1
Ephrin Receptor Signaling	0	0.00495	#NUM!	RAC1

Supplementary Table 15: Differentially expressed genes between iAs exposed HRTPT cells (P3, P8, P10) and HRTPT cells following recovery from iAs exposure (P2, P11).

probe	FC	log2(FC)	Raw.pval	log(Pvalue)	Gene Symbol
TC0200009068.hg.1	11.823	3.5635	5.78E-09	8.2378	
TC0200015624.hg.1	6.613	2.7253	6.31E-07	6.2	
TC0700006913.hg.1	6.425	2.6837	0.000752	3.1239	GPNMB
TC1000012050.hg.1	4.8468	2.277	8.43E-08	7.0743	FGFR2
TC1000009025.hg.1	4.5237	2.1775	2.51E-07	6.6008	EMX2
TC0100015155.hg.1	4.0972	2.0346	1.31E-08	7.8838	VAV3
TC0400007799.hg.1	4.0266	2.0095	0.000164	3.7842	SLC4A4
TC0700010348.hg.1	4.007	2.0025	1.91E-06	5.7197	SOSTDC1
TC0500010043.hg.1	3.8689	1.9519	4.94E-07	6.3061	SEMA5A
TSUnmapped00000340.hg.1	3.4471	1.7854	1.23E-08	7.9107	
TSUnmapped00000845.hg.1	3.4471	1.7854	1.23E-08	7.9107	
TC0200009067.hg.1	3.3615	1.7491	0.00012	3.9223	TMEM37
TC0200015616.hg.1	3.2787	1.7131	5.94E-05	4.226	ERBB4
TC0200007418.hg.1	3.1093	1.6366	0.000173	3.7611	SLC3A1
TC1600008992.hg.1	2.9855	1.578	6.76E-05	4.17	
TC0400010242.hg.1	2.8841	1.5281	1.57E-05	4.804	PPARGC1A
TC0700008182.hg.1	2.8222	1.4968	0.005039	2.2976	
TC0200010634.hg.1	2.7475	1.4581	1.09E-07	6.9618	
TC1300006543.hg.1	2.6118	1.3851	3.41E-08	7.4675	FGF9
TC0300013282.hg.1	2.6038	1.3806	0.003741	2.4271	RNA5SP149
TC0400012621.hg.1	2.5497	1.3503	0.003615	2.4419	ACSL1
TC0200010630.hg.1	2.5444	1.3473	9.84E-05	4.0072	
TC1700010994.hg.1	2.4595	1.2983	0.000275	3.5603	
TC0100015586.hg.1	2.44	1.2869	1.73E-09	8.7614	PDZK1
TC0700006468.hg.1	2.4254	1.2782	0.003886	2.4105	
TC1600011364.hg.1	2.4108	1.2695	0.0012	2.9207	NPIPB5
TC1500007695.hg.1	2.3539	1.235	0.039202	1.4067	PAQR5
TC0200009756.hg.1	2.3176	1.2126	5.58E-06	5.2536	PKP4
TC1600010375.hg.1	2.3028	1.2034	9.43E-09	8.0254	CRNDE
TC1600011514.hg.1	2.3015	1.2026	0.002922	2.5343	SMG1P2
TC1100008018.hg.1	2.2224	1.1521	0.015263	1.8163	NEAT1
TC0600011173.hg.1	2.1903	1.1311	0.000518	3.286	GUSBP2
TSUnmapped00000154.hg.1	2.1741	1.1204	1.38E-07	6.8588	

TC0400012829.hg.1	2.1567	1.1088	2.59E-06	5.5869	ARHGEF38
TC1600011501.hg.1	2.1411	1.0983	0.001158	2.9363	NPIPB3
TC1000010567.hg.1	2.1258	1.088	0.031064	1.5077	
TC1600009929.hg.1	2.0918	1.0647	0.002389	2.6218	
TC1600009958.hg.1	2.0587	1.0417	0.001079	2.9672	LOC613037
TC0500011352.hg.1	2.0515	1.0367	0.000926	3.0333	EDIL3
TC1600011505.hg.1	2.0302	1.0216	0.000697	3.1567	NPIPB4
TSUnmapped00000267.hg.1	2.0076	1.0055	7.71E-08	7.113	
TC1600008164.hg.1	0.49984	-1.0005	5.81E-06	5.236	FAM65A
TC0600011960.hg.1	0.49897	-1.003	1.96E-06	5.7069	TNFRSF21
TC1100007844.hg.1	0.49893	-1.0031	0.000401	3.3965	ASRGL1
TC1700012073.hg.1	0.49758	-1.007	0.000109	3.9614	P4HB
TC0400010418.hg.1	0.49759	-1.007	0.000436	3.3606	RELL1
TC0100017116.hg.1	0.49717	-1.0082	5.73E-05	4.2422	C1orf116
TC0800006869.hg.1	0.49649	-1.0102	0.038739	1.4118	SLC7A2
TC1700009651.hg.1	0.49563	-1.0127	0.000291	3.5364	TP53
TC0100016112.hg.1	0.49506	-1.0143	0.000991	3.0038	TAGLN2
TC1000012428.hg.1	0.49347	-1.019	2.71E-07	6.5663	AKR1C3
TC0X00010793.hg.1	0.49317	-1.0198	2.43E-05	4.6142	ZDHHC9
TC1100011367.hg.1	0.49317	-1.0198	0.000301	3.5211	UNC93B1
TC1400008940.hg.1	0.4929	-1.0206	0.00038	3.4204	NFKBIA
TC1800008474.hg.1	0.49214	-1.0228	1.96E-07	6.707	SLC39A6
TC1000012139.hg.1	0.49177	-1.0239	7.20E-05	4.1424	CTBP2
TC0100013229.hg.1	0.49161	-1.0244	4.10E-07	6.3874	HSPG2
TC1800007431.hg.1	0.49138	-1.0251	0.012426	1.9057	MALT1
TC1900007844.hg.1	0.49088	-1.0266	0.000994	3.0028	LSR
TC0100018482.hg.1	0.49053	-1.0276	0.000881	3.0551	HIST2H2AA3
TC0700009061.hg.1	0.4902	-1.0286	1.19E-05	4.9248	CALU
TC2000008095.hg.1	0.48952	-1.0306	1.17E-06	5.9317	TPD52L2
TC0600010057.hg.1	0.48923	-1.0314	3.13E-07	6.5045	ACAT2
TC1100013040.hg.1	0.48914	-1.0317	4.79E-05	4.3201	TM7SF2
TC2200007242.hg.1	0.48913	-1.0317	0.000135	3.8692	APOL1
TC2200009257.hg.1	0.48866	-1.0331	5.06E-05	4.2957	TCN2
TC0900009489.hg.1	0.48852	-1.0335	2.32E-08	7.6342	GLDC
TC1200007686.hg.1	0.48736	-1.037	1.93E-05	4.7155	KRT18
TC1400009147.hg.1	0.48609	-1.0407	2.19E-05	4.6603	PYGL
TC2200008886.hg.1	0.48585	-1.0414	3.45E-05	4.4619	CYB5R3

TC1600007449.hg.1	0.48523	-1.0433	2.12E-05	4.673	SLX1A
TC0100015871.hg.1	0.48486	-1.0444	1.71E-05	4.7671	S100A16
TC1400009529.hg.1	0.48263	-1.051	0.000481	3.3177	ACTN1
TC0300013151.hg.1	0.48248	-1.0515	1.19E-05	4.925	NCEH1
TC1700008867.hg.1	0.48045	-1.0576	7.67E-08	7.1153	SLC9A3R1
TC1000010273.hg.1	0.47748	-1.0665	3.65E-07	6.4379	NRP1
TC2100007485.hg.1	0.47686	-1.0684	0.000101	3.9937	
TC0800010193.hg.1	0.47666	-1.069	0.000343	3.4644	TM2D2
TC0300007067.hg.1	0.47652	-1.0694	0.004089	2.3884	MYD88
TC0100010284.hg.1	0.47521	-1.0734	4.49E-06	5.3482	PEA15
TC0100011736.hg.1	0.47495	-1.0742	0.001822	2.7395	DEGS1
TC1100012992.hg.1	0.4746	-1.0752	1.20E-06	5.9207	HSD17B12
TC1900010851.hg.1	0.47327	-1.0793	0.000388	3.4107	ETHE1
TC1900009076.hg.1	0.47068	-1.0872	0.004501	2.3467	PLPP2
TC0100015696.hg.1	0.47025	-1.0885	0.001321	2.8792	HIST2H2BF
TC1800006897.hg.1	0.46998	-1.0893	0.000168	3.7741	LAMA3
TC0100009876.hg.1	0.4684	-1.0942	0.0009	3.0456	HIST2H2AA3
TC0100014193.hg.1	0.46737	-1.0974	0.018817	1.7254	TXNDC12
TC0300013417.hg.1	0.4662	-1.101	2.14E-08	7.6689	LIPH
TC1400007950.hg.1	0.46593	-1.1018	0.000942	3.026	CALM1
TC2200008592.hg.1	0.46575	-1.1024	0.000192	3.7166	APOL2
TC1700006585.hg.1	0.46509	-1.1044	0.00135	2.8697	CTNS
TC2100007316.hg.1	0.46381	-1.1084	9.64E-05	4.0159	
TC1200012859.hg.1	0.4635	-1.1094	9.54E-07	6.0202	RHOF
TC0900009274.hg.1	0.46329	-1.11	0.000127	3.8967	TUBB4B
TC0700012917.hg.1	0.46283	-1.1115	0.001787	2.7478	TPK1
TC0600011185.hg.1	0.45824	-1.1258	0.000225	3.6476	HIST1H2BK
TC1000008054.hg.1	0.45763	-1.1278	0.000203	3.6917	PLAU
TC0900006522.hg.1	0.45658	-1.1311	5.02E-07	6.299	SLC1A1
TC0800012176.hg.1	0.4565	-1.1313	3.07E-06	5.5134	PLEC
TC0900010506.hg.1	0.45652	-1.1313	1.53E-05	4.8164	KRT18P24
TC0500007114.hg.1	0.45413	-1.1388	9.12E-06	5.0401	RAI14
TC1800007014.hg.1	0.4541	-1.1389	3.78E-05	4.423	DSG2
TC0800007367.hg.1	0.45395	-1.1394	0.000949	3.0229	ADAM9
TC0X00007190.hg.1	0.45381	-1.1398	0.00642	2.1925	PORCN
TC1600010732.hg.1	0.45335	-1.1413	0.000158	3.8	NQO1
TC1700011121.hg.1	0.45286	-1.1429	1.14E-08	7.9423	TOB1

TC0100013205.hg.1	0.45218	-1.145	4.55E-05	4.342	ECE1
TC0100015544.hg.1	0.44764	-1.1596	0.002637	2.5789	HIST2H2BB
TC1900007012.hg.1	0.44609	-1.1646	0.000206	3.6865	LDLR
TC1100009514.hg.1	0.44454	-1.1696	2.07E-05	4.6831	TMEM45B
TC0900011582.hg.1	0.44094	-1.1813	7.99E-08	7.0972	FAM129B
TC1900011756.hg.1	0.43965	-1.1856	1.33E-07	6.876	PVR
TC0100007486.hg.1	0.43909	-1.1874	1.37E-08	7.8638	SFN
TC2000009903.hg.1	0.43768	-1.1921	4.57E-07	6.3402	HM13
TC1600011408.hg.1	0.43718	-1.1937	1.50E-05	4.8251	CKLF
TC0100018403.hg.1	0.43559	-1.199	9.37E-07	6.0284	ERRFI1
TC0600008120.hg.1	0.43538	-1.1997	7.72E-06	5.1127	TMEM63B
TC1600011372.hg.1	0.43497	-1.201	9.60E-06	5.0177	SULT1A4
TC1400009186.hg.1	0.43439	-1.2029	0.00141	2.8507	ERO1A
TC0700008073.hg.1	0.43344	-1.2061	0.007152	2.1456	POR
TC0700010167.hg.1	0.4307	-1.2152	0.000647	3.189	ACTB
TC1200008933.hg.1	0.42796	-1.2245	1.82E-06	5.7388	TPCN1
TC1100010156.hg.1	0.42765	-1.2255	1.39E-08	7.8564	BTBD10
TC0400007556.hg.1	0.42746	-1.2261	0.001443	2.8408	SRD5A3
TC0900007667.hg.1	0.42584	-1.2316	0.0003	3.5236	PSAT1
TC0100015598.hg.1	0.42433	-1.2368	0.000113	3.9473	TXNIP
TC1100008514.hg.1	0.42418	-1.2372	0.000572	3.2426	TSKU
TC1700008920.hg.1	0.42345	-1.2397	1.35E-10	9.8706	ITGB4
TC0900012047.hg.1	0.42159	-1.2461	0.001807	2.7431	
TC0600012040.hg.1	0.42155	-1.2462	8.17E-06	5.088	TRAM2
TC0500010835.hg.1	0.42013	-1.2511	1.92E-05	4.7166	PLK2
TC0500007050.hg.1	0.41968	-1.2526	0.009578	2.0187	PDZD2
TC1900006507.hg.1	0.41848	-1.2568	6.21E-06	5.207	CNN2
TC0200011503.hg.1	0.41792	-1.2587	3.49E-05	4.4577	PXDN
TC0700013082.hg.1	0.41709	-1.2616	2.59E-06	5.5869	PRKAG2
TC0800012147.hg.1	0.41704	-1.2617	1.49E-07	6.8272	TSTA3
TC2000008279.hg.1	0.41645	-1.2638	1.42E-05	4.8486	PCNA
TC0100018451.hg.1	0.41535	-1.2676	0.000736	3.1331	GBP2
TC1900008143.hg.1	0.41529	-1.2678	1.21E-07	6.9186	AXL
TC1100012318.hg.1	0.41418	-1.2717	1.97E-05	4.7065	IL18
TC0X00009044.hg.1	0.40965	-1.2875	0.007735	2.1115	GPR143
TC1700008228.hg.1	0.40865	-1.2911	7.28E-06	5.1377	ITGA3
TC0700006783.hg.1	0.40776	-1.2942	3.46E-06	5.4606	TSPAN13

TC1000011524.hg.1	0.40544	-1.3025	1.13E-06	5.9472	PIK3AP1
TC1700012468.hg.1	0.40305	-1.311	6.87E-05	4.1632	HN1
TC1700011097.hg.1	0.40263	-1.3125	4.28E-07	6.3685	LRRC59
TC1200008675.hg.1	0.40239	-1.3133	5.99E-09	8.2229	TXNRD1
TC0200009402.hg.1	0.4017	-1.3158	0.000251	3.6	MGAT5
TC0800008243.hg.1	0.39819	-1.3285	0.000101	3.9977	PDP1
TC0100010867.hg.1	0.39771	-1.3302	7.39E-05	4.1312	LAMC2
TC1500007833.hg.1	0.39691	-1.3331	1.04E-06	5.984	PML
TC1400006659.hg.1	0.39309	-1.3471	3.29E-05	4.4824	MMP14
TC0400012458.hg.1	0.39256	-1.349	0.001726	2.763	HPGD
TC1700011813.hg.1	0.38875	-1.3631	0.011145	1.9529	ST6GALNAC1
TC2100008527.hg.1	0.38806	-1.3656	3.80E-05	4.4204	SLC37A1
TC0600013326.hg.1	0.38424	-1.3799	1.83E-10	9.7368	PERP
TC0600008622.hg.1	0.38417	-1.3802	8.19E-05	4.0869	SH3BGRL2
TC1400007466.hg.1	0.38283	-1.3852	2.33E-05	4.6328	FUT8
TC1900006977.hg.1	0.38166	-1.3896	0.003451	2.462	ICAM1
TC0200013750.hg.1	0.37851	-1.4016	4.64E-09	8.3333	FHL2
TC1700012216.hg.1	0.37814	-1.403	7.13E-05	4.1466	LGALS9
TC1900011399.hg.1	0.37713	-1.4069	9.77E-06	5.0103	MBOAT7
TC1000008891.hg.1	0.37376	-1.4198	1.41E-08	7.8498	DUSP5
TC1700009236.hg.1	0.37329	-1.4216	8.81E-09	8.055	SLC16A3
TC2200008687.hg.1	0.37196	-1.4268	1.11E-07	6.954	TMEM184B
TC1000008236.hg.1	0.36729	-1.445	0.000253	3.5967	TSPAN14
TC0600013757.hg.1	0.36379	-1.4588	0.003136	2.5036	SOD2
TC0X00006799.hg.1	0.36262	-1.4635	6.09E-13	12.215	SAT1
TC0300013520.hg.1	0.35963	-1.4754	4.71E-07	6.3271	CLDN1
TC0100007584.hg.1	0.35964	-1.4754	6.77E-05	4.1696	PTPRU
TC1600011453.hg.1	0.35665	-1.4874	0.000203	3.6933	MC1R
TC0600011809.hg.1	0.35591	-1.4904	8.23E-09	8.0843	CCND3
TC0300011815.hg.1	0.35052	-1.5124	4.28E-06	5.3686	DCBLD2
TC0400007901.hg.1	0.3482	-1.522	0.000298	3.5252	SHROOM3
TC1900008824.hg.1	0.34732	-1.5257	0.000207	3.6847	
TC1100007273.hg.1	0.34525	-1.5343	0.000114	3.9421	CD44
TC1900008287.hg.1	0.34356	-1.5414	0.000102	3.9902	
TC0200015958.hg.1	0.34326	-1.5426	0.002907	2.5366	DNER
TC1900011382.hg.1	0.34146	-1.5502	0.005958	2.2249	
TC0800009529.hg.1	0.34073	-1.5533	0.00079	3.1024	PPP1R3B

TC0600012434.hg.1	0.33895	-1.5608	5.47E-08	7.262	ME1
TC0300006677.hg.1	0.33659	-1.5709	7.59E-08	7.12	SLC6A6
TC1200010743.hg.1	0.33483	-1.5785	0.000462	3.3355	
TC0700006629.hg.1	0.33469	-1.5791	1.73E-07	6.761	FSCN1
TC0100017167.hg.1	0.33074	-1.5962	8.76E-05	4.0576	LAMB3
TC1600011546.hg.1	0.32976	-1.6005	0.00131	2.8826	PLLP
TC1700007135.hg.1	0.3288	-1.6047	8.32E-07	6.0801	TRIM16L
TC1100012966.hg.1	0.32413	-1.6254	0.001238	2.9074	MICAL2
TC0600009597.hg.1	0.32354	-1.628	0.000156	3.8064	TNFAIP3
TC2000008315.hg.1	0.3222	-1.634	8.54E-05	4.0688	
TC0800010002.hg.1	0.3213	-1.638	6.50E-11	10.187	DUSP4
TC0700012684.hg.1	0.32023	-1.6428	4.70E-09	8.3282	AKR1B1
TC1100008236.hg.1	0.31912	-1.6478	2.63E-07	6.5799	CCND1
TC0800006975.hg.1	0.31813	-1.6523	0.000571	3.2434	BMP1
TC0600007374.hg.1	0.31744	-1.6554	0.000189	3.7234	HIST1H2AI
TC0800009961.hg.1	0.31289	-1.6763	5.90E-07	6.2288	CLU
TC0100012675.hg.1	0.31013	-1.689	8.24E-06	5.0842	ACOT7
TC0800007888.hg.1	0.30276	-1.7237	5.09E-05	4.2932	C8orf34
TC0200011138.hg.1	0.29549	-1.7588	0.000245	3.6106	UGT1A8
TC1700008719.hg.1	0.29405	-1.7659	0.000284	3.5468	KPNA2
TC1000012117.hg.1	0.29359	-1.7681	1.23E-06	5.9097	CHST15
TC0600008146.hg.1	0.29299	-1.7711	2.69E-06	5.5696	RUNX2
TC0100013028.hg.1	0.29212	-1.7754	1.18E-08	7.9299	EPHA2
TC1100011602.hg.1	0.29158	-1.778	3.59E-06	5.4446	UCP2
TC1200006896.hg.1	0.29118	-1.78	9.62E-09	8.0169	GPRC5A
TC0900008891.hg.1	0.28576	-1.8071	7.17E-06	5.1443	LRRC8A
TC1200010538.hg.1	0.28108	-1.831	5.43E-06	5.265	AMIGO2
TC0800011305.hg.1	0.28019	-1.8355	0.000138	3.8605	NCALD
TC0700011809.hg.1	0.2745	-1.8651	2.20E-07	6.6571	TFPI2
TC0100007645.hg.1	0.26936	-1.8924	5.65E-05	4.2482	
TC2200007356.hg.1	0.26814	-1.8989	3.17E-06	5.4986	KDELR3
TC0500009863.hg.1	0.26748	-1.9025	3.91E-07	6.4073	LPCAT1
TC1300007561.hg.1	0.26659	-1.9073	0.00012	3.9197	SCEL
TC1100011375.hg.1	0.26547	-1.9134	5.80E-05	4.2369	C11orf24
TC1100011259.hg.1	0.25626	-1.9643	0.000468	3.3302	FOSL1
TC0400007806.hg.1	0.25497	-1.9716	0.000111	3.953	NPFFR2
TC1100009110.hg.1	0.25447	-1.9744	3.61E-09	8.4421	NNMT

TC0300010350.hg.1	0.25146	-1.9916	8.20E-09	8.0861	WNT7A
TC0600011232.hg.1	0.25107	-1.9938	0.001114	2.9529	HIST1H1B
TC1400007584.hg.1	0.24949	-2.0029	1.90E-10	9.7207	TTC9
TC1900007465.hg.1	0.24739	-2.0151	0.000395	3.4036	ZNF486
TC0500009424.hg.1	0.24601	-2.0232	4.50E-07	6.3464	FGF18
TC2000009216.hg.1	0.24592	-2.0238	2.67E-10	9.573	SLPI
TC0300010930.hg.1	0.24203	-2.0468	0.008511	2.07	SLC6A20
TC0700010671.hg.1	0.23726	-2.0755	5.82E-08	7.2352	PDE1C
TC1100012957.hg.1	0.23052	-2.117	3.78E-10	9.4225	TRIM22
TC1500006925.hg.1	0.22674	-2.1409	2.08E-07	6.6823	THBS1
TC0500012842.hg.1	0.22595	-2.146	1.93E-08	7.7144	DUSP1
TC0100010798.hg.1	0.22564	-2.1479	9.94E-05	4.0028	QSOX1
TC0500012294.hg.1	0.22316	-2.1639	3.14E-11	10.504	PCDH1
TC1100007899.hg.1	0.22214	-2.1705	1.31E-06	5.8813	RARRES3
TC2200007312.hg.1	0.21938	-2.1885	0.000738	3.1321	LGALS1
TC2000008217.hg.1	0.2156	-2.2136	1.35E-05	4.8702	SLC4A11
TC0400009129.hg.1	0.21487	-2.2184	0.000484	3.3156	RXFP1
TC1700012274.hg.1	0.21284	-2.2322	1.71E-09	8.7664	ITGB3
TC0400012949.hg.1	0.21135	-2.2423	2.93E-07	6.5329	SLC39A8
TC0100014081.hg.1	0.20712	-2.2715	2.42E-06	5.616	PDZK1IP1
TC0400011015.hg.1	0.20651	-2.2757	1.86E-05	4.7312	CXCL3
TC0400009258.hg.1	0.19456	-2.3617	0.001522	2.8175	PALLD
TC1300009580.hg.1	0.19444	-2.3626	0.012917	1.8888	SLC15A1
TC0200008666.hg.1	0.18887	-2.4045	0.004131	2.384	IL1R1
TC0400011920.hg.1	0.18542	-2.4311	1.23E-08	7.9094	SLC7A11
TC1500010369.hg.1	0.1845	-2.4383	1.77E-06	5.7511	MFGE8
TC1900010807.hg.1	0.18441	-2.439	2.77E-06	5.557	CEACAM1
TC1500007062.hg.1	0.17982	-2.4754	0.00292	2.5346	CKMT1B
TC1700006999.hg.1	0.1749	-2.5154	4.44E-06	5.3525	ADORA2B
TC1400008677.hg.1	0.17152	-2.5435	0.004188	2.378	SLC7A7
TC1600006658.hg.1	0.17124	-2.5459	0.000145	3.8382	IL32
TC2000008316.hg.1	0.17065	-2.5509	7.27E-06	5.1385	LRRN4
TC0100013445.hg.1	0.16766	-2.5764	4.45E-11	10.352	IFI6
TC0700013394.hg.1	0.16603	-2.5905	0.000554	3.2567	CCDC146
TC1200010229.hg.1	0.16516	-2.5981	0.00017	3.7704	PTHLH
TC0700007198.hg.1	0.16375	-2.6104	0.004703	2.3277	ANLN
TC1500007067.hg.1	0.16116	-2.6334	0.002376	2.6242	CKMT1A

TC1800006448.hg.1	0.15798	-2.6622	0.000122	3.9126	TYMS
TC2100007208.hg.1	0.15792	-2.6627	9.06E-07	6.0428	MX1
TC0400010125.hg.1	0.15476	-2.6919	3.84E-06	5.4157	FGFBP1
TC1500009504.hg.1	0.15369	-2.7019	0.001087	2.9637	PYGO1
TC0600014277.hg.1	0.15129	-2.7246	0.00037	3.4323	HLA-DMA
TC0100009364.hg.1	0.14921	-2.7446	1.10E-05	4.957	CSF1
TC0500007044.hg.1	0.1482	-2.7544	2.71E-05	4.5673	CDH6
TC0400011748.hg.1	0.14583	-2.7777	0.008592	2.0659	CCNA2
TC0700012230.hg.1	0.1455	-2.7809	1.38E-06	5.8601	NRCAM
TC2000007341.hg.1	0.14068	-2.8295	0.000997	3.0014	FAM83D
TC0100016206.hg.1	0.13915	-2.8453	7.59E-05	4.1199	SH2D1B
TC0400007840.hg.1	0.13876	-2.8494	2.43E-05	4.6141	CXCL1
TC1900011233.hg.1	0.13802	-2.857	0.000387	3.4124	KLK10
TC0900007552.hg.1	0.13794	-2.8579	0.006383	2.195	GDA
TC0600011945.hg.1	0.12846	-2.9607	0.011625	1.9346	RCAN2
TC1600007235.hg.1	0.1017	-3.2976	0.007249	2.1397	PLK1
TC1900011742.hg.1	0.099967	-3.3224	0.013715	1.8628	CEACAM6
TC1200009834.hg.1	0.097926	-3.3522	2.47E-09	8.6071	MFAP5
TC1500007409.hg.1	0.097731	-3.355	7.57E-09	8.1211	GCNT3
TC0200013861.hg.1	0.094827	-3.3986	1.42E-07	6.8463	MALL
TC0900008150.hg.1	0.08967	-3.4792	1.20E-06	5.9222	TMOD1
TC0600011938.hg.1	0.087875	-3.5084	0.000246	3.6094	CLIC5
TC1200009829.hg.1	0.081115	-3.6239	6.17E-05	4.2095	CLEC4E
TC1900009559.hg.1	0.076812	-3.7025	9.65E-06	5.0153	MUC16
TC0100017084.hg.1	0.074383	-3.7489	0.004536	2.3433	CTSE
TC0300012236.hg.1	0.071818	-3.7995	0.003439	2.4636	MUC13
TC0100014988.hg.1	0.070912	-3.8178	0.001297	2.887	F3
TC0600014273.hg.1	0.066498	-3.9105	0.007165	2.1448	HLA-DRB1
TC0500007738.hg.1	0.06374	-3.9717	2.26E-05	4.646	MAP1B
TC0600014106.hg.1	0.061388	-4.0259	0.005185	2.2852	CFB
TC0800011064.hg.1	0.059141	-4.0797	0.017829	1.7489	CALB1
TC2100006834.hg.1	0.058336	-4.0995	0.008615	2.0647	LOC284825
TC2200007204.hg.1	0.053933	-4.2127	2.33E-05	4.6323	HMOX1
TC0500012470.hg.1	0.052216	-4.2594	0.005671	2.2463	CD74
TC0400007836.hg.1	0.047995	-4.381	5.49E-05	4.2607	CXCL8
TSUnmapped00000976.hg.1	0.047762	-4.388	6.00E-08	7.222	
TC1900007242.hg.1	0.04709	-4.4084	5.22E-09	8.2824	UCA1

TC0700006890.hg.1	0.042925	-4.542	3.39E-06	5.4692	IL6
TC2000009058.hg.1	0.041941	-4.5755	1.74E-06	5.7602	TGM2
TC1100013152.hg.1	0.039074	-4.6777	0.00041	3.3875	SAA2-SAA4
TC1900006814.hg.1	0.038126	-4.7131	0.006961	2.1573	
TC1100006995.hg.1	0.036336	-4.7824	0.000687	3.1632	SAA1
TC1900011741.hg.1	0.03592	-4.7991	0.002018	2.6952	CEACAM5
TC0300012768.hg.1	0.028993	-5.1082	3.15E-05	4.5021	CP
TC0600007650.hg.1	0.020653	-5.5975	0.008328	2.0794	HLA-DRA
TC0400011013.hg.1	0.01919	-5.7035	0.021238	1.6729	PPBP
TC1400010618.hg.1	0.017087	-5.8709	0.001273	2.8952	FLJ22447
TC0900008847.hg.1	0.016101	-5.9567	0.002616	2.5824	LCN2
TC0200010980.hg.1	0.015532	-6.0086	0.010691	1.971	CCL20
TC1900009443.hg.1	0.010156	-6.6215	0.000402	3.3953	C3

probe	FC	log2(FC)	raw.pval	#NAME?	Gene Symbol
TC0X00006799.hg.1	0.36262	-1.4635	6.09E-13	12.215	SAT1
TC0500012294.hg.1	0.22316	-2.1639	3.14E-11	10.504	PCDH1
TC0100013445.hg.1	0.16766	-2.5764	4.45E-11	10.352	IFI6
TC0800010002.hg.1	0.3213	-1.638	6.50E-11	10.187	DUSP4
TC1700008920.hg.1	0.42345	-1.2397	1.35E-10	9.8706	ITGB4
TC0600013326.hg.1	0.38424	-1.3799	1.83E-10	9.7368	PERP
TC1400007584.hg.1	0.24949	-2.0029	1.90E-10	9.7207	TTC9
TC2000009216.hg.1	0.24592	-2.0238	2.67E-10	9.573	SLPI
TC1100012957.hg.1	0.23052	-2.117	3.78E-10	9.4225	TRIM22
TC1700012274.hg.1	0.21284	-2.2322	1.71E-09	8.7664	ITGB3
TC0100015586.hg.1	2.44	1.2869	1.73E-09	8.7614	PDZK1
TC1200009834.hg.1	0.097926	-3.3522	2.47E-09	8.6071	MFAP5
TC1100009110.hg.1	0.25447	-1.9744	3.61E-09	8.4421	NNMT
TC0200013750.hg.1	0.37851	-1.4016	4.64E-09	8.3333	FHL2
TC0700012684.hg.1	0.32023	-1.6428	4.70E-09	8.3282	AKR1B1
TC1900007242.hg.1	0.04709	-4.4084	5.22E-09	8.2824	UCA1
TC0200009068.hg.1	11.823	3.5635	5.78E-09	8.2378	
TC1200008675.hg.1	0.40239	-1.3133	5.99E-09	8.2229	TXNRD1
TC1500007409.hg.1	0.097731	-3.355	7.57E-09	8.1211	GCNT3
TC0300010350.hg.1	0.25146	-1.9916	8.20E-09	8.0861	WNT7A
TC0600011809.hg.1	0.35591	-1.4904	8.23E-09	8.0843	CCND3
TC1700009236.hg.1	0.37329	-1.4216	8.81E-09	8.055	SLC16A3
TC1600010375.hg.1	2.3028	1.2034	9.43E-09	8.0254	CRNDE
TC1200006896.hg.1	0.29118	-1.78	9.62E-09	8.0169	GPRC5A
TC1700011121.hg.1	0.45286	-1.1429	1.14E-08	7.9423	TOB1
TC0100013028.hg.1	0.29212	-1.7754	1.18E-08	7.9299	EPHA2
TSUnmapped00000340.hg.1	3.4471	1.7854	1.23E-08	7.9107	

TSUnmapped00000845.hg.1	3.4471	1.7854	1.23E-08	7.9107	
TC0400011920.hg.1	0.18542	-2.4311	1.23E-08	7.9094	SLC7A11
TC0100015155.hg.1	4.0972	2.0346	1.31E-08	7.8838	VAV3
TC0100007486.hg.1	0.43909	-1.1874	1.37E-08	7.8638	SFN
TC1100010156.hg.1	0.42765	-1.2255	1.39E-08	7.8564	BTBD10
TC1000008891.hg.1	0.37376	-1.4198	1.41E-08	7.8498	DUSP5
TC0500012842.hg.1	0.22595	-2.146	1.93E-08	7.7144	DUSP1
TC0300013417.hg.1	0.4662	-1.101	2.14E-08	7.6689	LIPH
TC0900009489.hg.1	0.48852	-1.0335	2.32E-08	7.6342	GLDC
TC1300006543.hg.1	2.6118	1.3851	3.41E-08	7.4675	FGF9
TC0600012434.hg.1	0.33895	-1.5608	5.47E-08	7.262	ME1
TC0700010671.hg.1	0.23726	-2.0755	5.82E-08	7.2352	PDE1C
TSUnmapped00000976.hg.1	0.047762	-4.388	6.00E-08	7.222	
TC0300006677.hg.1	0.33659	-1.5709	7.59E-08	7.12	SLC6A6
TC1700008867.hg.1	0.48045	-1.0576	7.67E-08	7.1153	SLC9A3R1
TSUnmapped00000267.hg.1	2.0076	1.0055	7.71E-08	7.113	
TC0900011582.hg.1	0.44094	-1.1813	7.99E-08	7.0972	FAM129B
TC1000012050.hg.1	4.8468	2.277	8.43E-08	7.0743	FGFR2
TC0200010634.hg.1	2.7475	1.4581	1.09E-07	6.9618	
TC2200008687.hg.1	0.37196	-1.4268	1.11E-07	6.954	TMEM184B
TC1900008143.hg.1	0.41529	-1.2678	1.21E-07	6.9186	AXL
TC1900011756.hg.1	0.43965	-1.1856	1.33E-07	6.876	PVR
TSUnmapped00000154.hg.1	2.1741	1.1204	1.38E-07	6.8588	
TC0200013861.hg.1	0.094827	-3.3986	1.42E-07	6.8463	MALL
TC0800012147.hg.1	0.41704	-1.2617	1.49E-07	6.8272	TSTA3
TC0700006629.hg.1	0.33469	-1.5791	1.73E-07	6.761	FSCN1
TC1800008474.hg.1	0.49214	-1.0228	1.96E-07	6.707	SLC39A6
TC1500006925.hg.1	0.22674	-2.1409	2.08E-07	6.6823	THBS1
TC0700011809.hg.1	0.2745	-1.8651	2.20E-07	6.6571	TFPI2
TC1000009025.hg.1	4.5237	2.1775	2.51E-07	6.6008	EMX2
TC1100008236.hg.1	0.31912	-1.6478	2.63E-07	6.5799	CCND1
TC1000012428.hg.1	0.49347	-1.019	2.71E-07	6.5663	AKR1C3
TC0400012949.hg.1	0.21135	-2.2423	2.93E-07	6.5329	SLC39A8
TC0600010057.hg.1	0.48923	-1.0314	3.13E-07	6.5045	ACAT2
TC1000010273.hg.1	0.47748	-1.0665	3.65E-07	6.4379	NRP1
TC0500009863.hg.1	0.26748	-1.9025	3.91E-07	6.4073	LPCAT1
TC0100013229.hg.1	0.49161	-1.0244	4.10E-07	6.3874	HSPG2
TC1700011097.hg.1	0.40263	-1.3125	4.28E-07	6.3685	LRRC59
TC0500009424.hg.1	0.24601	-2.0232	4.50E-07	6.3464	FGF18
TC2000009903.hg.1	0.43768	-1.1921	4.57E-07	6.3402	HM13
TC0300013520.hg.1	0.35963	-1.4754	4.71E-07	6.3271	CLDN1
TC0500010043.hg.1	3.8689	1.9519	4.94E-07	6.3061	SEMA5A
TC0900006522.hg.1	0.45658	-1.1311	5.02E-07	6.299	SLC1A1

TC0800009961.hg.1	0.31289	-1.6763	5.90E-07	6.2288	CLU
TC0200015624.hg.1	6.613	2.7253	6.31E-07	6.2	
TC1700007135.hg.1	0.3288	-1.6047	8.32E-07	6.0801	TRIM16L
TC2100007208.hg.1	0.15792	-2.6627	9.06E-07	6.0428	MX1
TC0100018403.hg.1	0.43559	-1.199	9.37E-07	6.0284	ERRFI1
TC1200012859.hg.1	0.4635	-1.1094	9.54E-07	6.0202	RHOF
TC1500007833.hg.1	0.39691	-1.3331	1.04E-06	5.984	PML
TC1000011524.hg.1	0.40544	-1.3025	1.13E-06	5.9472	PIK3AP1
TC2000008095.hg.1	0.48952	-1.0306	1.17E-06	5.9317	TPD52L2
TC0900008150.hg.1	0.08967	-3.4792	1.20E-06	5.9222	TMOD1
TC1100012992.hg.1	0.4746	-1.0752	1.20E-06	5.9207	HSD17B12
TC1000012117.hg.1	0.29359	-1.7681	1.23E-06	5.9097	CHST15
TC1100007899.hg.1	0.22214	-2.1705	1.31E-06	5.8813	RARRES3
TC0700012230.hg.1	0.1455	-2.7809	1.38E-06	5.8601	NRCAM
TC2000009058.hg.1	0.041941	-4.5755	1.74E-06	5.7602	TGM2
TC1500010369.hg.1	0.1845	-2.4383	1.77E-06	5.7511	MFGE8
TC1200008933.hg.1	0.42796	-1.2245	1.82E-06	5.7388	TPCN1
TC0700010348.hg.1	4.007	2.0025	1.91E-06	5.7197	SOSTDC1
TC0600011960.hg.1	0.49897	-1.003	1.96E-06	5.7069	TNFRSF21
TC0100014081.hg.1	0.20712	-2.2715	2.42E-06	5.616	PDZK1IP1
TC0400012829.hg.1	2.1567	1.1088	2.59E-06	5.5869	ARHGEF38
TC0700013082.hg.1	0.41709	-1.2616	2.59E-06	5.5869	PRKAG2
TC0600008146.hg.1	0.29299	-1.7711	2.69E-06	5.5696	RUNX2
TC1900010807.hg.1	0.18441	-2.439	2.77E-06	5.557	CEACAM1
TC0800012176.hg.1	0.4565	-1.1313	3.07E-06	5.5134	PLEC
TC2200007356.hg.1	0.26814	-1.8989	3.17E-06	5.4986	KDELR3
TC0700006890.hg.1	0.042925	-4.542	3.39E-06	5.4692	IL6
TC0700006783.hg.1	0.40776	-1.2942	3.46E-06	5.4606	TSPAN13
TC1100011602.hg.1	0.29158	-1.778	3.59E-06	5.4446	UCP2
TC0400010125.hg.1	0.15476	-2.6919	3.84E-06	5.4157	FGFBP1
TC0300011815.hg.1	0.35052	-1.5124	4.28E-06	5.3686	DCBLD2
TC1700006999.hg.1	0.1749	-2.5154	4.44E-06	5.3525	ADORA2B
TC0100010284.hg.1	0.47521	-1.0734	4.49E-06	5.3482	PEA15
TC1200010538.hg.1	0.28108	-1.831	5.43E-06	5.265	AMIGO2
TC0200009756.hg.1	2.3176	1.2126	5.58E-06	5.2536	PKP4
TC1600008164.hg.1	0.49984	-1.0005	5.81E-06	5.236	FAM65A
TC1900006507.hg.1	0.41848	-1.2568	6.21E-06	5.207	CNN2
TC0900008891.hg.1	0.28576	-1.8071	7.17E-06	5.1443	LRRC8A
TC2000008316.hg.1	0.17065	-2.5509	7.27E-06	5.1385	LRRN4
TC1700008228.hg.1	0.40865	-1.2911	7.28E-06	5.1377	ITGA3
TC0600008120.hg.1	0.43538	-1.1997	7.72E-06	5.1127	TMEM63B
TC0600012040.hg.1	0.42155	-1.2462	8.17E-06	5.088	TRAM2
TC0100012675.hg.1	0.31013	-1.689	8.24E-06	5.0842	ACOT7

TC0500007114.hg.1	0.45413	-1.1388	9.12E-06	5.0401	RAI14
TC1600011372.hg.1	0.43497	-1.201	9.60E-06	5.0177	SULT1A4
TC1900009559.hg.1	0.076812	-3.7025	9.65E-06	5.0153	MUC16
TC1900011399.hg.1	0.37713	-1.4069	9.77E-06	5.0103	MBOAT7
TC0100009364.hg.1	0.14921	-2.7446	1.10E-05	4.957	CSF1
TC0300013151.hg.1	0.48248	-1.0515	1.19E-05	4.925	NCEH1
TC0700009061.hg.1	0.4902	-1.0286	1.19E-05	4.9248	CALU
TC2000008217.hg.1	0.2156	-2.2136	1.35E-05	4.8702	SLC4A11
TC2000008279.hg.1	0.41645	-1.2638	1.42E-05	4.8486	PCNA
TC1600011408.hg.1	0.43718	-1.1937	1.50E-05	4.8251	CKLF
TC0900010506.hg.1	0.45652	-1.1313	1.53E-05	4.8164	KRT18P24
TC0400010242.hg.1	2.8841	1.5281	1.57E-05	4.804	PPARGC1A
TC0100015871.hg.1	0.48486	-1.0444	1.71E-05	4.7671	S100A16
TC0400011015.hg.1	0.20651	-2.2757	1.86E-05	4.7312	CXCL3
TC0500010835.hg.1	0.42013	-1.2511	1.92E-05	4.7166	PLK2
TC1200007686.hg.1	0.48736	-1.037	1.93E-05	4.7155	KRT18
TC1100012318.hg.1	0.41418	-1.2717	1.97E-05	4.7065	IL18
TC1100009514.hg.1	0.44454	-1.1696	2.07E-05	4.6831	TMEM45B
TC1600007449.hg.1	0.48523	-1.0433	2.12E-05	4.673	SLX1A
TC1400009147.hg.1	0.48609	-1.0407	2.19E-05	4.6603	PYGL
TC0500007738.hg.1	0.06374	-3.9717	2.26E-05	4.646	MAP1B
TC1400007466.hg.1	0.38283	-1.3852	2.33E-05	4.6328	FUT8
TC2200007204.hg.1	0.053933	-4.2127	2.33E-05	4.6323	HMOX1
TC0X00010793.hg.1	0.49317	-1.0198	2.43E-05	4.6142	ZDHHC9
TC0400007840.hg.1	0.13876	-2.8494	2.43E-05	4.6141	CXCL1
TC0500007044.hg.1	0.1482	-2.7544	2.71E-05	4.5673	CDH6
TC0300012768.hg.1	0.028993	-5.1082	3.15E-05	4.5021	CP
TC1400006659.hg.1	0.39309	-1.3471	3.29E-05	4.4824	MMP14
TC2200008886.hg.1	0.48585	-1.0414	3.45E-05	4.4619	CYB5R3
TC0200011503.hg.1	0.41792	-1.2587	3.49E-05	4.4577	PXDN
TC1800007014.hg.1	0.4541	-1.1389	3.78E-05	4.423	DSG2
TC2100008527.hg.1	0.38806	-1.3656	3.80E-05	4.4204	SLC37A1
TC0100013205.hg.1	0.45218	-1.145	4.55E-05	4.342	ECE1
TC1100013040.hg.1	0.48914	-1.0317	4.79E-05	4.3201	TM7SF2
TC2200009257.hg.1	0.48866	-1.0331	5.06E-05	4.2957	TCN2
TC0800007888.hg.1	0.30276	-1.7237	5.09E-05	4.2932	C8orf34
TC0400007836.hg.1	0.047995	-4.381	5.49E-05	4.2607	CXCL8
TC0100007645.hg.1	0.26936	-1.8924	5.65E-05	4.2482	
TC0100017116.hg.1	0.49717	-1.0082	5.73E-05	4.2422	C1orf116
TC1100011375.hg.1	0.26547	-1.9134	5.80E-05	4.2369	C11orf24
TC0200015616.hg.1	3.2787	1.7131	5.94E-05	4.226	ERBB4
TC1200009829.hg.1	0.081115	-3.6239	6.17E-05	4.2095	CLEC4E
TC1600008992.hg.1	2.9855	1.578	6.76E-05	4.17	

TC0100007584.hg.1	0.35964	-1.4754	6.77E-05	4.1696	PTPRU
TC1700012468.hg.1	0.40305	-1.311	6.87E-05	4.1632	HN1
TC1700012216.hg.1	0.37814	-1.403	7.13E-05	4.1466	LGALS9
TC1000012139.hg.1	0.49177	-1.0239	7.20E-05	4.1424	CTBP2
TC0100010867.hg.1	0.39771	-1.3302	7.39E-05	4.1312	LAMC2
TC0100016206.hg.1	0.13915	-2.8453	7.59E-05	4.1199	SH2D1B
TC0600008622.hg.1	0.38417	-1.3802	8.19E-05	4.0869	SH3BGRL2
TC2000008315.hg.1	0.3222	-1.634	8.54E-05	4.0688	
TC0100017167.hg.1	0.33074	-1.5962	8.76E-05	4.0576	LAMB3
TC2100007316.hg.1	0.46381	-1.1084	9.64E-05	4.0159	
TC0200010630.hg.1	2.5444	1.3473	9.84E-05	4.0072	
TC0100010798.hg.1	0.22564	-2.1479	9.94E-05	4.0028	QSOX1
TC0800008243.hg.1	0.39819	-1.3285	0.000101	3.9977	PDP1
TC2100007485.hg.1	0.47686	-1.0684	0.000101	3.9937	
TC1900008287.hg.1	0.34356	-1.5414	0.000102	3.9902	
TC1700012073.hg.1	0.49758	-1.007	0.000109	3.9614	P4HB
TC0400007806.hg.1	0.25497	-1.9716	0.000111	3.953	NPFFR2
TC0100015598.hg.1	0.42433	-1.2368	0.000113	3.9473	TXNIP
TC1100007273.hg.1	0.34525	-1.5343	0.000114	3.9421	CD44
TC0200009067.hg.1	3.3615	1.7491	0.00012	3.9223	TMEM37
TC1300007561.hg.1	0.26659	-1.9073	0.00012	3.9197	SCEL
TC1800006448.hg.1	0.15798	-2.6622	0.000122	3.9126	TYMS
TC0900009274.hg.1	0.46329	-1.11	0.000127	3.8967	TUBB4B
TC2200007242.hg.1	0.48913	-1.0317	0.000135	3.8692	APOL1
TC0800011305.hg.1	0.28019	-1.8355	0.000138	3.8605	NCALD
TC1600006658.hg.1	0.17124	-2.5459	0.000145	3.8382	IL32
TC0600009597.hg.1	0.32354	-1.628	0.000156	3.8064	TNFAIP3
TC1600010732.hg.1	0.45335	-1.1413	0.000158	3.8	NQO1
TC0400007799.hg.1	4.0266	2.0095	0.000164	3.7842	SLC4A4
TC1800006897.hg.1	0.46998	-1.0893	0.000168	3.7741	LAMA3
TC1200010229.hg.1	0.16516	-2.5981	0.00017	3.7704	PTHLH
TC0200007418.hg.1	3.1093	1.6366	0.000173	3.7611	SLC3A1
TC0600007374.hg.1	0.31744	-1.6554	0.000189	3.7234	HIST1H2AI
TC2200008592.hg.1	0.46575	-1.1024	0.000192	3.7166	APOL2
TC1600011453.hg.1	0.35665	-1.4874	0.000203	3.6933	MC1R
TC1000008054.hg.1	0.45763	-1.1278	0.000203	3.6917	PLAU
TC1900007012.hg.1	0.44609	-1.1646	0.000206	3.6865	LDLR
TC1900008824.hg.1	0.34732	-1.5257	0.000207	3.6847	
TC0600011185.hg.1	0.45824	-1.1258	0.000225	3.6476	HIST1H2BK
TC0200011138.hg.1	0.29549	-1.7588	0.000245	3.6106	UGT1A8
TC0600011938.hg.1	0.087875	-3.5084	0.000246	3.6094	CLIC5
TC0200009402.hg.1	0.4017	-1.3158	0.000251	3.6	MGAT5
TC1000008236.hg.1	0.36729	-1.445	0.000253	3.5967	TSPAN14

TC1700010994.hg.1	2.4595	1.2983	0.000275	3.5603	
TC1700008719.hg.1	0.29405	-1.7659	0.000284	3.5468	KPNA2
TC1700009651.hg.1	0.49563	-1.0127	0.000291	3.5364	TP53
TC0400007901.hg.1	0.3482	-1.522	0.000298	3.5252	SHROOM3
TC0900007667.hg.1	0.42584	-1.2316	0.0003	3.5236	PSAT1
TC1100011367.hg.1	0.49317	-1.0198	0.000301	3.5211	UNC93B1
TC0800010193.hg.1	0.47666	-1.069	0.000343	3.4644	TM2D2
TC0600014277.hg.1	0.15129	-2.7246	0.00037	3.4323	HLA-DMA
TC1400008940.hg.1	0.4929	-1.0206	0.00038	3.4204	NFKBIA
TC1900011233.hg.1	0.13802	-2.857	0.000387	3.4124	KLK10
TC1900010851.hg.1	0.47327	-1.0793	0.000388	3.4107	ETHE1
TC1900007465.hg.1	0.24739	-2.0151	0.000395	3.4036	ZNF486
TC1100007844.hg.1	0.49893	-1.0031	0.000401	3.3965	ASRGL1
TC1900009443.hg.1	0.010156	-6.6215	0.000402	3.3953	C3
TC1100013152.hg.1	0.039074	-4.6777	0.00041	3.3875	SAA2-SAA4
TC0400010418.hg.1	0.49759	-1.007	0.000436	3.3606	RELL1
TC1200010743.hg.1	0.33483	-1.5785	0.000462	3.3355	
TC1100011259.hg.1	0.25626	-1.9643	0.000468	3.3302	FOSL1
TC1400009529.hg.1	0.48263	-1.051	0.000481	3.3177	ACTN1
TC0400009129.hg.1	0.21487	-2.2184	0.000484	3.3156	RXFP1
TC0600011173.hg.1	2.1903	1.1311	0.000518	3.286	GUSBP2
TC0700013394.hg.1	0.16603	-2.5905	0.000554	3.2567	CCDC146
TC0800006975.hg.1	0.31813	-1.6523	0.000571	3.2434	BMP1
TC1100008514.hg.1	0.42418	-1.2372	0.000572	3.2426	TSKU
TC0700010167.hg.1	0.4307	-1.2152	0.000647	3.189	ACTB
TC1100006995.hg.1	0.036336	-4.7824	0.000687	3.1632	SAA1
TC1600011505.hg.1	2.0302	1.0216	0.000697	3.1567	NPIPB4
TC0100018451.hg.1	0.41535	-1.2676	0.000736	3.1331	GBP2
TC2200007312.hg.1	0.21938	-2.1885	0.000738	3.1321	LGALS1
TC0700006913.hg.1	6.425	2.6837	0.000752	3.1239	GPNMB
TC0800009529.hg.1	0.34073	-1.5533	0.00079	3.1024	PPP1R3B
TC0100018482.hg.1	0.49053	-1.0276	0.000881	3.0551	HIST2H2AA3
TC0100009876.hg.1	0.4684	-1.0942	0.0009	3.0456	HIST2H2AA3
TC0500011352.hg.1	2.0515	1.0367	0.000926	3.0333	EDIL3
TC1400007950.hg.1	0.46593	-1.1018	0.000942	3.026	CALM1
TC0800007367.hg.1	0.45395	-1.1394	0.000949	3.0229	ADAM9
TC0100016112.hg.1	0.49506	-1.0143	0.000991	3.0038	TAGLN2
TC1900007844.hg.1	0.49088	-1.0266	0.000994	3.0028	LSR
TC2000007341.hg.1	0.14068	-2.8295	0.000997	3.0014	FAM83D
TC1600009958.hg.1	2.0587	1.0417	0.001079	2.9672	LOC613037
TC1500009504.hg.1	0.15369	-2.7019	0.001087	2.9637	PYGO1
TC0600011232.hg.1	0.25107	-1.9938	0.001114	2.9529	HIST1H1B
TC1600011501.hg.1	2.1411	1.0983	0.001158	2.9363	NPIPB3

TC1600011364.hg.1	2.4108	1.2695	0.0012	2.9207	NPIPB5
TC1100012966.hg.1	0.32413	-1.6254	0.001238	2.9074	MICAL2
TC1400010618.hg.1	0.017087	-5.8709	0.001273	2.8952	FLJ22447
TC0100014988.hg.1	0.070912	-3.8178	0.001297	2.887	F3
TC1600011546.hg.1	0.32976	-1.6005	0.00131	2.8826	PLLP
TC0100015696.hg.1	0.47025	-1.0885	0.001321	2.8792	HIST2H2BF
TC1700006585.hg.1	0.46509	-1.1044	0.00135	2.8697	CTNS
TC1400009186.hg.1	0.43439	-1.2029	0.00141	2.8507	ERO1A
TC0400007556.hg.1	0.42746	-1.2261	0.001443	2.8408	SRD5A3
TC0400009258.hg.1	0.19456	-2.3617	0.001522	2.8175	PALLD
TC0400012458.hg.1	0.39256	-1.349	0.001726	2.763	HPGD
TC0700012917.hg.1	0.46283	-1.1115	0.001787	2.7478	TPK1
TC0900012047.hg.1	0.42159	-1.2461	0.001807	2.7431	
TC0100011736.hg.1	0.47495	-1.0742	0.001822	2.7395	DEGS1
TC1900011741.hg.1	0.03592	-4.7991	0.002018	2.6952	CEACAM5
TC1500007067.hg.1	0.16116	-2.6334	0.002376	2.6242	CKMT1A
TC1600009929.hg.1	2.0918	1.0647	0.002389	2.6218	
TC0900008847.hg.1	0.016101	-5.9567	0.002616	2.5824	LCN2
TC0100015544.hg.1	0.44764	-1.1596	0.002637	2.5789	HIST2H2BB
TC0200015958.hg.1	0.34326	-1.5426	0.002907	2.5366	DNER
TC1500007062.hg.1	0.17982	-2.4754	0.00292	2.5346	CKMT1B
TC1600011514.hg.1	2.3015	1.2026	0.002922	2.5343	SMG1P2
TC0600013757.hg.1	0.36379	-1.4588	0.003136	2.5036	SOD2
TC0300012236.hg.1	0.071818	-3.7995	0.003439	2.4636	MUC13
TC1900006977.hg.1	0.38166	-1.3896	0.003451	2.462	ICAM1
TC0400012621.hg.1	2.5497	1.3503	0.003615	2.4419	ACSL1
TC0300013282.hg.1	2.6038	1.3806	0.003741	2.4271	RNA5SP149
TC0700006468.hg.1	2.4254	1.2782	0.003886	2.4105	
TC0300007067.hg.1	0.47652	-1.0694	0.004089	2.3884	MYD88
TC0200008666.hg.1	0.18887	-2.4045	0.004131	2.384	IL1R1
TC1400008677.hg.1	0.17152	-2.5435	0.004188	2.378	SLC7A7
TC1900009076.hg.1	0.47068	-1.0872	0.004501	2.3467	PLPP2
TC0100017084.hg.1	0.074383	-3.7489	0.004536	2.3433	CTSE
TC0700007198.hg.1	0.16375	-2.6104	0.004703	2.3277	ANLN
TC0700008182.hg.1	2.8222	1.4968	0.005039	2.2976	
TC0600014106.hg.1	0.061388	-4.0259	0.005185	2.2852	CFB
TC0500012470.hg.1	0.052216	-4.2594	0.005671	2.2463	CD74
TC1900011382.hg.1	0.34146	-1.5502	0.005958	2.2249	
TC0900007552.hg.1	0.13794	-2.8579	0.006383	2.195	GDA
TC0X00007190.hg.1	0.45381	-1.1398	0.00642	2.1925	PORCN
TC1900006814.hg.1	0.038126	-4.7131	0.006961	2.1573	
TC0700008073.hg.1	0.43344	-1.2061	0.007152	2.1456	POR
TC0600014273.hg.1	0.066498	-3.9105	0.007165	2.1448	HLA-DRB1

TC1600007235.hg.1	0.1017	-3.2976	0.007249	2.1397	PLK1
TC0X00009044.hg.1	0.40965	-1.2875	0.007735	2.1115	GPR143
TC0600007650.hg.1	0.020653	-5.5975	0.008328	2.0794	HLA-DRA
TC0300010930.hg.1	0.24203	-2.0468	0.008511	2.07	SLC6A20
TC0400011748.hg.1	0.14583	-2.7777	0.008592	2.0659	CCNA2
TC2100006834.hg.1	0.058336	-4.0995	0.008615	2.0647	LOC284825
TC0500007050.hg.1	0.41968	-1.2526	0.009578	2.0187	PDZD2
TC0200010980.hg.1	0.015532	-6.0086	0.010691	1.971	CCL20
TC1700011813.hg.1	0.38875	-1.3631	0.011145	1.9529	ST6GALNAC1
TC0600011945.hg.1	0.12846	-2.9607	0.011625	1.9346	RCAN2
TC1800007431.hg.1	0.49138	-1.0251	0.012426	1.9057	MALT1
TC1300009580.hg.1	0.19444	-2.3626	0.012917	1.8888	SLC15A1
TC1900011742.hg.1	0.099967	-3.3224	0.013715	1.8628	CEACAM6
TC1100008018.hg.1	2.2224	1.1521	0.015263	1.8163	NEAT1
TC0800011064.hg.1	0.059141	-4.0797	0.017829	1.7489	CALB1
TC0100014193.hg.1	0.46737	-1.0974	0.018817	1.7254	TXNDC12
TC0400011013.hg.1	0.01919	-5.7035	0.021238	1.6729	PPBP
TC1000010567.hg.1	2.1258	1.088	0.031064	1.5077	
TC0800006869.hg.1	0.49649	-1.0102	0.038739	1.4118	SLC7A2
TC1500007695.hg.1	2.3539	1.235	0.039202	1.4067	PAQR5

Supplementary Table 16A: Upregulated Gene Set Enrichment Analysis for significant genes between iAs+ and iAs- with nominal p-value < 0.05.

GS	Size	ES	NES	NOM p-val	FDR q-val	FWER p-val	Rank at Max	Leading Edge
REACTOME_METABOLISM_OF_LIPIDS	20	0.35	1.97	0	1	0.694	188	tags=100%, list=67%, signal=285%
REN_ALVEOLAR_RHABDOMYOSARCOMA_DN	30	0.24	1.61	0	1	1	219	tags=100%, list=78%, signal=415%
CATTTCA_MIR203	5	0.84	2.23	0	0.35	0.16	6	tags=80%, list=2%, signal=80%
TTTTGAG_MIR373	5	0.81	2.15	0	0.306	0.262	17	tags=80%, list=6%, signal=84%
HNF1_C	7	0.66	2.09	0	0.31	0.375	99	tags=100%, list=35%, signal=151%
ER_Q6_01	6	0.68	2.03	0	0.342	0.5	42	tags=83%, list=15%, signal=96%
SOX5_01	8	0.59	1.96	0	0.368	0.66	34	tags=63%, list=12%, signal=69%
CHAF1B_TARGET_GENES	21	0.29	1.63	0	0.568	0.998	204	tags=100%, list=73%, signal=344%
TGCGCANK_UNKNOWN	6	0.66	1.93	0.004	0.396	0.748	98	tags=100%, list=35%, signal=151%
MIR2052	6	0.67	1.9	0.004	0.364	0.793	22	tags=83%, list=8%, signal=89%
AMEF2_Q6	7	0.58	1.84	0.005	0.337	0.904	11	tags=57%, list=4%, signal=58%
SNRNP70_TARGET_GENES	11	0.49	1.92	0.006	0.377	0.77	146	tags=100%, list=52%, signal=202%
ZNF618_TARGET_GENES	18	0.39	2.03	0.01	0.284	0.512	155	tags=94%, list=56%, signal=199%
MULLIGHAN_NPM1_SIGNATURE_3_UP	5	0.68	1.77	0.011	1	0.985	41	tags=80%, list=15%, signal=92%
GATA4_Q3	5	0.69	1.88	0.011	0.378	0.848	89	tags=100%, list=32%, signal=144%
GREB1_TARGET_GENES	18	0.36	1.84	0.012	0.317	0.905	82	tags=67%, list=29%, signal=88%
MIR5696	7	0.54	1.73	0.014	0.503	0.983	131	tags=100%, list=47%, signal=184%
DELYS_THYROID_CANCER_DN	5	0.69	1.84	0.015	1	0.936	89	tags=100%, list=32%, signal=144%
HMGA1_TARGET_GENES	10	0.44	1.66	0.017	0.559	0.997	124	tags=90%, list=44%, signal=156%
ZNF843_TARGET_GENES	12	0.45	1.87	0.02	0.35	0.854	60	tags=67%, list=22%, signal=81%
FEV_TARGET_GENES	19	0.35	1.85	0.022	0.366	0.89	159	tags=95%, list=57%, signal=205%
ZNF8_TARGET_GENES	9	0.48	1.73	0.022	0.53	0.982	149	tags=100%, list=53%, signal=208%
MIR124_3P	17	0.32	1.6	0.024	0.607	0.999	166	tags=94%, list=59%, signal=218%
PECE_MAMMARY_STEM_CELL_DN	7	0.53	1.67	0.027	1	1	134	tags=100%, list=48%, signal=188%
CTATGCA_MIR153	5	0.68	1.84	0.027	0.359	0.904	10	tags=60%, list=4%, signal=61%
VANDESLUIS_COMMD1_TARGETS_GROUP_3_UP	8	0.49	1.65	0.032	1	1	146	tags=100%, list=52%, signal=204%
REACTOME_CONSTITUTIVE_SIGNALING_BY_ABERRANT_PI3K_IN_CANCER	5	0.58	1.6	0.032	1	1	11	tags=60%, list=4%, signal=61%
MIR670_5P	6	0.58	1.68	0.032	0.608	0.994	120	tags=100%, list=43%, signal=172%
MIR506_3P	17	0.32	1.61	0.032	0.611	0.999	166	tags=94%, list=59%, signal=218%
CACTGCC_MIR34A_MIR34C_MIR449	8	0.48	1.63	0.035	0.581	0.998	147	tags=100%, list=53%, signal=205%
DR4_Q2	6	0.56	1.67	0.036	0.591	0.997	126	tags=100%, list=45%, signal=178%
BERTUCCI_MEDULLARY_VS_DUCTAL_BREAST_CANCER_DN	7	0.52	1.65	0.038	1	1	57	tags=71%, list=20%, signal=88%
MEF2C_TARGET_GENES	11	0.41	1.59	0.038	0.596	0.999	169	tags=100%, list=61%, signal=244%
ZBTB12_TARGET_GENES	17	0.31	1.55	0.038	0.632	1	140	tags=88%, list=50%, signal=166%
STAT4_01	8	0.47	1.58	0.039	0.602	0.999	17	tags=50%, list=6%, signal=52%

HAHTOLA_MYCOSIS_FUNGOIDES_SKIN_UP	5	0.62	1.63	0.043	1	1	109	tags=100%, list=39%, signal=161%
ZWANG_CLASS_1_TRANSIENTLY_INDUCED_BY_EGF	23	0.24	1.48	0.045	0.857	1	217	tags=100%, list=78%, signal=413%
RTAAACA_FREAC2_01	16	0.32	1.54	0.045	0.622	1	13	tags=31%, list=5%, signal=31%
VANTVEER_BREAST_CANCER_ESR1_UP	7	0.52	1.58	0.047	0.74	1	59	tags=71%, list=21%, signal=88%
VDR_Q3	6	0.53	1.55	0.047	0.642	1	134	tags=100%, list=48%, signal=188%
REACTOME_METABOLISM_OF_VITAMINS_AND_COFACTORS	8	0.47	1.59	0.048	0.89	1	92	tags=88%, list=33%, signal=127%
SNIP1_TARGET_GENES	10	0.43	1.67	0.048	0.564	0.997	161	tags=100%, list=58%, signal=228%

Supplementary Table 16B: Downregulated Gene Set Enrichment Analysis for significant genes between iAs+ and iAs- with nominal p-value < 0.05.

GS	Size	ES	NES	NOM p-val	FDR q-val	FWER p-val	Rank at Max	Leading Edge
HARRIS_HYPOXIA	7	-0.89	-2.19	0	0.009	0.009	23	tags=86%, list=8%, signal=91%
GAURNIER_PSMD4_TARGETS	9	-0.81	-2.13	0	0.014	0.028	45	tags=89%, list=16%, signal=103%
WP_NETWORK_MAP_OF_SARSCOV2_SIGNALING_PATHWAY	17	-0.65	-2.03	0	0.034	0.131	76	tags=71%, list=27%, signal=91%
REACTOME_INTERLEUKIN_4_AND_INTERLEUKIN_13_SIGNALING	10	-0.73	-2	0	0.042	0.193	17	tags=50%, list=6%, signal=51%
HAN_JNK_SINGALING_DN	5	-0.91	-1.96	0	0.049	0.329	16	tags=80%, list=6%, signal=83%
JINESH_BLEBBISHIELD_VS_LIVE_CONTROL_DN	25	-0.58	-1.95	0	0.05	0.371	56	tags=48%, list=20%, signal=55%
SENESE_HDAC1_AND_HDAC2_TARGETS_UP	16	-0.64	-1.93	0	0.054	0.472	29	tags=44%, list=10%, signal=46%
REACTOME_G_ALPHA_I_SIGNALLING_EVENTS	10	-0.7	-1.91	0	0.062	0.601	15	tags=50%, list=5%, signal=51%
SMID_BREAST_CANCER_LUMINAL_B_DN	31	-0.53	-1.9	0	0.049	0.625	38	tags=39%, list=14%, signal=40%
BIOCARTA_INFLAM_PATHWAY	5	-0.86	-1.88	0	0.047	0.733	44	tags=100%, list=16%, signal=117%
WP_CYTOKINES_AND_INFLAMMATORY_RESPONSE	5	-0.86	-1.87	0	0.047	0.767	44	tags=100%, list=16%, signal=117%
SMID_BREAST_CANCER_NORMAL_LIKE_UP	12	-0.75	-2.13	0.001	0.009	0.028	30	tags=67%, list=11%, signal=71%
JINESH_BLEBBISHIELD_TO_IMMUNE_CELL_FUSION_PBSHMS_DN	27	-0.57	-1.97	0.001	0.055	0.288	54	tags=48%, list=19%, signal=54%
KEGG_CYTOKINE_CYTOKINE_RECEPTOR_INTERACTION	10	-0.72	-1.97	0.001	0.048	0.293	66	tags=80%, list=24%, signal=101%
NABA_MATRISOME_ASSOCIATED	26	-0.56	-1.91	0.001	0.068	0.58	86	tags=65%, list=31%, signal=86%
REACTOME_PEPTIDE_LIGAND_BINDING_RECEPTORS	11	-0.66	-1.9	0.001	0.056	0.615	15	tags=45%, list=5%, signal=46%
REACTOME_CLASS_A_1_RHODOPSIN_LIKE_RECEPTORS	13	-0.64	-1.9	0.001	0.051	0.62	70	tags=69%, list=25%, signal=88%
REACTOME_MHC_CLASS_II_ANTIGEN_PRESENTATION	6	-0.81	-1.89	0.001	0.048	0.671	45	tags=83%, list=16%, signal=97%

IWANAGA_CARCINOGENESIS_BY_KRAS_PTEN_UP	5	-0.85	-1.88	0.001	0.046	0.736	23	tags=80%, list=8%, signal=86%
BOQUEST_STEM_CELL_DN	10	-0.68	-1.86	0.001	0.055	0.836	45	tags=60%, list=16%, signal=69%
WP_SARSCOV2_INNATE_IMMUNITY_EVASION_AND_CELLSPECIFIC_IMMUNE_RESPONSE	6	-0.78	-1.85	0.001	0.052	0.849	66	tags=100%, list=24%, signal=128%
LIANG_SILENCED_BY_METHYLATION_2	11	-0.68	-1.93	0.002	0.06	0.47	13	tags=45%, list=5%, signal=46%
REACTOME_ADAPTIVE_IMMUNE_SYSTEM	15	-0.62	-1.91	0.002	0.064	0.585	45	tags=47%, list=16%, signal=53%
ONDER_CDH1_TARGETS_3_DN	10	-0.69	-1.9	0.002	0.048	0.639	66	tags=80%, list=24%, signal=101%
BLANCO_MELO_COVID19_BRONCHIAL_EPITHELIAL_CELLS_SARS_COV_2_INFECTION_UP	18	-0.6	-1.89	0.002	0.048	0.659	69	tags=61%, list=25%, signal=76%
REACTOME_GPCR_LIGAND_BINDING	15	-0.63	-1.89	0.002	0.048	0.693	88	tags=80%, list=32%, signal=111%
HSIAO_LIVER_SPECIFIC_GENES	9	-0.7	-1.88	0.002	0.048	0.702	20	tags=44%, list=7%, signal=46%
NABA_SECRETED_FACTORS	13	-0.62	-1.87	0.002	0.048	0.76	86	tags=77%, list=31%, signal=106%
DAZARD_RESPONSE_TO_UV_NHEK_UP	12	-0.65	-1.86	0.002	0.054	0.838	76	tags=75%, list=27%, signal=99%
BOSCO_EPITHELIAL_DIFFERENTIATION_MODULE	8	-0.73	-1.9	0.003	0.058	0.601	33	tags=63%, list=12%, signal=69%
WP_ALLOGRAFT_REJECTION	5	-0.86	-1.9	0.003	0.054	0.62	45	tags=100%, list=16%, signal=117%
WP_OVERVIEW_OF_PROINFLAMMATORY_AND_PROFIBROTIC_MEDIATORS	8	-0.74	-1.88	0.003	0.049	0.73	44	tags=75%, list=16%, signal=86%
KIM_GLIS2_TARGETS_UP	7	-0.75	-1.84	0.003	0.058	0.893	45	tags=71%, list=16%, signal=83%
BOYLAN_MULTIPLE_MYELOMA_PCA1_UP	6	-0.77	-1.82	0.003	0.067	0.928	8	tags=50%, list=3%, signal=50%
WP_MIRNAS_INVOLVEMENT_IN_THE_IMMUNE_RESPONSE_IN_SEPSIS	6	-0.77	-1.82	0.003	0.067	0.93	15	tags=50%, list=5%, signal=52%
ALCALAY_AML_BY_NPM1_LOCALIZATION_DN	6	-0.77	-1.82	0.003	0.069	0.946	49	tags=83%, list=18%, signal=99%
REACTOME_CYTOKINE_SIGNALING_IN_IMMUNE_SYSTEM	31	-0.51	-1.82	0.003	0.068	0.946	63	tags=45%, list=23%, signal=52%
BLANCO_MELO_COVID19_SARS_COV_2_INFECTION_A594_ACE2_EXPRESSING_CELLS_UP	14	-0.6	-1.8	0.003	0.077	0.969	38	tags=50%, list=14%, signal=55%
NAKAMURA_TUMOR_ZONE_PERIPHERAL_VS_CENTRAL_DN	22	-0.53	-1.78	0.003	0.087	0.991	31	tags=32%, list=11%, signal=33%
WP_OVERVIEW_OF_NANOPARTICLE_EFFECTS	5	-0.79	-1.73	0.003	0.117	1	17	tags=60%, list=6%, signal=63%
KRIEG_HYPOXIA_VIA_KDM3A	7	-0.7	-1.73	0.003	0.119	1	49	tags=71%, list=18%, signal=84%
THUM_SYSTOLIC_HEART_FAILURE_UP	17	-0.59	-1.85	0.004	0.053	0.843	28	tags=41%, list=10%, signal=43%
NABA_MATRISOME	36	-0.49	-1.79	0.004	0.085	0.985	86	tags=56%, list=31%, signal=70%
KEGG_LEISHMANIA_INFECTION	6	-0.75	-1.77	0.004	0.096	0.994	45	tags=67%, list=16%, signal=78%

REACTOME_INNATE_IMMUNE_SYSTEM	35	-0.48	-1.75	0.004	0.108	0.999	38	tags=31%, list=14%, signal=32%
MCLACHLAN_DENTAL_CARIES_UP	25	-0.5	-1.73	0.004	0.118	1	22	tags=32%, list=8%, signal=32%
WINZEN_DEGRADED_VIA_KHSRP	10	-0.64	-1.75	0.005	0.109	0.999	66	tags=70%, list=24%, signal=88%
ZSCAN2_TARGET_GENES	7	-0.72	-1.76	0.005	1	0.978	20	tags=43%, list=7%, signal=45%
MIR338_5P	7	-0.69	-1.7	0.005	1	1	13	tags=43%, list=5%, signal=44%
LIN_SILENCED_BY_TUMOR_MICROENVIRONMENT	10	-0.66	-1.83	0.006	0.063	0.912	71	tags=80%, list=25%, signal=103%
BLANCO_MELO_RESPIRATORY_SYNCYTIAL_VIRUS_INFECTION_A594_CELLS_UP	25	-0.52	-1.79	0.006	0.084	0.984	48	tags=44%, list=17%, signal=48%
BLANCO_MELO_COVID19_SARS_COV_2_POS_PATIENT_LUNG_TISSUE_UP	5	-0.8	-1.79	0.006	0.084	0.986	60	tags=100%, list=22%, signal=125%
REACTOME_INTERLEUKIN_10_SIGNALING	8	-0.69	-1.76	0.006	0.106	0.999	63	tags=75%, list=23%, signal=94%
SCHUETZ_BREAST_CANCER_DUCTAL_INVASIVE_UP	23	-0.53	-1.75	0.006	0.106	0.999	87	tags=65%, list=31%, signal=87%
KEGG_SYSTEMIC_LUPUS_ERYTHEMATOSUS	5	-0.8	-1.75	0.006	0.108	0.999	45	tags=80%, list=16%, signal=94%
VECCHI_GASTRIC_CANCER_EARLY_UP	21	-0.53	-1.72	0.006	0.125	1	71	tags=57%, list=25%, signal=71%
RIZKI_TUMOR_INVASIVENESS_3D_UP	5	-0.75	-1.64	0.006	0.179	1	49	tags=80%, list=18%, signal=95%
REACTOME_REGULATION_OF_INSULIN_LIKE_GROWTH_FACTOR_IGF_TRANSPORT_AND_UPTAKE_BY_INSULIN_LIKE_GROWTH_FACTOR_BINDING_PROTEINS_IGFBPS	10	-0.64	-1.76	0.007	0.108	0.999	75	tags=70%, list=27%, signal=92%
HALMOS_CEBPA_TARGETS_UP	5	-0.78	-1.73	0.007	0.118	1	66	tags=100%, list=24%, signal=129%
WP_COMPLEMENT_AND_COAGULATION_CASCADES	5	-0.77	-1.69	0.007	0.146	1	23	tags=60%, list=8%, signal=64%
ONDER_CDH1_SIGNALING_VIA_CTNNB1	10	-0.62	-1.69	0.007	0.149	1	38	tags=50%, list=14%, signal=56%
ZHENG_GLIOBLASTOMA_PLASTICITY_UP	14	-0.61	-1.81	0.008	0.071	0.957	119	tags=100%, list=43%, signal=166%
ONDER_CDH1_TARGETS_2_DN	41	-0.46	-1.73	0.008	0.119	1	68	tags=44%, list=24%, signal=50%
SMIRNOV_CIRCULATING_ENDOTHELIOCYTES_IN_CANCER_UP	11	-0.59	-1.67	0.008	0.159	1	17	tags=36%, list=6%, signal=37%
HALLMARK_IL6_JAK_STAT3_SIGNALING	10	-0.63	-1.73	0.008	0.232	0.201	69	tags=70%, list=25%, signal=90%
REACTOME_SIGNALING_BY_GPCR	20	-0.54	-1.77	0.009	0.094	0.992	88	tags=65%, list=32%, signal=88%
POOLA_INVASIVE_BREAST_CANCER_UP	15	-0.56	-1.7	0.009	0.135	1	57	tags=53%, list=20%, signal=63%
LY_AGING_OLD_DN	5	-0.75	-1.67	0.009	0.16	1	49	tags=80%, list=18%, signal=95%
ONDER_CDH1_TARGETS_1_DN	13	-0.6	-1.74	0.01	0.114	1	91	tags=77%, list=33%, signal=109%

KEGG_HEMATOPOIETIC_CELL_LINEAGE	8	-0.65	-1.67	0.01	0.164	1	69	tags=75%, list=25%, signal=97%
TENEDINI_MEGAKARYOCYTE_MARKERS	7	-0.71	-1.72	0.011	0.122	1	69	tags=86%, list=25%, signal=111%
CREIGHTON_ENDOCRINE_THERAPY_RESISTANCE_3	32	-0.48	-1.7	0.011	0.133	1	33	tags=31%, list=12%, signal=31%
REACTOME_SIGNALING_BY_INTERLEUKINS	22	-0.52	-1.7	0.011	0.131	1	63	tags=45%, list=23%, signal=54%
FOROUTAN_INTEGRATED_TGFB_EMT_UP	5	-0.74	-1.63	0.011	0.18	1	77	tags=100%, list=28%, signal=136%
MIR6721_5P	6	-0.71	-1.69	0.011	1	1	45	tags=67%, list=16%, signal=78%
MIR5579_3P	5	-0.77	-1.67	0.011	1	1	28	tags=60%, list=10%, signal=65%
HES2_TARGET_GENES	36	-0.45	-1.65	0.011	1	1	20	tags=22%, list=7%, signal=21%
REACTOME_CHEMOKINE_RECEPTORS_BIND_CHEMOKINES	5	-0.78	-1.73	0.012	0.118	1	66	tags=100%, list=24%, signal=129%
COLINA_TARGETS_OF_4EBP1_AND_4EBP2	17	-0.54	-1.71	0.013	0.134	1	51	tags=47%, list=18%, signal=54%
PROVENZANI_METASTASIS_DN	9	-0.63	-1.67	0.013	0.166	1	30	tags=44%, list=11%, signal=48%
WP_ANTIVIRAL_AND_ANTIINFLAMMATORY_EFFECTS_OF_NRF2_ON_SARSCOV2_PATHWAY	6	-0.69	-1.63	0.014	0.182	1	17	tags=50%, list=6%, signal=52%
YAN_ESCAPE_FROM_ANOIKIS	5	-0.75	-1.66	0.015	0.165	1	48	tags=80%, list=17%, signal=95%
ICHIBA_GRAFT_VERSUS_HOST_DISEASE_35D_UP	8	-0.65	-1.65	0.015	0.179	1	87	tags=88%, list=31%, signal=124%
BOYLAN_MULTIPLE_MYELOMA_C_D_DN	13	-0.54	-1.65	0.015	0.179	1	36	tags=38%, list=13%, signal=42%
LEE_LIVER_CANCER_E2F1_UP	5	-0.74	-1.62	0.015	0.192	1	61	tags=80%, list=22%, signal=101%
FOSTER_KDM1A_TARGETS_UP	6	-0.69	-1.65	0.016	0.179	1	48	tags=67%, list=17%, signal=79%
LIU_VAV3_PROSTATE_CARCINOGENESIS_UP	6	-0.7	-1.63	0.016	0.184	1	16	tags=50%, list=6%, signal=52%
BOQUEST_STEM_CELL_UP	7	-0.67	-1.62	0.016	0.198	1	32	tags=57%, list=11%, signal=63%
SENESE_HDAC2_TARGETS_UP	7	-0.7	-1.72	0.017	0.125	1	15	tags=43%, list=5%, signal=44%
WP_IL18_SIGNALING_PATHWAY	16	-0.54	-1.66	0.017	0.166	1	17	tags=31%, list=6%, signal=31%
KEGG_CELL_ADHESION_MOLECULES_CAMS	7	-0.64	-1.6	0.017	0.21	1	45	tags=57%, list=16%, signal=66%
DAZARD_RESPONSE_TO_UV_SCC_UP	7	-0.68	-1.67	0.018	0.161	1	66	tags=71%, list=24%, signal=91%
TONKS_TARGETS_OF_RUNX1_RUNX1T1_FUSION_HSC_UP	11	-0.59	-1.65	0.018	0.181	1	65	tags=64%, list=23%, signal=80%
REACTOME_C_TYPE_LECTIN_RECEPTORS_CLRS	6	-0.7	-1.62	0.018	0.197	1	27	tags=50%, list=10%, signal=54%
HALLMARK_COMPLEMENT	15	-0.56	-1.72	0.018	0.129	0.222	38	tags=40%, list=14%, signal=44%

REACTOME_CELL_SURFACE_INTERACTIONS_AT_THE_VASCULAR_WALL	10	-0.61	-1.69	0.019	0.145	1	69	tags=70%, list=25%, signal=90%
SABATES_COLORECTAL_ADENOMA_UP	15	-0.55	-1.67	0.02	0.167	1	66	tags=53%, list=24%, signal=66%
GAUSSMANN_MLL_AF4_FUSION_TARGETS_G_UP	11	-0.59	-1.64	0.02	0.18	1	97	tags=82%, list=35%, signal=120%
LI_AMPLIFIED_IN_LUNG_CANCER	11	-0.57	-1.62	0.02	0.191	1	33	tags=36%, list=12%, signal=40%
GROSS_HYPOXIA_VIA_ELK3_ONLY_UP	5	-0.73	-1.6	0.02	0.21	1	81	tags=100%, list=29%, signal=138%
DUTERTRE_ESTRADIOL_RESPONSE_24HR_UP	9	-0.6	-1.59	0.02	0.215	1	68	tags=67%, list=24%, signal=85%
HALLMARK_ALLOGRAFT_REJECTION	10	-0.61	-1.69	0.02	0.109	0.273	45	tags=50%, list=16%, signal=57%
HALLMARK_INTERFERON_GAMMA_RESPONSE	13	-0.55	-1.64	0.021	0.122	0.384	48	tags=46%, list=17%, signal=53%
HINATA_NFKB_TARGETS_FIBROBLAST_UP	11	-0.59	-1.63	0.022	0.184	1	38	tags=45%, list=14%, signal=51%
MARKEY_RB1_CHRONIC_LOF_DN	8	-0.63	-1.65	0.023	0.177	1	17	tags=38%, list=6%, signal=39%
ICHIBA_GRAFT_VERSUS_HOST_DISEASE_D7_UP	8	-0.63	-1.6	0.024	0.211	1	45	tags=50%, list=16%, signal=58%
SENGUPTA_NASOPHARYNGEAL_CARCINOMA_WITH_LMP1_DN	12	-0.58	-1.64	0.025	0.179	1	36	tags=50%, list=13%, signal=55%
GRAESSMANN_APOPTOSIS_BY_SERUM_DEPRIVATION_DN	10	-0.59	-1.59	0.025	0.219	1	23	tags=40%, list=8%, signal=42%
TAKEDA_TARGETS_OF_NUP98_HOXA9_FUSION_8D_UP	9	-0.61	-1.64	0.026	0.177	1	82	tags=78%, list=29%, signal=107%
SATO_SILENCED_BY_METHYLATION_IN_PANCREATIC_CANCER_1	23	-0.49	-1.64	0.026	0.176	1	63	tags=48%, list=23%, signal=57%
VERHAAK_AML_WITH_NPM1_MUTATED_UP	16	-0.52	-1.6	0.026	0.21	1	20	tags=31%, list=7%, signal=32%
GRAHAM_CML_QUIESCENT_VS_CML_DIVIDING_UP	5	-0.69	-1.57	0.026	0.235	1	66	tags=80%, list=24%, signal=103%
SENGUPTA_NASOPHARYNGEAL_CARCINOMA_WITH_LMP1_UP	9	-0.59	-1.56	0.026	0.252	1	62	tags=56%, list=22%, signal=69%
LENAOUR_DENDRITIC_CELL_MATURATION_DN	9	-0.61	-1.6	0.027	0.208	1	15	tags=33%, list=5%, signal=34%
MARTENS_TRETINOIN_RESPONSE_DN	12	-0.56	-1.59	0.027	0.214	1	49	tags=50%, list=18%, signal=58%
FOROUTAN_INTEGRATED_TGFB_EMT_DN	10	-0.59	-1.59	0.027	0.213	1	47	tags=50%, list=17%, signal=58%
BLANCO_MELO_BETA_INTERFERON_TREATED_BRONCHIAL_EPITHELIAL_CELLS_UP	28	-0.45	-1.55	0.027	0.257	1	77	tags=46%, list=28%, signal=58%
MIR545_3P	6	-0.67	-1.56	0.028	1	1	76	tags=83%, list=27%, signal=112%
KEGG_CHEMOKINE_SIGNALING_PATHWAY	7	-0.66	-1.61	0.029	0.202	1	38	tags=57%, list=14%, signal=64%
ALTEMEIER_RESPONSE_TO_LPS_WITH_MECHANICAL_VENTILATION	13	-0.54	-1.58	0.029	0.22	1	77	tags=62%, list=28%, signal=81%
FOROUTAN_TGFB_EMT_DN	12	-0.54	-1.57	0.029	0.239	1	58	tags=50%, list=21%, signal=60%

BLANCO_MELO_COVID19_SARS_COV_2_INFECTION_A594_CELLS_UP	8	-0.63	-1.62	0.03	0.193	1	27	tags=50%, list=10%, signal=54%
WINTER_HYPOXIA_METAGENE	19	-0.5	-1.61	0.03	0.202	1	23	tags=32%, list=8%, signal=32%
HINATA_NFKB_TARGETS_KERATINOCYTE_UP	13	-0.55	-1.62	0.031	0.19	1	15	tags=31%, list=5%, signal=31%
IK1_01	5	-0.72	-1.57	0.031	1	1	13	tags=40%, list=5%, signal=41%
LINDSTEDT_DENDRITIC_CELL_MATURATION_A	9	-0.59	-1.56	0.032	0.25	1	62	tags=56%, list=22%, signal=69%
LEE_LIVER_CANCER_CIPROFIBRATE_UP	5	-0.7	-1.53	0.032	0.304	1	61	tags=80%, list=22%, signal=101%
DLX2_TARGET_GENES	6	-0.67	-1.56	0.032	1	1	31	tags=50%, list=11%, signal=55%
MAHAJAN_RESPONSE_TO_IL1A_UP	8	-0.62	-1.59	0.033	0.214	1	94	tags=88%, list=34%, signal=128%
MORI_MATURE_B_LYMPHOCYTE_UP	5	-0.68	-1.5	0.034	0.332	1	45	tags=60%, list=16%, signal=70%
HALLMARK_HYPOXIA	13	-0.56	-1.62	0.035	0.12	0.444	23	tags=38%, list=8%, signal=40%
GRAESSMANN_RESPONSE_TO_MC_AND_SERUM_DEPRIVATION_UP	13	-0.55	-1.61	0.036	0.201	1	52	tags=46%, list=19%, signal=54%
TIAN_TNF_SIGNALING_VIA_NFKB	8	-0.62	-1.57	0.037	0.24	1	38	tags=50%, list=14%, signal=56%
MIR3119	8	-0.6	-1.55	0.037	1	1	101	tags=88%, list=36%, signal=133%
IGLESIAS_E2F_TARGETS_UP	7	-0.63	-1.56	0.038	0.254	1	25	tags=43%, list=9%, signal=46%
GGGNNTTTCC_NFKB_Q6_01	5	-0.69	-1.53	0.039	1	1	16	tags=40%, list=6%, signal=42%
WP_HEPATITIS_B_INFECTION	5	-0.68	-1.49	0.04	0.33	1	15	tags=40%, list=5%, signal=42%
HALLMARK_COAGULATION	13	-0.53	-1.55	0.04	0.162	0.615	109	tags=77%, list=39%, signal=120%
MCBRYAN_PUBERTAL_BREAST_4_5WK_UP	20	-0.48	-1.59	0.041	0.213	1	81	tags=55%, list=29%, signal=72%
GERY_CEBP_TARGETS	9	-0.6	-1.56	0.041	0.253	1	17	tags=33%, list=6%, signal=34%
NR1H4_TARGET_GENES	11	-0.56	-1.55	0.041	1	1	20	tags=27%, list=7%, signal=28%
ZWANG_TRANSIENTLY_UP_BY_2ND_EGF_PULSE_ONLY	25	-0.45	-1.52	0.042	0.318	1	114	tags=68%, list=41%, signal=105%
LIEN_BREAST_CARCINOMA_METAPLASTIC_VS_DUCTAL_UP	7	-0.62	-1.52	0.043	0.317	1	17	tags=43%, list=6%, signal=44%
WP_CHEMOKINE_SIGNALING_PATHWAY	5	-0.68	-1.49	0.043	0.331	1	6	tags=40%, list=2%, signal=40%
XU_HGF_SIGNALING_NOT_VIA_AKT1_48HR_UP	5	-0.68	-1.47	0.043	0.348	1	17	tags=40%, list=6%, signal=42%
CREL_01	6	-0.65	-1.49	0.044	1	1	16	tags=33%, list=6%, signal=35%
MED25_TARGET_GENES	5	-0.68	-1.49	0.044	1	1	15	tags=40%, list=5%, signal=42%

ANDERSEN_CHOLANGIOCARCINOMA_CLASS2	20	-0.48	-1.56	0.047	0.253	1	91	tags=60%, list=33%, signal=83%
GOBERT_OLIGODENDROCYTE_DIFFERENTIATION_UP	11	-0.55	-1.54	0.047	0.278	1	69	tags=64%, list=25%, signal=81%
SMID_BREAST_CANCER_BASAL_UP	43	-0.41	-1.5	0.047	0.329	1	67	tags=40%, list=24%, signal=44%
PID_REG_GR_PATHWAY	5	-0.67	-1.48	0.047	0.342	1	15	tags=40%, list=5%, signal=42%
DELYS_THYROID_CANCER_UP	42	-0.4	-1.48	0.047	0.344	1	129	tags=67%, list=46%, signal=105%
WANG_MLL_TARGETS	10	-0.57	-1.57	0.048	0.243	1	96	tags=80%, list=34%, signal=118%
SENGUPTA_NASOPHARYNGEAL_CARCINOMA_DN	18	-0.48	-1.52	0.048	0.315	1	38	tags=33%, list=14%, signal=36%
SANA_RESPONSE_TO_IFNG_UP	9	-0.57	-1.51	0.048	0.329	1	78	tags=67%, list=28%, signal=90%
MANNE_COVID19_COMBINED_COHORT_VS_HEALTHY_DONOR_PLATELETS_DN	5	-0.67	-1.5	0.048	0.333	1	40	tags=60%, list=14%, signal=69%
BLANCO_MELO_BRONCHIAL_EPITHELIAL_CELLS_INFLUENZA_A_DEL_NS1_INFECTION_DN	5	-0.69	-1.51	0.049	0.323	1	51	tags=80%, list=18%, signal=96%

Supplementary Table 17: Ingenuity Pathway Analysis of Supplementary Table 15.

© 2000-2022 QIAGEN. All rights reserved.

Ingenuity Canonical Pathways	-log(p-value)	Ratio	z-score	Molecules
Phagosome Maturation	13.1	0.177	#NUM!	ATP6V0C,ATP6V0D1,ATP6V0E1,ATP6V1E1,CTSB,CTSE,CTSZ,DYNC1H1,DYNLL1,DYNLRB1,HLA-A,HLA-B,HLA-C,HLA-DRA,HLA-DRB1,PRDX1,PRDX2,RAB5A,RAB5C,RAB7A,RAC1,TCIRG1,TUBA1A,TUBA1B,TUBA1C,TUBA4A,TUBB,TUBB4B
Remodeling of Epithelial Adherens Junctions	12.8	0.279	#NUM!	ACTB,ACTG1,ACTN1,ACTN4,ACTR2,ARPC2,CTNNB1,IQGAP1,MET,RAB5A,RAB5C,RAB7A,TUBA1A,TUBA1B,TUBA1C,TUBA4A,TUBB,TUBB4B,VCL
ILK Signaling	12.7	0.154	#NUM!	ACTB,ACTG1,ACTN1,ACTN4,ATF2,CCND1,CDC42,CFL1,CREB3,CREB5,CREBBP,CTNNB1,FLNA,HIF1A,ITGB1,ITGB3,ITGB4,ITGB8,KRT18,MUC1,MYH9,MYL6,PPP2R2A,RAC1,RELA,RHOB,RHOC,RHOF,TMSB10/TMSB4X,VCL,VIM
Hepatic Fibrosis Signaling Pathway	10.9	0.102	#NUM!	ATF2,CALM1 (includes others),CCND1,CDC42,CREB3,CREB5,CREBBP,CSNK1D,CTNNB1,CXCL8,FTL,FZD6,GNAI1,GNAI2,HIF1A,ICAM1,IL18,IL1R1,ITGA3,ITGAV,ITGB1,ITGB3,ITGB4,ITGB8,JAK1,LRP6,MYD88,MYL12A,MYL12B,MYL6,NFKBIA,PRKAG2,PRKCI,RAC1,RELA,RHOB,RHOC,RHOF,SOD2,SPP1,TGFBR2,TIMP1,WNT7A
RHOGDI Signaling	10.9	0.136	#NUM!	ACTB,ACTG1,ACTR2,ARHGAP1,ARHGDIA,ARPC2,CD44,CDC42,CDH6,CFL1,CREBBP,EZR,GNAI1,GNAI2,GNB1,ITGA3,ITGAV,ITGB1,ITGB3,ITGB4,ITGB8,MYH9,MYL12A,MYL12B,MYL6,PIP5K1A,RAC1,RHOB,RHOC,RHOF
Regulation of Actin-based Motility by Rho	10.2	0.183	#NUM!	ACTB,ACTR2,ARHGDIA,ARPC2,CDC42,CFL1,ITGA3,ITGAV,ITGB1,ITGB3,ITGB4,ITGB8,MYL12A,MYL12B,MYL6,PFN1,PIP5K1A,RAC1,RHOB,RHOC,RHOF
Sertoli Cell-Sertoli Cell Junction Signaling	10.2	0.136	#NUM!	ACTB,ACTG1,ACTN1,ACTN4,ATF2,CDC42,CLDN1,CTNNB1,F11R,ITGA3,ITGAV,ITGB1,ITGB3,ITGB4,ITGB8,JUP,MTMR2,NECTIN2,OCLN,PRKAG2,RAC1,TUBA1A,TUBA1B,TUBA1C,TUBA4A,TUBB,TUBB4B,VCL
Germ Cell-Sertoli Cell Junction Signaling	9.92	0.147	#NUM!	ACTB,ACTG1,ACTN1,ACTN4,CDC42,CFL1,CTNNB1,IQGAP1,ITGA3,ITGB1,JUP,MTMR2,NECTIN2,RAC1,RHOB,RHOC,RHOF,TGFBR2,TUBA1A,TUBA1B,TUBA1C,TUBA4A,TUBB,TUBB4B,VCL
Actin Cytoskeleton Signaling	9.79	0.123	#NUM!	ACTB,ACTG1,ACTN1,ACTN4,ACTR2,ARPC2,CDC42,CFL1,EZR,FGF18,FGF9,FLNA,IQGAP1,ITGA3,ITGAV,ITGB1,ITGB3,ITGB4,ITGB8,MYH9,MYL12A,MYL12B,MYL6,PFN1,PIP5K1A,RAC1,SSH3,TMSB10/TMSB4X,VAV3,VCL
Leukocyte Extravasation Signaling	9.44	0.135	#NUM!	ACTB,ACTG1,ACTN1,ACTN4,ARHGAP1,CD44,CDC42,CLDN1,CTNNB1,CYBA,EDIL3,EZR,F11R,GNAI1,GNAI2,ICAM1,ITGA3,ITGB1,MMP14,MMP7,MYL6,PRKCI,RAC1,TIMP1,VAV3,VCL
Clathrin-mediated Endocytosis Signaling	9.44	0.135	#NUM!	ACTB,ACTG1,ACTR2,AP2B1,APOL1,ARPC2,CDC42,CLU,FGF18,FGF9,HSPA8,ITGB1,ITGB3,ITGB4,ITGB8,LDLR,MET,MYO1E,RAB11A,RAB5A,RAB5C,RAB7A,RAC1,STAM,UBB,UBC
CLEAR Signaling Pathway	9.38	0.112	#NUM!	ATF2,ATP6V0C,ATP6V0D1,ATP6V0E1,ATP6V1E1,CD63,CREB3,CREB5,CREBBP,CTNS,CTSB,FGFR2,GBA,GNS,HIF1A,KCNIP3,NEU1,PML,PPARGC1A,PPP2R2A,PRKAG2,PRKCI,PSAP,RAB7A,SFN,TCIRG1,TGFBR2,TP53,YWHAB,YWHAH,YWHAQ,YWHAZ
Integrin Signaling	9.22	0.127	#NUM!	ACTB,ACTG1,ACTN1,ACTN4,ACTR2,ARF1,ARF4,ARPC2,CAPN1,CAPNS1,CDC42,ITGA3,ITGAV,ITGB1,ITGB3,ITGB4,ITGB8,MYL12A,MYL12B,PFN1,RAC1,RHOB,RHOC,RHOF,TSPAN1,TSPAN4,VCL
Protein Kinase A Signaling	9.13	0.0951	#NUM!	ADD1,AKAP12,ATF2,CALM1 (includes others),CREB3,CREB5,CREBBP,CTNNB1,DUSP1,DUSP3,DUSP4,DUSP5,FLNA,GNAI1,GNAI2,GNB1,GYS1,H1-5,KDELR1,KDELR2,KDELR3,MYL12A,MYL12B,MYL6,NFKBIA,PDE1C,PRKAG2,PRKCI,PTP4A1,PTPN1,PTPRU,PYGL,RELA,SFN,TGFBR2,YWHAB,YWHAH,YWHAQ,YWHAZ
Caveolar-mediated Endocytosis Signaling	8.99	0.213	#NUM!	ACTB,ACTG1,CD55,FLNA,HLA-A,HLA-B,HLA-C,ITGA3,ITGAV,ITGB1,ITGB3,ITGB4,ITGB8,PTPN1,RAB5A,RAB5C
PI3K/AKT Signaling	8.41	0.125	#NUM!	BCL2L1,CCND1,CDC37,CTNNB1,GYS1,HSP90AA1,IL1R1,INPPL1,ITGA3,ITGAV,ITGB1,ITGB3,ITGB4,ITGB8,JAK1,MCL1,NFKBIA,PPP2R2A,RELA,SFN,TP53,YWHAB,YWHAH,YWHAQ,YWHAZ
Signaling by Rho Family GTPases	8.22	0.109	#NUM!	ACTB,ACTG1,ACTR2,ARPC2,CDC42,CDH6,CFL1,EZR,GNAI1,GNAI2,GNB1,IQGAP1,ITGA3,ITGAV,ITGB1,ITGB3,ITGB4,ITGB8,MYL12A,MYL12B,MYL6,PIP5K1A,PRKCI,RAC1,RELA,RHOB,RHOC,RHOF,VIM
Osteoarthritis Pathway	8.19	0.114	#NUM!	ANXA2,ANXA5,ATF2,CREB3,CREB5,CREBBP,CTNNB1,CXCL8,FGF18,FZD6,HIF1A,IL1R1,ITGA3,ITGAV,ITGB1,ITGB3,ITGB4,ITGB8,PPARGC1A,PRKAG2,PTHLH,RAC1,RELA,RUNX2,SLC39A8,SPP1,TGFBR2
MSP-RON Signaling In Cancer Cells Pathway	7.84	0.143	#NUM!	ATF2,CCND1,CREB3,CREB5,CREBBP,CTNNB1,FLNA,HIF1A,ITGB1,ITGB4,KLK10,MET,NFKBIA,RELA,SFN,VIM,YWHAB,YWHAH,YWHAQ,YWHAZ
Axonal Guidance Signaling	7.47	0.0806	#NUM!	ACTR2,ADAM9,ARPC2,BMP1,CDC42,CFL1,DPYSL2,EPHA2,FZD6,GNAI1,GNAI2,GNB1,ITGA3,ITGAV,ITGB1,ITGB3,ITGB4,ITGB8,MET,MMP14,MMP7,MYL12A,MYL12B,MYL6,NRP1,NTN4,PFN1,PLXNB2,PRKAG2,PRKCI,RAC1,SDC2,SEMA5A,SEMA6A,TUBA1A,TUBA1B,TUBA1C,TUBA4A,TUBB,TUBB4B,WNT7A
Tumor Microenvironment Pathway	7.35	0.123	#NUM!	CCND1,CD44,CFLAR,CSF1,CXCL8,FGF18,FGF9,HIF1A,HLA-A,HLA-B,HLA-C,ICAM1,IL6,ITGB3,LGALS9,MMP14,MMP7,PLAU,RAC1,RELA,SLC2A1,SPP1
Neuroinflammation Signaling Pathway	7.12	0.0946	#NUM!	APP,ATF2,CALB1,CFLAR,CREB3,CREB5,CREBBP,CTNNB1,CXCL8,GLS,HLA-A,HLA-B,HLA-C,HLA-DMA,HLA-DRA,HLA-DRB1,HMOX1,ICAM1,IL18,IL1R1,IL6,JAK1,MFGE8,MYD88,PSENEN,RAC1,RELA,SOD2,STAT1,TGFBR2
Hypoxia Signaling in the Cardiovascular System	7.08	0.184	#NUM!	ATF2,CREB3,CREB5,CREBBP,CSNK1D,HIF1A,HSP90AA1,NFKBIA,NQO1,P4HB,TP53,UBE2H,UBE2L3,UBE2V1
Regulation of Cellular Mechanics by Calpain Protease	7.01	0.169	#NUM!	ACTN1,ACTN4,CAPN1,CAPNS1,CCNA2,CCND1,CCND3,EZR,ITGA3,ITGAV,ITGB1,ITGB3,ITGB4,ITGB8,VCL
Epithelial Adherens Junction Signaling	7	0.127	#NUM!	ACTR2,ARPC2,CDC42,CFL1,CTNNB1,IQGAP1,MET,MYL12A,MYL12B,NECTIN2,PPP2R2A,PRKAG2,RAC1,SFN,TGFBR2,VCL,YWHAB,YWHAH,YWHAQ,YWHAZ
IL-8 Signaling	6.72	0.11	#NUM!	BCL2L1,CCND1,CCND3,CDC42,CXCL1,CXCL8,GNAI1,GNAI2,GNB1,HMOX1,ICAM1,IQGAP1,ITGAV,ITGB3,LASP1,MYL12B,NFKBIA,PRKCI,RAC1,RELA,RHOB,RHOC,RHOF
Ferroptosis Signaling Pathway	6.11	0.129	#NUM!	ARF1,ARF4,CTSB,FDFT1,FTL,G3BP1,H2AC18/H2AC19,H2BC12,HMGCR,HMOX1,MACROH2A1,PRKAG2,SAT1,SLC39A8,SLC7A11,TP53,TXNRD1
Virus Entry via Endocytic Pathways	6.1	0.144	#NUM!	ACTB,ACTG1,AP2B1,CD55,CDC42,FLNA,HLA-A,HLA-B,HLA-C,ITGB1,ITGB3,ITGB4,ITGB8,PRKCI,RAC1
Semaphorin Neuronal Repulsive Signaling Pathway	5.97	0.12	#NUM!	CD44,CFL1,DPYSL2,ITGA3,ITGAV,ITGB1,ITGB3,ITGB4,ITGB8,MYL12A,MYL12B,MYL6,NRP1,PRKAG2,RAC1,SEMA5A,SEMA6A,TP53
Sirtuin Signaling Pathway	5.72	0.0887	#NUM!	ACLY,APP,CXCL8,GABARAPL1,GLS,H1-5,HIF1A,MAP1LC3B,NDUFS5,NQO1,PFKFB3,PGAM1,PGK1,PPARGC1A,RELA,SCNN1A,SLC2A1,SOD2,TP53,TUBA1A,TUBA1B,TUBA1C,TUBA4A,UCP2,VDAC1,VDAC3
Semaphorin Signaling in Neurons	5.6	0.18	#NUM!	ARHGAP1,CDC42,CFL1,DPYSL2,ITGB1,MET,NRP1,RAC1,RHOB,RHOC,RHOF
EIF2 Signaling	5.56	0.0969	#NUM!	ACTB,CCND1,EIF3C,EIF4A1,EIF4G2,HNRNPA1,PTBP1,RNA5-8SN5,RPL22,RPL23,RPL26,RPL31,RPL32,RPL34,RPL37,RPL41,RPL5,RPS15A,RPS24,RPS3A,RPS6,RPS7

Agranulocyte Adhesion and Diapedesis	5.55	0.1	#NUM!	ACTB,ACTG1,CCL20,CKLF,CLDN1,CXCL1,CXCL16,CXCL3,CXCL8,EZR,GNAI1,GNAI2,ICAM1,IL18,IL1R1,ITGB1,MMP14,MMP7,MYH9,MYL6,PPBP
Role of Tissue Factor in Cancer	5.49	0.129	#NUM!	BCL2L1,CDC42,CFL1,CSF1,CXCL1,CXCL8,F2RL1,F3,ITGA3,ITGAV,ITGB1,ITGB3,P4HB,RAC1,TP53
ERK/MAPK Signaling	5.39	0.0977	#NUM!	ATF2,CREB3,CREB5,CREBBP,DUSP1,DUSP4,ITGA3,ITGAV,ITGB1,ITGB3,ITGB4,ITGB8,PPP2R2A,PRKAG2,PRKCI,RAC1,STAT1,YWHAB,YWHAH,YWHAQ,YWHAZ
Molecular Mechanisms of Cancer	5.33	0.074	#NUM!	BCL2L1,BMP1,CCND1,CCND3,CDC42,CFLAR,CREBBP,CTNNB1,FZD6,GNAI1,GNAI2,GNB1,HIF1A,ITGA3,ITGAV,ITGB1,ITGB3,ITGB4,ITGB8,JAK1,LRP6,NFKBIA,PRKAG2,PRKCI,PSENEN,RAC1,RELA,RHOB,RHOC,RHOF,TGFBR2,TP53,WNT7A
Crosstalk between Dendritic Cells and Natural Killer Cells	5.32	0.143	#NUM!	ACTB,ACTG1,FSCN1,HLA-A,HLA-B,HLA-C,HLA-DRA,HLA-DRB1,IL18,IL6,NECTIN2,RELA,TNFSF10
NRF2-mediated Oxidative Stress Response	5.25	0.0928	#NUM!	ABCC4,ACTB,ACTG1,CREBBP,DNAJA1,DNAJB1,EPHX1,FOSL1,FTL,GSR,GSTP1,HACD3,HMOX1,HSP90AA1,NQO1,PRDX1,PRKCI,SOD2,TXN,TXNRD1,UBB,VCP
Ephrin Receptor Signaling	5.25	0.099	#NUM!	ACTR2,ARPC2,ATF2,CDC42,CFL1,CREB3,CREB5,CREBBP,EPHA2,GNAI1,GNAI2,GNB1,ITGA3,ITGAV,ITGB1,ITGB3,ITGB4,ITGB8,RAC1,SDC2
RAC Signaling	5.22	0.117	#NUM!	ACTR2,ARPC2,CD44,CDC42,CFL1,IQGAP1,ITGA3,ITGAV,ITGB1,ITGB3,ITGB4,ITGB8,PIP5K1A,PRKCI,RAC1,RELA
Paxillin Signaling	5.22	0.131	#NUM!	ACTB,ACTG1,ACTN1,ACTN4,ARF1,CDC42,ITGA3,ITGAV,ITGB1,ITGB3,ITGB4,ITGB8,RAC1,VCL
Actin Nucleation by ARP-WASP Complex	5.21	0.14	#NUM!	ACTR2,ARPC2,CDC42,ITGA3,ITGAV,ITGB1,ITGB3,ITGB4,ITGB8,RAC1,RHOB,RHOC,RHOF
AMPK Signaling	5.11	0.0909	#NUM!	ACTB,ATF2,CCNA2,CCND1,CREB3,CREB5,CREBBP,GNAI1,GNAI2,GNB1,GYS1,HMGCR,PFKFB3,PFKP,PPARGC1A,PPP2R2A,PRKAG2,RAB11A,RAB6A,RAB7A,RAB8A,SLC2A1
Tight Junction Signaling	4.88	0.101	#NUM!	ACTB,ACTG1,CASK,CDC42,CLDN1,CTNNB1,F11R,MYH9,MYL6,NECTIN2,OCLN,PPP2R2A,PRKAG2,PRKCI,RAC1,RELA,TGFBR2,VCL
Role Of Osteoclasts In Rheumatoid Arthritis Signaling Pathway	4.85	0.0812	#NUM!	ADAM9,ATF2,CALM1 (includes others),CCND1,CDC42,COL4A2,CREB3,CREB5,CREBBP,CSF1,CTNNB1,IL18,IL1R1,ITGB3,MMP14,MMP7,NFKBIA,PTPN1,RAC1,RELA,RHOB,RHOC,RHOF,SPP1,VAV3
Colorectal Cancer Metastasis Signaling	4.82	0.0849	#NUM!	BCL2L1,CCND1,CDC42,CTNNB1,FZD6,GNAI1,GNAI2,GNB1,IL6,JAK1,LRP6,MMP14,MMP7,PRKAG2,RAC1,RELA,RHOB,RHOC,RHOF,STAT1,TGFBR2,TP53,WNT7A
Sumoylation Pathway	4.72	0.126	#NUM!	ARHGDIA,CDC42,CREBBP,CTBP2,NFKBIA,PCNA,PML,RAC1,RAN,RHOB,RHOC,RHOF,TP53
Antigen Presentation Pathway	4.65	0.205	#NUM!	CD74,HLA-A,HLA-B,HLA-C,HLA-DMA,HLA-DRA,HLA-DRB1,TAPBP
Macropinocytosis Signaling	4.65	0.145	#NUM!	ACTN4,CDC42,CSF1,ITGB1,ITGB3,ITGB4,ITGB8,MET,PRKCI,RAB5A,RAC1
Multiple Sclerosis Signaling Pathway	4.64	0.0901	#NUM!	C3,CAPN1,CAPNS1,CXCL1,CXCL8,DUSP1,HLA-A,HLA-B,HLA-C,HLA-DMA,HLA-DRA,HLA-DRB1,IL18,IL6,PARP14,PLAU,RNF213,SLC8B1,STAT1,TNFSF10
Superpathway of Cholesterol Biosynthesis	4.62	0.241	#NUM!	ACAT2,DHCR24,DHCR7,FDFT1,HMGCR,MSMO1,TM7SF2
Granulocyte Adhesion and Diapedesis	4.56	0.0952	#NUM!	CCL20,CKLF,CLDN1,CXCL1,CXCL16,CXCL3,CXCL8,EZR,GNAI1,GNAI2,ICAM1,IL18,IL1R1,ITGB1,MMP14,MMP7,PPBP,SDC2
HIF1α Signaling	4.52	0.0913	#NUM!	CREBBP,ELOC,HIF1A,HMOX1,HSP90AA1,HSPA8,IL6,MET,MMP14,MMP7,PKM,PRKCI,RAC1,RAN,RPS6,SAT1,SLC2A1,TP53,VIM
Cholesterol Biosynthesis I	4.52	0.385	#NUM!	DHCR24,DHCR7,FDFT1,MSMO1,TM7SF2
Cholesterol Biosynthesis II (via 24,25-dihydrolanosterol)	4.52	0.385	#NUM!	DHCR24,DHCR7,FDFT1,MSMO1,TM7SF2
Cholesterol Biosynthesis III (via Desmosterol)	4.52	0.385	#NUM!	DHCR24,DHCR7,FDFT1,MSMO1,TM7SF2
Fcγ Receptor-mediated Phagocytosis in Macrophages and Monocytes	4.45	0.128	#NUM!	ACTB,ACTG1,ACTR2,ARPC2,CDC42,EZR,HMOX1,PIP5K1A,PRKCI,RAB11A,RAC1,VAV3
Pulmonary Fibrosis Idiopathic Signaling Pathway	4.44	0.0767	#NUM!	ACTB,ACTG1,ATF2,AXL,CCND1,COL4A2,CREBBP,CTNNB1,FGF18,FGF9,FGFR2,FZD6,IL6,ITGAV,ITGB1,MMP14,MMP7,MUC1,PLAU,RELA,TGFBR2,THBS1,TP53,VIM,WNT7A
mTOR Signaling	4.35	0.0888	#NUM!	CDC42,EIF3C,EIF4A1,EIF4B,EIF4G2,HIF1A,HMOX1,PPP2R2A,PRKAG2,PRKCI,RAC1,RHOB,RHOC,RHOF,RPS15A,RPS24,RPS3A,RPS6,RPS7
TR/RXR Activation	4.23	0.131	#NUM!	AKR1C3,ENO1,HIF1A,LDLR,ME1,PFKP,PPARGC1A,RCAN2,SLC16A3,SLC2A1,UCP2
Coronavirus Replication Pathway	4.18	0.178	#NUM!	HNRNPA1,IFITM1,TUBA1A,TUBA1B,TUBA1C,TUBA4A,TUBB,TUBB4B
PTEN Signaling	4.14	0.1	#NUM!	BCL2L1,CCND1,CDC42,FGFR2,INPPL1,ITGA3,ITGAV,ITGB1,ITGB3,ITGB4,ITGB8,RAC1,RELA,TGFBR2,YWHAH
Coronavirus Pathogenesis Pathway	4.12	0.0882	#NUM!	ADAM9,CCND1,CXCL8,HIF1A,IL6,JAK1,NFKBIA,RAB7A,RELA,RPS15A,RPS24,RPS3A,RPS6,RPS7,STAT1,STING1,TGFBR2,TP53
Glioma Invasiveness Signaling	4.07	0.137	#NUM!	CD44,CDC42,ITGAV,ITGB3,PLAU,RAC1,RHOB,RHOC,RHOF,TIMP1
Role of IL-17F in Allergic Inflammatory Airway Diseases	4.04	0.17	#NUM!	ATF2,CREB3,CREB5,CREBBP,CXCL1,CXCL8,IL6,RELA
Macrophage Classical Activation Signaling Pathway	4.03	0.0899	#NUM!	CCL20,CXCL8,GBP2,HIF1A,HLA-DMA,HLA-DRA,HLA-DRB1,IL18,IL6,JAK1,MYD88,NFKBIA,PARP14,PKM,RELA,STAT1,TNFSF10
ERK5 Signaling	4.02	0.135	#NUM!	ATF2,CREB3,CREB5,CREBBP,FOSL1,SFN,YWHAB,YWHAH,YWHAQ,YWHAZ
Hepatic Fibrosis / Hepatic Stellate Cell Activation	3.89	0.0876	#NUM!	COL4A2,CSF1,CXCL3,CXCL8,ECE1,FGFR2,ICAM1,IL1R1,IL6,KLF6,MET,MYH9,MYL6,RELA,STAT1,TGFBR2,TIMP1
Role of Macrophages, Fibroblasts and Endothelial Cells in Rheumatoid Arthritis	3.89	0.0723	#NUM!	ATF2,CALM1 (includes others),CCND1,CREB3,CREB5,CREBBP,CSF1,CSNK1D,CTNNB1,CXCL8,F2RL1,FZD6,ICAM1,IL18,IL1R1,IL32,IL6,LRP6,MYD88,NFKBIA,PRKCI,RAC1,RELA,WNT7A
Wound Healing Signaling Pathway	3.87	0.0794	#NUM!	COL4A2,CXCL8,FGFR2,IL18,IL1R1,IL6,ITGA3,ITGB1,ITGB4,JAK1,LAMA3,LAMB3,LAMC1,LAMC2,NFKBIA,RAC1,STAT1,TGFBR2,TNFSF10,VIM
RHOA Signaling	3.87	0.105	#NUM!	ACTB,ACTG1,ACTR2,ANLN,ARHGAP1,ARPC2,CFL1,EZR,MYL12A,MYL12B,MYL6,PFN1,PIP5K1A
Glycolysis I	3.82	0.222	#NUM!	ENO1,GAPDH,PFKP,PGAM1,PGK1,PKM
14-3-3-mediated Signaling	3.77	0.102	#NUM!	PRKCI,SFN,TUBA1A,TUBA1B,TUBA1C,TUBA4A,TUBB,TUBB4B,VIM,YWHAB,YWHAH,YWHAQ,YWHAZ
Estrogen Receptor Signaling	3.65	0.066	#NUM!	ATF2,CCND1,CFL1,CREB3,CREB5,CREBBP,CTBP2,GNAI1,GNAI2,GNB1,HIF1A,HSP90AA1,JAK1,MMP14,MMP7,MYL12A,MYL12B,MYL6,NDUFS5,PCNA,PPARGC1A,PRKAG2,PRKCI,RELA,RUNX2,SOD2,TP53
p53 Signaling	3.62	0.112	#NUM!	BCL2L1,CCND1,CSNK1D,CTNNB1,HIF1A,PCNA,PERP,PML,SFN,THBS1,TP53
HGF Signaling	3.6	0.0985	#NUM!	ATF2,CCND1,CDC42,IL6,ITGA3,ITGAV,ITGB1,ITGB3,ITGB4,ITGB8,MET,PRKCI,RAC1
PAK Signaling	3.53	0.103	#NUM!	CDC42,CFL1,ITGA3,ITGAV,ITGB1,ITGB3,ITGB4,ITGB8,MYL12A,MYL12B,MYL6,RAC1

p70S6K Signaling	3.5	0.0963	#NUM!	BCAP31,F2RL1,GNAI1,GNAI2,JAK1,PPP2R2A,PRKCI,RPS6,SFN,YWHAB,YWHAH,YWHAQ,YWHAZ
WNT/β-catenin Signaling	3.42	0.0862	#NUM!	CCND1,CD44,CREBBP,CSNK1D,CTNNB1,FZD6,GJA1,LRP6,MMP7,PPP2R2A,TGFBR2,TP53,UBB,UBC,WNT7A
Glucocorticoid Receptor Signaling	3.41	0.0583	#NUM!	ACTB,ANXA1,BCL2L1,CREBBP,CXCL3,CXCL8,DUSP1,GJA1,HLA-A,HLA-B,HLA-C,HLA-DMA,HLA-DRA,HLA-DRB1,HSP90AA1,HSPA8,ICAM1,IL1R1,IL6,JAK1,KRT18,KRT19,KRT7,KRT8,NDUFS5,NFKBIA,PLAU,PRKAG2,RAC1,RELA,SLPI,STAT1,TGFBR2,YWHAH
Ephrin B Signaling	3.41	0.125	#NUM!	CAP1,CDC42,CFL1,CTNNB1,GNAI1,GNAI2,GNB1,RAC1,VAV3
Iron homeostasis signaling pathway	3.41	0.0942	#NUM!	ACO1,ATP6V0C,ATP6V0D1,ATP6V0E1,ATP6V1E1,BMP1,CP,FTL,HIF1A,HMOX1,IL6,JAK1,TCIRG1
Th1 Pathway	3.36	0.0984	#NUM!	HLA-A,HLA-B,HLA-DMA,HLA-DRA,HLA-DRB1,ICAM1,IL18,IL6,JAK1,LGALS9,PSENEN,STAT1
Gap Junction Signaling	3.3	0.0808	#NUM!	ACTB,ACTG1,CSNK1D,CTNNB1,DBN1,GJA1,GNAI1,GNAI2,PRKAG2,PRKCI,TUBA1A,TUBA1B,TUBA1C,TUBA4A,TUBB,TUBB4B
Pulmonary Healing Signaling Pathway	3.28	0.0804	#NUM!	CCND1,CDC42,CFL1,CTNNB1,FGFR2,FZD6,HIF1A,MMP14,MMP7,MYD88,NFKBIA,PRKAG2,PRKCI,RAC1,THBS1,WNT7A
Regulation of eIF4 and p70S6K Signaling	3.24	0.0829	#NUM!	EIF3C,EIF4A1,EIF4G2,ITGA3,ITGAV,ITGB1,ITGB3,ITGB4,ITGB8,PPP2R2A,RPS15A,RPS24,RPS3A,RPS6,RPS7
iNOS Signaling	3.23	0.149	#NUM!	CALM1 (includes others),CREBBP,JAK1,MYD88,NFKBIA,RELA,STAT1
Endocannabinoid Developing Neuron Pathway	3.2	0.0945	#NUM!	ATF2,CCND1,CREB3,CREB5,CREBBP,CTNNB1,GNAI1,GNAI2,GNB1,MGLL,PRKAG2,RAC1
PFKFB4 Signaling Pathway	3.17	0.146	#NUM!	ATF2,CREB3,CREB5,CREBBP,PRKAG2,TKT,TP53
Role of Osteoblasts, Osteoclasts and Chondrocytes in Rheumatoid Arthritis	3.16	0.0759	#NUM!	BMP1,CALM1 (includes others),CSF1,CSNK1D,CTNNB1,FZD6,IL18,IL1R1,IL6,ITGB3,LRP6,MMP14,NFKBIA,RELA,RUNX2,SPP1,WNT7A
Role of IL-17A in Psoriasis	3.14	0.286	#NUM!	CCL20,CXCL1,CXCL3,CXCL8
Renal Cell Carcinoma Signaling	3.11	0.114	#NUM!	CDC42,CREBBP,ELOC,HIF1A,MET,RAC1,SLC2A1,UBB,UBC
Interferon Signaling	3.1	0.167	#NUM!	IFI6,IFITM1,JAK1,MX1,RELA,STAT1
Cell Cycle: G2/M DNA Damage Checkpoint Regulation	3.06	0.14	#NUM!	PLK1,SFN,TP53,YWHAB,YWHAH,YWHAQ,YWHAZ
FAT10 Cancer Signaling Pathway	3.06	0.14	#NUM!	CTNNB1,IL6,NFKBIA,PCNA,RELA,TGFBR2,TP53
Prostate Cancer Signaling	3.06	0.0965	#NUM!	ATF2,CCND1,CREB3,CREB5,CREBBP,CTNNB1,GSTP1,HSP90AA1,NFKBIA,RELA,TP53
Gαq Signaling	3.04	0.0824	#NUM!	CALM1 (includes others),CDC42,GNAI1,GNAI2,GNB1,GYS1,HMOX1,NFKBIA,PRKCI,RAC1,RELA,RHOB,RHOC,RHOF
Production of Nitric Oxide and Reactive Oxygen Species in Macrophages	3	0.0785	#NUM!	APOL1,CDC42,CLU,CREBBP,CYBA,JAK1,NFKBIA,PPP2R2A,PRKCI,RAC1,RELA,RHOB,RHOC,RHOF,STAT1
Neuregulin Signaling	2.97	0.094	#NUM!	ERBB4,ERRFI1,HSP90AA1,ITGA3,ITGAV,ITGB1,ITGB3,ITGB4,ITGB8,PRKCI,RPS6
Triacylglycerol Biosynthesis	2.96	0.135	#NUM!	DGAT1,ELOVL1,GPAT4,LPCAT1,MBOAT7,PLPP2,PORCN
Inhibition of Matrix Metalloproteases	2.91	0.154	#NUM!	HSPG2,MMP14,MMP7,SDC2,TFPI2,TIMP1
MSP-RON Signaling In Macrophages Pathway	2.9	0.0924	#NUM!	ATF2,CREB3,CREB5,CREBBP,HLA-DMA,HLA-DRA,HLA-DRB1,KLK10,RELA,SBNO2,STAT1
Gluconeogenesis I	2.88	0.185	#NUM!	ENO1,GAPDH,ME1,PGAM1,PGK1
Agrin Interactions at Neuromuscular Junction	2.87	0.116	#NUM!	ACTB,ACTG1,CDC42,DAG1,ERBB4,ITGB1,LAMC1,RAC1
Natural Killer Cell Signaling	2.85	0.0758	#NUM!	CDC42,CFL1,HLA-A,HLA-B,HLA-C,HSPA8,IL18,ITGB1,MYD88,NECTIN2,PVR,RAC1,RELA,TNFSF10,VAV3
FGF Signaling	2.84	0.105	#NUM!	ATF2,CREB3,CREB5,CREBBP,FGF18,FGF9,FGFR2,MET,RAC1
Calcium Signaling	2.83	0.0731	#NUM!	ASPH,ATF2,ATP2A2,ATP2B4,CALM1 (includes others),CREB3,CREB5,CREBBP,MYH9,MYL6,PRKAG2,RCAN2,SLC8B1,TPM1,TPM3,TPM4
Role Of Osteoblasts In Rheumatoid Arthritis Signaling Pathway	2.83	0.0708	#NUM!	CSNK1D,CTNNB1,CTSB,CTSE,CTSZ,CXCL8,FZD6,IL18,IL6,JAK1,LRP6,MMP14,MMP7,RUNX2,STAT1,TNFSF10,WNT7A
Pathogen Induced Cytokine Storm Signaling Pathway	2.82	0.0618	#NUM!	C3,CCL20,CKLF,COL4A2,CXCL1,CXCL16,CXCL3,CXCL8,FTL,HLA-DMA,HLA-DRA,HLA-DRB1,HMGCR,IL18,IL1R1,IL6,JAK1,MYD88,PPBP,SLC2A1,STAT1,STING1,TNFSF10
LXR/RXR Activation	2.79	0.0894	#NUM!	APOL1,C3,CLU,FDFT1,HMGCR,IL18,IL1R1,IL6,LDLR,RELA,SAA1
ID1 Signaling Pathway	2.78	0.0746	#NUM!	APP,BCL2L1,CCND1,CREBBP,CTNNB1,FGFR2,HIF1A,IL6,PLXNB2,RHOC,TGFBR2,TGM2,TP53,TYMS,VIM
HOTAIR Regulatory Pathway	2.74	0.0798	#NUM!	CD44,CREBBP,CTNNB1,ICAM1,MET,MMP14,MMP7,NFKBIA,RELA,RHOC,SPP1,VIM,WNT7A
Role of IL-17A in Arthritis	2.72	0.123	#NUM!	ATF2,CCL20,CXCL1,CXCL3,CXCL8,NFKBIA,RELA
Synaptogenesis Signaling Pathway	2.65	0.0635	#NUM!	ACTR2,AP2B1,ARPC2,ATF2,CALM1 (includes others),CDC42,CDH6,CFL1,CREB3,CREB5,CREBBP,CTNNB1,EPHA2,HSPA8,MAP1B,PRKAG2,RAB5A,RAB5C,RAC1,THBS1
Endocannabinoid Cancer Inhibition Pathway	2.65	0.0816	#NUM!	ATF2,CCND1,CCND3,CREB3,CREB5,CREBBP,CTNNB1,GNAI1,GNAI2,HIF1A,PRKAG2,VIM
γ-linolenate Biosynthesis II (Animals)	2.6	0.211	#NUM!	ACSL1,CYB5R3,FADS1,FADS2
Ribonucleotide Reductase Signaling Pathway	2.58	0.0765	#NUM!	ACTB,ATF2,CCND1,CREB3,CREB5,CREBBP,CTNNB1,HIF1A,IL6,MET,PARP14,THBS1,TP53
fMLP Signaling in Neutrophils	2.57	0.084	#NUM!	ACTR2,ARPC2,CALM1 (includes others),CDC42,GNAI1,GNAI2,GNB1,NFKBIA,PRKCI,RAC1,RELA
Atherosclerosis Signaling	2.57	0.084	#NUM!	APOL1,CLU,CSF1,CXCL8,F3,ICAM1,IL18,IL6,PLAAT4,RELA,TNFRSF12A
TREM1 Signaling	2.57	0.104	#NUM!	CXCL3,CXCL8,ICAM1,IL18,IL6,ITGB1,MYD88,RELA
Regulation Of The Epithelial Mesenchymal Transition By Growth Factors Pathway	2.54	0.0729	#NUM!	CDC42,FGF18,FGF9,FGFR2,ID2,IL6,JAK1,MET,OCLN,RAC1,RELA,TGFBR2,TNFSF10,VIM

Factors Promoting Cardiogenesis in Vertebrates	2.53	0.0789	#NUM!	ATF2,BMP1,CCND1,CREB3,CREB5,CREBBP,CTNNB1,FZD6,LRP6,PRKCI,TGFBR2,WNT7A
Th1 and Th2 Activation Pathway	2.53	0.0756	#NUM!	HLA-A,HLA-B,HLA-DMA,HLA-DRA,HLA-DRB1,ICAM1,IL18,IL6,JAK1,LGALS9,PSENEN,STAT1,TGFBR2
P2Y Purigenic Receptor Signaling Pathway	2.52	0.0827	#NUM!	ATF2,CREB3,CREB5,CREBBP,GNAI1,GNAI2,GNB1,ITGB3,PRKAG2,PRKCI,RELA
Calcium Transport I	2.51	0.3	#NUM!	ANXA5,ATP2A2,ATP2B4
Regulation of the Epithelial-Mesenchymal Transition Pathway	2.48	0.0718	#NUM!	CTNNB1,FGF18,FGF9,FGFR2,FZD6,HIF1A,ID2,JAK1,MET,PSENEN,PYGO1,RELA,TGFBR2,WNT7A
Huntington's Disease Signaling	2.45	0.0636	#NUM!	ATF2,BCL2L1,CAPN1,CAPNS1,CREB3,CREB5,CREBBP,DNAJB1,GLS,GNB1,HSPA8,PRKCI,PSMB2,PSMD11,TGM2,TP53,UBB,UBC
Cardiac Hypertrophy Signaling	2.45	0.0651	#NUM!	ATF2,CALM1 (includes others),CDC42,CREBBP,GNAI1,GNAI2,GNB1,IL6,MYL12A,MYL12B,MYL6,PRKAG2,RAC1,RHOB,RHOC,RHOF,TGFBR2
Cardiac Hypertrophy Signaling (Enhanced)	2.45	0.0535	#NUM!	ATF2,ATP2A2,CALM1 (includes others),CTNNB1,CXCL8,FGF18,FGF9,FGFR2,FZD6,GNAI1,GNAI2,GNB1,IL18,IL1R1,IL6,ITGA3,ITGAV,ITGB1,ITGB3,ITGB4,ITGB8,PDE1C,PRKAG2,PRKCI,RELA,RPS6,TGFBR2,TNFSF10,WNT7A
Necroptosis Signaling Pathway	2.44	0.0769	#NUM!	AXL,CAPN1,CAPNS1,CFLAR,JAK1,PYGL,STAT1,TNFSF10,TP53,UBC,VDAC1,VDAC3
Erythropoietin Signaling Pathway	2.42	0.0734	#NUM!	BCL2L1,CCND1,CTNNB1,CXCL8,HIF1A,IL18,IL6,NFKBIA,PRKCI,RAC1,RELA,TNFSF10,TP53
Inhibition of Angiogenesis by TSP1	2.42	0.147	#NUM!	HSPG2,SDC2,TGFBR2,THBS1,TP53
Airway Pathology in Chronic Obstructive Pulmonary Disease	2.41	0.0847	#NUM!	CCL20,CXCL1,CXCL3,CXCL8,FGF18,FGF9,IL18,IL6,LCN2,TNFSF10
BEX2 Signaling Pathway	2.4	0.0976	#NUM!	CCND1,CTNNB1,HIF1A,LGALS1,NFKBIA,PPP2R2A,RELA,SPP1
Pentose Phosphate Pathway	2.39	0.273	#NUM!	PGD,TALDO1,TKT
p38 MAPK Signaling	2.36	0.0833	#NUM!	ATF2,CREB3,CREB5,CREBBP,DUSP1,IL18,IL1R1,STAT1,TGFBR2,TP53
MYC Mediated Apoptosis Signaling	2.35	0.12	#NUM!	BCL2L1,CFLAR,CREBBP,MCL1,PRKAG2,TP53
CDX Gastrointestinal Cancer Signaling Pathway	2.34	0.0693	#NUM!	BMP1,CCND1,CTNNB1,CXCL8,FZD6,IL18,IL6,JAK1,KLF5,RAC1,RELA,SLC15A1,TNFSF10,WNT7A
BAG2 Signaling Pathway	2.33	0.0952	#NUM!	ANXA2,CTSB,HSP90AA1,HSPA8,PSMB2,PSMD11,RELA,TP53
Role Of Chondrocytes In Rheumatoid Arthritis Signaling Pathway	2.33	0.078	#NUM!	CFLAR,CXCL8,IL18,IL1R1,IL6,ITGB1,MMP14,MMP7,PLAU,PRKAG2,RUNX2
IL-17A Signaling in Airway Cells	2.32	0.104	#NUM!	CCL20,CXCL1,CXCL3,IL6,JAK1,NFKBIA,RELA
Stearate Biosynthesis I (Animals)	2.32	0.104	#NUM!	ACOT7,ACSL1,DHCR24,ELOVL1,MBOAT7,PORCN,SRD5A3
Amyloid Processing	2.31	0.118	#NUM!	APP,CAPN1,CAPNS1,CSNK1D,PRKAG2,PSENEN
Vitamin-C Transport	2.29	0.174	#NUM!	LRRC8A,SLC2A1,TXN,TXNRD1
IL-15 Production	2.29	0.0813	#NUM!	AXL,EPHA2,ERBB4,FGFR2,IL6,JAK1,MET,PRKCI,RELA,STAT1
IGF-1 Signaling	2.28	0.0865	#NUM!	IGFBP7,JAK1,PRKAG2,PRKCI,SFN,YWHAB,YWHAH,YWHAQ,YWHAZ
Role of Hypercytokinemia/hyperchemokinemia in the Pathogenesis of Influenza	2.27	0.093	#NUM!	CXCL3,CXCL8,IL18,IL6,JAK1,MX1,MYD88,STAT1
HIPPO signaling	2.27	0.093	#NUM!	CD44,CSNK1D,PPP2R2A,SFN,YWHAB,YWHAH,YWHAQ,YWHAZ
Acute Phase Response Signaling	2.26	0.0703	#NUM!	C3,CFB,CP,FTL,HMOX1,IL18,IL1R1,IL6,MYD88,NFKBIA,RELA,SAA1,SOD2
Dopamine-DARPP32 Feedback in cAMP Signaling	2.26	0.0703	#NUM!	ATF2,ATP2A2,CALM1 (includes others),CREB3,CREB5,CREBBP,CSNK1D,GNAI1,GNAI2,KCNJ16,PPP2R2A,PRKAG2,PRKCI
Protein Ubiquitination Pathway	2.24	0.062	#NUM!	CRYAB,DNAJA1,DNAJB1,ELOC,HLA-A,HLA-B,HLA-C,HSP90AA1,HSPA8,PSMB2,PSMD11,UBB,UBC,UBE2H,UBE2L3,UBE2V1,UCHL1
Xenobiotic Metabolism AHR Signaling Pathway	2.24	0.092	#NUM!	AHR,CREBBP,GSTP1,HSP90AA1,IL6,NQO1,RELA,UGT1A9 (includes others)
Gαs Signaling	2.24	0.08	#NUM!	ADD1,ADORA2B,ATF2,CREB3,CREB5,CREBBP,GNAI1,GNAI2,GNB1,PRKAG2
PI3K Signaling in B Lymphocytes	2.24	0.0759	#NUM!	ATF2,C3,CALM1 (includes others),CD81,MALT1,NFKBIA,PIK3AP1,PRKCI,RAC1,RELA,VAV3
Pancreatic Adenocarcinoma Signaling	2.21	0.0794	#NUM!	BCL2L1,CCND1,CDC42,HMOX1,JAK1,RAC1,RELA,STAT1,TGFBR2,TP53
HMGB1 Signaling	2.21	0.0719	#NUM!	CDC42,CXCL8,ICAM1,IL18,IL1R1,IL6,RAC1,RELA,RHOB,RHOC,RHOF,TNFSF10
GP6 Signaling Pathway	2.19	0.0787	#NUM!	CALM1 (includes others),COL4A2,ITGB3,LAMA3,LAMB3,LAMC1,LAMC2,PRKCI,RAC1,VAV3
CXCR4 Signaling	2.19	0.0714	#NUM!	CDC42,GNAI1,GNAI2,GNB1,MYL12A,MYL12B,MYL6,PRKCI,RAC1,RHOB,RHOC,RHOF
GNRH Signaling	2.15	0.0681	#NUM!	ATF2,CALM1 (includes others),CDC42,CREB3,CREB5,CREBBP,GNAI1,GNAI2,GNB1,PRKAG2,PRKCI,RAC1,RELA
IL-10 Signaling	2.15	0.0972	#NUM!	HMOX1,IL18,IL1R1,IL6,JAK1,NFKBIA,RELA
Apelin Adipocyte Signaling Pathway	2.12	0.0879	#NUM!	GNAI1,GNAI2,GPX1,GSTP1,HIF1A,PPARGC1A,PRKAG2,RAC1
Oxytocin Signaling Pathway	2.12	0.0603	#NUM!	ATF2,ATP2B4,CALM1 (includes others),CREB3,CREB5,CREBBP,CXCL8,GJA1,GNAI1,GNAI2,GNB1,IL6,MYH9,MYL6,PRKAG2,PRKCI,RELA
NAD Signaling Pathway	2.11	0.0728	#NUM!	GJA1,H1-5,H2BC12,HIF1A,NT5E,PARP14,PPARGC1A,PRKAG2,SOD2,TP53,TPCN1
Autophagy	2.09	0.0648	#NUM!	ATF2,CALM1 (includes others),CREB3,CREB5,CREBBP,GABARAPL1,HIF1A,MAP1LC3B,MYD88,PPP2R2A,PRKAG2,RAB7A,RIPK2,TP53
IL-17A Signaling in Gastric Cells	2.09	0.154	#NUM!	CCL20,CXCL1,CXCL8,RELA

Gα12/13 Signaling	2.05	0.0752	#NUM!	CDC42,CDH6,CTNNB1,F2RL1,MYL12A,MYL12B,MYL6,NFKBIA,RELA,VAV3
Glutathione Redox Reactions II	2.04	0.4	#NUM!	GSR,TXNDC12
Polyamine Regulation in Colon Cancer	2.04	0.103	#NUM!	CTNNB1,EIF5A,OAZ1,SAT1,SMS,TP53
CDK5 Signaling	2	0.0783	#NUM!	CAPN1,ITGA3,ITGB1,LAMA3,LAMB3,LAMC1,LAMC2,PPP2R2A,PRKAG2
IL-1 Signaling	1.99	0.0833	#NUM!	GNAI1,GNAI2,GNB1,IL1R1,MYD88,NFKBIA,PRKAG2,RELA
Death Receptor Signaling	1.99	0.0833	#NUM!	ACTB,ACTG1,CFLAR,NFKBIA,PARP14,RELA,TNFRSF21,TNFSF10
Role of PKR in Interferon Induction and Antiviral Response	1.99	0.0735	#NUM!	ATF2,HSP90AA1,HSPA8,IL18,JAK1,MYD88,NFKBIA,RELA,STAT1,TP53
NF-κB Activation by Viruses	1.97	0.0897	#NUM!	ITGA3,ITGAV,ITGB1,ITGB3,NFKBIA,PRKCI,RELA
GADD45 Signaling	1.97	0.1	#NUM!	CCND1,CCND3,PCNA,RELA,TGFBR2,TP53
Toll-like Receptor Signaling	1.97	0.0897	#NUM!	IL18,MYD88,NFKBIA,RELA,TNFAIP3,UBB,UBC
IL-7 Signaling Pathway	1.97	0.0897	#NUM!	CCND1,CCND3,JAK1,MCL1,MET,SLC2A1,STAT1
Aryl Hydrocarbon Receptor Signaling	1.95	0.0692	#NUM!	AHR,CCNA2,CCND1,CCND3,GSTP1,HSP90AA1,IL6,NQO1,RELA,TGM2,TP53
Reelin Signaling in Neurons	1.95	0.0725	#NUM!	ACTR2,APP,ARHGEF38,ARPC2,CDC42,CFL1,ITGA3,ITGB1,MAP1B,RAC1
Thrombin Signaling	1.95	0.0622	#NUM!	ARHGEF38,CDC42,GNAI1,GNAI2,GNB1,MYL12A,MYL12B,MYL6,PRKCI,RAC1,RELA,RHOB,RHOC,RHOF
VEGF Signaling	1.91	0.0808	#NUM!	ACTB,ACTG1,ACTN1,ACTN4,BCL2L1,HIF1A,SFN,VCL
Apelin Cardiomyocyte Signaling Pathway	1.91	0.0808	#NUM!	ATP2A2,GNAI1,GNAI2,HIF1A,MYL12A,MYL12B,MYL6,PRKCI
Estrogen-Dependent Breast Cancer Signaling	1.88	0.0864	#NUM!	ATF2,CCND1,CREB3,CREB5,CREBBP,HSD17B12,RELA
Pentose Phosphate Pathway (Non-oxidative Branch)	1.87	0.333	#NUM!	TALDO1,TKT
Zymosterol Biosynthesis	1.87	0.333	#NUM!	MSMO1,TM7SF2
B Cell Development	1.86	0.109	#NUM!	HLA-A,HLA-B,HLA-DMA,HLA-DRA,HLA-DRB1
IL-17 Signaling	1.84	0.0642	#NUM!	ATF2,CCL20,CXCL1,CXCL3,CXCL8,HSP90AA1,IL18,IL6,JAK1,LCN2,RELA,TNFSF10
Opioid Signaling Pathway	1.84	0.0573	#NUM!	AP2B1,ATF2,CALM1 (includes others),CDC42,CREB3,CREB5,CREBBP,CTNNB1,GNAI1,GNAI2,GNB1,NFKBIA,PDE1C,PRKAG2,PRKCI,RAC1
Ephrin A Signaling	1.82	0.106	#NUM!	CDC42,CFL1,EPHA2,RAC1,VAV3
PXR/RXR Activation	1.81	0.0923	#NUM!	ALAS1,IL6,PPARGC1A,PRKAG2,RELA,UGT1A9 (includes others)
Activation of IRF by Cytosolic Pattern Recognition Receptors	1.81	0.0923	#NUM!	ATF2,CREBBP,IL6,NFKBIA,RELA,STAT1
PEDF Signaling	1.8	0.0833	#NUM!	BCL2L1,CFLAR,NFKBIA,RAC1,RELA,SOD2,TP53
Apelin Muscle Signaling Pathway	1.79	0.104	#NUM!	GNAI1,GNAI2,GNB1,PPARGC1A,PRKAG2
WNT/Ca+ pathway	1.78	0.0909	#NUM!	ATF2,CREB3,CREB5,CREBBP,FZD6,RELA
Androgen Signaling	1.77	0.0651	#NUM!	CALM1 (includes others),CCND1,CREBBP,DNAJB1,GNAI1,GNAI2,GNB1,HSP90AA1,PRKAG2,PRKCI,RELA
PD-1, PD-L1 cancer immunotherapy pathway	1.75	0.0755	#NUM!	BCL2L1,HLA-A,HLA-B,HLA-C,HLA-DMA,HLA-DRA,HLA-DRB1,JAK1
Phagosome Formation	1.74	0.0462	#NUM!	ACTR2,ADORA2B,ARPC2,C3,CDC42,CFL1,CLEC4E,F2RL1,FZD6,GPR108,GPR143,GPRC5A,HMOX1,ITGA3,ITGAV,ITGB1,ITGB3,ITGB4,ITGB8,MALT1,MYD88,MYH9,MYL12A,MYL12B,MYL6,NPFFR2,PIP5K1A,PLAAT4,PRKCI,RAC1,RXFP1,VAV3
Thioredoxin Pathway	1.74	0.286	#NUM!	TXN,TXNRD1
Glioblastoma Multiforme Signaling	1.73	0.0643	#NUM!	CCND1,CCND3,CDC42,CTNNB1,FZD6,RAC1,RHOB,RHOC,RHOF,TP53,WNT7A
Chronic Myeloid Leukemia Signaling	1.73	0.0748	#NUM!	BCL2L1,CCND1,CCND3,CTBP2,MECOM,RELA,TGFBR2,TP53
BMP signaling pathway	1.72	0.0805	#NUM!	ATF2,BMP1,CREBBP,PRKAG2,RELA,RUNX2,SOSTDC1
Regulation Of The Epithelial Mesenchymal Transition In Development Pathway	1.72	0.0805	#NUM!	CTNNB1,FZD6,HIF1A,PSENEN,PYGO1,RELA,WNT7A
Cellular Effects of Sildenafil (Viagra)	1.71	0.0667	#NUM!	ACTB,ACTG1,CALM1 (includes others),MYH9,MYL12A,MYL12B,MYL6,PDE1C,PRKAG2,SLC4A11
α-Adrenergic Signaling	1.71	0.0741	#NUM!	CALM1 (includes others),GNAI1,GNAI2,GNB1,GYS1,PRKAG2,PRKCI,PYGL
Oxytocin In Brain Signaling Pathway	1.68	0.0609	#NUM!	ATF2,CALM1 (includes others),CREB3,CREB5,CREBBP,GNAI1,GNAI2,GNB1,IL6,PLAAT4,PRKCI,PTPN1
Immunogenic Cell Death Signaling Pathway	1.65	0.0778	#NUM!	ANXA1,BCAP31,CFLAR,HSP90AA1,HSPA8,IL6,STING1
Inflammasome pathway	1.64	0.15	#NUM!	CTSB,IL18,MYD88
Role of MAPK Signaling in Promoting the Pathogenesis of Influenza	1.62	0.0714	#NUM!	ATF2,ATP6V0C,ATP6V0D1,ATP6V0E1,ATP6V1E1,NFKBIA,PLAAT4,TCIRG1
Superoxide Radicals Degradation	1.62	0.25	#NUM!	NQO1,SOD2
Insulin Secretion Signaling Pathway	1.62	0.0551	#NUM!	ATF2,CREB3,CREB5,CREBBP,EIF4A1,EIF4G2,JAK1,PRKAG2,PRKCI,SCNN1A,SLC2A1,SPCS3,SRP14,SRP72,STAT1
Adipogenesis pathway	1.59	0.0667	#NUM!	CTBP2,CTNNB1,FGFR2,FZD6,HIF1A,KLF5,TP53,TXNIP,XBP1

Senescence Pathway	1.59	0.0535	#NUM!	CALM1 (includes others),CAPN1,CAPNS1,CCND1,CCND3,CREBBP,CXCL8,DHCR24,IL6,PML,PPP2R2A,SAA1,SOD2,STING1,TGFBR2,TP53
IL-4 Signaling	1.58	0.0753	#NUM!	HLA-A,HLA-B,HLA-DMA,HLA-DRA,HLA-DRB1,INPPL1,JAK1
HER-2 Signaling in Breast Cancer	1.58	0.0573	#NUM!	ARF1,ARF4,CCND1,CDC42,ITGB1,ITGB3,ITGB4,ITGB8,NFKBIA,PRKCI,RELA,RPS6,TP53
Ovarian Cancer Signaling	1.58	0.0633	#NUM!	CCND1,CD44,CTNNB1,FGF9,FZD6,GJA1,MMP7,PRKAG2,TP53,WNT7A
SNARE Signaling Pathway	1.57	0.0662	#NUM!	CALM1 (includes others),HSPA8,MYH9,MYL6,PRKAG2,RAB11A,RAB6A,RAB7A,RAB8A
IL-12 Signaling and Production in Macrophages	1.57	0.0662	#NUM!	APOL1,CLU,IL18,MYD88,NFKBIA,PRKCI,RAB7A,RELA,STAT1
Complement System	1.56	0.108	#NUM!	C3,CD55,CD59,CFB
Th2 Pathway	1.56	0.0657	#NUM!	HLA-A,HLA-B,HLA-DMA,HLA-DRA,HLA-DRB1,ICAM1,JAK1,PSENEN,TGFBR2
Lymphotoxin β Receptor Signaling	1.55	0.0909	#NUM!	BCL2L1,CREBBP,CXCL1,NFKBIA,RELA
Amyotrophic Lateral Sclerosis Signaling	1.54	0.069	#NUM!	BCL2L1,CAPN1,CAPNS1,GPX1,RAB5A,RAB5C,RAC1,TP53
Bladder Cancer Signaling	1.54	0.069	#NUM!	CCND1,CXCL8,FGF18,FGF9,MMP14,MMP7,THBS1,TP53
Sperm Motility	1.53	0.0549	#NUM!	AXL,CALM1 (includes others),EPHA2,ERBB4,FGFR2,GNAI1,GNAI2,GNB1,JAK1,MET,PDE1C,PLAAT4,PRKAG2,PRKCI
IL-17A Signaling in Fibroblasts	1.52	0.105	#NUM!	IL6,LCN2,NFKBIA,RELA
CSDE1 Signaling Pathway	1.52	0.0893	#NUM!	CCND3,CTNNB1,PTBP1,RAC1,VIM
Inhibition of ARE-Mediated mRNA Degradation Pathway	1.51	0.0617	#NUM!	PPP2R2A,PRKAG2,PSMB2,PSMD11,SFN,TNFSF10,YWHAB,YWHAH,YWHAQ,YWHAZ
Acetyl-CoA Biosynthesis III (from Citrate)	1.51	1	#NUM!	ACLY
Thiamin Salvage III	1.51	1	#NUM!	TPK1
Apelin Endothelial Signaling Pathway	1.49	0.0638	#NUM!	CALM1 (includes others),GNAI1,GNAI2,GNB1,HIF1A,ICAM1,PRKAG2,PRKCI,RELA
Cholecystokinin/Gastrin-mediated Signaling	1.48	0.0672	#NUM!	ATF2,CDC42,IL18,PRKCI,RAC1,RHOB,RHOC,RHOF
Role of MAPK Signaling in Inhibiting the Pathogenesis of Influenza	1.48	0.0779	#NUM!	ATF2,CXCL8,IL6,MYD88,NFKBIA,PLAAT4
Breast Cancer Regulation by Stathmin1	1.48	0.0455	#NUM!	ADORA2B,ATF2,CCND1,CCND3,CREB3,CREB5,CREBBP,F2RL1,FZD6,GNB1,GPR108,GPR143,GPRC5A,HIF1A,NPFFR2,PPP2R2A,PRKAG2,PRKCI,RAC1,RXFP1,TP53,TUBA1A,TUBA1B,TUBA1C,TUBA4A,TUBB,TUBB4B
NGF Signaling	1.47	0.0667	#NUM!	ATF2,CDC42,CREB3,CREB5,CREBBP,RAC1,RELA,TP53
Neuroprotective Role of THOP1 in Alzheimer's Disease	1.45	0.0661	#NUM!	APP,ECE1,HLA-A,HLA-B,HLA-C,KLK10,PRKAG2,TMPRSS4
ATM Signaling	1.43	0.07	#NUM!	ATF2,CREB3,CREB5,CREBBP,NFKBIA,PPP2R2A,TP53
Aldosterone Signaling in Epithelial Cells	1.42	0.0595	#NUM!	CRYAB,DNAJA1,DNAJB1,DUSP1,HSP90AA1,HSPA8,ICMT,PIP5K1A,PRKCI,SCNN1A
PCP (Planar Cell Polarity) Pathway	1.41	0.0833	#NUM!	ATF2,FZD6,PFN1,RAC1,WNT7A
Xenobiotic Metabolism Signaling	1.4	0.0515	#NUM!	AHR,CHST15,CREBBP,FTL,GSTP1,HMOX1,HSP90AA1,IL6,NQO1,PPARGC1A,PPP2R2A,PRKCI,RELA,SULT1A3/SULT1A4,UGT1A9 (includes others)
Role of JAK family kinases in IL-6-type Cytokine Signaling	1.38	0.12	#NUM!	IL6,JAK1,STAT1
CDP-diacylglycerol Biosynthesis I	1.38	0.12	#NUM!	GPAT4,LPCAT1,MBOAT7
Mitochondrial Dysfunction	1.38	0.0585	#NUM!	ACO1,APP,CYB5R3,GSR,NDUFS5,PSENEN,SOD2,UCP2,VDAC1,VDAC3
JAK/STAT Signaling	1.37	0.0732	#NUM!	BCL2L1,IL6,JAK1,PTPN1,RELA,STAT1
FXR/RXR Activation	1.36	0.0635	#NUM!	APOL1,C3,CLU,CREBBP,G6PC3,IL18,PPARGC1A,SAA1
Apoptosis Signaling	1.36	0.0673	#NUM!	BCL2L1,CAPN1,CAPNS1,MCL1,NFKBIA,RELA,TP53
Dilated Cardiomyopathy Signaling Pathway	1.34	0.06	#NUM!	ACTB,ACTG1,ATP2A2,DAG1,DSG2,MYH9,MYL6,PRKAG2,TPM1
Role of JAK1, JAK2 and TYK2 in Interferon Signaling	1.34	0.115	#NUM!	JAK1,RELA,STAT1
Corticotropin Releasing Hormone Signaling	1.33	0.0596	#NUM!	ATF2,CALM1 (includes others),CREB3,CREB5,CREBBP,GNAI1,GNAI2,PRKAG2,PRKCI
MIF Regulation of Innate Immunity	1.32	0.0909	#NUM!	CD74,NFKBIA,RELA,TP53
LPS-stimulated MAPK Signaling	1.31	0.0706	#NUM!	ATF2,CDC42,NFKBIA,PRKCI,RAC1,RELA
PPAR Signaling	1.3	0.0654	#NUM!	CREBBP,HSP90AA1,IL18,IL1R1,NFKBIA,PPARGC1A,RELA
Phosphatidylglycerol Biosynthesis II (Non-plastidic)	1.3	0.111	#NUM!	GPAT4,LPCAT1,MBOAT7
RAR Activation	1.26	0.0539	#NUM!	ACTB,AKR1B1,AKR1C3,CREBBP,DUSP1,PML,PPARGC1A,PRKAG2,PRKCI,RAC1,RELA
Glutathione Redox Reactions I	1.26	0.107	#NUM!	GPX1,GSR,GSTP1
Role of Pattern Recognition Receptors in Recognition of Bacteria and Viruses	1.25	0.0577	#NUM!	C3,CXCL8,IL18,IL6,MYD88,PRKCI,RELA,RIPK2,TNFSF10
Mitotic Roles of Polo-Like Kinase	1.24	0.0746	#NUM!	CAPN1,HSP90AA1,PLK1,PLK2,PPP2R2A
nNOS Signaling in Neurons	1.24	0.0851	#NUM!	CALM1 (includes others),CAPN1,CAPNS1,PRKCI
Oleate Biosynthesis II (Animals)	1.22	0.154	#NUM!	FADS1,FADS2

Guanosine Nucleotides Degradation III	1.22	0.154	#NUM!	GDA,NT5E
Antioxidant Action of Vitamin C	1.21	0.0625	#NUM!	HMOX1,NFKBIA,PLAAT4,RELA,SLC2A1,TXN,TXNRD1
D-myo-inositol (1,4,5,6)-Tetrakisphosphate Biosynthesis	1.21	0.0546	#NUM!	DUSP1,DUSP4,DUSP5,G6PC3,INPPL1,MTMR2,NUDT4,PPP2R2A,PTPN1,SSH3
D-myo-inositol (3,4,5,6)-tetrakisphosphate Biosynthesis	1.21	0.0546	#NUM!	DUSP1,DUSP4,DUSP5,G6PC3,INPPL1,MTMR2,NUDT4,PPP2R2A,PTPN1,SSH3
Asparagine Degradation I	1.21	0.5	#NUM!	ASRGL1
Epoxysqualene Biosynthesis	1.21	0.5	#NUM!	FDFT1
Spermine Biosynthesis	1.21	0.5	#NUM!	SMS
GDP-L-fucose Biosynthesis I (from GDP-D-mannose)	1.21	0.5	#NUM!	GFUS
Glutamine Degradation I	1.21	0.5	#NUM!	GLS
Unfolded protein response	1.21	0.0667	#NUM!	DNAJA1,DNAJB1,HSPA8,P4HB,VCP,XBP1
3-phosphoinositide Biosynthesis	1.2	0.0526	#NUM!	DUSP1,DUSP4,DUSP5,G6PC3,INPPL1,MTMR2,NUDT4,PIP5K1A,PPP2R2A,PTPN1,SSH3
cAMP-mediated signaling	1.19	0.0511	#NUM!	ADORA2B,AKAP12,ATF2,CALM1 (includes others),CREB3,CREB5,CREBBP,DUSP1,DUSP4,GNAI1,GNAI2,PDE1C
White Adipose Tissue Browning Pathway	1.17	0.058	#NUM!	ATF2,CREB3,CREB5,CREBBP,CTBP2,FGFR2,PPARGC1A,PRKAG2
Mevalonate Pathway I	1.16	0.143	#NUM!	ACAT2,HMGCR
IL-13 Signaling Pathway	1.15	0.0603	#NUM!	AGR2,BCL2L1,CTNNB1,DUSP1,JAK1,STAT1,TP53
Insulin Receptor Signaling	1.14	0.0571	#NUM!	ACLY,GYS1,INPPL1,JAK1,PRKAG2,PRKCI,PTPN1,SCNN1A
Melatonin Signaling	1.13	0.0694	#NUM!	CALM1 (includes others),GNAI1,GNAI2,PRKAG2,PRKCI
Basal Cell Carcinoma Signaling	1.13	0.0694	#NUM!	BMP1,CTNNB1,FZD6,TP53,WNT7A
TNFR1 Signaling	1.13	0.0784	#NUM!	CDC42,NFKBIA,RELA,TNFAIP3
Human Embryonic Stem Cell Pluripotency	1.12	0.0542	#NUM!	BMP1,CTNNB1,FGFR2,FZD6,GNAI1,GNAI2,GNB1,TGFBR2,WNT7A
G-Protein Coupled Receptor Signaling	1.12	0.0413	#NUM!	ADORA2B,ATF2,CALM1 (includes others),CCND1,CDC42,CREB3,CREB5,CREBBP,CTNNB1,DUSP1,DUSP4,F2RL1,FZD6,GNAI1,GNAI2,GNB1,GPR108,GPR143,GPRC5A,MYL12A,MYL12B,MYL6,NFKBIA,NPFFR2,PDE1C,PRKAG2,RAC1,RELA,RXFP1
Hepatic Cholestasis	1.12	0.0524	#NUM!	CXCL8,IL18,IL1R1,IL6,MYD88,NFKBIA,PRKAG2,PRKCI,RELA,TNFSF10
Hereditary Breast Cancer Signaling	1.12	0.0563	#NUM!	ACTB,CCND1,CCND3,CREBBP,SFN,TP53,UBB,UBC
TNFR2 Signaling	1.12	0.0938	#NUM!	NFKBIA,RELA,TNFAIP3
Xenobiotic Metabolism General Signaling Pathway	1.1	0.0559	#NUM!	AHR,CREBBP,FTL,GSTP1,HMOX1,NQO1,PRKCI,UGT1A9 (includes others)
Small Cell Lung Cancer Signaling	1.1	0.0625	#NUM!	BCL2L1,CCND1,CCND3,NFKBIA,RELA,TP53
Sphingosine-1-phosphate Signaling	1.09	0.0583	#NUM!	CDC42,GNAI1,GNAI2,RAC1,RHOB,RHOC,RHOF
3-phosphoinositide Degradation	1.07	0.0513	#NUM!	DUSP1,DUSP4,DUSP5,G6PC3,INPPL1,MTMR2,NUDT4,PPP2R2A,PTPN1,SSH3
Extrinsic Prothrombin Activation Pathway	1.06	0.125	#NUM!	F3,TFPI
D-myo-inositol (1,4,5)-trisphosphate Degradation	1.06	0.125	#NUM!	BPNT2,INPPL1
Role of Cytokines in Mediating Communication between Immune Cells	1.06	0.0741	#NUM!	CXCL8,IL18,IL32,IL6
4-1BB Signaling in T Lymphocytes	1.05	0.0882	#NUM!	ATF2,NFKBIA,RELA
Role of JAK2 in Hormone-like Cytokine Signaling	1.05	0.0882	#NUM!	JAK1,PTPN1,STAT1
NADH Repair	1.04	0.333	#NUM!	GAPDH
Serine Biosynthesis	1.04	0.333	#NUM!	PSAT1
1,25-dihydroxyvitamin D3 Biosynthesis	1.04	0.333	#NUM!	POR
Hypusine Biosynthesis	1.04	0.333	#NUM!	EIF5A
D-myo-inositol-5-phosphate Metabolism	1.03	0.0503	#NUM!	DUSP1,DUSP4,DUSP5,G6PC3,INPPL1,MTMR2,NUDT4,PPP2R2A,PTPN1,SSH3
Coagulation System	1.02	0.0857	#NUM!	F3,PLAU,TFPI
IL-9 Signaling	1.02	0.0857	#NUM!	JAK1,RELA,STAT1
VDR/RXR Activation	1.02	0.0641	#NUM!	CALB1,PRKCI,RUNX2,SERPINB1,SPP1
Neurotrophin/TRK Signaling	1.02	0.0641	#NUM!	ATF2,CDC42,CREB3,CREB5,CREBBP
RAN Signaling	1.01	0.118	#NUM!	KPNA2,RAN
Maturity Onset Diabetes of Young (MODY) Signaling	1	0.0633	#NUM!	APOL1,APOL2,CREBBP,GAPDH,UCP2
MIF-mediated Glucocorticoid Regulation	0.996	0.0833	#NUM!	CD74,NFKBIA,RELA
Role of BRCA1 in DNA Damage Response	0.983	0.0625	#NUM!	ACTB,BABAM2,PLK1,STAT1,TP53

Mouse Embryonic Stem Cell Pluripotency	0.975	0.0577	#NUM!	CREBBP,CTNNB1,FZD6,ID2,JAK1,TP53
IL-6 Signaling	0.975	0.0547	#NUM!	CXCL8,IL18,IL1R1,IL6,MCL1,NFKBIA,RELA
Role of CHK Proteins in Cell Cycle Checkpoint Control	0.971	0.069	#NUM!	PCNA,PLK1,PPP2R2A,TP53
TWEAK Signaling	0.971	0.0811	#NUM!	NFKBIA,RELA,TNFRSF12A
MSP-RON Signaling Pathway	0.971	0.069	#NUM!	ACTB,ACTG1,CSF1,KLK10
Differential Regulation of Cytokine Production in Macrophages and T Helper Cells by IL-17A and IL-17F	0.971	0.111	#NUM!	CXCL1,IL6
Superpathway of Geranylgeranyldiphosphate Biosynthesis I (via Mevalonate)	0.971	0.111	#NUM!	ACAT2,HMGCR
Relaxin Signaling	0.951	0.0516	#NUM!	GNAI1,GNAI2,GNB1,NFKBIA,PDE1C,PRKAG2,RELA,RXFP1
FLT3 Signaling in Hematopoietic Progenitor Cells	0.947	0.061	#NUM!	ATF2,CREB3,CREB5,CREBBP,STAT1
Synaptic Long Term Potentiation	0.939	0.0534	#NUM!	ATF2,CALM1 (includes others),CREB3,CREB5,CREBBP,PRKAG2,PRKCI
Purine Nucleotides Degradation II (Aerobic)	0.932	0.105	#NUM!	GDA,NT5E
DNA damage-induced 14-3-3σ Signaling	0.932	0.105	#NUM!	SFN,TP53
SPINK1 Pancreatic Cancer Pathway	0.932	0.0667	#NUM!	CTSB,F2RL1,KLK10,TGFBR2
Heme Degradation	0.924	0.25	#NUM!	HMOX1
Spermine and Spermidine Degradation I	0.924	0.25	#NUM!	SAT1
CCR3 Signaling in Eosinophils	0.9	0.0522	#NUM!	CALM1 (includes others),CFL1,GNAI1,GNAI2,GNB1,PRKCI,RAC1
Cyclins and Cell Cycle Regulation	0.9	0.0588	#NUM!	CCNA2,CCND1,CCND3,PPP2R2A,TP53
Superpathway of Inositol Phosphate Compounds	0.9	0.0462	#NUM!	DUSP1,DUSP4,DUSP5,G6PC3,INPPL1,MTMR2,NUDT4,PIP5K1A,PPP2R2A,PTPN1,SSH3
Mechanisms of Viral Exit from Host Cells	0.87	0.0732	#NUM!	ACTB,ACTG1,PRKCI
CDC42 Signaling	0.863	0.0399	#NUM!	ACTR2,ARPC2,ATF2,CDC42,CDC42SE1,CFL1,HLA-A,HLA-B,HLA-C,HLA-DMA,HLA-DRA,HLA-DRB1,IQGAP1,ITGA3,ITGAV,ITGB1,ITGB3,ITGB4,ITGB8,MYL12A,MYL12B,MYL6,PRKCI
Androgen Biosynthesis	0.86	0.0952	#NUM!	AKR1C3,SRD5A3
TEC Kinase Signaling	0.857	0.0398	#NUM!	ACTB,ACTG1,CDC42,GNAI1,GNAI2,GNB1,ITGA3,ITGAV,ITGB1,ITGB3,ITGB4,ITGB8,JAK1,PRKCI,RAC1,RELA,RHOB,RHOC,RHOF,STAT1,TNFRSF21,TNFSF10,VAV3
Induction of Apoptosis by HIV1	0.842	0.0615	#NUM!	BCL2L1,NFKBIA,RELA,TP53
Xenobiotic Metabolism CAR Signaling Pathway	0.842	0.0471	#NUM!	CHST15,CREBBP,GSTP1,HSP90AA1,PPARGC1A,PPP2R2A,PRKCI,SULT1A3/SULT1A4,UGT1A9 (includes others)
Creatine-phosphate Biosynthesis	0.836	0.2	#NUM!	CKMT1A/CKMT1B
Pentose Phosphate Pathway (Oxidative Branch)	0.836	0.2	#NUM!	PGD
Superpathway of Serine and Glycine Biosynthesis I	0.836	0.2	#NUM!	PSAT1
Tetrapyrrole Biosynthesis II	0.836	0.2	#NUM!	ALAS1
Myo-inositol Biosynthesis	0.836	0.2	#NUM!	BPNT2
dTMP De Novo Biosynthesis	0.836	0.2	#NUM!	TYMS
Tyrosine Degradation I	0.836	0.2	#NUM!	HGD
Acetate Conversion to Acetyl-CoA	0.836	0.2	#NUM!	ACSL1
Xenobiotic Metabolism PXR Signaling Pathway	0.833	0.0469	#NUM!	CHST15,CREBBP,GSTP1,HSP90AA1,PPARGC1A,PRKAG2,PRKCI,SULT1A3/SULT1A4,UGT1A9 (includes others)
Oncostatin M Signaling	0.827	0.0698	#NUM!	JAK1,PLAU,STAT1
Superpathway of D-myo-inositol (1,4,5)-trisphosphate Metabolism	0.827	0.0909	#NUM!	BPNT2,INPPL1
Role of PI3K/AKT Signaling in the Pathogenesis of Influenza	0.824	0.0606	#NUM!	GNAI1,GNAI2,NFKBIA,RELA
Glutamate Receptor Signaling	0.824	0.0606	#NUM!	CALM1 (includes others),GLS,GNB1,SLC1A1
CD40 Signaling	0.807	0.0597	#NUM!	ICAM1,NFKBIA,RELA,TNFAIP3
PPARα/RXRα Activation	0.804	0.0462	#NUM!	CREBBP,HSP90AA1,IL1R1,IL6,NFKBIA,PPARGC1A,PRKAG2,RELA,TGFBR2
G Protein Signaling Mediated by Tubby	0.804	0.0682	#NUM!	GNAI1,GNAI2,GNB1
BER (Base Excision Repair) Pathway	0.804	0.0682	#NUM!	ARL6IP5,PCNA,TP53
Differential Regulation of Cytokine Production in Intestinal Epithelial Cells by IL-17A and IL-17F	0.796	0.087	#NUM!	CXCL1,LCN2
Pyrimidine Deoxyribonucleotides De Novo Biosynthesis I	0.796	0.087	#NUM!	CMPK1,TYMS
Apelin Cardiac Fibroblast Signaling Pathway	0.796	0.087	#NUM!	IL6,PRKAG2
Cell Cycle: G1/S Checkpoint Regulation	0.79	0.0588	#NUM!	CCND1,CCND3,RPL5,TP53
Pyroptosis Signaling Pathway	0.783	0.0538	#NUM!	GBP2,IL18,IL1R1,PRKAG2,TXNIP

Estrogen Biosynthesis	0.783	0.0667	#NUM!	AKR1C3,HSD17B12,POR
IL-22 Signaling	0.767	0.0833	#NUM!	JAK1,STAT1
Role of RIG1-like Receptors in Antiviral Innate Immunity	0.764	0.0652	#NUM!	CREBBP,NFKBIA,RELA
Role of NANOG in Mammalian Embryonic Stem Cell Pluripotency	0.764	0.05	#NUM!	BMP1,CTNNB1,FZD6,JAK1,TP53,WNT7A
Nitric Oxide Signaling in the Cardiovascular System	0.764	0.05	#NUM!	ATP2A2,CALM1 (includes others),HSP90AA1,PDE1C,PRKAG2,PRKCI
IL-23 Signaling Pathway	0.764	0.0652	#NUM!	HIF1A,NFKBIA,RELA
Glycine Cleavage Complex	0.762	0.167	#NUM!	GLDC
Rapoport-Luebering Glycolytic Shunt	0.762	0.167	#NUM!	PGAM1
Renin-Angiotensin Signaling	0.752	0.0496	#NUM!	ATF2,PRKAG2,PRKCI,RAC1,RELA,STAT1
TGF-β Signaling	0.745	0.0521	#NUM!	CDC42,CREBBP,RUNX2,TGFBR2,TGIF1
Melanocyte Development and Pigmentation Signaling	0.719	0.051	#NUM!	ATF2,CREB3,CREB5,CREBBP,PRKAG2
Estrogen-mediated S-phase Entry	0.712	0.0769	#NUM!	CCNA2,CCND1
Ceramide Biosynthesis	0.701	0.143	#NUM!	SPTLC3
Glycogen Biosynthesis II (from UDP-D-Glucose)	0.701	0.143	#NUM!	GYS1
Cardiac β-adrenergic Signaling	0.697	0.0444	#NUM!	AKAP12,ATP2A2,GNAI1,GNAI2,GNB1,PDE1C,PPP2R2A,PRKAG2
G Beta Gamma Signaling	0.668	0.0465	#NUM!	CDC42,GNAI1,GNAI2,GNB1,PRKAG2,PRKCI
Circadian Rhythm Signaling	0.666	0.041	#NUM!	ATF2,CREB3,CREB5,CREBBP,CSNK1D,GNAI1,GNAI2,GNB1,HIF1A,PRKAG2,PRKCI
Role of WNT/GSK-3β Signaling in the Pathogenesis of Influenza	0.648	0.0513	#NUM!	CSNK1D,CTNNB1,FZD6,WNT7A
MicroRNA Biogenesis Signaling Pathway	0.64	0.0428	#NUM!	DDX17,DNAJB1,HMOX1,HNRNPA1,HSP90AA1,HSPA8,RAN,TP53
GABA Receptor Signaling	0.638	0.0455	#NUM!	AP2B1,GNAI1,GNAI2,GNB1,UBB,UBC
IL-3 Signaling	0.635	0.0506	#NUM!	JAK1,PRKCI,RAC1,STAT1
Thyroid Cancer Signaling	0.635	0.0506	#NUM!	CCND1,CTNNB1,CXCL8,TP53
Chemokine Signaling	0.622	0.05	#NUM!	CALM1 (includes others),CFL1,GNAI1,GNAI2
Role of p14/p19ARF in Tumor Suppression	0.62	0.0667	#NUM!	RAC1,TP53
Citrulline Biosynthesis	0.606	0.111	#NUM!	GLS
Heme Biosynthesis II	0.606	0.111	#NUM!	ALAS1
FAT10 Signaling Pathway	0.597	0.0536	#NUM!	MAP1LC3B,PSMB2,PSMD11
Role of NFAT in Cardiac Hypertrophy	0.59	0.0405	#NUM!	CALM1 (includes others),GNAI1,GNAI2,GNB1,IL6,PRKAG2,PRKCI,RCAN2,TGFBR2
CD27 Signaling in Lymphocytes	0.582	0.0526	#NUM!	BCL2L1,NFKBIA,RELA
Methylglyoxal Degradation III	0.58	0.0625	#NUM!	AKR1B1,AKR1C3
LPS/IL-1 Mediated Inhibition of RXR Function	0.569	0.0394	#NUM!	ABCC4,ACSL1,ALAS1,CHST15,GSTP1,IL18,IL1R1,MYD88,PPARGC1A,SULT1A3/SULT1A4
Ketolysis	0.567	0.1	#NUM!	ACAT2
Airway Inflammation in Asthma	0.561	0.0606	#NUM!	CCL20,CXCL8
Endometrial Cancer Signaling	0.542	0.05	#NUM!	CCND1,CTNNB1,TP53
Retinoic acid Mediated Apoptosis Signaling	0.542	0.05	#NUM!	CFLAR,PARP14,TNFSF10
Ketogenesis	0.532	0.0909	#NUM!	ACAT2
Dolichyl-diphosphooligosaccharide Biosynthesis	0.532	0.0909	#NUM!	ALG3
Ethanol Degradation II	0.526	0.0571	#NUM!	ACSL1,ADH6
Phospholipase C Signaling	0.524	0.0352	#NUM!	ATF2,CALM1 (includes others),CDC42,CREB3,CREB5,CREBBP,GNB1,HMOX1,ITGA3,ITGAV,ITGB1,ITGB3,ITGB4,ITGB8,MYL12A,MYL12B,MYL6,PRKCI,RAC1,RELA,RHOB,RHOC,RHOF,TGM2
Melatonin Degradation I	0.516	0.0484	#NUM!	POR,SULT1A3/SULT1A4,UGT1A9 (includes others)
Acute Myeloid Leukemia Signaling	0.502	0.044	#NUM!	CCND1,JUP,PML,RELA
Hematopoiesis from Multipotent Stem Cells	0.5	0.0833	#NUM!	CSF1
Docosahexaenoic Acid (DHA) Signaling	0.478	0.0526	#NUM!	APP,BCL2L1
Cell Cycle Regulation by BTG Family Proteins	0.478	0.0526	#NUM!	CCND1,PPP2R2A
Glycogen Degradation II	0.472	0.0769	#NUM!	PYGL
Type II Diabetes Mellitus Signaling	0.466	0.0392	#NUM!	ACSL1,NFKBIA,PKM,PRKAG2,PRKCI,RELA
Glioma Signaling	0.463	0.0403	#NUM!	CALM1 (includes others),CCND1,CCND3,PRKCI,TP53

Superpathway of Melatonin Degradation	0.46	0.0448	#NUM!	POR,SULT1A3/SULT1A4,UGT1A9 (includes others)
Thyroid Hormone Metabolism II (via Conjugation and/or Degradation)	0.45	0.05	#NUM!	SULT1A3/SULT1A4,UGT1A9 (includes others)
ERBB4 Signaling	0.449	0.0441	#NUM!	ERBB4,PRKCI,PSENEN
Acyl-CoA Hydrolysis	0.446	0.0714	#NUM!	ACOT7
Urate Biosynthesis/Inosine 5'-phosphate Degradation	0.446	0.0714	#NUM!	NT5E
Colanic Acid Building Blocks Biosynthesis	0.446	0.0714	#NUM!	GFUS
eNOS Signaling	0.445	0.0385	#NUM!	CALM1 (includes others),CCNA2,HSP90AA1,HSPA8,PRKAG2,PRKCI
UVA-Induced MAPK Signaling	0.438	0.0408	#NUM!	BCL2L1,PARP14,STAT1,TP53
GM-CSF Signaling	0.428	0.0429	#NUM!	BCL2L1,CCND1,STAT1
April Mediated Signaling	0.423	0.0476	#NUM!	NFKBIA,RELA
Telomere Extension by Telomerase	0.423	0.0667	#NUM!	HNRNPA1
Fatty Acid Activation	0.423	0.0667	#NUM!	ACSL1
Retinoate Biosynthesis I	0.423	0.0476	#NUM!	AKR1B1,AKR1C3
Superpathway of Citrulline Metabolism	0.423	0.0667	#NUM!	GLS
Choline Biosynthesis III	0.423	0.0667	#NUM!	HMOX1
Glycogen Degradation III	0.423	0.0667	#NUM!	PYGL
Serotonin Degradation	0.418	0.0423	#NUM!	ADH6,SULT1A3/SULT1A4,UGT1A9 (includes others)
B Cell Activating Factor Signaling	0.41	0.0465	#NUM!	NFKBIA,RELA
Adenosine Nucleotides Degradation II	0.401	0.0625	#NUM!	NT5E
Parkinson's Signaling	0.401	0.0625	#NUM!	UCHL1
GPCR-Mediated Integration of Enteroendocrine Signaling Exemplified by an L Cell	0.382	0.04	#NUM!	GNAI1,GNAI2,PRKAG2
1D-myo-inositol Hexakisphosphate Biosynthesis II (Mammalian)	0.381	0.0588	#NUM!	INPPL1
D-myo-inositol (1,3,4)-trisphosphate Biosynthesis	0.381	0.0588	#NUM!	INPPL1
Role of OCT4 in Mammalian Embryonic Stem Cell Pluripotency	0.375	0.0435	#NUM!	SPP1,TP53
Apelin Pancreas Signaling Pathway	0.375	0.0435	#NUM!	PRKAG2,RELA
CREB Signaling in Neurons	0.369	0.033	#NUM!	ADORA2B,ATF2,CALM1 (includes others),CREB3,CREB5,CREBBP,F2RL1,FGFR2,FZD6,GNAI1,GNAI2,GNB1,GPR108,GPR143,GPRC5A,NPFFR2,PRKAG2,PRKCI,RXFP1,TGFBR2
Dermatan Sulfate Biosynthesis (Late Stages)	0.364	0.0426	#NUM!	CHST15,SULT1A3/SULT1A4
Mismatch Repair in Eukaryotes	0.363	0.0556	#NUM!	PCNA
Isoleucine Degradation I	0.363	0.0556	#NUM!	ACAT2
Bile Acid Biosynthesis, Neutral Pathway	0.363	0.0556	#NUM!	AKR1C3
nNOS Signaling in Skeletal Muscle Cells	0.354	0.0417	#NUM!	CALM1 (includes others),DAG1
Systemic Lupus Erythematosus In T Cell Signaling Pathway	0.35	0.0326	#NUM!	ATF2,CD44,CDC42,CREB3,CREB5,CREBBP,EZR,GNAI1,GNAI2,HLA-A,HLA-B,HLA-C,HLA-DMA,HLA-DRA,HLA-DRB1,IL6,PPP2R2A,RAC1,RHOB,RHOC,RHOF
Glutaryl-CoA Degradation	0.345	0.0526	#NUM!	ACAT2
Mitochondrial L-carnitine Shuttle Pathway	0.345	0.0526	#NUM!	ACSL1
Spliceosomal Cycle	0.343	0.0408	#NUM!	HSPA8,SF3B4
Melanoma Signaling	0.333	0.04	#NUM!	CCND1,TP53
Chondroitin Sulfate Biosynthesis (Late Stages)	0.333	0.04	#NUM!	CHST15,SULT1A3/SULT1A4
UVC-Induced MAPK Signaling	0.323	0.0392	#NUM!	PRKCI,TP53
UVB-Induced MAPK Signaling	0.314	0.0385	#NUM!	PRKCI,TP53
Endoplasmic Reticulum Stress Pathway	0.313	0.0476	#NUM!	XBP1
Dendritic Cell Maturation	0.308	0.0319	#NUM!	ATF2,CREB3,CREB5,CREBBP,FSCN1,HLA-A,HLA-B,HLA-C,HLA-DMA,HLA-DRA,HLA-DRB1,ICAM1,IL18,IL32,IL6,MYD88,NFKBIA,RELA,STAT1
Endocannabinoid Neuronal Synapse Pathway	0.304	0.0336	#NUM!	GNAI1,GNAI2,GNB1,MGLL,PRKAG2
Putrescine Degradation III	0.299	0.0455	#NUM!	SAT1
Fc Epsilon RI Signaling	0.298	0.0339	#NUM!	INPPL1,PRKCI,RAC1,VAV3
PDGF Signaling	0.298	0.0349	#NUM!	INPPL1,JAK1,STAT1
GPCR-Mediated Nutrient Sensing in Enteroendocrine Cells	0.298	0.0339	#NUM!	GNAI1,GNAI2,PRKAG2,PRKCI
Phototransduction Pathway	0.297	0.037	#NUM!	GNB1,PRKAG2

EGF Signaling	0.288	0.0364	#NUM!	JAK1,STAT1
Cardiomyocyte Differentiation via BMP Receptors	0.287	0.0435	#NUM!	ATF2
The Visual Cycle	0.287	0.0435	#NUM!	AKR1C3
TCA Cycle II (Eukaryotic)	0.287	0.0435	#NUM!	ACO1
Tumoricidal Function of Hepatic Natural Killer Cells	0.274	0.0417	#NUM!	ICAM1
CNTF Signaling	0.272	0.0351	#NUM!	JAK1,STAT1
Prolactin Signaling	0.266	0.033	#NUM!	CREBBP,PRKCI,STAT1
RANK Signaling in Osteoclasts	0.266	0.033	#NUM!	CALM1 (includes others),NFKBIA,RELA
Nicotine Degradation III	0.265	0.0345	#NUM!	POR,UGT1A9 (includes others)
Chondroitin Sulfate Biosynthesis	0.265	0.0345	#NUM!	CHST15,SULT1A3/SULT1A4
Bupropion Degradation	0.262	0.04	#NUM!	POR
Ethanol Degradation IV	0.262	0.04	#NUM!	ACSL1
Triacylglycerol Degradation	0.257	0.0339	#NUM!	ABHD2,MGLL
Endothelin-1 Signaling	0.256	0.0312	#NUM!	ECE1,GNAI1,GNAI2,HMOX1,PLAAT4,PRKCI
ERBB Signaling	0.254	0.0323	#NUM!	CDC42,ERBB4,PRKCI
D-myo-inositol (1,4,5)-Trisphosphate Biosynthesis	0.251	0.0385	#NUM!	PIP5K1A
Tryptophan Degradation III (Eukaryotic)	0.251	0.0385	#NUM!	ACAT2
Dermatan Sulfate Biosynthesis	0.25	0.0333	#NUM!	CHST15,SULT1A3/SULT1A4
Non-Small Cell Lung Cancer Signaling	0.249	0.0319	#NUM!	CCND1,CCND3,TP53
NAD Salvage Pathway II	0.24	0.037	#NUM!	NT5E
Pregnenolone Biosynthesis	0.24	0.037	#NUM!	MICAL2
Thrombopoietin Signaling	0.231	0.0317	#NUM!	PRKCI,STAT1
Histidine Degradation VI	0.231	0.0357	#NUM!	MICAL2
Sonic Hedgehog Signaling	0.221	0.0345	#NUM!	PRKAG2
Ubiquinol-10 Biosynthesis (Eukaryotic)	0.204	0.0323	#NUM!	MICAL2
Dopamine Degradation	0.196	0.0312	#NUM!	SULT1A3/SULT1A4
Neurovascular Coupling Signaling Pathway	0	0.031	#NUM!	ADORA2B,APP,CALM1 (includes others),GJA1,KCNJ16,PLAAT4,PRKAG2
Synaptic Long Term Depression	0	0.0255	#NUM!	GNAI1,GNAI2,PLAAT4,PPP2R2A,PRKCI
Ceramide Signaling	0	0.022	#NUM!	PPP2R2A,RELA
Role of NFAT in Regulation of the Immune Response	0	0.0254	#NUM!	ATF2,CALM1 (includes others),CSNK1D,GNAI1,GNAI2,GNB1,HLA-A,HLA-B,HLA-DMA,HLA-DRA,HLA-DRB1,KPNA2,NFKBIA,RCAN2,RELA
CCR5 Signaling in Macrophages	0	0.01	#NUM!	CALM1 (includes others),GNAI1,GNAI2,GNB1,PRKCI
Calcium-induced T Lymphocyte Apoptosis	0	0.0174	#NUM!	ATP2A2,CALM1 (includes others),HLA-A,HLA-B,HLA-DMA,HLA-DRA,HLA-DRB1,PRKCI
Cytotoxic T Lymphocyte-mediated Apoptosis of Target Cells	0	0.00706	#NUM!	HLA-A,HLA-B,HLA-C
CTLA4 Signaling in Cytotoxic T Lymphocytes	0	0.028	#NUM!	AHR,AP2B1,ARF1,BCL2L1,HLA-A,HLA-B,HLA-C,HLA-DMA,HLA-DRA,HLA-DRB1,HMOX1,ITGA3,ITGB1,MALT1,PPP2R2A,RAC1,VAV3
T Helper Cell Differentiation	0	0.0191	#NUM!	HLA-A,HLA-B,HLA-DMA,HLA-DRA,HLA-DRB1,IL18,IL6,STAT1,TGFBR2
CD28 Signaling in T Helper Cells	0	0.0251	#NUM!	ACTR2,ARPC2,CALM1 (includes others),CDC42,HLA-A,HLA-B,HLA-DMA,HLA-DRA,HLA-DRB1,MALT1,NFKBIA,RAC1,RELA
IL-15 Signaling	0	0.00756	#NUM!	BCL2L1,CXCL8,JAK1,RELA
Angiopoietin Signaling	0	0.0263	#NUM!	NFKBIA,RELA
Neuropathic Pain Signaling In Dorsal Horn Neurons	0	0.0198	#NUM!	PRKAG2,PRKCI
ICOS-ICOSL Signaling in T Helper Cells	0	0.0178	#NUM!	CALM1 (includes others),HLA-A,HLA-B,HLA-DMA,HLA-DRA,HLA-DRB1,NFKBIA,RAC1,RELA
Lipid Antigen Presentation by CD1	0	0.00482	#NUM!	AP2B1,PSAP
DNA Methylation and Transcriptional Repression Signaling	0	0.0179	#NUM!	RELA,TP53
Antiproliferative Role of Somatostatin Receptor 2	0	0.013	#NUM!	GNB1
Growth Hormone Signaling	0	0.0282	#NUM!	PRKCI,STAT1
Type I Diabetes Mellitus Signaling	0	0.0236	#NUM!	HLA-A,HLA-B,HLA-C,HLA-DMA,HLA-DRA,HLA-DRB1,IL1R1,JAK1,MYD88,NFKBIA,RELA,STAT1
Allograft Rejection Signaling	0	0.0123	#NUM!	HLA-A,HLA-B,HLA-C,HLA-DMA,HLA-DRA,HLA-DRB1
Autoimmune Thyroid Disease Signaling	0	0.0131	#NUM!	HLA-A,HLA-B,HLA-C,HLA-DMA,HLA-DRA,HLA-DRB1

Graft-versus-Host Disease Signaling	0	0.0179	#NUM!	HLA-A,HLA-B,HLA-C,HLA-DMA,HLA-DRA,HLA-DRB1,IL18,IL6
Communication between Innate and Adaptive Immune Cells	0	0.00857	#NUM!	CXCL8,HLA-A,HLA-B,HLA-C,HLA-DRA,HLA-DRB1,IL18,IL6
Systemic Lupus Erythematosus Signaling	0	0.0127	#NUM!	HLA-A,HLA-B,HLA-C,IL18,IL6,RNU6-1,RNU6-15P,SF3B4
FAK Signaling	0	0.025	#NUM!	ACTR2,ADORA2B,ARPC2,CAPN1,CAPNS1,CCND1,CTNNB1,F2RL1,FZD6,GPR108,GPR143,GPRC5A,IL1R1,ITGA3,ITGAV,ITGB1,ITGB3,ITGB4,ITGB8,MET,MMP14,NPFFR2,RAC1,RXFP1,TGFBR2,TP53
Altered T Cell and B Cell Signaling in Rheumatoid Arthritis	0	0.0202	#NUM!	CSF1,HLA-A,HLA-B,HLA-DMA,HLA-DRA,HLA-DRB1,IL18,IL6,RELA,SPP1
Leptin Signaling in Obesity	0	0.0132	#NUM!	PRKAG2
Regulation of IL-2 Expression in Activated and Anergic T Lymphocytes	0	0.0173	#NUM!	CALM1 (includes others),MALT1,NFKBIA,RAC1,RELA,TGFBR2,TOB1,VAV3
Granzyme A Signaling	0	0.0267	#NUM!	H1-5,NDUFS5
NUR77 Signaling in T Lymphocytes	0	0.0195	#NUM!	CALM1 (includes others),HLA-A,HLA-B,HLA-C,HLA-DMA,HLA-DRA,HLA-DRB1,PCNA,PRKCI,TNFSF10
PKCθ Signaling in T Lymphocytes	0	0.0179	#NUM!	HLA-A,HLA-B,HLA-DMA,HLA-DRA,HLA-DRB1,MALT1,NFKBIA,RAC1,RELA,VAV3
Role of MAPK Signaling in the Pathogenesis of Influenza	0	0.0244	#NUM!	ATF2,PLAAT4
Antiproliferative Role of TOB in T Cell Signaling	0	0.00704	#NUM!	CCNA2,TGFBR2,TOB1
OX40 Signaling Pathway	0	0.0188	#NUM!	BCL2L1,HLA-A,HLA-B,HLA-C,HLA-DMA,HLA-DRA,HLA-DRB1,NFKBIA,RELA
Intrinsic Prothrombin Activation Pathway	0	0.0238	#NUM!	KLK10
Cell Cycle Control of Chromosomal Replication	0	0.0179	#NUM!	PCNA
Role of JAK1 and JAK3 in γc Cytokine Signaling	0	0.029	#NUM!	JAK1,STAT1
Telomerase Signaling	0	0.0278	#NUM!	HSP90AA1,PPP2R2A,TP53
Hematopoiesis from Pluripotent Stem Cells	0	0.00674	#NUM!	CSF1,CXCL8,IL6
VEGF Family Ligand-Receptor Interactions	0	0.0238	#NUM!	NRP1,PRKCI
ERB2-ERBB3 Signaling	0	0.0154	#NUM!	CCND1
GDNF Family Ligand-Receptor Interactions	0	0.0263	#NUM!	CDC42,RAC1
Netrin Signaling	0	0.0278	#NUM!	PRKAG2,RAC1
Heparan Sulfate Biosynthesis	0	0.0227	#NUM!	CHST15,SULT1A3/SULT1A4
Heparan Sulfate Biosynthesis (Late Stages)	0	0.0247	#NUM!	CHST15,SULT1A3/SULT1A4
Pyrimidine Ribonucleotides De Novo Biosynthesis	0	0.025	#NUM!	CMPK1
tRNA Splicing	0	0.0217	#NUM!	PDE1C
Pyridoxal 5'-phosphate Salvage Pathway	0	0.0308	#NUM!	CSNK1D,PLK1
Phospholipases	0	0.0299	#NUM!	HMOX1,PLAAT4
Glutathione-mediated Detoxification	0	0.027	#NUM!	GSTP1
tRNA Charging	0	0.0256	#NUM!	GARS1
Nicotine Degradation II	0	0.0303	#NUM!	POR,UGT1A9 (includes others)
Retinol Biosynthesis	0	0.0213	#NUM!	AKR1C3
Salvage Pathways of Pyrimidine Ribonucleotides	0	0.0309	#NUM!	CMPK1,CSNK1D,PLK1
Pyrimidine Ribonucleotides Interconversion	0	0.027	#NUM!	CMPK1
Noradrenaline and Adrenaline Degradation	0	0.027	#NUM!	ADH6
Acetone Degradation I (to Methylglyoxal)	0	0.0233	#NUM!	POR
Oxidative Ethanol Degradation III	0	0.0175	#NUM!	ACSL1
Fatty Acid β-oxidation I	0	0.0286	#NUM!	ACSL1
Gαi Signaling	0	0.0286	#NUM!	GNAI1,GNAI2,GNB1,PRKAG2
STAT3 Pathway	0	0.0296	#NUM!	FGFR2,IL1R1,RAC1,TGFBR2
Oxidative Phosphorylation	0	0.00901	#NUM!	NDUFS5
SAPK/JNK Signaling	0	0.012	#NUM!	ATF2,CDC42,DUSP4,GNB1,RAC1,TP53
IL-2 Signaling	0	0.0164	#NUM!	JAK1
B Cell Receptor Signaling	0	0.0236	#NUM!	ATF2,BCL2L1,CALM1 (includes others),CDC42,CFL1,CREB3,CREB5,CREBBP,INPPL1,MALT1,NFKBIA,PIK3AP1,RAC1,RELA,VAV3
Dopamine Receptor Signaling	0	0.025	#NUM!	PPP2R2A,PRKAG2
Notch Signaling	0	0.0263	#NUM!	PSENEN

NF-κB Signaling	0	0.0193	#NUM!	CREBBP,FGFR2,IL18,IL1R1,MALT1,MYD88,NFKBIA,RELA,TGFBR2,TNFAIP3,UBE2V1
T Cell Receptor Signaling	0	0.0292	#NUM!	ATF2,CALM1 (includes others),CTNNB1,DUSP5,HLA-A,HLA-B,HLA-C,HLA-DMA,HLA-DRA,HLA-DRB1,ICAM1,ITGA3,ITGB1,MALT1,NFKBIA,RAC1,RELA,VAV3
Gustation Pathway	0	0.0197	#NUM!	LIPH,LRRC8A,PRKAG2,SCNN1A
Cancer Drug Resistance By Drug Efflux	0	0.0172	#NUM!	TP53
Adrenomedullin signaling pathway	0	0.0302	#NUM!	C3,CALM1 (includes others),HIF1A,IL18,PRKAG2,RELA
Th17 Activation Pathway	0	0.0186	#NUM!	AHR,CCL20,HIF1A,HSP90AA1,IL1R1,IL6,JAK1,MYD88,RELA
NER (Nucleotide Excision Repair, Enhanced Pathway)	0	0.00962	#NUM!	PCNA
SPINK1 General Cancer Pathway	0	0.029	#NUM!	IL6,JAK1
T Cell Exhaustion Signaling Pathway	0	0.0229	#NUM!	HLA-A,HLA-B,HLA-C,HLA-DMA,HLA-DRA,HLA-DRB1,IL6,JAK1,LGALS9,MGAT5,PPP2R2A,STAT1,TGFBR2
Systemic Lupus Erythematosus In B Cell Signaling Pathway	0	0.029	#NUM!	BCL2L1,CALM1 (includes others),CCND1,CCND3,CTNNB1,CXCL8,IL18,IL6,INPPL1,JAK1,MALT1,MCL1,MYD88,PIK3AP1,PLAAT4,PRKCI,RAC1,RELA,STAT1,STING1,TNFSF10
Kinetochore Metaphase Signaling Pathway	0	0.027	#NUM!	H2AC18/H2AC19,MACROH2A1,PLK1
Eicosanoid Signaling	0	0.0294	#NUM!	AKR1C3,PLAAT4
